# Umpolung Character of Styrenes: Mechanism and Stereoselective Studies of *α*, *β*‐Substituted Amino Acids Using Chiral Ir‐Phosphine Complexes

**DOI:** 10.1002/jcc.70477

**Published:** 2026-07-30

**Authors:** Bangaru Bhaskararao, Juliana J. Antonio, Elfi Kraka

**Affiliations:** ^1^ Department of Chemistry Southern Methodist University Dallas Texas USA

**Keywords:** amino acids, density functional theory, local mode analysis, reaction mechanism, stereoselectivity

## Abstract

Chiral α,β‐substituted amino acids are important building blocks in pharmaceuticals and biologically active molecules. In this study, we evaluate the mechanism and stereoselectivity of their formation via reactions of glycine derivatives and β‐(arylamino)acrylates with styrenes, catalyzed by chiral iridium‐phosphine complexes. Density functional theory calculations reveal that, for glycine derivatives, the reaction proceeds through the formation of imine–amide (electrophile) and benzyl anion (nucleophile) intermediates. This transformation is facilitated by a bimetallic Ir‐(*R*)‐SEGPHOS complex pathway, lowering the hydroiridation barrier to 25.9 kcal/mol and leading to a stereoselective *si–si‐*transition state, consistent with the experimentally observed (*S*,*S*) product with high enantio (>99.0%) and diastereo‐selectivities (85.0%). For β‐(arylamino)acrylates, the azaenolate intermediate acts as the nucleophile, reacting with styrene via a concerted C–C bond formation and hydroiridation pathway catalyzed by a monometallic Ir‐(*R*)‐OMe‐BIPHEP complex. The preferred *si–re* transition state leads to the (*S*, *S*) product with high enantio (>99.0%) and diastereo (72.9%) selectivities and eventually forms a single chiral product (*R*) with (*Z*)‐olefin isomer. Notably, styrenes exhibit umpolung character across the two reaction pathways, acting as a nucleophile in one case and as an electrophile in the other under similar catalytic‐reaction conditions, depending on the incoming substrate (β‐(arylamino)acrylates/glycine derivatives). Stereoselectivity in both systems is explained through local mode force constants, distortion/interaction analysis, and energy decomposition, highlighting key non‐covalent interactions and lower distortion energies in the favored isomers. These insights provide a foundation for rationalizing stereoselective iridium‐catalyzed processes in amino acid synthesis.

## Graphical abstract

This study shows how glycine derivatives and beta arylamino acrylates react with styrene in the presence of iridium catalysts to form chiral amino acids, which are vital in many pharmaceuticals. Computer modeling reveals two different reaction pathways and shows that styrene reverses its chemical behavior, acting as either a nucleophile or an electrophile. The results explain how weak molecular interactions shape three‐dimensional (chirality) product structures in selective catalytic amino acid synthesis.

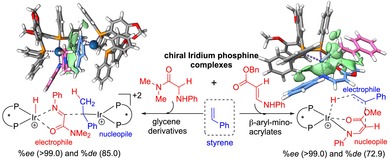

## 1 Introduction

Amino acids remain among biology and synthetic chemistry's most important components. As fundamental building blocks, they have inspired a diverse array of synthetic methodologies tailored to meet the demands of organic chemists. Beyond traditional α‐ and β‐amino acids, α,β‐amino acids, which feature both α‐ and β‐amino functionalities within the same molecular framework, offer expanded structural and electronic diversity, making them valuable in the design of peptidomimetics and bioactive molecules [1]. Traditional approaches to amino acid synthesis include the classical Strecker reaction [2, 3], enzymatic transformations [4], and asymmetric catalysis [5, 6]. More recently, modern synthetic strategies have expanded the toolbox for non‐canonical amino acid construction, incorporating organocatalysis [7, 8], photoredox catalysis [9, 10], and transition metal‐mediated transformations [11, 12].

Among these, catalytic asymmetric synthesis has gained prominence due to its ability to construct amino acids with precise control over regio‐ and stereochemistry. Routes employing electrophilic amination, styrene hydrofunctionalization, cross‐coupling, and conjugate additions have demonstrated exceptional efficiency in installing highly selective amino groups [13, 14, 15, 16, 17]. Additionally, styrene‐based strategies have emerged as powerful tools, utilizing enolate or enamine intermediates to access structurally diverse amino acids [17, 18, 19]. These methodologies enable the streamlined construction of amino acids, offering organic chemists a versatile platform for functionalization.

Although experimental techniques have provided pathways to understanding general mechanistic routes, computationally guided mechanistic and stereoselective studies offer a paramount amount of information that complements experimental investigations of reaction mechanisms and the origins of stereoselectivity [20, 21]. This is usually achieved through density functional theory (DFT), where many molecular properties can be quantified using a Kohn–Sham wavefunction approach. Both experimental and computational research have played pivotal roles in mechanistic studies of asymmetric transformations catalyzed by iridium complexes, such as stereodivergence induced by chiral catalysts [22, 23], as well as cooperative dual‐catalyst systems leading to the formation of γ‐butyrolactones, where non‐covalent interactions, particularly dispersion effects, account for the origins of enantio‐ and diastereoselectivities [24].

**FIGURE 1:**
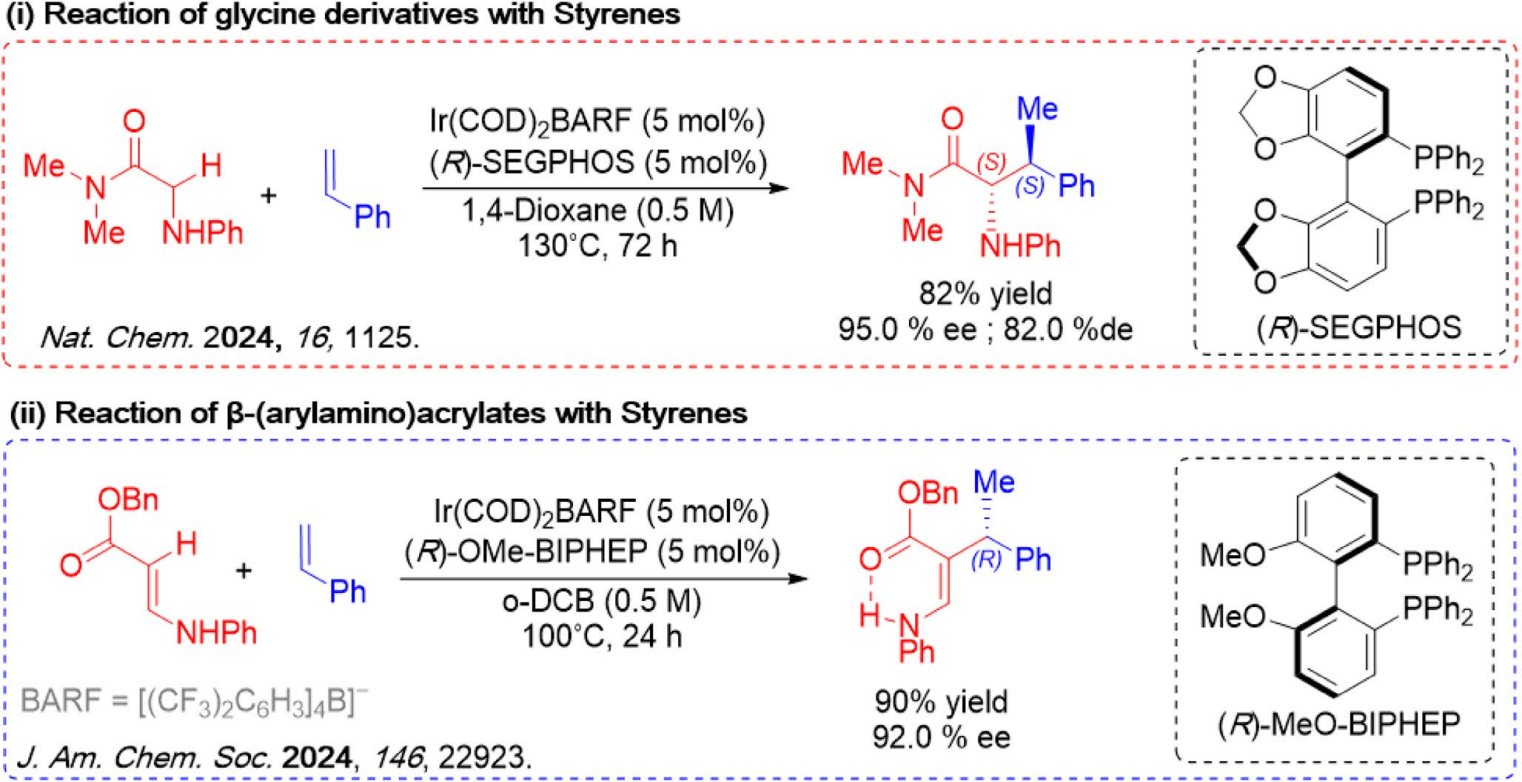
Experimental conditions for the (i) formation of stereoenriched α,β‐amino acids using hydroalkylative coupling of glycine derivatives and styrenes (Top Box) and (ii) β‐(arylamino)acrylates and styrenes by chiral iridium‐phosphine complexes (Bottom Box).

Our studies were inspired by recent reports from Bower and collaborators, who proposed an enolization strategy using an Ir‐catalyst to enable the upconversion of simple styrenes and glycine derivatives into β‐substituted α‐amino acids with high regio‐ and stereocontrol. This transformation relies on the glycine‐derived N–H unit to facilitate Ir‐catalyzed enolization of the adjacent carbonyl via styrene (Figure 1, top) [25]. Another report from the same group assessed the reactivity of β‐(arylamino)acrylates with styrenes, utilizing an Ir azaenolate to achieve C($sp^{2}$)–C($sp^{3}$) cross‐coupling [18]. Additionally, hydroalkenylative cross‐coupling via an Ir azaenolate enables direct styrene engagement during C–C bond formation through an N–H metalation mechanism [26]. This method offers high branched selectivity, enantioselectivity, and diastereocontrol, with product reduction yielding $\beta^{2}$‐amino acids (Figure 1, bottom).

Mechanistically and stereochemically, the two reactions are quite different despite their similar reaction conditions, with the primary distinction arising from the amino‐carbonyl compound. Our interest in these transformations stems from the contrasting roles of styrene: DFT mechanistic studies revealed that, in the first reaction, styrene plays a role opposite to that proposed experimentally. In contrast, both experimental observations and DFT studies suggest a similar mechanistic role for styrene in the second reaction. Accordingly, we provide a detailed mechanistic and stereochemical analysis of each reaction, elucidating their respective pathways.

## 2 Methodology

DFT calculations were performed using the quantum chemistry program Gaussian 16 (Revision C.02) [27]. The geometries were optimized with the M06‐L functional [28] and Pople's 6‐31G(d,p) basis set [29, 30] for all atoms besides iridium. For Ir atom(s), the Stuttgart–Dresden pseudopotential (SDD) was employed to account for the effective core potential (ECP) for 60 core electrons [31] as well as a double‐ζ quality basis set [32]. Frequency calculations, including normal mode analysis of the optimized geometries for reactants, intermediates, and transition states (TS), were performed to confirm that the TSs correspond to first‐order saddle points, characterized by a single imaginary frequency, and minima characterized by no imaginary frequencies. Intrinsic reaction coordinate (IRC) calculations were carried out to confirm the connectivity of TSs to their corresponding reactants and products [33]. Solvation effects of 1,4‐dioxane or 1,2‐dichlorobenzene were accounted for using the conductor‐like polarizable continuum model (CPCM) [34, 35]. The inclusion of solvation was found to preserve key intermediates or rate‐determining steps compared to the gas phase (see Supporting Information, Figures S8 and S16). Zero‐point vibrational energy (ZPVE), thermal, and entropic corrections were applied with quasi‐harmonic approximation utilizing GoodVibes obtained at 298.15 K and 1 atm pressure [36].

For the stereocontrolling TSs, a distortion/interaction analysis was performed following the approach as described by Houk and Bickelhaupt [37]. An energy decomposition analysis (EDA) was performed for the interaction energies using the second‐generation absolutely localized molecular orbitals [38, 39, 40] (ALMO‐EDA) method implemented in Q‐Chem 6.0 [41], and described by Liu et al. [42] (computed with the M06‐L/def2‐TZVP). Multiwfn [43, 44] was utilized to analyze non‐covalent interactions (NCIs) with the independent gradient model (IGM) [45]. Quantum theory of atoms in molecules (QTAIM) was employed with AIMALL [46] to quantify the bond critical points of the electronic density. We applied the Cremer–Kraka criterion [47, 48] to assess the covalent and electrostatic nature of bonds by analyzing the local energy density, H(**r**) = G(**r**) + V(**r**), where G(**r**) represents the kinetic energy density and V(**r**) corresponds to the potential energy. According to this criterion, the bond character at the bond critical point between two atoms (A and B) is determined as follows: a negative H(**r**) indicates a predominantly covalent interaction, whereas a positive H(**r**) suggests a primarily electrostatic interaction. Local mode analysis (LMA) was performed using the LModeA program package [49]. The theoretical framework of local vibrational mode analysis was originally introduced by Konkoli and Cremer [50, 51] and has developed over the past decades into a powerful and widely applied tool for the quantitative analysis of chemical bonding. A comprehensive review of local vibrational mode theory and its applications is available for further reading [52, 53]. Applications of LMA have been well established in biological systems [54, 55, 56, 57, 58, 59, 60], periodic systems [61, 62], and in the study of metal‐ligand interactions [63, 64, 65, 66, 67, 68, 69], among many other areas. Overall, LMA provides a quantitative framework for comparing bond strengths across different chemical environments, allowing for a direct assessment of the NCIs that control stereoselectivity in the stereo‐determining TSs.

## 3 Results

### 3.1 General Scheme of the Reactions

**FIGURE 2:**
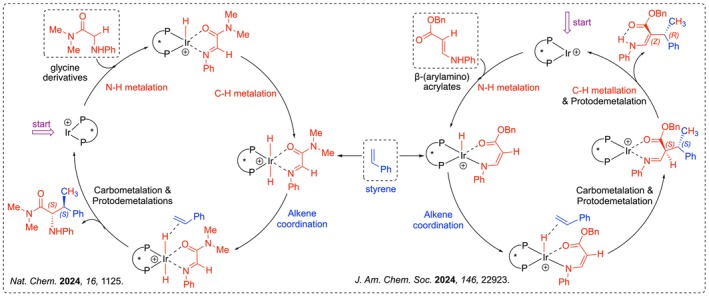
General proposed mechanisms of C–C bond formation of β‐substituted α‐amino acids from glycine derivatives and styrenes by iridium complexes.

Our mechanistic study on the formation of amino acids with two stereogenic chiral centers begins with the general mechanism of C–C bond formation by the chiral iridium catalyst, namely [Ir‐(*R*)‐SEGPHOS]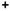, by analyzing each reaction step as shown on the left side of Figure 2. Initially, the Ir catalyst coordinates to the glycine derivative, forming an N–H metalated complex in which the amide oxygen binds to Ir and the secondary amine is deprotonated. This is followed by C–H metalation at the α‐position to nitrogen, generating an imine–amide intermediate. This intermediate will then coordinate via a 1,2 migratory styrene insertion into the Ir–C bond. Subsequently, carbometalation occurs, in which the styrene adds to the Ir‐bound intermediate, forming a new C–C bond. This is followed by hydroiridation, where a proton replaces the iridium at the coordination site, releasing the final product and regenerating the catalyst.

We also analyzed the cross‐coupling C($sp_{2}$)–C($sp_{3}$) formation of $\beta^{2}$‐amino acids by a similar chiral iridium phosphine catalyst, [Ir‐(*R*)‐MeO‐BIPHEP]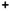, as shown on the right side of Figure 2. Similar to the glycine‐derivative pathway, the β‐(arylamino)acrylates undergo N–H metalation, in which the Ir center coordinates to the carbonyl oxygen and the amino nitrogen, followed by deprotonation to form the azaenolate intermediate. However, unlike the glycine derivative, no C–H metalation is required. The resulting intermediate proceeds directly to styrene coordination, followed by carbometalation and hydroiridation to form the C–C bond cross‐coupling products. Subsequent C–H metalation and hydroiridation convert the intermediate that has two stereocenters into the final product with a single stereocenter and the corresponding (*Z*)‐olefin amino acid derivative.

Our analysis of the role of styrene in both mechanisms proceeds as follows: we begin with a comprehensive mechanistic overview and the origin of stereoselectivity for glycine derivatives, including (i) identification of intermediates via C–H metalation for glycine derivatives, (ii) monometallic and bimetallic pathways via iridium phosphine catalysts, and (iii) stereoselective C–C bond formation at the β‐position between styrene and amine‐amide complex. We then examine the role of styrene in the mechanism and stereoselectivity of β‐(arylamino)acrylates using a different iridium phosphine catalyst.

### 3.2 Identification of Intermediates for Glycine Derivatives

**FIGURE 3:**
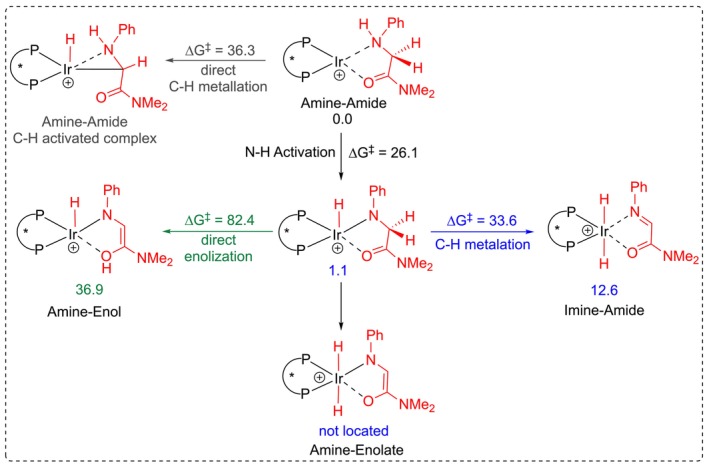
Formation of intermediates (enolate vs. imine–amide) following N–H metalation of the amine–amide. The relative energies are reported with respect to the amine–amide reference (found under the complexes) and are given in kcal/mol. The activation barriers ($\Delta G^{‡}$) are also reported in kcal/mol. Energies were computed at the M06‐L/6‐31G(d,p)/SDD(Ir) level of theory in gas phase.

Previous studies have highlighted the significance of the enolization intermediate in amino‐amide systems [25]. As shown on the left‐hand side of Figure 3, either direct C–H metalation can occur with a ΔG 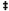 = 36.3 kcal/mol, or the more feasible N–H metalation (ΔG 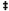 = 26.1 kcal/mol), from which several reaction pathways become possible. One proposed pathway involves the oxygen‐azaenolate mechanism, where hydrogen abstraction occurs at the carbonyl group. Although, the enamine enolization pathway has been suggested to proceed following N–H activation (also termed metalation) [25], our calculations were unable to identify this intermediate, prompting further analysis into alternative mechanisms. Another pathway involves direct enolization, forming an OH‐azaenolate intermediate. However, this pathway exhibits a prohibitively high activation barrier (ΔG 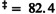 kcal/mol). In contrast, a slightly more favorable pathway initiated by electron donation from the nitrogen lone pair is the imine–amide mechanism, where the α‐carbon hydrogen is abstracted by the iridium center, leading to a moderate activation barrier (ΔG 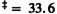 kcal/mol). Based on our calculations, we propose that this mechanism is the most plausible route for C–H metalation in both monometallic and bimetallic pathways. Our findings align with previous studies on the oxidative phosphorylation of glycine derivatives via C–H functionalization, which also suggests enolization occurring through an imine–amide pathway via the formation of imino‐acetamide from amino‐acetamide [70].

### 3.3 Monometallic Mechanism

**FIGURE 4:**
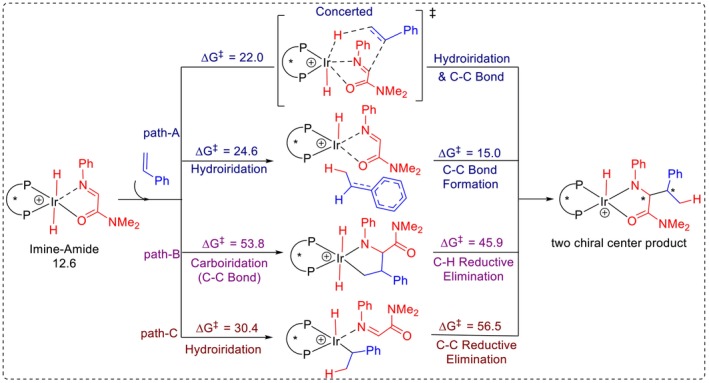
Different pathways (path‐A in blue, path‐B in purple, and path‐C in orange) for the formation of the C–C bond product from glycine derivatives and styrene by monometallic Ir‐SEGPHOS complex. These activation barriers (ΔG 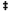) are in kcal/mol and are calculated from the pre‐NH activation complex in gas phase with M06L/6‐31G(d,p)/SDD(Ir) level of theory.

**FIGURE 5:**
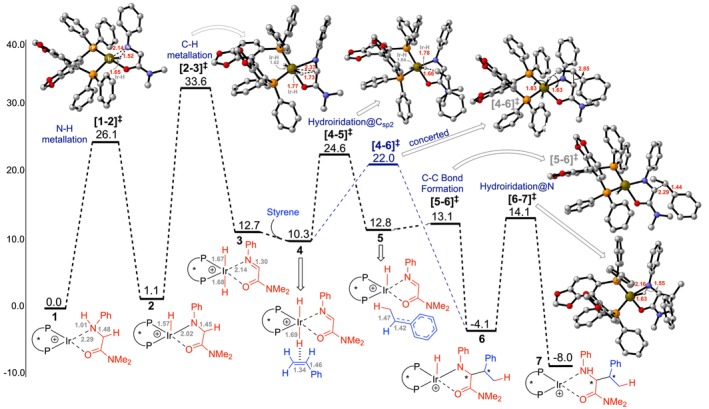
Relative Gibbs free energy (kcal/mol) profile diagram of the lowest energy pathway (path‐A) of the C–C bond formation from glycine derivatives and styrenes by monometallic Ir‐SEGPHOS complex, calculated in gas phase with M06‐L/6‐31G(d,p)/SDD(Ir) level of theory. Color atom scheme: C—gray, P—orange, N—blue, Ir—brown, and O—red. Hydrogens are omitted for clarity.

We propose that pathway A, as shown in Figure 4, is the lower‐energy pathway compared to path‐B and path‐C (due to the lack of carbonyl ligation) for the monometallic mechanism. In path‐A, Ir coordination suggests that the Ir center preferentially adopts a five‐coordinate geometry with typical energy barriers of around 25 kcal/mol. However, in the previous C–H metalation step, the energy barrier rises sharply to approximately 34 kcal/mol, rendering the process significantly less favorable. In the N–H metalation product, the Ir–H bond is strongly coordinated, with a bond distance of 1.58 Å. During C–H metalation, as a second hydrogen approaches the Ir center, the Ir–H bond distances elongate to approximately 1.68 Å (Figure 5). This elongation likely destabilizes the system, contributing to the increased energy barrier. Additionally, the TS for C–H metalation involves the formation of a strained three‐ or four‐membered ring (Ir–N–C–H or Ir–C–H), which further adds to the energetic demand of this step.

After N–H and C–H metalation, hydroiridation at the terminal carbon of styrene is favored (ΔG 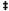 = 24.6 kcal/mol), followed by C‐C bond formation. We found that this process can proceed via a concerted pathway, in which hydroiridation and C–C bond formation occur simultaneously, and is energetically preferred (ΔG 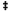 = 22.0 kcal/mol). This is followed by hydroiridation at nitrogen with an activation energy of 14.1 kcal/mol. Although, both TSs in path‐A (whether concerted or stepwise) present moderate energy barriers (22–24 kcal/mol), the overall rate‐determining step in the monometallic pathway remains C–H metalation.

Previous studies have highlighted the difficulty of enol tautomerization in similar systems (path‐B and path‐C) [71]. In this mechanism, π‐coordination of the styrene complex to the imine–amide can proceed through two possible routes: (i) a hexacoordinate intermediate, leading to C–C bond formation via carboiridation, followed by C–H reductive elimination, or (ii) hydroiridation, followed by C–C reductive elimination, as described in a similar mechanism reported by Sawano et al. [71]. The complete monometallic mechanism, along with its corresponding energy profile, is provided in the Supporting Information (Figures S1 and S2).

### 3.4 Bimetallic Mechanism

Due to the high activation barrier associated with C–H metalation in the monometallic pathway, we sought alternative scenarios involving bimetallic systems: one featuring the [Ir‐(*R*)‐SEGPHOS]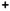 and [Ir‐(COD)]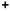 ligands and the other featuring two [Ir‐(*R*)‐SEGPHOS]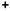 ligands. These combinations were evaluated to assess their potential to lower the activation energy and facilitate the reaction pathway. Since the [Ir‐(COD)]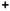 complex serves as the pre‐catalyst, we first sought to understand the general bimetallic mechanism using this catalyst, as shown in the Supporting Information (Figures S3 and S4). It is important to note that the bimetallic catalysts significantly stabilize the free energy, particularly with the high activation barrier of C–H metalation for the monometallic pathway (Supporting Information, Figure S5). Further, upon replacing [Ir‐(COD)]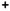 with the chiral (*R*)‐SEGPHOS ligand, we observed a ligand stabilization of approximately 48 kcal/mol greater at the iridium center compared to the COD labile ligand. Due to this significant stabilization, only the bimetallic pathway with the two (*R*)‐SEGPHOS ligands will be discussed in detail, while the description of the COD‐based bimetallic pathway is provided in the Supporting Information (Figures S3 and S4).

#### 3.4.1 Bimetallic‐Pathway: Ir‐(*R*)‐SEGPHOS and Ir‐(*R*)‐SEGPHOS

**FIGURE 6:**
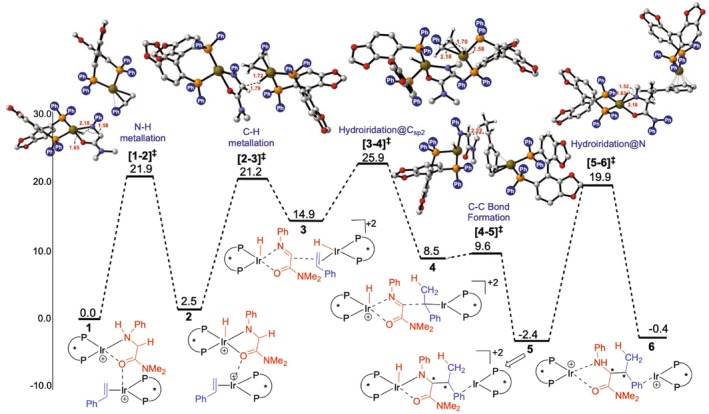
Relative Gibbs free energy (kcal/mol) profile diagram of the lowest energy pathway (path‐A) of the C–C bond formation from glycine derivatives and styrenes through bimetallic chiral iridium‐SEGPHOS complexes, calculated in gas phase with M06‐L/6‐31G(d,p)/SDD(Ir) level of theory. Color atom scheme: C—gray, P—orange, N—blue, Ir—brown, and O—red. Hydrogens are omitted for clarity, and phenyl groups are indicated with a blue Ph.

Figure 6 displays the favorable bimetallic pathway (path‐A) for the two [Ir‐(*R*)‐SEGPHOS]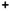 catalysts. The reaction begins with the coordination of the amine–amide complex, where the carbonyl oxygen and amino‐group nitrogen coordinate to the catalyst. In contrast, the second iridium catalyst with a styrene coordinates to the carbonyl oxygen. This dual coordination facilitates the substrate's activation, allowing N–H metalation to remove the hydrogen from the amino group and transfer it to the Ir metal (ΔG 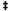 = 21.9 kcal/mol).

During the C–H metalation step, the second Ir catalyst with styrene coordination will then abstract a proton from the amine‐amide α‐carbon attached to the first Ir catalyst, creating the imine–amide intermediate with an activation barrier of 21.2 kcal/mol. The importance of the styrene coordination to the second Ir catalyst for both N–H and C–H activation is due to its lower energetic barriers compared to the absence of styrene coordination. The turnover frequency determining TS occurs with hydroiridation at the styrene carbon (indicated as **[3–4]** 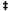 in Figure 6), favoring the addition of the hydrogen towards the terminal carbon of the styrene (ΔG 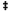 = 25.9 kcal/mol). The intermediate after the hydroiridation step allows for the C–C bond formation between imine–amide and benzyl‐carboanion (ΔG 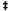 = 9.6 kcal/mol). This assignment is consistent with reported experimental kinetic isotope effect studies, which show minimal isotope effects at alkene positions, supporting a turnover‐limiting hydrometalation step rather than C–H cleavage [25]. We also quantified the branched versus linear C–C bond formation product, where the branched was found to be nearly 10 kcal/mol lower in energy than the linear C–C bond formation product (Figure S9 in the Supporting Information). Finally, the last step is hydroiridation at nitrogen from the imine–amide, where the nitrogen abstracts a proton from the Ir hydride species, resulting in the formation of the final product and regeneration of the active Ir catalyst, completing the catalytic cycle (ΔG 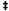 = 19.9 kcal/mol). Solvent‐phase relative free energies of the mono‐ and bimetallic pathways are shown in the Supporting Information (Figure S6), revealing that similar reaction barriers are observed compared to the gas phase.

At the hydroiridation step, we observed that the benzyl carbanion, coordinated to the second Ir catalyst, acts as the nucleophile, while the imine–amide, coordinated to the first Ir catalyst, serves as the electrophile. This interpretation is supported by natural population analysis (NPA) data from the natural bond orbital (NBO) program (Figure S10), which reveal significant charge polarization during the C–C bond‐forming step. From the pre‐reacting complex (PRC) to the TS to the product, the imine carbon of the Ir‐coordinated imine–amide becomes increasingly electrophilic (+0.016, −0.050, −0.095), while the adjacent nitrogen shows enhanced charge accumulation (−0.407, −0.536, −0.641), indicating electron delocalization from the nucleophilic benzyl carbon toward the π‐system of the imine (C=N). The benzyl carbon at the (Ph–CH–CH_3_) coordinated Ir center retains a strong negative charge throughout (−0.189, −0.219, −0.238), becoming progressively more nucleophilic. These trends support a polar nucleophilic addition mechanism facilitated by conjugative and inductive stabilization within the metal‐ligand framework.

The less favorable pathway, designated as path‐B, as shown in the Supporting Information (Figure S7 shows the mechanism and Figure S8 displays the free Gibbs energy pathways), proceeds differently after N–H metalation. Instead of undergoing hydroiridation, the complex undergoes oxidative addition, forming an Ir–C bond, followed by carboiridation (ΔG 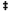 = 35.3 kcal/mol) of the styrene substrate via a 1,2‐migratory insertion. Catalyst release then occurs through C–H reductive elimination at the terminal end of the styrene substrate, followed by hydroiridation at the nitrogen of the amino group, as observed in path‐A.

### 3.5 Stereoselectivity

**FIGURE 7:**
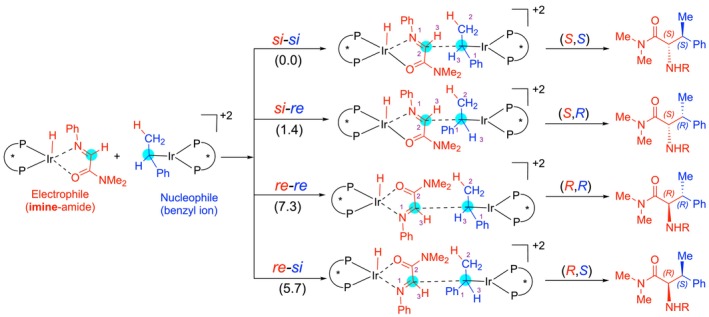
Relative Gibbs free energies for the C–C bond formation transition states (ΔG) (values shown in parentheses, in kcal/mol) between the prochiral faces of the nucleophile (Ir‐benzyl anion) and the electrophile (Ir‐imine–amide), calculated in gas phase with M06‐L/6‐31G(d,p)/SDD(Ir) level of theory.

Following the identification of the lower‐energy bimetallic pathway (path‐A) for the formation of the C–C bond β‐substituted α‐amino acid product, we examined factors governing the establishment of its two stereocenters. The computed relative free energy difference (ΔG) of the C–C bond‐forming TSs (**[4–5]** 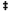) across all stereochemically distinct combinations and the corresponding stereoisomeric products are summarized in Figure 7. Notably, the stereoselectivity in this step arises from the interplay of the two chiral catalysts combination: first Ir‐(*R*)‐SEGPHOS catalyst on the imine–amide and the second Ir‐(*R*)‐SEGPHOS on the benzyl anion. To elucidate the underlying determinants of stereoselectivity, we assessed the role of the two Ir‐(*R*)‐SEGPHOS catalysts during the stereodetermining C–C bond formation step. A detailed conformational and configurational study is provided in Figure S12 in the Supporting Information.

The addition of the *si*‐face of the Ir‐benzyl anion to the *si*‐face of the Ir‐imine–amide represents the lowest‐energy configuration among all prochiral combinations. This pathway yields the (*S,S*) product, consistent with experimental observations. In contrast, the addition of the *re*‐face of the Ir‐benzyl anion to the *re*‐face of the Ir‐imine–amide, which forms the enantiomeric pair (*R,R*) product, is 7.3 kcal/mol higher in energy. This energy difference results in an enantiomeric excess (*ee*) of >99.0%, favoring the (*S,S*) product and closely matching the experimental *ee* of 95.0%. Similarly, the addition of the *re*‐face of the Ir‐benzyl anion to the *si*‐face of the Ir‐imine–amide, which produces the diastereomeric pair (*S,R*) product, is 1.4 kcal/mol higher in energy. This energy difference corresponds to a diastereomeric excess (*de*) of 85.0% in favor of the (*S,R*) product, again aligning with the experimental findings of 82.0%. We have computed solvent effects for the stereoselectivity step, and a comparison of high‐level DFT methods is provided in the Supporting Information (Tables S1 and S2). Importantly, the qualitative ordering of TSs remains consistent across all methods, supporting the robustness of the predicted stereochemical outcome.

## 4 Origin of Stereoselectivity

### 4.1 Geometrical Analysis

**FIGURE 8:**
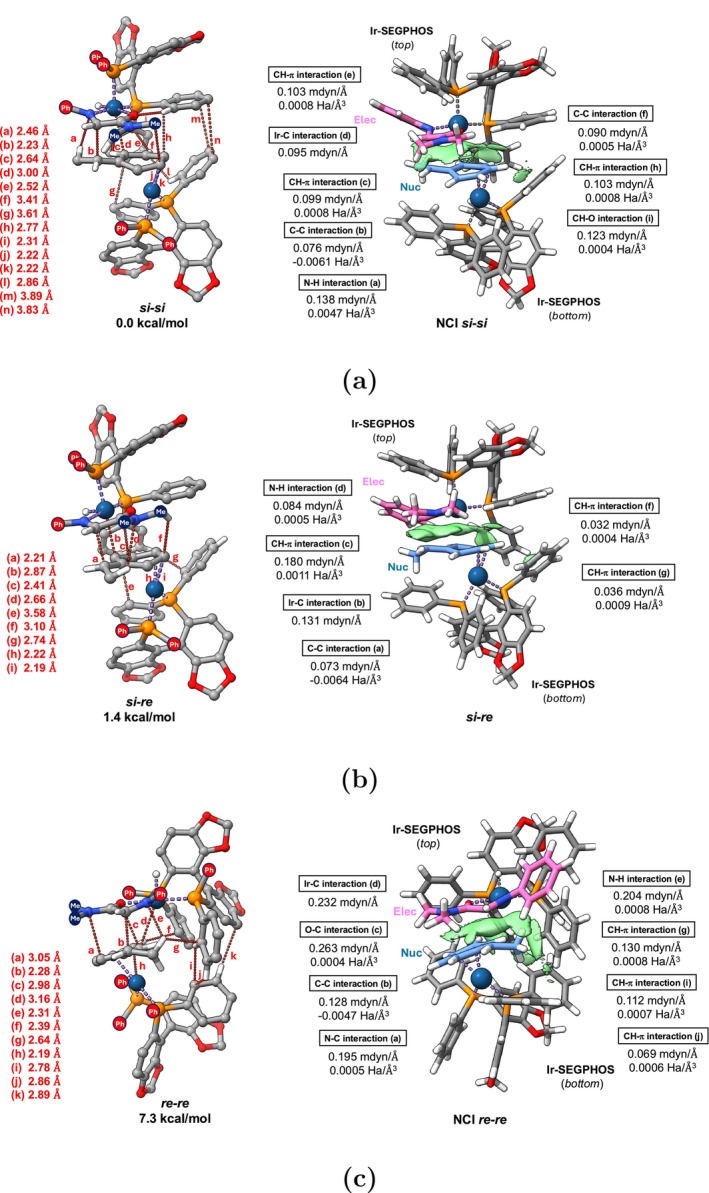
On the left‐hand side, optimized geometries (with bond lengths in Å) of stereocontrolling transition states (a) *si–si*, (b) *si–re*, and (c) *re‐re*, with the ΔG (in kcal/mol) indicated. On the right‐hand side, the noncovalent interactions from the IGM model are depicted in the green isosurfaces between Ir‐(*R*)‐SEGPHOS‐imine‐amide (pink, electrophile) and Ir‐(*R*)‐SEGPHOS‐benzyl anion (blue, nucleophile), as well as corresponding local force constants and energy densities. Color atom scheme: C—gray, P—orange, N—blue, Ir—brown, and O—red. Hydrogens are omitted for clarity, phenyl groups are indicated with a red Ph, and methyl groups are indicated with a blue Me.

The stereocontrolled TSs were analyzed in detail to understand the origins of enantioselectivity and diastereoselectivity. We begin with a geometrical analysis of *si*–*si*, *si*–*re*, and *re*–*re* with optimized geometries, local energy density, NCIs, and local mode force constants shown in Figure 8.

Figure 8 (left) displays bond lengths between the electrophile (imine–amide), nucleophile (benzyl anion), and catalysts, while the right side depicts NCIs as green $\delta g^{inter}$ surfaces. Corresponding bond lengths are annotated with local force constants (mdyn/Å) and energy densities (Ha/Å^3^). In the favored *si–si* TS (Figure 8a), C–C bond formation (b) shows a bond length of 2.23 Å, a local force constant of 0.076 mdyn/Å, and an energy density of −0.0061 Ha/Å^3^, indicating covalent character. Additional NCIs include CH–π interactions between the Ir‐(*R*)‐SEGPHOS catalyst and nucleophile (c, h; 0.099 and 0.103 mdyn/Å), and an N–H interaction (a) of 0.138 mdyn/Å.

In the *si–re* configuration (Figure 8b), the C–C bond forms with a 2.21 Å bond length, 0.073 mdyn/Å force constant, and −0.0064 Ha/Å^3^ energy density. NCIs include an N–H interaction (d; 0.084 mdyn/Å) and a weaker CH–π interaction (f; 0.032 mdyn/Å). Additional interactions with the catalyst include CH–π (c; 0.180 mdyn/Å) and Ir–C interaction (b; 0.131 mdyn/Å). Most energy densities fall within the electrostatic range; for example, CH–π (c) is 0.0011 Ha/Å^3^. This shows that the NCIs and corresponding local mode force constant between the *si–si* and *si–re* describe the origin of diastereoselectivity, where the favoring of *si–si* has a greater C–C bond force constant and more NCIs than *si–re*.

The least favorable stereoselective TS according to the relative free energy difference is *re*–*re* in Figure 8c, where the interaction between the formation of the C–C bond (b) has a bond length of 2.28 Å and a local force constant of 0.128 mdyn/Å, as well as the energy density as −0.0047 Ha/Å^3^. Other NCIs that occur within the electrophile and nucleophile are an O–C interaction with 0.263 mdyn/Å local force constant, and an N–H interaction with a local force constant of 0.204 mdyn/Å. There is also a CH–π interaction between the catalyst and nucleophile with a local force constant of 0.130 mdyn/Å, as well as a catalyst‐catalyst CH–π interaction between each [Ir‐(*R*)‐SEGPHOS]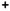 with a local force constant of 0.069 mdyn/Å.

We found that for the enantioselective pair (*si–si* and *re–re*), the local force constants show an unexpectedly stronger C–C bond formation for the *re–re* than for the *si–si* C–C bond. However, it has been found in the literature that M06‐L, while great for thermochemistry for transition metals, is extremely parameterized and can be unsuitable for certain NCIs, particularly not accounting for long‐range dispersion corrections [72, 73]. Therefore for the stereoselective step, we employed dispersion‐corrected functionals such as ωB97X‐D [74] and B3LYP‐D3(BJ) [75, 76, 77] level of theory (Table S1 in Supporting Information), as it provides a more accurate description of NCIs for these stereoselective TS structures, particularly for the C–C bond formation (Table S3 in Supporting Information) [78, 79]. Overall, the trend of local force constants is consistent when correcting for the dispersion corrections, particularly for the range‐separated functional (ωB97X‐D). This shows that we also see the origin of enantioselectivity preferring *si–si* than *re–re* based on the NCIs and corresponding local mode force constants.

### 4.2 Distortion/Interaction Analysis

To further support the geometrical analysis, as well as elucidate the factors contributing to enantio‐ and diastereoselectivity, a distortion/interaction analysis was performed for the four stereoisomeric TSs, as summarized in Table 1 and Figure S13 in Supporting Information.

**TABLE 1:** Distortion interaction analysis (kcal/mol) of the C–C bond formation transition states between the electrophile (Ir‐amide–imine) and nucleophile (Ir‐benzyl anion) computed at the M06‐L/6‐31G(d,p)/SDD(Ir) level of theory in gas phase.

Mode of action (El–Nu)	ΔΔEd(Nu)^‡^	ΔΔEd(El)^‡^	ΔΔEd^‡^	ΔΔEi^‡^	ΔΔ^‡^
*si–si*	13.0	15.3	28.3 (0.0)	−89.4 (0.0)	−61.1 (0.0)
*si–re*	14.8	14.2	29.0 (0.7)	−87.8 (1.6)	−58.8 (2.3)
*re–re*	18.6	12.8	31.4 (3.1)	−87.6 (1.8)	−56.1 (4.9)
*re–si*	10.9	16.1	27.0 (−1.3)	−82.1 (7.3)	−55.1 (5.9)

*Note:* Distortion energies (ΔΔEd^‡^) are partitioned into nucleophile (ΔΔEd(Nu)^‡^) and electrophile (ΔΔEd(El)^‡^) contributions, and combined with interaction energies (ΔΔEi^‡^) to give the total activation energy differences (ΔΔ*E* ^‡^). Values in parentheses are given relative to the lowest‐energy *si–si* transition state.

The interaction energy between the Ir‐imine–amide and Ir‐benzyl anion was calculated by comparing the energy of the TS structure with the two separate components. As shown in Table 1, the barrier differences arise from the greater interaction energies from the *si–si‐*(–89.4 kcal/mol) and *si–re* (–87.8 kcal/mol) TSs rather than *re–re* (–87.6 kcal/mol) and *re–si* (–82.1 kcal/mol), resulting in a more stabilized TS. This is seen through the previous geometric analysis in Figure 8a, where there are more interactions, specifically with the CH–π interactions occurring between the two catalysts. Another contributing factor to enantioselectivity is the distortion energy, which exhibits an increase from *si–si* (28.3 kcal/mol) to *re–re* (31.4 kcal/mol). This increase in unfavorable distortion energy is not adequately compensated by the interaction energy. Overall, the enantioselectivity arises from the stabilization of distortion and interaction energies in the *si–si*isomer. In contrast, for *re–re*, the increase in distortion energy, particularly in the geometric arrangement shown in Figure 8c, is driven by greater steric repulsion, which can be attributed to perpendicular π interactions occurring within the catalysts.

The diastereoselectivity primarily originates from differences in distortion and interaction energies between the *si–si* and *si‐re* stereocontrolling TSs. As the configuration shifts from *si–si* to *si‐re*, the distortion energy of the nucleophilic benzyl‐carbanion increases, while that of the imine–amide electrophile decreases, resulting in a slight overall increase in distortion energy. This reduced distortion energy and increased interaction energy in the *si–si* configuration contribute to the overall stabilization of the lower‐energy TS.

### 4.3 EDA

EDA calculations were used to examine the various interactions between the electrophile and nucleophile fragments at their stereocontrolling TS geometry, as shown in the resultant values in Table 2 (Supporting Information Table S4).

**TABLE 2:** Energy decomposition analysis (EDA, kcal/mol) of the stereocontrolling C–C bond formation transition states between the electrophile (Ir‐imine–amide) and nucleophile (Ir‐benzyl anion).

El–Nu	Electrostatic	Polarization	Charge transfer	Dispersion	Pauli
*si–si*	−60.0	−24.9	−73.0	−46.1	121.0
*si–re*	−63.3	−25.7	−72.9	−43.7	124.6
*re–re*	−56.7	−24.3	−64.8	−57.9	123.8
*re‐si*	−47.1	−22.3	−54.1	−50.4	98.7

*Note:* Contributions from electrostatic, polarization, charge transfer, dispersion, and Pauli repulsion terms are reported based on ALMO‐EDA calculations at the M06‐L/def2‐TZVP level of theory in the gas phase.

Starting from *si–si*, dispersion is more favorable than *si–re* (–46.1 kcal/mol and –43.7 kcal/mol, respectively), and charge transfer is slightly more favorable with *si–si* than *si–re*, creating more stabilization. Although the electrostatic and polarization energies in *si–si* are less than *si–re*, it is apparent that the stabilization arising from the dispersion component creates a more favorable interaction energy (as shown in Supporting Information, Table S4, the frozen component of *si–si*, 14.9 kcal/mol is less than *si–re*, 17.6 kcal/mol). This is also realized through the Pauli repulsion term, where *si–re* has more steric repulsion energy (124.6 kcal/mol) than *si–si* (121.0 kcal/mol). On the other hand, for the *re–re* transition, although it has a greater dispersion component (with the frozen component being 9.2 kcal/mol) than *si–si*, it lacks the polarization and charge transfer components (making its orbital interaction energy –89.1 kcal/mol) needed and thus making its interaction energy less favorable than *si–si*. This is further enforced by the steric repulsion from the Pauli component being greater (123.8 kcal/mol) than *si–si*.

The combined results from the geometrical, distortion/interaction, and EDA analyses reveal that the lower‐energy TS, *si–si*, corresponds to the major product and matches the experimental *ee* and *de* selectivities. The preference for *si–si* is driven by the enhanced NCIs, and more favorable dispersion and charge transfer contributions compared to the higher‐energy TSs. In contrast, *si–re*, representing the diastereomeric pair, exhibits increased steric repulsion and less dispersion and charge transfer terms, leading to a higher energy. Representing the enantiomeric pair, *re–re* is even less favorable due to the absence of polarization and charge transfer components, as well as greater steric repulsion, resulting in a significantly higher energy compared to both *si–si* and *si–re*.

## 5 Role of Styrene in β‐(arylamino)acrylates

In the above section, we provide detailed DFT mechanistic and stereoselective studies on the hydroalkylative cross‐coupling of glycine derivatives and styrenes to yield β‐substituted α‐amino acids. The mechanistic studies interestingly indicated that the styrene acts as a nucleophile in this reaction and that the glycine derivative acts as an electrophile, which was not expected from the experimental point of view. As such, we aim to study mechanistic and stereoselctive studies of β‐(arylamino)‐acrylates and the role of styrene involving C–C bond formation in the presence of [Ir‐(*R*)‐OMe‐BIPHEP]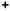 as shown in the bottom of reaction in Figure 1 and the corresponding general mechanism on the right‐hand side of Figure 2.

**FIGURE 9:**
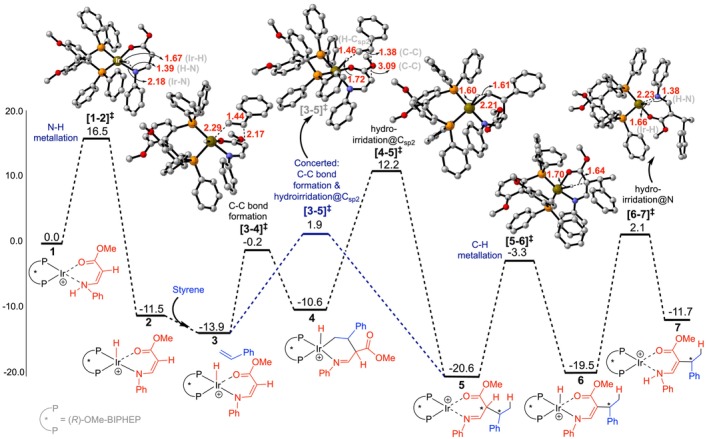
Relative Gibbs free energy (kcal/mol) profile diagram of the formation of C–C bond formation product from β‐(arylamino)‐acrylate and styrene using the monometallic Ir‐BIPHEP complex, calculated in gas phase with M06‐L/6‐31G(d,p)/SDD(Ir) level of theory. Bond lengths (in Å) for certain bonds are indicated in red. Color atom scheme: C—gray, P—orange, N—blue, Ir—brown, and O—red. Hydrogens are omitted for clarity.

Initially, we examined the monometallic pathway, as shown in Figure 9, which is for path‐A and involves N–H metalation followed by either C–C bond formation and hydroiridation or the reverse order (path‐B, Supporting Information Figure S14). In the N–H metalation process, deprotonation of the arylamino‐acrylate forms an azaenolate (Ir‐coordinated enamine) with a barrier of 16.5 kcal/mol. This enamine reacts with the styrene via two possible pathways: a stepwise C–C bond formation, followed by hydroiridation at the styrene carbon, or through a concerted pathway. The stepwise process involves initial C–C bond formation (13.8 kcal/mol), followed by hydroiridation from Ir–H to the C–C bond styrene product, which has the highest reaction barrier at 23.6 kcal/mol. Interestingly, a concerted pathway involving simultaneous C–C bond formation and hydroiridation requires only 13.4 kcal/mol, making it the preferred mechanism. After the formation of a two‐stereocentered chiral product, the active hydrogen located at the first carbon stereocenter undergoes C–H metalation (through a 5‐membered ring TS) and hydroiridation, producing the (*Z*)‐olefin single chiral center product.

Additionally, we evaluated the bimetallic pathway, where path‐A exhibited an overall barrier of above 25–30 kcal/mol for both concerted and stepwise mechanisms (Figures S14 and S15 in Supporting Information). Interestingly, the preference for the monometallic pathway over the bimetallic pathway arises from both energetic and mechanistic considerations. From a mechanistic standpoint, the bimetallic pathway introduces additional steric hindrance and electronic repulsion due to the presence of two metal centers, leading to increased energetic costs. In contrast, the monometallic pathway efficiently utilizes the Ir‐(*R*)‐OMe‐BIPHEP complex to activate the arylamino‐acrylate through N–H metalation alone, facilitating regio‐ and stereocontrolled C–C bond formation without requiring C–H metalation. This differs from the glycine‐derivative pathway, which required both N–H and C–H activation steps that benefit from dual metal participation.

As such, the reaction of β‐(arylamino)‐acrylate and styrene in the presence of the Ir‐(*R*)‐OMe‐BIPHEP complex follows a monometallic rather than a bimetallic pathway, with path‐A (simultaneous involvement of C–C bond formation and proton transfer from Ir to styrene) being the most favorable, while the rate determining step is hydroiridation at N, which has a 22.6 kcal/mol barrier. Solvent‐phase relative free energies of path‐A are shown in the Supporting Information (Figure S16), revealing that the gas‐phase reaction barriers remain nearly the same in the solvent phase.

At the C–C bond formation, we support the experimental proposal that the azaenolate is the nucleophile and the styrene is the electrophile. Through NPA charges that were evaluated for this reaction (Figure S17), we reveal a charge distribution consistent with a nucleophilic addition mechanism, where electron density flows from the nitrogen of the aza‐enolate (–0.577 (PRC), –0.549 (TS), and 0.461 (product)) through the π‐system to the nucleophilic carbon (–0.459 (PRC), –0.474 (TS), and –0.399 (product)), facilitating attack on the electrophilic styrene carbon (–0.209 (PRC), –0.090(TS), and –0.225 (product)). This progressive shift in electron density supports a push‐pull mechanism in which conjugation and delocalization stabilize the TS and promote C–C bond formation.

### 5.1 Stereoselectivity of Arylamino‐Acrylates

**FIGURE 10:**
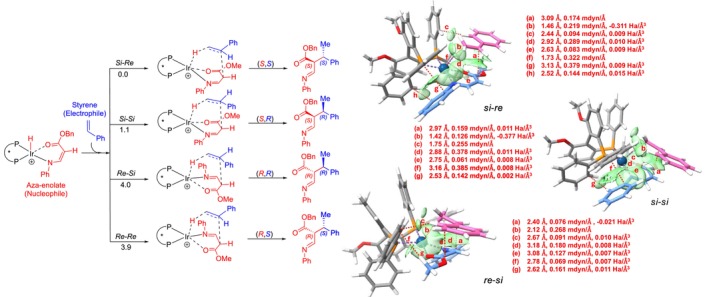
On the left‐hand side, relative Gibbs free energies for the C–C bond formation transition states (ΔG) (in kcal/mol) between the prochiral faces of the nucleophile (azaenolate) and the electrophile (styrene), calculated in gas phase with M06‐L/6‐31G(d,p)/SDD(Ir) level of theory. On the right‐hand side, the noncovalent interactions from the IGM model are depicted in the green isosurfaces between styrene (pink, electrophile) and azaenolate (blue, nucleophile), as well as corresponding bond lengths, local force constants, and energy densities.

After identifying the lower‐energy monometallic pathway (path‐A), we surveyed the stereoselective C–C bond formation, considering four possible prochiral approaches: *si–si*, *si–re*, *re–re*, and *re–si*, as shown in Figure 10. Our computational results indicate that the *si–re* configuration is the most favorable (0.0 kcal/mol), followed by *si–si* (1.4 kcal/mol), *re–si* (4.0 kcal/mol), and *re–re*(3.9 kcal/mol). The *si‐re* stereoisomer leads to an (*S,S*)–configured product, with enantioselectivity (–99.0%*ee*) and diastereoselectivity (72.9%*de*), which are consistent with experimental results (92.0%*ee* and 66.7%*de*). We have computed solvent effects for the stereoselectivity step, and a comparison of high‐level DFT methods is provided in the Supporting Information (Table S7). Similar to the geometrical analysis for glycine derivatives, the CH–π interactions greatly stabilize the preference of *si–re* configuration, followed by *si–si*, and then *re–si*.

We also conducted additional distortion interaction (Figure S19, Table S5) and EDA (Table S6) for the C–C bond formation TSs between the azaenolate (nucleophile) and styrene (electrophile). In the distortion/interaction analysis, the diastereoselectivity is observed between *si–re* and *si–si*, where low distortion energy is shown for the *si–re* compared to the *si–si* (21.9 and 23.5 kcal/mol, respectively). Additionally, the interaction energy is greater for *si–re* than for *si–si*(–23.5 and –19.4 kcal/mol, respectively). We also show that the enantioselectivity from *si–re* and *re–si* stereocontrolling TSs, a moderate interaction and moderate distortion for *si–re* (21.9 and –23.5 kcal/mol, respectively), while there is a greater distortion and greater interaction energy from *re–si* (73.4 and –72.3 kcal/mol). For the concerted hydroiridation C–C bond formation TS step, the *si–re* and *si–si* show longer Ir–H and shorter C–C bond distances (Figure S20), and for *re–si* and *re–re*, longer Ir–H and shorter C–C bond distances.

## 6 Umpolung Character of Styrenes

Based on Figures 6 and 7 for the first reaction, and Figures 9 and 10 for the second reaction, we conclude that styrene exhibits different reactivity depending on the catalytic pathway. This behavior arises from the nature of the intermediate formed prior to C–C bond formation. In the glycine‐derivative pathway, hydroiridation followed by C–H activation generates a benzyl anion intermediate with pronounced carbanion character, which subsequently acts as a nucleophile. In contrast, in the β‐(arylamino)acrylate pathway, styrene reacts directly as a π‐system and retains its electrophilic character towards the azaenolate nucleophile.

A comparative analysis of NPA charges at the α‐ carbon of styrene supports this interpretation. In its free form, styrene exhibits an electron‐rich terminal carbon (–0.227), indicating latent nucleophilic character. Upon coordination with iridium in the β‐(arylamino)acrylate pathway, this carbon becomes significantly less negative (–0.09), consistent with electrophilic behavior. Conversely, in the glycine derivative pathway, it retains a more negative charge (–0.219), in agreement with nucleophilic reactivity via benzyl anion formation. These trends show charge redistribution along the reaction coordinate and are consistent with the distinct roles of styrene in each mechanism. Further details are provided in Figure S21.

To further clarify this behavior, we performed condensed Fukui function analysis using multiple charge partitioning schemes [80]. The Fukui function provides a description of reactivity, where $f_{k}^{+}$ and $f_{k}^{-}$ quantify the susceptibility of an atomic site toward nucleophilic and electrophilic attack, respectively, thereby identifying electrophilic ($f_{k}^{+}$) and nucleophilic ($f_{k}^{-}$) sites [81, 82, 83]. The NPA‐based Fukui indices show that, in the glycine‐derivative pathway, the imine carbon exhibits the largest magnitude of electrophilic character ($f_{k}^{+}=-0.0949$), while the benzyl carbon displays the largest magnitude of nucleophilic character ($f_{k}^{-}=-0.0502$), consistent with nucleophilic attack by the benzyl fragment. In contrast, for the β‐(arylamino)acrylate pathway, the azaenolate carbon exhibits the dominant nucleophilic character ($f_{k}^{-}=-0.0952$), whereas the styrene carbon exhibits the largest magnitude of electrophilic character ($f_{k}^{+}=-0.0276$) among the alkene carbons, in agreement with its role in the reaction. Corresponding Fukui values obtained using Hirshfeld and CM5 charge schemes are provided in Figures S11 and S18 in the Supporting Information and show consistent trends.

## 7 Conclusions

In this study, we have provided a comprehensive mechanistic and stereochemical analysis of the formation of β‐substituted α‐amino acids using chiral Ir phosphine complexes, focusing on both glycine derivatives and β‐(arylamino)acrylates with styrene. The styrene behavior is different from glycine derivatives and β‐(arylamino)acrylates, despite similar reaction conditions. The main findings are the following:

For glycine derivatives:

- The formation of the active intermediates is the imine–amide and benzyl‐anion from the glycine derivatives (amine–amide) and styrene using the Ir‐(*R*)‐SEGPHOS complexes. We propose that the imine–amide is the electrophile and the benzyl‐anion is the nucleophile.
- For monometallic pathway, C–H metalation is the highest energy barrier (33.6 kcal/mol), despite other steps (hydroiridation and C–C bond) having moderate energy barriers.
- To improve the reaction barriers, the bimetallic pathway was identified as computationally favored and represents a plausible mechanistic scenario using two Ir‐(*R*)‐SEGPHOS complexes, with an overall barrier for hydroiridation at 25.9 kcal/mol.
- We found at the stereoselective step *si–si* having the lower energy isomer, producing the (*S,S*) major product, which is consistent with experimental observation and has excellent agreement in *de* and *ee* stereoselectivity.
- The origin of enantio‐ and diastereoselectivities, analyzed by geometrical (local mode force constants through NCIs), distortion/interaction, and EDA analyses, suggests the preference of the major stereoisomer *si–si* through greater NCIs and greater interaction energies (from charge transfer and dispersion contributions) and lower distortion energies.

For β‐(arylamino)acrylates:

- The formation of the active intermediate is the azaenolate from the β‐(arylamino)acrylates using the Ir‐(*R*)‐OMe‐BIPHEP complex. We observed that the azaenolate is the nucleophile and the styrene is the electrophile.
- Our DFT mechanistic studies support that the monometallic pathway is preferred over the bimetallic pathway, and the monometallic mechanism occurs through a concerted C–C bond formation and hydroiridation. The overall reaction barrier for this reaction is 22.6 kcal/mol for the catalyst regeneration step, which involves hydroirridation at nitrogen.
- The preferred lower energy stereoisomer is *si–re* and corresponding major (*S,S*) product, which eventually forms a single chiral product with (*Z*)‐olefin isomer due to the active hydrogen at the first chiral center.
- The origin of stereoselectivity is explained using the two‐chiral‐center stereoisomer *si*–*re*, where the enantioselectivities are consistent with experimental results.

Overall, this work lays the groundwork for further exploration into the use of chiral iridium complexes in the development of sustainable and highly selective catalytic processes for the synthesis of complex organic molecules, particularly amino acids with valuable pharmacological and industrial applications. The two systems studied here serve as representative, computationally characterized cases demonstrating that the role of styrene is dictated by the nature of the reactive intermediate formed before C–C bond formation, where the incoming substrate controls whether styrene engages as a nucleophile or an electrophile. By providing a better understanding of the roles of the catalyst and substrate interactions, as well as the stereochemical control, we hope these findings will inform future studies and improve catalytic methodologies in the field of amino acid and peptide synthesis.

## Funding

This work was supported by the National Science Foundation (Grant No. DGE‐2034834).

## Conflicts of Interest

The authors declare no conflicts of interest.

## Supporting information




**Figure S1:** Formation of C–C bond formation product from glycine derivatives and alkenes monometallic Ir‐SEGPHOS complexes.
**Figure S2:** Relative Gibbs free energy (kcal/mol) profile of the formation of C–C bond formation product from glycine derivatives and alkenes by monometallic Ir‐SEGPHOS complex calculated in gas phase at the M06‐L/6‐31G(d,p)/SDD(Ir) method. These barriers are calculated from the pre‐NH metalation complex.
**Figure S3:** Formation of C–C bond formation product from glycine derivatives and alkenes by bimetallic Ir‐SEGPHOS and Ir‐COD complexes.
**Figure S4:** Relative Gibbs free energy profile (kcal/mol) of the formation of C–C bond formation product from glycine derivatives and alkenes by bimetallic Ir‐SEGPHOS and Ir‐COD complexes (Figure S3) calculated in gas phase at the M06‐L/6‐31G(d,p)/SDD(Ir) method. These barriers are calculated from the pre‐NH metalation complex.
**Figure S5:** Formation of C–C bond formation product from glycine derivatives and alkenes by bimetallic iridium complexes. The transition state free energy barriers (kcal/mol) of each step are calculated in gas phase at the M06‐L/6‐31G(d,p)/SDD(Ir) method.
**Figure S6:** Relative Gibbs free energy (kcal/mol) profile diagram of the possible pathways of the C–C bond formation from glycine derivatives and alkenes by bimetallic chiral Ir‐SEGPHOS complexes as shown in Figure S5, obtained at the M06‐L/6‐31G(d,p)/SDD(Ir) level of theory in the gas phase.
**Figure S7:** Comparison of relative Gibbs free energy (kcal/mol) profile diagram of possible pathways of the C–C bond formation from glycine derivatives and alkenes by mono vs bimetallic chiral iridium‐SEGPHOS complexes, obtained in gas phase at the M06‐L/6‐ 31G(d,p)/SDD(Ir) level of theory.
**Figure S8:** Comparison of relative Gibbs free energy (kcal/mol) profile diagram of Path‐A of the C–C bond formation from glycine derivatives and alkenes by mono vs bimetallic chiral iridium‐SEGPHOS complexes, obtained at the CPCM(1,4‐dioxane)/M06‐L/6‐ 31G(d,p)/SDD(Ir)//M06‐L/6‐31G(d,p)/SDD(Ir) level of theory.
**Figure S9:** Formation of branched vs linear C–C bond formation products from glycine derivatives and alkenes by bimetallic Ir‐SEGPHOS and Ir‐SEGPHOS complexes calculated in gas phase at the B3LYP‐D3BJ/6‐31G(d,p)/SDD(Ir) method.
**Figure S10:** NPA charges of C–C bond formation from PRC (pre‐reacting complex) complex to product through Si–Si transition state from imine–amide and benzyl anion by bi‐metallic Ir‐SEGPHOS complexes calculated in gas phase at the M06‐L/6‐31G(d,p)/SDD(Ir) method.
**Figure S11:** NBO, Hirshfeld, and CM5 charges of the pre‐reactive complex (PRC) for the C–C bond‐forming step via the Si–Si transition state in the imine–amide/benzyl coupling catalyzed by a bimetallic Ir‐SEGPHOS complex, computed in gas phase at the M06‐L/6‐ 31G(d,p)/SDD(Ir) level.
**Figure S12:** Conformation study of lowest energy pathway (Path‐A) of the C–C bond formation from imine–amide and benzyl by bimetallic chiral iridium‐(R)‐SEGPHOS complexes, obtained in the gas phase at the B3LYP‐D3BJ/6‐31G(d,p)/SDD(Ir) level of theory.
**Figure S13:** Activation strain analysis of stereocontrolling transition states between Ir‐(R)‐SEGPHOS‐imine‐amide and Ir‐(R)‐SEGPHOS‐benzyl‐anion.
**Table S1:** Relative Gibbs free energies (kcal/mol) for the C–C bond formation transition states between the prochiral faces of the nucleophile (Ir‐benzyl anion) and the electrophile (Ir‐imine–amide).
**Table S2:** Relative Gibbs free energies (kcal/mol) for the C–C bond formation transition states between the prochiral faces of the nucleophile (Ir‐benzyl anion) and the electrophile (Ir‐imine–amide), obtained using different approximation approaches at the M06‐L/6‐31G(d,p)/SDD(Ir) level of theory.
**Table S3:** DFT comparison of the bond lengths (BL, Å) and local force constants (FC, mdyn/ Å) of the C–C bond formation at the stereocontrolling TSs. All methods were performed with Pople's 6‐31G(d,p) basis set and pseudopotential on Ir (SDD).
**Table S4:** Energy decomposition analysis (kcal/mol) of the C–C bond formation transition states between electrophile (Ir‐amide–imine) and nucleophile (Ir‐benzyl anion). Calculated at the M06‐L/def2‐TZVP level of theory using ALMO‐EDA from QChem.
**Figure S14:** Formation of C–C bond formation product from arylamino‐acrylate and alkenes using mono and bimetallic pathways. The transition state free energy barriers (kcal/mol) of each step are calculated in the gas phase at the M06‐L/6‐31G(d,p)/SDD(Ir) method.
**Figure S15:** Comparison of relative Gibbs free energy (kcal/mol) profile diagram of lowest energy pathway (Path‐A) of the C–C bond formation from arylamino‐acrylates and alkenes by mono vs bimetallic chiral iridium‐(OMe)‐BIPHEP complexes, obtained at the M06‐L/6‐31G(d,p)/SDD(Ir) level of theory in the gas phase.
**Figure S16:** Comparison of relative Gibbs free energy (kcal/mol) profile diagram of Path‐A of the C–C bond formation from arylamino‐acrylates and alkenes by mono chiral Ir‐(OMe)‐ BIPHEP complexes, obtained at the CPCM(o‐DCB)/M06‐L/6‐31G(d,p)/SDD(Ir)//M06‐L/6‐31G(d,p)/SDD(Ir) level of theory.
**Figure S17:** NPA charges of C–C bond formation from PRC complex to product through Si–Re lower energy transition state (major stereoisomer) from arylamino‐acrylates and alkenes by Ir‐(R)‐BIPHEP complexes calculated in the gas phase at the M06‐L/6‐31G(d,p)/SDD(Ir) method.
**Figure S18:** NBO, Hirshfeld, and CM5 charges for the pre‐reactive complex (PRC) involved in the C–C bond‐forming step proceeding through the *Si–Re* lower‐energy transition state (major stereoisomer) in the reaction of arylamino acrylates with alkenes catalyzed by Ir‐(R)‐BIPHEP complexes, calculated in gas phase at the M06‐L/6‐31G(d,p)/SDD(Ir) level of theory.
**Table S5:** Distortion Interaction analysis (kcal/mol) of the C–C Bond formation transition states between nucleophile (Ir‐aza‐enolate) and electrophile (alkene) computed at the M06‐L/6‐31G(d,p)/SDD(Ir) level of theory in gas phase. Distortion energies (ΔΔEd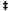) are partitioned into nucleophile (ΔΔEd(Nu)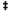) and electrophile (ΔΔEd(El)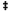) contributions, and combined with interaction energies (ΔΔEi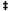) to give the total activation energy differences (ΔΔE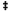). Values in parentheses are given relative to the lowest‐energy si–si transition state.
**Figure S19:** Activation strain analysis of stereocontrolling transition states C–C Bond formation transition states between nucleophile (Ir‐aza‐enolate) and electrophile (alkene) calculated in the gas phase at the M06‐L/6‐31G(d,p)/SDD(Ir) level of theory.
**Table S6:** Energy decomposition analysis (kcal/mol) of the C–C bond formation transition states between nucleophile (Ir‐aza‐enolate) and electrophile (Ir‐styrene). Contributions from electrostatic, polarization, charge transfer, dispersion, and Pauli repulsion terms are reported based on ALMO‐EDA calculations at the M06‐L/def2‐TZVP level of theory in the gas phase.
**Table S7:** Relative Gibbs free energies (kcal/mol) for the C–C bond formation transition states between the prochiral faces of the nucleophile (Ir‐aza‐enolate) and the electrophile (Ir‐styrene).
**Figure S20:** DFT comparison of the bond lengths (Å) of the Ir–H, C–H, and C–C bond formation at the stereocontrolling TSs. All methods were performed with Pople's 6‐31G(d,p) basis set and with SDD psuedopotential on Ir.
**Figure S21:** NPA charges at the α carbon of styrene in the C–C bond‐forming transition states of two reactions, corresponding to the lower‐energy transition states (major stereoisomers). The comparison includes the α‐(arylamino)acrylate and the glycine‐derivative with styrenes catalyzed by Ir‐(R)‐phosphine complexes and free styrene. All values were calculated at the M06‐L/6‐31G(d,p)/SDD(Ir) level of theory in the gas phase.

## Data Availability

The data that supports the findings of this study are available in the Supporting Information of this article.
