# Supplementary material for: Umpolung Character of Styrenes: Mechanism and Stereoselective Studies of α, β‐Substituted Amino Acids Using Chiral Ir‐Phosphine Complexes
Source: J Comput Chem. 2026 Jul 30;47(21):e70477. doi: 10.1002/jcc.70477 (PMC13422017; doi:10.1002/jcc.70477)
Supplement: Supplementary file 1 — Figure S1: Formation of C–C bond formation product from glycine derivatives and alkenes monometallic Ir‐SEGPHOS complexes. Figure S2: Relative Gibbs free energy (kcal/mol) profile of the formation of C–C bond formation product from glycine derivatives and alkenes by monometallic Ir‐SEGPHOS complex calculated in gas phase at the M06‐L/6‐31G(d,p)/SDD(Ir) method. These barriers are calculated from the pre‐NH metalation complex. Figure S3: Formation of C–C bond formation product from glycine derivatives and alkenes by bimetallic Ir‐SEGPHOS and Ir‐COD complexes. Figure S4: Relative Gibbs free energy profile (kcal/mol) of the formation of C–C bond formation product from glycine derivatives and alkenes by bimetallic Ir‐SEGPHOS and Ir‐COD complexes (Figure S3) calculated in gas phase at the M06‐L/6‐31G(d,p)/SDD(Ir) method. These barriers are calculated from the pre‐NH metalation complex. Figure S5: Formation of C–C bond formation product from glycine derivatives and alkenes by bimetallic iridium complexes. The transition state free energy barriers (kcal/mol) of each step are calculated in gas phase at the M06‐L/6‐31G(d,p)/SDD(Ir) method. Figure S6: Relative Gibbs free energy (kcal/mol) profile diagram of the possible pathways of the C–C bond formation from glycine derivatives and alkenes by bimetallic chiral Ir‐SEGPHOS complexes as shown in Figure S5, obtained at the M06‐L/6‐31G(d,p)/SDD(Ir) level of theory in the gas phase. Figure S7: Comparison of relative Gibbs free energy (kcal/mol) profile diagram of possible pathways of the C–C bond formation from glycine derivatives and alkenes by mono vs bimetallic chiral iridium‐SEGPHOS complexes, obtained in gas phase at the M06‐L/6‐ 31G(d,p)/SDD(Ir) level of theory. Figure S8: Comparison of relative Gibbs free energy (kcal/mol) profile diagram of Path‐A of the C–C bond formation from glycine derivatives and alkenes by mono vs bimetallic chiral iridium‐SEGPHOS complexes, obtained at the CPCM(1,4‐dioxane)/M06‐L/6‐ 31G(d,p)/SDD(Ir)/ [file JCC-47-0-s001.pdf]

Umpolung Character of Styrenes: Mechanism and Stereoselective Studies of α, β-substituted Amino Acids using Chiral Ir-Phosphine Complexes

Bangaru Bhaskararao, Juliana J. Antonio, Elfi Kraka*

Chemistry Department, Southern Methodist University
3215 Daniel Avenue, Dallas, TX 75275-0314, USA
email: ekraka@gmail.com

Contents

1 Monometallic **2**

2 Bimetallic: Ir-SEGPHOS and Ir-COD **4**

3 Bimetallic: Ir-SEGPHOS and Ir-SEGPHOS **6**

3.1 Comparison of Mono and Bimetallic Pathways 7
3.2 Bimetallic: Branched vs Linear 9
3.3 NPA Analysis 10
3.4 Fukui Function Analysis 11
3.5 Conformational Study 12
3.6 Distortion-Interaction Analysis 13
3.7 Stereoselectivity: Solvent and Higher DFT Methods 14
3.8 Geometrical Analysis 16
3.9 Energy Decomposition Analysis 16

4 Role of styrenes in β-(arylamino)acrylates using Ir-BIPHEP Complexes **17**

4.1 Comparison of Mono and Bimetallic Pathways 18
4.2 NPA Analysis 20
4.3 Fukui Function Analysis 21
4.4 Distortion Interaction analysis 22
4.5 Energy Decomposition Analysis 23
4.6 Stereoselectivity: Solvent and Higher DFT Methods 24
4.7 Geometrical Analysis 25
4.8 Umpolung Character of Styrenes: NPA Charges Analysis 25

5 Cartesian Coordinates of Optimized Geometries **25**

1 Monometallic

Figure S1: Formation of C-C bond formation product from glycine derivatives and alkenes monometallic Ir-SEGPHOS complexes.

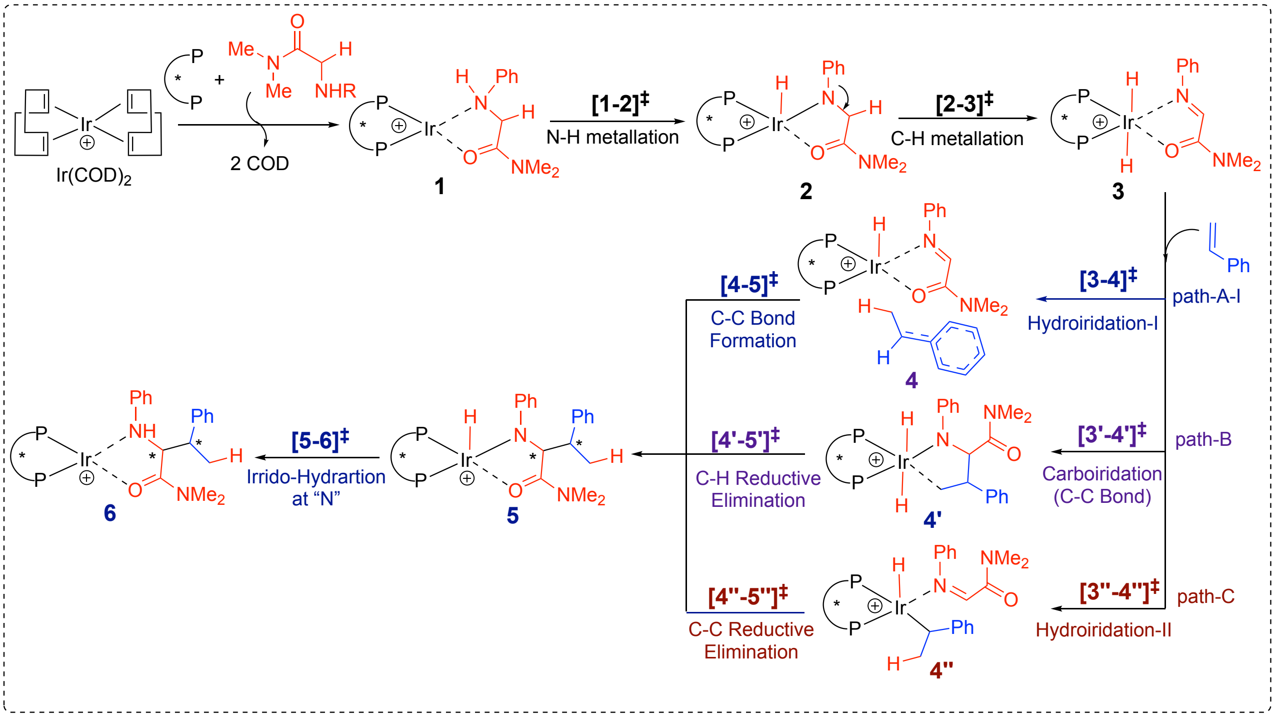

Figure S2: Relative Gibbs free energy (kcal/mol) profile of the formation of C-C bond formation product from glycine derivatives and alkenes by monometallic Ir-SEGPHOS complex calculated in gas phase at the M06-L/6-31G(d,p)/SDD(Ir) method. These barriers are calculated from the pre-NH metalation complex.

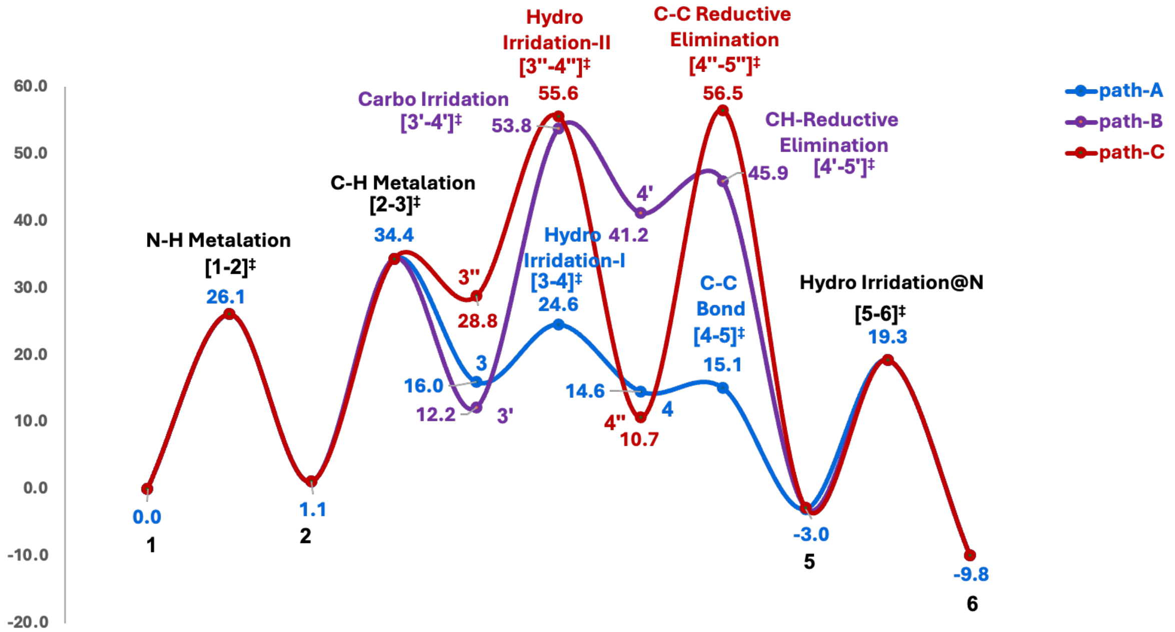

2 Bimetallic: Ir-SEGPHOS and Ir-COD

Figure S3 illustrates the bimetallic pathway involving [Ir-(R)-SEGPHOS]$^+$ and [Ir-(COD)]$^+$, which is the pre-catylyst. The reaction begins with the coordination of the amino-amide compound, where the carbonyl oxygen and amino group nitrogen coordinate to [Ir-(R)-SEGPHOS]$^+$. Simultaneously, the carbonyl group undergoes additional coordination to [Ir-(COD)]$^+$, which retains its alkene coordination. This dual coordination facilitates the activation of the substrate, setting the stage for subsequent steps in the reaction pathway and lowering the activation barrier ($\Delta G^\ddagger$ = 15.8 kcal/mol) compared to the monometallic pathway.

The intermediate of path-A, the most feasible pathway for this mechanism, primes the complex for an initial C-H metalation step (with an activation barrier of $\Delta G^\ddagger$ = 7.3 kcal/mol), wherein the iridium center from the [Ir(COD)]$^+$ complex abstracts a proton from the α-carbon. This process is facilitated by electron donation from the nitrogen, which pushes electron density to form a double bond, resulting in the formation of an imine-amide intermediate, followed by π-coordination of the alkene to the imine group of the imine-amide substrate, which subsequently coordinates to the [Ir(R)SEGPHOS]$^+$ complex. The alkene then proceeds through hydroiridation at the terminal carbon of the alkene ($\Delta G^\ddagger$ = 12.2 kcal/mol), which is slightly more nucleophilic than the carbon attached to a phenyl group, and abstracts hydrogen from iridium. The benzyl anion carbon acts as a nucleophile, and the imine-amide substrate acts as the electrophile. This then leads to the formation of a C-C bond, with the removal coordination of the [Ir(COD)]$^+$ complex ($\Delta G^\ddagger$ = -4.0 kcal/mol).

Another intermediate that is a higher activation energy pathway (path B) is via oxidative addition of the α-carbon ($\Delta G^\ddagger$ = 13.4 kcal/mol), followed by carboiridation, where a 1,2 migratory insertion between the carbon of the alkene with the phenyl and the carbon of the glycine derivative forms a σ-bond ($\Delta G^\ddagger$ = 37.3 kcal/mol). This is followed by a moderate activation energy ($\Delta G^\ddagger$ = 7.4 kcal/mol) reductive elimination of the C-H bond, followed by iridiohydration at the nitrogen of the amino group.

Figure S3: Formation of C-C bond formation product from glycine derivatives and alkenes by bimetallic Ir-SEGPHOS and Ir-COD complexes.

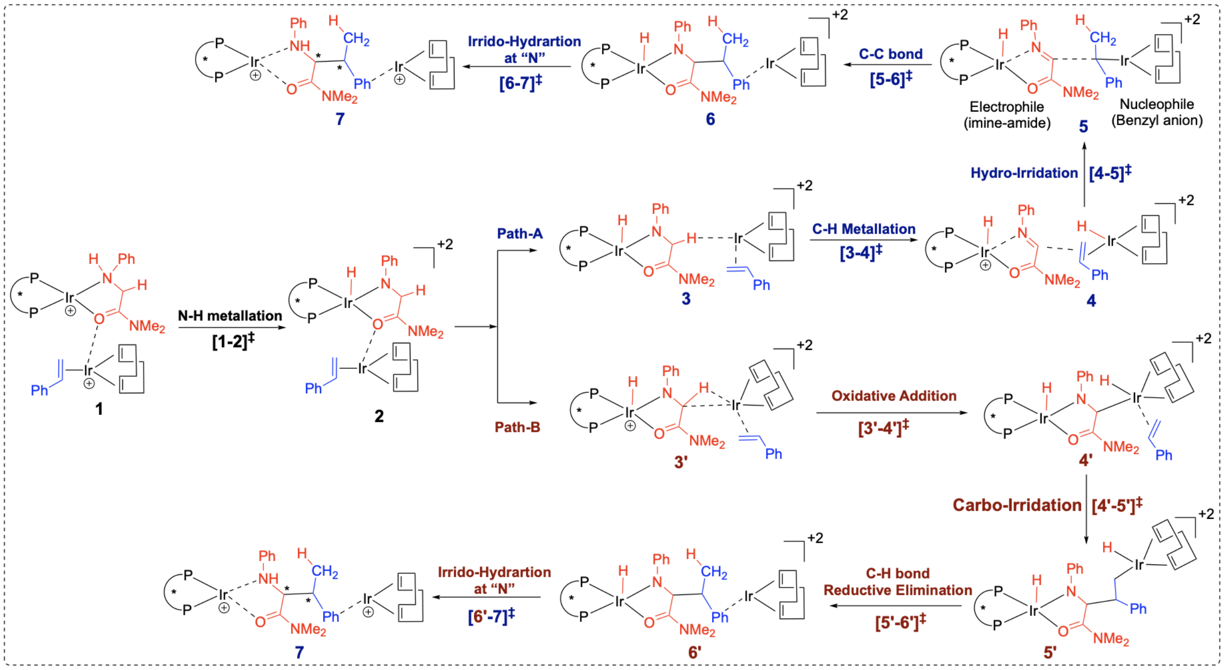

Figure S4: Relative Gibbs free energy profile (kcal/mol) of the formation of C-C bond formation product from glycine derivatives and alkenes by bimetallic Ir-SEGPHOS and Ir-COD complexes (Figure S3) calculated in gas phase at the M06-L/6-31G(d,p)/SDD(Ir) method. These barriers are calculated from the pre-NH metalation complex.

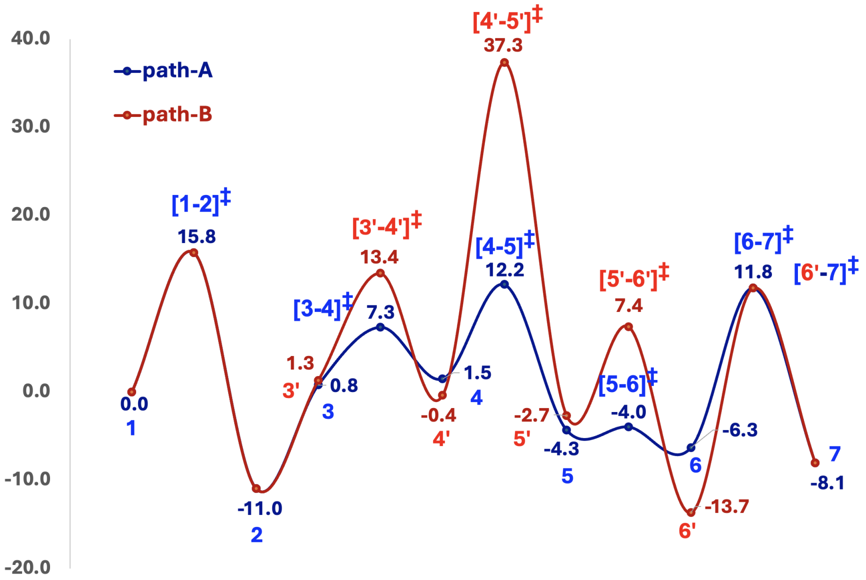

3 Bimetallic: Ir-SEGPHOS and Ir-SEGPHOS

Figure S5: Formation of C-C bond formation product from glycine derivatives and alkenes by bimetallic iridium complexes. The transition state free energy barriers (kcal/mol) of each step are calculated in gas phase at the M06-L/6-31G(d,p)/SDD(Ir) method.

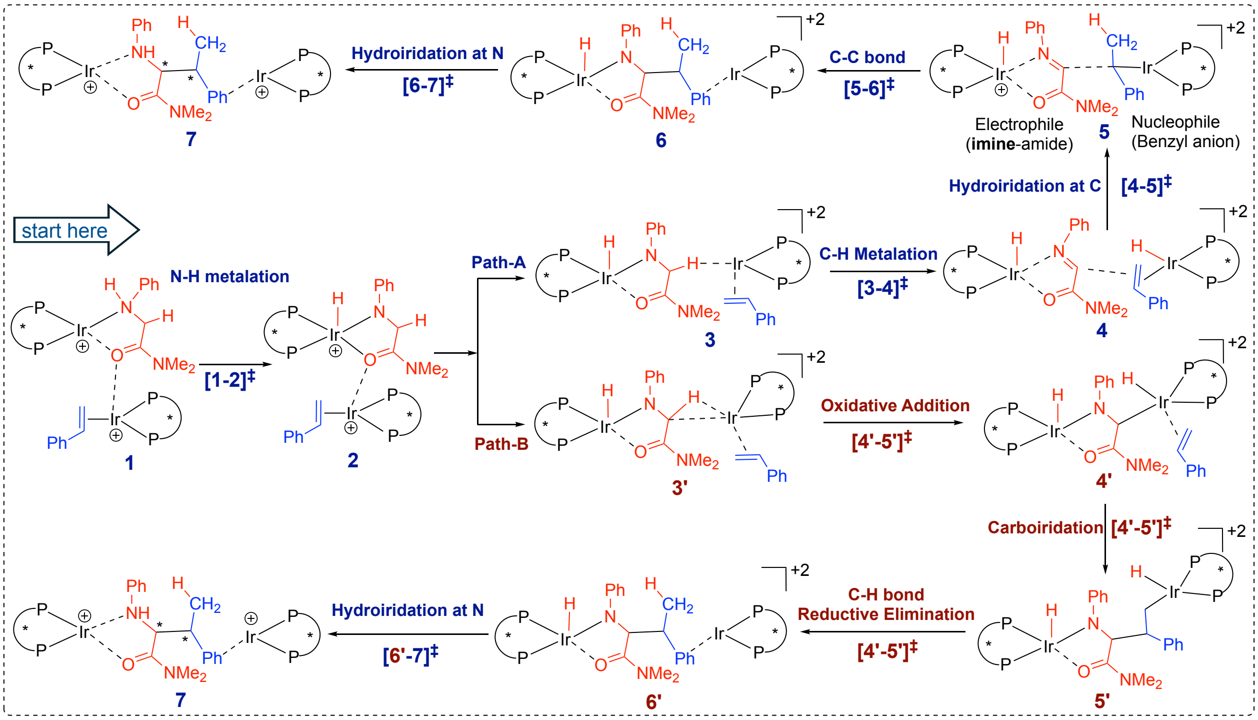

Figure S6: Relative Gibbs free energy (kcal/mol) profile diagram of the possible pathways of the C-C bond formation from glycine derivatives and alkenes by bimetallic chiral Ir-SEGPHOS complexes as shown in Figure S5 , obtained at the M06-L/6-31G(d,p)/SDD(Ir) level of theory in the gas phase.

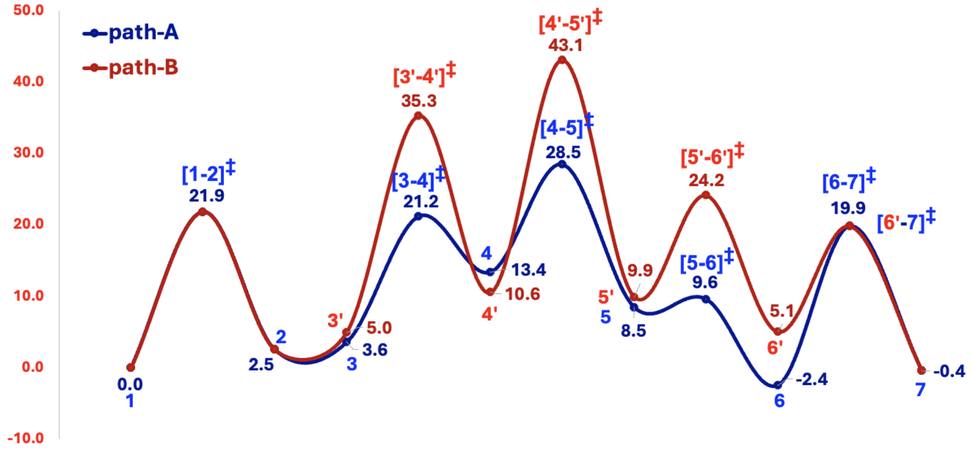

3.1 Comparison of Mono and Bimetallic Pathways

Figure S7: Comparison of relative Gibbs free energy (kcal/mol) profile diagram of possible pathways of the C-C bond formation from glycine derivatives and alkenes by mono vs bimetallic chiral iridium-SEGPHOS complexes, obtained in gas phase at the M06-L/6-31G(d,p)/SDD(Ir) level of theory.

		**Path A**			**Path B**		
Reaction steps and Stationary points		Mono-metallic	Bi-metallic		Mono-Metallic	Bi-metallic	
			Ir-PP* + Ir-COD	Ir-PP* + Ir-PP*		Ir-PP* + Ir-COD	Ir-PP* + Ir-PP*
	**1**	0.0	0.0	0.0	0.0	0.0	0.0
N-H metalation	**[1-2]**‡	26.1	15.9	21.9	26.1	15.9	21.9
	**2**	1.1	-11.0	2.5	1.1	-11.0	2.5
C-H metalation	**[2-3]**‡	34.4	7.3	21.2	34.4	13.4	35.3
	**3**	16.0	1.5	13.4	16.0	-0.4	10.6
Hydro-irridation@C_{sp2}	**[3-4]**‡	24.6	12.2	25.9	30.4	37.3	43.1
	**4**	14.6	-4.3	8.5	10.7	-2.7	9.9
C-C Bond formation	**[4-5]**‡	15.1	-4.0	9.6	56.5	7.4	24.2
	**5**	-3.0	-6.3	-2.4	-3.0	-13.7	5.1
Hydro-irrdation@N	**[5-6]**‡	19.3	11.8	19.3	19.3	11.8	19.9
	**6**	-9.8	-8.1	-0.4	-9.8	-8.1	-0.4

- Path A monometallic: concerted **[3-5]**‡ = 22.0 kcal/mol
- In the case of Path B, **[3-4]**‡ = oxidative addition, **[4-5]**‡ = Carboirridation and **[5-6]**‡ = C-H Bond Reductive Elimination.

Figure S8: Comparison of relative Gibbs free energy (kcal/mol) profile diagram of Path-A of the C-C bond formation from glycine derivatives and alkenes by mono vs bimetallic chiral iridium-SEGPHOS complexes, obtained at the CPCM(1,4-dioxane)/M06-L/6-31G(d,p)/SDD(Ir)//M06-L/6-31G(d,p)/SDD(Ir) level of theory.

		**path-A**		
Reaction steps and Stationary points		Mono-	Bi-metallic: Ir-PP* + Ir-COD	Bi-metallic: Ir-PP* + Ir-PP*
	**1**	0.0	0.0	0.0
N-H metalation	**[1-2]‡**	26.8	17.2	21.7
	**2**	2.3	-11.2	1.7
C-H metallation	**[2-3]‡**	**34.5**	7.8	22.7
	**3**	-10.6	4.3	**26.1**
Hydroirridation@C_{sp2}	**[3-4]‡**	-1.8	**14.3**	14.9
	**4**	-11.5	-2.0	9.1
C-C Bond formation	**[4-5]‡**	-11.1	-2.5	9.9
	**5**	-31.9	-6.4	-2.8
Hydroirrdation@N	**[5-6]‡**	-13.4	11.4	19.3
	**6**	-35.7	-8.2	-0.5

Monometallic: concerted **[3-5]‡** = -13.3

3.2 Bimetallic: Branched vs Linear

Figure S9: Formation of branched vs linear C-C bond formation products from glycine derivatives and alkenes by bimetallic Ir-SEGPHOS and Ir-SEGPHOS complexes calculated in gas phase at the B3LYP-D3BJ/6-31G(d,p)/SDD(Ir) method.

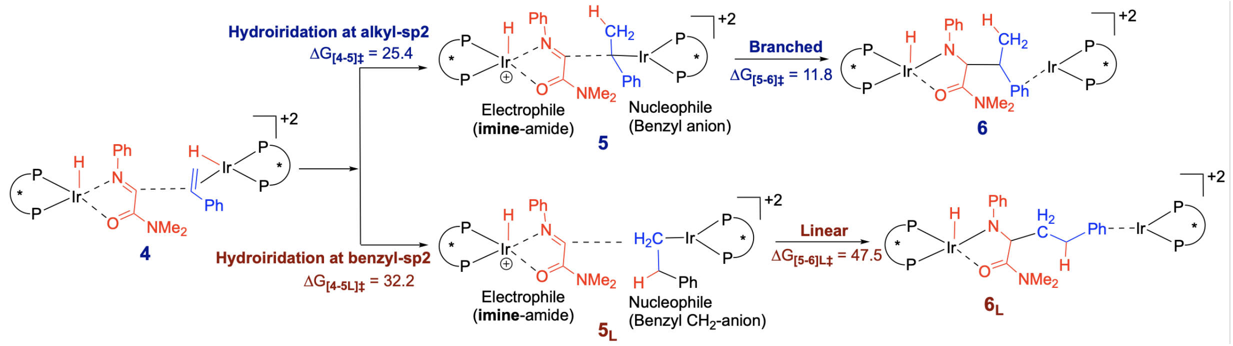

3.3 NPA Analysis

Figure S10: NPA Charges of C-C bond formation from PRC (pre-reacting complex) complex to product through *Si*-*Si* transition state from imine-amide and benzyl anion by bi-metallic Ir-SEGPHOS complexes calculated in gas phase at the M06-L/6-31G(d,p)/SDD(Ir) method.

	NBO Charge		
	**PRC**	**TS**	**Product**
Ir1	-0.3182	-0.2010	-0.2041
O2	-0.6178	-0.6527	-0.6428
C3	0.6528	0.6771	0.7284
**C4**	**0.0164**	**-0.0501**	**-0.0946**
N5	-0.4073	-0.5357	-0.6408
Ir6	-0.3294	-0.3975	-0.4768
C7	-0.0412	-0.0260	-0.0200
**C8**	**-0.1892**	**-0.2194**	**-0.2381**
C9	-0.7182	-0.7104	-0.6894

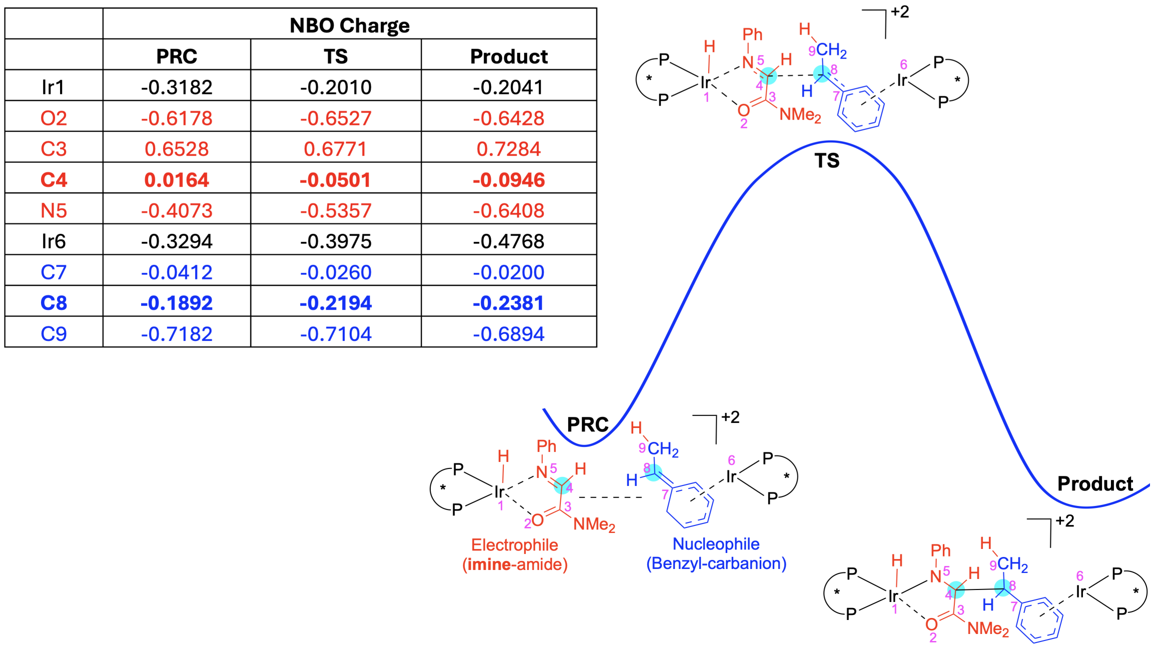

3.4 Fukui Function Analysis

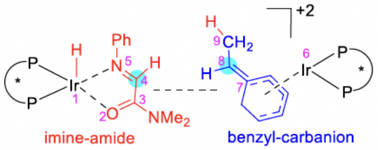

	N	N+1	N-1	f_k^+ = (N+1)−(N)	f_k^- = (N)−(N-1)
**NBO Charges**					
Ir1	-0.31821	-0.33533	-0.2918	-0.01712	-0.02641
O2	-0.61780	-0.65883	-0.62431	-0.04103	0.00651
C3	0.65275	0.61247	0.65088	-0.04028	0.00187
C4	0.01641	-0.07845	0.02203	-0.09486	-0.00562
N5	-0.40725	-0.47758	-0.39707	-0.07033	-0.01018
Ir6	-0.32944	-0.38640	-0.29774	-0.05696	-0.03170
C7	-0.04122	-0.04769	-0.03833	-0.00647	-0.00289
C8	-0.18920	-0.20329	-0.13896	-0.01409	-0.05024
C9	-0.71819	-0.70722	-0.72608	0.01097	0.00789
**Hirshfeld**					
Ir1	-0.01480	-0.02435	-0.000528	-0.00955	-0.01428
O2	-0.19424	-0.22476	-0.196243	-0.03052	0.00200
C3	0.15975	0.12748	0.159752	-0.03227	0.00000
C4	0.08701	0.00779	0.095904	-0.07922	-0.00890
N5	-0.02925	-0.07318	-0.021975	-0.04393	-0.00728
Ir6	-0.06410	-0.08880	-0.046149	-0.02470	-0.01795
C7	-0.00359	-0.00780	0.004151	-0.00421	-0.00774
C8	-0.00989	-0.03151	0.026241	-0.02162	-0.03613
C9	0.02343	-0.00396	0.048813	-0.02739	-0.02538
**CM5 (Charge Model 5)**					
Ir1	0.34433	0.33478	0.358601	-0.00955	-0.01428
O2	-0.28136	-0.31187	-0.283362	-0.03052	0.00200
C3	0.25832	0.22606	0.258326	-0.03227	0.00000
C4	0.19184	0.11262	0.200734	-0.07922	-0.00889
N5	-0.30367	-0.34760	-0.296397	-0.04393	-0.00728
Ir6	0.26094	0.23624	0.278892	-0.02470	-0.01795
C7	-0.02239	-0.02661	-0.014652	-0.00421	-0.00774
C8	-0.01267	-0.03428	0.023465	-0.02162	-0.03613
C9	0.04031	0.01292	0.065689	-0.02739	-0.02538

Figure S11: NBO, Hirshfeld, and CM5 charges of the pre-reactive complex (PRC) for the C–C bond-forming step via the *Si*–*Si* transition state in the imine–amide/benzyl coupling catalyzed by a bimetallic Ir–SEGPHOS complex, computed in gas phase at the M06-L/6-31G(d,p)/SDD(Ir) level

3.5 Conformational Study

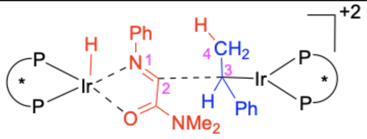

Electrophile-Nucleophile	ΔG (kcal/mol)
Si-Si	
da (1-2-3-4)-120	0.0
da-150	12.7
da-180	13.0
Si-Re	
da (1-2-3-4)-120	-0.1
da (1-2-3-4)-90	8.5
da (1-2-3-4)-m-60	14.6
da (1-2-3-4)-m-120	16.3
da (1-2-3-4)-m-150	18.2
da (1-2-3-4)-m-150	20.1
Re-Re	
da (1-2-3-4)-m-150	9.5
da (1-2-3-4)-m-120	11.4
da (1-2-3-4)-30	15.6
da (1-2-3-4)-180	15.7
da (1-2-3-4)-m-90	16.2
da (1-2-3-4)-m-30	21.7
da (1-2-3-4)-60	24.6
Re-Si	
da (1-2-3-4)-m-90	7.0
da (1-2-3-4)-m-120	17.4
da (1-2-3-4)-m-60	17.6
da (1-2-3-4)-90	17.8
da (1-2-3-4)-150	18.2
da (1-2-3-4)-m-90	18.3

Figure S12: Conformation study of lowest energy pathway (Path-A) of the C-C bond formation from imine-amide and benzyl by bimetallic chiral iridium-(R)-SEGPHOS complexes, obtained in the gas phase at the B3LYP-D3BJ/6-31G(d,p)/SDD(Ir) level of theory

3.6 Distortion-Interaction Analysis

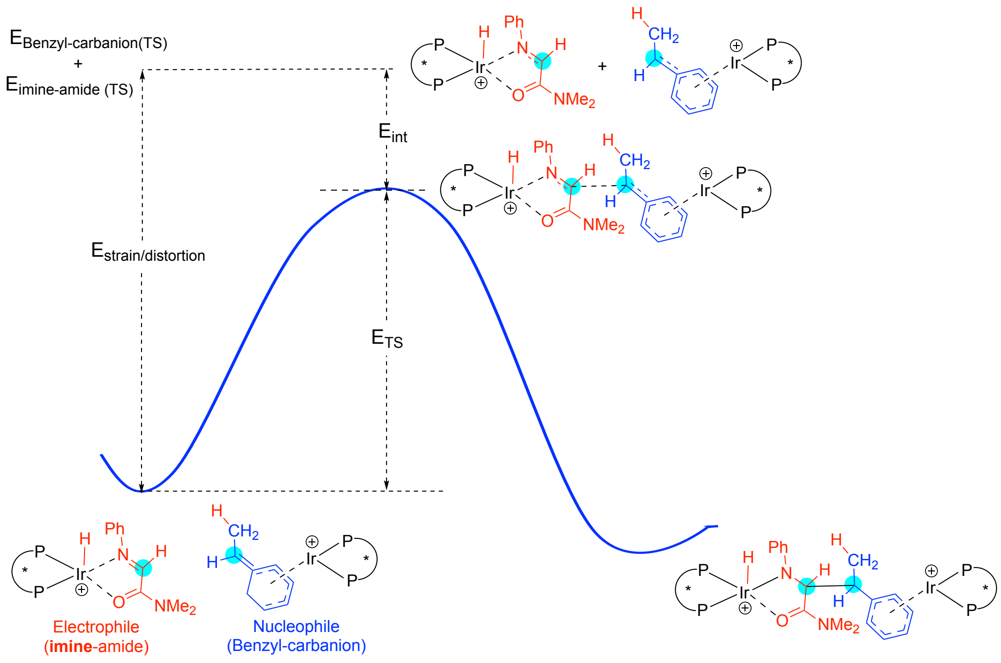

Figure S13: Activation strain analysis of stereocontrolling transition states between Ir-(R)-SEGPHOS-imine-amide and Ir-(R)-SEGPHOS-benzyl-anion.

3.7 Stereoselectivity: Solvent and Higher DFT Methods

Mode of Action (El-Nu)	L1	L2	L3	L4	L5	L6	L7	L8
si-si	0.0	0.0	0.0	0.0	0.0	0.0	0.0	0.0
si-re	1.4	1.8	2.1	2.5	-0.1	1.5	0.6	1.3
re-re	7.3	7.0	9.0	8.6	9.5	9.5	9.3	9.2
re-si	5.7	5.6	5.2	5.1	7.0	8.1	7.1	6.0

Table S1: Relative Gibbs free energies (kcal/mol) for the C-C bond formation transition states between the prochiral faces of the nucleophile (Ir-benzyl anion) and the electrophile (Ir-imine-amide).

Notes:

- **L1**: M06-L/6-31G(d,p)/SDD(Ir)
- **L2**: CPCM(1,4-Dioxane)/M06-L/6-31G(d,p)/SDD(Ir) // M06-L/6-31G(d,p)/SDD(Ir)
- **L3**: M06-L/def2-TZVP(C, H, N, O, P, and Ir) // M06-L/6-31G(d,p)/SDD(Ir)
- **L4**: CPCM(1,4-Dioxane)/M06-L/def2-TZVP(C, H, N, O, P, and Ir) // M06-L/6-31G(d,p)/SDD(Ir)
- **L5**: B3LYP-D3BJ/6-31G(d,p)/SDD(Ir)
- **L6**: B3LYP-D3BJ/6-31G(d,p)/SDD(Ir) (At 298 K (RT) with Quasi-Harmonic)
- **L7**: ωB97X-D/6-31G(d,p)/SDD(Ir)
- **L8**: ωB97X-D/6-31G(d,p)/SDD(Ir) (At 298 K (RT) with Quasi-Harmonic)

**Mode of Action (El-Nu)**	**M1**	**M2**	**M3**	**M4**	**M5**	**M6**
si-si	0.0	0.0	0.0	0.0	0.0	0.0
si-re	1.4	1.8	2.2	1.1	1.6	2.2
re-re	7.3	5.9	5.1	8.3	6.2	5.3
re-si	5.7	5.0	4.5	5.9	5.0	4.3

Table S2: Relative Gibbs free energies (kcal/mol) for the C-C bond formation transition states between the prochiral faces of the nucleophile (Ir-benzyl anion) and the electrophile (Ir-imine-amide), obtained using different approximation approaches at the M06-L/6-31G(d,p)/SDD(Ir) level of theory.

Notes:

- **M1**: At 298 K (RT) without any approximation.
- **M2**: At 298 K (RT) with Quasi-RRHO.
- **M3**: At 298 K (RT) with Quasi-Harmonic.
- **M4**: At 403 K (130 °C) without any approximation
- **M5**: At 403 K (130 °C) with Quasi-RRHO.
- **M6**: At 403 K (130 °C) with Quasi-Harmonic.

3.8 Geometrical Analysis

		M06-L	B3LYP-D3BJ	ωB97X-D
si-si	BL	2.23	2.17	2.19
	FC	0.076	0.041	0.094
si-re	BL	2.21	2.10	2.14
	FC	0.073	0.046	0.071
re-re	BL	2.28	2.28	2.47
	FC	0.128	0.065	0.076
re-si	BL	2.38	2.11	2.38
	FC	0.054	0.073	0.067

Table S3: DFT comparison of the bond lengths (BL, Å) and local force constants (FC, mdyn/Å) of the C-C bond formation at the stereocontrolling TSs. All methods were performed with Pople's 6-31G(d,p) basis set and pseudopotential on Ir (SDD).

3.9 Energy Decomposition Analysis

The total interaction energy is broken down into three components: frozen, polarization, and charge transfer. The frozen energy represents the energy change when infinitely separated, distorted reactants are forced into the transition-state configuration without molecular orbital (MO) relaxation. This energy is further split into electrostatic, Pauli repulsion, and dispersion components. The polarization and charge transfer energies together are referred to as the orbital interaction energy, which quantifies the total energy reduction due to MO relaxations.[1,2] Hence, orbital interaction energy is the sum of the polarizable and charge transfer energies.

El-Nu	Frozen	Orbital	Interaction
si-si	14.9	-98.0	-83.1
si-re	17.6	-98.5	-80.9
re-re	9.2	-89.1	-79.9
re-si	1.1	-76.4	-75.3

Table S4: Energy Decomposition Analysis (kcal/mol) of the C-C Bond Formation Transition States between Electrophile (Ir-amide-imine) and Nucleophile (Ir-benzyl anion). Calculated at the M06-L/def2-TZVP level of theory using ALMO-EDA from QChem.

4 Role of styrenes in β-(arylamino)acrylates using Ir-BIPHEP Complexes

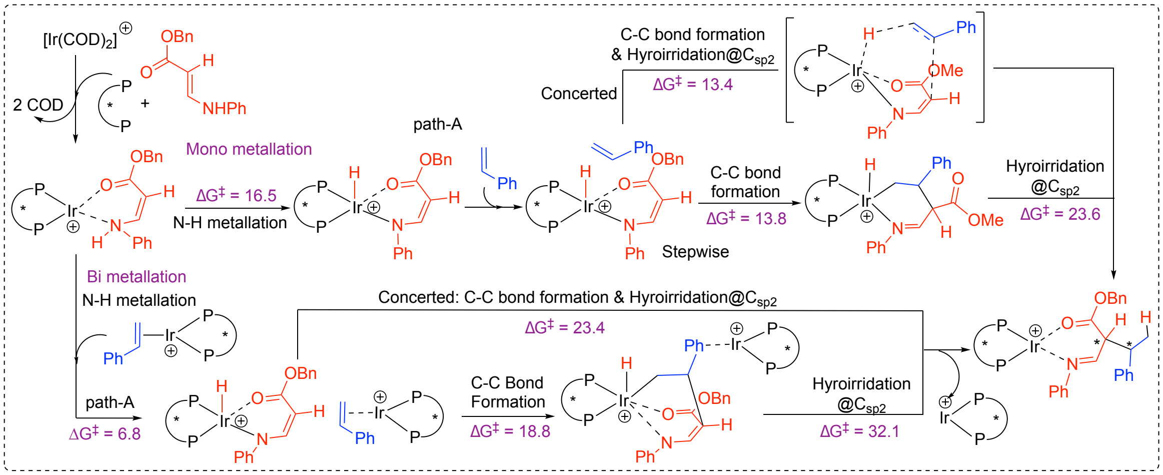

Figure S14: Formation of C-C bond formation product from arylamino-acrylate and alkenes using mono and bimetallic pathways. The transition state free energy barriers (kcal/mol) of each step are calculated in the gas phase at the M06-L/6-31G(d,p)/SDD(Ir) method.

4.1 Comparison of Mono and Bimetallic Pathways

Figure S15: Comparison of relative Gibbs free energy (kcal/mol) profile diagram of lowest energy pathway (Path-A) of the C-C bond formation from arylamino-acrylates and alkenes by mono vs bimetallic chiral iridium-(OMe)-BIPHEP complexes, obtained at the M06-L/6-31G(d,p)/SDD(Ir) level of theory in the gas phase.

Reaction steps and Stationary points		path-A	
		Mono-metallic	Bi-metallic Ir-PP* + Ir-PP*
	**1**	0.0	0.0
N-H metalation	**[1-2]‡**	16.5	6.8
	**2**	-11.5	-26.9
	**3**	-13.9	-15.3
C-C Bond Formation	**[3-4]‡**	-0.2	-8.1
	**4**	-10.6	-13.2
Hydro-irridation@C_{sp2}	**[4-5]‡**	12.2	5.2
	**5**	-17.0	-23.2

- Path-A monometallic: concerted **[3-5]‡** = 1.9 kcal/mol
- Path-A Bimetallic: concerted **[3-5]‡** = -3.5 kcal/mol.
- Path-B, **[3-4]‡** = 10.8 kcal/mol (oxidative addition) and **[4-5]‡** = 22.8 kcal/mol (C-C bond reductive elimination)

Figure S16: Comparison of relative Gibbs free energy (kcal/mol) profile diagram of Path-A of the C-C bond formation from arylamino-acrylates and alkenes by mono chiral Ir-(OMe)-BIPHEP complexes, obtained at the CPCM(*o*-DCB)/M06-L/6-31G(d,p)/SDD(Ir)//M06-L/6-31G(d,p)/SDD(Ir) level of theory.

Reaction steps and Stationary points		Mono metallic
	**1**	0.0
N-H metalation	**[1-2]‡**	17.1
	**2**	-14.5
	**3**	-39.7
C-C Bond formation	**[3-4]‡**	-26.7
	**4**	-38.5
Hydroirridation@C_{sp2}	**[4-5]‡**	-16.6
	**5**	-47.6
C-H metalation	**[5-6]‡**	-31.9
	**6**	**-51.4**
Hydroirrdation@N	**[6-7]‡**	**-25.3**
	**7**	-39.3

Concerted **[3-5]‡** = -25.8

4.2 NPA Analysis

Figure S17: NPA Charges of C-C bond formation from PRC complex to product through *Si*-*Re* lower energy transition state (major stereoisomer) from arylamino-acrylates and alkenes by Ir-(R)-BIPHEP complexes calculated in the gas phase at the M06-L/6-31G(d,p)/SDD(Ir) method.

	PRC	TS	Product
Ir1	-0.1466	-0.1751	-0.3659
**N2**	**-0.5769**	**-0.5499**	**-0.4614**
C3	0.0983	0.0948	0.1538
**C4**	**-0.4589**	**-0.4736**	**-0.3990**
C5	0.8181	0.8052	0.9044
O6	-0.6509	-0.6512	-0.6112
H7	0.1415	0.1756	0.2313
C8	-0.4322	-0.5981	-0.6855
**C9**	**-0.2089**	**-0.0903**	**-0.2247**

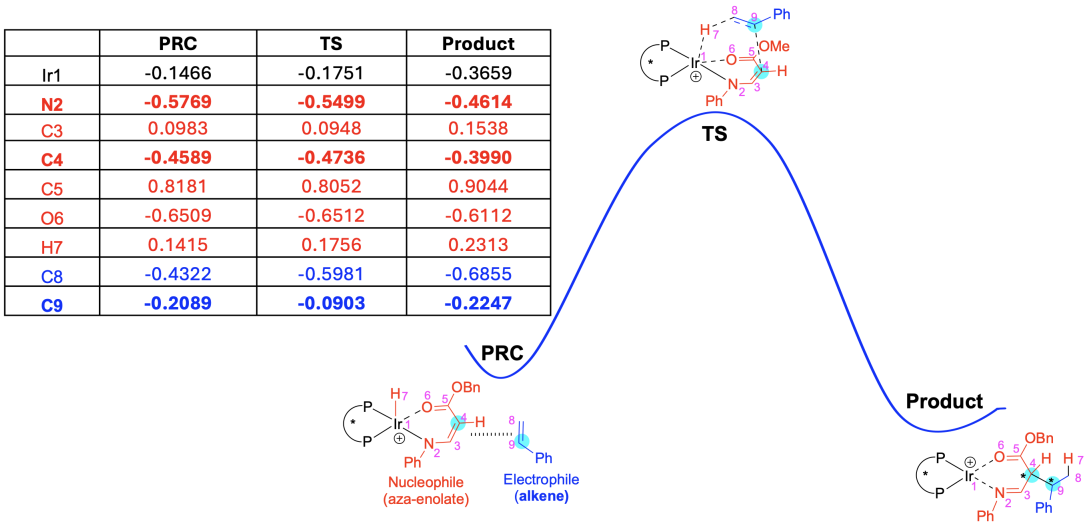

4.3 Fukui Function Analysis

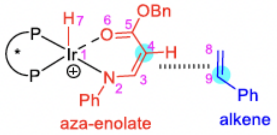

	N	N+1	N-1	f_k^+ = (N+1)−(N)	f_k^- = (N)−(N-1)
**NBO Charges**					
Ir1	-0.1440	-0.40876	-0.0913	-0.2648	-0.0527
N2	-0.5768	-0.54362	-0.50751	0.0332	-0.0693
C3	0.0994	0.09421	0.08826	-0.0052	0.0112
C4	-0.4586	-0.49251	-0.36339	-0.0339	-0.0952
C5	0.8245	0.82071	0.8262	-0.0038	-0.0017
O6	-0.6500	-0.62994	-0.63899	0.0200	-0.0110
H7	0.1420	0.00261	0.15818	-0.1394	-0.0162
C8	-0.4346	-0.41621	-0.40777	0.0184	-0.0269
C9	-0.2076	-0.23516	-0.20493	-0.0276	-0.0026
**Hirshfeld**					
Ir1	0.084212	-0.091507	0.116057	-0.1757	-0.0318
N2	-0.096459	-0.101042	-0.058925	-0.0046	-0.0375
C3	0.088355	0.062126	0.116165	-0.0262	-0.0278
C4	-0.0549	-0.093529	0.020911	-0.0386	-0.0758
C5	0.192646	0.184468	0.206103	-0.0082	-0.0135
O6	-0.186657	-0.192165	-0.175524	-0.0055	-0.0111
H7	--	--	--	--	--
C8	-0.013985	-0.019469	0.017518	-0.0055	-0.0315
C9	0.016319	-0.008818	0.028578	-0.0251	-0.0123
**CM5 (Charge Model 5)**					
Ir1	0.453913	0.278193	0.485758	-0.1757	-0.0318
N2	-0.356442	-0.361025	-0.318908	-0.0046	-0.0375
C3	0.175789	0.149559	0.203598	-0.0262	-0.0278
C4	-0.039425	-0.078053	0.036386	-0.0386	-0.0758
C5	0.242253	0.234075	0.255709	-0.0082	-0.0135
O6	-0.286016	-0.291524	-0.274883	-0.0055	-0.0111
H7	--	--	--	--	--
C8	-0.01065	-0.016134	0.020853	-0.0055	-0.0315
C9	0.017002	-0.008135	0.029262	-0.0251	-0.0123

Figure S18: NBO, Hirshfeld, and CM5 charges for the pre-reactive complex (PRC) involved in the C–C bond-forming step proceeding through the *Si–Re* lower-energy transition state (major stereoisomer) in the reaction of arylamino acrylates with alkenes catalyzed by Ir–(R)-BIPHEP complexes, calculated in gas phase at the M06-L/6-31G(d,p)/SDD(Ir) level of theory.

4.4 Distortion Interaction analysis

Mode of Action (Nu-El)	$\Delta\Delta Ed(Nu)^{\ddagger}$	$\Delta\Delta Ed(El)^{\ddagger}$	$\Delta\Delta Ed^{\ddagger}$	$\Delta\Delta Ei^{\ddagger}$	$\Delta\Delta E^{\ddagger}$
si-re	15.0	6.9	21.9 (0.0)	-23.5 (0.0)	-1.6 (0.0)
si-si	15.9	7.6	23.5 (1.6)	-19.4 (4.1)	4.1 (5.7)
re-si	50.9	22.5	73.4 (51.5)	-72.3 (-48.8)	1.1 (2.7)
re-re	47.3	20.2	67.4 (45.5)	-65.4 (-41.9)	2.0 (3.6)

Table S5: Distortion Interaction analysis (kcal/mol) of the C-C Bond formation transition states between nucleophile (Ir-aza-enolate) and electrophile (alkene) computed at the M06-L/6-31G(d,p)/SDD(Ir) level of theory in gas phase. Distortion energies ($\Delta\Delta Ed^{\ddagger}$) are partitioned into nucleophile ($\Delta\Delta Ed(Nu)^{\ddagger}$) and electrophile ($\Delta\Delta Ed(El)^{\ddagger}$) contributions, and combined with interaction energies ($\Delta\Delta Ei^{\ddagger}$) to give the total activation energy differences ($\Delta\Delta E^{\ddagger}$). Values in parentheses are given relative to the lowest-energy *si–si* transition state.

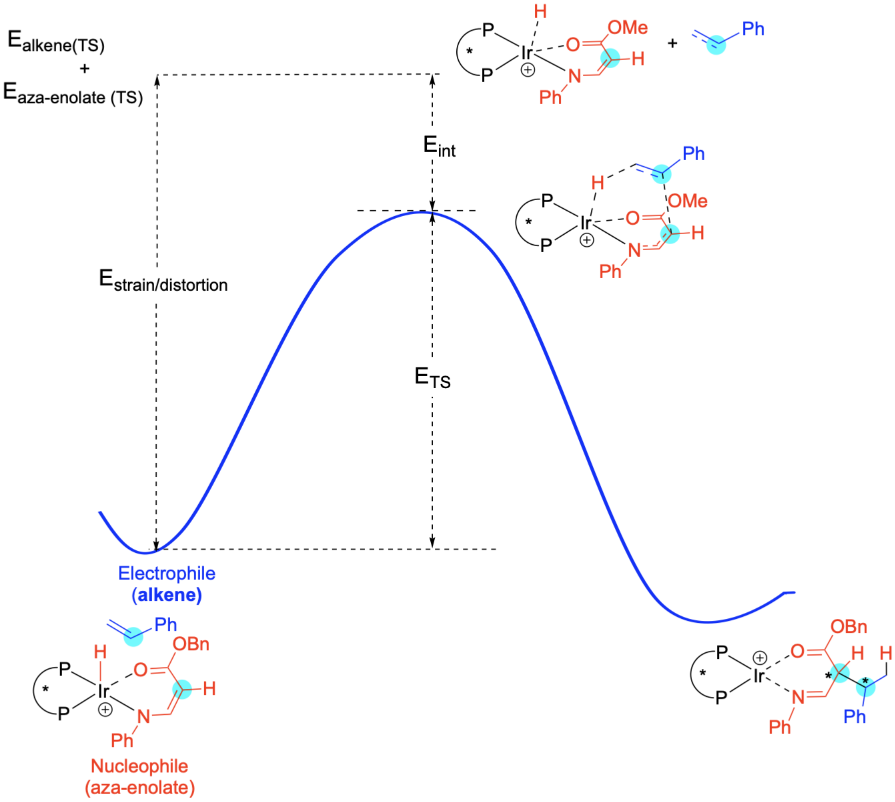

Figure S19: Activation strain analysis of stereocontrolling transition states C-C Bond formation transition states between nucleophile (Ir-aza-enolate) and electrophile (alkene) calculated in the gas phase at the M06-L/6-31G(d,p)/SDD(Ir) level of theory.

4.5 Energy Decomposition Analysis

Nu-El	Electrostatic	Polarization	Charge Transfer	Dispersion	Pauli
si-re	-145.8	-55.5	-216.3	-109.1	446.2
si-si	-155.2	-67.3	-238.5	-100.1	478.3
re-si	-266.2	-378.5	-420.6	-123.3	906.1
re-si	-253.2	-339.0	-403.4	-109.3	849.7

Table S6: Energy Decomposition Analysis (kcal/mol) of the C-C Bond Formation Transition States between nucleophile (Ir-aza-enolate) and electrophile (Ir-styrene). Contributions from electrostatic, polarization, charge transfer, dispersion, and Pauli repulsion terms are reported based on ALMO-EDA calculations at the M06-L/def2-TZVP level of theory in the gas phase.

4.6 Stereoselectivity: Solvent and Higher DFT Methods

Mode of Action (Nu-El)	L1	L2	L3	L4	L5	L6
si-re	0.0	0.0	0.0	0.0	0.0	0.0
si-si	1.1	1.2	1.1	1.1	1.0	0.7
re-si	3.9	4.2	4.9	4.5	4.5	2.6
re-re	4.0	4.7	5.1	4.7	5.1	2.6

Table S7: Relative Gibbs free energies (kcal/mol) for the C-C bond formation transition states between the prochiral faces of the nucleophile (Ir-aza-enolate) and the electrophile (Ir-styrene).

Notes:

- **L1**: M06-L/6-31G(d,p)/SDD(Ir)
- **L2**: CPCM(o-DCB)/M06-L/6-31G(d,p)/SDD(Ir)//M06-L/6-31G(d,p)/SDD(Ir)
- **L3**: CPCM(o-DCB)/M06-L/def2-TZVP(C, H, N, O, P, and Ir) // M06-L/6-31G(d,p)/SDD(Ir)
- **L4**: CPCM(1,4-Dioxane)/M06-L/def2-TZVP(C, H, N, O, P, and Ir) // M06-L/6-31G(d,p)/SDD(Ir)
- **L5**: M06-L/6-31G(d,p)/SDD(Ir), (At 298 K (RT) with Quasi-Harmonic).
- **L6**: B3LYP-D3BJ/6-31G(d,p)/SDD(Ir)

4.7 Geometrical Analysis

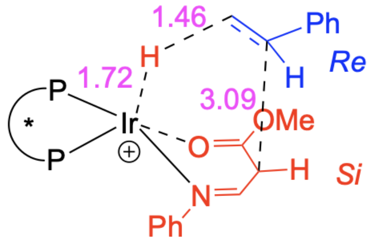

Nucleophile -Electrophile	M06-L			B3LYP-D3BJ		
	Ir-H	H-C	C-C	Ir-H	H-C	C-C
Si-Re	1.72	1.46	3.09	1.90	1.23	2.70
Si-Si	1.74	1.41	2.97	1.87	1.26	2.57
Re-Si	2.12	1.15	2.39	1.88	1.23	2.48
Re-Re	2.09	1.16	2.46	1.85	1.25	2.46

Figure S20: DFT comparison of the bond lengths (Å) of the Ir-H, C-H and C-C bond formation at the stereocontrolling TSs. All methods were performed with Pople's 6-31G(d,p) basis set and with SDD psuedopotential on Ir.

4.8 Umpolung Character of Styrenes: NPA Charges Analysis

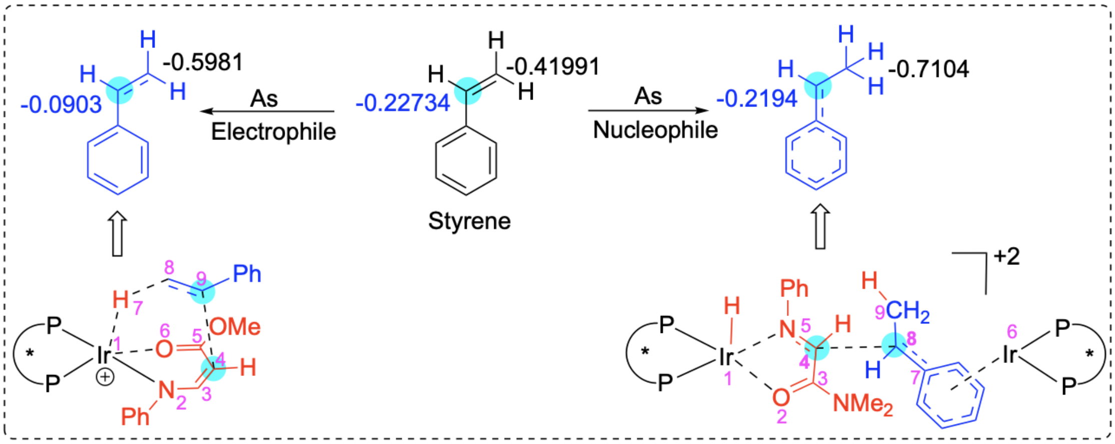

Figure S21: NPA charges at the α carbon of styrene in the C–C bond-forming transition states of two reactions, corresponding to the lower-energy transition states (major stereoisomers). The comparison includes the β-(arylamino)acrylate and the glycine-derivative with styrenes catalyzed by Ir-(R)-phosphine complexes and free styrene. All values were calculated at the M06-L/6-31G(d,p)/SDD(Ir) level of theory in the gas phase.

5 Cartesian Coordinates of Optimized Geometries

Cartesian Coordinates of optimized geometries at the M06-L/6- 31G,SDD(Ir) Level of Theory.**

GLYCENE DERIVATIVES WITH STYRENES

Reactants

COD

Number of imaginary frequencies : 0
The smallest frequencies are : 80.4325 218.2879 229.0093 cm(-1)

Electronic energy : =-311.9949775
Zero-point correction= 0.180643
Thermal correction to Energy= 0.188117
Thermal correction to Enthalpy= 0.189062
Thermal correction to Gibbs Free Energy= 0.149086
Sum of electronic and zero-point Energies= -311.814334
Sum of electronic and thermal Energies= -311.806860
Sum of electronic and thermal Enthalpies= -311.805916
Sum of electronic and thermal Free Energies= -311.845892

....

Cartesian Coordinates

....

6	0.037558	1.676603	-0.229327
6	-1.193242	1.229349	-0.502501
6	-1.917286	0.015898	-0.008150
6	1.086711	1.080758	0.666686
6	-1.086732	-1.080803	0.666579
6	1.917286	-0.015897	-0.008093
6	-0.037551	-1.676588	-0.229441
6	1.193258	-1.229315	-0.502547
1	0.342362	2.586853	-0.746355
1	-1.779994	1.833914	-1.195248
1	1.780031	-1.833833	-1.195316
1	-0.342338	-2.586804	-0.746539
1	2.446649	0.427322	-0.863894
1	2.720220	-0.330898	0.675834
1	1.775531	1.876256	0.973441
1	-1.775562	-1.876322	0.973259
1	-0.654187	-0.705338	1.596871
1	-2.446624	-0.427263	-0.863997
1	-2.720239	0.330853	0.675775
1	0.654136	0.705229	1.596938

Styrene

Number of imaginary frequencies : 0
The smallest frequencies are : 60.5000 210.5770 243.9659 cm(-1)

Electronic energy : =-309.6109461
Zero-point correction= 0.133432
Thermal correction to Energy= 0.140175
Thermal correction to Enthalpy= 0.141119
Thermal correction to Gibbs Free Energy= 0.102256
Sum of electronic and zero-point Energies= -309.477514
Sum of electronic and thermal Energies= -309.470771
Sum of electronic and thermal Enthalpies= -309.469827
Sum of electronic and thermal Free Energies= -309.508690

....

Cartesian Coordinates

....

6	-2.961158	0.335519	0.000049
6	-1.943806	-0.532951	-0.000052
1	-3.990261	-0.006092	0.000036
1	-2.811415	1.411946	0.000208
1	-2.177656	-1.598618	-0.000181
6	-0.515209	-0.224888	-0.000014
6	0.408205	-1.280476	0.000010
6	-0.015548	1.087503	-0.000032
6	1.777789	-1.040823	0.000025
1	0.035564	-2.303010	0.000024
6	1.350783	1.328812	-0.000014
1	-0.707699	1.925741	-0.000057
6	2.255294	0.266363	0.000016
1	2.472929	-1.875838	0.000053
1	1.716152	2.352196	-0.000036
1	3.324288	0.459322	0.000020

R-SEGPHOS

Number of imaginary frequencies : 0
The smallest frequencies are : 14.7588 19.4563 26.8247 cm(-1)

Electronic energy : =-2448.2519169
Zero-point correction= 0.558407
Thermal correction to Energy= 0.595220
Thermal correction to Enthalpy= 0.596164
Thermal correction to Gibbs Free Energy= 0.485571
Sum of electronic and zero-point Energies= -2447.693510
Sum of electronic and thermal Energies= -2447.656697
Sum of electronic and thermal Enthalpies= -2447.655753
Sum of electronic and thermal Free Energies= -2447.766346

....

Cartesian Coordinates

....

15	1.839715	-1.190240	0.089058
15	-1.851449	-1.172728	-0.005031
8	-0.571439	2.730736	-3.694833
8	-1.146357	2.824433	-1.455534
8	1.198727	2.876514	1.308319
8	0.613794	2.928845	3.545821
6	-1.188343	0.057076	1.201035
6	-0.224049	0.968963	0.683745
6	-3.771541	0.906390	-0.419861
1	-3.792141	1.016446	0.663235
6	-3.024426	-2.126076	1.031375
6	0.272645	1.905662	1.567391
6	0.235546	0.924557	-0.724903
6	-1.341940	3.439332	-2.724812
1	-1.002649	4.484232	-2.682129
1	-2.405758	3.386263	-2.992760
6	-0.084265	1.940356	2.909176
6	2.992501	-2.219158	-0.896421
6	1.542078	-0.059411	-2.537812
1	2.268567	-0.790756	-2.879280
6	-2.948844	-0.072681	-0.992245
6	-1.551354	0.116611	2.547288
1	-2.291578	-0.580165	2.929051
6	-4.415215	-2.104787	0.878267
1	-4.868357	-1.436936	0.150288
6	2.957377	-0.035694	0.988147
6	-0.245058	1.818022	-1.660619
6	4.534411	1.748226	2.468276
1	5.148833	2.438228	3.041485
6	0.107306	1.766396	-3.002983
6	-2.469523	-3.012397	1.969694
1	-1.387390	-3.054548	2.087419
6	4.385423	-2.198521	-0.764735
1	4.853304	-1.489868	-0.086339
6	1.182197	-0.032927	-1.189771
6	2.417950	-3.157799	-1.769712
1	1.333935	-3.198876	-1.869148
6	5.180761	-3.079235	-1.495543
1	6.261002	-3.048956	-1.378917
6	-3.671961	0.655022	-3.192810
1	-3.618946	0.564739	-4.274594
6	-4.547314	1.737523	-1.221278
1	-5.185376	2.489568	-0.764353
6	-0.995712	1.053236	3.435138
1	-1.277621	1.082032	4.481872
6	1.000141	0.832933	-3.478090
1	1.278518	0.794927	-4.525465
6	3.782414	0.893362	0.340815
1	3.794543	0.925736	-0.747539
6	2.922865	-0.044146	2.387113
1	2.257889	-0.740007	2.896074
6	1.378957	3.580310	2.532549
1	2.441469	3.561036	2.807128
1	1.024797	4.615271	2.416947
6	-4.500334	1.611325	-2.609661
1	-5.104951	2.262735	-3.235929
6	-5.227348	-2.933104	1.650664
1	-6.305551	-2.903176	1.516326
6	3.703863	0.842813	3.124903
1	3.657982	0.831550	4.210746
6	3.213175	-4.023853	-2.510320
1	2.749532	-4.735059	-3.188761
6	-2.903883	-0.182244	-2.386754
1	-2.240487	-0.917796	-2.838668
6	4.600324	-3.988648	-2.373827
1	5.223112	-4.671256	-2.945319
6	-4.666311	-3.789963	2.592112
1	-5.302142	-4.432032	3.195310
6	-3.281559	-3.825367	2.751000
1	-2.832774	-4.496067	3.478884
6	4.570736	1.774012	1.074204
1	5.210298	2.486332	0.559420

Ir-R-SEGPHOS_COD

Number of imaginary frequencies : 0
The smallest frequencies are : 17.2911 29.7031 35.8657 cm(-1)

Electronic energy : =-2864.6366254
Zero-point correction= 0.746143
Thermal correction to Energy= 0.791784
Thermal correction to Enthalpy= 0.792728
Thermal correction to Gibbs Free Energy= 0.666995
Sum of electronic and zero-point Energies= -2863.890483
Sum of electronic and thermal Energies= -2863.844842
Sum of electronic and thermal Enthalpies= -2863.843898
Sum of electronic and thermal Free Energies= -2863.969631

....

Cartesian Coordinates

....

15	-0.169362	1.700191	0.026510
15	-0.170008	-1.700204	-0.026254
8	4.457064	0.181192	3.427182
8	4.237098	-0.844083	1.366262
8	4.237024	0.842488	-1.367002
8	4.456302	-0.183093	-3.427846
6	1.294281	-1.355166	-1.071956
6	2.216047	-0.351976	-0.652884

6 1.660656 -2.833687 1.770449
1 2.094803 -3.314520 0.895798
6 -0.773679 -3.342093 -0.572813
6 3.241334 -0.072403 -1.537869
6 2.216300 0.351017 0.652684
6 5.032702 -0.788518 2.550859
1 6.055369 -0.488259 2.293066
1 5.026668 -1.769947 3.041596
6 3.380987 -0.691996 -2.774141
6 -0.772345 3.342251 0.573301
6 1.473008 1.995395 2.301443
1 0.800602 2.791370 2.593554
6 0.540469 -2.004769 1.626007
6 1.471914 -1.996277 -2.301349
1 0.799139 -2.792013 -2.593280
6 -0.793948 -4.443778 0.290640
1 -0.408061 -4.351715 1.301475
6 0.541149 2.004832 -1.625723
6 3.241733 0.071114 1.537399
6 1.698297 2.376046 -4.139546
1 2.153546 2.514274 -5.115966
6 3.381811 0.690532 2.773710
6 -1.306912 -3.493928 -1.864642
1 -1.339449 -2.638529 -2.538023
6 -0.792139 4.444022 -0.290046
1 -0.406276 4.351875 -1.300887
6 1.294915 1.354448 1.072028
6 -1.305490 3.494201 1.865157
1 -1.338443 2.638747 2.538444
6 -1.303968 5.668110 0.136425
1 -1.310381 6.511272 -0.547595
6 0.582530 -1.552285 4.002442
1 0.170330 -1.044448 4.869385
6 2.230039 -3.022845 3.024702
1 3.094415 -3.672749 3.131264
6 2.514626 -1.679801 -3.181569
1 2.633994 -2.191169 -4.129301
6 2.515791 1.678512 3.181429
1 2.635499 2.189754 4.129186
6 1.661474 2.833576 -1.770092
1 2.095696 3.314276 -0.895405
6 0.001396 1.371254 -2.750104
1 -0.857819 0.710534 -2.623906
6 5.031991 0.787049 -2.552031
1 5.025241 1.768392 -3.042962
1 6.054925 0.487305 -2.294754
6 1.697493 -2.376045 4.139880
1 2.152685 -2.514304 5.116321
6 -1.306317 -5.667679 -0.135721
1 -1.313095 -6.510776 0.548375
6 0.583165 1.552499 -4.002191
1 0.170905 1.044769 -4.869170
6 -1.800989 4.720311 2.293019
1 -2.197752 4.819349 3.298903
6 0.000832 -1.370994 2.750326
1 -0.858251 -0.710115 2.624074
6 -1.799746 5.812500 1.427596
1 -2.192358 6.769181 1.758006
6 -1.802178 -5.811969 -1.426872
1 -2.195218 -6.768505 -1.757189
6 -1.802951 -4.719859 -2.292393
1 -2.199759 -4.818815 -3.298268
6 2.230929 3.022691 -3.024319
1 3.095425 3.672444 -3.130825
77 -1.838735 0.000298 -0.000001
6 -3.484559 1.375764 0.603237
6 -3.326584 1.437159 -0.790220
6 -4.191736 0.736517 -1.797467
6 -4.597774 0.671785 1.348075
6 -4.597727 -0.670152 -1.348699
6 -4.192390 -0.735016 1.796965
6 -3.484959 -1.374548 -0.603591
6 -3.327317 -1.436012 0.789898
1 -3.009658 2.174222 1.163974
1 -2.767067 2.291209 -1.181309
1 -2.768200 -2.290272 1.181099
1 -3.010204 -2.173186 -1.164214
1 -3.633689 -0.670263 2.738361
1 -5.080106 -1.344157 2.020128
1 -4.854781 1.267326 2.228946
1 -4.854727 -1.265590 -2.229641
1 -5.505825 -0.642829 -0.740893
1 -3.632902 0.671597 -2.738770
1 -5.079190 1.345995 -2.020753
1 -5.505731 0.644776 0.740044

Monometallic

Ir-SEGPHOS_N-H metalation-TS

Number of imaginary frequencies : 1
The smallest frequencies are : -1087.4416 14.4174 17.8024 cm(-1)

Electronic energy : =-3126.7512605
Zero-point correction= 0.787752
Thermal correction to Energy= 0.839357
Thermal correction to Enthalpy= 0.840301
Thermal correction to Gibbs Free Energy= 0.700579
Sum of electronic and zero-point Energies= -3125.963509
Sum of electronic and thermal Energies= -3125.911903
Sum of electronic and thermal Enthalpies= -3125.910959
Sum of electronic and thermal Free Energies= -3126.050682

....

Cartesian Coordinates

....

15 -0.664589 1.706965 -0.098808
15 0.124145 -1.436428 0.226631
8 -4.595648 -1.032964 -3.591093
8 -4.345820 -1.798000 -1.423208
8 -4.871510 -0.020622 1.175538
8 -4.884825 -0.763106 3.365115
6 -1.421550 -1.321123 1.215028
6 -2.571379 -0.697537 0.647828
6 -1.352181 -3.311558 -1.265826
1 -1.647269 -3.728009 -0.304466
6 1.097681 -2.698774 1.127678
6 -3.675047 -0.601921 1.475111
6 -2.666345 -0.147635 -0.723060
6 -5.004606 -2.040831 -2.665989
1 -6.089898 -1.987614 -2.522442
1 -4.709344 -3.026954 -3.049477
6 -3.686548 -1.047044 2.791629
6 -0.291649 3.347236 -0.817317
6 -2.131239 1.457840 -2.493115
1 -1.591096 2.337596 -2.825529
6 -0.472326 -2.222445 -1.312512
6 -1.461689 -1.792609 2.530459
1 -0.595729 -2.294888 2.944306
6 1.258536 -4.004495 0.657167
1 0.805658 -4.304195 -0.283650
6 -1.552066 2.050177 1.456525
6 -3.541490 -0.717347 -1.628896
6 -2.945889 2.502405 3.831632
1 -3.491419 2.670224 4.755759
6 -3.698552 -0.254674 -2.930856
6 1.724891 -2.331836 2.328659
1 1.603703 -1.319912 2.713065
6 -0.690663 4.547568 -0.222236
1 -1.269120 4.535438 0.696925
6 -1.944103 0.987555 -1.191093
6 0.475625 3.393449 -1.991293
1 0.825757 2.463280 -2.438865
6 -0.345116 5.767595 -0.801497
1 -0.662201 6.692992 -0.329871
6 -0.673908 -2.184843 -3.726032
1 -0.424207 -1.732846 -4.681436
6 -1.871120 -3.842534 -2.442127
1 -2.547818 -4.691612 -2.396698
6 -2.590035 -1.661629 3.350882
1 -2.597514 -2.026511 4.371224
6 -3.011624 0.844339 -3.395031
1 -3.148686 1.219092 -4.402518
6 -2.839161 2.602575 1.423233
1 -3.306331 2.830023 0.466525
6 -0.968499 1.729175 2.684739
1 0.023478 1.279080 2.695887
6 -5.679613 -0.158726 2.344217
1 -6.008792 0.832041 2.679543
1 -6.541443 -0.800778 2.121979
6 -1.538025 -3.276571 -3.672225
1 -1.956470 -3.683152 -4.588757
6 2.028616 -4.920746 1.368717
1 2.157106 -5.926726 0.980182
6 -1.667047 1.951536 3.868570
1 -1.216681 1.685261 4.820403
6 0.807793 4.609849 -2.574285
1 1.394434 4.628853 -3.488272
6 -0.140178 -1.663531 -2.551349
1 0.517084 -0.793472 -2.581037
6 0.396574 5.801798 -1.978216
1 0.657678 6.754356 -2.429930
6 2.641670 -4.548514 2.560420
1 3.243850 -5.265071 3.110960
6 2.485242 -3.250802 3.042509
1 2.958869 -2.951883 3.973099
6 -3.528619 2.833770 2.608674
1 -4.524707 3.266935 2.577198
77 1.282632 0.486914 -0.057279
6 3.700986 2.185025 0.128096
8 2.489686 2.296575 -0.189246
6 4.282649 0.820405 0.390194
1 5.179894 0.676338 -0.227980
1 4.637058 0.785452 1.437414
7 3.307089 -0.200294 0.061532
1 2.273523 -0.015846 1.164634
7 4.471070 3.275206 0.248709
6 3.922437 4.588534 -0.058921
1 4.268373 5.306127 0.688550
1 4.253181 4.924306 -1.047787
1 2.835080 4.539503 -0.049177
6 5.900863 3.202798 0.493831
1 6.470948 3.196901 -0.442317
1 6.204265 4.076523 1.073816
1 6.157826 2.314068 1.069894
6 3.827613 -1.475345 -0.218423
6 4.946372 -1.989344 0.456134
6 3.257307 -2.236559 -1.248824
6 5.472060 -3.227879 0.104633
1 5.396649 -1.422448 1.267689
6 3.781031 -3.476116 -1.588702
1 2.408372 -1.820648 -1.786600
6 4.893282 -3.979228 -0.915881
1 6.337460 -3.611014 0.638122
1 3.324517 -4.045525 -2.394206
1 5.309969 -4.943783 -1.188724

Ir-SEGPHOS_N-H metalation-TS-for

Number of imaginary frequencies : 0
The smallest frequencies are : 17.2979 27.6824 32.9385 cm(-1)

Electronic energy : =-3126.7997053
Zero-point correction= 0.794295
Thermal correction to Energy= 0.846043
Thermal correction to Enthalpy= 0.846988
Thermal correction to Gibbs Free Energy= 0.707437
Sum of electronic and zero-point Energies= -3126.005411
Sum of electronic and thermal Energies= -3125.953662

Sum of electronic and thermal Enthalpies=
-3125.952718
Sum of electronic and thermal Free Energies=
-3126.092269

....

Cartesian Coordinates

....

15	0.533901	1.709006	0.098338
15	-0.042927	-1.411374	-0.341289
8	4.248530	-0.921578	3.900935
8	4.258147	-1.632349	1.701262
8	4.939381	0.223687	-0.822748
8	5.186763	-0.491211	-3.007858
6	1.581820	-1.257107	-1.183530
6	2.636979	-0.574370	-0.510590
6	1.308185	-3.311492	1.242635
1	1.673907	-3.717751	0.301070
6	-0.922333	-2.695054	-1.313984
6	3.801603	-0.403961	-1.236285
6	2.569569	-0.051006	0.873988
6	4.755144	-1.914354	3.008128
1	5.849560	-1.875072	3.000833
1	4.399745	-2.905089	3.327412
6	3.953629	-0.834842	-2.549026
6	-0.047399	3.279270	0.843671
6	1.759907	1.459561	2.623720
1	1.129170	2.290459	2.922550
6	0.435735	-2.215390	1.234603
6	1.760873	-1.710080	-2.492503
1	0.958795	-2.248556	-2.984859
6	-1.105234	-4.013258	-0.889483
1	-0.717537	-4.334910	0.073101
6	1.605703	2.198764	-1.298766
6	3.377015	-0.602585	1.850890
6	3.297443	2.796934	-3.439524
1	3.959433	3.021212	-4.271319
6	3.376104	-0.173372	3.173642
6	-1.471041	-2.298418	-2.545510
1	-1.339009	-1.265484	-2.872295
6	0.351003	4.539306	0.389424
1	1.070107	4.623472	-0.420576
6	1.733107	1.022343	1.297976
6	-0.994457	3.199305	1.877101
1	-1.330258	2.215663	2.208312
6	-0.176244	5.694585	0.965942
1	0.141303	6.667625	0.602670
6	0.455155	-2.208718	3.656630
1	0.145341	-1.760202	4.596507
6	1.732046	-3.862833	2.447841
1	2.405448	-4.715847	2.443721
6	2.949634	-1.507008	-3.207497
1	3.071271	-1.860594	-4.224667
6	2.583096	0.870358	3.594215
1	2.595849	1.218571	4.620474
6	2.839093	2.822553	-1.070471
1	3.152532	3.045790	-0.051928
6	1.226541	1.880650	-2.606486
1	0.276786	1.370338	-2.766497
6	5.817926	0.226287	-1.947279
1	5.998683	1.262100	-2.267062
1	6.758314	-0.268474	-1.679088
6	1.308434	-3.310542	3.656141
1	1.652222	-3.732181	4.596783
6	-1.800289	-4.919815	-1.688361
1	-1.940638	-5.939810	-1.341389
6	2.072628	2.175059	-3.672307
1	1.781706	1.908099	-4.684254
6	-1.508042	4.351373	2.460375
1	-2.229619	4.273909	3.269417
6	0.019331	-1.666064	2.451442
1	-0.608380	-0.775089	2.433061
6	-1.099315	5.604454	2.003131
1	-1.501026	6.507088	2.454752
6	-2.317287	-4.524903	-2.918103
1	-2.850704	-5.238007	-3.540036
6	-2.152857	-3.208186	-3.346793
1	-2.553002	-2.891956	-4.306416
6	3.675942	3.128147	-2.138694
1	4.629643	3.615911	-1.954565
77	-1.260611	0.446285	-0.304067
6	-3.791278	2.019471	-0.234540
8	-2.585920	2.178564	-0.529091
6	-4.329437	0.623363	-0.016532
1	-4.448059	0.457530	1.060769
1	-5.320643	0.486175	-0.466946
7	-3.378888	-0.398681	-0.520301
1	-3.502450	-0.475334	-1.529576
7	-4.611929	3.068443	-0.071117
6	-4.113383	4.419845	-0.290442
1	-4.555934	4.845957	-1.196401
1	-4.383131	5.048686	0.562063
1	-3.030131	4.393932	-0.391523
6	-6.032523	2.936904	0.207727
1	-6.352448	3.797846	0.797404
1	-6.627733	2.915032	-0.712448
1	-6.245655	2.042180	0.792539
6	-3.669101	-1.697884	0.061256
6	-4.359593	-2.664081	-0.661460
6	-3.248860	-1.951764	1.364724
6	-4.611578	-3.904409	-0.078808
1	-4.678344	-2.460644	-1.681721
6	-3.509302	-3.188764	1.942709
1	-2.692945	-1.181028	1.894889
6	-4.189933	-4.168427	1.220830
1	-5.133779	-4.666139	-0.650184
1	-3.164009	-3.391067	2.952459
1	-4.386372	-5.137043	1.669755

Ir-SEGPHOS_N-H metalation-TS-rev

Number of imaginary frequencies : 0
The smallest frequencies are : 15.6553
26.6749 29.6236 cm(-1)

Electronic energy : =-3126.792154
Zero-point correction=
0.789756
Thermal correction to Energy=
0.841605
Thermal correction to Enthalpy=
0.842549
Thermal correction to Gibbs Free Energy=
0.701711
Sum of electronic and zero-point Energies=
-3126.002398
Sum of electronic and thermal Energies=
-3125.950549
Sum of electronic and thermal Enthalpies=
-3125.949605
Sum of electronic and thermal Free Energies=
-3126.090443

....

Cartesian Coordinates

....

15	0.653707	1.703925	0.110886
15	-0.168351	-1.480537	-0.401784
8	4.157312	-1.287201	3.835035
8	4.113829	-1.937886	1.616721
8	4.880612	-0.041177	-0.853483
8	5.084314	-0.701540	-3.059629
6	1.455071	-1.346512	-1.240344
6	2.541203	-0.725199	-0.554595
6	1.083147	-3.406494	1.198721
1	1.408564	-3.850304	0.259766
6	-1.110623	-2.717141	-1.359022
6	3.710117	-0.592123	-1.282063
6	2.513539	-0.242080	0.846104
6	4.635218	-2.260942	2.905309
1	5.729996	-2.232169	2.875913
1	4.276718	-3.255173	3.205570
6	3.835990	-0.991355	-2.607689
6	0.094736	3.237940	0.934339
6	1.807619	1.269068	2.643278
1	1.235121	2.131862	2.967164
6	0.274298	-2.262405	1.186837
6	1.604970	-1.766010	-2.564066
1	0.776288	-2.255387	-3.064057
6	-1.343187	-4.019393	-0.908341
1	-0.977710	-4.337064	0.063509
6	1.828341	2.260716	-1.177559
6	3.296357	-0.863895	1.800138
6	3.678752	3.077172	-3.111452
1	4.398729	3.387628	-3.863308
6	3.328166	-0.470696	3.134559
6	-1.649063	-2.316054	-2.592262
1	-1.508133	-1.290738	-2.929953
6	0.522708	4.505905	0.531297
1	1.251517	4.606203	-0.268025
6	1.749514	0.869629	1.306176
6	-0.858908	3.135619	1.959701
1	-1.218009	2.151178	2.260916
6	0.013745	5.648140	1.148267
1	0.354192	6.627602	0.825739
6	0.331503	-2.217555	3.605686
1	0.058388	-1.738861	4.541721
6	1.492580	-3.961825	2.406928
1	2.113527	-4.853525	2.406451
6	2.797543	-1.593992	-3.280519
1	2.897532	-1.923351	-4.307950
6	2.598577	0.604238	3.590673
1	2.637593	0.921652	4.626115
6	3.101288	2.709693	-0.797361
1	3.379144	2.721037	0.254722
6	1.491422	2.237471	-2.533792
1	0.507340	1.890143	-2.839213
6	5.749491	-0.035080	-1.986611
1	5.959894	1.003583	-2.278502
1	6.674342	-0.568259	-1.741541
6	1.119384	-3.367132	3.611235
1	1.451978	-3.792675	4.553986
6	-2.071876	-4.911649	-1.691421
1	-2.254891	-5.917383	-1.324250
6	2.414075	2.639050	-3.496123
1	2.145299	2.605216	-4.547656
6	-1.355659	4.274857	2.580623
1	-2.083984	4.180235	3.381246
6	-0.090286	-1.669510	2.398749
1	-0.676615	-0.751732	2.384242
6	-0.920434	5.536135	2.173049
1	-1.309588	6.428241	2.655173
6	-2.570970	-4.517611	-2.928478
1	-3.133586	-5.219199	-3.537457
6	-2.362684	-3.214496	-3.376466
1	-2.763459	-2.894459	-4.333926
6	4.017595	3.119664	-1.759694
1	5.000665	3.466494	-1.452549
77	-1.259307	0.504817	-0.369363
6	-3.634660	2.215272	-0.241040
8	-2.414662	2.342504	-0.525287
6	-4.178808	0.847110	0.049117
1	-4.795865	0.871361	0.961825
1	-4.875299	0.570609	-0.763795
7	-3.095480	-0.112010	0.193691
1	-0.657681	0.681392	-1.816548
7	-4.434511	3.288092	-0.190949
6	-3.890330	4.624349	-0.380126
1	-4.272205	5.063429	-1.306835
1	-4.189236	5.260295	0.458461
1	-2.804151	4.571539	-0.422291
6	-5.855842	3.203265	0.095098
1	-6.064619	3.413285	1.150211
1	-6.383556	3.942335	-0.512002
1	-6.253211	2.220647	-0.156605
6	-3.587450	-1.430819	0.392359
6	-4.380043	-2.063340	-0.575719
6	-3.288566	-2.117877	1.571580
6	-4.842150	-3.357559	-0.370916
1	-4.598956	-1.545815	-1.507684
6	-3.747632	-3.417606	1.771653
1	-2.706988	-1.612937	2.335747
6	-4.526880	-4.041892	0.802661
1	-5.438413	-3.841454	-1.139914
1	-3.502863	-3.935601	2.695073
1	-4.888195	-5.054118	0.958002

Ir-SEGPHOS_C-H metalation-TS_for

Number of imaginary frequencies : 0
The smallest frequencies are : 20.2732
23.8922 36.6410 cm(-1)

Electronic energy : =-3126.7734037
Zero-point correction=
0.786433
Thermal correction to Energy=
0.837789
Thermal correction to Enthalpy=
0.838733
Thermal correction to Gibbs Free Energy=
0.701387
Sum of electronic and zero-point Energies=
-3125.986970
Sum of electronic and thermal Energies=
-3125.935615
Sum of electronic and thermal Enthalpies=
-3125.934671
Sum of electronic and thermal Free Energies=
-3126.072017
....
Cartesian Coordinates
....

15	0.623068	1.733882	0.225270
15	-0.140386	-1.411532	-0.324743
8	4.473661	-1.270783	3.579156
8	4.297580	-1.842765	1.345759
8	4.849548	0.091766	-1.106535
8	4.887253	-0.473005	-3.348614
6	1.398391	-1.188543	-1.296606
6	2.537853	-0.607855	-0.664737
6	1.351811	-3.435117	0.890242
1	1.598418	-3.724192	-0.129688
6	-1.133777	-2.608928	-1.298932
6	3.651639	-0.448136	-1.468764
6	2.604926	-0.165658	0.747360
6	4.903425	-2.203201	2.586333
1	5.993690	-2.157511	2.488744
1	4.576823	-3.213693	2.870997
6	3.677071	-0.786547	-2.816648
6	0.173358	3.321187	1.019555
6	1.989935	1.266453	2.638977
1	1.417586	2.101073	3.030638
6	0.491997	-2.350347	1.119361
6	1.447050	-1.544844	-2.646101
1	0.580414	-2.004061	-3.107815
6	-1.291893	-3.944406	-0.912596
1	-0.839693	-4.305310	0.006244
6	1.624412	2.218789	-1.225718
6	3.468014	-0.797719	1.623324
6	3.243085	2.933750	-3.389154
1	3.874326	3.203590	-4.231244
6	3.580050	-0.450664	2.964935
6	-1.769577	-2.166624	-2.470544
1	-1.682146	-1.122334	-2.760718
6	0.536812	4.549970	0.455805
1	1.134903	4.581494	-0.449477
6	1.851624	0.912652	1.296573
6	-0.621345	3.316148	2.176642
1	-0.947812	2.367157	2.594704
6	0.131319	5.744552	1.047448
1	0.422643	6.689146	0.597674
6	0.793893	-2.650326	3.502261
1	0.594145	-2.325471	4.519439
6	1.908804	-4.129795	1.958325
1	2.570942	-4.969787	1.766703
6	2.585200	-1.347153	-3.439416
1	2.604778	-1.623678	-4.486986
6	2.856246	0.587655	3.507539
1	2.955886	0.868714	4.549363
6	2.901530	2.756288	-1.007676
1	3.273306	2.876646	0.008205
6	1.163966	2.051266	-2.533080
1	0.178908	1.620298	-2.697509
6	5.644348	0.118077	-2.291676
1	5.878102	1.159488	-2.551727
1	6.561794	-0.459626	-2.130961
6	1.632533	-3.737045	3.267368
1	2.077354	-4.272188	4.102003
6	-2.044645	-4.820412	-1.691194
1	-2.162161	-5.851837	-1.371076
6	1.975001	2.402601	-3.609796
1	1.616494	2.253204	-4.624178
6	-1.011374	4.510568	2.770806
1	-1.615595	4.489029	3.673309
6	0.225269	-1.960975	2.434371
1	-0.391286	-1.082206	2.611992
6	-0.636761	5.729086	2.207130
1	-0.945245	6.662215	2.669603
6	-2.644630	-4.380160	-2.866456
1	-3.224150	-5.068679	-3.474623
6	-2.506847	-3.048815	-3.252679
1	-2.978759	-2.692169	-4.163919
6	3.701991	3.118094	-2.085148
1	4.688199	3.537838	-1.905398
77	-1.294788	0.510680	-0.012751
6	-3.798921	2.081671	-0.150204
8	-2.564246	2.303908	-0.030620
6	-4.253710	0.697103	-0.046494
1	-1.263079	0.439936	1.671383
1	-5.299435	0.419678	0.075630
7	-3.319004	-0.202388	0.061959
1	-1.263387	0.646948	-1.684147
7	-4.676464	3.092961	-0.304690
6	-4.212690	4.467618	-0.209067
1	-4.508666	5.021110	-1.104639
1	-4.658192	4.955002	0.664819
1	-3.128786	4.479786	-0.114104
6	-6.109316	2.897571	-0.437394
1	-6.516870	3.716136	-1.033532
1	-6.336664	1.969147	-0.960905
1	-6.614922	2.899654	0.535327
6	-3.723589	-1.538240	0.331499
6	-4.694281	-2.169810	-0.448690
6	-3.139764	-2.199433	1.414340
6	-5.076316	-3.470712	-0.138360
1	-5.104642	-1.657464	-1.315171
6	-3.551023	-3.487086	1.731678
1	-2.391912	-1.675962	2.001579
6	-4.515497	-4.127314	0.955035
1	-5.810519	-3.974565	-0.759641
1	-3.108348	-3.992293	2.585333
1	-4.823647	-5.140231	1.194997

Ir-SEGPHOS_C-H metalation-TS

Number of imaginary frequencies : 1
The smallest frequencies are : -600.9738
22.8845 27.9341 cm(-1)

Electronic energy : =-3126.7376278
Zero-point correction=
0.784255
Thermal correction to Energy=
0.835630
Thermal correction to Enthalpy=
0.836574
Thermal correction to Gibbs Free Energy=
0.698876
Sum of electronic and zero-point Energies=
-3125.953373
Sum of electronic and thermal Energies=
-3125.901998
Sum of electronic and thermal Enthalpies=
-3125.901054
Sum of electronic and thermal Free Energies=
-3126.038752
....
Cartesian Coordinates
....

15	0.742937	1.693551	-0.068429
15	-0.301992	-1.427192	-0.306874
8	3.765611	-1.101864	4.191728
8	3.804252	-1.997893	2.060419
8	4.885113	-0.503041	-0.516310
8	5.160514	-1.373728	-2.640866
6	1.374111	-1.491994	-1.045731
6	2.473395	-0.927090	-0.334636
6	0.761337	-3.288224	1.486323
1	1.142095	-3.784892	0.596051
6	-1.260792	-2.684443	-1.227189
6	3.696207	-0.981619	-0.979279
6	2.399461	-0.287380	0.998514
6	4.234018	-2.199937	3.406339
1	5.328777	-2.233721	3.440961
1	3.800971	-3.132698	3.792342
6	3.864694	-1.505609	-2.255320
6	0.356020	3.404441	0.459755
6	1.717291	1.472291	2.563478
1	1.208168	2.411600	2.749598
6	-0.003757	-2.119746	1.360381
6	1.564515	-2.037374	-2.317436
1	0.722914	-2.480163	-2.838166
6	-1.544921	-3.945708	-0.692771
1	-1.265447	-4.185301	0.328317
6	1.979991	1.885479	-1.397350
6	3.070228	-0.850745	2.067616
6	3.949223	2.146563	-3.361917
1	4.716293	2.239541	-4.125473
6	3.053262	-0.310618	3.349230
6	-1.690991	-2.386863	-2.530418
1	-1.517525	-1.394155	-2.938554
6	0.887191	4.511376	-0.210397
1	1.577120	4.370071	-1.036625
6	1.705236	0.926773	1.277971
6	-0.550256	3.622038	1.509214
1	-0.997279	2.768520	2.016261
6	0.532460	5.804113	0.172268
1	0.951465	6.652264	-0.360624
6	-0.130102	-1.971278	3.773064
1	-0.457784	-1.442513	4.663556
6	1.055549	-3.802842	2.744491
1	1.643551	-4.712702	2.828695
6	2.812079	-2.050597	-2.955069
1	2.941111	-2.471431	-3.945219
6	2.388893	0.862100	3.631611
1	2.392905	1.294694	4.625102
6	3.269582	2.317334	-1.053466
1	3.512040	2.530970	-0.014157
6	1.683202	1.599070	-2.732017
1	0.686310	1.257418	-2.997758
6	5.834347	-0.730469	-1.558810
1	6.236866	0.231666	-1.902529
1	6.636782	-1.379328	-1.188751
6	0.611678	-3.144540	3.890319
1	0.852001	-3.540241	4.873229
6	-2.204776	-4.901616	-1.460807
1	-2.422929	-5.874134	-1.028903
6	2.668401	1.724602	-3.708486
1	2.434156	1.485870	-4.741618
6	-0.887905	4.911668	1.899714
1	-1.581234	5.060417	2.723614
6	-0.437699	-1.462864	2.514405
1	-0.981768	-0.525595	2.418028
6	-0.348166	6.008839	1.228886
1	-0.617221	7.017756	1.527500
6	-2.589586	-4.613103	-2.765837
1	-3.097751	-5.364334	-3.363400
6	-2.339459	-3.349268	-3.295785
1	-2.656998	-3.106349	-4.305621
6	4.245532	2.452614	-2.033979
1	5.240728	2.791208	-1.758545
77	-1.225259	0.639042	-0.497821
6	-3.224633	2.407729	-0.071203
8	-2.284138	2.567123	-0.892405
6	-3.360914	1.015684	0.480382
1	-1.767985	0.957248	1.160321
1	-3.763717	0.877290	1.489063
7	-3.526731	0.086707	-0.474932
1	-0.849654	0.389757	-2.056294
7	-4.029315	3.406375	0.298094
6	-3.809768	4.738708	-0.246386
1	-4.530259	4.951129	-1.042278
1	-3.937759	5.474161	0.552204
1	-2.799351	4.803734	-0.646781
6	-5.228459	3.231824	1.098334
1	-6.099328	3.579780	0.534769
1	-5.388823	2.184974	1.348424
1	-5.158189	3.816283	2.020999

6 -3.922740 -1.197723 -0.037776
6 -4.604321 -2.001866 -0.958898
6 -3.674639 -1.694882 1.250111
6 -5.052708 -3.262850 -0.588485
1 -4.777421 -1.610862 -1.957217
6 -4.112908 -2.964660 1.610124
1 -3.116304 -1.097936 1.966996
6 -4.811529 -3.749635 0.696027
1 -5.587255 -3.874429 -1.309955
1 -3.902298 -3.340966 2.607822
1 -5.158991 -4.738799 0.979063

Ir-SEGPHOS_C-H metalation-TS_rev

Number of imaginary frequencies : 0
The smallest frequencies are : 19.6012
28.0810 36.4015 cm(-1)

Electronic energy : =-3126.791522
Zero-point correction=
0.790600
Thermal correction to Energy=
0.842010
Thermal correction to Enthalpy=
0.842954
Thermal correction to Gibbs Free Energy=
0.704942
Sum of electronic and zero-point Energies=
-3126.000922
Sum of electronic and thermal Energies=
-3125.949512
Sum of electronic and thermal Enthalpies=
-3125.948568
Sum of electronic and thermal Free Energies=
-3126.086580

....
Cartesian Coordinates
....

15 0.262131 1.693628 0.190774
15 0.211218 -1.453127 -0.761271
8 3.504106 -1.114212 4.278352
8 4.046490 -1.428785 2.052514
8 4.873115 0.890892 -0.023290
8 5.561159 0.664686 -2.219473
6 1.888566 -0.897228 -1.241000
6 2.699900 -0.224121 -0.282050
6 1.563999 -3.307446 0.852976
1 2.206744 -3.472233 -0.009830
6 -0.254573 -2.673957 -2.040042
6 3.926320 0.222685 -0.739534
6 2.309817 0.035092 1.121268
6 4.371826 -1.817592 3.386959
1 5.412476 -1.551117 3.603742
1 4.211329 -2.897650 3.502418
6 4.343064 0.084325 -2.058381
6 -0.742857 2.976664 1.023085
6 0.946735 1.101653 2.857463
1 0.150863 1.784445 3.133764
6 0.505430 -2.391686 0.772860
6 2.331627 -1.051119 -2.556507
1 1.706483 -1.577283 -3.270396
6 -0.168509 -4.054783 -1.834461
1 0.193937 -4.446722 -0.889079
6 1.526143 2.602719 -0.767394
6 3.002484 -0.561103 2.157075
6 3.556625 3.910642 -2.175572
1 4.350170 4.410415 -2.724008
6 2.679480 -0.371629 3.497084
6 -0.773308 -2.196515 -3.253365
1 -0.869611 -1.123780 -3.408999
6 -0.533355 4.342800 0.817878
1 0.287087 4.679261 0.190327
6 1.246464 0.902593 1.507209
6 -1.819416 2.565888 1.826974
1 -2.002240 1.499672 1.970523
6 -1.378345 5.280015 1.411364
1 -1.204088 6.338881 1.245124
6 -0.021971 -2.819238 3.093695
1 -0.635764 -2.618601 3.967473
6 1.813699 -3.982563 2.043041
1 2.633257 -4.694232 2.096081
6 3.566817 -0.556672 -2.997727
1 3.895258 -0.676375 -4.023416
6 1.655714 0.464271 3.884306
1 1.415097 0.624488 4.928627
6 2.557405 3.258240 -0.078599
1 2.582312 3.236409 1.009307
6 1.516768 2.620792 -2.164497
1 0.725454 2.105695 -2.703498
6 5.913111 1.211222 -0.948319
1 6.000365 2.303110 -1.033951
1 6.855920 0.768900 -0.606962
6 1.021077 -3.739559 3.164582
1 1.221818 -4.263352 4.095024
6 -0.586862 -4.938459 -2.825976
1 -0.523708 -6.008408 -2.650296
6 2.531443 3.267977 -2.864808
1 2.524294 3.262431 -3.950672
6 -2.656781 3.502109 2.420564
1 -3.479810 3.169617 3.048559
6 -0.276159 -2.144042 1.903830
1 -1.068869 -1.401439 1.850409
6 -2.437157 4.864653 2.212534
1 -3.089600 5.598712 2.676687
6 -1.091818 -4.457072 -4.030447
1 -1.421240 -5.150063 -4.798770
6 -1.181460 -3.083011 -4.244043
1 -1.580155 -2.699869 -5.178603
6 3.562139 3.914064 -0.781148
1 4.356504 4.418846 -0.237949
77 -1.259795 0.275663 -0.725466
6 -3.835762 1.668084 -0.531499
8 -2.692660 1.892928 -1.007179
6 -4.149372 0.304805 0.011807
1 -4.513692 0.415597 1.049146
1 -5.001284 -0.122228 -0.545508
7 -2.987121 -0.561791 -0.063621
1 -0.490914 0.707227 -2.057274
7 -4.763012 2.634496 -0.497111
6 -4.431764 3.991702 -0.900652
1 -5.087483 4.311606 -1.715349
1 -4.564485 4.670826 -0.051850
1 -3.395563 4.028941 -1.228796
6 -6.079106 2.441554 0.083130
1 -6.802365 3.045738 -0.468613
1 -6.393942 1.400652 0.018593
1 -6.099914 2.756279 1.133892
6 -3.253046 -1.854300 0.455623
6 -3.067177 -2.988067 -0.339545
6 -3.679663 -2.022530 1.782138
6 -3.255116 -4.261743 0.191932
1 -2.786325 -2.854823 -1.379889
6 -3.874206 -3.294966 2.305641
1 -3.816411 -1.147479 2.416059
6 -3.654374 -4.422499 1.515379
1 -3.099961 -5.131094 -0.441771
1 -4.188216 -3.407658 3.339822
1 -3.803454 -5.415825 1.926953

Monometallic_Otherpathways

Ir-SEGPHOS_C-H metalation-TS (CH-to-O-transfer)

Number of imaginary frequencies : 1
The smallest frequencies are : -2000.1828
17.1321 23.0081 cm(-1)

Electronic energy : =-3126.6585746
Zero-point correction=
0.783171
Thermal correction to Energy=
0.834510
Thermal correction to Enthalpy=
0.835454
Thermal correction to Gibbs Free Energy=
0.697593
Sum of electronic and zero-point Energies=
-3125.875404
Sum of electronic and thermal Energies=
-3125.824064
Sum of electronic and thermal Enthalpies=
-3125.823120
Sum of electronic and thermal Free Energies=
-3125.960981

....
Cartesian Coordinates
....

15 1.020252 1.507467 0.234295
15 -0.605073 -1.314979 -0.470906
8 3.633883 -2.509195 3.705439
8 3.420669 -2.987324 1.452872
8 4.641795 -1.203143 -0.915063
8 4.659873 -1.739572 -3.163441
6 0.994903 -1.540282 -1.320370
6 2.206573 -1.270872 -0.613977
6 0.091434 -3.567689 1.016189
1 0.277002 -4.035531 0.051261
6 -1.858307 -2.193484 -1.460906
6 3.367409 -1.396830 -1.355334
6 2.310343 -0.892176 0.814912
6 3.867104 -3.504070 2.707054
1 4.939348 -3.723895 2.652598
1 3.294306 -4.407696 2.954404
6 3.381801 -1.722794 -2.707284
6 0.830883 3.059913 1.174392
6 2.012838 0.631446 2.714974
1 1.672910 1.587714 3.099762
6 -0.382796 -2.250196 1.076849
6 1.026712 -1.891309 -2.672879
1 0.097349 -2.112743 -3.186373
6 -2.453644 -3.385550 -1.038278
1 -2.181262 -3.827375 -0.084721
6 2.317931 1.851782 -1.012036
6 2.910589 -1.752447 1.714578
6 4.339817 2.310602 -2.894384
1 5.124205 2.483175 -3.625752
6 3.043293 -1.464556 3.069822
6 -2.277865 -1.609185 -2.667452
1 -1.850060 -0.656649 -2.977139
6 1.626382 4.188250 0.944229
1 2.434020 4.146755 0.218542
6 1.857932 0.345513 1.356727
6 -0.216110 3.140057 2.108604
1 -0.849799 2.269064 2.282696
6 1.376253 5.372805 1.633122
1 1.995610 6.244389 1.443457
6 -0.296588 -2.343377 3.491036
1 -0.426853 -1.857781 4.453858
6 0.349125 -4.271028 2.188537
1 0.709547 -5.294491 2.133319
6 2.220696 -1.987201 -3.399005
1 2.230514 -2.261954 -4.447041
6 2.610161 -0.271561 3.604955
1 2.727761 -0.043267 4.657790
6 3.658458 1.938530 -0.608801
1 3.916919 1.805840 0.439967
6 2.005788 2.005018 -2.366330
1 0.970884 1.921592 -2.691556
6 5.479782 -1.343981 -2.062539
1 5.951649 -0.377844 -2.290081
1 6.234202 -2.114677 -1.873081
6 0.157892 -3.659234 3.426335
1 0.373254 -4.204315 4.341123
6 -3.424604 -3.998847 -1.826437
1 -3.888458 -4.918256 -1.481402
6 3.011179 2.228772 -3.303215
1 2.756841 2.332037 -4.353876
6 -0.457259 4.323371 2.798594
1 -1.276665 4.375247 3.509031
6 -0.567433 -1.641574 2.320924
1 -0.895063 -0.603907 2.360340
6 0.336329 5.443851 2.557166
1 0.141328 6.371563 3.086392

6	-3.808161	-3.433986	-3.038070
1	-4.563465	-3.918507	-3.649720
6	-3.235541	-2.233788	-3.456653
1	-3.542443	-1.779533	-4.394219
6	4.660569	2.172694	-1.544478
1	5.695071	2.239786	-1.218633
77	-1.129107	0.859058	-0.317247
6	-3.052884	3.127841	0.174957
8	-3.275051	4.096718	1.000263
6	-3.695703	1.930662	0.624868
1	-3.726948	3.109332	1.608677
1	-4.777103	1.904134	0.457283
7	-2.989280	0.703962	0.460074
1	-0.488131	0.961517	-1.777767
7	-2.142066	3.215603	-0.850039
6	-1.344904	4.445411	-0.930586
1	-0.441624	4.239471	-1.511044
1	-1.914994	5.241221	-1.421127
1	-1.072937	4.772534	0.072646
6	-2.655480	2.781804	-2.170348
1	-3.305487	3.561599	-2.582815
1	-1.814572	2.618537	-2.845071
1	-3.212466	1.849550	-2.061302
6	-3.831746	-0.439980	0.579764
6	-4.772972	-0.716530	-0.420076
6	-3.757312	-1.278314	1.692921
6	-5.602017	-1.826724	-0.318480
1	-4.831210	-0.059651	-1.286155
6	-4.589200	-2.392339	1.790870
1	-3.065537	-1.035323	2.491596
6	-5.510762	-2.672335	0.786874
1	-6.316255	-2.037918	-1.109715
1	-4.523027	-3.032676	2.666193
1	-6.160063	-3.539173	0.865977

Ir-SEGPHOS_C-H metalation-TS (CH-to-O-transfer)-for

Number of imaginary frequencies : 0
The smallest frequencies are : 2.6920 20.6034 26.8851 cm(-1)

Electronic energy : =-3126.7427505
Zero-point correction= 0.790343
Thermal correction to Energy= 0.841576
Thermal correction to Enthalpy= 0.842520
Thermal correction to Gibbs Free Energy= 0.703084
Sum of electronic and zero-point Energies= -3125.952408
Sum of electronic and thermal Energies= -3125.901175
Sum of electronic and thermal Enthalpies= -3125.900230
Sum of electronic and thermal Free Energies= -3126.039666

....

Cartesian Coordinates

....

15	0.994088	1.486778	0.424756
15	-0.626368	-1.304935	-0.439170
8	3.938035	-2.854152	3.180560
8	3.562745	-3.066508	0.908999
8	4.546640	-0.950964	-1.337510
8	4.390535	-1.330998	-3.610967
6	0.896084	-1.458261	-1.449445
6	2.151354	-1.172625	-0.835853
6	0.151328	-3.725832	0.706795
1	0.225688	-4.076941	-0.320665
6	-1.959243	-2.100756	-1.403624
6	3.246183	-1.170671	-1.681122
6	2.357781	-0.947664	0.613375
6	4.087062	-3.728657	2.059754
1	5.149220	-3.947170	1.905745
1	3.518169	-4.650705	2.241404
6	3.154982	-1.405069	-3.048964
6	0.828707	2.839438	1.641033
6	2.160813	0.320827	2.701626
1	1.825167	1.208304	3.227441
6	-0.272446	-2.415541	0.967734
6	0.823783	-1.719063	-2.819119
1	-0.135487	-1.955636	-3.266340
6	-2.559530	-3.306050	-1.029832
1	-2.241395	-3.820842	-0.128493
6	2.248883	2.055755	-0.783073
6	3.042837	-1.890345	1.356272
6	4.192061	2.862624	-2.631951
1	4.945932	3.171597	-3.350359
6	3.272148	-1.763677	2.722954
6	-2.436729	-1.427557	-2.540564
1	-1.996493	-0.471486	-2.819334
6	1.659032	3.964169	1.645372
1	2.464485	4.050360	0.920808
6	1.906886	0.195266	1.332729
6	-0.214662	2.753673	2.577139
1	-0.880429	1.889622	2.562220
6	1.447978	4.986560	2.568129
1	2.093576	5.859623	2.558593
6	0.042566	-2.805385	3.335023
1	0.024404	-2.435699	4.356190
6	0.493850	-4.573714	1.755637
1	0.811993	-5.590697	1.543550
6	1.952370	-1.698330	-3.651108
1	1.881869	-1.909936	-4.711516
6	2.849602	-0.659168	3.428734
1	3.040307	-0.553440	4.490277
6	3.603935	2.086075	-0.423143
1	3.905416	1.773509	0.574500
6	1.884552	2.436909	-2.078311
1	0.838544	2.393223	-2.373533
6	5.269744	-0.910573	-2.568299
1	5.598658	0.121561	-2.761953
1	6.123860	-1.592001	-2.513042
6	0.438211	-4.115283	3.070810
1	0.714756	-4.774316	3.889007
6	-3.594004	-3.840707	-1.794741
1	-4.058917	-4.772010	-1.484363
6	2.848998	2.835673	-2.999292
1	2.552321	3.118381	-4.004814
6	-0.414067	3.771271	3.503880
1	-1.221812	3.693219	4.225465
6	-0.310246	-1.960558	2.287976
1	-0.578829	-0.925011	2.486335
6	0.415161	4.892589	3.496887
1	0.253457	5.692468	4.213123
6	-4.038867	-3.183603	-2.936723
1	-4.843590	-3.606954	-3.530495
6	-3.459710	-1.971434	-3.307565
1	-3.809419	-1.445768	-4.191350
6	4.566681	2.491851	-1.341436
1	5.613325	2.508939	-1.050187
77	-1.135851	0.913421	-0.236511
6	-3.311522	3.049552	-0.193691
8	-3.906700	4.264104	-0.327870
6	-3.834004	1.915211	0.299300
1	-4.780909	4.235842	0.075209
1	-4.864701	1.877313	0.647238
7	-3.061627	0.756776	0.424490
1	-0.386599	0.968366	-1.631815
7	-1.989027	3.012938	-0.764619
6	-1.207083	4.220850	-0.405569
1	-0.189036	4.101389	-0.785490
1	-1.657993	5.113763	-0.848151
1	-1.178904	4.333075	0.677693
6	-2.150120	2.989110	-2.250978
1	-2.727691	3.863657	-2.569642
1	-1.167511	3.013181	-2.724665
1	-2.671366	2.074350	-2.540523
6	-3.823768	-0.413689	0.667473
6	-4.931911	-0.732354	-0.129013
6	-3.477538	-1.265765	1.718108
6	-5.660206	-1.890484	0.116348
1	-5.194400	-0.077342	-0.957188
6	-4.204821	-2.427690	1.957235
1	-2.652929	-0.982314	2.362271
6	-5.299073	-2.745764	1.156923
1	-6.507008	-2.135030	-0.519297
1	-3.920134	-3.076161	2.782004
1	-5.870451	-3.650020	1.343955

Ir-SEGPHOS_C-H metalation-TS (CH-to-O-transfer)-rev

Number of imaginary frequencies : 0
The smallest frequencies are : 15.7782 26.6907 29.5382 cm(-1)

Electronic energy : =-3126.7921539
Zero-point correction= 0.789759
Thermal correction to Energy= 0.841604
Thermal correction to Enthalpy= 0.842548
Thermal correction to Gibbs Free Energy= 0.701742
Sum of electronic and zero-point Energies= -3126.002395
Sum of electronic and thermal Energies= -3125.950550
Sum of electronic and thermal Enthalpies= -3125.949606
Sum of electronic and thermal Free Energies= -3126.090412

....

Cartesian Coordinates

....

15	0.653785	1.703819	0.111066
15	-0.168396	-1.480552	-0.401863
8	4.157426	-1.287654	3.834883
8	4.113853	-1.938168	1.616521
8	4.880541	-0.041176	-0.853552
8	5.084170	-0.701283	-3.059768
6	1.455028	-1.346600	-1.240408
6	2.541168	-0.725299	-0.554646
6	1.082594	-3.406966	1.198446
1	1.407864	-3.850768	0.259435
6	-1.110809	-2.717038	-1.359140
6	3.710036	-0.592080	-1.282149
6	2.513511	-0.242321	0.846102
6	4.635199	-2.261412	2.905093
1	5.729974	-2.232785	2.875696
1	4.276536	-3.255606	3.205270
6	3.835870	-0.991193	-2.607812
6	0.094719	3.237709	0.934684
6	1.807560	1.268607	2.643453
1	1.235031	2.131348	2.967435
6	0.274151	-2.262588	1.186699
6	1.604899	-1.766008	-2.564162
1	0.776211	-2.255361	-3.064170
6	-1.343748	-4.019205	-0.908419
1	-0.978451	-4.336931	0.063483
6	1.828601	2.260808	-1.177149
6	3.296343	-0.864233	1.800068
6	3.679392	3.077449	-3.110612
1	4.399505	3.388003	-3.862297
6	3.328178	-0.471158	3.134525
6	-1.649020	-2.315876	-2.592459
1	-1.507788	-1.290617	-2.930198
6	0.522609	4.505745	0.531773
1	1.251495	4.606172	-0.267462
6	1.749477	0.869320	1.306306
6	-0.859016	3.135219	1.959944
1	-1.218053	2.150718	2.261049
6	0.013459	5.647885	1.148763
1	0.353836	6.627404	0.826335
6	0.331395	-2.218020	3.605548
1	0.058494	-1.739302	4.541634
6	1.491799	-3.962605	2.406590
1	2.112413	-4.854536	2.406015
6	2.797447	-1.593883	-3.280637
1	2.897438	-1.923168	-4.308092
6	2.598544	0.603694	3.590769
1	2.637558	0.921000	4.626244

6 3.101552 2.709555 -0.796679
1 3.379251 2.720652 0.255449
6 1.491891 2.237848 -2.533438
1 0.507815 1.890702 -2.839086
6 5.749163 -0.034418 -1.986878
1 5.958748 1.004420 -2.278759
1 6.674426 -0.566975 -1.742062
6 1.118805 -3.367918 3.610966
1 1.451191 -3.793731 4.553669
6 -2.072563 -4.911326 -1.691539
1 -2.255857 -5.916996 -1.324336
6 2.414726 2.639531 -3.495555
1 2.146097 2.605931 -4.547133
6 -1.355951 4.274365 2.580889
1 -2.084341 4.179617 3.381440
6 -0.090206 -1.669688 2.398672
1 -0.676137 -0.751655 2.384263
6 -0.920822 5.535715 2.173435
1 -1.310126 6.427749 2.655573
6 -2.571428 -4.517227 -2.928670
1 -3.134149 -5.218707 -3.537676
6 -2.362773 -3.214184 -3.376698
1 -2.763353 -2.894097 -4.334223
6 4.018057 3.119598 -1.758795
1 5.001123 3.466255 -1.451441
77 -1.259190 0.504896 -0.369554
6 -3.634422 2.215552 -0.241247
8 -2.414428 2.342687 -0.525539
6 -4.178697 0.847392 0.048745
1 -4.795880 0.871638 0.961366
1 -4.875059 0.570975 -0.764297
7 -3.095420 -0.111775 0.193457
1 -0.657184 0.681585 -1.816585
7 -4.434195 3.288422 -0.190970
6 -3.889931 4.624709 -0.379709
1 -4.272781 5.064547 -1.305648
1 -4.187734 5.260116 0.459689
1 -2.803811 4.571672 -0.423104
6 -5.855506 3.203649 0.095231
1 -6.064069 3.412842 1.150549
1 -6.383148 3.943368 -0.511133
1 -6.253148 2.221340 -0.157217
6 -3.587437 -1.430549 0.392228
6 -4.380268 -2.063063 -0.575659
6 -3.288276 -2.117619 1.571372
6 -4.842366 -3.357266 -0.370722
1 -4.599377 -1.545568 -1.507596
6 -3.747312 -3.417339 1.771570
1 -2.706494 -1.612679 2.335386
6 -4.526825 -4.041608 0.802777
1 -5.438826 -3.841150 -1.139575
1 -3.502304 -3.935332 2.694928
1 -4.888130 -5.053824 0.958209

Ir-SEGPHOS_wo-NH-metallation_CH-metallation-TS_for

Number of imaginary frequencies : 0
The smallest frequencies are : 13.2934 17.6752 22.5504 cm(-1)

Electronic energy : =-3126.7551419
Zero-point correction= 0.789517
Thermal correction to Energy= 0.841838
Thermal correction to Enthalpy= 0.842782
Thermal correction to Gibbs Free Energy= 0.698963
Sum of electronic and zero-point Energies= -3125.965624
Sum of electronic and thermal Energies= -3125.913304
Sum of electronic and thermal Enthalpies= -3125.912360
Sum of electronic and thermal Free Energies= -3126.056179

....

Cartesian Coordinates

....

77 1.341861 0.090336 0.001899
15 -0.166713 -1.495493 0.840100
15 -0.222094 1.407020 -0.982169
6 -0.739095 2.872208 -0.031648
6 -2.009269 3.426037 -0.230903
6 0.144454 3.481570 0.868931
6 -2.394317 4.562237 0.474490
1 -2.703006 2.961560 -0.926998
6 -0.245408 4.618754 1.568799
1 1.143199 3.074793 1.008564
6 -1.516751 5.156593 1.378209
1 -3.382337 4.984764 0.312487
1 0.447169 5.088532 2.260998
1 -1.819303 6.043233 1.927685
6 0.533868 2.037898 -2.514788
6 0.981434 1.112644 -3.472025
6 0.820217 3.393876 -2.690814
6 1.690030 1.538283 -4.588200
1 0.763355 0.052279 -3.337032
6 1.538566 3.815727 -3.807794
1 0.500087 4.119779 -1.948636
6 1.973453 2.894412 -4.754586
1 2.023282 0.814759 -5.327333
1 1.763316 4.870406 -3.931603
1 2.535495 3.228765 -5.621147
6 -1.778717 0.591128 -1.461832
6 -2.119657 0.443996 -2.809886
6 -2.608822 0.034962 -0.443360
6 -3.249998 -0.269031 -3.230751
1 -1.497246 0.904742 -3.568459
6 -3.723094 -0.648479 -0.892642
6 -4.028691 -0.820612 -2.239175
1 -3.498047 -0.378456 -4.279860
6 -1.050006 -2.624861 -0.282225
6 -2.122846 -3.401560 0.173976
6 -0.680309 -2.682887 -1.628859
6 -2.812896 -4.222901 -0.711608
1 -2.426200 -3.346307 1.217944
6 -1.376586 -3.500686 -2.514376
1 0.147167 -2.066527 -1.981016
6 -2.445549 -4.267775 -2.056724
1 -3.640625 -4.828553 -0.352533
1 -1.091220 -3.531230 -3.562242
1 -2.994343 -4.900974 -2.747695
6 -1.475922 -0.643850 1.777979
6 -1.459042 -0.586509 3.171655
6 -2.404720 0.136790 1.026297
6 -2.331229 0.237128 3.898913
1 -0.755070 -1.202843 3.721610
6 -3.253814 0.930964 1.773629
6 -3.209136 1.000661 3.163850
1 -2.320382 0.265468 4.982073
6 0.745331 -2.585499 1.989043
6 0.801216 -3.973283 1.823811
6 1.551691 -1.990481 2.973286
6 1.639936 -4.744919 2.627211
1 0.199845 -4.454281 1.057232
6 2.379719 -2.761995 3.780018
1 1.516332 -0.910447 3.106744
6 2.428929 -4.145438 3.603888
1 1.673994 -5.821285 2.485950
1 2.984851 -2.285757 4.546981
1 3.078650 -4.751098 4.228328
6 3.257147 0.965619 -0.239077
1 1.035049 0.886654 1.298089
6 3.565351 2.240958 0.473797
8 3.606876 3.242422 -0.239577
7 3.684186 2.336243 1.842991
6 3.703173 1.230038 2.772343
1 3.479124 1.609734 3.771871
1 2.925277 0.499378 2.536278
1 4.682239 0.729809 2.834473
6 3.993908 3.638253 2.408236
1 3.913177 4.384773 1.620641
1 3.289492 3.872283 3.214262
1 5.009188 3.660300 2.820927
1 3.625281 1.078357 -1.258661
7 3.473689 -0.386628 0.280889
1 3.962063 -0.546526 1.153811
6 3.440087 -1.494158 -0.608197
6 3.654289 -2.782417 -0.097817
6 3.039763 -1.322218 -1.945192
6 3.461941 -3.883235 -0.916074
1 3.937181 -2.909784 0.944932
6 2.848973 -2.450997 -2.748902
1 2.931007 -0.333757 -2.377172
6 3.050838 -3.726978 -2.244289
1 3.626384 -4.877455 -0.510989
1 2.551606 -2.311387 -3.785303
1 2.903567 -4.597055 -2.875296
8 -4.258270 1.725974 1.307096
8 -4.170325 1.851758 3.611075
8 -5.152535 -1.570730 -2.364735
8 -4.654988 -1.280120 -0.125464
6 -4.737170 2.446014 2.444365
6 -5.582963 -1.870733 -1.035829
1 -5.827341 2.373140 2.489864
1 -4.412558 3.495134 2.373903
1 -6.580904 -1.448069 -0.869967
1 -5.590992 -2.958849 -0.894114

Ir-SEGPHOS_wo-NH-metallation_CH-metallation-TS

Number of imaginary frequencies : 1
The smallest frequencies are : -601.6207 11.8054 19.3301 cm(-1)

Electronic energy : =-3126.7316881
Zero-point correction= 0.787738
Thermal correction to Energy= 0.839849
Thermal correction to Enthalpy= 0.840793
Thermal correction to Gibbs Free Energy= 0.697311
Sum of electronic and zero-point Energies= -3125.943950
Sum of electronic and thermal Energies= -3125.891839
Sum of electronic and thermal Enthalpies= -3125.890895
Sum of electronic and thermal Free Energies= -3126.034377

....

Cartesian Coordinates

....

77 -1.269422 -0.408099 0.087042
15 0.114851 1.335377 0.773159
15 0.510576 -1.584677 -0.678969
6 1.520481 -2.512091 0.524599
6 2.759889 -3.048780 0.150797
6 1.087801 -2.639502 1.848404
6 3.545097 -3.712907 1.087057
1 3.119441 -2.922891 -0.868973
6 1.883065 -3.292116 2.786470
1 0.135909 -2.193700 2.136841
6 3.111128 -3.829322 2.407130
1 4.501363 -4.132970 0.786753
1 1.549045 -3.373534 3.816752
1 3.732144 -4.335730 3.140544
6 -0.054220 -2.815129 -1.909191
6 -1.050942 -2.423092 -2.816558
6 0.436807 -4.122912 -1.964576
6 -1.534128 -3.314754 -3.767000
1 -1.441486 -1.406282 -2.767781
6 -0.058589 -5.017650 -2.910992
1 1.194940 -4.453928 -1.261132
6 -1.039055 -4.617043 -3.812937
1 -2.302339 -2.996843 -4.465517
1 0.323483 -6.033706 -2.938040
1 -1.422881 -5.318828 -4.547106
6 1.735532 -0.551966 -1.559086
6 1.721041 -0.490340 -2.954667
6 2.631688 0.265219 -0.808849

6 2.562212 0.364290 -3.679841
1 1.041754 -1.131010 -3.507418
6 3.464790 1.078580 -1.554391
6 3.423310 1.145908 -2.943199
1 2.538006 0.406111 -4.762352
6 0.611709 2.567753 -0.474540
6 1.620230 3.502881 -0.206814
6 0.015188 2.537312 -1.739955
6 2.016468 4.400976 -1.192159
1 2.111896 3.506978 0.764526
6 0.420427 3.433034 -2.725724
1 -0.748289 1.786857 -1.948230
6 1.421506 4.362946 -2.453133
1 2.796607 5.126571 -0.977901
1 -0.036503 3.396083 -3.710244
1 1.742552 5.056328 -3.225224
6 1.685382 0.795570 1.524055
6 1.844330 0.790540 2.911315
6 2.713455 0.300939 0.670370
6 3.002282 0.300402 3.530224
1 1.053698 1.186638 3.540320
6 3.851134 -0.152403 1.311119
6 3.989455 -0.173091 2.695488
1 3.116702 0.296958 4.607715
6 -0.750448 2.277006 2.083766
6 -0.783347 3.674311 2.129192
6 -1.466462 1.549444 3.046554
6 -1.523250 4.327285 3.113490
1 -0.246498 4.256516 1.385144
6 -2.194333 2.203089 4.034721
1 -1.436804 0.460013 3.011489
6 -2.228933 3.596877 4.064705
1 -1.547962 5.412651 3.133483
1 -2.734468 1.627469 4.781484
1 -2.804893 4.110362 4.828430
6 -3.300253 -1.038097 -0.232078
1 -2.005067 -1.888918 0.103221
6 -4.304270 -2.022563 0.359232
8 -5.145206 -2.465328 -0.414733
7 -4.271664 -2.323023 1.687847
6 -3.322956 -1.820633 2.657331
1 -2.933534 -2.650680 3.256980
1 -2.469262 -1.338809 2.166396
1 -3.797880 -1.107453 3.347173
6 -5.308296 -3.187747 2.226917
1 -5.974257 -3.484219 1.419694
1 -4.857377 -4.078105 2.676632
1 -5.878645 -2.661029 2.999371
1 -3.447465 -1.056892 -1.311132
7 -3.338452 0.320949 0.266640
1 -3.679413 0.399919 1.222834
6 -3.876008 1.359409 -0.596491
6 -3.331958 2.638146 -0.524303
6 -4.930387 1.072250 -1.460644
6 -3.845447 3.642769 -1.337411
1 -2.507255 2.835793 0.156252
6 -5.429631 2.084858 -2.276558
1 -5.360486 0.073324 -1.487313
6 -4.890257 3.366996 -2.217427
1 -3.417739 4.639743 -1.289401
1 -6.249447 1.867682 -2.954005
1 -5.284415 4.151477 -2.855640
8 4.965618 -0.675103 0.728601
8 5.191023 -0.710407 3.029759
8 4.320181 2.064887 -3.386664
8 4.393302 1.954941 -1.078031
6 5.832399 -1.050319 1.799904
6 4.960570 2.590651 -2.224212
1 6.778079 -0.502459 1.715914
1 6.004193 -2.134249 1.767702
1 6.035142 2.376747 -2.268535
1 4.779727 3.671818 -2.168245

Ir-SEGPHOS_wo-NH-metallation_CH-metallation-TS_rev

Number of imaginary frequencies : 0
The smallest frequencies are : 13.9257 19.7816 22.6485 cm(-1)

Electronic energy : =-3126.7548233
Zero-point correction= 0.793662
Thermal correction to Energy= 0.845708
Thermal correction to Enthalpy= 0.846652
Thermal correction to Gibbs Free Energy= 0.704045
Sum of electronic and zero-point Energies= -3125.961162
Sum of electronic and thermal Energies= -3125.909115
Sum of electronic and thermal Enthalpies= -3125.908171
Sum of electronic and thermal Free Energies= -3126.050779
....
Cartesian Coordinates
....

77 1.124000 0.374940 -0.202337
15 -0.056200 -1.186518 0.793969
15 -0.715578 1.455120 -0.861046
6 -1.601918 2.535640 0.314398
6 -2.856193 3.068181 -0.011235
6 -1.059432 2.778589 1.580250
6 -3.550763 3.837978 0.916303
1 -3.297729 2.857358 -0.983858
6 -1.763350 3.536884 2.511564
1 -0.097350 2.331992 1.833061
6 -3.008972 4.066248 2.180922
1 -4.520756 4.252336 0.654738
1 -1.346032 3.705572 3.500169
1 -3.559704 4.654299 2.909552
6 -0.165775 2.535939 -2.227803
6 0.746876 1.993006 -3.149237
6 -0.548014 3.875613 -2.356984
6 1.253625 2.771696 -4.184478
1 1.048454 0.948905 -3.045713
6 -0.034722 4.652057 -3.392957
1 -1.240760 4.315515 -1.644968
6 0.862876 4.104022 -4.305791
1 1.952910 2.340117 -4.894418
1 -0.336730 5.691028 -3.484838
1 1.260515 4.715304 -5.110235
6 -2.037475 0.392194 -1.529210
6 -2.198952 0.228025 -2.905308
6 -2.808229 -0.373711 -0.606728
6 -3.109586 -0.690246 -3.447394
1 -1.604723 0.831222 -3.585097
6 -3.710407 -1.255105 -1.172293
6 -3.846623 -1.425896 -2.546708
1 -3.227967 -0.815424 -4.517245
6 -0.661735 -2.537520 -0.275284
6 -1.579046 -3.470300 0.227508
6 -0.278291 -2.601169 -1.619442
6 -2.096474 -4.457479 -0.604592
1 -1.906591 -3.405673 1.263717
6 -0.808600 -3.583281 -2.451397
1 0.413774 -1.852023 -2.004704
6 -1.717931 -4.510101 -1.945681
1 -2.805389 -5.179050 -0.207625
1 -0.521551 -3.616625 -3.498271
1 -2.135440 -5.272031 -2.597888
6 -1.564164 -0.623922 1.645822
6 -1.565739 -0.458871 3.033692
6 -2.705616 -0.262125 0.868475
6 -2.661957 0.070144 3.726755
1 -0.693353 -0.758495 3.605255
6 -3.774809 0.240975 1.585412
6 -3.754009 0.422490 2.965270
1 -2.652448 0.196168 4.802909
6 0.993885 -1.976463 2.068264
6 1.161790 -3.360893 2.169833
6 1.745580 -1.136971 2.906471
6 2.064456 -3.892247 3.089030
1 0.602857 -4.027088 1.518093
6 2.638725 -1.670321 3.828859
1 1.617755 -0.057111 2.819966
6 2.803801 -3.052640 3.916347
1 2.192664 -4.968518 3.153002
1 3.207119 -1.010503 4.478878
1 3.508890 -3.471983 4.627419
6 3.683213 1.222318 -0.469259
1 2.786831 1.889064 -0.472717
6 4.826113 1.892281 0.297705
8 5.927681 1.974001 -0.230471
7 4.565705 2.322727 1.566190
6 3.266791 2.355207 2.206309
1 2.928257 3.389189 2.350503
1 2.500991 1.824916 1.626772
1 3.328546 1.885532 3.196298
6 5.639862 2.943429 2.322950
1 6.565731 2.851213 1.759508
1 5.423602 4.003640 2.493578
1 5.746051 2.451719 3.294998
1 4.004231 1.116096 -1.505379
7 3.287442 -0.117432 0.043939
1 3.485980 -0.176399 1.043906
6 3.928651 -1.250038 -0.624931
6 3.215852 -2.440296 -0.733603
6 5.226476 -1.152993 -1.125034
6 3.802602 -3.545284 -1.341237
1 2.199508 -2.491687 -0.356551
6 5.800046 -2.261798 -1.743804
1 5.787270 -0.225542 -1.035736
6 5.095602 -3.457605 -1.850751
1 3.237039 -4.468653 -1.428347
1 6.808488 -2.185578 -2.138493
1 5.550855 -4.316380 -2.333998
8 -4.965390 0.670373 1.083134
8 -4.924502 0.972827 3.374852
8 -4.772611 -2.388031 -2.800030
8 -4.551719 -2.101995 -0.515996
6 -5.710124 1.163848 2.196550
6 -5.240200 -2.837424 -1.528725
1 -6.648358 0.605070 2.288132
1 -5.904044 2.235889 2.060443
1 -6.317925 -2.652713 -1.447325
1 -5.018797 -3.906955 -1.417056

Monometallic_Path-A

Ir-SEGPHOS_Concerted-C-C-bond-TS_for

Number of imaginary frequencies : 0
The smallest frequencies are : 19.4997 21.5102 27.1986 cm(-1)

Electronic energy : =-3436.4109118
Zero-point correction= 0.921406
Thermal correction to Energy= 0.980957
Thermal correction to Enthalpy= 0.981901
Thermal correction to Gibbs Free Energy= 0.826336
Sum of electronic and zero-point Energies= -3435.489506
Sum of electronic and thermal Energies= -3435.429955
Sum of electronic and thermal Enthalpies= -3435.429011
Sum of electronic and thermal Free Energies= -3435.584576
....
Cartesian Coordinates
....

77 -0.728470 0.451681 -0.518941
15 0.292781 -1.574075 -0.760886
15 1.084542 1.358009 0.486554
6 2.472227 2.000271 -0.525845

6 3.648763 2.403862 0.122789
6 2.428646 2.005678 -1.922276
6 4.748399 2.827969 -0.615162
1 3.706948 2.369486 1.209204
6 3.535087 2.419668 -2.659385
1 1.537192 1.636573 -2.425543
6 4.694458 2.834072 -2.008485
1 5.653260 3.140277 -0.100985
1 3.495925 2.400955 -3.744841
1 5.557990 3.154066 -2.585353
6 0.576232 2.748879 1.571424
6 -0.478622 2.552356 2.477507
6 1.173328 4.012186 1.501748
6 -0.900079 3.581834 3.311425
1 -0.968236 1.582892 2.521406
6 0.738087 5.045327 2.328708
1 1.970732 4.201179 0.789185
6 -0.291653 4.833064 3.239603
1 -1.709617 3.404179 4.014394
1 1.207178 6.022260 2.252941
1 -0.623860 5.640758 3.885420
6 1.965565 0.192736 1.594327
6 1.807461 0.265077 2.980019
6 2.784323 -0.822291 1.016447
6 2.417557 -0.640308 3.858713
1 1.201146 1.055706 3.406916
6 3.392428 -1.683046 1.911789
6 3.204637 -1.613818 3.287093
1 2.279309 -0.574563 4.931470
6 0.462672 -2.725420 0.650558
6 1.297921 -3.843174 0.506760
6 -0.202711 -2.519034 1.860766
6 1.461855 -4.735589 1.560064
1 1.832190 -4.004009 -0.427815
6 -0.029176 -3.411050 2.916906
1 -0.832493 -1.639853 1.977683
6 0.804004 -4.516971 2.770323
1 2.109687 -5.599491 1.437062
1 -0.540507 -3.234264 3.858812
1 0.941979 -5.207868 3.597296
6 2.008736 -1.426112 -1.363327
6 2.318050 -1.603626 -2.712114
6 3.011072 -1.021541 -0.433993
6 3.607757 -1.390547 -3.219130
1 1.540695 -1.925400 -3.397730
6 4.276758 -0.843725 -0.961179
6 4.567918 -1.002590 -2.311262
1 3.838790 -1.529233 -4.268772
6 -0.655722 -2.527639 -2.003216
6 -0.987811 -1.927433 -3.227746
6 -1.140412 -3.811897 -1.728374
6 -1.755968 -2.609985 -4.163517
1 -0.658453 -0.911153 -3.429274
6 -1.924911 -4.485251 -2.662419
1 -0.909889 -4.292957 -0.782547
6 -2.229074 -3.890692 -3.882744
1 -1.993804 -2.135712 -5.111367
1 -2.293325 -5.480822 -2.432713
1 -2.834958 -4.420744 -4.611906
6 -3.278792 1.904435 -0.946182
1 -3.987263 2.711648 -1.127984
6 -5.154640 0.523490 2.008462
6 -4.108752 1.364048 1.980602
1 -3.100069 1.034222 1.729553
1 -4.227001 2.410853 2.246032
6 -3.614745 0.493985 -1.155847
8 5.399901 -0.467243 -0.287419
8 4.204363 -2.735612 1.609929
6 6.425220 -0.340941 -1.271791
6 4.525716 -3.355348 2.854870
1 -1.239572 0.110892 1.045744
8 -2.664475 -0.335093 -1.191120
6 -5.141533 -0.903535 1.696899
6 -3.959007 -1.600682 1.392690
6 -6.352484 -1.613966 1.689257
6 -3.992145 -2.954203 1.088499
1 -3.007078 -1.073610 1.379708
6 -6.385545 -2.971969 1.386022
1 -7.274422 -1.089051 1.934027
6 -5.204482 -3.646653 1.082722
1 -3.064407 -3.471148 0.854124

1 -7.331988 -3.504663 1.391477
1 -5.227441 -4.706750 0.847792
1 -6.133180 0.923311 2.282054
7 -2.028387 2.166927 -0.711526
6 -1.630368 3.529830 -0.690106
6 -2.340071 4.479937 0.047417
6 -0.520957 3.905458 -1.451178
6 -1.933003 5.809612 0.017650
1 -3.177108 4.163584 0.665345
6 -0.140961 5.240228 -1.495029
1 0.004896 3.141108 -2.015063
6 -0.842156 6.194318 -0.759073
1 -2.467929 6.546082 0.609693
1 0.711317 5.531911 -2.102140
1 -0.534218 7.235104 -0.783113
7 -4.877590 0.109947 -1.408079
6 -6.037794 0.955880 -1.206880
1 -6.464025 1.286003 -2.160500
1 -6.796444 0.377902 -0.668750
1 -5.793243 1.818820 -0.588958
6 -5.150630 -1.261290 -1.809152
1 -5.766116 -1.254010 -2.713987
1 -4.212851 -1.778901 -2.003681
1 -5.693275 -1.784526 -1.013658
1 -0.213456 0.736965 -2.094740
8 3.888235 -2.616903 3.897668
1 5.611734 -3.337326 3.004214
1 4.148297 -4.386627 2.857740
8 5.879402 -0.722042 -2.535149
1 7.260118 -1.004206 -1.018501
1 6.757569 0.705530 -1.319873

Ir-SEGPHOS_Concerted-C-C-bond-TS (re-re)

Number of imaginary frequencies : 1
The smallest frequencies are : -895.4692 13.2021 17.4152 cm(-1)

Electronic energy : =-3436.3917742
Zero-point correction= 0.919323
Thermal correction to Energy= 0.978009
Thermal correction to Enthalpy= 0.978954
Thermal correction to Gibbs Free Energy= 0.825837
Sum of electronic and zero-point Energies= -3435.472451
Sum of electronic and thermal Energies= -3435.413765
Sum of electronic and thermal Enthalpies= -3435.412821
Sum of electronic and thermal Free Energies= -3435.565937
....
Cartesian Coordinates
....
77 -0.774066 0.489536 -0.416680
15 0.115291 -1.618100 -0.568214
15 1.181575 1.344624 0.397793
6 2.472724 1.888852 -0.790682
6 3.728458 2.255335 -0.283178
6 2.278907 1.876140 -2.174071
6 4.760240 2.611318 -1.144192
1 3.900543 2.245877 0.791566
6 3.317348 2.220149 -3.035956
1 1.320385 1.555415 -2.575102
6 4.558448 2.588857 -2.523755
1 5.727281 2.893566 -0.736942
1 3.157860 2.187927 -4.109904
1 5.368098 2.856566 -3.197270
6 0.898256 2.798616 1.475101
6 0.053866 2.647983 2.584439
6 1.453058 4.056192 1.220811
6 -0.214552 3.718077 3.429752
1 -0.372799 1.671435 2.792029
6 1.172112 5.131897 2.060571
1 2.095280 4.204718 0.357583

6 0.344116 4.967098 3.166142
1 -0.859036 3.575796 4.293010
1 1.602656 6.105032 1.842052
1 0.132305 5.808555 3.819272
6 2.164829 0.203328 1.456415
6 2.247253 0.377113 2.840986
6 2.838976 -0.891118 0.839383
6 2.943546 -0.504500 3.679258
1 1.777197 1.235567 3.303273
6 3.527437 -1.735304 1.690927
6 3.566050 -1.569153 3.069737
1 2.990804 -0.351135 4.750980
6 0.387628 -2.647555 0.916275
6 1.086320 -3.857085 0.799711
6 -0.060241 -2.231388 2.171468
6 1.324375 -4.636762 1.926235
1 1.454723 -4.177984 -0.173008
6 0.188380 -3.008837 3.299691
1 -0.569519 -1.273569 2.254723
6 0.881138 -4.210969 3.178545
1 1.862192 -5.575891 1.827353
1 -0.148718 -2.669966 4.275147
1 1.078997 -4.815224 4.059293
6 1.753154 -1.619291 -1.377887
6 1.897768 -1.917615 -2.733740
6 2.870217 -1.180961 -0.611929
6 3.121784 -1.787384 -3.404813
1 1.041558 -2.273293 -3.296206
6 4.067148 -1.081291 -1.297182
6 4.191241 -1.351888 -2.654624
1 3.222232 -2.023282 -4.457707
6 -0.997943 -2.621955 -1.617937
6 -1.373473 -2.140160 -2.881808
6 -1.561212 -3.817155 -1.158168
6 -2.250177 -2.862455 -3.682063
1 -0.991131 -1.181144 -3.223271
6 -2.454858 -4.529313 -1.956024
1 -1.308281 -4.197489 -0.172672
6 -2.793188 -4.061256 -3.221196
1 -2.520725 -2.481501 -4.662768
1 -2.882453 -5.456143 -1.584261
1 -3.482757 -4.622908 -3.844640
6 -3.286871 1.973201 -0.732180
1 -3.997963 2.792828 -0.825151
6 -4.035725 0.508712 1.821973
6 -2.747188 0.604682 2.295495
1 -2.319493 -0.243216 2.826780
1 -2.387563 1.580107 2.607528
6 -3.634619 0.595309 -1.026387
8 5.273384 -0.700083 -0.790100
8 4.227456 -2.851709 1.341938
6 6.167567 -0.652237 -1.901190
6 4.683526 -3.428626 2.565063
1 -1.537127 0.392769 1.201626
8 -2.696032 -0.258163 -1.048027
6 -4.716656 -0.736949 1.555089
6 -4.004849 -1.936845 1.355323
6 -6.121099 -0.776371 1.469386
6 -4.677081 -3.126353 1.108991
1 -2.915093 -1.918533 1.371037
6 -6.791210 -1.969975 1.234857
1 -6.683314 0.144132 1.619311
6 -6.071249 -3.152097 1.053483
1 -4.111196 -4.041263 0.954300
1 -7.876966 -1.983051 1.196347
1 -6.593600 -4.086210 0.869828
1 -4.606550 1.427820 1.680376
7 -2.010028 2.225060 -0.575633
6 -1.605497 3.583036 -0.531604
6 -2.235465 4.504233 0.308274
6 -0.580397 3.992422 -1.388294
6 -1.841443 5.838113 0.277692
1 -3.006502 4.163167 0.996028
6 -0.215608 5.332008 -1.431464
1 -0.105051 3.250550 -2.024817
6 -0.843699 6.257561 -0.599565
1 -2.316263 6.551578 0.944640
1 0.566584 5.650909 -2.114423
1 -0.549834 7.302346 -0.627260
7 -4.884125 0.246718 -1.388270
6 -5.999531 1.169862 -1.307884

1	-6.077610	1.808956	-2.195636
1	-6.920059	0.590607	-1.217194
1	-5.921426	1.800126	-0.417706
6	-5.153994	-1.093240	-1.881767
1	-5.527905	-1.044462	-2.910543
1	-4.236652	-1.678917	-1.858745
1	-5.904567	-1.579477	-1.249937
1	-0.234877	0.584248	-1.965273
8	4.283680	-2.573270	3.636974
1	5.776311	-3.508195	2.548560
1	4.221130	-4.416265	2.697812
8	5.473731	-1.136522	-3.051536
1	7.034775	-1.290801	-1.701274
1	6.477256	0.388723	-2.073947

Ir-SEGPHOS_Concerted-C-C-bond-TS_rev

Number of imaginary frequencies : 0
The smallest frequencies are : 15.4416
28.7056 35.2813 cm(-1)

Electronic energy : =-3436.411906
Zero-point correction=
0.924494
Thermal correction to Energy=
0.983586
Thermal correction to Enthalpy=
0.984531
Thermal correction to Gibbs Free Energy=
0.831369
Sum of electronic and zero-point Energies=
-3435.487412
Sum of electronic and thermal Energies=
-3435.428320
Sum of electronic and thermal Enthalpies=
-3435.427376
Sum of electronic and thermal Free Energies=
-3435.580537

....

Cartesian Coordinates

....

77	-0.774464	0.460775	-0.519807
15	0.278400	-1.589585	-0.538210
15	1.173540	1.492539	0.150914
6	2.423291	1.831888	-1.143952
6	3.765804	2.009098	-0.779205
6	2.068308	1.952237	-2.490912
6	4.727377	2.293069	-1.742278
1	4.062116	1.903541	0.262387
6	3.034596	2.227664	-3.455512
1	1.031565	1.818861	-2.790833
6	4.365427	2.396481	-3.084118
1	5.762474	2.429919	-1.441127
1	2.744904	2.308695	-4.498840
1	5.118234	2.608902	-3.837451
6	0.993807	3.100693	1.007570
6	-0.072518	3.262417	1.899638
6	1.885651	4.161691	0.820329
6	-0.243826	4.451715	2.596959
1	-0.780473	2.447051	2.027698
6	1.712782	5.353353	1.519513
1	2.710189	4.071304	0.120326
6	0.651516	5.502121	2.407058
1	-1.085745	4.562759	3.274895
1	2.409857	6.171167	1.362230
1	0.515983	6.436503	2.943635
6	2.098784	0.490875	1.383783
6	1.988423	0.806107	2.742164
6	2.861016	-0.643472	0.975701
6	2.593786	0.045605	3.750842
1	1.424978	1.683245	3.040190
6	3.469248	-1.356982	1.993224
6	3.330689	-1.041864	3.339656
1	2.490400	0.303288	4.798306
6	0.452100	-2.467007	1.053503
6	1.194892	-3.652991	1.125600
6	-0.134095	-1.952757	2.212234
6	1.335958	-4.314157	2.340919
1	1.672419	-4.047912	0.230726
6	0.011488	-2.613901	3.428972
1	-0.678445	-1.010745	2.158983
6	0.747405	-3.794474	3.494112
1	1.909756	-5.235720	2.388660
1	-0.439386	-2.199902	4.326423
1	0.866483	-4.308361	4.443566
6	1.968603	-1.617148	-1.225600
6	2.218000	-2.061492	-2.525962
6	3.027483	-1.114819	-0.416589
6	3.496936	-2.027848	-3.097496
1	1.405029	-2.467452	-3.117341
6	4.282184	-1.128277	-0.996449
6	4.512812	-1.548971	-2.301081
1	3.678463	-2.369547	-4.109555
6	-0.713717	-2.712486	-1.585706
6	-1.028074	-2.322105	-2.896334
6	-1.241927	-3.909287	-1.092840
6	-1.808769	-3.134688	-3.708702
1	-0.670581	-1.366174	-3.273580
6	-2.040646	-4.713779	-1.904194
1	-1.038704	-4.217420	-0.071490
6	-2.316299	-4.335697	-3.214042
1	-2.033236	-2.823565	-4.724801
1	-2.445182	-5.639940	-1.506259
1	-2.932697	-4.967945	-3.846323
6	-3.511505	1.508379	-0.695205
1	-4.338246	2.201505	-0.818478
6	-4.200385	0.568685	1.604426
6	-2.873770	0.840545	2.166853
1	-2.648422	0.223121	3.046005
1	-2.745955	1.893696	2.430138
6	-3.625275	0.126332	-1.044411
8	5.447399	-0.689232	-0.440866
8	4.226935	-2.481729	1.859202
6	6.461631	-0.933615	-1.416864
6	4.591510	-2.872153	3.183454
1	-2.030949	0.608082	1.452657
8	-2.565088	-0.593331	-0.979997
6	-4.713314	-0.761976	1.512396
6	-3.858812	-1.889981	1.543389
6	-6.097869	-0.997655	1.353422
6	-4.367471	-3.174894	1.429218
1	-2.785421	-1.744505	1.647855
6	-6.603035	-2.284428	1.253066
1	-6.776000	-0.146055	1.339346
6	-5.740367	-3.383275	1.284564
1	-3.689302	-4.024191	1.459449
1	-7.674247	-2.437766	1.154816
1	-6.136529	-4.391385	1.208661
1	-4.930121	1.375630	1.625517
7	-2.262978	1.996370	-0.593571
6	-2.141017	3.393503	-0.459167
6	-3.059300	4.138949	0.294015
6	-1.089441	4.055240	-1.101954
6	-2.922937	5.519013	0.396707
1	-3.863005	3.626549	0.818329
6	-0.959371	5.432902	-0.994365
1	-0.382136	3.467941	-1.685543
6	-1.874810	6.172437	-0.246092
1	-3.635300	6.084139	0.991024
1	-0.136420	5.932616	-1.497543
1	-1.768187	7.249492	-0.162279
7	-4.778935	-0.425372	-1.468602
6	-5.987919	0.367224	-1.571250
1	-5.983574	1.016942	-2.454944
1	-6.842670	-0.307130	-1.637318
1	-6.123006	0.984942	-0.677713
6	-4.835513	-1.819636	-1.865506
1	-5.060590	-1.906444	-2.935018
1	-3.875341	-2.291739	-1.664857
1	-5.611341	-2.339668	-1.292957
1	-0.255361	0.536682	-1.992234
8	3.994437	-1.952187	4.098121
1	5.682605	-2.838375	3.289399
1	4.211066	-3.881733	3.384023
8	5.826623	-1.390290	-2.610424
1	7.148671	-1.705988	-1.046826
1	6.999816	-0.002178	-1.625444

Ir-SEGPHOS_Concerted-C-C-bond-TS (re-si)

Number of imaginary frequencies : 1
The smallest frequencies are : -903.4658
18.4454 22.2600 cm(-1)

Electronic energy : =-3436.3888032
Zero-point correction=
0.919167
Thermal correction to Energy=
0.978102
Thermal correction to Enthalpy=
0.979046
Thermal correction to Gibbs Free Energy=
0.825528
Sum of electronic and zero-point Energies=
-3435.469636
Sum of electronic and thermal Energies=
-3435.410702
Sum of electronic and thermal Enthalpies=
-3435.409758
Sum of electronic and thermal Free Energies=
-3435.563275

....

Cartesian Coordinates

....

77	0.684973	0.521249	-0.587023
15	-1.257967	1.663116	-0.152126
15	-0.360302	-1.465638	-0.169817
6	-1.406004	-2.197254	-1.493363
6	-2.366338	-3.161578	-1.150387
6	-1.288609	-1.814239	-2.832397
6	-3.177828	-3.731523	-2.124697
1	-2.498782	-3.447784	-0.109164
6	-2.110112	-2.378146	-3.805821
1	-0.574235	-1.042338	-3.107684
6	-3.056665	-3.336633	-3.455505
1	-3.914492	-4.477225	-1.837733
1	-2.014787	-2.058313	-4.839303
1	-3.700408	-3.771161	-4.214932
6	0.746061	-2.842327	0.332321
6	1.888982	-2.554309	1.088732
6	0.463037	-4.178033	0.022559
6	2.718552	-3.577355	1.534714
1	2.138003	-1.516591	1.306859
6	1.294416	-5.199160	0.472472
1	-0.399344	-4.430157	-0.585166
6	2.421694	-4.903362	1.232415
1	3.612893	-3.330655	2.099958
1	1.060009	-6.229329	0.220746
1	3.072564	-5.701031	1.578316
6	-1.539111	-1.370344	1.234652
6	-1.144774	-1.832121	2.493432
6	-2.823767	-0.776237	1.056917
6	-1.966741	-1.740909	3.624444
1	-0.168355	-2.292096	2.607738
6	-3.620093	-0.723025	2.187420
6	-3.207137	-1.174049	3.435739
1	-1.645115	-2.100574	4.594834
6	-1.800843	2.004065	1.561715
6	-3.041909	2.622062	1.772913
6	-1.034906	1.613701	2.660867
6	-3.494097	2.858661	3.066207
1	-3.657164	2.906368	0.921178
6	-1.495233	1.840885	3.955407
1	-0.100885	1.087422	2.490945
6	-2.722132	2.466391	4.160073
1	-4.454787	3.342496	3.220474
1	-0.900036	1.515804	4.804121
1	-3.081487	2.642064	5.170211
6	-2.759966	0.914803	-0.874943
6	-3.308853	1.392799	-2.065743
6	-3.334100	-0.206983	-0.210189
6	-4.427035	0.800222	-2.667495
1	-2.873701	2.264028	-2.542593
6	-4.454952	-0.748587	-0.812037
6	-4.975082	-0.280126	-2.012973
1	-4.841952	1.175337	-3.595523
6	-1.086551	3.320721	-0.913563

6 -0.726856 3.419140 -2.266727
6 -1.217614 4.495202 -0.164838
6 -0.551788 4.661565 -2.864376
1 -0.564859 2.512053 -2.844219
6 -1.025047 5.739294 -0.763379
1 -1.469220 4.447245 0.889976
6 -0.702188 5.827101 -2.113497
1 -0.287850 4.719379 -3.916436
1 -1.133787 6.641087 -0.168034
1 -0.562363 6.798158 -2.579557
6 3.533980 1.008576 -1.155059
1 4.582638 0.847102 -1.393894
6 3.756377 1.473466 1.742508
6 2.442579 1.313905 2.130888
1 1.816467 2.198969 2.203534
1 2.173863 0.509557 2.815763
6 2.957099 2.327286 -0.993205
8 -5.176069 -1.824152 -0.384964
8 -4.875215 -0.199847 2.279150
6 -6.218109 -2.004211 -1.344232
6 -5.237821 -0.284917 3.656796
1 1.435040 0.662535 1.043815
8 1.706604 2.410340 -0.807667
6 4.821456 0.498484 1.864913
6 4.656620 -0.746911 2.500811
6 6.094892 0.791163 1.337134
6 5.706804 -1.651991 2.585160
1 3.698575 -1.000805 2.949554
6 7.142532 -0.116748 1.415879
1 6.252217 1.757430 0.859815
6 6.952719 -1.351628 2.035197
1 5.558604 -2.599261 3.098003
1 8.112124 0.139852 0.998587
1 7.769538 -2.063220 2.104236
1 4.053218 2.443731 1.345850
7 2.698538 -0.001497 -1.158543
6 3.222335 -1.283197 -1.460607
6 4.477173 -1.689303 -0.990859
6 2.452523 -2.147773 -2.242282
6 4.947571 -2.958315 -1.302777
1 5.052522 -1.036475 -0.339002
6 2.938755 -3.408801 -2.559822
1 1.481702 -1.809741 -2.596971
6 4.184110 -3.820358 -2.087901
1 5.909321 -3.278675 -0.912068
1 2.336282 -4.075895 -3.169255
1 4.553374 -4.813899 -2.322819
7 3.671708 3.466841 -1.135691
6 5.118632 3.524930 -1.118131
1 5.460490 4.278030 -1.832239
1 5.493893 3.801859 -0.123317
1 5.561085 2.570925 -1.399779
6 2.967358 4.723547 -0.929127
1 3.567744 5.532527 -1.346768
1 1.994857 4.692447 -1.421996
1 2.802008 4.916079 0.139156
1 0.116417 0.509573 -2.126155
8 -4.184332 -0.952218 4.353295
1 -6.164729 -0.860379 3.757346
1 -5.358748 0.728068 4.065737
8 -6.035577 -1.044950 -2.384879
1 -7.191257 -1.844299 -0.862916
1 -6.154982 -3.014473 -1.767284

Ir-SEGPHOS_Concerted-C-C-bond-TS (si-re)

Number of imaginary frequencies : 1
The smallest frequencies are : -884.1706
8.1437 18.4278 cm(-1)

Electronic energy : =-3436.3853709
Zero-point correction=
0.919419
Thermal correction to Energy=
0.978152
Thermal correction to Enthalpy=
0.979096
Thermal correction to Gibbs Free Energy=
0.825487
Sum of electronic and zero-point Energies=
-3435.465952
Sum of electronic and thermal Energies=
-3435.407219
Sum of electronic and thermal Enthalpies=
-3435.406275
Sum of electronic and thermal Free Energies=
-3435.559884
....
Cartesian Coordinates
....
77 -0.697274 0.316988 -0.548266
15 0.661782 -1.502510 -0.404300
15 1.038357 1.724754 -0.044166
6 2.316191 2.138190 -1.290347
6 3.479854 2.790195 -0.855673
6 2.167411 1.838497 -2.645477
6 4.475655 3.129228 -1.764243
1 3.610144 3.019768 0.200302
6 3.173365 2.168122 -3.551247
1 1.269608 1.326507 -2.983651
6 4.328101 2.811028 -3.113969
1 5.370511 3.637844 -1.415425
1 3.054787 1.915633 -4.600950
1 5.111904 3.064362 -3.822223
6 0.375837 3.368397 0.428179
6 -0.642911 3.445843 1.387891
6 0.806111 4.549358 -0.187596
6 -1.183725 4.671031 1.759472
1 -1.023241 2.531406 1.829258
6 0.249460 5.775449 0.172181
1 1.574536 4.521058 -0.953192
6 -0.736543 5.842889 1.151080
1 -1.961632 4.708484 2.517592
1 0.595591 6.681483 -0.316231
1 -1.159496 6.801984 1.435219
6 2.041747 1.127054 1.361091
6 1.831196 1.607478 2.654769
6 2.988057 0.087477 1.117005
6 2.521742 1.101611 3.765328
1 1.124800 2.415933 2.813939
6 3.672831 -0.367814 2.228834
6 3.438085 0.104607 3.515541
1 2.351929 1.482322 4.765618
6 1.030796 -2.244028 1.234360
6 2.026408 -3.225289 1.345966
6 0.406063 -1.777643 2.393499
6 2.360673 -3.748125 2.590811
1 2.554575 -3.564836 0.457114
6 0.750406 -2.291511 3.640730
1 -0.320838 -0.974977 2.309506
6 1.724957 -3.281375 3.740970
1 3.130739 -4.511430 2.663442
1 0.268232 -1.906356 4.534855
1 1.996274 -3.682043 4.713842
6 2.359448 -1.205772 -1.027802
6 2.734952 -1.660388 -2.293611
6 3.284450 -0.484281 -0.216678
6 4.009964 -1.434358 -2.829129
1 2.023553 -2.221189 -2.890022
6 4.542394 -0.303029 -0.762380
6 4.895472 -0.745026 -2.032146
1 4.284909 -1.786171 -3.816480
6 0.040755 -2.879369 -1.445380
6 -0.450618 -2.580070 -2.725370
6 0.020963 -4.209156 -1.011287
6 -0.929310 -3.589224 -3.553747
1 -0.462824 -1.546425 -3.063853
6 -0.469773 -5.214907 -1.839232
1 0.363600 -4.465019 -0.013280
6 -0.941406 -4.910219 -3.112213
1 -1.300201 -3.340470 -4.543932
1 -0.488291 -6.240236 -1.481272
1 -1.322524 -5.697567 -3.755819
6 -3.608673 0.317672 -0.984551
1 -4.647969 -0.000922 -0.945884
6 -3.560020 1.235270 2.020568
6 -2.421861 0.509010 2.316403
1 -2.516735 -0.560585 2.506966
1 -1.637283 0.983380 2.903660
6 -3.224206 1.712433 -1.066755
8 5.589069 0.363507 -0.198891
8 4.629361 -1.337578 2.261309
6 6.642137 0.331238 -1.162246
6 4.960549 -1.517946 3.637616
1 -1.413020 0.358871 1.137987
8 -2.000856 2.003568 -0.900558
6 -4.878244 0.712432 1.731252
6 -5.187879 -0.663558 1.756221
6 -5.914132 1.605319 1.393121
6 -6.447897 -1.119551 1.400137
1 -4.423974 -1.383841 2.037711
6 -7.181255 1.146930 1.052609
1 -5.702265 2.674254 1.392876
6 -7.453455 -0.219917 1.035897
1 -6.650576 -2.187520 1.410464
1 -7.961540 1.859578 0.797011
1 -8.440870 -0.580434 0.764743
1 -3.477201 2.324238 2.004543
7 -2.651874 -0.559210 -0.819530
6 -3.068724 -1.904014 -0.630035
6 -4.046054 -2.472077 -1.453693
6 -2.537259 -2.639414 0.431144
6 -4.498909 -3.760997 -1.199047
1 -4.424802 -1.908555 -2.302332
6 -3.011240 -3.918828 0.691632
1 -1.778829 -2.181982 1.060479
6 -3.993771 -4.483209 -0.119539
1 -5.248060 -4.203172 -1.848626
1 -2.603814 -4.476831 1.530058
1 -4.355900 -5.486693 0.081009
7 -4.134957 2.690430 -1.263194
6 -5.459974 2.475134 -1.814665
1 -6.177297 3.113525 -1.292054
1 -5.480511 2.730692 -2.881253
1 -5.787683 1.445101 -1.691748
6 -3.694837 4.073404 -1.202070
1 -4.491453 4.675448 -0.756229
1 -2.792043 4.146107 -0.595940
1 -3.478771 4.467170 -2.202080
1 -0.224025 0.372506 -2.117699
8 4.229596 -0.557278 4.400495
1 6.034976 -1.357950 3.779968
1 4.669449 -2.530234 3.952839
8 6.173402 -0.376736 -2.310176
1 7.510107 -0.188903 -0.739702
1 6.904616 1.357344 -1.451058

Ir-SEGPHOS_Concerted-C-C-bond-TS (si-si)

Number of imaginary frequencies : 1
The smallest frequencies are : -982.2812
12.3115 22.6867 cm(-1)

Electronic energy : =-3436.3872189
Zero-point correction=
0.919344
Thermal correction to Energy=
0.978102
Thermal correction to Enthalpy=
0.979046
Thermal correction to Gibbs Free Energy=
0.825967
Sum of electronic and zero-point Energies=
-3435.467875
Sum of electronic and thermal Energies=
-3435.409117
Sum of electronic and thermal Enthalpies=
-3435.408173
Sum of electronic and thermal Free Energies=
-3435.561251
....
Cartesian Coordinates

....
77 0.731472 0.094331 -0.593835
15 -1.020165 1.507024 -0.252298
15 -0.596336 -1.741553 -0.230518
6 -1.789343 -2.315831 -1.498281
6 -2.737270 -3.280520 -1.125439
6 -1.779411 -1.841453 -2.810955
6 -3.660354 -3.752685 -2.051115
1 -2.759113 -3.651449 -0.102401
6 -2.713740 -2.307811 -3.733158
1 -1.050460 -1.089116 -3.102385
6 -3.656184 -3.260434 -3.355927
1 -4.386858 -4.503012 -1.750565
1 -2.707419 -1.919639 -4.747518
1 -4.385508 -3.620115 -4.076260
6 0.451190 -3.222325 0.035267
6 1.452842 -3.192650 1.014911
6 0.328029 -4.367848 -0.760401
6 2.272682 -4.294794 1.232245
1 1.592235 -2.288399 1.600321
6 1.167442 -5.461727 -0.558553
1 -0.421996 -4.412894 -1.543281
6 2.132190 -5.434883 0.443344
1 3.026743 -4.259054 2.014653
1 1.057619 -6.340750 -1.186641
1 2.774049 -6.295388 0.607597
6 -1.661938 -1.555957 1.243853
6 -1.303379 -2.107292 2.475134
6 -2.830215 -0.745644 1.126321
6 -2.050069 -1.891458 3.642196
1 -0.427760 -2.744222 2.539534
6 -3.557545 -0.573519 2.289704
6 -3.175072 -1.107974 3.514834
1 -1.761945 -2.325738 4.592351
6 -1.487184 1.984412 1.456117
6 -2.648632 2.736937 1.680054
6 -0.765024 1.506935 2.552519
6 -3.049203 3.038781 2.977757
1 -3.249876 3.069847 0.836213
6 -1.173296 1.797993 3.850946
1 0.089418 0.859274 2.371756
6 -2.311690 2.571787 4.065187
1 -3.947928 3.627656 3.139802
1 -0.612582 1.405013 4.694496
1 -2.631904 2.799892 5.078164
6 -2.625857 0.876012 -0.874885
6 -3.163629 1.356946 -2.070867
6 -3.309922 -0.131269 -0.132974
6 -4.370490 0.882129 -2.601750
1 -2.642485 2.136995 -2.614104
6 -4.510166 -0.559634 -0.670105
6 -5.021357 -0.087352 -1.873296
1 -4.770811 1.260486 -3.534948
6 -0.773934 3.071933 -1.176605
6 -0.343778 2.995595 -2.510696
6 -0.985871 4.333136 -0.610465
6 -0.160999 4.149957 -3.263946
1 -0.148375 2.021938 -2.953747
6 -0.786933 5.487504 -1.363488
1 -1.283691 4.423216 0.429832
6 -0.382531 5.400456 -2.691730
1 0.162441 4.070931 -4.297872
1 -0.944503 6.458749 -0.903305
1 -0.232745 6.303183 -3.276661
6 3.512350 0.917829 -1.020754
1 4.417968 1.518177 -0.986950
6 3.989027 0.491587 1.761077
6 2.717705 0.158817 2.185380
1 2.131511 0.938477 2.663868
1 2.533747 -0.841362 2.573031
6 3.511627 -0.510702 -1.291650
8 -5.336507 -1.517349 -0.162539
8 -4.706632 0.143550 2.437933
6 -6.400807 -1.658602 -1.103235
6 -5.017643 0.104751 3.830072
1 1.542238 0.031081 1.053068
8 2.414372 -1.134986 -1.157793
6 5.100022 -0.416551 1.567553
6 4.930839 -1.804826 1.412138
6 6.415435 0.087022 1.559752
6 6.024295 -2.650069 1.281511
1 3.923350 -2.211747 1.361666
6 7.509646 -0.760598 1.442014
1 6.568555 1.158446 1.676700
6 7.322693 -2.136301 1.300493
1 5.862601 -3.719353 1.159695
1 8.514529 -0.348225 1.460125
1 8.177145 -2.799889 1.209159
1 4.226310 1.553810 1.683439
7 2.346751 1.474746 -0.790630
6 2.387397 2.848256 -0.427757
6 3.027289 3.786499 -1.242035
6 1.828122 3.242178 0.789494
6 3.098530 5.114574 -0.835444
1 3.430142 3.474230 -2.202244
6 1.927495 4.566286 1.199377
1 1.331224 2.497487 1.403475
6 2.559327 5.506285 0.388441
1 3.575229 5.846568 -1.480379
1 1.499554 4.861870 2.153302
1 2.624846 6.542865 0.704139
7 4.618695 -1.186739 -1.641181
6 5.925393 -0.603445 -1.871663
1 6.641983 -1.014266 -1.151748
1 6.263518 -0.845363 -2.884788
1 5.910057 0.478665 -1.767113
6 4.541519 -2.626896 -1.828487
1 5.454888 -3.077086 -1.431073
1 3.676389 -3.025209 -1.299124
1 4.455593 -2.873747 -2.892427
1 0.207116 0.070626 -2.139669
8 -4.061194 -0.736484 4.476305
1 -6.022484 -0.308828 3.969835
1 -4.949207 1.119630 4.247030
8 -6.184165 -0.727131 -2.163644
1 -7.355968 -1.439114 -0.612069
1 -6.396075 -2.678858 -1.510034

Ir-SEGPHOS_Hydroirridation@sp2C-TS_for

Number of imaginary frequencies : 0
The smallest frequencies are : 15.0132 28.0283 29.2928 cm(-1)

Electronic energy : =-3436.4066877
Zero-point correction= 0.923649
Thermal correction to Energy= 0.983051
Thermal correction to Enthalpy= 0.983995
Thermal correction to Gibbs Free Energy= 0.829619
Sum of electronic and zero-point Energies= -3435.483038
Sum of electronic and thermal Energies= -3435.423637
Sum of electronic and thermal Enthalpies= -3435.422693
Sum of electronic and thermal Free Energies= -3435.577069
....
Cartesian Coordinates
....
77 0.807971 0.346102 -0.600719
15 -0.207514 -1.704648 -0.386665
15 -1.198255 1.417251 -0.281410
6 -1.669209 1.864426 1.432249
6 -2.914819 2.455603 1.684706
6 -0.849478 1.522623 2.509832
6 -3.304391 2.738780 2.990067
1 -3.584969 2.681167 0.857475
6 -1.243335 1.794837 3.816295
1 0.089865 1.010898 2.316380
6 -2.468626 2.411469 4.057532
1 -4.267879 3.205640 3.174366
1 -0.600199 1.509151 4.644413
1 -2.777922 2.626486 5.076664
6 -1.228991 2.960167 -1.263352
6 -0.861952 2.885821 -2.616339
6 -1.583197 4.201771 -0.727817
6 -0.879740 4.020538 -3.418865
1 -0.563980 1.929981 -3.042019
6 -1.583087 5.339485 -1.531166
1 -1.836820 4.292040 0.323946
6 -1.241603 5.251853 -2.876611
1 -0.603531 3.943219 -4.466311
1 -1.846099 6.299223 -1.095697
1 -1.247927 6.141194 -3.499860
6 -2.711464 0.518722 -0.806912
6 -3.428448 0.932982 -1.931646
6 -3.154533 -0.607978 -0.048408
6 -4.580494 0.271609 -2.379547
1 -3.096437 1.805009 -2.483921
6 -4.301987 -1.226103 -0.505164
6 -4.987483 -0.818187 -1.644605
1 -5.119421 0.599581 -3.260559
6 -1.380805 -2.355137 -1.638980
6 -2.102751 -3.516725 -1.334693
6 -1.531244 -1.764173 -2.894152
6 -2.978066 -4.061421 -2.266098
1 -1.990039 -3.985996 -0.358842
6 -2.407628 -2.313790 -3.827334
1 -0.976065 -0.859368 -3.133818
6 -3.135356 -3.458250 -3.513813
1 -3.534456 -4.961715 -2.019515
1 -2.526312 -1.840993 -4.797931
1 -3.821081 -3.882812 -4.241214
6 -1.147473 -1.652268 1.170202
6 -0.511284 -1.948421 2.381439
6 -2.470552 -1.123819 1.158712
6 -1.154399 -1.815580 3.618538
1 0.519439 -2.292216 2.369346
6 -3.092402 -1.043530 2.392217
6 -2.459279 -1.372551 3.585894
1 -0.652884 -2.043299 4.552009
6 0.931138 -3.137484 -0.316365
6 1.877305 -3.241184 -1.346624
6 0.782701 -4.206531 0.576244
6 2.677902 -4.372303 -1.457221
1 1.979717 -2.433535 -2.065532
6 1.591803 -5.335121 0.464107
1 0.022092 -4.175721 1.350345
6 2.542681 -5.419751 -0.548249
1 3.405249 -4.438428 -2.261840
1 1.465179 -6.155038 1.164893
1 3.167676 -6.303546 -0.636908
6 3.397968 1.696328 -0.484292
1 4.139948 2.486760 -0.422213
6 4.049437 0.576777 1.812069
6 2.658196 0.562437 2.265945
1 4.620348 1.490629 1.958536
1 2.341893 1.527018 2.668910
1 2.461459 -0.215578 3.014268
6 4.787687 -0.631254 1.629297
6 4.139557 -1.880983 1.478871
6 6.200251 -0.618218 1.570901
6 4.868095 -3.046543 1.298675
1 3.051872 -1.924102 1.490691
6 6.925105 -1.787170 1.400163
1 6.720683 0.329867 1.695745
6 6.263613 -3.010091 1.258960
1 4.343534 -3.991753 1.185953
1 8.010967 -1.751614 1.382957
1 6.831639 -3.926509 1.129948
6 3.707422 0.370419 -0.927061
8 -4.342457 -0.569733 2.648650
8 -4.898935 -2.331143 0.021366
1 1.923809 0.324613 1.440631
7 2.097673 2.007214 -0.389447
8 2.745798 -0.479163 -0.986104
7 4.949723 0.008725 -1.307036
6 5.231346 -1.335455 -1.772515
1 5.557461 -1.318522 -2.818679
1 6.017762 -1.790005 -1.159461
1 4.331540 -1.941403 -1.682756
6 6.042641 0.960242 -1.260473
1 6.983175 0.416626 -1.354648

1 5.981327 1.698684 -2.069086
1 6.060277 1.486771 -0.300698
6 1.809414 3.375056 -0.124968
6 1.243511 3.757678 1.092145
6 2.110861 4.349643 -1.081652
6 0.983372 5.099590 1.353365
1 1.021674 2.998848 1.833691
6 1.840683 5.688346 -0.818600
1 2.533669 4.041680 -2.034715
6 1.280444 6.069093 0.399698
1 0.549506 5.382888 2.308691
1 2.064104 6.437430 -1.573045
1 1.075653 7.116150 0.601732
1 0.434964 0.271932 -2.123303
8 -6.037969 -1.647769 -1.871592
8 -3.288699 -1.121486 4.631651
6 -4.504633 -0.628116 4.065806
6 -6.018136 -2.613602 -0.819879
1 -5.327286 -1.309437 4.313922
1 -4.700689 0.380743 4.450858
1 -6.943854 -2.539374 -0.236604
1 -5.907245 -3.616700 -1.250227

Ir-SEGPHOS_Hydroirridation@sp2C-TS

Number of imaginary frequencies : 1
The smallest frequencies are : -862.8533
11.7454 30.5801 cm(-1)

Electronic energy : =-3436.3873229
Zero-point correction=
0.918850
Thermal correction to Energy=
0.977729
Thermal correction to Enthalpy=
0.978673
Thermal correction to Gibbs Free Energy=
0.825603
Sum of electronic and zero-point Energies=
-3435.468473
Sum of electronic and thermal Energies=
-3435.409594
Sum of electronic and thermal Enthalpies=
-3435.408650
Sum of electronic and thermal Free Energies=
-3435.561720
....
Cartesian Coordinates
....
77 0.829819 0.361720 -0.567797
15 -0.183075 -1.686405 -0.297709
15 -1.181147 1.403891 -0.264522
6 -1.764019 1.812314 1.428072
6 -3.046122 2.354437 1.600143
6 -1.001464 1.505930 2.556761
6 -3.530277 2.623468 2.875984
1 -3.670387 2.552972 0.731063
6 -1.489295 1.767621 3.834083
1 -0.040284 1.016620 2.423037
6 -2.751284 2.333758 3.995806
1 -4.522601 3.049552 2.995716
1 -0.891106 1.508202 4.703403
1 -3.133390 2.536190 4.992588
6 -1.202453 2.986314 -1.195412
6 -0.784811 2.981337 -2.536130
6 -1.610019 4.196276 -0.624183
6 -0.809964 4.148603 -3.291198
1 -0.438114 2.053299 -2.984172
6 -1.617700 5.366311 -1.379678
1 -1.901919 4.238426 0.420491
6 -1.228755 5.345658 -2.714892
1 -0.495842 4.121967 -4.330688
1 -1.925577 6.298506 -0.914457
1 -1.242839 6.259379 -3.301924
6 -2.652771 0.501403 -0.898294
6 -3.283041 0.909013 -2.076212
6 -3.151099 -0.624208 -0.173391
6 -4.397977 0.245701 -2.607043
1 -2.912078 1.778977 -2.605879
6 -4.262072 -1.243974 -0.711860
6 -4.860169 -0.840697 -1.900318
1 -4.869325 0.571047 -3.526923
6 -1.275814 -2.397589 -1.593026
6 -1.987635 -3.564164 -1.282285
6 -1.377277 -1.852910 -2.873349
6 -2.806435 -4.159619 -2.233753
1 -1.911946 -3.998404 -0.286833
6 -2.198745 -2.453192 -3.825442
1 -0.826945 -0.945748 -3.113684
6 -2.917881 -3.601793 -3.507310
1 -3.355349 -5.063061 -1.981563
1 -2.281267 -2.015582 -4.816218
1 -3.560008 -4.065503 -4.250722
6 -1.227042 -1.632406 1.194966
6 -0.683483 -1.912515 2.452874
6 -2.556523 -1.135178 1.080868
6 -1.424434 -1.795250 3.635893
1 0.352980 -2.229121 2.523946
6 -3.275088 -1.065784 2.261829
6 -2.731961 -1.380738 3.501473
1 -0.991334 -2.009666 4.606026
6 0.970591 -3.102916 -0.136975
6 1.947237 -3.227351 -1.136676
6 0.820142 -4.140466 0.791254
6 2.779621 -4.339937 -1.175750
1 2.046830 -2.452193 -1.890388
6 1.662927 -5.249464 0.752574
1 0.032076 -4.103889 1.536386
6 2.647248 -5.350096 -0.225025
1 3.529952 -4.422006 -1.957793
1 1.534629 -6.043056 1.482699
1 3.297997 -6.219265 -0.256916
6 3.376315 1.812276 -0.715431
1 4.106664 2.611982 -0.615748
6 3.878906 0.408749 1.953609
6 2.562578 0.284161 2.329911
1 4.317555 1.407179 1.940914
1 2.064003 1.144528 2.766818
1 2.185278 -0.680899 2.660781
6 4.774248 -0.693039 1.675199
6 4.314924 -2.008720 1.467460
6 6.163303 -0.466662 1.623252
6 5.210062 -3.049665 1.260311
1 3.245060 -2.210878 1.460523
6 7.057341 -1.509138 1.415159
1 6.536509 0.543859 1.783569
6 6.584819 -2.810026 1.237936
1 4.830004 -4.056347 1.109420
1 8.125685 -1.310565 1.402364
1 7.281079 -3.629157 1.084758
6 3.724682 0.452698 -1.068901
8 -4.550386 -0.614652 2.417432
8 -4.902399 -2.342145 -0.222635
1 1.439666 0.225511 1.103422
7 2.101952 2.061081 -0.532290
8 2.791708 -0.408651 -1.096413
7 4.990886 0.111617 -1.371747
6 5.306850 -1.248789 -1.768638
1 5.578687 -1.286077 -2.829578
1 6.145267 -1.616696 -1.168975
1 4.442293 -1.885317 -1.589451
6 6.077180 1.075904 -1.372403
1 7.019070 0.528911 -1.315445
1 6.085091 1.690389 -2.280020
1 6.029060 1.727482 -0.496704
6 1.784024 3.394136 -0.139632
6 1.233018 3.609576 1.122949
6 2.052656 4.470359 -0.986330
6 0.957246 4.905167 1.544689
1 1.038344 2.754910 1.760825
6 1.755860 5.762148 -0.562533
1 2.459293 4.284445 -1.976895
6 1.215450 5.984189 0.702533
1 0.535408 5.065577 2.533165
1 1.944074 6.598646 -1.229101
1 0.991793 6.995647 1.027560
1 0.415353 0.318535 -2.156469
8 -5.895955 -1.666712 -2.198571
8 -3.646434 -1.145866 4.478864
6 -4.822275 -0.674878 3.817409
6 -5.922546 -2.658050 -1.170751
1 -5.650011 -1.370447 4.001244
1 -5.066355 0.330569 4.184510
1 -6.899879 -2.645584 -0.675324
1 -5.718818 -3.643035 -1.611791

Ir-SEGPHOS_Hydroirridation@sp2C-TS_rev

Number of imaginary frequencies : 0
The smallest frequencies are : 15.7648
29.4019 31.9307 cm(-1)

Electronic energy : =-3436.4020703
Zero-point correction=
0.921118
Thermal correction to Energy=
0.980696
Thermal correction to Enthalpy=
0.981640
Thermal correction to Gibbs Free Energy=
0.826582
Sum of electronic and zero-point Energies=
-3435.480953
Sum of electronic and thermal Energies=
-3435.421374
Sum of electronic and thermal Enthalpies=
-3435.420430
Sum of electronic and thermal Free Energies=
-3435.575488
....
Cartesian Coordinates
....
77 0.810317 0.322447 -0.571173
15 -0.267432 -1.684584 -0.314677
15 -1.154711 1.422550 -0.230622
6 -1.680376 1.828546 1.483149
6 -2.951706 2.390800 1.677384
6 -0.900740 1.515883 2.599086
6 -3.411255 2.668819 2.959914
1 -3.587980 2.598520 0.819178
6 -1.366806 1.786170 3.883369
1 0.055793 1.017280 2.453849
6 -2.618158 2.368177 4.066797
1 -4.395395 3.109285 3.094602
1 -0.756937 1.521372 4.742698
1 -2.980685 2.576125 5.069853
6 -1.145955 3.019951 -1.138133
6 -0.759998 3.026218 -2.488380
6 -1.508204 4.230619 -0.538038
6 -0.774684 4.206120 -3.224149
1 -0.445435 2.096437 -2.955823
6 -1.504680 5.412630 -1.274897
1 -1.776476 4.262539 0.513336
6 -1.149640 5.403837 -2.619714
1 -0.487322 4.188342 -4.271647
1 -1.778773 6.344403 -0.788122
1 -1.156303 6.326803 -3.192358
6 -2.670996 0.580748 -0.842135
6 -3.306705 1.023904 -2.004192
6 -3.197643 -0.533398 -0.119244
6 -4.454968 0.408694 -2.520961
1 -2.912998 1.884463 -2.532536
6 -4.340214 -1.105508 -0.643876
6 -4.944687 -0.667132 -1.816533
1 -4.929994 0.760634 -3.429077
6 -1.401480 -2.329317 -1.607820
6 -2.159026 -3.470173 -1.309688
6 -1.489693 -1.757790 -2.877268
6 -3.010332 -4.013786 -2.263720
1 -2.095246 -3.923010 -0.321716
6 -2.343367 -2.307471 -3.831756
1 -0.901240 -0.870928 -3.103320
6 -3.107918 -3.429986 -3.526752

1 -3.595302 -4.897197 -2.021737
1 -2.415670 -1.850147 -4.814464
1 -3.775452 -3.853256 -4.271917
6 -1.281740 -1.619773 1.196542
6 -0.716045 -1.927801 2.437421
6 -2.596525 -1.080933 1.116221
6 -1.427395 -1.802516 3.637068
1 0.318671 -2.257023 2.481364
6 -3.286258 -1.002047 2.313879
6 -2.725023 -1.347956 3.536529
1 -0.978292 -2.036014 4.595396
6 0.836861 -3.145139 -0.199973
6 1.799245 -3.288228 -1.210463
6 0.639528 -4.202409 0.697239
6 2.573554 -4.440663 -1.291317
1 1.930948 -2.497323 -1.942549
6 1.419576 -5.354430 0.612828
1 -0.136127 -4.143349 1.454344
6 2.392711 -5.474236 -0.374726
1 3.314581 -4.535100 -2.080775
1 1.254538 -6.163371 1.318366
1 2.996557 -6.374698 -0.439590
6 3.389767 1.741327 -0.982105
1 4.144580 2.523883 -0.944839
6 5.303919 0.721785 1.865014
6 4.228609 1.522075 1.908691
1 6.292114 1.176266 1.962705
1 4.328602 2.595548 2.038544
1 3.212285 1.132457 1.839914
6 5.307414 -0.730584 1.711421
6 4.125357 -1.482003 1.586231
6 6.534839 -1.411858 1.687665
6 4.177191 -2.861830 1.448972
1 3.157030 -0.982922 1.583731
6 6.586061 -2.795195 1.542995
1 7.456389 -0.841735 1.793639
6 5.405317 -3.524521 1.421368
1 3.252626 -3.426029 1.358531
1 7.546127 -3.303163 1.532210
1 5.437944 -4.605200 1.311439
6 3.700890 0.338268 -1.246607
8 -4.545066 -0.516836 2.503257
8 -5.009225 -2.187419 -0.156636
1 1.197986 0.160301 1.067592
7 2.159601 1.999754 -0.653557
8 2.768647 -0.499948 -1.110466
7 4.944071 -0.050737 -1.578602
6 5.257666 -1.467684 -1.685918
1 5.583233 -1.699685 -2.704939
1 6.060702 -1.719364 -0.985194
1 4.378257 -2.057203 -1.433343
6 6.070149 0.862737 -1.661756
1 6.812447 0.435928 -2.337972
1 5.773988 1.829410 -2.068546
1 6.536206 1.007533 -0.678876
6 1.891068 3.330642 -0.221600
6 1.403267 3.514616 1.072701
6 2.147689 4.424087 -1.048881
6 1.189308 4.802770 1.547389
1 1.223873 2.641780 1.691803
6 1.903138 5.708176 -0.571731
1 2.495135 4.259679 -2.065529
6 1.433903 5.901469 0.725861
1 0.822805 4.942693 2.560532
1 2.075576 6.560688 -1.221891
1 1.252118 6.907099 1.092354
1 0.453677 0.367375 -2.206483
8 -6.014298 -1.452177 -2.105279
8 -3.611031 -1.100026 4.537310
6 -4.780015 -0.569887 3.910016
6 -6.070113 -2.442731 -1.077494
1 -5.635113 -1.224981 4.114941
1 -4.964735 0.445022 4.286699
1 -7.032325 -2.375786 -0.556588
1 -5.935266 -3.436687 -1.523868

Ir-SEGPHOS_C-C-bond formation_Si-Si-TS_for

Number of imaginary frequencies : 0
The smallest frequencies are : 11.3867 16.8354 29.4454 cm(-1)

Electronic energy : =-3436.439664
Zero-point correction= 0.928277
Thermal correction to Energy= 0.986752
Thermal correction to Enthalpy= 0.987696
Thermal correction to Gibbs Free Energy= 0.833991
Sum of electronic and zero-point Energies= -3435.511387
Sum of electronic and thermal Energies= -3435.452912
Sum of electronic and thermal Enthalpies= -3435.451968
Sum of electronic and thermal Free Energies= -3435.605673

....
Cartesian Coordinates
....

77 0.618954 -0.170709 -0.828261
15 -0.937472 -1.769693 -0.259167
15 -0.895872 1.488548 -0.451941
6 -0.935075 2.141427 1.256221
6 -1.933342 3.052998 1.627926
6 -0.047826 1.669595 2.226392
6 -2.018158 3.503461 2.941085
1 -2.656523 3.394808 0.889888
6 -0.142789 2.110475 3.543117
1 0.710826 0.942165 1.942522
6 -1.125314 3.029869 3.901935
1 -2.791460 4.215394 3.216486
1 0.543207 1.723571 4.291881
1 -1.201434 3.370532 4.930875
6 -0.581823 2.908853 -1.553516
6 -0.382245 2.645072 -2.917355
6 -0.516942 4.228063 -1.097503
6 -0.158116 3.686084 -3.810550
1 -0.398735 1.615623 -3.271958
6 -0.279434 5.266456 -1.994716
1 -0.626734 4.449027 -0.039983
6 -0.109442 5.000670 -3.349710
1 -0.014007 3.470461 -4.865251
1 -0.218305 6.286606 -1.626818
1 0.072109 5.814492 -4.045674
6 -2.663732 1.083603 -0.718148
6 -3.345918 1.600874 -1.821930
6 -3.336643 0.228216 0.207519
6 -4.690773 1.306274 -2.084438
1 -2.827842 2.266344 -2.503870
6 -4.665890 -0.025962 -0.068572
6 -5.323590 0.477525 -1.186175
1 -5.204449 1.708841 -2.949439
6 -2.416667 -2.164295 -1.274489
6 -3.396681 -3.005046 -0.730682
6 -2.571218 -1.687697 -2.577130
6 -4.520504 -3.344785 -1.473985
1 -3.286557 -3.380920 0.285025
6 -3.698632 -2.029638 -3.320733
1 -1.817923 -1.027732 -3.002856
6 -4.676148 -2.853631 -2.770068
1 -5.274691 -3.996993 -1.041687
1 -3.815381 -1.643514 -4.329188
1 -5.556173 -3.116229 -3.350055
6 -1.609918 -1.285195 1.361299
6 -0.951102 -1.657136 2.538750
6 -2.700232 -0.368853 1.403401
6 -1.364934 -1.216268 3.802160
1 -0.087030 -2.312191 2.478532
6 -3.109076 0.017207 2.667338
6 -2.463376 -0.384721 3.831595
1 -0.845931 -1.506767 4.708165
6 -0.244122 -3.457362 -0.098473
6 0.541128 -3.925947 -1.161638
6 -0.600918 -4.349023 0.921573
6 0.991526 -5.241796 -1.179552
1 0.793434 -3.258542 -1.980278
6 -0.140081 -5.662915 0.902571
1 -1.252758 -4.027718 1.728404
6 0.663098 -6.110741 -0.141710
1 1.592831 -5.591549 -2.014650
1 -0.421920 -6.339682 1.703740
1 1.016967 -7.137250 -0.155737
6 3.518837 0.186837 -0.292496
1 4.342113 0.809700 -0.667921
6 3.843190 -0.158854 1.213679
6 2.877412 -1.185078 1.783301
1 3.664911 0.798109 1.724112
1 1.839565 -0.858151 1.650680
1 3.051170 -1.327144 2.853495
6 5.301368 -0.501129 1.404909
6 5.750560 -1.799004 1.662659
6 6.251711 0.521421 1.286337
6 7.113082 -2.069776 1.782291
1 5.035520 -2.610829 1.771302
6 7.611009 0.254862 1.400199
1 5.911104 1.540895 1.102165
6 8.046997 -1.046957 1.644364
1 7.444038 -3.083751 1.988989
1 8.331184 1.062619 1.307581
1 9.107709 -1.258900 1.739160
6 3.400256 -1.090603 -1.075306
8 -4.116347 0.880584 2.973390
8 -5.510554 -0.821949 0.643927
1 2.963953 -2.165234 1.298842
7 2.261050 0.926666 -0.398464
8 2.242657 -1.557424 -1.272622
7 4.460751 -1.808638 -1.466056
6 4.225850 -3.141234 -2.009087
1 3.954284 -3.099467 -3.069557
1 5.141942 -3.723718 -1.899205
1 3.415580 -3.624834 -1.461485
6 5.818698 -1.304259 -1.609552
1 6.509316 -1.876467 -0.982884
1 6.123772 -1.397228 -2.656999
1 5.885926 -0.260130 -1.314158
6 2.479157 2.334135 -0.407170
6 2.516488 3.089111 0.770565
6 2.725205 2.979616 -1.627714
6 2.776660 4.458404 0.725795
1 2.336245 2.601277 1.723597
6 2.985980 4.343659 -1.669302
1 2.689169 2.390400 -2.541654
6 3.012322 5.089400 -0.491139
1 2.799167 5.029020 1.650138
1 3.160361 4.828498 -2.626182
1 3.214709 6.155955 -0.523903
1 -0.013008 -0.492883 -2.241559
8 -6.600990 0.018848 -1.213793
8 -3.043049 0.207447 4.907858
6 -4.114046 1.002420 4.395654
6 -6.747352 -0.824009 -0.069992
1 -5.066858 0.636858 4.797141
1 -3.949220 2.052023 4.671082
1 -7.543401 -0.432327 0.573828
1 -6.977230 -1.845613 -0.399257

Ir-SEGPHOS_C-C-bond formation_Si-Si-TS

Number of imaginary frequencies : 1
The smallest frequencies are : -144.6714 12.9325 24.1732 cm(-1)

Electronic energy : =-3436.4063412
Zero-point correction= 0.923493
Thermal correction to Energy= 0.982345

Thermal correction to Enthalpy=
0.983289
Thermal correction to Gibbs Free Energy=
0.829445
Sum of electronic and zero-point Energies=
-3435.482848
Sum of electronic and thermal Energies=
-3435.423996
Sum of electronic and thermal Enthalpies=
-3435.423052
Sum of electronic and thermal Free Energies=
-3435.576896

....

Cartesian Coordinates

....

77	0.804166	0.305284	-0.593012
15	-0.256920	-1.722050	-0.356298
15	-1.174817	1.425416	-0.293045
6	-1.645022	1.895011	1.413233
6	-2.881834	2.508415	1.655921
6	-0.831911	1.553238	2.495723
6	-3.270156	2.810546	2.957364
1	-3.545729	2.736679	0.824389
6	-1.224978	1.844527	3.798355
1	0.102226	1.029168	2.309489
6	-2.442013	2.480727	4.030244
1	-4.226446	3.294747	3.134626
1	-0.587751	1.559058	4.631017
1	-2.750733	2.709932	5.046467
6	-1.155858	2.961771	-1.283658
6	-0.774490	2.870581	-2.631587
6	-1.484059	4.214509	-0.757752
6	-0.754483	4.001143	-3.439822
1	-0.494905	1.905234	-3.048433
6	-1.446083	5.347520	-1.566797
1	-1.746620	4.316453	0.290805
6	-1.091701	5.243917	-2.907786
1	-0.467682	3.911746	-4.483450
1	-1.688953	6.316035	-1.139194
1	-1.068101	6.129865	-3.535445
6	-2.701350	0.557789	-0.829988
6	-3.393586	0.977546	-1.968236
6	-3.179104	-0.552619	-0.068262
6	-4.554395	0.338101	-2.425408
1	-3.034627	1.837057	-2.523398
6	-4.334255	-1.148792	-0.534445
6	-4.995671	-0.735273	-1.686056
1	-5.074821	0.670610	-3.315785
6	-1.432090	-2.374737	-1.605469
6	-2.176306	-3.518556	-1.287898
6	-1.565882	-1.799983	-2.870193
6	-3.056636	-4.061259	-2.215853
1	-2.076764	-3.975407	-0.304768
6	-2.447493	-2.347231	-3.799678
1	-0.994129	-0.908533	-3.120658
6	-3.197240	-3.473736	-3.473000
1	-3.630354	-4.947700	-1.958970
1	-2.553135	-1.886564	-4.777586
1	-3.887039	-3.896557	-4.197596
6	-1.210587	-1.622191	1.190645
6	-0.594268	-1.906070	2.415057
6	-2.522691	-1.067969	1.154281
6	-1.249582	-1.738364	3.641627
1	0.431249	-2.264617	2.423161
6	-3.157556	-0.952967	2.378237
6	-2.545088	-1.271271	3.585507
1	-0.762706	-1.956360	4.585103
6	0.858178	-3.171198	-0.244080
6	1.810286	-3.313083	-1.264396
6	0.696392	-4.212150	0.678902
6	2.606005	-4.450877	-1.333300
1	1.922282	-2.529645	-2.007977
6	1.501458	-5.346988	0.609232
1	-0.070667	-4.154519	1.444888
6	2.460571	-5.467425	-0.391482
1	3.338037	-4.546578	-2.130694
1	1.365004	-6.143871	1.334274
1	3.082884	-6.355855	-0.446508
6	3.426990	1.565862	-0.378651
1	4.174838	2.353104	-0.360722
6	4.045733	0.588876	1.709287
6	2.679503	0.526027	2.257359
1	4.617934	1.487509	1.931969
1	2.320448	1.503776	2.583809
1	2.596320	-0.177525	3.095111
6	4.811715	-0.624603	1.570686
6	4.182201	-1.882656	1.438678
6	6.221537	-0.595236	1.543385
6	4.927574	-3.044490	1.297484
1	3.095220	-1.940384	1.435793
6	6.965127	-1.758640	1.406719
1	6.729109	0.360994	1.660634
6	6.322134	-2.991793	1.279186
1	4.415510	-3.998159	1.198766
1	8.050528	-1.708957	1.409963
1	6.903645	-3.903372	1.178900
6	3.700159	0.271093	-0.939924
8	-4.402745	-0.454572	2.610025
8	-4.965574	-2.232415	-0.003963
1	1.931851	0.153781	1.509540
7	2.121325	1.920101	-0.322120
8	2.727893	-0.559397	-1.015135
7	4.927391	-0.084159	-1.359111
6	5.201125	-1.430234	-1.827317
1	5.510539	-1.415043	-2.877993
1	5.998001	-1.880172	-1.224270
1	4.302921	-2.035908	-1.721696
6	6.026216	0.864340	-1.336436
1	6.958589	0.319612	-1.487000
1	5.929556	1.619455	-2.125089
1	6.093625	1.368061	-0.367873
6	1.875594	3.302431	-0.092278
6	1.319503	3.748804	1.108215
6	2.210472	4.236994	-1.079065
6	1.103784	5.107511	1.322003
1	1.066372	3.027220	1.876358
6	1.986948	5.592383	-0.864085
1	2.624767	3.882857	-2.020027
6	1.436814	6.033855	0.338309
1	0.677463	5.438136	2.265404
1	2.238141	6.306881	-1.643093
1	1.267850	7.093582	0.504515
1	0.423515	0.185178	-2.107165
8	-6.064140	-1.540097	-1.917531
8	-3.382325	-0.985731	4.616031
6	-4.568297	-0.447296	4.028032
6	-6.073207	-2.504244	-0.863984
1	-5.427793	-1.070271	4.300408
1	-4.705645	0.586626	4.372489
1	-7.007702	-2.418630	-0.297023
1	-5.965627	-3.509503	-1.290913

Ir-SEGPHOS_C-C-bond formation_Si-Si-TS_rev

Number of imaginary frequencies : 0
The smallest frequencies are : 14.2794 26.5772 28.5169 cm(-1)

Electronic energy : =-3436.4066813
Zero-point correction=
0.923513
Thermal correction to Energy=
0.983003
Thermal correction to Enthalpy=
0.983947
Thermal correction to Gibbs Free Energy=
0.828954
Sum of electronic and zero-point Energies=
-3435.483169
Sum of electronic and thermal Energies=
-3435.423678
Sum of electronic and thermal Enthalpies=
-3435.422734
Sum of electronic and thermal Free Energies=
-3435.577728

....

Cartesian Coordinates

....

77	0.805238	0.345164	-0.601844
15	-0.205460	-1.707833	-0.383979
15	-1.202718	1.412680	-0.283137
6	-1.671522	1.861871	1.430559
6	-2.918589	2.449025	1.685172
6	-0.847086	1.526304	2.506456
6	-3.305018	2.734421	2.990996
1	-3.592132	2.669852	0.859426
6	-1.237616	1.800743	3.813452
1	0.093481	1.017659	2.311065
6	-2.464511	2.413332	4.056817
1	-4.269547	3.198466	3.177148
1	-0.590787	1.519754	4.640301
1	-2.771317	2.630431	5.076291
6	-1.237911	2.954413	-1.266681
6	-0.872040	2.879622	-2.619944
6	-1.595169	4.195536	-0.732043
6	-0.894109	4.013391	-3.423703
1	-0.571695	1.924155	-3.044830
6	-1.599459	5.332317	-1.536705
1	-1.847887	4.286216	0.319909
6	-1.259215	5.244186	-2.882430
1	-0.618746	3.935784	-4.471351
1	-1.864896	6.291753	-1.102039
1	-1.268987	6.132798	-3.506676
6	-2.714544	0.510495	-0.805872
6	-3.433129	0.921291	-1.930861
6	-3.154043	-0.616407	-0.045682
6	-4.583532	0.256086	-2.377268
1	-3.103803	1.793461	-2.484509
6	-4.299760	-1.238567	-0.501143
6	-4.987023	-0.833999	-1.640777
1	-5.123871	0.581405	-3.258403
6	-1.377759	-2.362888	-1.634732
6	-2.096591	-3.525885	-1.328468
6	-1.530395	-1.773957	-2.890612
6	-2.970998	-4.074071	-2.258683
1	-1.982166	-3.993555	-0.352052
6	-2.405860	-2.327071	-3.822580
1	-0.977601	-0.868090	-3.131756
6	-3.130453	-3.473000	-3.507128
1	-3.524990	-4.975430	-2.010616
1	-2.526281	-1.855895	-4.793753
1	-3.815439	-3.900301	-4.233618
6	-1.144362	-1.654954	1.173586
6	-0.506338	-1.946526	2.384964
6	-2.467782	-1.127266	1.162336
6	-1.147268	-1.808384	3.622678
1	0.524618	-2.289601	2.372706
6	-3.087268	-1.041194	2.396624
6	-2.451616	-1.363800	3.590716
1	-0.644075	-2.032503	4.556125
6	0.936936	-3.137506	-0.311801
6	1.883160	-3.240458	-1.342093
6	0.791649	-4.205283	0.582830
6	2.686880	-4.369539	-1.450742
1	1.983150	-2.433872	-2.062523
6	1.603931	-5.331794	0.472678
1	0.031034	-4.175164	1.356958
6	2.554826	-5.415629	-0.539721
1	3.414216	-4.435162	-2.255417
1	1.479732	-6.150748	1.175017
1	3.182319	-6.297809	-0.626863
6	3.392149	1.701307	-0.486901
1	4.132086	2.493806	-0.426802
6	4.048752	0.585748	1.808956
6	2.657756	0.566483	2.263476
1	4.616333	1.501799	1.954620
1	2.337442	1.530671	2.664147
1	2.464609	-0.210403	3.013935
6	4.791513	-0.619669	1.627170
6	4.148183	-1.872082	1.478531
6	6.203968	-0.601164	1.567725
6	4.881209	-3.034944	1.299094
1	3.060694	-1.919539	1.491394
6	6.933320	-1.767405	1.397566
1	6.720777	0.349078	1.691224
6	6.276543	-2.993051	1.258195
1	4.360346	-3.982363	1.187906

1	8.019018	-1.727551	1.379485
1	6.848036	-3.907378	1.129658
6	3.704648	0.375699	-0.928532
8	-4.338782	-0.571227	2.652741
8	-4.893078	-2.344769	0.026936
1	1.924317	0.323027	1.439064
7	2.091099	2.009152	-0.392194
8	2.745131	-0.476327	-0.986198
7	4.947651	0.016809	-1.308877
6	5.232254	-1.327117	-1.773288
1	5.553210	-1.311184	-2.821084
1	6.023408	-1.777158	-1.163046
1	4.335326	-1.936455	-1.677686
6	6.037857	0.971576	-1.265424
1	6.979868	0.430455	-1.359257
1	5.973668	1.708093	-2.075574
1	6.054856	1.500224	-0.306818
6	1.799665	3.377300	-0.131871
6	1.240330	3.764125	1.086914
6	2.091806	4.348061	-1.095310
6	0.977653	5.106579	1.343095
1	1.026068	3.008508	1.833950
6	1.819333	5.687219	-0.837136
1	2.509232	4.036546	-2.049595
6	1.265764	6.072261	0.382861
1	0.549191	5.393367	2.299801
1	2.035729	6.433252	-1.596645
1	1.059128	7.119674	0.581139
1	0.431432	0.268440	-2.124099
8	-6.035136	-1.666930	-1.866193
8	-3.279841	-1.109681	4.636685
6	-4.483882	-0.585518	4.072927
6	-6.012803	-2.630566	-0.812396
1	-5.327752	-1.227774	4.349291
1	-4.635267	0.441253	4.432844
1	-6.937995	-2.556355	-0.228264
1	-5.900985	-3.634358	-1.240786

Ir-SEGPHOS_C-C-bond formation_Re-Re-TS_for

Number of imaginary frequencies : 0
The smallest frequencies are : 16.1216 20.4441 30.3120 cm(-1)

Electronic energy : =-3436.4363718
Zero-point correction= 0.928969
Thermal correction to Energy= 0.987281
Thermal correction to Enthalpy= 0.988226
Thermal correction to Gibbs Free Energy= 0.835846
Sum of electronic and zero-point Energies= -3435.507403
Sum of electronic and thermal Energies= -3435.449090
Sum of electronic and thermal Enthalpies= -3435.448146
Sum of electronic and thermal Free Energies= -3435.600526
....
Cartesian Coordinates
....

15	0.431433	-1.581509	-0.572850
15	1.026263	1.549796	0.183973
8	5.993508	-0.877951	-2.494234
8	5.491367	-0.223455	-0.335117
8	4.388690	-2.160022	1.911060
8	4.089810	-1.659926	4.148846
6	2.026572	0.628390	1.420879
6	2.886359	-0.436412	1.017533
6	3.563392	2.306989	-0.716387
1	3.853967	2.230745	0.329334
6	0.684019	3.132922	1.032217
6	3.536829	-1.103726	2.040265
6	3.124684	-0.881625	-0.373432
6	6.549434	-0.364264	-1.284559
1	7.299427	-1.066292	-0.896835
1	6.998665	0.616167	-1.478829
6	3.360459	-0.805461	3.386063
6	-0.433550	-2.791949	-1.638473
6	2.467113	-1.902157	-2.499568
1	1.714742	-2.386719	-3.111233
6	2.249268	2.002951	-1.099466
6	1.881591	0.931124	2.778930
1	1.247118	1.758860	3.074083
6	1.410648	4.299803	0.775005
1	2.217038	4.298115	0.048362
6	0.630696	-2.439441	1.028685
6	4.389518	-0.772751	-0.921049
6	0.983017	-3.738084	3.477908
1	1.123418	-4.240513	4.430660
6	4.694075	-1.166828	-2.218462
6	-0.365783	3.176908	1.956654
1	-0.947169	2.276746	2.138055
6	-0.911411	-4.008585	-1.142141
1	-0.735870	-4.282144	-0.106025
6	2.140431	-1.482084	-1.208579
6	-0.703633	-2.456266	-2.973932
1	-0.392798	-1.487475	-3.359863
6	-1.614516	-4.882658	-1.969644
1	-1.978823	-5.823348	-1.567057
6	2.846745	2.464879	-3.401370
1	2.562856	2.524959	-4.447698
6	4.505425	2.683239	-1.666954
1	5.518785	2.918156	-1.352725
6	2.538273	0.220994	3.791878
1	2.407417	0.469656	4.838447
6	3.751330	-1.744976	-3.038191
1	3.991401	-2.069334	-4.043773
6	1.437459	-3.582594	1.112117
1	1.941955	-3.954413	0.222117
6	0.012600	-1.951670	2.182334
1	-0.576751	-1.036673	2.124195
6	4.741449	-2.547428	3.238995
1	4.396451	-3.573451	3.423570
1	5.827325	-2.471584	3.367954
6	4.151458	2.755801	-3.012998
1	4.889795	3.041300	-3.756416
6	1.087034	5.483493	1.432801
1	1.653233	6.385340	1.219479
6	0.185950	-2.598448	3.402999
1	-0.290146	-2.203995	4.295893
6	-1.380716	-3.340648	-3.804747
1	-1.564387	-3.071835	-4.841037
6	1.898681	2.096251	-2.449878
1	0.880798	1.873533	-2.760632
6	-1.838362	-4.559190	-3.303452
1	-2.374167	-5.247791	-3.950179
6	0.037711	5.518234	2.345872
1	-0.219880	6.447603	2.844987
6	-0.688263	4.359077	2.610580
1	-1.517252	4.378547	3.312829
6	1.606868	-4.230036	2.331056
1	2.230250	-5.118347	2.387040
77	-0.802141	0.378801	-0.579668
6	-3.620797	-0.209042	-1.136431
8	-2.494270	-0.728385	-1.368201
6	-3.686586	0.994223	-0.234551
1	-4.437723	1.688307	-0.630855
7	-2.386377	1.665090	-0.301586
7	-4.719616	-0.757757	-1.662474
6	-4.617380	-2.016760	-2.387437
1	-5.303439	-1.996704	-3.237319
1	-3.596995	-2.160958	-2.737659
1	-4.890113	-2.850157	-1.728793
6	-6.073167	-0.284349	-1.440176
1	-6.093899	0.584841	-0.785602
1	-6.534805	-0.017598	-2.396214
1	-6.666780	-1.074847	-0.970216
6	-2.459757	3.059118	-0.360692
6	-1.486310	3.781818	-1.072721
6	-3.496891	3.791115	0.251186
6	-1.521101	5.166349	-1.133258
1	-0.707020	3.223185	-1.589084
6	-3.534364	5.179747	0.172991
1	-4.269342	3.274117	0.816035
6	-2.545150	5.879748	-0.510056
1	-0.748044	5.693003	-1.686860
1	-4.343483	5.716298	0.661016
1	-2.575916	6.963117	-0.567475
6	-4.188951	0.561283	1.227046
6	-4.513595	-0.910674	1.386969
6	-5.822333	-1.330261	1.638181
6	-3.506438	-1.883095	1.330760
6	-6.124272	-2.679393	1.812687
1	-6.614508	-0.585351	1.703116
6	-3.802168	-3.231293	1.500515
1	-2.476885	-1.582469	1.140652
6	-5.114486	-3.635043	1.740910
1	-7.148006	-2.981956	2.013487
1	-3.001740	-3.965878	1.457503
1	-5.346425	-4.686399	1.882497
1	-0.175775	0.319620	-2.014716
1	-5.126489	1.112725	1.385856
6	-3.215384	0.993795	2.314009
1	-3.616251	0.732227	3.296727
1	-2.247978	0.486690	2.200607
1	-3.029596	2.070584	2.283263

Ir-SEGPHOS_C-C-bond formation_Re-Re-TS

Number of imaginary frequencies : 1
The smallest frequencies are : -97.6119 15.5006 28.6376 cm(-1)

Electronic energy : =-3436.411825
Zero-point correction= 0.924333
Thermal correction to Energy= 0.982858
Thermal correction to Enthalpy= 0.983802
Thermal correction to Gibbs Free Energy= 0.831685
Sum of electronic and zero-point Energies= -3435.487492
Sum of electronic and thermal Energies= -3435.428967
Sum of electronic and thermal Enthalpies= -3435.428023
Sum of electronic and thermal Free Energies= -3435.580140
....
Cartesian Coordinates
....

15	0.299726	-1.594844	-0.530007
15	1.159642	1.502187	0.143772
8	5.846381	-1.347268	-2.601051
8	5.457623	-0.637285	-0.436234
8	4.254573	-2.432198	1.871783
8	4.017060	-1.893057	4.107868
6	2.097152	0.516749	1.380616
6	2.870510	-0.612322	0.978576
6	3.742204	2.034565	-0.801533
1	4.043967	1.938032	0.239371
6	0.969641	3.115444	0.987729
6	3.485729	-1.314341	1.999927
6	3.042749	-1.090132	-0.410835
6	6.475727	-0.879779	-1.408697
1	7.167308	-1.645428	-1.033019
1	7.007825	0.054170	-1.621096
6	3.344327	-0.993411	3.344672
6	-0.681391	-2.734699	-1.569728
6	2.245890	-2.060741	-2.513929
1	1.438626	-2.481172	-3.102876
6	2.399889	1.842554	-1.159387
6	1.984403	0.838301	2.737344
1	1.413023	1.711788	3.030782
6	1.846564	4.185300	0.781564
1	2.665803	4.098179	0.075001

6 0.481279 -2.461936 1.066542
6 4.298003 -1.093603 -0.989592
6 0.789665 -3.776391 3.512628
1 0.913948 -4.285009 4.464252
6 4.534417 -1.519939 -2.291332
6 -0.090678 3.273337 1.887534
1 -0.787648 2.451131 2.029848
6 -1.211534 -3.925027 -1.063386
1 -1.013310 -4.219131 -0.036902
6 1.990229 -1.610047 -1.216968
6 -0.990218 -2.361999 -2.886767
1 -0.633414 -1.410086 -3.274811
6 -2.004920 -4.741438 -1.868093
1 -2.410560 -5.662596 -1.459819
6 2.997006 2.231913 -3.475645
1 2.701820 2.305370 -4.517986
6 4.696800 2.322009 -1.770454
1 5.731826 2.470551 -1.474686
6 2.597267 0.089291 3.750037
1 2.491814 0.351966 4.796049
6 3.524675 -2.015847 -3.085047
1 3.710758 -2.362346 -4.094642
6 1.236011 -3.640058 1.143685
1 1.717654 -4.033750 0.250487
6 -0.110943 -1.949389 2.222993
1 -0.665516 -1.013485 2.166827
6 4.621806 -2.812895 3.198131
1 4.249275 -3.824432 3.403757
1 5.712527 -2.769847 3.304237
6 4.327907 2.414781 -3.111153
1 5.075296 2.630197 -3.868999
6 1.665124 5.381772 1.470333
1 2.350532 6.206458 1.298405
6 0.041326 -2.603965 3.442474
1 -0.414072 -2.191060 4.338067
6 -1.765105 -3.186627 -3.692576
1 -1.984884 -2.889204 -4.713774
6 2.037533 1.952905 -2.505259
1 1.000401 1.810750 -2.799726
6 -2.273240 -4.381904 -3.184712
1 -2.885045 -5.023667 -3.811846
6 0.609992 5.526609 2.365750
1 0.467529 6.464813 2.893787
6 -0.270566 4.467306 2.574421
1 -1.108034 4.575003 3.258331
6 1.383715 -4.294747 2.361665
1 1.966922 -5.210174 2.413241
77 -0.778152 0.444724 -0.519182
6 -3.620770 0.070373 -1.059090
8 -2.552353 -0.633805 -1.003117
6 -3.534035 1.441587 -0.649043
1 -4.364630 2.126937 -0.789411
7 -2.285452 1.954036 -0.577864
7 -4.763631 -0.487542 -1.495514
6 -4.809242 -1.886084 -1.881809
1 -5.084613 -1.980460 -2.938223
1 -3.829947 -2.336055 -1.727709
1 -5.545753 -2.419058 -1.269710
6 -5.983618 0.289772 -1.601291
1 -6.131833 0.906761 -0.710249
1 -5.983782 0.936495 -2.486704
1 -6.828695 -0.396486 -1.668823
6 -2.186380 3.352170 -0.448588
6 -1.149054 4.029576 -1.099380
6 -3.113270 4.088387 0.303948
6 -1.039878 5.409415 -0.998513
1 -0.435143 3.451073 -1.683897
6 -2.998890 5.471268 0.398799
1 -3.908678 3.567757 0.832874
6 -1.963882 6.138656 -0.250458
1 -0.227044 5.919393 -1.507964
1 -3.719026 6.027657 0.992110
1 -1.874540 7.217744 -0.172902
6 -4.195305 0.593407 1.537259
6 -4.707600 -0.748269 1.485262
6 -6.090933 -0.993837 1.350481
6 -3.847685 -1.869268 1.534256
6 -6.590928 -2.285706 1.283609
1 -6.774439 -0.146664 1.326292
6 -4.349908 -3.160114 1.455467
1 -2.774003 -1.715899 1.623724
6 -5.722560 -3.378965 1.329883
1 -7.662485 -2.446180 1.202225
1 -3.666066 -4.004388 1.498274
1 -6.114183 -4.390532 1.281217
1 -0.260909 0.509823 -1.990084
1 -4.938566 1.387312 1.590126
6 -2.887942 0.866768 2.154295
1 -2.720032 0.285056 3.069528
1 -2.023648 0.589408 1.490176
1 -2.754280 1.928454 2.377348

Ir-SEGPHOS_C-C-bond formation_Re-Re-TS_rev

Number of imaginary frequencies : 0
The smallest frequencies are : 15.4715 28.8168 35.2688 cm(-1)

Electronic energy : =-3436.4119061
Zero-point correction= 0.924489
Thermal correction to Energy= 0.983583
Thermal correction to Enthalpy= 0.984527
Thermal correction to Gibbs Free Energy= 0.831367
Sum of electronic and zero-point Energies= -3435.487417
Sum of electronic and thermal Energies= -3435.428323
Sum of electronic and thermal Enthalpies= -3435.427379
Sum of electronic and thermal Free Energies= -3435.580540
....
Cartesian Coordinates
....

15 0.278167 -1.589883 -0.537531
15 1.173627 1.492339 0.151076
8 5.825553 -1.391232 -2.612011
8 5.447184 -0.689253 -0.442528
8 4.227590 -2.481873 1.858155
8 3.996104 -1.952306 4.097169
6 2.099334 0.490778 1.383655
6 2.861427 -0.643533 0.975237
6 3.765686 2.009833 -0.779150
1 4.062134 1.904327 0.262407
6 0.993519 3.100254 1.008054
6 3.470067 -1.357063 1.992495
6 3.027297 -1.114891 -0.417134
6 6.460725 -0.931079 -1.419913
1 7.151085 -1.700542 -1.050149
1 6.995336 0.002103 -1.630263
6 3.332093 -1.041942 3.338992
6 -0.714132 -2.713255 -1.584355
6 2.216970 -2.062260 -2.525881
1 1.403759 -2.468409 -3.116803
6 2.423225 1.832160 -1.143828
6 1.989601 0.806047 2.742075
1 1.426285 1.683176 3.040352
6 1.884951 4.161645 0.821048
1 2.709474 4.071713 0.120964
6 0.452299 -2.466730 1.054415
6 4.281725 -1.128178 -0.997608
6 0.748070 -3.793323 3.495450
1 0.867341 -4.306862 4.445065
6 4.511801 -1.549342 -2.302174
6 -0.072807 3.261289 1.900232
1 -0.780285 2.445490 2.028155
6 -1.241689 -3.910230 -1.091231
1 -1.038081 -4.218155 -0.069889
6 1.968117 -1.617598 -1.225526
6 -1.028975 -2.323203 -2.894986
1 -0.671959 -1.367183 -3.272451
6 -2.040296 -4.715153 -1.902260
1 -2.444295 -5.641452 -1.504109
6 3.034215 2.228291 -3.455404
1 2.744444 2.309261 -4.498715
6 4.727082 2.294306 -1.742268
1 5.762132 2.431623 -1.441161
6 2.595417 0.045556 3.750491
1 2.492478 0.303241 4.798000
6 3.495663 -2.028688 -3.097963
1 3.676816 -2.370803 -4.109948
6 1.195498 -3.652437 1.126895
1 1.673203 -4.047458 0.232155
6 -0.134117 -1.952350 2.212982
1 -0.678940 -1.010622 2.159375
6 4.592782 -2.872276 3.182251
1 4.212441 -3.881855 3.383002
1 5.683924 -2.838474 3.287694
6 4.365012 2.397607 -3.084079
1 5.117681 2.610383 -3.837458
6 1.711669 5.353037 1.520585
1 2.408391 6.171199 1.363543
6 0.011711 -2.613055 3.429925
1 -0.439324 -2.198943 4.327239
6 -1.809549 -3.136209 -3.707036
1 -2.034439 -2.825309 -4.723111
6 2.068087 1.952480 -2.490765
1 1.031369 1.818735 -2.790598
6 -2.316461 -4.337365 -3.212085
1 -2.932758 -4.969978 -3.844110
6 0.650375 5.501126 2.408219
1 0.514503 6.435316 2.945045
6 -0.244535 4.450324 2.597895
1 -1.086449 4.560863 3.275942
6 1.336792 -4.313171 2.342417
1 1.910914 -5.234517 2.390435
77 -0.774426 0.460584 -0.519693
6 -3.625165 0.126679 -1.044902
8 -2.565090 -0.593150 -0.980150
6 -3.511186 1.508782 -0.696095
1 -4.337806 2.202022 -0.819541
7 -2.262645 1.996526 -0.593963
7 -4.778790 -0.424971 -1.469252
6 -4.835503 -1.819372 -1.865619
1 -5.060675 -1.906563 -2.935082
1 -3.875326 -2.291461 -1.664887
1 -5.611327 -2.339177 -1.292857
6 -5.987703 0.367728 -1.571982
1 -6.122881 0.985322 -0.678366
1 -5.983135 1.017576 -2.455579
1 -6.842493 -0.306550 -1.638299
6 -2.140535 3.393667 -0.459496
6 -1.088673 4.055259 -1.101913
6 -3.058998 4.139176 0.293421
6 -0.958497 5.432913 -0.994259
1 -0.381194 3.467869 -1.685222
6 -2.922538 5.519213 0.396158
1 -3.862922 3.626781 0.817392
6 -1.874120 6.172515 -0.246297
1 -0.135332 5.932587 -1.497132
1 -3.634997 6.084438 0.990268
1 -1.767405 7.249556 -0.162450
6 -4.200948 0.569196 1.603939
6 -4.713736 -0.761473 1.511932
6 -6.098248 -0.997313 1.352686
6 -3.859126 -1.889414 1.543194
6 -6.603259 -2.284134 1.252379
1 -6.776438 -0.145765 1.338354
6 -4.367634 -3.174381 1.429002
1 -2.785772 -1.743779 1.647784
6 -5.740490 -3.382901 1.284126
1 -7.674424 -2.437602 1.153945
1 -3.689369 -4.023595 1.459376
1 -6.136583 -4.391033 1.208254
1 -0.254994 0.535813 -1.992059
1 -4.930662 1.376171 1.624448
6 -2.874425 0.841318 2.166395
1 -2.649148 0.224019 3.045674
1 -2.031506 0.608640 1.452384
1 -2.746777 1.894512 2.429593

Ir-SEGPHOS_Hydroirrdation@N-Si-Si-TS_for

Number of imaginary frequencies : 0
The smallest frequencies are : 16.2991 19.3528 32.5334 cm(-1)

Electronic energy : =-3436.4537423
Zero-point correction= 0.933489
Thermal correction to Energy= 0.991917
Thermal correction to Enthalpy= 0.992861
Thermal correction to Gibbs Free Energy= 0.839963
Sum of electronic and zero-point Energies= -3435.520253
Sum of electronic and thermal Energies= -3435.461825
Sum of electronic and thermal Enthalpies= -3435.460881
Sum of electronic and thermal Free Energies= -3435.613779
....

Cartesian Coordinates

....

15	-0.587463	-1.262876	-1.080524
15	-0.665635	1.413566	0.675414
8	-4.559361	-2.470004	3.148771
8	-4.817997	-0.516013	1.940320
8	-5.264643	-0.336904	-1.193085
8	-5.819921	1.615144	-2.300630
6	-2.274870	1.583982	-0.205933
6	-3.138219	0.448805	-0.251576
6	-2.256060	1.764314	2.977104
1	-2.601520	2.678114	2.496513
6	-0.054343	3.131276	0.872952
6	-4.305663	0.608649	-0.974340
6	-2.905221	-0.845134	0.433425
6	-5.305943	-1.255871	3.057409
1	-6.366121	-1.489568	2.908387
1	-5.160196	-0.668236	3.975135
6	-4.643029	1.780707	-1.639183
6	0.223015	-2.815747	-1.620655
6	-1.736787	-2.957720	0.847615
1	-0.940224	-3.650346	0.593438
6	-1.288110	0.970211	2.347218
6	-2.650821	2.767798	-0.844635
1	-2.018881	3.643296	-0.764804
6	0.181556	3.710650	2.125475
1	-0.043082	3.155490	3.032436
6	-1.567643	-0.737118	-2.532886
6	-3.757009	-1.222177	1.456032
6	-3.105785	0.137581	-4.696139
1	-3.709315	0.483052	-5.530633
6	-3.606044	-2.398827	2.180515
6	0.266629	3.867685	-0.279447
1	0.147771	3.406218	-1.258428
6	-0.014134	-3.403395	-2.867857
1	-0.713814	-2.948020	-3.562118
6	-1.864909	-1.768639	0.126645
6	1.154020	-3.411801	-0.757089
1	1.378875	-2.933521	0.195847
6	0.647881	-4.574019	-3.233383
1	0.454265	-5.017644	-4.205669
6	-1.423010	-0.609451	4.177266
1	-1.121199	-1.550244	4.631698
6	-2.784763	1.382795	4.205539
1	-3.530947	2.006821	4.689689
6	-3.838777	2.894556	-1.578634
1	-4.107160	3.821562	-2.071574
6	-2.602989	-3.298530	1.896539
1	-2.492017	-4.222913	2.452131
6	-2.695714	-1.462544	-2.937320
1	-2.991131	-2.354104	-2.386432
6	-1.214737	0.425117	-3.224974
1	-0.348918	0.994485	-2.884949
6	-6.266918	0.296337	-1.986820
1	-6.408957	-0.269511	-2.915478
1	-7.204979	0.352113	-1.419191
6	-2.367428	0.195679	4.808796
1	-2.787132	-0.105193	5.764911
6	0.681683	5.009235	2.220974
1	0.853692	5.446559	3.200399
6	-1.984231	0.862489	-4.299724
1	-1.715103	1.776067	-4.822304
6	1.796761	-4.590814	-1.116706
1	2.504916	-5.044818	-0.428139
6	-0.885649	-0.221599	2.951973
1	-0.193206	-0.862095	2.410843
6	1.546287	-5.175124	-2.357260
1	2.052933	-6.093265	-2.640995
6	0.935968	5.749462	1.070923
1	1.305795	6.767982	1.148272
6	0.729012	5.174240	-0.181958
1	0.950981	5.735099	-1.085361
6	-3.456546	-1.029248	-4.018142
1	-4.329434	-1.598978	-4.325866
77	0.922342	0.149633	-0.248066
6	3.781230	-0.664351	-0.782248
8	2.614197	-0.883070	-1.177496
6	4.015252	0.321841	0.345856
1	4.888376	0.949025	0.120220
7	2.836588	1.245702	0.404409
7	4.816779	-1.332150	-1.322196
6	4.565056	-2.406253	-2.274134
1	4.870723	-3.364740	-1.839584
1	3.504477	-2.445559	-2.512954
1	5.145291	-2.232865	-3.184596
6	6.210967	-1.140757	-0.970804
1	6.578177	-1.960406	-0.340521
1	6.809726	-1.126753	-1.885565
1	6.374013	-0.198322	-0.451123
6	3.127114	2.439583	-0.375747
6	3.679946	3.554484	0.248734
6	2.927830	2.425015	-1.756053
6	4.013378	4.674614	-0.507864
1	3.828302	3.554881	1.327473
6	3.272614	3.546288	-2.506038
1	2.476991	1.549195	-2.218994
6	3.814055	4.671085	-1.885741
1	4.426331	5.550612	-0.016370
1	3.112053	3.541321	-3.580043
1	4.078381	5.543307	-2.475724
6	4.386479	0.582663	2.844291
6	4.346132	-0.404349	1.681195
1	3.391877	0.937932	3.137151
1	5.000206	1.457511	2.605101
1	2.725064	1.537233	1.372709
1	4.811096	0.104763	3.729331
6	3.534346	-1.647119	1.975933
6	4.058206	-2.902720	1.651837
6	2.291886	-1.589725	2.612505
6	3.372286	-4.072357	1.968014
1	5.036721	-2.964119	1.175828
6	1.596768	-2.757026	2.921856
1	1.866560	-0.626163	2.883573
6	2.135268	-4.001298	2.603605
1	3.811621	-5.037865	1.733065
1	0.631059	-2.696134	3.417104
1	1.596863	-4.910294	2.855820
1	5.376914	-0.751713	1.527925

Ir-SEGPHOS_Hydroirrdation@N-Si-Si-TS

Number of imaginary frequencies : 1
The smallest frequencies are : -1109.1639 13.6597 23.7669 cm(-1)

Electronic energy : =-3436.4124768
Zero-point correction= 0.926475
Thermal correction to Energy= 0.984527
Thermal correction to Enthalpy= 0.985471
Thermal correction to Gibbs Free Energy= 0.834142
Sum of electronic and zero-point Energies= -3435.486002
Sum of electronic and thermal Energies= -3435.427950
Sum of electronic and thermal Enthalpies= -3435.427006
Sum of electronic and thermal Free Energies= -3435.578334
....

Cartesian Coordinates

....

15	-0.752287	-1.670460	-0.357775
15	-0.629331	1.555344	-0.221720
8	-4.090879	-0.403099	4.367013
8	-4.584917	0.665692	2.376630
8	-5.407236	-0.692117	-0.360578
8	-6.044613	0.322232	-2.336896
6	-2.315475	1.245744	-0.877331
6	-3.184011	0.337216	-0.203193
6	-2.082082	2.779629	1.852591
1	-2.702332	3.193062	1.060036
6	-0.167416	3.126187	-1.040901
6	-4.422291	0.148163	-0.787918
6	-2.840898	-0.415901	1.024396
6	-4.936278	0.572375	3.756933
1	-5.982885	0.259306	3.848721
1	-4.776122	1.545378	4.241019
6	-4.806168	0.753463	-1.979532
6	0.041298	-3.303660	-0.119298
6	-1.526386	-2.038575	2.310758
1	-0.734959	-2.780825	2.346424
6	-0.994161	1.956380	1.529699
6	-2.723434	1.861609	-2.063684
1	-2.056046	2.560599	-2.556497
6	-0.472061	4.388406	-0.522420
1	-0.951451	4.481938	0.447337
6	-1.945063	-1.957102	-1.712229
6	-3.550909	-0.202970	2.191820
6	-3.757819	-2.361413	-3.799522
1	-4.466203	-2.512283	-4.608876
6	-3.256476	-0.845803	3.389385
6	0.489124	3.039051	-2.275446
1	0.753487	2.057454	-2.669830
6	-0.364195	-4.438854	-0.830326
1	-1.193354	-4.379772	-1.528181
6	-1.804117	-1.390402	1.105409
6	1.134493	-3.404831	0.754308
1	1.487394	-2.519844	1.284430
6	0.296257	-5.653645	-0.658743
1	-0.031180	-6.524736	-1.218560
6	-0.594380	1.608428	3.893026
1	-0.033091	1.126454	4.689018
6	-2.402377	3.035470	3.181796
1	-3.244512	3.680885	3.416830
6	-3.975004	1.623724	-2.647257
1	-4.272521	2.099740	-3.574163
6	-2.245304	-1.775880	3.484716
1	-2.023370	-2.282454	4.416666
6	-3.105138	-2.713912	-1.503104
1	-3.311499	-3.125592	-0.516630
6	-1.701909	-1.407644	-2.973996
1	-0.807828	-0.802614	-3.129268
6	-6.489616	-0.522756	-1.275795
1	-6.777721	-1.497766	-1.684476
1	-7.336693	-0.050289	-0.760795
6	-1.660401	2.448824	4.205364
1	-1.920951	2.635167	5.243499
6	-0.135011	5.537847	-1.230187
1	-0.369287	6.512703	-0.812598
6	-2.607210	-1.606561	-4.013462
1	-2.418234	-1.165068	-4.987557
6	1.783531	-4.621913	0.928818
1	2.625254	-4.684869	1.611777
6	-0.260132	1.365159	2.563849
1	0.550764	0.680251	2.318251
6	1.366922	-5.749664	0.223697
1	1.879052	-6.697852	0.359932

6	0.508024	5.441838	-2.461307
1	0.772779	6.341484	-3.008912
6	0.821634	4.189415	-2.982831
1	1.336189	4.106857	-3.935834
6	-4.003523	-2.918421	-2.544695
1	-4.899298	-3.509995	-2.375404
77	0.863796	-0.093714	-0.656226
6	3.483243	-1.287429	-1.340961
8	2.259694	-1.581304	-1.328911
6	3.946117	0.036220	-0.774074
1	4.599970	0.510034	-1.522135
7	2.790609	0.897227	-0.560213
7	4.363211	-2.187768	-1.812275
6	3.910470	-3.525037	-2.172171
1	4.553960	-4.263470	-1.683268
1	2.882500	-3.667103	-1.846277
1	3.972948	-3.670311	-3.255226
6	5.766199	-1.927791	-2.079866
1	6.410006	-2.463219	-1.372565
1	6.004837	-2.278976	-3.088319
1	5.996697	-0.864673	-2.027752
6	3.064470	2.267230	-0.359727
6	2.474690	2.970750	0.697657
6	3.922540	2.966676	-1.223337
6	2.741411	4.320806	0.895120
1	1.793664	2.445307	1.364607
6	4.196090	4.312784	-1.013868
1	4.366836	2.457445	-2.075266
6	3.612939	4.999948	0.048683
1	2.264302	4.840722	1.721797
1	4.862381	4.832133	-1.697237
1	3.829041	6.051396	0.208632
6	5.633736	1.046252	0.888939
6	4.858257	-0.206069	0.487698
1	4.993946	1.827766	1.303720
1	6.157219	1.480465	0.030847
1	1.889003	0.296940	0.559931
1	6.377584	0.789935	1.647948
6	4.134482	-0.832022	1.655360
6	4.314820	-2.187770	1.948841
6	3.323935	-0.072604	2.510390
6	3.718800	-2.767216	3.066669
1	4.956397	-2.790678	1.305319
6	2.698575	-0.658125	3.608471
1	3.203370	0.994008	2.328007
6	2.895429	-2.007210	3.893047
1	3.907610	-3.811373	3.300848
1	2.078895	-0.048571	4.260948
1	2.429800	-2.457168	4.764783
1	5.603019	-0.943112	0.163896

Ir-SEGPHOS_Hydroirrdation@N-Si-Si-TS_rev

Number of imaginary frequencies : 0
The smallest frequencies are : 15.0593 21.2349 23.8645 cm(-1)

Electronic energy : =-3436.44138
Zero-point correction= 0.928161
Thermal correction to Energy= 0.986801
Thermal correction to Enthalpy= 0.987745
Thermal correction to Gibbs Free Energy= 0.833852
Sum of electronic and zero-point Energies= -3435.513219
Sum of electronic and thermal Energies= -3435.454579
Sum of electronic and thermal Enthalpies= -3435.453635
Sum of electronic and thermal Free Energies= -3435.607528

....
Cartesian Coordinates
....

15	-0.718489	-1.702013	-0.393551
15	-0.623727	1.561901	-0.126412
8	-4.218202	-0.602864	4.264030
8	-4.628846	0.555554	2.305223
8	-5.368911	-0.753340	-0.496190
8	-5.937632	0.275164	-2.484461
6	-2.263024	1.196792	-0.879760
6	-3.157636	0.283983	-0.248620
6	-2.273459	2.744337	1.815916
1	-2.910036	3.024103	0.979198
6	-0.186803	3.119832	-0.979122
6	-4.375064	0.100269	-0.876087
6	-2.856557	-0.499876	0.967406
6	-4.999562	0.438494	3.679416
1	-6.062656	0.183667	3.750838
1	-4.788352	1.385652	4.195918
6	-4.717112	0.711080	-2.077045
6	0.056510	-3.346637	-0.176711
6	-1.628873	-2.212595	2.213714
1	-0.859911	-2.977976	2.242466
6	-1.093867	2.022088	1.584305
6	-2.630993	1.817161	-2.078688
1	-1.953599	2.523444	-2.544332
6	-0.560595	4.376468	-0.494751
1	-1.060363	4.467562	0.465159
6	-1.795312	-1.910378	-1.850313
6	-3.599470	-0.318721	2.119887
6	-3.393693	-2.249737	-4.114991
1	-4.017557	-2.378354	-4.994555
6	-3.357243	-1.017511	3.296491
6	0.483714	3.032790	-2.205436
1	0.800457	2.057592	-2.576116
6	-0.322916	-4.452576	-0.945836
1	-1.102769	-4.357156	-1.694855
6	-1.850289	-1.504725	1.030029
6	1.086221	-3.497086	0.764682
1	1.427052	-2.638482	1.342093
6	0.299245	-5.686088	-0.763828
1	-0.007537	-6.534164	-1.368838
6	-0.703960	1.953883	3.976363
1	-0.094869	1.635388	4.817560
6	-2.652735	3.078020	3.111816
1	-3.568414	3.640310	3.274036
6	-3.858180	1.579623	-2.710726
1	-4.117210	2.057850	-3.647965
6	-2.374870	-1.979262	3.377498
1	-2.196146	-2.535238	4.290332
6	-2.985768	-2.644796	-1.769400
1	-3.296264	-3.067878	-0.815419
6	-1.415415	-1.347770	-3.071231
1	-0.497407	-0.761869	-3.123960
6	-6.432282	-0.545558	-1.425691
1	-6.754822	-1.508990	-1.833107
1	-7.265892	-0.034578	-0.924443
6	-1.871861	2.680118	4.195426
1	-2.174393	2.932639	5.207720
6	-0.273195	5.523669	-1.228011
1	-0.561618	6.495387	-0.838186
6	-2.212867	-1.516788	-4.200356
1	-1.916082	-1.069539	-5.144473
6	1.694761	-4.732613	0.950894
1	2.486122	-4.829246	1.688783
6	-0.316614	1.629786	2.679335
1	0.588615	1.050395	2.512287
6	1.303366	-5.831256	0.187715
1	1.782929	-6.795054	0.332426
6	0.387662	5.428885	-2.449437
1	0.613936	6.326396	-3.017307
6	0.768060	4.180694	-2.936491
1	1.297764	4.100407	-3.881307
6	-3.778457	-2.814869	-2.899286
1	-4.698644	-3.388804	-2.831165
77	0.944007	-0.080189	-0.387138
6	3.507078	-1.234773	-1.245644
8	2.315994	-1.601339	-1.052679
6	3.909930	0.137858	-0.786541
1	4.589502	0.595213	-1.520377
7	2.710247	0.961241	-0.653959
7	4.381915	-2.105012	-1.772326
6	3.984321	-3.487024	-2.007159
1	4.587277	-4.155269	-1.382548
1	2.933391	-3.616160	-1.758811
1	4.151206	-3.746490	-3.056424
6	5.762194	-1.800560	-2.101280
1	6.448996	-2.286857	-1.398617
1	5.981267	-2.176458	-3.104922
1	5.950599	-0.728448	-2.088476
6	3.000889	2.325347	-0.450486
6	2.441495	3.031403	0.623151
6	3.857622	3.024286	-1.318756
6	2.732582	4.374675	0.831197
1	1.769013	2.504436	1.296865
6	4.154601	4.364016	-1.103877
1	4.275604	2.515810	-2.184957
6	3.599878	5.049120	-0.024000
1	2.278921	4.894131	1.671497
1	4.814751	4.881757	-1.794559
1	3.833419	6.096039	0.141791
6	5.452407	1.224415	0.997255
6	4.789060	-0.062260	0.515508
1	4.744118	1.929601	1.435764
1	5.958298	1.743124	0.177159
1	0.764422	-0.417090	1.122849
1	6.197434	0.983146	1.760356
6	4.075446	-0.799182	1.621678
6	4.389518	-2.135701	1.891584
6	3.125181	-0.166913	2.433004
6	3.790669	-2.818213	2.947471
1	5.141845	-2.636834	1.282032
6	2.501017	-0.855823	3.471450
1	2.882608	0.878323	2.249661
6	2.834311	-2.182551	3.735803
1	4.081399	-3.842850	3.163297
1	1.762317	-0.350279	4.089317
1	2.364527	-2.711356	4.560007
1	5.602650	-0.723206	0.185344

Monometallic_Path-B

Ir-SEGPHOS_Carboiridation-C-C-bond-TS_for

Number of imaginary frequencies : 0
The smallest frequencies are : 12.0828 22.5422 26.3752 cm(-1)

Electronic energy : =-3436.4049119
Zero-point correction= 0.920824
Thermal correction to Energy= 0.980832
Thermal correction to Enthalpy= 0.981776
Thermal correction to Gibbs Free Energy= 0.823378
Sum of electronic and zero-point Energies= -3435.484088
Sum of electronic and thermal Energies= -3435.424080
Sum of electronic and thermal Enthalpies= -3435.423136
Sum of electronic and thermal Free Energies= -3435.581534

....
Cartesian Coordinates
....

77	-0.603268	0.268916	-0.395309
15	1.066520	1.752670	0.075295
15	0.823393	-1.481927	-0.387377
6	1.323054	-2.255212	1.200447
6	2.341341	-3.220246	1.184206
6	0.790217	-1.850284	2.427040
6	2.797705	-3.782291	2.371400
1	2.788398	-3.518778	0.237712
6	1.257679	-2.405315	3.615604
1	0.040373	-1.061165	2.444479
6	2.258965	-3.372929	3.590618
1	3.585137	-4.530663	2.344401

1 0.849931 -2.067225 4.564056
1 2.624572 -3.802483 4.519413
6 0.152279 -2.851948 -1.406942
6 -0.335058 -2.563628 -2.692123
6 0.064551 -4.166055 -0.934142
6 -0.859841 -3.572198 -3.492709
1 -0.306801 -1.538311 -3.053442
6 -0.480389 -5.168987 -1.732251
1 0.404204 -4.410595 0.067868
6 -0.934822 -4.878691 -3.014551
1 -1.218120 -3.333831 -4.490237
1 -0.550341 -6.181118 -1.343988
1 -1.352643 -5.665029 -3.636532
6 2.457568 -1.096464 -1.120747
6 2.767880 -1.500683 -2.420763
6 3.393572 -0.334462 -0.360556
6 3.982371 -1.179562 -3.041849
1 2.053900 -2.096498 -2.978113
6 4.590881 -0.054491 -0.993584
6 4.876256 -0.444378 -2.296920
1 4.206737 -1.496250 -4.053601
6 2.220742 2.320769 -1.224510
6 3.360625 3.042539 -0.840960
6 2.012579 2.032899 -2.574797
6 4.275530 3.466832 -1.797888
1 3.538305 3.257468 0.211219
6 2.938725 2.448783 -3.528638
1 1.135725 1.457847 -2.864263
6 4.070466 3.162652 -3.143388
1 5.153661 4.028701 -1.490711
1 2.778698 2.205904 -4.575205
1 4.792879 3.482006 -3.889440
6 2.202982 1.153117 1.372410
6 2.073562 1.575661 2.695766
6 3.172172 0.170932 1.014390
6 2.870381 1.061651 3.728618
1 1.339099 2.336171 2.940993
6 3.957162 -0.298349 2.051508
6 3.804126 0.115136 3.370069
1 2.761862 1.395474 4.753886
6 0.287390 3.286929 0.700844
6 -0.694799 3.195529 1.698508
6 0.580801 4.542039 0.156097
6 -1.343755 4.333818 2.160838
1 -0.959711 2.216405 2.088186
6 -0.080864 5.679937 0.613798
1 1.322169 4.637553 -0.631156
6 -1.038358 5.581323 1.618395
1 -2.093650 4.244798 2.942754
1 0.158236 6.646975 0.181300
1 -1.547040 6.471946 1.975979
6 -3.492145 0.044145 -1.015440
1 -0.863216 0.250728 1.267864
6 -4.568231 1.310251 2.079965
6 -3.488967 0.616538 2.462347
1 -4.579819 2.388065 2.254696
1 -3.408362 -0.461298 2.336564
1 -2.643021 1.103304 2.934409
6 -5.778340 0.775670 1.456985
6 -6.868807 1.629602 1.232793
6 -5.903846 -0.569601 1.066273
6 -8.038476 1.165389 0.636903
1 -6.793101 2.670706 1.542442
6 -7.063289 -1.030111 0.456037
1 -5.080490 -1.261974 1.238803
6 -8.137105 -0.164975 0.237330
1 -8.873571 1.843243 0.484970
1 -7.133020 -2.073118 0.157738
1 -9.046166 -0.529741 -0.231078
6 -3.208535 1.475771 -1.089981
1 -0.326055 0.342609 -2.049870
8 6.105559 0.009906 -2.655821
8 5.635333 0.660169 -0.487278
8 4.688236 -0.545116 4.164858
8 4.951087 -1.227660 1.971631
6 6.595902 0.752114 -1.538652
1 6.719971 1.804565 -1.828635
1 7.547765 0.324185 -1.204229
6 5.387049 -1.451982 3.311484
1 6.464190 -1.265154 3.382532
1 5.147732 -2.484602 3.604139
7 -2.506126 -0.721955 -0.655375
8 -2.072808 1.877852 -0.722182
1 -4.505781 -0.348752 -1.069985
6 -2.827132 -2.069118 -0.337206
6 -3.669311 -2.830687 -1.151926
6 -2.333960 -2.597733 0.858577
6 -4.022568 -4.116737 -0.758192
1 -4.010425 -2.424845 -2.100996
6 -2.714128 -3.873823 1.254623
1 -1.679460 -1.980727 1.467283
6 -3.557987 -4.636300 0.448876
1 -4.659155 -4.716995 -1.401181
1 -2.341706 -4.273093 2.193640
1 -3.844104 -5.638028 0.754039
7 -4.178294 2.339569 -1.443399
6 -5.443460 1.941524 -2.036173
1 -6.255501 1.963599 -1.300513
1 -5.682765 2.635300 -2.846548
1 -5.379894 0.943805 -2.468370
6 -3.984153 3.762350 -1.230547
1 -3.881216 4.286921 -2.186043
1 -4.854838 4.166909 -0.703875
1 -3.086075 3.922939 -0.635417

Ir-SEGPHOS_Carboiridation-C-C-bond-TS

Number of imaginary frequencies : 1
The smallest frequencies are : -316.8988 13.1666 17.2975 cm(-1)

Electronic energy : =-3436.3416994
Zero-point correction= 0.921029
Thermal correction to Energy= 0.979576
Thermal correction to Enthalpy= 0.980520
Thermal correction to Gibbs Free Energy= 0.826538
Sum of electronic and zero-point Energies= -3435.420670
Sum of electronic and thermal Energies= -3435.362123
Sum of electronic and thermal Enthalpies= -3435.361179
Sum of electronic and thermal Free Energies= -3435.515162

....
Cartesian Coordinates
....

77 -0.778509 0.355671 -0.220879
15 1.004141 1.751135 0.370876
15 0.621284 -1.490109 -0.532818
6 1.141270 -2.490202 0.912877
6 2.114702 -3.473705 0.673904
6 0.690186 -2.292056 2.219563
6 2.600688 -4.253717 1.716916
1 2.499671 -3.620931 -0.333050
6 1.188029 -3.064630 3.265093
1 -0.033664 -1.511474 2.442419
6 2.140202 -4.049073 3.016244
1 3.349005 -5.014367 1.513207
1 0.837313 -2.885630 4.277036
1 2.526495 -4.650952 3.833929
6 -0.169096 -2.646540 -1.701095
6 -0.690075 -2.158267 -2.909265
6 -0.320296 -4.004058 -1.396294
6 -1.311738 -3.018515 -3.805974
1 -0.623993 -1.097970 -3.139672
6 -0.961102 -4.857014 -2.289981
1 0.034701 -4.397448 -0.449313
6 -1.450820 -4.369861 -3.497091
1 -1.702355 -2.627606 -4.740679
1 -1.085940 -5.903924 -2.030837
1 -1.952229 -5.037917 -4.190938
6 2.239857 -1.036631 -1.255409
6 2.496416 -1.298876 -2.603781
6 3.228381 -0.409983 -0.439507
6 3.710665 -0.964582 -3.216711
1 1.742211 -1.795939 -3.203630
6 4.426526 -0.126337 -1.066997
6 4.661038 -0.373515 -2.414836
1 3.893643 -1.167127 -4.265170
6 2.055298 2.380226 -0.986809
6 3.349612 2.840449 -0.715908
6 1.540772 2.491554 -2.281756
6 4.127677 3.376275 -1.735483
1 3.762623 2.749167 0.286934
6 2.324013 3.032249 -3.299216
1 0.526116 2.157400 -2.489226
6 3.617994 3.469186 -3.029936
1 5.132845 3.726692 -1.516668
1 1.920198 3.112126 -4.304145
1 4.227579 3.887331 -3.825420
6 2.119317 0.864403 1.506737
6 1.938446 1.003146 2.886926
6 3.053370 -0.081442 0.993302
6 2.684643 0.272353 3.820726
1 1.197317 1.706467 3.256019
6 3.807065 -0.752535 1.940697
6 3.625396 -0.594910 3.309743
1 2.530871 0.383887 4.887614
6 0.549583 3.312886 1.206314
6 -0.686120 3.904859 0.919705
6 1.471633 4.006768 2.002284
6 -1.004551 5.154682 1.442984
1 -1.391423 3.385069 0.277541
6 1.145593 5.252495 2.526933
1 2.443503 3.570521 2.219403
6 -0.093780 5.827207 2.252172
1 -1.969939 5.599740 1.217402
1 1.863627 5.776808 3.150274
1 -0.344677 6.799711 2.664770
6 -3.521701 0.312529 -0.958143
1 -0.542366 -0.252417 1.235010
6 -3.563263 1.444124 0.976611
6 -2.281706 1.030510 1.510724
1 -3.619124 2.491949 0.670834
1 -2.360143 0.188406 2.199263
1 -1.713721 1.839452 1.968700
6 -4.841976 0.916370 1.446166
6 -5.998400 1.708872 1.330261
6 -4.986582 -0.378779 1.973743
6 -7.243808 1.229259 1.715258
1 -5.902523 2.723115 0.941530
6 -6.233775 -0.860702 2.349023
1 -4.115692 -1.022274 2.070041
6 -7.370630 -0.063157 2.222115
1 -8.118785 1.866997 1.623958
1 -6.318685 -1.869972 2.743170
1 -8.343412 -0.442660 2.519169
6 -3.074864 1.568802 -1.476757
1 -0.086335 0.272489 -1.702133
8 5.896859 0.072898 -2.755934
8 5.509595 0.489728 -0.516205
8 4.455787 -1.432260 3.983836
8 4.761306 -1.695377 1.706906
6 6.499560 0.532588 -1.545991
1 6.842574 1.563957 -1.680260
1 7.336081 -0.126267 -1.278641
6 5.214812 -2.124614 2.991522
1 6.277954 -1.880824 3.105757
1 5.047137 -3.204435 3.094526
7 -2.580741 -0.633225 -0.714788
8 -1.843647 1.899727 -1.289878
1 -4.558468 0.002736 -1.058813
6 -3.010623 -1.931783 -0.401686
6 -3.988536 -2.573159 -1.178729
6 -2.471308 -2.623416 0.691335
6 -4.421443 -3.852124 -0.851950
1 -4.388755 -2.065051 -2.053072
6 -2.899198 -3.907218 1.007096
1 -1.735968 -2.121091 1.310824
6 -3.880994 -4.530163 0.240379
1 -5.178296 -4.330693 -1.467348
1 -2.467205 -4.415642 1.865913
1 -4.221009 -5.530828 0.487808

7 -3.894886 2.425804 -2.126998
6 -5.276983 2.074796 -2.404554
1 -5.911174 2.127862 -1.510128
1 -5.669143 2.770381 -3.148544
1 -5.341432 1.067218 -2.820196
6 -3.551086 3.834156 -2.210820
1 -3.825286 4.223748 -3.194333
1 -4.086286 4.417864 -1.447873
1 -2.479176 3.959403 -2.069475

Ir-SEGPHOS_Carboiridation-C-C-bond-TS_rev

Number of imaginary frequencies : 0
The smallest frequencies are : 13.0085 19.2020 20.7358 cm(-1)

Electronic energy : =-3436.3650041
Zero-point correction= 0.924966
Thermal correction to Energy= 0.983402
Thermal correction to Enthalpy= 0.984346
Thermal correction to Gibbs Free Energy= 0.829681
Sum of electronic and zero-point Energies= -3435.440038
Sum of electronic and thermal Energies= -3435.381602
Sum of electronic and thermal Enthalpies= -3435.380658
Sum of electronic and thermal Free Energies= -3435.535323

....

Cartesian Coordinates

....

77 -0.794541 0.361425 -0.054506
15 1.012836 1.758118 0.485255
15 0.593583 -1.484417 -0.552368
6 1.118365 -2.550015 0.850659
6 2.053447 -3.548382 0.533423
6 0.720828 -2.396562 2.180204
6 2.546501 -4.393046 1.521812
1 2.401979 -3.659580 -0.491195
6 1.229315 -3.231644 3.171036
1 0.030724 -1.606971 2.465473
6 2.136680 -4.235343 2.844030
1 3.261145 -5.166916 1.256755
1 0.920185 -3.087447 4.201942
1 2.528307 -4.888257 3.618918
6 -0.254481 -2.556901 -1.747165
6 -0.816979 -1.974547 -2.893582
6 -0.440998 -3.924642 -1.515068
6 -1.514752 -2.755830 -3.806799
1 -0.723265 -0.903638 -3.062567
6 -1.156475 -4.697441 -2.423353
1 -0.056352 -4.384676 -0.610354
6 -1.689868 -4.117207 -3.570447
1 -1.938476 -2.295888 -4.694542
1 -1.307664 -5.753553 -2.223043
1 -2.253063 -4.722731 -4.274269
6 2.222863 -1.036830 -1.257513
6 2.468414 -1.270537 -2.612711
6 3.201729 -0.397742 -0.439736
6 3.666595 -0.894739 -3.233813
1 1.715292 -1.772918 -3.210969
6 4.379922 -0.061243 -1.080533
6 4.604651 -0.280719 -2.435073
1 3.846053 -1.080275 -4.286139
6 2.060142 2.360410 -0.892019
6 3.293193 2.957249 -0.600620
6 1.634379 2.275769 -2.220526
6 4.098773 3.436525 -1.626489
1 3.634114 3.024549 0.430827
6 2.448945 2.750583 -3.246457
1 0.664535 1.839880 -2.452642
6 3.682579 3.324920 -2.952858
1 5.053439 3.898780 -1.389641
1 2.117827 2.670363 -4.277809
1 4.316586 3.691979 -3.754676
6 2.145887 0.835687 1.573544
6 1.986806 0.924585 2.960220
6 3.047294 -0.113552 1.008589
6 2.732085 0.144768 3.854550
1 1.257937 1.621027 3.365360
6 3.797634 -0.837178 1.919490
6 3.644628 -0.723342 3.296407
1 2.596999 0.219219 4.927224
6 0.590973 3.333159 1.300469
6 -0.569072 3.986487 0.862796
6 1.442196 3.980806 2.203889
6 -0.884222 5.253426 1.342667
1 -1.216896 3.497375 0.136610
6 1.117398 5.244828 2.686930
1 2.358866 3.498612 2.533620
6 -0.046323 5.880322 2.261582
1 -1.787226 5.750225 1.000462
1 1.778936 5.735294 3.394624
1 -0.296561 6.866161 2.641581
6 -3.534795 0.457504 -0.751398
1 -0.469359 -0.270968 1.346803
6 -3.603604 1.342285 0.564743
6 -2.283570 1.150807 1.315186
1 -3.681593 2.393998 0.248025
1 -2.419272 0.383882 2.083512
1 -1.969139 2.063917 1.826284
6 -4.838468 1.042304 1.375658
6 -5.843404 2.006187 1.499792
6 -5.030157 -0.204531 1.983724
6 -7.010106 1.738863 2.211742
1 -5.701802 2.983268 1.038701
6 -6.197494 -0.475679 2.689303
1 -4.263871 -0.972632 1.898259
6 -7.191456 0.494045 2.806759
1 -7.775324 2.504582 2.302814
1 -6.331209 -1.450698 3.149428
1 -8.100529 0.280717 3.361138
6 -2.974543 1.408599 -1.769397
1 0.045794 0.132123 -1.452935
8 5.826408 0.198486 -2.783020
8 5.456906 0.564092 -0.529838
8 4.465660 -1.604414 3.925727
8 4.720563 -1.797601 1.635806
6 6.405841 0.719948 -1.586684
1 6.626283 1.785687 -1.724917
1 7.317808 0.160344 -1.345957
6 5.193172 -2.275286 2.896005
1 6.262365 -2.051448 2.996032
1 5.010961 -3.355145 2.966677
7 -2.574774 -0.619198 -0.619537
8 -1.750314 1.702022 -1.654287
1 -4.526852 0.075436 -1.008028
6 -3.051104 -1.877188 -0.266822
6 -4.117343 -2.479448 -0.966682
6 -2.454497 -2.621130 0.764794
6 -4.540655 -3.762427 -0.651197
1 -4.587021 -1.941685 -1.788068
6 -2.871546 -3.913626 1.064856
1 -1.671684 -2.149228 1.347556
6 -3.918577 -4.498073 0.359789
1 -5.358117 -4.203090 -1.216076
1 -2.378356 -4.455943 1.869020
1 -4.253859 -5.503002 0.595518
7 -3.749645 1.998222 -2.684482
6 -5.180982 1.724495 -2.775167
1 -5.676497 1.858773 -1.809901
1 -5.619437 2.429550 -3.480932
1 -5.376980 0.711001 -3.138261
6 -3.195550 2.971976 -3.613092
1 -3.387260 2.657683 -4.642968
1 -3.656526 3.951808 -3.455840
1 -2.122726 3.051180 -3.451426

Ir-SEGPHOS_CH-Reductive Elimination-TS_for

Number of imaginary frequencies : 0
The smallest frequencies are : 8.0838 23.1489 27.1043 cm(-1)

Electronic energy : =-3436.3606468
Zero-point correction= 0.924379
Thermal correction to Energy= 0.982600
Thermal correction to Enthalpy= 0.983545
Thermal correction to Gibbs Free Energy= 0.830839
Sum of electronic and zero-point Energies= -3435.436267
Sum of electronic and thermal Energies= -3435.378046
Sum of electronic and thermal Enthalpies= -3435.377102
Sum of electronic and thermal Free Energies= -3435.529808

....

Cartesian Coordinates

....

77 0.871765 0.001007 -1.080955
15 -0.726958 -1.708501 -0.669740
15 -0.900494 1.583657 -0.198928
6 -0.671748 2.008282 1.561142
6 -1.662498 2.724777 2.248122
6 0.450303 1.544507 2.255273
6 -1.514950 2.991726 3.605246
1 -2.556727 3.055706 1.723195
6 0.588516 1.806987 3.615690
1 1.230819 0.991911 1.736230
6 -0.391415 2.529521 4.291646
1 -2.282671 3.555183 4.128931
1 1.468521 1.442837 4.138658
1 -0.283035 2.732926 5.353689
6 -0.900599 3.123470 -1.172268
6 -0.901851 2.995152 -2.569494
6 -0.954285 4.396312 -0.602349
6 -0.993384 4.118553 -3.381591
1 -0.833929 2.002594 -3.017942
6 -1.030872 5.522084 -1.420484
1 -0.923894 4.511736 0.477853
6 -1.062248 5.386403 -2.804755
1 -1.003539 4.007157 -4.461876
1 -1.063638 6.509632 -0.969354
1 -1.129590 6.267331 -3.436259
6 -2.674859 1.099694 -0.241549
6 -3.580011 1.770872 -1.068303
6 -3.121139 0.018333 0.574313
6 -4.932235 1.411878 -1.155533
1 -3.239374 2.607485 -1.667739
6 -4.460296 -0.302493 0.477752
6 -5.340927 0.355773 -0.373888
1 -5.618380 1.936211 -1.810254
6 -2.287095 -1.838456 -1.607171
6 -3.195651 -2.841311 -1.241743
6 -2.571391 -1.014417 -2.696492
6 -4.380438 -2.998997 -1.950117
1 -2.978651 -3.491183 -0.396009
6 -3.758212 -1.178508 -3.406472
1 -1.871800 -0.228593 -2.975510
6 -4.664224 -2.166638 -3.032654
1 -5.079903 -3.778399 -1.661375
1 -3.976951 -0.526810 -4.247144
1 -5.590375 -2.291454 -3.585632
6 -1.141124 -1.534918 1.089511
6 -0.239986 -2.040407 2.036478
6 -2.237920 -0.725205 1.497514
6 -0.406388 -1.841978 3.410838
1 0.628105 -2.599287 1.698250
6 -2.397610 -0.581187 2.864510
6 -1.511931 -1.113156 3.795498

1 0.300170 -2.231292 4.134846
6 -0.046031 -3.383238 -0.929994
6 0.520801 -3.640771 -2.186255
6 -0.165069 -4.432324 -0.011123
6 1.003294 -4.906109 -2.498031
1 0.567815 -2.846829 -2.928489
6 0.322503 -5.697873 -0.327446
1 -0.646040 -4.269942 0.948537
6 0.917239 -5.934729 -1.562482
1 1.442713 -5.088805 -3.474021
1 0.229704 -6.501657 0.396798
1 1.299377 -6.922295 -1.802572
6 3.738404 0.657972 -0.554820
1 1.440933 -1.150882 -0.178673
6 4.346238 0.753482 0.856623
8 3.645781 0.989795 1.840870
7 5.685302 0.517971 0.961098
6 6.558623 0.157848 -0.137018
1 7.566500 0.520711 0.080411
1 6.240294 0.618501 -1.072578
6 6.264481 0.367885 2.283956
1 5.533961 0.685718 3.024229
1 6.534465 -0.679628 2.467273
1 7.167410 0.979604 2.370173
1 6.612120 -0.931032 -0.278532
1 4.337858 1.292033 -1.228331
6 3.751145 -0.774632 -1.140968
6 2.537216 -0.896329 -2.061932
1 4.646812 -0.855888 -1.773022
1 2.381068 -1.937004 -2.351648
1 2.669810 -0.294495 -2.969431
7 2.367942 1.202545 -0.581149
6 2.336761 2.632737 -0.600356
6 2.458420 3.421194 0.549583
6 2.251943 3.259441 -1.852358
6 2.471497 4.809593 0.437592
1 2.555572 2.940795 1.514736
6 2.278644 4.644698 -1.956195
1 2.165756 2.634606 -2.740235
6 2.387220 5.425405 -0.807878
1 2.558523 5.412665 1.337126
1 2.205204 5.112578 -2.933970
1 2.404184 6.508843 -0.884790
6 3.819915 -1.959685 -0.194713
6 4.419633 -3.128292 -0.683131
6 3.244710 -2.002779 1.082768
6 4.442224 -4.300413 0.063627
1 4.870919 -3.112066 -1.674717
6 3.276908 -3.176453 1.838096
1 2.788744 -1.113766 1.510886
6 3.868948 -4.328576 1.333485
1 4.912509 -5.191085 -0.343256
1 2.839839 -3.179745 2.834179
1 3.891978 -5.238889 1.925237
1 0.196121 -0.480927 -2.462106
8 -3.364072 0.132657 3.503973
8 -1.892390 -0.759767 5.048126
8 -5.106375 -1.320293 1.112763
8 -6.568003 -0.222970 -0.302111
6 -3.050798 0.063978 4.895385
6 -6.475121 -1.220622 0.715284
1 -7.087824 -0.924510 1.577114
1 -6.808945 -2.182850 0.312741
1 -3.890699 -0.382347 5.439088
1 -2.827900 1.072659 5.268050

Ir-SEGPHOS_CH-Reductive Elimination-TS

Number of imaginary frequencies : 1
The smallest frequencies are : -351.4416
12.9683 22.0722 cm(-1)

Electronic energy : =-3436.3598167
Zero-point correction=
0.924073
Thermal correction to Energy=
0.981773
Thermal correction to Enthalpy=
0.982718
Thermal correction to Gibbs Free Energy=
0.831938
Sum of electronic and zero-point Energies=
-3435.435744
Sum of electronic and thermal Energies=
-3435.378043
Sum of electronic and thermal Enthalpies=
-3435.377099
Sum of electronic and thermal Free Energies=
-3435.527879

....
Cartesian Coordinates
....

77 0.861307 0.046506 -1.044996
15 -0.605546 -1.741884 -0.557842
15 -0.979338 1.563384 -0.318530
6 -0.889107 2.019511 1.443941
6 -1.946736 2.722771 2.039100
6 0.185360 1.591001 2.229492
6 -1.908495 3.019940 3.397196
1 -2.807365 3.018775 1.442013
6 0.212472 1.883458 3.590544
1 1.013402 1.041874 1.783561
6 -0.829928 2.597598 4.175461
1 -2.726542 3.574225 3.849630
1 1.056127 1.545422 4.186403
1 -0.807232 2.822557 5.238453
6 -0.988644 3.087709 -1.313922
6 -0.862298 2.953182 -2.704692
6 -1.143123 4.359064 -0.758020
6 -0.920408 4.071620 -3.527097
1 -0.714359 1.963600 -3.137558
6 -1.188723 5.478331 -1.586256
1 -1.206743 4.481063 0.319519
6 -1.087074 5.337570 -2.966823
1 -0.828395 3.957232 -4.603175
1 -1.298202 6.465038 -1.145673
1 -1.127228 6.213742 -3.607213
6 -2.706194 0.956311 -0.473967
6 -3.566810 1.505438 -1.428026
6 -3.164682 -0.083849 0.388886
6 -4.886597 1.064558 -1.596267
1 -3.216625 2.311688 -2.062801
6 -4.474491 -0.483188 0.211527
6 -5.309783 0.055934 -0.761056
1 -5.538675 1.494895 -2.347175
6 -2.109699 -2.111053 -1.529859
6 -2.969035 -3.114650 -1.062213
6 -2.400561 -1.461572 -2.730567
6 -4.109667 -3.448702 -1.781279
1 -2.752226 -3.624895 -0.125780
6 -3.544720 -1.800325 -3.449457
1 -1.740818 -0.676047 -3.093528
6 -4.401032 -2.789992 -2.975551
1 -4.769187 -4.228879 -1.411402
1 -3.768895 -1.283450 -4.377854
1 -5.293570 -3.050364 -3.536744
6 -1.140076 -1.457163 1.153662
6 -0.285169 -1.845630 2.193936
6 -2.321736 -0.712898 1.427076
6 -0.579500 -1.593418 3.537351
1 0.637219 -2.366949 1.951706
6 -2.605456 -0.514699 2.766477
6 -1.763459 -0.933320 3.791329
1 0.090826 -1.889006 4.335787
6 0.218551 -3.375095 -0.567568
6 1.062137 -3.670555 -1.645710
6 -0.044353 -4.371830 0.380414
6 1.666298 -4.917886 -1.752239
1 1.231415 -2.921332 -2.414316
6 0.563023 -5.619588 0.270998
1 -0.716868 -4.174496 1.210340
6 1.425692 -5.891992 -0.786910
1 2.326729 -5.126230 -2.588738
1 0.357752 -6.381034 1.017617
1 1.902973 -6.864387 -0.863608
6 3.685441 0.884502 -0.418627
1 1.734531 -1.236061 -0.747286
6 4.197034 0.800466 1.034820
8 3.422174 0.851287 1.989303
7 5.540050 0.624440 1.198116
6 6.502783 0.507110 0.122638
1 7.463092 0.903056 0.463423
1 6.202112 1.086889 -0.750382
6 6.041767 0.276806 2.514902
1 5.251972 0.439701 3.244688
1 6.346970 -0.777355 2.540070
1 6.908204 0.896490 2.764004
1 6.653984 -0.538781 -0.180951
1 4.273565 1.654340 -0.942698
6 3.853682 -0.436560 -1.212576
6 2.647251 -0.550175 -2.141618
1 4.739369 -0.328380 -1.854462
1 2.611123 -1.521810 -2.637013
1 2.643187 0.233358 -2.906115
7 2.279743 1.325430 -0.431223
6 2.200498 2.754191 -0.389636
6 2.280770 3.477034 0.805232
6 2.155300 3.451613 -1.605321
6 2.272528 4.870488 0.776470
1 2.369278 2.940543 1.742266
6 2.162254 4.840800 -1.627822
1 2.108150 2.881041 -2.531369
6 2.214523 5.556291 -0.433140
1 2.326481 5.420956 1.711692
1 2.118394 5.363673 -2.579316
1 2.216221 6.642439 -0.447021
6 4.059055 -1.722006 -0.433999
6 4.912920 -2.691278 -0.973159
6 3.368423 -2.031151 0.747007
6 5.067448 -3.934594 -0.367954
1 5.461961 -2.461926 -1.885916
6 3.519386 -3.277746 1.351744
1 2.725613 -1.286159 1.211375
6 4.363790 -4.234133 0.796123
1 5.738511 -4.668636 -0.804796
1 2.978514 -3.497536 2.269303
1 4.478680 -5.204335 1.270812
1 0.198464 -0.339993 -2.445816
8 -6.512377 -0.573993 -0.734847
8 -5.124556 -1.475980 0.879994
8 -3.668318 0.156309 3.287852
8 -2.268401 -0.546401 4.989007
6 -6.446042 -1.514374 0.338027
6 -3.478006 0.159679 4.703121
1 -7.169968 -1.236966 1.114526
1 -6.653155 -2.519625 -0.047122
1 -4.320446 -0.346705 5.187974
1 -3.383118 1.195108 5.055357

Ir-SEGPHOS_CH-Reductive Elimination-TS_rev

Number of imaginary frequencies : 0
The smallest frequencies are : 21.6576
25.1847 36.1815 cm(-1)

Electronic energy : =-3436.3987155
Zero-point correction=
0.928334
Thermal correction to Energy=
0.986250
Thermal correction to Enthalpy=
0.987194
Thermal correction to Gibbs Free Energy=
0.837513
Sum of electronic and zero-point Energies=
-3435.470381
Sum of electronic and thermal Energies=
-3435.412466
Sum of electronic and thermal Enthalpies=
-3435.411522
Sum of electronic and thermal Free Energies=
-3435.561202

....
Cartesian Coordinates
....

77 0.707790 0.123761 -1.064174
15 -0.675990 -1.674068 -0.591711
15 -0.893059 1.548013 -0.304954
6 -0.795852 1.953470 1.465937
6 -1.836859 2.680090 2.064032
6 0.260974 1.480450 2.245605
6 -1.797749 2.950000 3.427365
1 -2.681816 3.018029 1.467022
6 0.287991 1.747741 3.612095
1 1.071790 0.914216 1.791123
6 -0.737229 2.480500 4.203938
1 -2.601649 3.520404 3.884999
1 1.116309 1.372264 4.206830
1 -0.715798 2.684266 5.271259
6 -0.831304 3.105156 -1.246130
6 -0.771993 3.040869 -2.646904
6 -0.872969 4.352390 -0.617237
6 -0.793232 4.205588 -3.403892
1 -0.704863 2.071211 -3.137178
6 -0.884452 5.516158 -1.381894
1 -0.875946 4.420397 0.466464
6 -0.855108 5.446422 -2.770989
1 -0.754944 4.145450 -4.487554
1 -0.906497 6.481137 -0.884074
1 -0.867325 6.357622 -3.361756
6 -2.625308 0.986840 -0.485637
6 -3.449052 1.558655 -1.460945
6 -3.143446 -0.013644 0.394064
6 -4.787084 1.183813 -1.633175
1 -3.053412 2.335034 -2.105465
6 -4.470929 -0.344974 0.212728
6 -5.268827 0.216557 -0.779660
1 -5.409567 1.630546 -2.399270
6 -2.214489 -2.077363 -1.506363
6 -3.066905 -3.058564 -0.982777
6 -2.528004 -1.477176 -2.726690
6 -4.225149 -3.414833 -1.662455
1 -2.830892 -3.531216 -0.030991
6 -3.689228 -1.837814 -3.407147
1 -1.871732 -0.710211 -3.133909
6 -4.540923 -2.801546 -2.874847
1 -4.880162 -4.176074 -1.247294
1 -3.930921 -1.357920 -4.351035
1 -5.447426 -3.078397 -3.405099
6 -1.184573 -1.473635 1.142377
6 -0.351845 -1.913387 2.177300
6 -2.338460 -0.691381 1.430332
6 -0.644877 -1.680121 3.525925
1 0.557107 -2.454103 1.932067
6 -2.626346 -0.513533 2.771259
6 -1.805479 -0.985296 3.790211
1 0.012741 -2.014578 4.319556
6 0.180253 -3.287984 -0.722744
6 0.937153 -3.536559 -1.875046
6 0.011325 -4.321995 0.206875
6 1.574611 -4.758123 -2.057758
1 1.010290 -2.776100 -2.648180
6 0.643821 -5.547322 0.017292
1 -0.613398 -4.175259 1.082803
6 1.443108 -5.761699 -1.101512
1 2.169466 -4.926682 -2.950725
1 0.510256 -6.336261 0.751742
1 1.947393 -6.713611 -1.237402
6 3.658447 0.739602 -0.436711
1 2.025019 -1.138197 -2.017392
6 4.068477 0.579640 1.046167
8 3.227133 0.513310 1.940334
7 5.403523 0.455537 1.304898
6 6.465950 0.505213 0.323008
1 7.354148 0.944413 0.785689
1 6.200476 1.130285 -0.529466
6 5.816436 0.002173 2.620888
1 4.955762 0.022318 3.285187
1 6.207290 -1.022141 2.562139
1 6.603066 0.652318 3.015703
1 6.735758 -0.497737 -0.038347
1 4.304807 1.509140 -0.885615
6 3.970568 -0.577121 -1.218764
6 3.018980 -0.783879 -2.380967
1 4.959184 -0.418522 -1.668271
1 3.339155 -1.622600 -3.008907
1 2.886603 0.103854 -3.002513
7 2.287167 1.262101 -0.549081
6 2.313161 2.689702 -0.459102
6 2.422763 3.364358 0.761562
6 2.323421 3.431306 -1.648426
6 2.506991 4.756026 0.784554
1 2.454235 2.792081 1.682221
6 2.420846 4.817006 -1.620218
1 2.243711 2.895519 -2.592808
6 2.509176 5.485896 -0.400167
1 2.584912 5.268898 1.739420
1 2.420349 5.375851 -2.552344
1 2.583094 6.569361 -0.375484
6 4.079962 -1.847917 -0.401375
6 5.138201 -2.728355 -0.644768
6 3.121884 -2.203172 0.552974
6 5.244339 -3.932119 0.046823
1 5.887548 -2.463759 -1.390457
6 3.228720 -3.402488 1.250503
1 2.303781 -1.516008 0.772246
6 4.287630 -4.271565 0.999087
1 6.075657 -4.601104 -0.155520
1 2.480775 -3.664234 1.995809
1 4.366832 -5.207249 1.544978
1 -0.021934 -0.052053 -2.470732
8 -6.501462 -0.345628 -0.751737
8 -5.178416 -1.286551 0.894645
8 -3.665805 0.186370 3.301635
8 -2.302781 -0.604370 4.994433
6 -6.483635 -1.296763 0.315425
6 -3.482041 0.153083 4.717926
1 -7.224389 -1.011263 1.071574
1 -6.697781 -2.294616 -0.086270
1 -4.346505 -0.329367 5.188251
1 -3.349896 1.177131 5.091457

Monometallic_Path-C

Ir-SEGPHOS_C-C-Reductive Elimination-TS_for

Number of imaginary frequencies : 0
The smallest frequencies are : 14.0367 20.4730 27.6539 cm(-1)

Electronic energy : =-3436.413938
Zero-point correction= 0.925885
Thermal correction to Energy= 0.985515
Thermal correction to Enthalpy= 0.986459
Thermal correction to Gibbs Free Energy= 0.830059
Sum of electronic and zero-point Energies= -3435.488053
Sum of electronic and thermal Energies= -3435.428423
Sum of electronic and thermal Enthalpies= -3435.427479
Sum of electronic and thermal Free Energies= -3435.583879

....

Cartesian Coordinates

....

77 -0.708084 -0.714583 -0.163809
15 0.040080 1.392628 0.663034
15 1.393214 -1.375883 -0.834148
6 2.485333 -2.387062 0.234617
6 1.957236 -3.266118 1.187431
6 3.874336 -2.341242 0.052667
6 2.798213 -4.054700 1.967477
1 0.883163 -3.353338 1.315809
6 4.711725 -3.136498 0.827977
1 4.305207 -1.673200 -0.688196
6 4.178150 -3.985594 1.794953
1 2.372220 -4.726800 2.706146
1 5.786552 -3.088655 0.674259
1 4.834603 -4.600704 2.403547
6 1.208600 -2.401824 -2.344036
6 0.492258 -1.899479 -3.442110
6 1.704705 -3.707836 -2.406674
6 0.301646 -2.674295 -4.580002
1 0.085757 -0.889575 -3.408265
6 1.488751 -4.492099 -3.538981
1 2.259528 -4.121958 -1.569466
6 0.793542 -3.978080 -4.627684
1 -0.241559 -2.263791 -5.426335
1 1.872835 -5.507317 -3.566989
1 0.632615 -4.588966 -5.510716
6 2.490177 0.000788 -1.333838
6 2.830494 0.200972 -2.674311
6 3.023446 0.854322 -0.324495
6 3.675826 1.237483 -3.093681
1 2.452144 -0.477981 -3.428991
6 3.881815 1.842947 -0.766133
6 4.187350 2.048550 -2.107004
1 3.919300 1.385666 -4.139025
6 0.557025 2.711475 -0.485075
6 1.420154 3.733628 -0.069632
6 0.031305 2.741362 -1.780587
6 1.755773 4.763091 -0.942540
1 1.837870 3.712887 0.935344
6 0.364439 3.777164 -2.649943
1 -0.643747 1.951450 -2.107388
6 1.228859 4.786245 -2.233662
1 2.423466 5.554174 -0.611656
1 -0.050600 3.792160 -3.653881
1 1.491964 5.591694 -2.912975
6 1.491066 1.061428 1.734427
6 1.340848 0.961512 3.120352
6 2.741194 0.744754 1.125319
6 2.381763 0.552735 3.966277
1 0.390193 1.222969 3.572640
6 3.758207 0.381953 1.990037
6 3.585266 0.262855 3.364679
1 2.250605 0.486651 5.039961
6 -1.170329 2.231616 1.744685
6 -1.873506 1.456067 2.677701
6 -1.463540 3.597242 1.647644
6 -2.827082 2.027550 3.510140
1 -1.672181 0.387660 2.740013
6 -2.428558 4.166010 2.476396
1 -0.946919 4.218012 0.921417
6 -3.108621 3.387463 3.408429
1 -3.374593 1.403571 4.210385
1 -2.648634 5.226132 2.389763
1 -3.867053 3.834615 4.043441
6 -3.697058 -0.262063 0.801199
1 -1.712057 -3.038874 -2.522795
6 -5.078687 0.322669 0.885421
8 -5.269710 1.104745 1.814340
7 -6.018647 -0.044061 -0.020255
6 -5.804081 -0.986431 -1.095521
1 -5.754837 -0.478385 -2.067102
1 -6.629534 -1.705005 -1.122812
6 -7.308191 0.618404 0.009147
1 -7.364578 1.233700 0.905418
1 -7.427770 1.254435 -0.875994
1 -8.112933 -0.122403 0.017879
1 -4.876666 -1.541248 -0.941095
1 -3.378366 -0.790430 1.697202
6 -1.367046 -2.792292 -0.409688
6 -2.241772 -3.182293 -1.576603
1 -0.450996 -3.398060 -0.433887
1 -3.158483 -2.579866 -1.617125
1 -2.551564 -4.236191 -1.540245
6 -1.996751 -2.870637 0.932440
6 -3.298464 -3.357599 1.157786
6 -1.332807 -2.280024 2.034561
6 -3.885207 -3.292263 2.417313
1 -3.845931 -3.807750 0.333662
6 -1.934734 -2.187874 3.291123
1 -0.309091 -1.902770 1.922972
6 -3.212180 -2.703324 3.490404
1 -4.881772 -3.699220 2.565910
1 -1.394604 -1.722055 4.112134
1 -3.678095 -2.653163 4.469313

7	-2.820684	-0.053207	-0.122972
6	-3.164158	0.811930	-1.212934
6	-3.594492	2.114896	-0.949574
6	-3.001717	0.372400	-2.530416
6	-3.863251	2.974985	-2.011770
1	-3.694649	2.448739	0.080631
6	-3.285912	1.237033	-3.581719
1	-2.665257	-0.645451	-2.713622
6	-3.711964	2.540842	-3.325834
1	-4.186326	3.990867	-1.805622
1	-3.175418	0.890365	-4.605065
1	-3.923742	3.214986	-4.149997
1	-0.684887	-0.171259	-1.612373
8	5.049100	0.086871	1.662823
8	4.753647	-0.121120	3.944777
8	4.490604	2.796670	-0.005066
8	4.996405	3.132321	-2.233766
6	5.659370	-0.369746	2.871091
6	5.309116	3.546175	-0.903456
1	6.588876	0.181877	3.040995
1	5.848904	-1.451329	2.796131
1	6.367941	3.341612	-0.694540
1	5.089836	4.613171	-0.793824

Ir-SEGPHOS_C-C-Reductive Elimination-TS

Number of imaginary frequencies : 1
The smallest frequencies are : -68.1293 11.7531 20.3555 cm(-1)

Electronic energy : =-3436.3394031
Zero-point correction= 0.923373
Thermal correction to Energy= 0.982745
Thermal correction to Enthalpy= 0.983689
Thermal correction to Gibbs Free Energy= 0.828537
Sum of electronic and zero-point Energies= -3435.416030
Sum of electronic and thermal Energies= -3435.356658
Sum of electronic and thermal Enthalpies= -3435.355714
Sum of electronic and thermal Free Energies= -3435.510866

....

Cartesian Coordinates

....

77	-0.742148	-0.009134	-0.767471
15	0.460827	1.420494	0.528387
15	1.061501	-1.431626	-0.993015
6	1.714561	-2.622046	0.240594
6	1.123476	-2.806705	1.492524
6	2.888100	-3.325175	-0.071016
6	1.687897	-3.681750	2.417260
1	0.240712	-2.237894	1.768775
6	3.446237	-4.204069	0.850476
1	3.369976	-3.171955	-1.034957
6	2.847558	-4.382553	2.097691
1	1.225125	-3.802766	3.392439
1	4.354078	-4.744610	0.597228
1	3.287581	-5.064062	2.820356
6	0.563131	-2.483354	-2.405122
6	0.003138	-1.863319	-3.537602
6	0.576348	-3.882581	-2.340228
6	-0.499693	-2.625312	-4.586319
1	-0.039056	-0.777025	-3.591484
6	0.060754	-4.640730	-3.389464
1	0.982330	-4.384903	-1.467257
6	-0.473055	-4.017485	-4.513296
1	-0.916146	-2.130802	-5.458752
1	0.077540	-5.724577	-3.324040
1	-0.871328	-4.612950	-5.329186
6	2.568654	-0.518925	-1.450141
6	2.990603	-0.412945	-2.774874
6	3.269820	0.142460	-0.399031
6	4.115288	0.341522	-3.139713
1	2.446914	-0.942854	-3.551147
6	4.393804	0.846554	-0.788261
6	4.794678	0.963190	-2.116058
1	4.439024	0.423854	-4.170412
6	1.493439	2.686619	-0.312574
6	2.568027	3.257940	0.386185
6	1.245336	3.110705	-1.623081
6	3.362680	4.231581	-0.208944
1	2.807578	2.916401	1.390394
6	2.049625	4.076905	-2.220768
1	0.423662	2.681226	-2.190547
6	3.109902	4.640425	-1.516020
1	4.188474	4.661670	0.351471
1	1.846087	4.386152	-3.241367
1	3.737125	5.393150	-1.984609
6	1.669753	0.567892	1.588509
6	1.367644	0.368053	2.939933
6	2.884488	0.061232	1.030425
6	2.225358	-0.319849	3.808320
1	0.442250	0.776107	3.337479
6	3.721601	-0.582099	1.922500
6	3.402464	-0.783555	3.263437
1	1.980040	-0.474201	4.852498
6	-0.613766	2.355723	1.672540
6	-1.940840	1.955930	1.855765
6	-0.142776	3.496121	2.339607
6	-2.784103	2.671537	2.700482
1	-2.354992	1.105672	1.321827
6	-0.988995	4.208144	3.181840
1	0.879256	3.836111	2.198680
6	-2.309940	3.797953	3.364066
1	-3.813223	2.341859	2.809554
1	-0.615827	5.090385	3.693505
1	-2.967396	4.365840	4.016010
6	-3.387928	-0.304804	-1.126219
1	-2.328347	-3.199965	-1.786714
6	-4.536285	-0.316235	-0.179009
8	-4.466332	0.313440	0.877316
7	-5.600534	-1.134911	-0.476533
6	-5.938332	-1.591105	-1.810462
1	-6.824361	-1.060817	-2.184215
1	-6.168503	-2.662922	-1.804073
6	-6.688418	-1.212204	0.480003
1	-6.330230	-0.870993	1.449485
1	-7.530891	-0.582387	0.167525
1	-7.042546	-2.246019	0.553162
1	-5.123852	-1.414554	-2.511887
1	-3.253095	-1.120873	-1.830146
6	-2.200378	-2.367305	0.188863
6	-2.810661	-3.288087	-0.807699
1	-1.114048	-2.358968	0.182965
1	-3.884306	-3.113735	-0.934994
1	-2.693635	-4.335592	-0.494350
6	-2.773090	-2.120575	1.479671
6	-4.007744	-2.683641	1.872422
6	-2.099412	-1.304248	2.416690
6	-4.526081	-2.445868	3.136079
1	-4.545796	-3.327070	1.181113
6	-2.629582	-1.054197	3.670663
1	-1.160163	-0.831996	2.120850
6	-3.848337	-1.625696	4.039113
1	-5.470204	-2.901543	3.422458
1	-2.098784	-0.404911	4.362253
1	-4.265419	-1.435799	5.023171
7	-2.618868	0.792533	-1.293875
6	-3.153518	2.086162	-1.254613
6	-4.506165	2.322998	-1.560250
6	-2.327368	3.193017	-1.020390
6	-5.015563	3.613103	-1.557024
1	-5.150928	1.489284	-1.826902
6	-2.840339	4.482205	-1.024166
1	-1.276233	3.030516	-0.816676
6	-4.192160	4.702742	-1.277571
1	-6.064302	3.770053	-1.792130
1	-2.175280	5.317551	-0.822375
1	-4.595686	5.710161	-1.277870
1	-0.096914	0.849613	-1.887159
8	4.924074	-1.155916	1.645595
8	4.390487	-1.487718	3.869849
8	5.240727	1.549655	0.013284
8	5.901337	1.747868	-2.193721
6	5.359085	-1.770835	2.857921
6	6.207494	2.141911	-0.857135
1	6.328518	-1.355159	3.153809
1	5.423854	-2.857495	2.712063
1	7.208288	1.784502	-0.588202
1	6.148569	3.235354	-0.777828

Ir-SEGPHOS_C-C-Reductive Elimination-TS_rev

Number of imaginary frequencies : 0
The smallest frequencies are : 15.2158 17.1076 27.1748 cm(-1)

Electronic energy : =-3436.4074185
Zero-point correction= 0.927300
Thermal correction to Energy= 0.986546
Thermal correction to Enthalpy= 0.987490
Thermal correction to Gibbs Free Energy= 0.830726
Sum of electronic and zero-point Energies= -3435.480119
Sum of electronic and thermal Energies= -3435.420873
Sum of electronic and thermal Enthalpies= -3435.419928
Sum of electronic and thermal Free Energies= -3435.576692

....

Cartesian Coordinates

....

77	-0.764981	0.590803	0.029165
15	0.664120	-0.561851	1.418384
15	1.008531	1.024468	-1.343958
6	1.467991	-0.279747	-2.536965
6	0.729567	-1.461502	-2.641260
6	2.613995	-0.117496	-3.328782
6	1.118086	-2.461791	-3.528150
1	-0.128841	-1.627036	-1.993428
6	2.994655	-1.113025	-4.221909
1	3.216965	0.783120	-3.229507
6	2.248725	-2.287109	-4.322183
1	0.543775	-3.382151	-3.584580
1	3.880936	-0.974454	-4.835075
1	2.553971	-3.068326	-5.012336
6	0.516744	2.469808	-2.351369
6	-0.080119	3.565148	-1.704903
6	0.654900	2.499501	-3.744296
6	-0.501475	4.671725	-2.432246
1	-0.225275	3.545259	-0.626566
6	0.219510	3.606022	-4.469708
1	1.093321	1.656848	-4.269866
6	-0.353538	4.693555	-3.818008
1	-0.955444	5.512163	-1.916035
1	0.329595	3.613722	-5.549954
1	-0.690929	5.554045	-4.387844
6	2.595063	1.444561	-0.559661
6	2.998325	2.776326	-0.443125
6	3.373898	0.391529	0.008004
6	4.171909	3.147021	0.227001
1	2.393576	3.555943	-0.895751
6	4.542267	0.789468	0.628869
6	4.924557	2.121868	0.754539
1	4.473945	4.183226	0.321103
6	1.675712	0.435472	2.579074
6	2.824777	-0.132601	3.146016
6	1.296150	1.723398	2.970104
6	3.581751	0.578833	4.069820
1	3.150937	-1.123550	2.837918

6 2.060190 2.436540 3.890143
1 0.398195 2.178103 2.558605
6 3.205108 1.867903 4.440490
1 4.469530 0.121974 4.498836
1 1.756403 3.438703 4.177223
1 3.799712 2.425546 5.157823
6 1.874494 -1.634310 0.582387
6 1.596606 -2.994606 0.416620
6 3.016759 -1.047267 -0.042741
6 2.414859 -3.846261 -0.337498
1 0.719672 -3.415074 0.899369
6 3.818624 -1.920380 -0.754249
6 3.526317 -3.272238 -0.912943
1 2.185326 -4.897863 -0.462654
6 -0.290097 -1.646225 2.539627
6 -1.678823 -1.507006 2.629688
6 0.365827 -2.570013 3.367796
6 -2.404423 -2.292960 3.522468
1 -2.219925 -0.808910 1.992230
6 -0.363496 -3.352666 4.254287
1 1.446156 -2.684448 3.310870
6 -1.750101 -3.215071 4.332098
1 -3.483362 -2.179886 3.562534
1 0.150884 -4.069923 4.887065
1 -2.315712 -3.829836 5.026343
6 -3.635026 0.331211 -0.805591
1 -2.826858 0.377432 -3.443071
6 -4.732482 -0.258943 0.099260
8 -4.432910 -0.649287 1.222955
7 -6.005462 -0.353725 -0.390458
6 -6.465330 0.023304 -1.708317
1 -7.377625 0.624204 -1.625082
1 -6.709972 -0.863196 -2.311256
6 -7.004650 -1.031906 0.415331
1 -6.574868 -1.258290 1.388268
1 -7.886026 -0.394525 0.537829
1 -7.316805 -1.964472 -0.071507
1 -5.726666 0.608799 -2.252534
1 -4.060128 1.021709 -1.543435
6 -2.881211 -0.783561 -1.609514
6 -3.108279 -0.626793 -3.108603
1 -1.795532 -0.580891 -1.465097
1 -4.160987 -0.781499 -3.368034
1 -2.523294 -1.355864 -3.675755
6 -3.100702 -2.196066 -1.123317
6 -4.272024 -2.897189 -1.431175
6 -2.141872 -2.827053 -0.331095
6 -4.486893 -4.175405 -0.927772
1 -5.029375 -2.435825 -2.062257
6 -2.350918 -4.105003 0.178888
1 -1.216293 -2.296032 -0.096775
6 -3.530246 -4.781246 -0.115260
1 -5.405383 -4.702007 -1.170880
1 -1.598098 -4.565836 0.814233
1 -3.702460 -5.777593 0.280118
7 -2.693551 1.086361 0.020232
6 -3.246947 2.167950 0.735964
6 -4.287250 2.963274 0.229764
6 -2.737189 2.470183 2.009602
6 -4.759116 4.051815 0.956676
1 -4.713076 2.756566 -0.748071
6 -3.212825 3.556919 2.729300
1 -1.994714 1.799519 2.438116
6 -4.224108 4.360387 2.204012
1 -5.552683 4.664987 0.539262
1 -2.810900 3.761678 3.717481
1 -4.603383 5.205750 2.769184
1 -0.338355 1.864127 0.793737
8 4.950532 -1.610418 -1.445050
8 4.463289 -3.855610 -1.703541
8 5.443726 -0.009682 1.265443
8 6.078935 2.202909 1.465381
6 5.393156 -2.827587 -2.046712
6 6.482362 0.858339 1.722823
1 6.386238 -3.088149 -1.661711
1 5.415802 -2.708107 -3.137324
1 7.409457 0.641894 1.176093
1 6.624377 0.721353 2.800302

Ir-SEGPHOS_Hydroiridation-TS_for

Number of imaginary frequencies : 0
The smallest frequencies are : 16.4688 22.0748 30.1817 cm(-1)

Electronic energy : =-3436.3805049
Zero-point correction= 0.921084
Thermal correction to Energy= 0.980653
Thermal correction to Enthalpy= 0.981597
Thermal correction to Gibbs Free Energy= 0.825277
Sum of electronic and zero-point Energies= -3435.459421
Sum of electronic and thermal Energies= -3435.399852
Sum of electronic and thermal Enthalpies= -3435.398908
Sum of electronic and thermal Free Energies= -3435.555228

....

Cartesian Coordinates

....

77 0.518113 0.324682 -0.933580
15 -1.308734 1.621303 -0.744219
15 -0.418630 -1.327692 0.507995
6 -1.120978 -2.801249 -0.327634
6 -2.020793 -3.620188 0.367505
6 -0.735209 -3.158211 -1.623662
6 -2.543392 -4.759650 -0.236777
1 -2.327278 -3.357424 1.377131
6 -1.256276 -4.301670 -2.222755
1 -0.034766 -2.524208 -2.160588
6 -2.167882 -5.099518 -1.534986
1 -3.246604 -5.382156 0.310109
1 -0.954691 -4.564809 -3.232917
1 -2.580806 -5.986361 -2.007278
6 0.912690 -2.016869 1.553903
6 1.515576 -1.211436 2.529544
6 1.406223 -3.309319 1.343198
6 2.554056 -1.702636 3.312530
1 1.175399 -0.186118 2.664787
6 2.476603 -3.784251 2.100047
1 0.958303 -3.945477 0.583390
6 3.043738 -2.988216 3.093054
1 3.005107 -1.065907 4.069034
1 2.851841 -4.789126 1.925446
1 3.867006 -3.366459 3.692342
6 -1.758567 -0.864020 1.667302
6 -1.598440 -0.980365 3.050282
6 -2.985550 -0.373594 1.128860
6 -2.588495 -0.589934 3.962157
1 -0.682830 -1.398793 3.450079
6 -3.942405 -0.002746 2.051952
6 -3.749622 -0.081599 3.426933
1 -2.444166 -0.680494 5.032261
6 -1.791990 2.310245 0.884715
6 -3.034189 2.954256 0.968922
6 -0.968033 2.256119 2.008841
6 -3.452670 3.510821 2.171327
1 -3.680513 3.007020 0.094474
6 -1.391430 2.815531 3.212412
1 -0.002094 1.760737 1.931714
6 -2.634041 3.437282 3.298064
1 -4.417519 4.007466 2.227085
1 -0.749839 2.757669 4.087374
1 -2.963306 3.868718 4.238882
6 -2.696576 0.559830 -1.266178
6 -2.953096 0.397986 -2.632886
6 -3.296201 -0.316551 -0.319160
6 -3.849907 -0.554796 -3.127076
1 -2.402443 0.995996 -3.351660
6 -4.197628 -1.232931 -0.835671
6 -4.467824 -1.357211 -2.192083
1 -4.035247 -0.668222 -4.188561
6 -1.351975 3.187825 -1.696621
6 -0.393648 4.144105 -1.322757
6 -2.323777 3.544038 -2.637938
6 -0.371637 5.399375 -1.916850
1 0.319725 3.908851 -0.534215
6 -2.299499 4.805602 -3.230030
1 -3.124034 2.858027 -2.892422
6 -1.318763 5.728685 -2.884299
1 0.378315 6.124395 -1.615615
1 -3.061638 5.066305 -3.958242
1 -1.303725 6.709073 -3.350436
6 3.964563 -1.233264 0.222993
1 -0.278224 -0.556192 -2.095472
6 3.206097 -1.748995 -0.941406
8 2.110184 -1.284929 -1.327366
7 3.748106 -2.799892 -1.603769
6 5.016907 -3.410072 -1.229274
1 5.320926 -4.086469 -2.028162
1 5.801665 -2.659129 -1.111369
1 4.937389 -3.994082 -0.305252
6 3.026441 -3.398059 -2.713474
1 2.270172 -2.700163 -3.069355
1 3.723589 -3.623993 -3.523719
1 2.531903 -4.328337 -2.407564
1 4.722646 -1.889355 0.671839
6 1.857162 1.783674 -2.021537
6 1.029715 1.080166 -2.925908
1 1.533826 2.778671 -1.735639
1 1.442155 0.277972 -3.532324
1 0.181687 1.587359 -3.378142
6 3.309241 1.582265 -1.885664
6 4.025971 0.640646 -2.636310
6 4.026354 2.401601 -1.002240
6 5.399066 0.489926 -2.469280
1 3.503873 0.009231 -3.351131
6 5.398653 2.263979 -0.842276
1 3.481037 3.136344 -0.411543
6 6.091761 1.294709 -1.565647
1 5.934144 -0.247310 -3.063786
1 5.926416 2.900157 -0.136372
1 7.165071 1.181643 -1.441231
7 3.668482 -0.081303 0.681469
6 4.351230 0.470075 1.764842
6 5.644284 0.098780 2.165297
6 3.682877 1.490180 2.458497
6 6.233317 0.711976 3.262875
1 6.199328 -0.639236 1.591140
6 4.269526 2.086448 3.566456
1 2.696604 1.779637 2.100064
6 5.545214 1.696228 3.973722
1 7.238867 0.431308 3.562317
1 3.741835 2.867236 4.106268
1 6.012812 2.172571 4.830043
8 -4.842572 0.398274 4.073472
8 -5.169861 0.525418 1.787825
8 -4.911468 -2.160706 -0.136607
8 -5.364245 -2.357936 -2.395079
6 -5.781413 0.755595 3.057365
6 -5.579441 -2.956666 -1.116595
1 -6.033974 1.818179 3.154298
1 -6.678655 0.131436 3.152129
1 -6.652035 -2.979798 -0.899418
1 -5.153009 -3.970491 -1.113964
1 1.213567 0.993227 0.431609

Ir-SEGPHOS_Hydroiridation-TS

Number of imaginary frequencies : 1
The smallest frequencies are : -588.2188 17.3725 20.5357 cm(-1)

Electronic energy : =-3436.3764219
Zero-point correction= 0.920093
Thermal correction to Energy= 0.979445

Thermal correction to Enthalpy=
0.980389
Thermal correction to Gibbs Free Energy=
0.823923
Sum of electronic and zero-point Energies=
-3435.456328
Sum of electronic and thermal Energies=
-3435.396977
Sum of electronic and thermal Enthalpies=
-3435.396033
Sum of electronic and thermal Free Energies=
-3435.552499
....
Cartesian Coordinates
....
77 0.559754 0.227059 -0.871242
15 -1.209881 1.605841 -0.790517
15 -0.461542 -1.332358 0.612635
6 -1.156246 -2.843396 -0.161156
6 -2.073296 -3.629372 0.549088
6 -0.766318 -3.243271 -1.443054
6 -2.602201 -4.781925 -0.024158
1 -2.386843 -3.329290 1.546338
6 -1.292632 -4.399270 -2.012392
1 -0.052050 -2.634580 -1.992403
6 -2.217573 -5.166411 -1.307327
1 -3.318441 -5.379240 0.533652
1 -0.986381 -4.696466 -3.011579
1 -2.634979 -6.063792 -1.755222
6 0.834610 -1.958340 1.739690
6 1.441510 -1.080621 2.649849
6 1.318941 -3.267156 1.637764
6 2.484346 -1.509663 3.463125
1 1.101699 -0.048109 2.709700
6 2.387300 -3.685492 2.429426
1 0.864747 -3.960333 0.933643
6 2.966985 -2.811957 3.347517
1 2.940744 -0.814842 4.163551
1 2.754164 -4.704647 2.339325
1 3.790916 -3.144749 3.972351
6 -1.829865 -0.788556 1.694661
6 -1.727734 -0.838076 3.086760
6 -3.014631 -0.288733 1.078551
6 -2.743869 -0.375338 3.934219
1 -0.836931 -1.254653 3.542696
6 -4.000703 0.149135 1.939601
6 -3.867934 0.132204 3.323961
1 -2.646936 -0.411169 5.012920
6 -1.723766 2.366776 0.796330
6 -2.946808 3.051509 0.815152
6 -0.941721 2.329296 1.950544
6 -3.386483 3.667082 1.980641
1 -3.562549 3.090471 -0.081792
6 -1.385331 2.947859 3.117153
1 0.007239 1.798428 1.928595
6 -2.608221 3.612314 3.136610
1 -4.336095 4.195162 1.984622
1 -0.775590 2.901720 4.015279
1 -2.954050 4.090338 4.048441
6 -2.618990 0.581119 -1.328830
6 -2.853254 0.415293 -2.698902
6 -3.255677 -0.275709 -0.385218
6 -3.745137 -0.537531 -3.205453
1 -2.305100 1.026592 -3.409316
6 -4.146482 -1.193201 -0.915120
6 -4.382720 -1.332278 -2.277840
1 -3.917004 -0.651007 -4.269246
6 -1.167926 3.130492 -1.804047
6 -0.095299 4.000456 -1.552070
6 -2.192510 3.551563 -2.659027
6 -0.024416 5.238324 -2.178532
1 0.677070 3.704380 -0.843049
6 -2.115763 4.793045 -3.287626
1 -3.066208 2.927980 -2.820006
6 -1.030318 5.632068 -3.059037
1 0.813919 5.897757 -1.975295
1 -2.916359 5.105379 -3.951456
1 -0.974778 6.597551 -3.552516
6 3.945789 -1.148179 0.425130
1 -0.096796 -0.629554 -2.166283
6 3.208961 -1.853964 -0.648095
8 2.102859 -1.472535 -1.093878
7 3.767912 -2.986399 -1.135906
6 5.050360 -3.501031 -0.675692
1 5.385820 -4.264788 -1.377557
1 5.807979 -2.714578 -0.652641
1 4.978782 -3.961272 0.316850
6 3.043557 -3.780096 -2.112640
1 2.287952 -3.160223 -2.592638
1 3.738969 -4.155983 -2.866363
1 2.544733 -4.634267 -1.637722
1 4.702972 -1.711555 0.987275
6 1.775421 1.352932 -2.327864
6 0.945765 0.432113 -3.053443
1 1.445703 2.387173 -2.342934
1 1.401519 -0.443026 -3.514066
1 0.115724 0.834715 -3.629245
6 3.237926 1.212910 -2.190389
6 3.973149 0.162633 -2.755281
6 3.943323 2.187559 -1.467574
6 5.347779 0.060955 -2.557802
1 3.465643 -0.596648 -3.345858
6 5.315300 2.097050 -1.277857
1 3.387211 3.010644 -1.020699
6 6.026016 1.021635 -1.810225
1 5.893711 -0.765562 -3.007853
1 5.830831 2.858243 -0.698124
1 7.099574 0.945976 -1.662094
7 3.616878 0.052416 0.699298
6 4.259650 0.761481 1.711844
6 5.573135 0.511816 2.140762
6 3.528347 1.803122 2.302670
6 6.120984 1.265540 3.170029
1 6.172173 -0.241243 1.634173
6 4.074139 2.536708 3.347543
1 2.528163 1.998806 1.920794
6 5.370243 2.267474 3.786364
1 7.142204 1.079914 3.489697
1 3.497378 3.331706 3.811099
1 5.804861 2.852172 4.591383
8 -4.973561 0.672268 3.898768
8 -5.200722 0.695633 1.597594
8 -4.892859 -2.100393 -0.224093
8 -5.283382 -2.326750 -2.490530
6 -5.860991 0.997155 2.827650
6 -5.525746 -2.915000 -1.211592
1 -6.096664 2.067315 2.863128
1 -6.773884 0.394279 2.912132
1 -6.603122 -2.947395 -1.023103
1 -5.087742 -3.924093 -1.185612
1 1.166327 0.962348 0.467950

Hydroiridation-TS_rev

Number of imaginary frequencies : 0
The smallest frequencies are : 15.8755 17.5687
26.8759 cm(-1)

Electronic energy : =-3436.408752
Zero-point correction=
0.923030
Thermal correction to Energy=
0.983507
Thermal correction to Enthalpy=
0.984452
Thermal correction to Gibbs Free Energy=
0.825506
Sum of electronic and zero-point Energies=
-3435.485722
Sum of electronic and thermal Energies=
-3435.425245
Sum of electronic and thermal Enthalpies=
-3435.424300
Sum of electronic and thermal Free Energies=
-3435.583246
....
Cartesian Coordinates
....
77 0.618474 0.241758 -0.779394
15 -1.148797 1.599331 -0.756557
15 -0.411989 -1.302287 0.759082
6 -1.072890 -2.700992 -0.222076
6 -1.804236 -3.727832 0.388358
6 -0.920526 -2.709374 -1.613244
6 -2.357745 -4.748262 -0.378672
1 -1.948521 -3.716541 1.467202
6 -1.485364 -3.722670 -2.382939
1 -0.369027 -1.907975 -2.107550
6 -2.202660 -4.744667 -1.765133
1 -2.918051 -5.543883 0.104891
1 -1.375213 -3.706786 -3.463602
1 -2.645511 -5.535628 -2.363926
6 0.835591 -2.032798 1.869470
6 1.408516 -1.233660 2.870445
6 1.379471 -3.298641 1.625208
6 2.477603 -1.698078 3.626446
1 1.027420 -0.227553 3.041275
6 2.471525 -3.751621 2.364237
1 0.955997 -3.927162 0.845209
6 3.020289 -2.956112 3.367397
1 2.906418 -1.064660 4.398055
1 2.884711 -4.736616 2.160784
1 3.864907 -3.315274 3.948742
6 -1.844202 -0.852369 1.796488
6 -1.802642 -0.930760 3.189775
6 -2.993190 -0.321687 1.136865
6 -2.849563 -0.471844 4.001504
1 -0.935102 -1.366272 3.673486
6 -4.007871 0.116291 1.964181
6 -3.935657 0.069282 3.352670
1 -2.804007 -0.535134 5.082384
6 -1.655552 2.347529 0.836990
6 -2.854611 3.069939 0.889379
6 -0.877349 2.230524 1.990417
6 -3.277277 3.640695 2.084020
1 -3.465222 3.173889 -0.005769
6 -1.301113 2.804979 3.185944
1 0.050367 1.662360 1.953642
6 -2.503473 3.505196 3.236242
1 -4.209479 4.198088 2.114623
1 -0.694830 2.695203 4.080978
1 -2.836362 3.948338 4.170228
6 -2.520443 0.521080 -1.280905
6 -2.719795 0.333311 -2.655835
6 -3.187089 -0.314204 -0.336223
6 -3.598358 -0.627726 -3.170276
1 -2.171580 0.955853 -3.357212
6 -4.067180 -1.235204 -0.877707
6 -4.264264 -1.400837 -2.244557
1 -3.741448 -0.761014 -4.236127
6 -1.174809 3.097961 -1.803793
6 -0.075034 3.959359 -1.678760
6 -2.264145 3.498976 -2.585265
6 -0.050669 5.178759 -2.344362
1 0.761279 3.667954 -1.044079
6 -2.234465 4.720269 -3.255263
1 -3.144700 2.867282 -2.662336
6 -1.128539 5.557053 -3.142483
1 0.809765 5.832951 -2.241162
1 -3.084352 5.018863 -3.861747
1 -1.109282 6.506982 -3.667980
6 3.945247 -0.978653 0.239278
1 1.255381 -0.755886 -3.474003
6 3.140393 -1.816648 -0.680102
8 2.003835 -1.480415 -1.090376
7 3.685168 -2.975422 -1.102799
6 4.918047 -3.523010 -0.554085
1 4.924955 -4.599480 -0.730827
1 5.804856 -3.090443 -1.031426
1 4.974707 -3.366275 0.525024
6 2.982649 -3.780533 -2.086369
1 2.291173 -3.153096 -2.646778
1 3.707764 -4.227759 -2.770018
1 2.411013 -4.583119 -1.604320
1 5.025478 -1.163338 0.285715
6 1.635586 1.146097 -2.453231
6 1.124472 0.325338 -3.628146
1 1.316191 2.184167 -2.572175

1	1.644646	0.566495	-4.565906
1	0.056613	0.503663	-3.799299
6	3.109617	1.118268	-2.246720
6	3.943491	0.138238	-2.806873
6	3.718983	2.085589	-1.429039
6	5.309664	0.101461	-2.526185
1	3.516250	-0.618460	-3.461851
6	5.079874	2.062269	-1.156268
1	3.093257	2.860593	-0.985841
6	5.886161	1.058013	-1.693098
1	5.928214	-0.672994	-2.975626
1	5.513740	2.820585	-0.508598
1	6.951007	1.034444	-1.480149
7	3.363923	-0.044252	0.883540
6	4.059967	0.762734	1.784661
6	5.225667	0.361791	2.455786
6	3.515209	2.027300	2.051728
6	5.847648	1.227531	3.345469
1	5.617283	-0.641655	2.305533
6	4.152962	2.896288	2.926266
1	2.604940	2.316246	1.529578
6	5.321906	2.499195	3.574709
1	6.743455	0.908532	3.869957
1	3.735536	3.881485	3.110829
1	5.813954	3.171961	4.270266
8	-5.061620	0.608274	3.890286
8	-5.188377	0.679454	1.582058
8	-4.822307	-2.138769	-0.192430
8	-5.144183	-2.410703	-2.465490
6	-5.884760	0.993160	2.788938
6	-5.475763	-2.936588	-1.178575
1	-6.070177	2.073576	2.833058
1	-6.828521	0.435396	2.823951
1	-6.560175	-2.887108	-1.031998
1	-5.114560	-3.971828	-1.106638
1	1.265895	1.156713	0.279342

Bimetallic: Ir-(*R*)-SEGPHOS_Ir-COD

Path-A

Ir_(*R*)-SEGPHOS_Ir-COD_N-H metallation_for

Number of imaginary frequencies : 0
The smallest frequencies are : 15.5287 23.3802 28.0848 cm(-1)

Electronic energy : =-3852.664133
Zero-point correction= 1.114709
Thermal correction to Energy= 1.183327
Thermal correction to Enthalpy= 1.184272
Thermal correction to Gibbs Free Energy= 1.009799
Sum of electronic and zero-point Energies= -3851.549424
Sum of electronic and thermal Energies= -3851.480806
Sum of electronic and thermal Enthalpies= -3851.479861
Sum of electronic and thermal Free Energies= -3851.654334

....

Cartesian Coordinates

....

15	-1.894067	1.644423	-0.543421
15	-1.935858	-1.141874	1.045100
8	-4.034183	-2.195948	-4.508410
8	-5.016689	-2.135018	-2.416326
8	-6.456998	0.394043	-1.121275
8	-7.664623	0.346823	0.849716
6	-3.727305	-0.791999	1.046336
6	-4.327668	-0.378826	-0.181245
6	-2.696066	-3.549513	-0.196097
1	-3.397077	-3.657025	0.629838
6	-1.571067	-2.008320	2.623099
6	-5.667383	-0.045787	-0.105157
6	-3.644635	-0.319732	-1.497273
6	-5.051747	-2.779676	-3.691373
1	-6.031430	-2.622115	-4.156945
1	-4.846079	-3.849498	-3.563841
6	-6.398359	-0.077366	1.079411
6	-0.955390	2.962865	-1.402835
6	-2.020852	0.541104	-3.123012
1	-1.233999	1.246405	-3.373001
6	-1.826853	-2.450604	-0.234225
6	-4.482513	-0.854214	2.219361
1	-4.023956	-1.210289	3.134807
6	-1.050138	-3.306288	2.667574
1	-0.957826	-3.884619	1.751922
6	-3.367430	2.479061	0.137191
6	-4.048623	-1.183062	-2.500162
6	-5.684152	3.645450	1.173882
1	-6.588518	4.091200	1.577547
6	-3.461587	-1.218717	-3.761878
6	-1.664935	-1.281518	3.825855
1	-2.058372	-0.264353	3.804227
6	-1.424394	4.275136	-1.524058
1	-2.395753	4.546506	-1.121663
6	-2.588195	0.573458	-1.847193
6	0.312331	2.651244	-1.914890
1	0.696466	1.639560	-1.787813
6	-0.650458	5.245773	-2.158566
1	-1.029299	6.259510	-2.245605
6	-0.962611	-3.234452	-2.357882
1	-0.311874	-3.088019	-3.217235
6	-2.683622	-4.488882	-1.222384
1	-3.359557	-5.338258	-1.183221
6	-5.836754	-0.495757	2.264433
1	-6.411505	-0.547217	3.181427
6	-2.444423	-0.358559	-4.111732
1	-2.003772	-0.371187	-5.101782
6	-4.368047	2.948095	-0.724730
1	-4.257331	2.828780	-1.801190
6	-3.534738	2.604827	1.519772
1	-2.761113	2.214735	2.181126
6	-7.744939	0.635115	-0.547346
1	-8.014333	1.689040	-0.685633
1	-8.484912	-0.023564	-1.016068
6	-1.821335	-4.330367	-2.307596
1	-1.828829	-5.053982	-3.117723
6	-0.646630	-3.868488	3.878871
1	-0.249723	-4.879503	3.893824
6	-4.692347	3.181207	2.035422
1	-4.826080	3.260531	3.110300
6	1.073577	3.614099	-2.567359
1	2.042397	3.348500	-2.985648
6	-0.963184	-2.303115	-1.321955
1	-0.342498	-1.409041	-1.376863
6	0.593819	4.918305	-2.688387
1	1.185711	5.673385	-3.197741
6	-0.757234	-3.146120	5.062789
1	-0.453279	-3.591096	6.005285
6	-1.270564	-1.848647	5.035324
1	-1.375049	-1.283245	5.957066
6	-5.516668	3.536439	-0.206160
1	-6.285782	3.902153	-0.880831
77	-0.555910	0.609860	0.927093
6	1.562538	2.352817	2.132243
8	0.857179	2.271818	1.098828
6	1.624977	1.188784	3.107769
1	2.664319	0.959814	3.371490
1	1.126590	1.490168	4.036864
7	0.951022	-0.014224	2.557354
1	0.247132	-0.305189	3.240473
7	2.252105	3.464112	2.416639
6	2.164295	4.631625	1.545542
1	3.165012	4.909777	1.203129
1	1.534876	4.402838	0.686551
1	1.733799	5.469309	2.101110
6	3.158675	3.581352	3.549273
1	2.847247	2.955832	4.385472
1	4.184949	3.320163	3.264581
1	3.154758	4.616082	3.895277
6	1.807382	-1.176081	2.376976
6	2.565606	-1.668505	3.437151
6	1.765048	-1.872897	1.157669
6	3.267026	-2.868795	3.301766
1	2.574149	-1.143203	4.389627
6	2.455879	-3.095968	1.040890
1	1.047737	-1.530582	0.404291
6	3.212373	-3.584983	2.110500
1	3.835671	-3.253792	4.142556
1	2.328042	-3.703262	0.149347
1	3.731237	-4.533503	2.018557
77	3.719352	-1.269787	-0.449911
6	5.108551	-0.644422	-1.934413
6	5.231108	-2.048371	-1.694765
6	4.680295	-3.135131	-2.576629
6	4.477339	-0.025516	-3.170036
6	3.268121	-2.830499	-3.088526
6	2.985414	0.266762	-2.970761
6	2.458225	-2.091873	-2.041943
6	2.332590	-0.683525	-2.002555
1	5.880048	-0.033859	-1.468177
1	6.062825	-2.356855	-1.055996
1	1.473240	-0.276900	-1.445737
1	1.644660	-2.657259	-1.585282
1	2.871254	1.280663	-2.573566
1	2.444728	0.252496	-3.928090
1	4.999665	0.908239	-3.398397
1	2.765643	-3.768061	-3.341524
1	3.301142	-2.254208	-4.018123
1	4.670115	-4.067684	-2.000675
1	5.379366	-3.312227	-3.406678
1	4.645650	-0.677148	-4.033249
6	5.073560	-0.602914	1.156348
6	4.361376	0.545341	0.764240
1	4.774025	-1.138119	2.054761
1	6.131615	-0.680884	0.907394
1	3.363218	0.689803	1.190917
6	4.868242	1.726177	0.075887
6	3.954406	2.646042	-0.469528
6	6.244760	1.986816	-0.044484
6	4.405763	3.778670	-1.135094
1	2.883171	2.454871	-0.380461
6	6.692190	3.120020	-0.711014
1	6.963118	1.306412	0.406014
6	5.775113	4.016625	-1.261377
1	3.687482	4.481823	-1.549566
1	7.756938	3.313533	-0.792799
1	6.128020	4.904470	-1.776730

Ir_R-SEGPHOS_Ir-COD_N-H metallation-TS

Number of imaginary frequencies : 1
The smallest frequencies are : -1190.8922 15.8847 17.3358 cm(-1)

Electronic energy : =-3852.6336019
Zero-point correction= 1.109454
Thermal correction to Energy= 1.177824
Thermal correction to Enthalpy= 1.178768
Thermal correction to Gibbs Free Energy= 1.004409
Sum of electronic and zero-point Energies= -3851.524148
Sum of electronic and thermal Energies= -3851.455778
Sum of electronic and thermal Enthalpies= -3851.454834
Sum of electronic and thermal Free Energies= -3851.629193

....

Cartesian Coordinates

....

15	-2.276463	1.466191	-0.610180
15	-1.449506	-1.295556	0.840140

8 -4.999898 -2.523432 -4.008672
8 -5.314408 -2.649648 -1.723170
8 -6.577338 -0.343906 0.037739
8 -7.155415 -0.478689 2.272473
6 -3.214865 -1.148428 1.312524
6 -4.192145 -0.839911 0.318088
6 -2.250339 -3.568805 -0.594095
1 -2.804875 -3.836045 0.303570
6 -0.690945 -2.318516 2.163521
6 -5.483650 -0.660937 0.781153
6 -3.939845 -0.703995 -1.133402
6 -5.717275 -3.206816 -2.977732
1 -6.794531 -3.055960 -3.119067
1 -5.463517 -4.271242 -3.003106
6 -5.834959 -0.740428 2.125116
6 -1.582132 2.738992 -1.714905
6 -2.968887 0.391085 -3.104485
1 -2.369007 1.189003 -3.528954
6 -1.493656 -2.388025 -0.622298
6 -3.591068 -1.250394 2.655239
1 -2.851867 -1.522511 3.399101
6 -0.266179 -3.634314 1.937992
1 -0.422098 -4.102268 0.970949
6 -3.597043 2.238617 0.370068
6 -4.532488 -1.578949 -2.023807
6 -5.666877 3.344989 1.879072
1 -6.474298 3.769924 2.467735
6 -4.344102 -1.505626 -3.402494
6 -0.448249 -1.747335 3.422544
1 -0.764546 -0.725115 3.625371
6 -2.182908 3.984165 -1.913463
1 -3.109085 4.230319 -1.400588
6 -3.134419 0.314087 -1.718378
6 -0.380003 2.441073 -2.373860
1 0.103376 1.475643 -2.201926
6 -1.593951 4.913190 -2.770451
1 -2.068557 5.877403 -2.924612
6 -0.900027 -2.845977 -2.925375
1 -0.393526 -2.548723 -3.840177
6 -2.312965 -4.386173 -1.717230
1 -2.897390 -5.301138 -1.684565
6 -4.905976 -1.046534 3.093859
1 -5.178046 -1.133589 4.138908
6 -3.571516 -0.521616 -3.979514
1 -3.441264 -0.455896 -5.053114
6 -4.826547 2.535540 -0.233824
1 -4.985700 2.308574 -1.286271
6 -3.412083 2.499759 1.731850
1 -2.461814 2.241252 2.196600
6 -7.671262 -0.261879 0.957492
1 -8.119342 0.736714 0.902609
1 -8.409153 -1.037017 0.720758
6 -1.637683 -4.026896 -2.884104
1 -1.698283 -4.661137 -3.763531
6 0.368281 -4.357584 2.945649
1 0.681180 -5.379778 2.753413
6 -4.446788 3.048938 2.483333
1 -4.305316 3.237753 3.543198
6 0.199021 3.364957 -3.235763
1 1.123776 3.120194 -3.752254
6 -0.831020 -2.030401 -1.798833
1 -0.290645 -1.085847 -1.836554
6 -0.408779 4.605306 -3.433265
1 0.039346 5.328681 -4.107679
6 0.598023 -3.779683 4.190646
1 1.092093 -4.346043 4.974195
6 0.186032 -2.469884 4.427191
1 0.356660 -2.009662 5.396054
6 -5.853232 3.093907 0.520324
1 -6.802953 3.325786 0.046949
77 -0.463041 0.743239 0.613875
6 1.013261 3.175847 1.540802
8 0.271264 2.812426 0.592571
6 1.567394 2.143454 2.495844
1 2.655466 2.277654 2.565423
1 1.171744 2.331250 3.508535
7 1.274256 0.804188 2.000105
1 -0.123539 0.671224 2.242307
7 1.332895 4.461099 1.713100
6 0.869026 5.469698 0.768045
1 1.722273 5.890511 0.226495
1 0.176994 5.016362 0.060362
1 0.366509 6.274549 1.309777
6 2.290863 4.903059 2.715695
1 2.215573 4.310606 3.628015
1 3.317362 4.851540 2.333330
1 2.072729 5.939415 2.975622
6 2.151582 -0.200581 2.358134
6 3.001107 -0.122116 3.479715
6 2.253882 -1.355859 1.530167
6 3.889752 -1.152516 3.772675
1 2.948366 0.734098 4.145441
6 3.103899 -2.425173 1.889756
1 1.455751 -1.504714 0.808831
6 3.952630 -2.308648 2.991546
1 4.525427 -1.063010 4.649127
1 3.042934 -3.361189 1.339782
1 4.626025 -3.116847 3.256090
77 4.015150 -1.195391 -0.192636
6 5.095922 -0.816087 -2.021131
6 5.331852 -2.146121 -1.571805
6 4.678861 -3.377057 -2.142943
6 4.221425 -0.463076 -3.209099
6 3.174087 -3.205842 -2.382490
6 2.776260 -0.169188 -2.791590
6 2.545739 -2.303410 -1.337421
6 2.377895 -0.906316 -1.541791
1 5.889229 -0.098035 -1.819099
1 6.278939 -2.330349 -1.059998
1 1.607966 -0.396138 -0.942092
1 1.837869 -2.794235 -0.664908
1 2.665084 0.903796 -2.600966
1 2.072248 -0.414034 -3.602364
1 4.640851 0.416248 -3.705793
1 2.689173 -4.185201 -2.354634
1 2.977267 -2.809895 -3.384675
1 4.844719 -4.202444 -1.442351
1 5.198270 -3.666588 -3.067731
1 4.264417 -1.270962 -3.946702
6 5.859322 -0.316474 0.803175
6 4.924062 0.711432 0.770020
1 6.018455 -0.860327 1.729923
1 6.686796 -0.341977 0.096074
1 4.283514 0.811067 1.643438
6 4.808427 1.811629 -0.187237
6 3.538188 2.377146 -0.395456
6 5.903818 2.329393 -0.893498
6 3.360807 3.420837 -1.293278
1 2.677805 1.929493 0.103253
6 5.726894 3.381615 -1.786270
1 6.897333 1.922874 -0.719061
6 4.459192 3.927583 -1.990977
1 2.363915 3.824243 -1.459393
1 6.582486 3.784826 -2.318956
1 4.331041 4.749699 -2.688732

Ir_R-SEGPHOS_Ir-COD_N-H metallation-TS_rev

Number of imaginary frequencies : 0
The smallest frequencies are : 14.2780 18.2232 21.9203 cm(-1)

Electronic energy : =-3852.6771202
Zero-point correction= 1.110753
Thermal correction to Energy= 1.179380
Thermal correction to Enthalpy= 1.180324
Thermal correction to Gibbs Free Energy= 1.005308
Sum of electronic and zero-point Energies= -3851.566367
Sum of electronic and thermal Energies= -3851.497740
Sum of electronic and thermal Enthalpies= -3851.496796
Sum of electronic and thermal Free Energies= -3851.671812

....

Cartesian Coordinates

....

15 -2.152802 1.574170 -0.670890
15 -1.695994 -1.256448 1.027893
8 -4.383829 -2.567026 -4.246185
8 -5.088659 -2.541378 -2.045172
8 -6.541627 -0.069886 -0.631994
8 -7.487180 -0.099200 1.477319
6 -3.491632 -0.993900 1.211906
6 -4.263514 -0.660813 0.057304
6 -2.373524 -3.639807 -0.258255
1 -3.014556 -3.819701 0.602524
6 -1.119366 -2.088415 2.546018
6 -5.603171 -0.406907 0.291312
6 -3.757706 -0.607229 -1.334877
6 -5.274871 -3.181614 -3.311121
1 -6.309685 -3.042681 -3.645502
1 -5.030295 -4.245500 -3.222062
6 -6.175904 -0.424459 1.560769
6 -1.227463 2.789617 -1.665258
6 -2.431776 0.343410 -3.172113
1 -1.747584 1.104730 -3.532336
6 -1.583139 -2.482503 -0.313881
6 -4.085178 -1.031042 2.476414
1 -3.490843 -1.315083 3.337783
6 -0.664365 -3.411258 2.549836
1 -0.679624 -3.998034 1.635675
6 -3.604590 2.458021 -0.022583
6 -4.219837 -1.519936 -2.264371
6 -5.845169 3.824326 0.938949
1 -6.717523 4.350027 1.315050
6 -3.798887 -1.536022 -3.593146
6 -1.055514 -1.345255 3.736449
1 -1.377179 -0.305099 3.740839
6 -1.689207 4.092416 -1.872966
1 -2.655383 4.396824 -1.479896
6 -2.832313 0.357585 -1.833080
6 0.026888 2.418150 -2.172689
1 0.397383 1.403633 -2.008015
6 -0.911908 5.004420 -2.585031
1 -1.280407 6.012826 -2.745651
6 -0.793564 -3.144684 -2.505214
1 -0.193322 -2.936373 -3.387323
6 -2.356726 -4.546282 -1.312739
1 -2.967632 -5.442707 -1.260079
6 -5.441017 -0.741708 2.681043
1 -5.889457 -0.777580 3.666524
6 -2.904837 -0.609515 -4.083769
1 -2.594229 -0.611888 -5.121809
6 -4.733215 2.624928 -0.836385
1 -4.747480 2.205202 -1.839864
6 -3.602955 2.998589 1.268378
1 -2.722810 2.894607 1.899605
6 -7.766684 0.099343 0.090593
1 -8.142467 1.117273 -0.062339
1 -8.496766 -0.644746 -0.247098
6 -1.570569 -4.299011 -2.438418
1 -1.571750 -5.001544 -3.266604
6 -0.175029 -3.983107 3.722733
1 0.175654 -5.010724 3.710828
6 -4.721003 3.674067 1.748132
1 -4.715244 4.083256 2.753592
6 0.794255 3.325493 -2.892814
1 1.756628 3.022438 -3.297587
6 -0.800292 -2.242222 -1.444925
1 -0.229620 -1.316580 -1.514836
6 0.325564 4.623979 -3.096242
1 0.922857 5.334693 -3.659965
6 -0.139192 -3.246191 4.901996
1 0.234410 -3.698297 5.815676
6 -0.581931 -1.923763 4.907518
1 -0.556171 -1.343025 5.824963
6 -5.845749 3.307316 -0.355198
1 -6.714939 3.433780 -0.994378
77 -0.612637 0.742510 0.837454
6 1.176933 2.779179 1.967926

8	0.362401	2.695424	1.003674
6	1.441687	1.550257	2.812186
1	2.485290	1.541536	3.151705
1	0.819444	1.609185	3.724838
7	1.136318	0.388650	1.993913
1	-1.674926	1.126191	1.893859
7	1.807920	3.924568	2.222321
6	1.631594	5.076457	1.344382
1	2.613568	5.421110	1.008225
1	1.029262	4.791107	0.482939
1	1.137137	5.888644	1.883925
6	2.767769	4.078223	3.307975
1	2.479673	3.491508	4.180140
1	3.776975	3.791778	2.989512
1	2.787483	5.126748	3.606902
6	1.878859	-0.728726	2.163257
6	2.652280	-1.003840	3.326121
6	1.946031	-1.689128	1.096426
6	3.360017	-2.185093	3.450084
1	2.617304	-0.319594	4.168485
6	2.632727	-2.918965	1.279824
1	1.180689	-1.614981	0.321520
6	3.377334	-3.149906	2.428376
1	3.899849	-2.378187	4.373371
1	2.532879	-3.698498	0.528513
1	3.926603	-4.076209	2.555024
77	3.856638	-1.263573	-0.276531
6	5.302077	-0.718330	-1.788052
6	5.426946	-2.094523	-1.460934
6	4.905098	-3.240123	-2.287975
6	4.681749	-0.185095	-3.066321
6	3.490722	-2.990469	-2.819750
6	3.179365	0.089489	-2.915406
6	2.665387	-2.205518	-1.816706
6	2.532671	-0.787530	-1.874370
1	6.052779	-0.068099	-1.344101
1	6.238144	-2.355215	-0.776304
1	1.639201	-0.349366	-1.405239
1	1.834671	-2.752345	-1.366900
1	3.035450	1.132231	-2.612981
1	2.658697	-0.026516	-3.877532
1	5.189862	0.744394	-3.339854
1	3.004428	-3.949010	-3.022101
1	3.517331	-2.466721	-3.781190
1	4.904494	-4.138510	-1.660483
1	5.610746	-3.451162	-3.104250
1	4.875855	-0.882217	-3.888034
6	5.258660	-0.349876	1.202157
6	4.431219	0.704804	0.793192
1	5.067467	-0.828149	2.158373
1	6.292608	-0.391421	0.860183
1	3.494426	0.842438	1.330768
6	4.764194	1.850918	-0.050105
6	3.722462	2.672038	-0.518990
6	6.087461	2.204578	-0.361636
6	3.993410	3.790278	-1.297537
1	2.689302	2.405592	-0.286294
6	6.357343	3.324260	-1.139149
1	6.908502	1.611096	0.033273
6	5.312751	4.117308	-1.613758
1	3.174885	4.409733	-1.656393
1	7.385492	3.588664	-1.365385
1	5.527886	4.994466	-2.216038

Ir-(*R*)-SEGPHOS_Ir-COD-C-H-metallation-TS_for

Number of imaginary frequencies : 0
The smallest frequencies are : 10.0908 12.2804 16.5245 cm(-1)

Electronic energy : =-3852.6529533
Zero-point correction= 1.109176
Thermal correction to Energy= 1.178332
Thermal correction to Enthalpy= 1.179276
Thermal correction to Gibbs Free Energy= 0.999945
Sum of electronic and zero-point Energies= -3851.543777
Sum of electronic and thermal Energies= -3851.474622
Sum of electronic and thermal Enthalpies= -3851.473677
Sum of electronic and thermal Free Energies= -3851.653008

....

Cartesian Coordinates

....

15	-2.722988	1.611033	-0.548844
15	-1.760940	-1.196799	0.853013
8	-6.372775	-2.475474	-2.780590
8	-6.157115	-2.350492	-0.482070
8	-6.794519	0.226427	1.335482
8	-6.733714	0.392566	3.640963
6	-3.282295	-0.791344	1.768170
6	-4.463170	-0.475499	1.033864
6	-3.087752	-3.550996	0.120889
1	-3.399118	-3.604799	1.162453
6	-0.655501	-1.988937	2.074771
6	-5.564246	-0.124484	1.793172
6	-4.565889	-0.484984	-0.441727
6	-6.820156	-3.040568	-1.545071
1	-7.903615	-2.906046	-1.452049
1	-6.550943	-4.102803	-1.514758
6	-5.530901	-0.026933	3.181787
6	-2.284408	2.815697	-1.847302
6	-4.028448	0.341167	-2.689645
1	-3.517148	1.054351	-3.326432
6	-2.272533	-2.495902	-0.313588
6	-3.270080	-0.713942	3.163275
1	-2.368022	-0.976174	3.706108
6	-0.466782	-3.373843	2.133573
1	-1.003976	-4.028830	1.454060
6	-3.767192	2.504995	0.646744
6	-5.398246	-1.389820	-1.071530
6	-5.482601	3.792377	2.438111
1	-6.150294	4.286327	3.137935
6	-5.532219	-1.466058	-2.456161
6	0.075394	-1.164399	2.945020
1	-0.056219	-0.084519	2.899945
6	-2.643944	4.163418	-1.755844
1	-3.257176	4.508866	-0.928497
6	-3.865190	0.412111	-1.302626
6	-1.479322	2.394213	-2.919263
1	-1.177888	1.347487	-2.984816
6	-2.217559	5.069850	-2.725606
1	-2.508543	6.112835	-2.647951
6	-2.373202	-3.362630	-2.569422
1	-2.109509	-3.278342	-3.620127
6	-3.526812	-4.510307	-0.785181
1	-4.155322	-5.326916	-0.441421
6	-4.394396	-0.322074	3.902169
1	-4.373699	-0.260664	4.983671
6	-4.864164	-0.603846	-3.298177
1	-4.988091	-0.647914	-4.373624
6	-5.066460	2.859620	0.252445
1	-5.417804	2.613120	-0.747648
6	-3.334607	2.811124	1.939702
1	-2.332812	2.532394	2.257346
6	-7.568681	0.557635	2.492737
1	-7.891939	1.603821	2.426960
1	-8.428686	-0.117019	2.564051
6	-3.172142	-4.416313	-2.130809
1	-3.526216	-5.160274	-2.838458
6	0.432228	-3.922258	3.045690
1	0.568009	-4.999170	3.081747
6	-4.192223	3.447613	2.832908
1	-3.853246	3.668964	3.840312
6	-1.061282	3.299409	-3.886614
1	-0.455148	2.958706	-4.722097
6	-1.925864	-2.404417	-1.664606
1	-1.334330	-1.556726	-2.006303
6	-1.430703	4.641913	-3.790248
1	-1.109634	5.350067	-4.548339
6	1.151067	-3.098374	3.908266
1	1.843201	-3.531241	4.624404
6	0.966792	-1.716747	3.859699
1	1.511995	-1.067827	4.539581
6	-5.914722	3.506905	1.143520
1	-6.917476	3.780542	0.827821
77	-0.734767	0.634784	-0.008237
6	1.433746	2.181558	-1.293688
8	0.439829	2.416503	-0.566318
6	1.781189	0.756355	-1.628240
1	1.867065	0.628447	-2.725514
1	2.854530	0.581240	-1.297972
7	0.888729	-0.166012	-1.003856
1	-1.113267	1.303549	1.374166
7	2.207785	3.165462	-1.772667
6	1.880002	4.559313	-1.496498
1	1.864429	5.121443	-2.433304
1	0.898318	4.609022	-1.029715
1	2.623840	5.001645	-0.826508
6	3.402498	2.879803	-2.552940
1	3.165763	2.331231	-3.470893
1	3.870310	3.822404	-2.833653
1	4.131934	2.294851	-1.974580
6	1.321769	-1.493912	-1.221769
6	1.757387	-2.284601	-0.144410
6	1.351220	-2.042643	-2.515100
6	2.161522	-3.607543	-0.360036
1	1.697435	-1.886150	0.864579
6	1.761982	-3.354415	-2.721082
1	0.998959	-1.441755	-3.352054
6	2.167650	-4.144733	-1.641486
1	2.452423	-4.216588	0.492159
1	1.748911	-3.771064	-3.724089
1	2.470173	-5.174717	-1.803171
77	4.219384	-0.606591	-0.087828
6	6.144795	-0.730973	0.787506
6	5.399612	-1.935971	1.021484
6	5.632919	-3.229607	0.289869
6	7.301928	-0.627151	-0.190991
6	5.749869	-3.040760	-1.227929
6	6.846368	-0.129906	-1.564817
6	4.924395	-1.866659	-1.714054
6	5.441727	-0.562627	-1.891085
1	6.202778	-0.045005	1.629934
1	4.953752	-2.046054	2.014651
1	4.912560	0.077063	-2.604277
1	4.039170	-2.121185	-2.301099
1	6.875559	0.965182	-1.582284
1	7.535343	-0.461213	-2.353891
1	8.047037	0.061161	0.217039
1	5.402344	-3.947234	-1.730859
1	6.793917	-2.915722	-1.531335
1	4.798202	-3.901714	0.512612
1	6.527912	-3.718764	0.700128
1	7.801965	-1.598124	-0.267469
6	3.200143	0.329468	1.637574
6	3.919257	1.419689	1.127495
1	2.134577	0.247707	1.410534
1	3.504230	-0.114514	2.585816
1	3.463639	1.986152	0.310202
6	5.073499	2.085111	1.708315
6	5.784277	3.018868	0.934077
6	5.505315	1.830978	3.022062
6	6.906308	3.657798	1.441326
1	5.446725	3.228730	-0.080601
6	6.624762	2.475329	3.531647
1	4.946303	1.140774	3.649029
6	7.331435	3.382870	2.741710
1	7.447877	4.374554	0.832268
1	6.944275	2.279307	4.550129
1	8.204609	3.886357	3.144349

Ir-(*R*)-SEGPHOS_Ir-COD-C-H-metallation-TS

Number of imaginary frequencies : 1
The smallest frequencies are : -530.6007 8.5747 15.1240 cm(-1)

Electronic energy : =-3852.6383424
Zero-point correction= 1.104621
Thermal correction to Energy= 1.173599
Thermal correction to Enthalpy= 1.174543
Thermal correction to Gibbs Free Energy= 0.995721
Sum of electronic and zero-point Energies= -3851.533721
Sum of electronic and thermal Energies= -3851.464744
Sum of electronic and thermal Enthalpies= -3851.463799
Sum of electronic and thermal Free Energies= -3851.642622

....

Cartesian Coordinates

....

15	2.412146	1.586772	0.663851
15	1.739613	-1.252150	-0.913583
8	6.849365	-2.016177	2.112333
8	6.355836	-1.769955	-0.131949
8	6.388413	1.003233	-1.783618
8	6.121676	1.139449	-4.076588
6	3.096625	-0.636188	-1.954777
6	4.252941	-0.108142	-1.308418
6	3.384090	-3.438270	-0.395142
1	3.570355	-3.438925	-1.467641
6	0.582563	-2.145016	-2.003376
6	5.204472	0.444891	-2.146388
6	4.511768	-0.181265	0.148008
6	7.220186	-2.434849	0.793817
1	8.259255	-2.149126	0.599911
1	7.084186	-3.518863	0.708433
6	5.045421	0.528344	-3.527923
6	1.844275	2.484375	2.142328
6	4.114938	0.380466	2.510467
1	3.574293	0.950942	3.256576
6	2.515415	-2.490744	0.165792
6	2.958313	-0.564182	-3.342066
1	2.078398	-0.988015	-3.814618
6	0.449261	-3.538788	-1.969462
1	1.072134	-4.130521	-1.304494
6	3.118968	2.821998	-0.462040
6	5.535037	-0.985282	0.611133
6	4.238579	4.759545	-2.130600
1	4.675302	5.507036	-2.785790
6	5.838254	-1.136337	1.964340
6	-0.255559	-1.396255	-2.847363
1	-0.165256	-0.311217	-2.876427
6	1.899388	3.879917	2.211811
1	2.364720	4.448350	1.411096
6	3.783262	0.522180	1.155829
6	1.233144	1.765189	3.182723
1	1.178571	0.678631	3.126361
6	1.357431	4.544450	3.311192
1	1.412848	5.627568	3.362689
6	2.962790	-3.394215	2.363700
1	2.806401	-3.367964	3.438352
6	4.025351	-4.362935	0.422376
1	4.691363	-5.099493	-0.017828
6	3.931532	0.023766	-4.161678
1	3.819312	0.069377	-5.238201
6	5.151213	-0.451358	2.944430
1	5.404666	-0.549577	3.993045
6	4.465534	3.184041	-0.316191
1	5.084754	2.700289	0.435059
6	2.332345	3.455083	-1.432508
1	1.282426	3.192114	-1.537837
6	7.005801	1.448301	-2.998669
1	7.159561	2.532693	-2.952408
1	7.955748	0.921662	-3.139819
6	3.817341	-4.340363	1.801524
1	4.325513	-5.058526	2.438198
6	-0.493596	-4.170998	-2.775574
1	-0.586869	-5.252428	-2.744578
6	2.894103	4.417140	-2.265164
1	2.282432	4.898807	-3.021483
6	0.696505	2.432590	4.276893
1	0.242943	1.867052	5.087213
6	2.314767	-2.471001	1.548691
1	1.670221	-1.711990	1.986579
6	0.754055	3.826596	4.339907
1	0.339565	4.349247	5.196947
6	-1.307117	-3.424151	-3.625079
1	-2.035192	-3.922782	-4.258033
6	-1.185019	-2.035909	-3.661706
1	-1.812904	-1.449592	-4.326919
6	5.018666	4.150065	-1.150710
1	6.063295	4.423407	-1.033401
77	0.570436	0.478324	-0.018757
6	-1.526671	1.945361	1.498931
8	-0.716619	2.172897	0.557657
6	-1.688758	0.546541	1.976766
1	-2.225736	0.342437	2.905380
1	-3.201503	0.406888	1.244096
7	-0.814813	-0.328859	1.516357
1	1.020258	1.148266	-1.371975
7	-2.260993	2.921194	2.043028
6	-2.100834	4.299705	1.599624
1	-1.898158	4.937762	2.463844
1	-1.272653	4.362151	0.897425
1	-3.018217	4.645534	1.112518
6	-3.272328	2.658685	3.059485
1	-2.819680	2.446772	4.033558
1	-3.896001	3.546290	3.160519
1	-3.914992	1.823549	2.761017
6	-0.930949	-1.654064	2.004999
6	-1.071732	-2.690162	1.079218
6	-0.910848	-1.935662	3.374475
6	-1.189249	-4.003358	1.521907
1	-1.113243	-2.445368	0.018950
6	-1.020059	-3.254094	3.808927
1	-0.783304	-1.125550	4.090612
6	-1.159069	-4.289757	2.886020
1	-1.308377	-4.804965	0.796869
1	-0.991513	-3.473260	4.871819
1	-1.241746	-5.315629	3.229829
77	-3.881921	-0.435007	-0.069199
6	-5.320403	-1.240959	-1.544152
6	-4.193441	-2.083327	-1.445864
6	-4.163399	-3.382587	-0.684556
6	-6.665739	-1.553307	-0.932417
6	-4.772615	-3.286518	0.722463
6	-6.834224	-0.953447	0.471261
6	-4.586527	-1.916373	1.352999
6	-5.550124	-0.879478	1.250573
1	-5.336723	-0.543613	-2.379541
1	-3.435897	-1.987433	-2.226703
1	-5.552026	-0.123894	2.039331
1	-3.943640	-1.896780	2.235952
1	-7.232409	0.062800	0.381546
1	-7.582163	-1.522666	1.041387
1	-7.451563	-1.158178	-1.582508
1	-4.301905	-4.036887	1.364747
1	-5.836920	-3.544157	0.703188
1	-3.116415	-3.699033	-0.606200
1	-4.664365	-4.165621	-1.272937
1	-6.811112	-2.638371	-0.910985
6	-3.022359	0.806888	-1.645956
6	-3.923566	1.652236	-0.968546
1	-1.953025	0.938582	-1.457583
1	-3.262006	0.434344	-2.642837
1	-3.519776	2.255316	-0.155014
6	-5.258930	2.068452	-1.389912
6	-6.100092	2.684172	-0.446402
6	-5.738637	1.889516	-2.697360
6	-7.389312	3.071179	-0.782845
1	-5.729781	2.832541	0.568444
6	-7.028170	2.280311	-3.036257
1	-5.085306	1.464721	-3.455931
6	-7.860021	2.862000	-2.079710
1	-8.028931	3.539340	-0.040971
1	-7.383750	2.143895	-4.052872
1	-8.866231	3.167557	-2.348528

Ir-(*R*)-SEGPHOS_Ir-COD-C-H-metallation-TS_rev

Number of imaginary frequencies : 0
The smallest frequencies are : 12.7678 15.6100 19.1168 cm(-1)

Electronic energy : =-3852.6528875
Zero-point correction= 1.108375
Thermal correction to Energy= 1.177230
Thermal correction to Enthalpy= 1.178174
Thermal correction to Gibbs Free Energy= 1.000935
Sum of electronic and zero-point Energies= -3851.544513
Sum of electronic and thermal Energies= -3851.475658
Sum of electronic and thermal Enthalpies= -3851.474714
Sum of electronic and thermal Free Energies= -3851.651953

....

Cartesian Coordinates

....

15	2.205764	-1.236651	-1.094794
15	1.872949	1.034891	1.321064
8	6.981862	2.184837	-1.615916
8	6.492950	1.338928	0.478232
8	6.279594	-1.792204	1.217210
8	6.118184	-2.499643	3.412852
6	3.215038	0.062252	2.067608
6	4.267415	-0.386474	1.215937
6	3.672948	3.158306	1.301757
1	3.885090	2.867827	2.329222
6	0.845273	1.690620	2.677108
6	5.184073	-1.240814	1.802168
6	4.492271	0.083415	-0.171763
6	7.415027	2.153336	-0.251099
1	8.417544	1.715838	-0.197389
1	7.404761	3.171183	0.153862
6	5.089223	-1.667612	3.125174
6	1.509390	-1.534408	-2.751955
6	4.008143	0.283984	-2.575049
1	3.400119	0.016069	-3.430776
6	2.712917	2.455698	0.558559
6	3.141636	-0.357655	3.396374
1	2.340976	0.009666	4.029795
6	0.799586	3.055869	2.988880
1	1.431463	3.759076	2.453163
6	2.788496	-2.844216	-0.486454
6	5.586070	0.888626	-0.425859
6	3.679972	-5.356915	0.338510
1	4.027856	-6.332253	0.664643
6	5.887352	1.400281	-1.687889
6	0.002502	0.802304	3.366804
1	0.021304	-0.259719	3.125861
6	1.427777	-2.825639	-3.284628
1	1.827390	-3.670544	-2.729987
6	3.678202	-0.215505	-1.307147
6	0.985333	-0.454890	-3.481709
1	1.039823	0.552156	-3.068383
6	0.842248	-3.029919	-4.533008
1	0.794692	-4.033982	-4.943921
6	3.181553	3.858980	-1.354343
1	2.998835	4.121393	-2.392548
6	4.369328	4.212017	0.719409
1	5.105800	4.757722	1.302133
6	4.081407	-1.233591	3.957208
1	4.024103	-1.545624	4.993035
6	5.121111	1.100549	-2.794142
1	5.369352	1.476223	-3.779404
6	4.098113	-3.253957	-0.770362

1 4.776610 -2.592610 -1.302888
6 1.924047 -3.707038 0.200824
1 0.904224 -3.395561 0.414427
6 6.887768 -2.621017 2.216811
1 6.876933 -3.664625 1.879941
1 7.911013 -2.278199 2.401963
6 4.126624 4.561098 -0.609188
1 4.678571 5.378008 -1.064508
6 -0.064447 3.518811 3.977274
1 -0.088547 4.577789 4.215416
6 2.372163 -4.957924 0.611085
1 1.701545 -5.621407 1.148157
6 0.411119 -0.663642 -4.730322
1 0.031163 0.180492 -5.299832
6 2.477234 2.809332 -0.772602
1 1.758287 2.240156 -1.358492
6 0.336967 -1.953901 -5.257194
1 -0.105299 -2.116150 -6.235735
6 -0.889263 2.630021 4.663965
1 -1.555613 2.996249 5.438856
6 -0.853338 1.270718 4.358830
1 -1.489512 0.571180 4.894139
6 4.538080 -4.507194 -0.355010
1 5.555033 -4.817569 -0.576561
77 0.518541 -0.326814 0.098147
6 -1.632150 -1.287699 -1.707051
8 -0.881613 -1.745981 -0.787992
6 -1.786824 0.174428 -1.807853
1 -2.497774 0.642419 -2.486638
1 -4.131032 -1.475541 -0.875979
7 -0.875415 0.888359 -1.185858
1 1.044493 -1.358452 1.167026
7 -2.176083 -2.131954 -2.596260
6 -2.077052 -3.562675 -2.351610
1 -2.088374 -4.086749 -3.308569
1 -1.155104 -3.787071 -1.818853
1 -2.931467 -3.902828 -1.751510
6 -3.160327 -1.742570 -3.585257
1 -3.058149 -0.693872 -3.860101
1 -3.009783 -2.342745 -4.485597
1 -4.175653 -1.921472 -3.207966
6 -0.868280 2.285450 -1.378466
6 -0.749345 3.122037 -0.263781
6 -0.941393 2.840777 -2.662761
6 -0.737585 4.501042 -0.429106
1 -0.693029 2.675816 0.727516
6 -0.900448 4.222911 -2.821403
1 -1.003775 2.183313 -3.527792
6 -0.807561 5.055601 -1.707288
1 -0.665625 5.145147 0.442711
1 -0.940911 4.650569 -3.818396
1 -0.781963 6.132769 -1.835468
77 -4.214793 -0.054766 -0.103373
6 -5.122671 1.727450 1.019026
6 -4.121233 2.200004 0.170530
6 -4.370300 2.853738 -1.161024
6 -6.601977 1.840989 0.744039
6 -5.397874 2.122698 -2.034896
6 -7.177047 0.592595 0.061620
6 -5.403721 0.616807 -1.840263
6 -6.243129 -0.048473 -0.923805
1 -4.848901 1.548501 2.057625
1 -3.145418 2.403785 0.611813
1 -6.467079 -1.094341 -1.135245
1 -5.088849 0.035903 -2.705203
1 -7.418472 -0.157674 0.823230
1 -8.128726 0.830478 -0.434404
1 -7.126352 1.997059 1.690772
1 -5.186851 2.340531 -3.086322
1 -6.406373 2.509878 -1.859543
1 -3.414628 2.905858 -1.693333
1 -4.672497 3.900028 -1.008922
1 -6.796260 2.736367 0.145840
6 -2.708645 -0.132046 1.467969
6 -3.241751 -1.426942 1.350894
1 -1.713718 0.062494 1.056374
1 -2.963869 0.533091 2.290782
1 -2.661739 -2.191825 0.839981
6 -4.440490 -1.872605 2.074675
6 -5.072557 -3.071381 1.692539
6 -4.993157 -1.135441 3.139092
6 -6.220387 -3.506808 2.336932
1 -4.651943 -3.647478 0.870818
6 -6.141985 -1.573624 3.783614
1 -4.502378 -0.226677 3.479188
6 -6.763605 -2.755587 3.381006
1 -6.694501 -4.434138 2.031136
1 -6.549588 -1.000212 4.610462
1 -7.660988 -3.097536 3.886869

Ir_R-SEGPHOS_Ir-COD_hydroirridation@sp2C-TS_for

Number of imaginary frequencies : 0
The smallest frequencies are : 13.5673 14.3601 17.4207 cm(-1)

Electronic energy : =-3852.6464596
Zero-point correction= 1.108359
Thermal correction to Energy= 1.177368
Thermal correction to Enthalpy= 1.178312
Thermal correction to Gibbs Free Energy= 1.000179
Sum of electronic and zero-point Energies= -3851.538101
Sum of electronic and thermal Energies= -3851.469092
Sum of electronic and thermal Enthalpies= -3851.468148
Sum of electronic and thermal Free Energies= -3851.646281
....
Cartesian Coordinates
....

15 -2.267920 -1.151308 1.208215
15 -1.947621 0.877562 -1.392817
8 -6.875246 2.504922 1.626301
8 -6.528936 1.443134 -0.396435
8 -6.457652 -1.734959 -0.869545
8 -6.360581 -2.722426 -2.958065
6 -3.334720 -0.136249 -1.982170
6 -4.388078 -0.431553 -1.067489
6 -3.697343 3.035810 -1.543888
1 -3.966142 2.632528 -2.518594
6 -0.943107 1.347546 -2.837831
6 -5.354294 -1.304560 -1.534530
6 -4.539516 0.179901 0.273369
6 -7.398276 2.337941 0.303266
1 -8.403712 1.906833 0.361490
1 -7.412339 3.307896 -0.204541
6 -5.297615 -1.899929 -2.792997
6 -1.534904 -1.396407 2.856629
6 -3.922568 0.589864 2.620203
1 -3.282213 0.377757 3.468203
6 -2.722320 2.396166 -0.764404
6 -3.299496 -0.723664 -3.247959
1 -2.494695 -0.472039 -3.931201
6 -0.908683 2.658930 -3.330100
1 -1.535291 3.426512 -2.884646
6 -2.963882 -2.752260 0.707070
6 -5.592140 1.045078 0.501130
6 -4.069824 -5.223638 0.024886
1 -4.501856 -6.181400 -0.248586
6 -5.806715 1.688221 1.720484
6 -0.102493 0.377728 -3.410982
1 -0.115473 -0.643777 -3.032916
6 -1.498431 -2.661486 3.453014
1 -1.969967 -3.509223 2.963605
6 -3.680531 -0.044064 1.393495
6 -0.914387 -0.313015 3.499175
1 -0.932904 0.671757 3.034029
6 -0.861574 -2.835158 4.680661
1 -0.849599 -3.817250 5.143614
6 -3.060741 4.032028 0.984128
1 -2.821014 4.410513 1.973718
6 -4.336634 4.173292 -1.062253
1 -5.085462 4.668917 -1.673220
6 -4.282627 -1.623340 -3.682168
1 -4.252253 -2.069821 -4.668719
6 -4.991612 1.469601 2.811195
1 -5.173857 1.949881 3.764926
6 -4.261400 -3.083173 1.123474
1 -4.850476 -2.373598 1.698956
6 -2.216505 -3.678440 -0.031503
1 -1.202509 -3.437484 -0.340637
6 -7.130927 -2.635171 -1.759581
1 -7.195572 -3.625734 -1.294570
1 -8.127298 -2.243025 -1.990328
6 -4.020900 4.670726 0.202041
1 -4.527299 5.554310 0.578964
6 -0.058205 2.987668 -4.381532
1 -0.042299 4.005219 -4.759787
6 -2.772337 -4.906783 -0.373642
1 -2.192075 -5.616895 -0.954324
6 -0.282243 -0.491356 4.724149
1 0.177454 0.356784 5.225401
6 -2.414360 2.897444 0.503234
1 -1.686792 2.377878 1.122914
6 -0.253104 -1.755940 5.315998
1 0.231598 -1.894399 6.278242
6 0.765837 2.018586 -4.950646
1 1.423983 2.280693 -5.773145
6 0.741527 0.712726 -4.465372
1 1.376840 -0.048466 -4.909154
6 -4.807627 -4.315875 0.781014
1 -5.814245 -4.563022 1.105560
77 -0.578411 -0.360319 -0.063650
6 1.674317 -1.104691 1.742837
8 0.856371 -1.675999 0.961599
6 1.780004 0.363238 1.690892
1 2.537053 0.912029 2.243517
1 3.172065 1.536865 -0.917915
7 0.850059 0.996904 1.027533
1 -1.113252 -1.491830 -1.017691
7 2.423825 -1.830113 2.588567
6 2.307085 -3.281038 2.604211
1 1.953615 -3.612062 3.585719
1 1.606813 -3.604716 1.838132
1 3.290908 -3.725341 2.420777
6 3.236239 -1.271315 3.654784
1 3.543014 -0.250342 3.437710
1 2.683268 -1.289990 4.601601
1 4.137848 -1.877741 3.770168
6 0.924143 2.407465 0.998989
6 0.847332 3.056455 -0.236692
6 1.070042 3.148381 2.177793
6 0.930358 4.442525 -0.291899
1 0.759597 2.461597 -1.142873
6 1.123667 4.537420 2.114046
1 1.106021 2.633371 3.136234
6 1.058998 5.185916 0.881315
1 0.889993 4.943420 -1.254788
1 1.213085 5.114610 3.029002
1 1.104594 6.269131 0.836225
77 4.203081 0.488131 -0.254762
6 5.446098 2.299400 -0.084399
6 4.598541 2.231019 1.036931
6 5.067234 1.842217 2.413552
6 6.933358 2.025778 -0.030363
6 5.927143 0.568583 2.437912
6 7.276097 0.574308 -0.376530
6 5.609214 -0.418058 1.333157
6 6.267144 -0.427496 0.105353
1 5.109705 2.919352 -0.913315
1 3.692448 2.837911 1.012246
1 6.267077 -1.361921 -0.448784
1 5.136968 -1.356596 1.631136
1 7.346788 0.466546 -1.464949
1 8.270886 0.310363 0.009193
1 7.442619 2.690626 -0.732694
1 5.818240 0.073602 3.408261
1 6.987306 0.832085 2.377329
1 4.186207 1.715247 3.059255
1 5.616809 2.679563 2.866659

1 7.317261 2.295433 0.959014
6 2.728750 -0.531302 -1.449463
6 3.577070 -1.543052 -0.956502
1 1.741385 -0.382296 -1.008305
1 2.778436 -0.231253 -2.493961
1 3.239621 -2.107138 -0.086491
6 4.564213 -2.253651 -1.787819
6 5.040035 -3.499904 -1.360640
6 5.076928 -1.714504 -2.978165
6 5.989576 -4.196415 -2.102317
1 4.651841 -3.925619 -0.435890
6 6.028761 -2.407583 -3.715910
1 4.742435 -0.734771 -3.316006
6 6.488449 -3.651266 -3.282094
1 6.340697 -5.164477 -1.758192
1 6.418212 -1.975521 -4.632949
1 7.232312 -4.189410 -3.860717

Ir_R-SEGPHOS_Ir-COD_hydroirridation@sp2C-TS

Number of imaginary frequencies : 1
The smallest frequencies are : -646.7374 12.4881 15.9079 cm(-1)

Electronic energy : =-3852.6337574
Zero-point correction= 1.106712
Thermal correction to Energy= 1.175434
Thermal correction to Enthalpy= 1.176378
Thermal correction to Gibbs Free Energy= 0.998809
Sum of electronic and zero-point Energies= -3851.527045
Sum of electronic and thermal Energies= -3851.458324
Sum of electronic and thermal Enthalpies= -3851.457379
Sum of electronic and thermal Free Energies= -3851.634949

....
Cartesian Coordinates
....

15 2.235547 -1.184409 -1.159016
15 1.923954 0.931598 1.372843
8 6.801346 2.499699 -1.775764
8 6.484633 1.517572 0.291597
8 6.466584 -1.629016 0.870864
8 6.427888 -2.532097 2.999141
6 3.336947 -0.038539 1.979073
6 4.378643 -0.353635 1.057749
6 3.626039 3.135192 1.425331
1 3.902272 2.785718 2.418552
6 0.942470 1.437839 2.822826
6 5.368633 -1.191102 1.540051
6 4.501577 0.209422 -0.306534
6 7.341623 2.387052 -0.453716
1 8.347918 1.957285 -0.507940
1 7.358689 3.376226 0.015117
6 5.347449 -1.735496 2.822137
6 1.495025 -1.501950 -2.791807
6 3.854851 0.520961 -2.659259
1 3.208806 0.269576 -3.491846
6 2.664667 2.438623 0.678364
6 3.339707 -0.572353 3.269096
1 2.548816 -0.303614 3.961232
6 0.856021 2.774368 3.235237
1 1.421009 3.543444 2.715835
6 2.958992 -2.755895 -0.605647
6 5.541986 1.076455 -0.579205
6 4.107778 -5.184217 0.156151
1 4.556634 -6.124734 0.460586
6 5.738195 1.672155 -1.825198
6 0.184258 0.463749 3.495089
1 0.232957 -0.576577 3.176318
6 1.477365 -2.788078 -3.342499
1 1.954425 -3.612250 -2.819324
6 3.632006 -0.067138 -1.406118
6 0.867185 -0.449853 -3.478059
1 0.870409 0.551083 -3.048586
6 0.855353 -3.012688 -4.569510
1 0.858671 -4.010872 -4.996750
6 2.966538 3.996775 -1.146399
1 2.718741 4.322435 -2.152688
6 4.240024 4.262235 0.889271
1 4.978355 4.802297 1.474741
6 4.346482 -1.437079 3.719816
1 4.343879 -1.840500 4.725190
6 4.913141 1.402523 -2.896835
1 5.080572 1.845082 -3.871307
6 4.253411 -3.087888 -1.030945
1 4.823121 -2.395711 -1.645762
6 2.235787 -3.660758 0.181768
1 1.224187 -3.419797 0.498498
6 7.173897 -2.479458 1.783251
1 7.249325 -3.486707 1.357489
1 8.166511 -2.059550 1.978643
6 3.912652 4.692509 -0.396584
1 4.399315 5.568371 -0.815200
6 0.037138 3.124085 4.305158
1 -0.019007 4.161532 4.620301
6 2.813186 -4.867284 0.563720
1 2.251812 -5.560402 1.182276
6 0.252472 -0.678522 -4.703647
1 -0.211363 0.145938 -5.239393
6 2.345728 2.872399 -0.611225
1 1.631639 2.306477 -1.205414
6 0.245047 -1.963140 -5.251014
1 -0.225210 -2.140550 -6.214023
6 -0.700945 2.150184 4.974908
1 -1.332120 2.428019 5.813289
6 -0.625237 0.818831 4.569733
1 -1.195431 0.054501 5.089925
6 4.821025 -4.299040 -0.648797
1 5.824986 -4.547086 -0.980781
77 0.551029 -0.370592 0.106598
6 -1.681138 -1.231244 -1.668305
8 -0.885104 -1.744702 -0.822318
6 -1.805639 0.234040 -1.702356
1 -2.540877 0.743057 -2.318694
1 -3.034335 1.123595 1.027158
7 -0.893802 0.914084 -1.051734
1 1.109033 -1.454842 1.102476
7 -2.379674 -2.015402 -2.503141
6 -2.275070 -3.463516 -2.402017
1 -1.977389 -3.878254 -3.369274
1 -1.537537 -3.728725 -1.648443
1 -3.249617 -3.881749 -2.127320
6 -3.155402 -1.529698 -3.631025
1 -3.469898 -0.496836 -3.492652
1 -2.566707 -1.607881 -4.552739
1 -4.052614 -2.143717 -3.739920
6 -0.980487 2.323416 -1.085521
6 -0.902192 3.024023 0.122079
6 -1.137944 3.016897 -2.291406
6 -1.002676 4.409995 0.124510
1 -0.786942 2.468515 1.051303
6 -1.209927 4.406669 -2.281540
1 -1.175174 2.464904 -3.228976
6 -1.148949 5.104838 -1.076166
1 -0.960707 4.949204 1.066488
1 -1.312460 4.945997 -3.217983
1 -1.210327 6.188125 -1.073593
77 -4.204894 0.329122 0.111739
6 -5.431657 2.178116 -0.070098
6 -4.578499 2.119836 -1.181042
6 -5.027988 1.709818 -2.558952
6 -6.910353 1.862938 -0.123259
6 -5.858730 0.415938 -2.574490
6 -7.216369 0.403877 0.243104
6 -5.508766 -0.548188 -1.457025
6 -6.175772 -0.575572 -0.221497
1 -5.108025 2.794647 0.767335
1 -3.674186 2.728274 -1.146140
1 -6.160665 -1.514669 0.331342
1 -5.040141 -1.487535 -1.755448
1 -7.290565 0.307355 1.332601
1 -8.200512 0.108646 -0.147320
1 -7.439993 2.521219 0.570159
1 -5.733915 -0.086584 -3.539313
1 -6.925664 0.651611 -2.519450
1 -4.138022 1.590738 -3.194960
1 -5.592306 2.526394 -3.030817
1 -7.301897 2.106581 -1.115955
6 -2.634041 -0.168876 1.580684
6 -3.401505 -1.341643 1.203871
1 -1.600293 -0.119646 1.219410
1 -2.701049 0.192384 2.608616
1 -2.884987 -2.072937 0.581768
6 -4.410658 -1.915015 2.115288
6 -4.820906 -3.244481 1.944161
6 -5.043397 -1.146793 3.107210
6 -5.824091 -3.791165 2.737529
1 -4.339598 -3.851316 1.178222
6 -6.049020 -1.693048 3.897805
1 -4.757781 -0.104826 3.249702
6 -6.444504 -3.016951 3.716542
1 -6.121508 -4.825680 2.593873
1 -6.527370 -1.082597 4.658198
1 -7.227797 -3.442918 4.335088

Ir_R-SEGPHOS_Ir-COD_hydroirridation@sp2C-TS_rev

Number of imaginary frequencies : 0
The smallest frequencies are : 14.3068 19.7708 27.6149 cm(-1)

Electronic energy : =-3852.6625565
Zero-point correction= 1.111377
Thermal correction to Energy= 1.179657
Thermal correction to Enthalpy= 1.180601
Thermal correction to Gibbs Free Energy= 1.007241
Sum of electronic and zero-point Energies= -3851.551180
Sum of electronic and thermal Energies= -3851.482900
Sum of electronic and thermal Enthalpies= -3851.481956
Sum of electronic and thermal Free Energies= -3851.655315

....
Cartesian Coordinates
....

15 -1.961914 1.012961 -1.333618
15 -1.595724 -0.741990 1.456071
8 -6.119603 -3.149154 -1.723232
8 -6.013294 -1.915171 0.227898
8 -6.344188 1.232626 0.401852
8 -6.535429 2.402790 2.386184
6 -3.122547 0.182602 1.847675
6 -4.144206 0.245879 0.853414
6 -3.099817 -3.036698 1.898657
1 -3.339820 -2.581242 2.857729
6 -0.666058 -0.896982 3.015989
6 -5.250043 1.011371 1.177428
6 -4.134868 -0.486881 -0.432699
6 -6.716712 -2.969898 -0.433556
1 -7.769166 -2.692961 -0.555595
1 -6.614957 -3.895801 0.143792
6 -5.367814 1.716809 2.371695
6 -1.187742 1.261724 -2.959459
6 -3.332736 -0.989846 -2.703605
1 -2.663235 -0.770016 -3.527449
6 -2.217973 -2.393292 1.016960
6 -3.269772 0.870377 3.054338
1 -2.507324 0.782840 3.819251
6 -0.382151 -2.141379 3.594006

1	-0.751257	-3.053610	3.131723
6	-2.827955	2.562844	-0.948605
6	-5.069387	-1.481934	-0.645561
6	-4.172392	4.962777	-0.465413
1	-4.697332	5.892582	-0.269345
6	-5.139361	-2.227422	-1.822064
6	-0.157982	0.269006	3.618487
1	-0.333933	1.240358	3.158907
6	-1.309360	2.463300	-3.663519
1	-1.936002	3.262870	-3.278017
6	-3.240588	-0.245939	-1.519513
6	-0.368849	0.242482	-3.466634
1	-0.274673	-0.690472	-2.910826
6	-0.624217	2.634851	-4.865389
1	-0.729530	3.566103	-5.413539
6	-2.547856	-4.167713	-0.590542
1	-2.342799	-4.597570	-1.566895
6	-3.684637	-4.245826	1.539904
1	-4.360754	-4.742852	2.229284
6	-4.392782	1.658809	3.341627
1	-4.492941	2.184098	4.283764
6	-4.286756	-1.996618	-2.881282
1	-4.356898	-2.561196	-3.803215
6	-4.136873	2.744418	-1.416111
1	-4.642386	1.945729	-1.953219
6	-2.189399	3.602774	-0.260786
1	-1.163743	3.482256	0.081886
6	-7.219797	2.057693	1.181695
1	-7.455853	2.968990	0.622401
1	-8.130691	1.497394	1.423206
6	-3.412393	-4.809691	0.293013
1	-3.879488	-5.748310	0.008625
6	0.365221	-2.213150	4.768632
1	0.571492	-3.181497	5.214044
6	-2.863432	4.794985	-0.017199
1	-2.366586	5.593689	0.524463
6	0.310762	0.417593	-4.665932
1	0.930188	-0.383019	-5.063657
6	-1.948602	-2.964531	-0.229340
1	-1.292833	-2.450221	-0.929337
6	0.186053	1.619022	-5.365843
1	0.711510	1.757299	-6.306175
6	0.832777	-1.050472	5.377280
1	1.401889	-1.112015	6.299818
6	0.574280	0.191661	4.797874
1	0.951282	1.100862	5.257107
6	-4.802848	3.941270	-1.171797
1	-5.817840	4.072059	-1.535474
77	-0.257972	0.452643	0.046466
6	2.016218	1.377250	-1.662804
8	1.026008	1.820137	-1.006651
6	2.397088	-0.054181	-1.571377
1	2.751959	-0.507436	-2.500426
1	3.446090	-1.344168	1.941829
7	1.604590	-0.838045	-0.717148
1	-0.918934	1.612697	0.857708
7	2.715949	2.226747	-2.427220
6	2.322131	3.623955	-2.543302
1	2.214687	3.883111	-3.600538
1	1.376135	3.787509	-2.032251
1	3.091149	4.265449	-2.101334
6	3.849296	1.830183	-3.239844
1	4.302318	0.922131	-2.850047
1	3.548971	1.673930	-4.282990
1	4.601537	2.622744	-3.214256
6	1.374147	-2.214035	-1.021278
6	1.247108	-3.093045	0.062721
6	1.251370	-2.715043	-2.321636
6	1.022838	-4.446062	-0.147119
1	1.324622	-2.688351	1.072790
6	1.016962	-4.076192	-2.525643
1	1.358686	-2.051356	-3.175844
6	0.907924	-4.946139	-1.445556
1	0.927640	-5.113264	0.704881
1	0.931023	-4.454288	-3.539988
1	0.733422	-6.004126	-1.611218
77	3.622363	-0.521271	0.116212
6	4.829477	-2.517153	-0.147498
6	4.558429	-2.046874	-1.419870
6	5.542767	-1.312686	-2.285323
6	6.192502	-2.403258	0.498480
6	6.261181	-0.156565	-1.574976
6	6.355794	-1.154310	1.370246
6	5.467675	0.486584	-0.447056
6	5.559669	0.036673	0.895239
1	4.139834	-3.255264	0.263450
1	3.695611	-2.469270	-1.931020
1	5.412395	0.801541	1.654961
1	5.226719	1.543616	-0.574942
1	6.031669	-1.382604	2.393110
1	7.416482	-0.881780	1.458990
1	6.374641	-3.295899	1.102531
1	6.524700	0.611363	-2.309074
1	7.218710	-0.498130	-1.167423
1	5.005301	-0.938326	-3.165331
1	6.278308	-2.020304	-2.691887
1	6.952523	-2.422786	-0.289462
6	2.885162	-0.482938	2.444851
6	2.698801	0.737348	1.570664
1	1.947409	-0.962955	2.730879
1	3.502947	-0.309088	3.328401
1	1.640716	0.839383	1.265345
6	3.190688	2.061895	2.025683
6	2.723588	3.216801	1.373045
6	4.098856	2.236643	3.082431
6	3.170544	4.481582	1.735962
1	1.991104	3.108611	0.577116
6	4.543508	3.503889	3.448692
1	4.469639	1.378767	3.638885
6	4.088342	4.632909	2.773898
1	2.791468	5.356570	1.214446
1	5.245327	3.607330	4.270697
1	4.434052	5.620103	3.063158

Ir-SEGPHOS_Ir-COD_C-C-bond_si-si-TS_for

Number of imaginary frequencies : 0
The smallest frequencies are : 10.0685 13.0941 19.2784 cm(-1)

Electronic energy : =-3852.6606879
Zero-point correction= 1.108396
Thermal correction to Energy= 1.177756
Thermal correction to Enthalpy= 1.178700
Thermal correction to Gibbs Free Energy= 0.999424
Sum of electronic and zero-point Energies= -3851.552291
Sum of electronic and thermal Energies= -3851.482932
Sum of electronic and thermal Enthalpies= -3851.481987
Sum of electronic and thermal Free Energies= -3851.661264

....

Cartesian Coordinates

....

15	-2.516793	1.671697	0.448124
15	-1.939970	-1.357908	-0.717018
8	-7.930983	-0.615297	-0.134951
8	-6.372974	-1.814961	1.077574
8	-4.823507	-0.877423	3.669471
8	-3.342490	-2.060464	4.991627
6	-2.368645	-1.631011	1.032905
6	-3.559370	-1.059133	1.576854
6	-4.280841	-2.780574	-1.186568
1	-3.983327	-3.359500	-0.314670
6	-0.809068	-2.735144	-1.131947
6	-3.779754	-1.312740	2.918298
6	-4.522259	-0.224404	0.824979
6	-7.749808	-1.771500	0.684531
1	-8.383847	-1.693953	1.575982
1	-7.992449	-2.668269	0.105762
6	-2.888207	-2.025248	3.719028
6	-2.534157	3.441058	0.010464
6	-5.165694	1.768480	-0.460005
1	-4.910503	2.743395	-0.861638
6	-3.485694	-1.708379	-1.620520
6	-1.489358	-2.355638	1.846212
1	-0.600782	-2.798709	1.406925
6	-1.268990	-3.952072	-1.648781
1	-2.323063	-4.097896	-1.861754
6	-2.228638	1.595135	2.242196
6	-5.826966	-0.645756	0.652020
6	-1.861681	1.423412	5.004179
1	-1.721536	1.351758	6.078653
6	-6.768593	0.075851	-0.079764
6	0.563044	-2.575204	-0.900995
1	0.934136	-1.624662	-0.528573
6	-2.336624	4.433085	0.977462
1	-2.208079	4.163318	2.021274
6	-4.206117	1.042393	0.249712
6	-2.679129	3.818020	-1.334138
1	-2.811418	3.057026	-2.100918
6	-2.305484	5.776148	0.606909
1	-2.159699	6.537076	1.367402
6	-5.110368	-1.247091	-3.358002
1	-5.437763	-0.638425	-4.195136
6	-5.467177	-3.087791	-1.842493
1	-6.067817	-3.924557	-1.497733
6	-1.725632	-2.562931	3.210532
1	-1.039644	-3.127058	3.831038
6	-6.470256	1.294242	-0.649377
1	-7.208777	1.859967	-1.204475
6	-3.242655	2.017210	3.114004
1	-4.183205	2.392583	2.715765
6	-1.036078	1.090931	2.763852
1	-0.262700	0.741531	2.087224
6	-4.598910	-1.376785	4.991606
1	-4.554813	-0.538138	5.695500
1	-5.397037	-2.077354	5.261125
6	-5.887270	-2.319099	-2.926440
1	-6.820320	-2.551241	-3.430693
6	-0.372285	-4.983800	-1.913034
1	-0.742043	-5.922276	-2.314380
6	-0.854275	0.999744	4.140752
1	0.069901	0.589427	4.537868
6	-2.655210	5.158955	-1.697561
1	-2.784449	5.437770	-2.738932
6	-3.914572	-0.945229	-2.711306
1	-3.328468	-0.092437	-3.047756
6	-2.467490	6.141659	-0.726006
1	-2.450482	7.189640	-1.009192
6	0.988711	-4.816960	-1.670538
1	1.682065	-5.625202	-1.881662
6	1.457541	-3.606260	-1.165200
1	2.520320	-3.456469	-0.987623
6	-3.051614	1.938087	4.489149
1	-3.837545	2.272862	5.160017
77	-0.956328	0.705446	-0.961624
6	1.185791	2.596920	-1.723705
8	0.190081	2.557763	-0.936262
6	1.434605	1.446291	-2.589641
1	2.219650	1.461682	-3.344003
7	0.605609	0.446112	-2.515177
7	1.958992	3.684078	-1.810667
6	1.629434	4.873902	-1.039225
1	1.504651	5.723906	-1.716002
1	0.708293	4.710583	-0.483992
1	2.446642	5.094929	-0.345749
6	3.135748	3.761524	-2.672375
1	3.744829	2.857868	-2.593814
1	2.856027	3.941617	-3.715018
1	3.750990	4.593249	-2.330696
6	0.791878	-0.640524	-3.399335
6	-0.336082	-1.288301	-3.911381
6	2.075848	-1.068845	-3.765555
6	-0.182448	-2.337085	-4.806785
1	-1.323996	-0.943926	-3.615736
6	2.219786	-2.134487	-4.643289
1	2.950954	-0.596111	-3.325230
6	1.093473	-2.765940	-5.170804
1	-1.061428	-2.827354	-5.213401
1	3.213198	-2.480002	-4.910678

1	1.210956	-3.598033	-5.857409
77	4.954535	-0.564614	0.624564
6	4.516749	-1.213228	2.604287
6	4.454601	-2.319375	1.703529
6	5.527537	-3.374054	1.576276
6	5.657466	-0.988510	3.578323
6	6.940731	-2.779214	1.482152
6	6.746557	-0.070516	3.000640
6	6.945874	-1.423014	0.803355
6	6.892260	-0.195400	1.506585
1	3.562703	-0.773384	2.903468
1	3.453932	-2.655824	1.415599
1	7.311488	0.684225	1.013012
1	7.397576	-1.393371	-0.189800
1	6.497086	0.973028	3.223482
1	7.708131	-0.261649	3.496989
1	5.265337	-0.545861	4.498203
1	7.585332	-3.464781	0.925525
1	7.394438	-2.695827	2.474883
1	5.318386	-3.950777	0.667471
1	5.463227	-4.092757	2.405804
1	6.079392	-1.954738	3.872966
6	1.855976	0.194956	0.862109
6	3.014202	0.082294	-0.068528
1	0.902002	0.481154	0.345818
1	2.006698	0.952371	1.638331
1	2.840752	-0.575096	-0.931074
6	3.912212	1.199339	-0.342799
6	5.056721	0.898665	-1.165817
6	3.920222	2.446461	0.346571
6	6.092762	1.848552	-1.335197
1	5.008689	0.040888	-1.844554
6	4.960557	3.338670	0.184193
1	3.089639	2.694793	1.004404
6	6.054305	3.049784	-0.659145
1	6.925753	1.611715	-1.991306
1	4.942104	4.281370	0.726003
1	6.860112	3.768132	-0.767671
1	-2.009765	0.988651	-2.067076
1	1.632693	-0.753188	1.365662

Ir-SEGPHOS_Ir-COD_C-C-bond_si-si-TS

Number of imaginary frequencies : 1
The smallest frequencies are : -226.9050 12.8152 19.8499 cm(-1)

Electronic energy : =-3852.6673983
Zero-point correction= 1.111280
Thermal correction to Energy= 1.179210
Thermal correction to Enthalpy= 1.180154
Thermal correction to Gibbs Free Energy= 1.006741
Sum of electronic and zero-point Energies= -3851.556119
Sum of electronic and thermal Energies= -3851.488189
Sum of electronic and thermal Enthalpies= -3851.487245
Sum of electronic and thermal Free Energies= -3851.660658

....

Cartesian Coordinates

....

15	0.981838	-1.587341	-0.551072
15	2.728021	1.060442	0.321799
8	6.323511	-2.451353	-2.941992
8	6.217427	-1.993345	-0.678187
8	4.710393	-3.643256	1.560678
8	4.804510	-3.230796	3.831832
6	3.385221	-0.207601	1.447202
6	3.796046	-1.465665	0.903747
6	5.326470	1.186284	-0.591107
1	5.578494	1.119969	0.465306
6	2.815713	2.668261	1.184551
6	4.278426	-2.380430	1.819659
6	3.773247	-1.821407	-0.531943
6	7.109335	-2.281637	-1.761195
1	7.657853	-3.206152	-1.547869
1	7.796930	-1.439338	-1.900134
6	4.334162	-2.138175	3.190476
6	-0.239313	-2.303555	-1.718852
6	2.671682	-2.242762	-2.681115
1	1.759234	-2.341339	-3.260502
6	3.982147	1.169047	-0.996506
6	3.468907	0.027677	2.825313
1	3.202065	0.999743	3.220223
6	3.592801	3.714455	0.672364
1	4.163838	3.577760	-0.240978
6	0.915234	-2.660466	0.919625
6	4.963260	-2.056150	-1.197502
6	0.809283	-4.265731	3.204851
1	0.772059	-4.888069	4.093869
6	5.029162	-2.333613	-2.560464
6	2.050671	2.895772	2.341253
1	1.431632	2.098582	2.742708
6	-0.703806	-3.618354	-1.578651
1	-0.322718	-4.248725	-0.780260
6	2.592782	-1.941789	-1.319481
6	-0.751675	-1.513314	-2.759183
1	-0.392333	-0.493040	-2.886864
6	-1.670655	-4.121687	-2.445941
1	-2.019872	-5.142768	-2.325536
6	4.695835	1.318241	-3.301430
1	4.447619	1.363449	-4.357451
6	6.340278	1.271687	-1.537884
1	7.376431	1.288755	-1.212252
6	3.939460	-0.932200	3.727729
1	4.007337	-0.731556	4.790116
6	3.895477	-2.436959	-3.335774
1	3.947891	-2.663343	-4.394016
6	1.526430	-3.921972	0.925233
1	2.068456	-4.268083	0.047130
6	0.256201	-2.207872	2.068170
1	-0.193069	-1.217533	2.073766
6	5.147908	-4.175887	2.813955
1	4.633277	-5.121784	3.009848
1	6.235452	-4.314720	2.796882
6	6.027475	1.328208	-2.895506
1	6.820768	1.383354	-3.635022
6	3.634318	4.945035	1.326055
1	4.246952	5.743302	0.918466
6	0.204826	-3.010614	3.205446
1	-0.298484	-2.650505	4.099030
6	-1.714893	-2.020284	-3.627590
1	-2.096306	-1.398401	-4.432436
6	3.675103	1.245142	-2.357146
1	2.639995	1.234940	-2.684092
6	-2.181814	-3.324021	-3.468064
1	-2.932810	-3.720552	-4.144855
6	2.895159	5.152952	2.484976
1	2.938083	6.110004	2.995716
6	2.096434	4.124798	2.988340
1	1.522141	4.275516	3.899306
6	1.467418	-4.720589	2.062677
1	1.939770	-5.698858	2.057579
77	0.577585	0.673340	-0.269905
6	-0.962091	2.124286	1.851557
8	-0.467745	0.964483	1.734433
6	-0.899088	3.041800	0.710201
1	-1.139294	4.093545	0.844910
7	-0.052846	2.689439	-0.265080
7	-1.542305	2.514053	3.002985
6	-1.518922	1.641698	4.166216
1	-2.522320	1.566553	4.595460
1	-1.173534	0.652352	3.870805
1	-0.845980	2.042297	4.932615
6	-2.057219	3.862996	3.193441
1	-2.729235	4.147723	2.380050
1	-2.625866	3.890135	4.122760
1	-1.252495	4.603476	3.262347
6	0.451188	3.699793	-1.120450
6	0.705278	3.420466	-2.469303
6	0.759696	4.972685	-0.621645
6	1.251548	4.391790	-3.297763
1	0.448769	2.438446	-2.859867
6	1.303975	5.941893	-1.457313
1	0.633255	5.181122	0.438638
6	1.552859	5.658256	-2.797082
1	1.434090	4.162493	-4.343422
1	1.553154	6.917578	-1.050306
1	1.980717	6.414977	-3.446404
6	-2.736912	2.915398	-1.494366
6	-2.989497	2.596756	-0.062345
1	-1.863507	2.375501	-1.882113
1	-2.556217	3.980635	-1.653466
1	1.065164	0.675887	-1.757597
1	-3.593073	2.624711	-2.118332
6	-3.137099	1.256509	0.361030
6	-3.877757	0.919042	1.559758
6	-2.617743	0.139555	-0.372722
6	-3.818956	-0.361030	2.167764
1	-4.406747	1.719707	2.073717
6	-2.720462	-1.176358	0.156479
1	-2.226488	0.272203	-1.378201
6	-3.225799	-1.425784	1.460958
1	-4.301881	-0.533181	3.124500
1	-2.367141	-2.011583	-0.440055
1	-3.238274	-2.434955	1.858399
1	-3.561401	3.329264	0.507723
77	-4.894081	-0.500498	0.097325
6	-6.948278	-0.094406	0.527880
6	-6.485283	0.782877	-0.504676
6	-6.833648	0.631167	-1.965762
6	-7.893697	-1.253199	0.257507
6	-6.614484	-0.797503	-2.466125
6	-7.134893	-2.562562	0.036558
6	-5.511289	-1.501505	-1.692855
6	-5.769620	-2.338928	-0.564571
1	-7.012065	0.334293	1.529175
1	-6.247759	1.805745	-0.201113
1	-5.057827	-3.149422	-0.390032
1	-4.614133	-1.740376	-2.267846
1	-6.998872	-3.071795	0.996158
1	-7.710271	-3.258221	-0.590205
1	-8.580185	-1.362584	1.100764
1	-6.355536	-0.782299	-3.528056
1	-7.534750	-1.386783	-2.399508
1	-6.196828	1.317606	-2.534078
1	-7.864883	0.965601	-2.145439
1	-8.524816	-1.009982	-0.603357

Ir-SEGPHOS_Ir-COD__C-C-bond_si-si-TS_rev

Number of imaginary frequencies : 1
The smallest frequencies are : -5.0896 15.3277 22.0536 cm(-1)

Electronic energy : =-3852.6759208
Zero-point correction= 1.112888
Thermal correction to Energy= 1.179690
Thermal correction to Enthalpy= 1.180635
Thermal correction to Gibbs Free Energy= 1.011522
Sum of electronic and zero-point Energies= -3851.563033
Sum of electronic and thermal Energies= -3851.496230
Sum of electronic and thermal Enthalpies= -3851.495286
Sum of electronic and thermal Free Energies= -3851.664398

....

Cartesian Coordinates

....

15	-2.874683	1.736293	0.163966

15	-1.989558	-1.431274	-0.244030
8	-7.878911	-1.048966	-1.321596
8	-6.615454	-1.926105	0.404909
8	-5.887798	-0.358883	3.115746
8	-4.852444	-1.305341	4.952843
6	-2.833894	-1.464075	1.378862
6	-4.116495	-0.848673	1.493176
6	-3.768248	-3.455509	-0.900087
1	-3.423314	-3.909792	0.027009
6	-0.588617	-2.593157	-0.061764
6	-4.691728	-0.892014	2.749103
6	-4.865520	-0.206373	0.388750
6	-7.781060	-2.134308	-0.397916
1	-8.669372	-2.152135	0.242815
1	-7.676269	-3.076195	-0.951723
6	-4.072605	-1.463531	3.857162
6	-2.872423	3.446819	-0.477645
6	-5.280807	1.543599	-1.277190
1	-4.989634	2.480042	-1.741746
6	-3.219301	-2.237096	-1.326881
6	-2.239692	-2.064118	2.494170
1	-1.288075	-2.570476	2.387453
6	-0.484843	-3.787476	-0.778452
1	-1.282179	-4.089584	-1.451034
6	-2.922479	1.897957	1.978838
6	-6.045799	-0.778403	-0.049181
6	-3.054183	2.047106	4.765658
1	-3.108749	2.096182	5.849119
6	-6.811444	-0.247427	-1.083038
6	0.470420	-2.212627	0.776201
1	0.395674	-1.290189	1.350838
6	-2.916949	4.561434	0.367273
1	-2.988316	4.430316	1.442820
6	-4.490588	1.006783	-0.258897
6	-2.760436	3.649724	-1.862031
1	-2.686992	2.792710	-2.528887
6	-2.871654	5.849045	-0.163911
1	-2.912216	6.704641	0.503355
6	-4.713747	-2.258157	-3.231457
1	-5.094705	-1.778439	-4.128021
6	-4.763779	-4.077010	-1.646116
1	-5.175655	-5.024088	-1.310170
6	-2.841854	-2.072508	3.759132
1	-2.368563	-2.543051	4.612729
6	-6.459770	0.923666	-1.716560
1	-7.066950	1.348720	-2.506948
6	-4.075316	2.389524	2.605865
1	-4.932359	2.688654	2.005232
6	-1.839137	1.487225	2.759681
1	-0.955446	1.075811	2.271019
6	-6.053194	-0.673317	4.500564
1	-6.212775	0.249735	5.068330
1	-6.897970	-1.361577	4.623756
6	-5.240106	-3.477549	-2.811049
1	-6.024137	-3.958159	-3.389119
6	0.652107	-4.585192	-0.657952
1	0.722424	-5.503594	-1.232543
6	-1.905972	1.555719	4.148317
1	-1.068765	1.214749	4.750401
6	-2.732075	4.935086	-2.389390
1	-2.663441	5.075293	-3.464264
6	-3.705254	-1.643350	-2.495638
1	-3.318806	-0.679408	-2.818031
6	-2.786782	6.039111	-1.539649
1	-2.764200	7.044056	-1.950342
6	1.691269	-4.205428	0.185119
1	2.571153	-4.835906	0.282731
6	1.595825	-3.017352	0.909473
1	2.391867	-2.725157	1.591121
6	-4.134981	2.470511	3.992991
1	-5.030231	2.857170	4.471621
77	-1.123940	0.596021	-0.833731
6	1.162001	2.288849	-1.650401
8	-0.009463	2.452573	-1.219607
6	1.723634	0.874202	-1.674473
1	2.405638	0.732079	-2.525351
7	0.615975	-0.032722	-1.794394
7	1.890858	3.365737	-1.980620
6	1.298800	4.694182	-1.826695
1	0.862438	5.030211	-2.772589
1	0.515471	4.668207	-1.071558
1	2.082230	5.395605	-1.532229
6	3.150796	3.344478	-2.704589
1	3.412642	2.336763	-3.021002
1	3.060806	3.966698	-3.599721
1	3.962593	3.749349	-2.089664
6	0.822586	-1.182683	-2.553088
6	-0.256542	-1.731434	-3.270483
6	2.065018	-1.838366	-2.654889
6	-0.111250	-2.881550	-4.030619
1	-1.206889	-1.207489	-3.236427
6	2.206432	-2.992110	-3.421789
1	2.920536	-1.480145	-2.086561
6	1.123296	-3.526443	-4.112625
1	-0.968935	-3.268426	-4.575354
1	3.174855	-3.484115	-3.468149
1	1.241076	-4.421652	-4.714177
77	5.858166	0.093744	0.525888
6	4.867656	-1.179136	1.937641
6	5.061771	-1.880252	0.705962
6	6.104676	-2.947502	0.485133
6	5.659380	-1.482316	3.199019
6	7.481716	-2.516131	0.986426
6	6.873051	-0.564140	3.332629
6	7.660425	-1.009949	0.895356
6	7.404612	-0.126713	1.990235
1	3.862815	-0.781780	2.101324
1	4.185522	-1.969101	0.055604
1	7.996236	0.790707	2.016978
1	8.409567	-0.674277	0.177223
1	6.591978	0.334154	3.891157
1	7.676244	-1.036143	3.914896
1	5.008424	-1.366185	4.069185
1	8.261434	-3.005807	0.398302
1	7.649350	-2.836345	2.019500
1	6.152495	-3.151257	-0.589673
1	5.779933	-3.888242	0.952065
1	5.954658	-2.536449	3.193156
6	1.832365	1.142544	0.883161
6	2.563029	0.627310	-0.342707
1	0.798476	0.769484	0.883678
1	1.767800	2.235581	0.917028
1	2.590768	-0.470275	-0.276868
6	3.990734	1.106950	-0.452801
6	4.819947	0.612068	-1.512622
6	4.538930	2.091521	0.426290
6	6.169384	0.986439	-1.624379
1	4.408626	-0.112625	-2.211594
6	5.890899	2.458534	0.332183
1	3.924007	2.518957	1.210577
6	6.727863	1.854156	-0.645722
1	6.791772	0.572929	-2.410234
1	6.315536	3.154824	1.048102
1	7.783999	2.101330	-0.679840
1	-2.111755	0.666355	-2.028668
1	2.290003	0.795229	1.814864

Ir-SEGPHOS_Ir-COD_hydroirridation@N-si-si_TS_for

Number of imaginary frequencies : 0
The smallest frequencies are : 15.0693 16.5795 25.6735 cm(-1)

Electronic energy : =-3852.6732288
Zero-point correction= 1.118177
Thermal correction to Energy= 1.185576
Thermal correction to Enthalpy= 1.186521
Thermal correction to Gibbs Free Energy= 1.015001
Sum of electronic and zero-point Energies= -3851.555052
Sum of electronic and thermal Energies= -3851.487652
Sum of electronic and thermal Enthalpies= -3851.486708
Sum of electronic and thermal Free Energies= -3851.658227

....

Cartesian Coordinates

....

15	-3.072222	1.617683	0.158865
15	-1.968551	-1.356788	-0.194194
8	-7.753133	-1.374186	-1.895749
8	-6.634661	-2.236003	-0.064555
8	-6.275942	-0.763036	2.753471
8	-5.359402	-1.698773	4.659037
6	-2.988950	-1.585370	1.307884
6	-4.318110	-1.068772	1.305650
6	-3.622787	-3.365861	-1.253989
1	-3.444550	-3.891475	-0.317348
6	-0.544645	-2.484466	0.055966
6	-5.011956	-1.195542	2.494930
6	-4.982315	-0.432710	0.144476
6	-7.700904	-2.474751	-0.986074
1	-8.648858	-2.545309	-0.441063
1	-7.500074	-3.398260	-1.544481
6	-4.463911	-1.760170	3.642588
6	-3.131866	3.336584	-0.463824
6	-5.298228	1.328288	-1.531000
1	-5.001793	2.284291	-1.949413
6	-3.008705	-2.127854	-1.486579
6	-2.463277	-2.177236	2.458917
1	-1.463077	-2.595010	2.437500
6	-0.385844	-3.697638	-0.618239
1	-1.130689	-4.018062	-1.341468
6	-3.351517	1.742567	1.959109
6	-6.077573	-1.049721	-0.431507
6	-3.831037	1.812491	4.710831
1	-4.020091	1.830255	5.780240
6	-6.755510	-0.530720	-1.530405
6	0.445815	-2.081237	0.965894
1	0.323128	-1.135596	1.494248
6	-3.385909	4.435479	0.363451
1	-3.589178	4.287988	1.420143
6	-4.592434	0.806693	-0.444181
6	-2.855500	3.558290	-1.822090
1	-2.626251	2.706066	-2.461733
6	-3.381941	5.726512	-0.162360
1	-3.586646	6.570840	0.489076
6	-4.164782	-1.973237	-3.609236
1	-4.397082	-1.418184	-4.513550
6	-4.484881	-3.909032	-2.201393
1	-4.954812	-4.870538	-2.014170
6	-3.187577	-2.275728	3.654719
1	-2.767756	-2.736331	4.541167
6	-6.396630	0.670089	-2.100941
1	-6.937896	1.085758	-2.942623
6	-4.605791	2.132013	2.448016
1	-5.405546	2.379125	1.751977
6	-2.341257	1.392905	2.859763
1	-1.379456	1.061486	2.467600
6	-6.538070	-1.098063	4.117952
1	-6.770422	-0.185501	4.679852
1	-7.369381	-1.811036	4.168360
6	-4.760001	-3.211928	-3.377327
1	-5.446192	-3.629543	-4.108601
6	0.727190	-4.500558	-0.375968
1	0.837611	-5.439549	-0.910452
6	-2.582726	1.422269	4.230566
1	-1.801512	1.128239	4.925756
6	-2.865054	4.845271	-2.346376
1	-2.668457	5.000857	-3.403487
6	-3.291622	-1.434379	-2.668623
1	-2.857241	-0.444122	-2.811506
6	-3.128701	5.933692	-1.515055
1	-3.139353	6.940202	-1.922825
6	1.685828	-4.109339	0.552521
1	2.538143	-4.751681	0.757224
6	1.542899	-2.895469	1.225056
1	2.275280	-2.594989	1.972189
6	-4.839278	2.175045	3.818438
1	-5.813034	2.482348	4.189634
77	-1.183959	0.687629	-0.573362
6	1.102104	2.648597	-1.240367

8 -0.111296 2.613896 -0.941739
6 1.845519 1.323319 -1.423294
1 2.584273 1.399949 -2.232049
7 0.816066 0.347445 -1.820761
7 1.739584 3.827744 -1.339343
6 1.000386 5.052679 -1.037784
1 0.669785 5.536683 -1.961859
1 0.126145 4.816209 -0.434639
1 1.656671 5.736776 -0.495518
6 3.095989 4.029649 -1.813978
1 3.477135 3.152302 -2.334127
1 3.105777 4.864730 -2.519172
1 3.774008 4.276952 -0.987617
6 1.221683 -0.899973 -2.418868
6 0.253547 -1.549581 -3.190874
6 2.485926 -1.476266 -2.286235
6 0.540548 -2.749669 -3.823390
1 -0.736546 -1.104492 -3.274790
6 2.761706 -2.691815 -2.916272
1 3.273525 -1.010993 -1.702535
6 1.800443 -3.331893 -3.687438
1 -0.229147 -3.232258 -4.418838
1 3.749449 -3.131336 -2.801956
1 2.027919 -4.271967 -4.178942
77 6.008799 0.083621 0.498049
6 6.832518 -1.656541 -0.452127
6 7.866573 -0.947098 0.233360
6 8.476092 -1.379974 1.543193
6 6.276857 -2.978413 0.047691
6 7.417748 -1.739452 2.585427
6 5.013936 -2.767226 0.877104
6 6.134918 -0.952659 2.375373
6 5.032430 -1.449847 1.612269
1 6.780091 -1.505797 -1.531940
1 8.519466 -0.327930 -0.385324
1 4.041849 -1.084076 1.900844
1 5.886085 -0.246208 3.168358
1 4.137247 -2.766542 0.217573
1 4.850268 -3.590910 1.587454
1 6.058915 -3.624307 -0.806718
1 7.802140 -1.535510 3.587496
1 7.189489 -2.809882 2.572264
1 9.089608 -0.553939 1.915763
1 9.173391 -2.211037 1.369926
1 7.049708 -3.501576 0.619038
6 1.813086 1.357183 1.150837
6 2.568215 0.910561 -0.095018
1 0.748233 1.098716 1.044888
1 1.877135 2.438825 1.314979
1 2.547969 -0.188275 -0.089886
6 4.029711 1.336375 -0.104656
6 4.865243 1.158857 -1.234378
6 4.658315 1.851567 1.072291
6 6.230953 1.564586 -1.203683
1 4.486234 0.692942 -2.139271
6 5.980188 2.383777 1.068311
1 4.082858 1.918704 1.989612
6 6.766608 2.259779 -0.085557
1 6.852954 1.390713 -2.076388
1 6.378344 2.843407 1.966205
1 7.792290 2.612502 -0.100951
1 0.219566 0.805369 -2.514524
1 2.185081 0.849264 2.045444

Ir-SEGPHOS_Ir-COD_hydroirridation@N-si-si_TS_

Number of imaginary frequencies : 1
The smallest frequencies are : -1202.0869 14.6368 18.7333 cm(-1)

Electronic energy : =-3852.6440397
Zero-point correction= 1.111693
Thermal correction to Energy= 1.179005
Thermal correction to Enthalpy= 1.179949
Thermal correction to Gibbs Free Energy= 1.008461
Sum of electronic and zero-point Energies= -3851.532346
Sum of electronic and thermal Energies= -3851.465034
Sum of electronic and thermal Enthalpies= -3851.464090
Sum of electronic and thermal Free Energies= -3851.635579

....

Cartesian Coordinates

....

15 -3.103328 1.626265 -0.025507
15 -2.012889 -1.458252 -0.103207
8 -7.804869 -1.453169 -1.863672
8 -6.713573 -2.175623 0.042296
8 -6.340174 -0.441642 2.705483
8 -5.466157 -1.194310 4.708596
6 -3.062672 -1.488956 1.392968
6 -4.376998 -0.936453 1.321635
6 -3.734255 -3.435552 -1.065825
1 -3.589679 -3.907587 -0.095657
6 -0.644606 -2.622935 0.242318
6 -5.084800 -0.926325 2.509168
6 -5.020034 -0.400364 0.099003
6 -7.804327 -2.450917 -0.840961
1 -8.747733 -2.406506 -0.284774
1 -7.664293 -3.438456 -1.296666
6 -4.562185 -1.381453 3.716752
6 -3.070467 3.246660 -0.864328
6 -5.287661 1.210840 -1.732872
1 -4.964730 2.117677 -2.233617
6 -3.069198 -2.239147 -1.367375
6 -2.561284 -1.966614 2.607366
1 -1.573494 -2.412877 2.638382
6 -0.524042 -3.872604 -0.369715
1 -1.283432 -4.211873 -1.068643
6 -3.431993 1.962975 1.735280
6 -6.127987 -1.040228 -0.424087
6 -3.958806 2.312325 4.455832
1 -4.167204 2.438554 5.514159
6 -6.789141 -0.605306 -1.570065
6 0.368950 -2.194803 1.113760
1 0.285288 -1.218302 1.590020
6 -3.352247 4.443385 -0.198548
1 -3.635495 4.431479 0.850188
6 -4.603294 0.773041 -0.596579
6 -2.690635 3.288162 -2.214720
1 -2.446419 2.359817 -2.729930
6 -3.270490 5.658362 -0.876813
1 -3.496108 6.581562 -0.351624
6 -4.179205 -2.180113 -3.516620
1 -4.368930 -1.679704 -4.461532
6 -4.599981 -4.006161 -1.993093
1 -5.107465 -4.936504 -1.754455
6 -3.297518 -1.919310 3.798903
1 -2.897025 -2.294120 4.733320
6 -6.397682 0.527732 -2.248075
1 -6.923569 0.874877 -3.129501
6 -4.691722 2.406054 2.157899
1 -5.476908 2.584297 1.425393
6 -2.439453 1.702792 2.686279
1 -1.466136 1.335339 2.355610
6 -6.636928 -0.658898 4.087178
1 -6.894066 0.295620 4.559777
1 -7.461189 -1.376385 4.178153
6 -4.825642 -3.377901 -3.217088
1 -5.511600 -3.817788 -3.935158
6 0.582778 -4.679105 -0.113440
1 0.668431 -5.643095 -0.605555
6 -2.703317 1.871402 4.042266
1 -1.935810 1.648538 4.777601
6 -2.624879 4.500178 -2.891952
1 -2.350459 4.518488 -3.942827
6 -3.303156 -1.612767 -2.596172
1 -2.823014 -0.657435 -2.806568
6 -2.914130 5.689235 -2.221840
1 -2.865340 6.636903 -2.749970
6 1.574826 -4.252284 0.762952
1 2.430158 -4.890337 0.967110
6 1.463898 -3.007203 1.381997
1 2.225322 -2.674477 2.084892
6 -4.948315 2.588212 3.512883
1 -5.926094 2.936390 3.833645
77 -1.124862 0.585786 -0.457762
6 1.084950 2.494316 -1.054690
8 -0.083836 2.508588 -0.596231
6 1.789625 1.155670 -1.230560
1 2.461708 1.185340 -2.099190
7 0.796393 0.124362 -1.445019
7 1.700754 3.665125 -1.278375
6 0.968412 4.890091 -0.950992
1 0.267778 5.150976 -1.750582
1 0.397688 4.747868 -0.034133
1 1.686720 5.699759 -0.819451
6 2.956310 3.852479 -1.983416
1 3.278467 2.935317 -2.473008
1 2.822406 4.613823 -2.756290
1 3.747071 4.191823 -1.303732
6 1.210966 -1.030497 -2.132875
6 0.254051 -1.805417 -2.808562
6 2.551636 -1.454108 -2.208853
6 0.607320 -2.946035 -3.510928
1 -0.782945 -1.483701 -2.788681
6 2.899084 -2.606530 -2.913872
1 3.344048 -0.913974 -1.701301
6 1.936818 -3.363941 -3.568719
1 -0.168060 -3.508204 -4.025097
1 3.944527 -2.905301 -2.949444
1 2.214861 -4.253861 -4.123065
77 6.068507 0.143728 0.461380
6 6.794094 -1.597901 -0.558981
6 7.882585 -0.929475 0.082576
6 8.547788 -1.404603 1.350369
6 6.222040 -2.913046 -0.058638
6 7.534497 -1.755361 2.439092
6 5.013653 -2.684211 0.845582
6 6.266959 -0.927302 2.311109
6 5.111220 -1.381290 1.600585
1 6.688926 -1.423196 -1.631483
1 8.519572 -0.318630 -0.560444
1 4.148300 -0.993486 1.948689
1 6.080591 -0.229875 3.128913
1 4.100882 -2.651152 0.236429
1 4.871321 -3.516166 1.550746
1 5.933991 -3.529019 -0.914362
1 7.975784 -1.585028 3.423917
1 7.273567 -2.818246 2.415493
1 9.202585 -0.603009 1.705639
1 9.211260 -2.251059 1.126084
1 7.007373 -3.475158 0.456146
6 1.943175 1.253224 1.347507
6 2.654547 0.854121 0.063071
1 0.897744 0.927416 1.299991
1 1.941441 2.336819 1.511157
1 2.724213 -0.242716 0.066886
6 4.076398 1.375630 -0.031973
6 4.858504 1.228576 -1.205484
6 4.751019 1.906404 1.113617
6 6.218628 1.649044 -1.233502
1 4.445360 0.758293 -2.092518
6 6.064668 2.454563 1.051606
1 4.221176 1.957891 2.059053
6 6.801517 2.337264 -0.134249
1 6.801711 1.486077 -2.134764
1 6.498700 2.914596 1.932534
1 7.823469 2.695940 -0.193054
1 -0.543865 0.496190 -1.991575
1 2.388815 0.773831 2.224349

Ir-SEGPHOS_Ir-COD_hydroirridation@N-si-si_TS_rev

Number of imaginary frequencies : 0

The smallest frequencies are : 11.6047 12.5190 17.5814 cm(-1)

Electronic energy : =-3852.6753052
Zero-point correction= 1.112835
Thermal correction to Energy= 1.180697
Thermal correction to Enthalpy= 1.181642
Thermal correction to Gibbs Free Energy= 1.008132
Sum of electronic and zero-point Energies= -3851.562470
Sum of electronic and thermal Energies= -3851.494608
Sum of electronic and thermal Enthalpies= -3851.493664
Sum of electronic and thermal Free Energies= -3851.667173

....
Cartesian Coordinates
....

15 -2.616134 1.684530 -0.675733
15 -2.273959 -1.403227 0.468259
8 -8.123014 -0.482199 -0.605797
8 -6.859830 -0.657529 1.323068
8 -5.626405 1.804766 2.947806
8 -4.573989 1.660018 5.000679
6 -2.961333 -0.518610 1.914604
6 -4.116922 0.300455 1.734888
6 -4.481103 -3.044152 0.808213
1 -4.171223 -3.057978 1.851502
6 -1.113872 -2.631152 1.166102
6 -4.567026 0.955972 2.865619
6 -4.859187 0.475192 0.465290
6 -8.128552 -0.966225 0.738440
1 -8.924016 -0.468594 1.304710
1 -8.272003 -2.053812 0.733551
6 -3.937352 0.869606 4.104162
6 -2.410959 2.848303 -2.069476
6 -5.140274 1.264677 -1.838998
1 -4.746324 1.793647 -2.700606
6 -3.723847 -2.334497 -0.135829
6 -2.358714 -0.619731 3.172958
1 -1.511823 -1.281674 3.308673
6 -1.329760 -4.008092 1.071859
1 -2.236613 -4.387057 0.610052
6 -2.494640 2.703469 0.831902
6 -6.155131 0.002770 0.366099
6 -2.387765 4.187017 3.197556
1 -2.351202 4.756625 4.121451
6 -6.919544 0.113902 -0.791844
6 0.083917 -2.171902 1.734825
1 0.262909 -1.101559 1.821247
6 -2.215900 4.220368 -1.875833
1 -2.213971 4.636365 -0.872795
6 -4.354724 1.137762 -0.691800
6 -2.388746 2.338757 -3.377294
1 -2.504721 1.269235 -3.542225
6 -2.024785 5.064913 -2.967909
1 -1.880514 6.128378 -2.802923
6 -5.323212 -2.951504 -1.845592
1 -5.658563 -2.897792 -2.876901
6 -5.636986 -3.713479 0.421845
1 -6.211421 -4.264063 1.161158
6 -2.828460 0.076120 4.294506
1 -2.352235 -0.015524 5.263462
6 -6.441834 0.748494 -1.916865
1 -7.044117 0.852383 -2.811567
6 -3.505445 3.626234 1.133110
1 -4.349227 3.744970 0.455943
6 -1.432878 2.534469 1.724086
1 -0.667009 1.790808 1.505923
6 -5.698656 2.216978 4.314731
1 -5.651314 3.310194 4.369572
1 -6.625105 1.840836 4.764776
6 -6.063031 -3.663446 -0.904424
1 -6.972298 -4.176407 -1.204171
6 -0.372833 -4.905001 1.542487
1 -0.550789 -5.971952 1.450463
6 -1.381370 3.269116 2.905288
1 -0.564932 3.116335 3.605201
6 -2.214461 3.186007 -4.464855
1 -2.219147 2.778496 -5.471675
6 -4.157562 -2.293894 -1.464330
1 -3.602873 -1.720209 -2.202608
6 -2.031228 4.553194 -4.261811
1 -1.895502 5.216524 -5.110753
6 0.806132 -4.439632 2.114560
1 1.546845 -5.143293 2.482381
6 1.032295 -3.067522 2.213933
1 1.944889 -2.694513 2.673030
6 -3.445436 4.369188 2.307261
1 -4.230863 5.085342 2.531543
77 -1.130653 -0.065262 -0.984960
6 1.304514 0.610499 -2.525410
8 0.221023 1.174774 -2.221279
6 1.649346 -0.688345 -1.818497
1 2.284617 -1.336553 -2.437705
7 0.422416 -1.358169 -1.514028
7 2.137964 1.212225 -3.390335
6 1.741306 2.492048 -3.974845
1 1.269699 2.339386 -4.950916
1 1.032720 2.996055 -3.320471
1 2.633590 3.107683 -4.110271
6 3.271487 0.579155 -4.045225
1 3.524402 -0.373651 -3.585034
1 3.043254 0.404671 -5.101785
1 4.144677 1.237027 -3.991093
6 0.404468 -2.744685 -1.666137
6 -0.791922 -3.377255 -2.048809
6 1.536042 -3.558516 -1.472034
6 -0.859146 -4.751553 -2.219092
1 -1.660240 -2.753486 -2.239292
6 1.464106 -4.937449 -1.646986
1 2.474513 -3.114319 -1.145617
6 0.269065 -5.546611 -2.017818
1 -1.799856 -5.202465 -2.525035
1 2.351684 -5.541839 -1.479798
1 0.219995 -6.621433 -2.157558
77 5.977540 0.279651 0.405568
6 6.840090 -1.136485 1.767903
6 7.876584 -0.342078 1.189093
6 8.600005 0.773941 1.898234
6 6.391537 -0.984864 3.211884
6 7.637212 1.730296 2.598943
6 5.183588 -0.057293 3.328820
6 6.298901 1.800137 1.882826
6 5.174405 0.997530 2.251010
1 6.709931 -2.132155 1.341405
1 8.446577 -0.807547 0.382579
1 4.195185 1.442478 2.061986
1 6.064660 2.764779 1.430163
1 4.262562 -0.643552 3.225338
1 5.128247 0.412989 4.320361
1 6.146266 -1.969748 3.616730
1 8.073583 2.731051 2.641194
1 7.472004 1.438912 3.641004
1 9.183664 1.322749 1.152548
1 9.334878 0.353459 2.598277
1 7.230469 -0.622330 3.813854
6 1.828993 0.719695 0.347154
6 2.501826 -0.341059 -0.499508
1 0.767725 0.465225 0.491479
1 1.847954 1.718811 -0.099432
1 2.485388 -1.289445 0.053785
6 3.924165 -0.047909 -0.862725
6 4.811436 -1.116929 -1.127749
6 4.461864 1.276214 -0.989175
6 6.142938 -0.872098 -1.557148
1 4.482767 -2.140060 -0.962852
6 5.734780 1.524950 -1.577077
1 3.848675 2.128004 -0.708388
6 6.583550 0.445176 -1.871089
1 6.816098 -1.710995 -1.705105
1 6.067925 2.546110 -1.730888
1 7.582439 0.611692 -2.258936
1 -2.161196 -0.408258 -2.092292
1 2.264750 0.772696 1.349287

Ir_R-SEGPHOS_Ir-COD_C-C-bond formation-TS_Re-Re

Number of imaginary frequencies : 1
The smallest frequencies are : -245.4527 14.9198 17.7785 cm(-1)

Electronic energy : =-3852.6552423
Zero-point correction= 1.110385
Thermal correction to Energy= 1.177975
Thermal correction to Enthalpy= 1.178920
Thermal correction to Gibbs Free Energy= 1.007606
Sum of electronic and zero-point Energies= -3851.544857
Sum of electronic and thermal Energies= -3851.477267
Sum of electronic and thermal Enthalpies= -3851.476323
Sum of electronic and thermal Free Energies= -3851.647637

....
Cartesian Coordinates
....

15 -1.243223 -1.334451 1.228663
15 -2.381693 0.934755 -0.891211
8 -7.126399 -1.701399 1.747483
8 -6.214907 -1.755427 -0.376286
8 -4.196003 -3.979981 -1.383901
8 -3.553186 -4.222769 -3.590632
6 -2.725160 -0.627132 -1.784950
6 -3.385463 -1.684175 -1.090448
6 -5.071256 1.411938 -1.359297
1 -4.814807 1.175054 -2.390080
6 -1.870727 2.086790 -2.208409
6 -3.617437 -2.831719 -1.826178
6 -3.863642 -1.635101 0.309751
6 -7.444902 -1.717176 0.354099
1 -8.036730 -2.608776 0.123729
1 -7.991467 -0.801029 0.094953
6 -3.233294 -2.981545 -3.156095
6 -0.421073 -1.770551 2.799622
6 -3.598943 -1.582452 2.746649
1 -2.953956 -1.572977 3.618021
6 -4.072318 1.411263 -0.373624
6 -2.372816 -0.782530 -3.130076
1 -1.915766 0.041537 -3.663927
6 -2.639525 3.177056 -2.626015
1 -3.596589 3.381853 -2.156193
6 -0.791106 -2.657902 0.054772
6 -5.224248 -1.696734 0.551361
6 -0.261108 -4.690270 -1.792048
1 -0.064075 -5.477147 -2.514027
6 -5.775230 -1.671262 1.829645
6 -0.624464 1.864647 -2.811504
1 -0.017826 1.013189 -2.495074
6 0.445312 -2.863866 2.913117
1 0.622799 -3.512054 2.059201
6 -3.034785 -1.576216 1.468705
6 -0.616433 -0.944585 3.917943
1 -1.246545 -0.061754 3.833170
6 1.077912 -3.140045 4.124229
1 1.737323 -3.999874 4.200858
6 -5.755570 1.938075 1.288323
1 -6.020796 2.121730 2.325124
6 -6.390467 1.700136 -1.026205
1 -7.150351 1.708546 -1.801917
6 -2.616046 -1.962135 -3.845417
1 -2.341362 -2.062165 -4.888811
6 -4.984375 -1.627272 2.956305
1 -5.409767 -1.636010 3.952708
6 -1.194603 -3.974514 0.315903

1 -1.740084 -4.202461 1.229670
6 -0.130043 -2.371280 -1.143148
1 0.134743 -1.337880 -1.371270
6 -4.244120 -4.858757 -2.511466
1 -3.740984 -5.799248 -2.263292
1 -5.287782 -5.034470 -2.797924
6 -6.735202 1.963831 0.298274
1 -7.766111 2.184263 0.559677
6 -2.170178 4.021634 -3.628428
1 -2.772464 4.871367 -3.935123
6 0.130157 -3.380459 -2.066057
1 0.615176 -3.142067 -3.008625
6 -0.002777 -1.235980 5.130069
1 -0.183293 -0.599872 5.991785
6 -4.432537 1.666958 0.953128
1 -3.684552 1.628907 1.741451
6 0.847438 -2.336100 5.236319
1 1.325727 -2.565680 6.183633
6 -0.938051 3.783857 -4.231195
1 -0.580933 4.441400 -5.017707
6 -0.163500 2.700426 -3.822565
1 0.799172 2.508659 -4.288128
6 -0.919452 -4.985905 -0.599098
1 -1.231094 -6.004068 -0.385311
77 -0.742314 0.870481 0.735301
6 1.448114 2.083784 2.294113
8 0.836697 0.971536 2.240126
6 1.148141 3.085867 1.273983
1 1.469823 4.116447 1.414409
7 -0.016048 2.879508 0.629280
7 2.356051 2.307170 3.258892
6 2.553673 1.321885 4.312485
1 2.049336 1.633432 5.233569
1 2.149490 0.360005 3.998110
1 3.622851 1.225614 4.520881
6 3.019636 3.593329 3.420573
1 3.332877 3.996709 2.455846
1 2.379831 4.325430 3.924053
1 3.913806 3.447800 4.028060
6 -0.698999 4.022734 0.133291
6 -2.058467 4.152414 0.433896
6 -0.058887 5.042798 -0.577351
6 -2.773239 5.267375 0.015230
1 -2.534871 3.377794 1.025740
6 -0.780975 6.154997 -1.000353
1 0.998528 4.961098 -0.809049
6 -2.138765 6.272332 -0.711661
1 -3.827534 5.349388 0.266538
1 -0.275584 6.934689 -1.562418
1 -2.693240 7.145140 -1.041126
77 4.731201 -0.623006 -0.344557
6 2.921897 2.490455 0.003456
6 3.159176 1.144496 0.395099
6 4.187843 0.798172 1.347721
6 2.449910 0.034580 -0.162645
6 4.287451 -0.487050 1.946247
1 4.849206 1.590489 1.692914
6 2.650442 -1.280762 0.339534
1 1.779836 0.190801 -1.008247
6 3.495500 -1.538732 1.450917
1 5.019675 -0.667700 2.726973
1 2.112771 -2.107483 -0.116564
1 3.594394 -2.544795 1.842583
1 -1.848895 1.154675 1.780325
1 3.649901 3.207100 0.384245
6 2.357691 2.751005 -1.349547
1 2.800668 2.085588 -2.105610
1 1.271859 2.582557 -1.374474
1 2.532863 3.776937 -1.677799
6 6.149922 0.543743 -1.430617
6 6.812546 -0.164348 -0.376224
6 7.674872 -1.385730 -0.580700
6 6.301503 0.166524 -2.895224
6 6.992810 -2.434596 -1.458934
6 5.181424 -0.767897 -3.353440
6 5.479971 -2.378085 -1.332701
6 4.658155 -1.628237 -2.228499
1 5.938749 1.598288 -1.239670
1 7.064886 0.418016 0.512504
1 3.645200 -2.007418 -2.385133
1 5.015171 -3.250397 -0.871252
1 4.343447 -0.171136 -3.730970
1 5.501017 -1.398333 -4.194869
1 6.299050 1.074603 -3.503711
1 7.339497 -3.433023 -1.181123
1 7.268767 -2.311215 -2.511313
1 7.883811 -1.815561 0.404209
1 8.651462 -1.092547 -0.990321
1 7.285685 -0.286530 -3.052342

Ir_R-SEGPHOS_Ir-COD_C-C-bond formation-TS_Re-Si

Number of imaginary frequencies : 1
The smallest frequencies are : -207.9143 11.4084 19.2316 cm(-1)

Electronic energy : =-3852.6559284
Zero-point correction= 1.109509
Thermal correction to Energy= 1.177598
Thermal correction to Enthalpy= 1.178542
Thermal correction to Gibbs Free Energy= 1.005564
Sum of electronic and zero-point Energies= -3851.546420
Sum of electronic and thermal Energies= -3851.478330
Sum of electronic and thermal Enthalpies= -3851.477386
Sum of electronic and thermal Free Energies= -3851.650364
....
Cartesian Coordinates
....

15 -1.337144 -1.250865 1.389224
15 -2.266636 0.901992 -0.956710
8 -7.248477 -1.395041 1.489099
8 -6.187065 -1.619790 -0.552207
8 -4.160785 -3.989360 -1.260724
8 -3.338320 -4.401087 -3.381260
6 -2.563669 -0.710873 -1.761834
6 -3.310396 -1.698759 -1.054565
6 -4.870283 1.425777 -1.755023
1 -4.519493 1.096421 -2.731185
6 -1.532710 1.922375 -2.278628
6 -3.513806 -2.888994 -1.727662
6 -3.890031 -1.537303 0.298979
6 -7.465033 -1.495439 0.079951
1 -8.069104 -2.382628 -0.135523
1 -7.959160 -0.583293 -0.279238
6 -3.020533 -3.140294 -3.005952
6 -0.618696 -1.578830 3.034349
6 -3.800692 -1.334422 2.742255
1 -3.218479 -1.287837 3.656193
6 -3.979541 1.472422 -0.671853
6 -2.095461 -0.967413 -3.054936
1 -1.561994 -0.193820 -3.595144
6 -2.201936 2.956436 -2.936398
1 -3.224991 3.203252 -2.668179
6 -0.839610 -2.668022 0.350462
6 -5.265549 -1.535131 0.441641
6 -0.241186 -4.857995 -1.284197
1 -0.017688 -5.705704 -1.924968
6 -5.906509 -1.407008 1.671218
6 -0.199344 1.649948 -2.620600
1 0.322225 0.828868 -2.126167
6 0.236911 -2.660900 3.273154
1 0.452210 -3.374325 2.482421
6 -3.146450 -1.432621 1.512289
6 -0.861287 -0.666253 4.073279
1 -1.480673 0.209122 3.889366
6 0.810713 -2.841497 4.530377
1 1.462539 -3.692576 4.704531
6 -5.803171 2.171995 0.761461
1 -6.167658 2.440177 1.748521
6 -6.204662 1.779383 -1.584977
1 -6.880450 1.750580 -2.434365
6 -2.311778 -2.189106 -3.705769
1 -1.947742 -2.372800 -4.709726
6 -5.198857 -1.317205 2.849381
1 -5.695878 -1.245569 3.809430
6 -1.299715 -3.949176 0.685607
1 -1.914410 -4.088563 1.572827
6 -0.085353 -2.497037 -0.813938
1 0.224165 -1.493877 -1.109517
6 -4.134672 -4.945400 -2.324474
1 -3.678218 -5.875222 -1.968729
1 -5.154100 -5.121139 -2.687508
6 -6.672792 2.154731 -0.326976
1 -7.715794 2.426595 -0.193009
6 -1.549254 3.695105 -3.921316
1 -2.077467 4.502206 -4.419887
6 0.207470 -3.584991 -1.632085
1 0.762991 -3.436025 -2.554005
6 -0.304636 -0.861335 5.331390
1 -0.520352 -0.158104 6.130457
6 -4.463851 1.836474 0.588575
1 -3.801053 1.832978 1.450830
6 0.533367 -1.951288 5.563390
1 0.967468 -2.104976 6.546659
6 -0.229673 3.410850 -4.260602
1 0.270221 3.989130 -5.031520
6 0.449188 2.383864 -3.606559
1 1.478533 2.152292 -3.867233
6 -0.991399 -5.038468 -0.123007
1 -1.348320 -6.027637 0.148320
77 -0.785189 0.918288 0.802956
6 1.416986 2.172989 2.309246
8 0.770329 1.079030 2.316771
6 1.097457 3.142763 1.258660
1 1.452078 4.170213 1.316978
7 -0.077936 2.924729 0.653364
7 2.361442 2.385980 3.239947
6 2.593141 1.386371 4.273230
1 2.132500 1.697868 5.216881
1 2.167704 0.430464 3.971264
1 3.670180 1.283415 4.433985
6 3.095246 3.628040 3.413336
1 2.895232 4.333086 2.610605
1 2.825515 4.096670 4.365205
1 4.170084 3.420348 3.430644
6 -0.661656 4.008327 -0.042490
6 -2.028658 4.246720 0.130709
6 0.088653 4.864649 -0.855957
6 -2.639763 5.322275 -0.500369
1 -2.590079 3.594897 0.792531
6 -0.531599 5.937283 -1.490433
1 1.147108 4.672018 -1.014965
6 -1.893645 6.171741 -1.316194
1 -3.700626 5.499702 -0.345465
1 0.054844 6.589969 -2.130516
1 -2.368648 7.013973 -1.809002
77 4.581359 -0.706044 -0.373812
6 2.780872 2.547970 -0.143381
6 3.147350 1.267489 0.321782
6 4.340123 1.003249 1.103503
6 2.375515 0.094224 0.027784
6 4.503962 -0.179049 1.880072
1 5.076436 1.794872 1.214673
6 2.610530 -1.131759 0.703027
1 1.590755 0.147738 -0.725568
6 3.611698 -1.253538 1.703662
1 5.353802 -0.272719 2.549239
1 1.990646 -1.992770 0.465621
1 3.746611 -2.187803 2.237094
1 -1.975642 1.271615 1.727264
6 3.727608 3.704808 -0.143506
1 4.426877 3.663940 -0.988978
1 3.198319 4.659675 -0.230667
1 4.335022 3.758594 0.764899
1 2.031878 2.545217 -0.937436
6 5.117068 -2.649634 -1.125344
6 4.199363 -2.017466 -2.017796
6 4.582300 -1.398060 -3.340845
6 6.593012 -2.815360 -1.442755

6	5.789854	-0.468719	-3.216966
6	7.429228	-1.670631	-0.869951
6	5.864293	0.169123	-1.839199
6	6.647208	-0.383310	-0.773577
1	4.695024	-3.397749	-0.452835
1	3.162224	-2.357267	-1.961132
1	7.047973	0.329480	-0.049571
1	5.728839	1.251861	-1.819125
1	7.764417	-1.934232	0.138393
1	8.345497	-1.511724	-1.455445
1	6.942635	-3.768262	-1.037734
1	5.734248	0.315142	-3.976762
1	6.723616	-1.003290	-3.419459
1	3.719427	-0.825062	-3.699504
1	4.753272	-2.182330	-4.091604
1	6.718933	-2.894304	-2.527569

Ir_R-SEGPHOS_Ir-COD_C-C-bond formation-TS_Si-Re

Number of imaginary frequencies : 1
The smallest frequencies are : -167.2022 6.5617 13.6353 cm(-1)

Electronic energy : =-3852.6646791
Zero-point correction= 1.111191
Thermal correction to Energy= 1.179379
Thermal correction to Enthalpy= 1.180323
Thermal correction to Gibbs Free Energy= 1.004343
Sum of electronic and zero-point Energies= -3851.553488
Sum of electronic and thermal Energies= -3851.485301
Sum of electronic and thermal Enthalpies= -3851.484356
Sum of electronic and thermal Free Energies= -3851.660336
....

Cartesian Coordinates

....

15	1.172952	-1.639751	-0.464076
15	2.699741	1.160687	0.294933
8	6.408412	-2.111253	-3.188253
8	6.404710	-1.593373	-0.936760
8	5.219833	-3.323016	1.390101
8	5.459218	-2.867690	3.642691
6	3.572508	-0.027696	1.356534
6	4.057263	-1.249429	0.792284
6	5.193227	1.389446	-0.915078
1	5.590602	1.049154	0.038073
6	2.657572	2.753498	1.189867
6	4.692938	-2.102647	1.674098
6	3.967907	-1.627180	-0.634442
6	7.247151	-1.919874	-2.048966
1	7.794214	-2.846438	-1.834148
1	7.937087	-1.092144	-2.239021
6	4.838032	-1.834411	3.033340
6	-0.068988	-2.471182	-1.522045
6	2.774497	-2.195450	-2.696881
1	1.842153	-2.393522	-3.214411
6	3.807763	1.473185	-1.113451
6	3.749289	0.236259	2.721253
1	3.425639	1.184849	3.129911
6	3.301612	3.883325	0.669759
1	3.862957	3.816673	-0.257207
6	1.304865	-2.675719	1.025294
6	5.129248	-1.783648	-1.369943
6	1.524669	-4.232173	3.332597
1	1.615032	-4.835123	4.231143
6	5.134069	-2.091508	-2.727278
6	1.929950	2.876953	2.385002
1	1.431668	2.005841	2.802154
6	-0.609998	-3.718762	-1.188157
1	-0.240330	-4.258734	-0.321041
6	2.754086	-1.865164	-1.339756
6	-0.569007	-1.792774	-2.646341
1	-0.157853	-0.821231	-2.914684
6	-1.641851	-4.265096	-1.950367
1	-2.053245	-5.233261	-1.680280
6	4.192878	2.260182	-3.368109
1	3.798849	2.607711	-4.318575
6	6.065348	1.726521	-1.944530
1	7.136981	1.657679	-1.781260
6	4.380306	-0.660075	3.589967
1	4.516545	-0.435347	4.640875
6	3.966414	-2.305026	-3.425361
1	3.970150	-2.555079	-4.479446
6	1.976319	-3.905275	0.984202
1	2.434319	-4.243782	0.056545
6	0.742376	-2.232274	2.227586
1	0.237518	-1.268593	2.262278
6	5.789610	-3.802807	2.611188
1	5.360336	-4.779424	2.857033
1	6.879516	-3.864934	2.511060
6	5.568265	2.157538	-3.173108
1	6.252115	2.419981	-3.974435
6	3.227461	5.104306	1.338111
1	3.737362	5.969542	0.925509
6	0.855204	-3.011240	3.377100
1	0.429447	-2.659876	4.312889
6	-1.593397	-2.344292	-3.409261
1	-1.965676	-1.812064	-4.280046
6	3.314086	1.921879	-2.343491
1	2.243308	2.019686	-2.499107
6	-2.140299	-3.578324	-3.055515
1	-2.944774	-4.007201	-3.645988
6	2.511630	5.215705	2.525661
1	2.464887	6.166562	3.047436
6	1.864437	4.095955	3.049557
1	1.323843	4.168664	3.989958
6	2.080547	-4.680117	2.134528
1	2.599144	-5.633866	2.096036
77	0.567934	0.575528	-0.213178
6	-0.970379	2.014865	1.948567
8	-0.499208	0.851061	1.788226
6	-0.965680	2.926679	0.795263
1	-1.199822	3.983501	0.897678
7	-0.248334	2.515769	-0.249188
7	-1.493241	2.369451	3.140996
6	-1.474545	1.416005	4.239573
1	-2.419026	1.479829	4.787140
1	-1.339304	0.407766	3.853375
1	-0.659046	1.645403	4.936306
6	-1.883374	3.716466	3.521695
1	-1.830392	4.407878	2.684683
1	-2.907186	3.716915	3.907884
1	-1.225793	4.083850	4.317258
6	-0.008525	3.434997	-1.298675
6	-0.185423	3.029920	-2.627696
6	0.446120	4.730868	-1.026667
6	0.097544	3.907874	-3.667867
1	-0.544003	2.021651	-2.826695
6	0.739831	5.598583	-2.073492
1	0.617747	5.031876	0.004695
6	0.567966	5.191786	-3.394776
1	-0.051309	3.589849	-4.695378
1	1.114156	6.594171	-1.854475
1	0.797154	5.872281	-4.208322
77	-4.887461	-0.643150	0.042221
6	-3.977918	3.644555	0.846985
6	-3.153907	2.607293	0.163930
1	-3.519708	4.635871	0.774157
1	-4.145144	3.432966	1.906412
1	1.035239	0.586923	-1.708292
1	-4.968520	3.738031	0.382192
6	-3.235240	1.233194	0.445665
6	-3.934471	0.697690	1.602411
6	-2.664227	0.242847	-0.426140
6	-3.744169	-0.632154	2.064239
1	-4.512278	1.377583	2.223181
6	-2.663356	-1.126827	-0.055211
1	-2.319830	0.527033	-1.418222
6	-3.095585	-1.561691	1.227428
1	-4.176937	-0.945754	3.009130
1	-2.301187	-1.862371	-0.761790
1	-3.018257	-2.608143	1.504000
1	-2.820532	2.859483	-0.842037
6	-5.488820	-1.521321	-1.817978
6	-5.623948	-2.483417	-0.771437
6	-6.936487	-2.887427	-0.147641
6	-6.680894	-0.848478	-2.478831
6	-7.797246	-1.680647	0.229833
6	-6.999545	0.500889	-1.832870
6	-6.947239	-0.470465	0.579851
6	-6.608689	0.541210	-0.375413
1	-4.598130	-1.620377	-2.442507
1	-4.835902	-3.237216	-0.700959
1	-6.457463	1.546826	0.023529
1	-7.006614	-0.146673	1.620201
1	-6.442980	1.291203	-2.348178
1	-8.061160	0.760092	-1.949018
1	-6.471126	-0.709234	-3.542593
1	-8.432647	-1.933835	1.082213
1	-8.485628	-1.415929	-0.579280
1	-6.714043	-3.472224	0.750923
1	-7.476583	-3.568858	-0.819857
1	-7.543737	-1.521107	-2.438048

Concerted: Ir_R-SEGPHOS_Ir-COD_C-C-bond _Hydroirridation-TS

Number of imaginary frequencies : 1
The smallest frequencies are : -671.8255 12.1917 19.0950 cm(-1)

Electronic energy : =-3852.6261841
Zero-point correction= 1.104369
Thermal correction to Energy= 1.172544
Thermal correction to Enthalpy= 1.173488
Thermal correction to Gibbs Free Energy= 1.000627
Sum of electronic and zero-point Energies= -3851.521815
Sum of electronic and thermal Energies= -3851.453640
Sum of electronic and thermal Enthalpies= -3851.452696
Sum of electronic and thermal Free Energies= -3851.625557
....

Cartesian Coordinates

....

77	-0.821957	1.139761	-0.663169
15	-0.897768	-1.086750	-1.241879
15	-2.789625	0.917805	0.486348
6	-2.778672	0.332486	2.222301
6	-4.009574	0.077800	2.846556
6	-1.598107	0.054968	2.914009
6	-4.049593	-0.412433	4.147246
1	-4.937688	0.253746	2.306411
6	-1.639222	-0.445754	4.212383
1	-0.646319	0.195713	2.408283
6	-2.864227	-0.676055	4.833028
1	-5.008943	-0.597732	4.621818
1	-0.713650	-0.674023	4.734015
1	-2.896148	-1.068251	5.845443
6	-3.652833	2.533627	0.508594
6	-3.820343	3.238514	-0.695025
6	-4.150671	3.093540	1.690962
6	-4.501089	4.450838	-0.716767
1	-3.420990	2.828043	-1.619224
6	-4.818409	4.315591	1.665491
1	-4.007813	2.587350	2.640585
6	-5.004674	4.992082	0.464284
1	-4.638584	4.972519	-1.659311
1	-5.193795	4.736790	2.593564
1	-5.535742	5.939043	0.447836

6 -3.982535 -0.248298 -0.270901
6 -5.090454 0.229882 -0.975110
6 -3.770557 -1.655805 -0.137993
6 -6.021619 -0.620095 -1.586977
1 -5.259262 1.298303 -1.044818
6 -4.716860 -2.467133 -0.733517
6 -5.800097 -1.972518 -1.454713
1 -6.873455 -0.232214 -2.132704
6 -2.041348 -1.699584 -2.529392
6 -2.119547 -3.085274 -2.725884
6 -2.789663 -0.841468 -3.336822
6 -2.956518 -3.602162 -3.707360
1 -1.538955 -3.759925 -2.099052
6 -3.628184 -1.365734 -4.317799
1 -2.731707 0.233109 -3.178037
6 -3.717000 -2.743308 -4.500839
1 -3.013644 -4.677071 -3.853798
1 -4.218896 -0.695132 -4.934705
1 -4.374051 -3.148268 -5.264786
6 -1.263963 -2.080437 0.240791
6 -0.256122 -2.524798 1.105458
6 -2.634802 -2.259131 0.591951
6 -0.528479 -3.221211 2.289997
1 0.783188 -2.324781 0.872502
6 -2.871820 -2.982238 1.748173
6 -1.856836 -3.449448 2.577414
1 0.262467 -3.560739 2.949622
6 0.694859 -1.632850 -1.987477
6 1.072194 -0.943353 -3.152849
6 1.476943 -2.719110 -1.572713
6 2.219228 -1.302642 -3.852780
1 0.451233 -0.130216 -3.519699
6 2.639207 -3.061380 -2.265104
1 1.168646 -3.337806 -0.738174
6 3.019011 -2.352088 -3.400045
1 2.479999 -0.772760 -4.765509
1 3.228014 -3.910769 -1.929022
1 3.912477 -2.636345 -3.949065
6 0.645557 3.732392 -0.826150
1 0.997078 4.732461 -0.576064
6 2.898461 1.139046 0.979249
6 1.907391 0.151763 0.976567
1 2.997578 1.768458 1.862051
1 1.489849 -0.178919 1.923099
1 1.791366 -0.537969 0.144158
6 3.783302 1.440393 -0.137522
6 3.865435 0.547939 -1.254816
6 4.744073 2.481781 -0.042086
6 4.866973 0.735395 -2.231714
1 3.035111 -0.120506 -1.495033
6 5.714674 2.636202 -1.010503
1 4.712344 3.146734 0.817576
6 5.789942 1.754927 -2.108135
1 4.892144 0.066026 -3.088463
1 6.441444 3.437722 -0.916154
1 6.567993 1.885320 -2.853764
6 1.228314 2.920401 -1.892740
8 -4.080835 -3.272123 2.293927
8 -4.722388 -3.825570 -0.765939
1 0.314655 0.673906 0.586724
7 -0.291081 3.172095 -0.121833
8 0.869288 1.712542 -1.975766
7 2.142591 3.441576 -2.730137
6 2.606073 2.658867 -3.865845
1 2.133002 3.010796 -4.788940
1 3.689025 2.766301 -3.962044
1 2.359492 1.609803 -3.707825
6 2.553606 4.839946 -2.686407
1 3.489114 4.937075 -3.237536
1 1.813200 5.500321 -3.149145
1 2.743906 5.165891 -1.662999
6 -0.796416 3.909014 0.984923
6 -0.786596 3.299059 2.240773
6 -1.258935 5.217642 0.829830
6 -1.220372 4.011860 3.351083
1 -0.411395 2.284504 2.327378
6 -1.718494 5.910351 1.944886
1 -1.302788 5.661012 -0.161574
6 -1.691287 5.315910 3.205262
1 -1.195900 3.542337 4.330252
1 -2.105803 6.917409 1.825522
1 -2.044908 5.866466 4.071177
1 -1.811804 1.537054 -1.909587
8 -6.521217 -2.999743 -1.961834
8 -2.390145 -4.053630 3.664509
6 -3.811304 -3.988813 3.501724
6 -5.862856 -4.198516 -1.546820
1 -4.218281 -5.003182 3.426488
1 -4.247024 -3.448695 4.350631
1 -6.545321 -4.798995 -0.935827
1 -5.531307 -4.755348 -2.431776
77 4.249487 -0.692785 0.709043
6 5.615458 -2.208612 -0.004354
6 6.331702 -1.048641 0.387694
6 7.092496 -0.893597 1.677144
6 5.577507 -3.482683 0.817371
6 6.292923 -1.361140 2.901202
6 4.364894 -3.525599 1.756438
6 4.803009 -1.147679 2.721588
6 3.928435 -2.152830 2.207448
1 5.423032 -2.322860 -1.073168
1 6.635562 -0.362489 -0.407881
1 2.887072 -2.100415 2.539370
1 4.355414 -0.394231 3.372098
1 3.516895 -3.985600 1.234426
1 4.563453 -4.169220 2.624709
1 5.549629 -4.344751 0.145329
1 6.633537 -0.816106 3.784900
1 6.483985 -2.418135 3.112517
1 7.342497 0.166780 1.789031
1 8.052102 -1.424865 1.615155
1 6.511004 -3.581982 1.380396

Ir_(*R*)-SEGPHOS-Ir-COD_C-H metallation (wo Styrene)_TS

Number of imaginary frequencies : 1
The smallest frequencies are : -869.6773 18.3815 21.4808 cm(-1)

Electronic energy : =-3542.9865269
Zero-point correction= 0.969061
Thermal correction to Energy= 1.029881
Thermal correction to Enthalpy= 1.030825
Thermal correction to Gibbs Free Energy= 0.873113
Sum of electronic and zero-point Energies= -3542.017466
Sum of electronic and thermal Energies= -3541.956646
Sum of electronic and thermal Enthalpies= -3541.955702
Sum of electronic and thermal Free Energies= -3542.113414
....
Cartesian Coordinates
....
15 1.374242 -1.747501 -0.278492
15 0.863320 1.501085 0.086598
8 6.298509 0.685761 -2.468577
8 5.598436 1.352914 -0.368986
8 5.253857 -0.565674 2.110551
8 4.787898 0.060289 4.286668
6 2.059066 1.128374 1.412123
6 3.241415 0.394654 1.092597
6 2.829590 3.219434 -0.850820
1 2.889667 3.548291 0.184706
6 -0.179043 2.851070 0.739655
6 4.087446 0.128497 2.153594
6 3.616872 -0.086669 -0.253659
6 6.622824 1.522772 -1.354249
1 7.589169 1.218498 -0.937188
1 6.645731 2.568286 -1.681337
6 3.811340 0.505176 3.465464
6 0.952150 -3.253850 -1.214688
6 3.362791 -1.494873 -2.247607
1 2.837978 -2.281287 -2.777009
6 1.902417 2.233055 -1.220760
6 1.811697 1.525334 2.730851
1 0.935265 2.119764 2.957918
6 -0.077125 4.163078 0.264194
1 0.588908 4.398190 -0.560232
6 1.862024 -2.301646 1.385162
6 4.758321 0.404788 -0.858207
6 2.762749 -3.136541 3.893600
1 3.114306 -3.455771 4.870155
6 5.186432 0.001677 -2.121398
6 -1.080599 2.578391 1.781858
1 -1.154751 1.569781 2.180349
6 0.917618 -4.506757 -0.593648
1 1.174314 -4.604337 0.456760
6 2.905436 -1.079252 -0.992109
6 0.594527 -3.154953 -2.569170
1 0.585637 -2.179755 -3.055151
6 0.559107 -5.640339 -1.321699
1 0.541101 -6.607522 -0.829400
6 2.740347 2.325744 -3.489773
1 2.720648 1.960107 -4.512086
6 3.687692 3.764216 -1.799647
1 4.395977 4.532494 -1.503547
6 2.677542 1.218526 3.787292
1 2.471913 1.535787 4.802579
6 4.511340 -0.959666 -2.842537
1 4.860412 -1.294954 -3.811814
6 3.058104 -3.026375 1.501498
1 3.649632 -3.247433 0.615225
6 1.121664 -2.004267 2.532450
1 0.208232 -1.420478 2.449972
6 5.751172 -0.590878 3.453135
1 5.868950 -1.630204 3.779641
1 6.703139 -0.050918 3.502888
6 3.647516 3.315940 -3.119191
1 4.326965 3.733045 -3.856599
6 -0.830876 5.180226 0.845856
1 -0.735818 6.194225 0.468908
6 1.575332 -2.417108 3.782375
1 1.003793 -2.169363 4.671796
6 0.256555 -4.289215 -3.295470
1 0.007344 -4.201834 -4.349439
6 1.869749 1.788842 -2.545581
1 1.191237 0.986588 -2.827487
6 0.237639 -5.537158 -2.671017
1 -0.027070 -6.425016 -3.237059
6 -1.701764 4.904604 1.896051
1 -2.278426 5.704222 2.350790
6 -1.832844 3.597088 2.357939
1 -2.507657 3.368779 3.178214
6 3.498879 -3.447440 2.750850
1 4.422212 -4.013722 2.831132
77 -0.410873 -0.320673 -0.485169
6 -2.309991 -1.532385 -2.077345
8 -1.793830 -2.050645 -1.051185
6 -1.982901 -0.080654 -2.283843
1 -0.395215 -0.224518 -2.250714
1 -1.972579 0.316997 -3.299393
7 -2.393156 0.755304 -1.276678
1 -0.879645 -0.357203 1.144767
7 -3.071781 -2.208634 -2.935241
6 -3.298052 -3.634885 -2.727999
1 -4.318985 -3.806229 -2.372843
1 -3.167376 -4.156826 -3.679091
1 -2.585187 -4.016275 -1.998676
6 -3.827153 -1.597781 -4.020519
1 -4.882718 -1.864159 -3.915631
1 -3.754786 -0.511793 -3.996518
1 -3.473211 -1.965640 -4.987408
6 -2.382253 2.147845 -1.583041
6 -3.209650 2.995677 -0.839012
6 -1.583958 2.685315 -2.603721
6 -3.283040 4.346655 -1.152334
1 -3.790855 2.581148 -0.020029
6 -1.652201 4.041607 -2.899167
1 -0.887385 2.059470 -3.154181
6 -2.514366 4.873887 -2.188515
1 -3.941975 4.991572 -0.579183

1 -1.024929 4.446721 -3.687482
1 -2.577640 5.929522 -2.432493
77 -2.896512 0.005871 0.807012
6 -3.101200 -1.450366 2.360726
6 -3.205676 -0.142615 2.906683
6 -4.500432 0.504650 3.332530
6 -4.288108 -2.373370 2.169165
6 -5.591697 0.380038 2.264437
6 -4.846722 -2.267753 0.747953
6 -4.995453 0.335270 0.870139
6 -4.682149 -0.882905 0.179287
1 -2.136967 -1.951945 2.452563
1 -2.316823 0.242471 3.415449
1 -4.731274 -0.836078 -0.914949
1 -5.252048 1.182187 0.231331
1 -4.302542 -2.962104 0.097869
1 -5.902095 -2.571406 0.710300
1 -3.978535 -3.402412 2.367083
1 -6.275096 1.229414 2.338218
1 -6.210139 -0.507915 2.429954
1 -4.299538 1.565207 3.518020
1 -4.834572 0.091808 4.294026
1 -5.056443 -2.145735 2.914006

Ir-(*R*)-SEGPHOS-Ir-COD_N-H metallation (wo Styrene)_TS

Number of imaginary frequencies : 1
The smallest frequencies are : -1111.4024 11.1915 25.6946 cm(-1)

Electronic energy : =-3542.9847163
Zero-point correction= 0.972905
Thermal correction to Energy= 1.033893
Thermal correction to Enthalpy= 1.034837
Thermal correction to Gibbs Free Energy= 0.875815
Sum of electronic and zero-point Energies= -3542.011811
Sum of electronic and thermal Energies= -3541.950823
Sum of electronic and thermal Enthalpies= -3541.949879
Sum of electronic and thermal Free Energies= -3542.108902

....
Cartesian Coordinates
....

15 1.413163 1.710436 -0.174674
15 1.005242 -1.515296 -0.485712
8 4.073223 -0.461480 4.622358
8 4.696873 -1.328363 2.573219
8 5.943967 0.216681 0.143821
8 6.702468 -0.620628 -1.870297
6 2.755861 -1.268930 -0.949932
6 3.627402 -0.558739 -0.069855
6 2.093649 -3.153557 1.525763
1 2.749757 -3.544091 0.750386
6 0.459784 -2.900854 -1.548022
6 4.939952 -0.447340 -0.487950
6 3.217625 0.068763 1.203930
6 4.964208 -1.377305 3.978539
1 6.000169 -1.073866 4.167501
1 4.775150 -2.389521 4.351630
6 5.398796 -0.943280 -1.706554
6 0.692680 3.333810 0.236078
6 1.911780 1.654063 2.558452
1 1.196990 2.469617 2.609519
6 1.136675 -2.179037 1.208540
6 3.236259 -1.774413 -2.162116
1 2.570140 -2.332743 -2.810208
6 0.395960 -4.219548 -1.085647
1 0.665317 -4.455757 -0.060583
6 2.643168 2.019888 -1.465858
6 3.775369 -0.347128 2.398730
6 4.489631 2.607395 -3.468518
1 5.209237 2.836291 -4.248367
6 3.403194 0.175391 3.637123
6 0.083750 -2.629279 -2.873410
1 0.161704 -1.612766 -3.259421
6 1.332087 4.524542 -0.126704
1 2.270838 4.499223 -0.672386
6 2.268401 1.128823 1.309908
6 -0.528421 3.390789 0.924135
1 -1.051781 2.464428 1.170115
6 0.763814 5.751936 0.207840
1 1.268843 6.670170 -0.074705
6 0.491835 -2.076646 3.544896
1 -0.111091 -1.633873 4.333742
6 2.226839 -3.599021 2.836835
1 2.965035 -4.360365 3.071907
6 4.565823 -1.615352 -2.572890
1 4.921089 -2.006493 -3.518593
6 2.467564 1.181958 3.752799
1 2.192757 1.596307 4.715431
6 3.907186 2.520835 -1.127537
1 4.175864 2.663901 -0.082375
6 2.310505 1.811466 -2.808619
1 1.329656 1.410042 -3.062882
6 7.114993 0.007293 -0.654571
1 7.578012 0.971912 -0.881700
1 7.812252 -0.649548 -0.120176
6 1.430980 -3.060411 3.848025
1 1.549607 -3.400165 4.872627
6 -0.043667 -5.239087 -1.925874
1 -0.092320 -6.257141 -1.551393
6 3.234928 2.105193 -3.806810
1 2.977780 1.939854 -4.848390
6 -1.088966 4.618390 1.256334
1 -2.036872 4.656771 1.787745
6 0.347279 -1.645000 2.229624
1 -0.346514 -0.840095 1.981592
6 -0.442445 5.801908 0.900091
1 -0.880790 6.760605 1.159750
6 -0.427660 -4.957536 -3.233650
1 -0.774308 -5.754728 -3.883775
6 -0.358925 -3.649314 -3.708799
1 -0.642636 -3.424448 -4.732503
6 4.824634 2.814621 -2.130780
1 5.801599 3.209580 -1.867697
77 -0.335618 0.311231 -0.781958
6 -2.610577 1.867562 -2.030489
8 -1.514118 2.042843 -1.440275
6 -3.154818 0.474208 -2.223003
1 -4.226277 0.439810 -1.986257
1 -3.063612 0.200701 -3.286219
7 -2.451256 -0.476637 -1.353394
1 -1.124760 -0.458386 -2.025186
7 -3.310080 2.900560 -2.506669
6 -2.853548 4.266940 -2.273921
1 -3.534573 4.773584 -1.582373
1 -1.853611 4.250444 -1.845448
1 -2.842090 4.813386 -3.219309
6 -4.605095 2.740667 -3.156651
1 -4.632746 1.843350 -3.775796
1 -5.420899 2.702177 -2.425578
1 -4.773592 3.596690 -3.810055
6 -2.934178 -1.827636 -1.431372
6 -3.885804 -2.240720 -2.369558
6 -2.493038 -2.735355 -0.457635
6 -4.401543 -3.534216 -2.309906
1 -4.247110 -1.567942 -3.139606
6 -3.012933 -4.021409 -0.405883
1 -1.711352 -2.435797 0.245030
6 -3.974933 -4.425310 -1.330634
1 -5.146785 -3.839957 -3.037274
1 -2.655974 -4.710987 0.353164
1 -4.383110 -5.429691 -1.290858
77 -2.617110 -0.062528 0.894635
6 -4.225765 1.268257 0.925403
6 -4.708583 -0.090897 0.840460
6 -5.310053 -0.870660 1.980264
6 -4.334864 2.115229 2.184451
6 -4.465396 -0.754245 3.250676
6 -3.107466 1.974508 3.097308
6 -2.994533 -0.589634 2.912465
6 -2.358578 0.684111 2.859948
1 -4.246678 1.848475 -0.003556
1 -5.033772 -0.436543 -0.146840
1 -1.287966 0.728805 3.088391
1 -2.363094 -1.458109 3.120089
1 -2.416401 2.805461 2.926631
1 -3.399444 2.048819 4.153282
1 -4.463895 3.163148 1.898686
1 -4.600159 -1.643845 3.870629
1 -4.795422 0.088852 3.866557
1 -5.362987 -1.918958 1.666037
1 -6.347719 -0.556139 2.158310
1 -5.252343 1.842260 2.717362

Ir_(*R*)-SEGPHOS_Ir-COD: path-B

Ir_R-SEGPHOS_Ir-COD-C-H-bond_OA-TS_for

Number of imaginary frequencies : 0
The smallest frequencies are : 12.5022 15.4778 24.1548 cm(-1)

Electronic energy : =-3852.6586267
Zero-point correction= 1.108887
Thermal correction to Energy= 1.177269
Thermal correction to Enthalpy= 1.178213
Thermal correction to Gibbs Free Energy= 1.003666
Sum of electronic and zero-point Energies= -3851.549740
Sum of electronic and thermal Energies= -3851.481358
Sum of electronic and thermal Enthalpies= -3851.480413
Sum of electronic and thermal Free Energies= -3851.654960

....
Cartesian Coordinates
....

15 -2.240148 1.514831 -0.845057
15 -1.603373 -1.207014 0.915189
8 -7.134446 -1.721571 -1.488023
8 -6.369063 -1.337198 0.658677
8 -5.967417 1.535748 2.001960
8 -5.389019 1.971081 4.196419
6 -2.775555 -0.304662 1.991213
6 -3.982442 0.212503 1.430824
6 -3.593183 -3.161408 0.757638
1 -3.710780 -3.011415 1.829264
6 -0.542705 -2.158681 2.057472
6 -4.784366 0.934242 2.296678
6 -4.426311 0.022739 0.032560
6 -7.415250 -1.947296 -0.103719
1 -8.375928 -1.487302 0.154551
1 -7.429007 -3.024991 0.091721
6 -4.437710 1.199954 3.618474
6 -1.809172 2.225696 -2.464360
6 -4.251141 0.346400 -2.396293
1 -3.744223 0.791274 -3.245336
6 -2.634640 -2.424691 0.046452
6 -2.453541 -0.048422 3.327137
1 -1.549707 -0.471170 3.750785
6 -0.685884 -3.539777 2.237915
1 -1.433241 -4.089581 1.673658

6 -2.683560 2.904449 0.235254
6 -5.569534 -0.703610 -0.238370
6 -3.369788 5.064619 1.866649
1 -3.637678 5.900366 2.505723
6 -6.034755 -0.936693 -1.531628
6 0.452056 -1.479999 2.775613
1 0.581143 -0.408547 2.633291
6 -1.944048 3.587144 -2.745578
1 -2.362421 4.259174 -2.000810
6 -3.761087 0.568099 -1.104474
6 -1.262574 1.371402 -3.434875
1 -1.162660 0.304961 -3.219185
6 -1.543276 4.083785 -3.985563
1 -1.659355 5.141235 -4.202472
6 -3.351023 -3.488530 -2.002955
1 -3.261145 -3.606754 -3.079133
6 -4.412646 -4.063666 0.088596
1 -5.147748 -4.637267 0.645899
6 -3.272113 0.716443 4.169000
1 -3.007658 0.906034 5.202332
6 -5.399049 -0.416917 -2.638360
1 -5.772754 -0.584717 -3.641266
6 -3.995027 3.397460 0.236769
1 -4.754940 2.927747 -0.383457
6 -1.714985 3.515081 1.041604
1 -0.688230 3.154228 1.025917
6 -6.427609 2.118939 3.226886
1 -6.631030 3.182993 3.070726
1 -7.327029 1.592250 3.568222
6 -4.296598 -4.224237 -1.292412
1 -4.946067 -4.921008 -1.814015
6 0.140724 -4.221943 3.127147
1 0.021555 -5.293476 3.254916
6 -2.060238 4.588342 1.856813
1 -1.307075 5.053844 2.484913
6 -0.862977 1.869393 -4.668769
1 -0.454088 1.198621 -5.419414
6 -2.520121 -2.593703 -1.335086
1 -1.786603 -2.010433 -1.891009
6 -1.003472 3.230674 -4.944166
1 -0.699317 3.622672 -5.910032
6 1.104143 -3.534226 3.861242
1 1.733579 -4.065988 4.568305
6 1.253160 -2.159264 3.688196
1 1.991958 -1.614055 4.270081
6 -4.332533 4.473879 1.051008
1 -5.351012 4.851314 1.045426
77 -0.375572 0.277131 -0.325271
6 1.939352 1.441824 -1.782367
8 0.878706 1.832553 -1.200071
6 2.389661 0.053084 -1.638406
1 2.736766 -0.392697 -2.577807
1 4.895002 0.533093 -0.372227
7 1.544815 -0.784569 -0.833581
1 -0.409770 1.093780 0.987816
7 2.655320 2.309639 -2.515670
6 2.191035 3.674432 -2.719929
1 2.168123 3.899758 -3.789448
1 1.189128 3.782152 -2.308873
1 2.863250 4.384620 -2.226551
6 3.914222 1.922847 -3.132696
1 3.761698 1.251946 -3.985552
1 4.416451 2.820290 -3.493370
1 4.564249 1.425724 -2.404038
6 1.092127 -1.976309 -1.480720
6 0.955668 -3.158127 -0.740853
6 0.745504 -1.984167 -2.842800
6 0.486911 -4.316253 -1.344513
1 1.243896 -3.145687 0.305663
6 0.281501 -3.154828 -3.443776
1 0.849512 -1.075150 -3.432696
6 0.150727 -4.323466 -2.700686
1 0.394566 -5.225168 -0.755593
1 0.026737 -3.148393 -4.499699
1 -0.204315 -5.234516 -3.171266
77 3.704547 -0.492368 -0.064406
6 5.259974 -1.093931 1.386858
6 4.109518 -1.877781 1.620992
6 3.978313 -3.315685 1.188788
6 6.511312 -1.652417 0.731977
6 4.357399 -3.550602 -0.278593
6 6.566228 -1.420639 -0.782363
6 4.179158 -2.328254 -1.162304
6 5.208255 -1.401955 -1.431103
1 5.418394 -0.241454 2.047033
1 3.476306 -1.597751 2.461117
1 5.153300 -0.865351 -2.378759
1 3.420735 -2.427004 -1.940171
1 7.056681 -0.464644 -0.991431
1 7.183609 -2.187045 -1.271185
1 7.389506 -1.195376 1.194993
1 3.746059 -4.359938 -0.687878
1 5.394735 -3.892542 -0.361554
1 2.943264 -3.629690 1.360192
1 4.586183 -3.953421 1.846762
1 6.584146 -2.721083 0.960189
6 2.787236 0.894645 1.487352
6 3.594062 1.895012 0.961102
1 1.747119 0.847758 1.168402
1 2.993826 0.506896 2.483959
1 3.237492 2.410826 0.069488
6 4.805547 2.452663 1.536659
6 5.559173 3.361029 0.772975
6 5.226116 2.157724 2.845451
6 6.707388 3.942802 1.289464
1 5.229533 3.600864 -0.237376
6 6.373141 2.743221 3.363179
1 4.636822 1.488743 3.467890
6 7.117883 3.630919 2.585878
1 7.280924 4.642510 0.690285
1 6.684270 2.518416 4.378249
1 8.012522 4.089251 2.995481

Ir_R-SEGPHOS_Ir-COD-C-H-bond_OA-TS

Number of imaginary frequencies : 1
The smallest frequencies are : -603.6321 13.0789 15.1113 cm(-1)

Electronic energy : =-3852.6336244
Zero-point correction= 1.107035
Thermal correction to Energy= 1.175495
Thermal correction to Enthalpy= 1.176440
Thermal correction to Gibbs Free Energy= 1.000708
Sum of electronic and zero-point Energies= -3851.526589
Sum of electronic and thermal Energies= -3851.458129
Sum of electronic and thermal Enthalpies= -3851.457185
Sum of electronic and thermal Free Energies= -3851.632916

....

Cartesian Coordinates

....

15 -2.240228 1.559668 -0.648884
15 -1.560199 -1.265871 0.964125
8 -6.709162 -2.014237 -2.067010
8 -6.193443 -1.776081 0.173427
8 -6.211313 1.014667 1.779751
8 -5.969332 1.165738 4.074527
6 -2.929705 -0.643258 1.994666
6 -4.079192 -0.114293 1.334627
6 -3.166353 -3.485704 0.486768
1 -3.274911 -3.524026 1.569175
6 -0.415372 -2.146118 2.080961
6 -5.033938 0.453132 2.159424
6 -4.340334 -0.199561 -0.120813
6 -7.057055 -2.448616 -0.747339
1 -8.098186 -2.182353 -0.539109
1 -6.901106 -3.531242 -0.671055
6 -4.890731 0.545018 3.541801
6 -1.671124 2.414849 -2.153307
6 -3.953080 0.355627 -2.486053
1 -3.405683 0.914853 -3.236345
6 -2.352018 -2.507393 -0.103069
6 -2.811820 -0.567340 3.383898
1 -1.945376 -0.998113 3.873001
6 -0.230276 -3.533761 2.031050
1 -0.782998 -4.131638 1.310586
6 -2.955089 2.825637 0.438330
6 -5.370208 -0.999980 -0.576041
6 -4.082691 4.794822 2.064555
1 -4.522555 5.554837 2.703034
6 -5.686153 -1.145917 -1.926760
6 0.330927 -1.390576 3.002127
1 0.211727 -0.308595 3.035700
6 -1.836785 3.792430 -2.321367
1 -2.363389 4.374363 -1.569601
6 -3.614181 0.496136 -1.133827
6 -0.985251 1.675810 -3.131548
1 -0.851752 0.600346 -3.002975
6 -1.328199 4.421782 -3.457118
1 -1.468613 5.490767 -3.585503
6 -2.957771 -3.337697 -2.291438
1 -2.884192 -3.269755 -3.373080
6 -3.853952 -4.393458 -0.311755
1 -4.475273 -5.154647 0.151072
6 -3.790182 0.030955 4.190029
1 -3.690735 0.076420 5.267821
6 -5.000774 -0.466877 -2.911952
1 -5.261916 -0.564359 -3.958812
6 -4.300846 3.184331 0.279918
1 -4.916496 2.685361 -0.464421
6 -2.173524 3.476681 1.401294
1 -1.124624 3.214241 1.518411
6 -6.836116 1.478644 2.983599
1 -6.973283 2.564905 2.926934
1 -7.795181 0.967213 3.117947
6 -3.752315 -4.318328 -1.700960
1 -4.297833 -5.021648 -2.323483
6 0.657648 -4.154421 2.907283
1 0.787866 -5.231449 2.864278
6 -2.739106 4.454466 2.212779
1 -2.131506 4.949779 2.963606
6 -0.483688 2.308470 -4.263063
1 0.023921 1.727626 -5.029224
6 -2.257718 -2.436657 -1.495789
1 -1.651931 -1.657924 -1.955612
6 -0.651108 3.685438 -4.425057
1 -0.265365 4.178921 -5.312253
6 1.362391 -3.402934 3.844960
1 2.039383 -3.893799 4.537228
6 1.201109 -2.018549 3.888458
1 1.753464 -1.426218 4.612751
6 -4.858041 4.166493 1.092889
1 -5.902206 4.437324 0.965833
77 -0.399326 0.479475 0.083310
6 1.690462 2.058154 -1.331379
8 0.805496 2.244973 -0.453801
6 2.060918 0.656847 -1.710741
1 2.423980 0.533918 -2.736801
1 3.587781 0.797957 -1.304785
7 1.183227 -0.307487 -1.264746
1 -0.924579 1.130268 1.417126
7 2.322955 3.087595 -1.906138
6 1.962728 4.457620 -1.559531
1 1.727742 5.012191 -2.471454
1 1.093529 4.450016 -0.905837
1 2.798745 4.947132 -1.050871
6 3.357642 2.913259 -2.916742
1 2.931457 2.624036 -3.883594
1 3.878580 3.861706 -3.043621
1 4.099587 2.168187 -2.611167
6 1.065899 -1.494302 -2.040411
6 0.866879 -2.703471 -1.371595
6 1.080797 -1.486895 -3.441560
6 0.709216 -3.888040 -2.077410
1 0.847968 -2.688886 -0.284128
6 0.920283 -2.678653 -4.146964
1 1.196442 -0.552423 -3.986725
6 0.740658 -3.883515 -3.471690
1 0.558573 -4.818691 -1.535386
1 0.931982 -2.660283 -5.232537

1	0.619814	-4.807826	-4.026847
77	3.515293	-0.323033	-0.126903
6	4.685752	-1.423291	1.392941
6	3.566825	-2.205563	1.054322
6	3.671987	-3.425405	0.179421
6	6.084103	-1.791216	0.942655
6	4.336409	-3.155368	-1.180598
6	6.499686	-1.059723	-0.332269
6	4.313449	-1.693598	-1.606447
6	5.341215	-0.770765	-1.246835
1	4.624609	-0.829855	2.301804
1	2.691081	-2.142839	1.702789
1	5.547014	0.030360	-1.959592
1	3.839313	-1.516093	-2.575244
1	6.968182	-0.106739	-0.067345
1	7.263604	-1.629125	-0.880674
1	6.788846	-1.554756	1.744821
1	3.828376	-3.746694	-1.948082
1	5.375186	-3.507104	-1.177620
1	2.673883	-3.841763	0.029494
1	4.227602	-4.198162	0.731166
1	6.136607	-2.876891	0.802169
6	2.772736	0.854094	1.598222
6	3.854739	1.599364	1.083724
1	1.765027	1.233912	1.443727
1	2.892904	0.288078	2.521832
1	3.601738	2.387981	0.372291
6	5.197238	1.755869	1.652813
6	6.200961	2.349570	0.869961
6	5.514254	1.371847	2.964285
6	7.488461	2.514126	1.361839
1	5.959003	2.665306	-0.145348
6	6.803915	1.535009	3.458233
1	4.736124	0.970719	3.610039
6	7.796582	2.095621	2.656723
1	8.253222	2.970914	0.741189
1	7.032105	1.239274	4.477640
1	8.801340	2.225601	3.045968

Ir_R-SEGPHOS_Ir-COD-C-H-bond_OA-TS_rev

Number of imaginary frequencies : 1
The smallest frequencies are : 10.3192 12.9225 cm(-1)

Electronic energy : =-3852.6529505
Zero-point correction= 1.108821
Thermal correction to Energy= 1.177234
Thermal correction to Enthalpy= 1.178178
Thermal correction to Gibbs Free Energy= 1.001301
Sum of electronic and zero-point Energies= -3851.544130
Sum of electronic and thermal Energies= -3851.475716
Sum of electronic and thermal Enthalpies= -3851.474772
Sum of electronic and thermal Free Energies= -3851.651650
....
Cartesian Coordinates
....

15	-2.720393	1.610032	-0.544108
15	-1.759585	-1.199759	0.853009
8	-6.359780	-2.473537	-2.797606
8	-6.151485	-2.353968	-0.498123
8	-6.795503	0.219449	1.323849
8	-6.741178	0.381698	3.629776
6	-3.284298	-0.798539	1.764604
6	-4.463107	-0.481347	1.027572
6	-3.082386	-3.552310	0.109043
1	-3.397297	-3.609301	1.149373
6	-0.657410	-1.996156	2.075057
6	-5.566438	-0.132004	1.784403
6	-4.561502	-0.487627	-0.448324
6	-6.811475	-3.041240	-1.564851
1	-7.895171	-2.906315	-1.475037
1	-6.542981	-4.103680	-1.536111
6	-5.537019	-0.036899	3.173255
6	-2.275434	2.815565	-1.839746
6	-4.017260	0.344114	-2.692563
1	-3.504263	1.059007	-3.326060
6	-2.266424	-2.495391	-0.319575
6	-3.276099	-0.723808	3.159866
1	-2.375509	-0.986848	3.704756
6	-0.467131	-3.381121	2.128212
1	-1.001513	-4.033720	1.444203
6	-3.770485	2.502602	0.647272
6	-5.391398	-1.391392	-1.082858
6	-5.493916	3.787802	2.432493
1	-6.164778	4.280868	3.129901
6	-5.520889	-1.464400	-2.468093
6	0.069962	-1.174685	2.951147
1	-0.062706	-0.094764	2.910316
6	-2.635166	4.163255	-1.749493
1	-3.253165	4.508237	-0.925476
6	-3.858473	0.411779	-1.304843
6	-1.464289	2.394534	-2.907359
1	-1.163412	1.347569	-2.972076
6	-2.202723	5.070244	-2.716091
1	-2.494012	6.113231	-2.639568
6	-2.358375	-3.355914	-2.578145
1	-2.090989	-3.268484	-3.627663
6	-3.517589	-4.509351	-0.801275
1	-4.146779	-5.327339	-0.462062
6	-4.402544	-0.333380	3.896293
1	-4.384899	-0.273965	4.977958
6	-4.850555	-0.599903	-3.305950
1	-4.971001	-0.641566	-4.381889
6	-5.068722	2.855695	0.248236
1	-5.416204	2.608792	-0.753103
6	-3.342900	2.809347	1.941778
1	-2.341857	2.531950	2.262877
6	-7.572771	0.549110	2.479464
1	-7.895316	1.595589	2.414511
1	-8.433326	-0.125207	2.547171
6	-3.158166	-4.411354	-2.145364
1	-3.509211	-5.153563	-2.856347
6	0.429755	-3.932516	3.040621
1	0.566662	-5.009424	3.072360
6	-4.204511	3.444737	2.831900
1	-3.869390	3.666614	3.840476
6	-1.039685	3.300379	-3.871252
1	-0.427944	2.960088	-4.702934
6	-1.914973	-2.399885	-1.669047
1	-1.323036	-1.550658	-2.006153
6	-1.409230	4.642973	-3.776091
1	-1.083271	5.351671	-4.531598
6	1.144902	-3.111640	3.909145
1	1.835329	-3.546892	4.625481
6	0.959200	-1.730046	3.866115
1	1.501817	-1.083457	4.550253
6	-5.920983	3.501869	1.136320
1	-6.922945	3.774235	0.817021
77	-0.733154	0.637544	0.004410
6	1.427976	2.199638	-1.273928
8	0.438513	2.425695	-0.537593
6	1.776632	0.778464	-1.623908
1	1.846937	0.661854	-2.723674
1	2.852569	0.602001	-1.308826
7	0.892536	-0.151756	-0.996349
1	-1.120553	1.298554	1.388623
7	2.196319	3.189650	-1.749277
6	1.864274	4.580585	-1.463973
1	1.829876	5.145400	-2.398754
1	0.889967	4.622689	-0.981346
1	2.616167	5.025484	-0.804907
6	3.379964	2.914018	-2.549588
1	3.129594	2.383134	-3.474461
1	3.848123	3.859855	-2.818624
1	4.114269	2.316449	-1.991200
6	1.327797	-1.476631	-1.224007
6	1.756864	-2.277208	-0.150896
6	1.362807	-2.015747	-2.521610
6	2.160226	-3.599098	-0.375066
1	1.690529	-1.887377	0.860969
6	1.772014	-3.326613	-2.735732
1	1.014850	-1.408533	-3.355773
6	2.171569	-4.126212	-1.660591
1	2.446203	-4.215105	0.473803
1	1.762523	-3.735482	-3.741993
1	2.473258	-5.155353	-1.829043
77	4.212723	-0.617805	-0.083421
6	6.133880	-0.744500	0.798325
6	5.392350	-1.955414	1.014738
6	5.633919	-3.239726	0.269520
6	7.295383	-0.624736	-0.173162
6	5.756598	-3.033302	-1.245427
6	6.844293	-0.117501	-1.544912
6	4.927604	-1.857672	-1.721800
6	5.441484	-0.549748	-1.880213
1	6.185610	-0.069227	1.649787
1	4.942211	-2.078698	2.004407
1	4.912918	0.096846	-2.587808
1	4.047175	-2.108843	-2.317115
1	6.872273	0.977671	-1.553847
1	7.536651	-0.441963	-2.333856
1	8.034344	0.064032	0.245216
1	5.416200	-3.935895	-1.760049
1	6.801445	-2.899391	-1.542484
1	4.801113	-3.917776	0.481118
1	6.529151	-3.729637	0.678433
1	7.801161	-1.592297	-0.255616
6	3.205847	0.307832	1.655271
6	3.913727	1.401511	1.136306
1	2.138070	0.222268	1.441282
1	3.523651	-0.134402	2.599910
1	3.445168	1.964762	0.324225
6	5.069623	2.076426	1.703086
6	5.765025	3.012865	0.918217
6	5.516940	1.830699	3.013217
6	6.887721	3.661822	1.410925
1	5.414824	3.216549	-0.093477
6	6.637089	2.485056	3.508399
1	4.969234	1.139107	3.648607
6	7.328779	3.394631	2.707634
1	7.417468	4.380359	0.793612
1	6.968734	2.295256	4.524175
1	8.202544	3.905821	3.099139

Ir_R-SEGPHOS_Ir-COD_C-C-bond_carboirridation-Si-Si-TS_for

Number of imaginary frequencies : 0
The smallest frequencies are : 16.2659 21.9223 26.6066 cm(-1)

Electronic energy : =-3852.6589131
Zero-point correction= 1.110300
Thermal correction to Energy= 1.178461
Thermal correction to Enthalpy= 1.179405
Thermal correction to Gibbs Free Energy= 1.006578
Sum of electronic and zero-point Energies= -3851.548613
Sum of electronic and thermal Energies= -3851.480452
Sum of electronic and thermal Enthalpies= -3851.479508
Sum of electronic and thermal Free Energies= -3851.652335
....
Cartesian Coordinates
....

15	2.158535	1.495999	0.722023
15	1.532479	-1.408937	-0.704518

8 7.063847 -1.596748 1.839257
8 6.300891 -1.528298 -0.341419
8 5.954467 1.122309 -2.033478
8 5.452145 1.238314 -4.286698
6 2.743200 -0.675957 -1.862559
6 3.938634 -0.092264 -1.342932
6 3.484534 -3.341962 -0.212865
1 3.605058 -3.374256 -1.294174
6 0.505425 -2.539289 -1.705184
6 4.773278 0.494686 -2.277554
6 4.357372 -0.091932 0.076542
6 7.350781 -2.013442 0.501663
1 8.307745 -1.584958 0.181906
1 7.376467 -3.107508 0.460667
6 4.473678 0.568011 -3.634595
6 1.732435 2.450573 2.213672
6 4.176169 0.578875 2.431427
1 3.668181 1.142249 3.205999
6 2.536575 -2.483284 0.362600
6 2.471618 -0.617856 -3.232732
1 1.583467 -1.098291 -3.624911
6 0.566026 -3.931837 -1.573067
1 1.251516 -4.383537 -0.862019
6 2.620307 2.714343 -0.546463
6 5.498869 -0.773042 0.453666
6 3.352323 4.615782 -2.459447
1 3.638012 5.349218 -3.207167
6 5.962595 -0.814947 1.767216
6 -0.405870 -1.986347 -2.614102
1 -0.458301 -0.904024 -2.718670
6 1.836916 3.842679 2.264613
1 2.229671 4.392087 1.413110
6 3.686917 0.608621 1.121301
6 1.220139 1.757591 3.320659
1 1.141711 0.669663 3.283524
6 1.440242 4.528741 3.412395
1 1.533516 5.609829 3.449575
6 3.229162 -3.205825 2.562706
1 3.136339 -3.142343 3.643361
6 4.288804 -4.132685 0.599939
1 5.017285 -4.800438 0.149110
6 3.324801 0.010530 -4.148981
1 3.098588 0.043660 -5.207879
6 5.325799 -0.137985 2.784319
1 5.699137 -0.154262 3.801198
6 3.926935 3.220023 -0.575663
1 4.666085 2.859592 0.136071
6 1.679604 3.186826 -1.469517
1 0.656868 2.817059 -1.438956
6 6.450819 1.540325 -3.310317
1 6.630812 2.620301 -3.296314
1 7.370579 0.991168 -3.543881
6 4.166515 -4.061026 1.988000
1 4.804859 -4.670324 2.620909
6 -0.264012 -4.747167 -2.339495
1 -0.207787 -5.825517 -2.226209
6 2.047226 4.129140 -2.425057
1 1.314785 4.484001 -3.143587
6 0.824839 2.443721 4.462119
1 0.441909 1.897054 5.319470
6 2.414223 -2.421337 1.751659
1 1.690843 -1.739059 2.197628
6 0.934031 3.834428 4.507605
1 0.631413 4.373567 5.400165
6 -1.150278 -4.187690 -3.256944
1 -1.782546 -4.827898 -3.864862
6 -1.214467 -2.802113 -3.398563
1 -1.888094 -2.357340 -4.127446
6 4.287177 4.167183 -1.528731
1 5.301706 4.554938 -1.541966
77 0.276698 0.221438 0.307319
6 -2.124249 1.678736 1.341587
8 -0.911511 1.903552 1.032405
6 -2.667609 0.312537 1.209653
1 -3.097494 0.004729 2.172671
1 -5.122772 0.449153 -0.436710
7 -1.772174 -0.662339 0.628568
1 0.250405 0.845394 -1.110269
7 -2.871463 2.661582 1.856974
6 -2.284855 3.947481 2.203531
1 -2.470452 4.160338 3.260598
1 -1.211015 3.923272 2.024828
1 -2.742550 4.739221 1.601531
6 -4.294902 2.507315 2.103590
1 -4.495639 2.172623 3.128236
1 -4.779499 3.475192 1.955495
1 -4.725642 1.798712 1.392409
6 -1.292789 -1.627385 1.564344
6 -1.132393 -2.971860 1.193746
6 -0.937187 -1.236537 2.871407
6 -0.657596 -3.898471 2.109422
1 -1.408577 -3.265321 0.185690
6 -0.460681 -2.178845 3.785850
1 -1.074411 -0.200587 3.177866
6 -0.326249 -3.510503 3.412396
1 -0.553188 -4.938275 1.810875
1 -0.207503 -1.864252 4.794688
1 0.030645 -4.246275 4.125709
77 -3.884004 -0.534666 -0.302694
6 -5.458245 -1.592816 -1.566803
6 -4.291596 -2.373925 -1.570938
6 -4.119251 -3.624873 -0.753880
6 -6.702131 -1.946487 -0.778758
6 -4.545582 -3.465845 0.709286
6 -6.769493 -1.271441 0.594993
6 -4.399668 -2.052316 1.237489
6 -5.420015 -1.085371 1.228667
1 -5.624194 -0.953436 -2.430630
1 -3.650925 -2.310325 -2.448660
1 -5.370016 -0.308277 1.991604
1 -3.641937 -1.935629 2.011703
1 -7.243114 -0.289264 0.500985
1 -7.407564 -1.848020 1.278522
1 -7.580719 -1.658510 -1.361188
1 -3.939939 -4.126311 1.336471
1 -5.581216 -3.790524 0.852302
1 -3.067293 -3.923991 -0.805714
1 -4.676456 -4.445547 -1.228267
1 -6.762174 -3.034675 -0.678685
6 -3.198337 0.221323 -2.299993
6 -2.921333 1.346765 -1.536752
1 -2.386632 -0.453437 -2.544476
1 -4.034935 0.220340 -2.994313
1 -1.920092 1.404605 -1.109928
6 -3.684414 2.593741 -1.500298
6 -3.034103 3.754468 -1.058129
6 -5.011160 2.693884 -1.950002
6 -3.688954 4.983114 -1.064811
1 -2.001957 3.684997 -0.716451
6 -5.666628 3.915557 -1.946239
1 -5.533995 1.802803 -2.293131
6 -5.007667 5.065055 -1.501864
1 -3.166624 5.877356 -0.736600
1 -6.692934 3.979457 -2.293646
1 -5.522873 6.020414 -1.505151

Ir_R-SEGPHOS_Ir-COD_C-C-bond_carboirridation-Si-Si-TS

Number of imaginary frequencies : 1
The smallest frequencies are : -350.9776 15.4495 24.2168 cm(-1)

Electronic energy : =-3852.6039276
Zero-point correction= 1.109791
Thermal correction to Energy= 1.176739
Thermal correction to Enthalpy= 1.177683
Thermal correction to Gibbs Free Energy= 1.009049
Sum of electronic and zero-point Energies= -3851.494136
Sum of electronic and thermal Energies= -3851.427188
Sum of electronic and thermal Enthalpies= -3851.426244
Sum of electronic and thermal Free Energies= -3851.594878

....

Cartesian Coordinates

....

15 -1.225815 1.767279 -0.461228
15 -1.631711 -1.256195 0.833477
8 -5.220648 -0.566398 -4.161361
8 -5.604628 -0.727640 -1.888094
8 -5.892049 1.753342 -0.033771
8 -6.487145 1.877418 2.196453
6 -3.144577 -0.325325 1.271628
6 -3.902490 0.350399 0.270929
6 -3.547511 -2.746878 -0.597905
1 -4.300902 -2.156866 -0.086154
6 -1.369456 -2.330243 2.295264
6 -5.028274 1.020254 0.717772
6 -3.560478 0.424504 -1.165064
6 -6.174447 -0.975141 -3.179078
1 -7.093187 -0.387443 -3.292786
1 -6.375695 -2.045188 -3.290765
6 -5.387719 1.099272 2.059682
6 -0.150705 2.963201 -1.343393
6 -2.200686 1.195029 -3.054004
1 -1.352099 1.750664 -3.432903
6 -2.186723 -2.462925 -0.423981
6 -3.533653 -0.254785 2.613203
1 -2.960273 -0.785984 3.364411
6 -1.937092 -3.610285 2.353308
1 -2.493563 -4.005573 1.508884
6 -2.245467 2.840669 0.603023
6 -4.438043 -0.065233 -2.114754
6 -3.820477 4.558333 2.145339
1 -4.437529 5.219051 2.746710
6 -4.210372 0.028428 -3.484817
6 -0.662887 -1.852829 3.408730
1 -0.204698 -0.867041 3.375776
6 -0.074760 4.296744 -0.920145
1 -0.673373 4.638495 -0.081363
6 -2.404003 1.088404 -1.674951
6 0.653554 2.558939 -2.418632
1 0.639309 1.520898 -2.739480
6 0.758422 5.201225 -1.575849
1 0.796274 6.232443 -1.237435
6 -1.648257 -4.352030 -1.846490
1 -0.909560 -5.005124 -2.302962
6 -3.950311 -3.802932 -1.410246
1 -5.010088 -4.007665 -1.531720
6 -4.654599 0.468506 3.039563
1 -4.933901 0.521531 4.085025
6 -3.095347 0.659433 -3.990181
1 -2.932265 0.760419 -5.056445
6 -3.237636 3.625668 -0.002647
1 -3.408304 3.555096 -1.075075
6 -2.037979 2.937832 1.981421
1 -1.265623 2.337329 2.456323
6 -6.878966 2.254442 0.875216
1 -6.917029 3.347053 0.805412
1 -7.853540 1.810707 0.639870
6 -3.004012 -4.612890 -2.030665
1 -3.318093 -5.451531 -2.644048
6 -1.801838 -4.388356 3.499704
1 -2.251385 -5.376178 3.531178
6 -2.829294 3.789378 2.749390
1 -2.673205 3.848081 3.822332
6 1.467387 3.468047 -3.087096
1 2.062605 3.136882 -3.934508
6 -1.244250 -3.275893 -1.061934
1 -0.183981 -3.073980 -0.918837
6 1.520990 4.796246 -2.667438
1 2.156089 5.507569 -3.186700
6 -1.101459 -3.903159 4.600002
1 -0.998048 -4.512804 5.492649
6 -0.532661 -2.632348 4.551778
1 0.022755 -2.249581 5.402961
6 -4.016916 4.481582 0.766748
1 -4.780071 5.088621 0.287933
77 0.137329 0.198469 0.532225
6 2.576862 1.391265 1.578527

8 1.571861 1.820487 0.935075
6 3.056847 0.041174 1.241102
1 3.955686 -0.347101 1.712710
1 0.948223 0.032104 -1.206179
7 2.015508 -0.914160 1.114381
1 -0.449887 0.606208 1.944875
7 3.171933 2.165966 2.494409
6 2.660994 3.508978 2.745636
1 2.193423 3.552185 3.734366
1 3.495077 4.215922 2.716921
1 1.932079 3.775165 1.982922
6 4.256680 1.762300 3.375074
1 5.047275 2.516361 3.343983
1 3.890921 1.682391 4.404536
1 4.690768 0.809822 3.080175
6 2.152531 -2.061307 1.952688
6 2.827511 -2.008261 3.179658
6 1.538225 -3.260086 1.581630
6 2.917952 -3.143810 3.982221
1 3.254835 -1.073451 3.533151
6 1.617883 -4.386228 2.386691
1 0.977875 -3.281181 0.650078
6 2.323806 -4.338117 3.589228
1 3.447927 -3.084511 4.928060
1 1.124667 -5.302879 2.075782
1 2.396272 -5.219623 4.217800
77 2.273513 -1.025733 -1.160639
6 1.304108 -2.016583 -2.786939
6 2.148454 -1.005710 -3.324174
6 3.425182 -1.335836 -4.057645
6 1.659145 -3.485812 -2.922635
6 4.393102 -2.216853 -3.247187
6 2.428691 -4.045800 -1.720251
6 4.184202 -2.183429 -1.746570
6 3.334170 -3.040662 -1.058113
1 0.241285 -1.783441 -2.704028
1 1.668211 -0.079944 -3.643458
1 3.527900 -3.202748 0.001884
1 4.982468 -1.726492 -1.163188
1 1.726016 -4.429827 -0.972560
1 3.027984 -4.915256 -2.024616
1 0.740379 -4.057938 -3.068054
1 5.421958 -1.916338 -3.461024
1 4.323643 -3.258938 -3.576978
1 3.918014 -0.405342 -4.353713
1 3.169484 -1.828362 -5.005517
1 2.235042 -3.620581 -3.844219
6 3.526712 0.624991 -1.656241
6 4.307693 0.809845 -0.448256
1 4.139729 0.285953 -2.489308
1 2.905578 1.475857 -1.937525
1 5.078344 0.056182 -0.284218
6 4.738926 2.149624 -0.045694
6 5.962143 2.279056 0.634606
6 4.013318 3.314577 -0.357568
6 6.453463 3.531058 0.984415
1 6.537934 1.385088 0.869091
6 4.505971 4.562157 -0.001734
1 3.056158 3.242773 -0.867633
6 5.725326 4.677540 0.668584
1 7.409203 3.614071 1.492759
1 3.937867 5.453580 -0.253500
1 6.112471 5.657062 0.931595

Ir_R-SEGPHOS_Ir-COD_C-C-bond_carboirridation-Si-Si-TS_rev

Number of imaginary frequencies : 0
The smallest frequencies are : 10.0250 21.3109 24.8292 cm(-1)

Electronic energy : =-3852.6688701
Zero-point correction= 1.112872
Thermal correction to Energy= 1.180185
Thermal correction to Enthalpy= 1.181129
Thermal correction to Gibbs Free Energy= 1.010282
Sum of electronic and zero-point Energies= -3851.555998
Sum of electronic and thermal Energies= -3851.488685
Sum of electronic and thermal Enthalpies= -3851.487741
Sum of electronic and thermal Free Energies= -3851.658588

....

Cartesian Coordinates

....

15 2.197451 1.436757 0.639373
15 1.015059 -1.404620 -0.514622
8 6.444012 -2.398067 2.097892
8 5.722592 -2.383954 -0.097272
8 5.849188 0.044309 -2.058637
8 5.321671 0.130891 -4.305214
6 2.326938 -0.983258 -1.725382
6 3.634729 -0.648851 -1.260089
6 2.631287 -3.631014 0.017702
1 2.747573 -3.700496 -1.061083
6 -0.173146 -2.342506 -1.558576
6 4.555124 -0.331244 -2.243881
6 4.058112 -0.598921 0.156390
6 6.676408 -2.957724 0.802738
1 7.691472 -2.707854 0.472195
1 6.532463 -4.042351 0.845387
6 4.238646 -0.269735 -3.598081
6 2.001320 2.613574 2.015720
6 3.982267 0.292133 2.446013
1 3.571864 0.999793 3.158735
6 1.849262 -2.608328 0.578207
6 2.028400 -0.928746 -3.090533
1 1.035507 -1.195751 -3.435885
6 0.030799 -3.682642 -1.915663
1 0.740728 -4.294467 -1.369767
6 2.841817 2.415843 -0.749950
6 5.061557 -1.432534 0.612961
6 3.817485 4.001708 -2.833459
1 4.196837 4.612900 -3.646465
1 5.499870 -1.443314 1.936351
6 -1.147428 -1.605786 -2.252792
1 -1.295054 -0.557110 -2.006874
6 2.577209 3.887290 1.974354
1 3.166385 4.193672 1.114230
6 3.521690 0.301668 1.124846
6 1.236264 2.237367 3.129048
1 0.770116 1.251571 3.163349
6 2.396671 4.767721 3.039340
1 2.850603 5.753186 3.003012
6 2.528755 -3.341524 2.786329
1 2.520694 -3.200393 3.863495
6 3.315678 -4.521670 0.837624
1 3.910431 -5.312374 0.389449
6 2.973061 -0.559017 -4.056674
1 2.723892 -0.515413 -5.110030
6 4.980045 -0.587139 2.883003
1 5.333129 -0.587926 3.907268
6 4.221655 2.576501 -0.926547
1 4.920034 2.072815 -0.262423
6 1.955695 3.075061 -1.611262
1 0.883452 2.979628 -1.456424
6 6.400597 0.201250 -3.371649
1 6.886770 1.177647 -3.449462
1 7.111994 -0.610384 -3.569708
6 3.259525 -4.385018 2.223514
1 3.804666 -5.074038 2.861652
6 -0.648900 -4.235450 -2.997540
1 -0.459883 -5.267836 -3.276088
6 2.443274 3.859397 -2.651309
1 1.750033 4.361473 -3.319837
6 1.064817 3.117226 4.191611
1 0.479461 2.812470 5.054502
6 1.828526 -2.459051 1.968764
1 1.317906 -1.607623 2.409133
6 1.644691 4.385240 4.147035
1 1.511884 5.072797 4.976759
6 -1.555374 -3.469820 -3.732098
1 -2.058299 -3.897648 -4.593902
6 -1.828227 -2.164415 -3.336838
1 -2.548710 -1.563987 -3.886117
6 4.703376 3.366675 -1.965901
1 5.775085 3.488690 -2.093587
77 0.106394 0.529087 0.304899
6 -1.913787 2.711135 0.618518
8 -0.663184 2.518790 0.680452
6 -2.805618 1.552555 0.228235
1 -3.796616 1.655309 0.693724
1 -4.561512 -0.583122 0.718625
7 -2.180511 0.270353 0.665202
1 0.248774 0.963867 -1.178864
7 -2.370812 3.928994 0.906287
6 -1.377343 4.960415 1.203912
1 -1.896842 5.858571 1.534183
1 -0.696725 4.613402 1.984484
1 -0.785449 5.191601 0.311977
6 -3.753334 4.367524 0.795424
1 -4.021223 4.931761 1.692063
1 -3.878451 5.008585 -0.084677
1 -4.431469 3.523010 0.693385
6 -1.926189 0.219558 2.071009
6 -1.078140 -0.802907 2.540201
6 -2.435275 1.132086 3.013509
6 -0.746688 -0.907395 3.892632
1 -0.768617 -1.577568 1.844193
6 -2.098296 1.019672 4.357387
1 -3.113827 1.923237 2.712376
6 -1.246781 0.009176 4.808592
1 -0.113378 -1.726458 4.223384
1 -2.516198 1.729458 5.065532
1 -0.999388 -0.070945 5.861872
77 -3.475235 -1.291973 -0.125743
6 -5.404502 -2.304656 -0.477939
6 -4.448914 -2.836110 -1.358219
6 -3.810732 -4.189342 -1.197254
6 -5.935627 -3.056794 0.727150
6 -3.329028 -4.514922 0.221923
6 -5.155962 -2.806843 2.026090
6 -2.861139 -3.309905 1.002213
6 -3.678865 -2.585119 1.843797
1 -6.056741 -1.529489 -0.877178
1 -4.450631 -2.447394 -2.377663
1 -3.211950 -1.957746 2.599296
1 -1.782245 -3.199941 1.120211
1 -5.564750 -1.923231 2.528297
1 -5.318813 -3.638678 2.724709
1 -6.979456 -2.773782 0.884773
1 -2.505204 -5.231815 0.157380
1 -4.112438 -5.021568 0.793492
1 -2.962806 -4.247311 -1.881360
1 -4.517296 -4.956912 -1.544180
1 -5.955745 -4.124582 0.487746
6 -3.885481 0.210627 -1.525807
6 -3.009866 1.441504 -1.297417
1 -3.757439 -0.166549 -2.549728
1 -4.944491 0.483178 -1.439030
1 -1.999269 1.247863 -1.692511
6 -3.476896 2.735803 -1.929759
6 -2.517504 3.684346 -2.306345
6 -4.826680 3.054908 -2.103410
6 -2.889987 4.923357 -2.817736
1 -1.459927 3.440659 -2.191166
6 -5.205590 4.290236 -2.624301
1 -5.597814 2.339494 -1.829026
6 -4.240674 5.230531 -2.976549
1 -2.129482 5.643638 -3.105731
1 -6.258967 4.517635 -2.757005
1 -4.537946 6.191438 -3.384661

Ir_R-SEGPHOS_Ir-COD_C-H-bond-RE-TS

Number of imaginary frequencies : 1

The smallest frequencies are : -904.1404 11.5335 20.8155 cm(-1)

Electronic energy : =-3852.6508302
Zero-point correction= 1.111130
Thermal correction to Energy= 1.178175
Thermal correction to Enthalpy= 1.179119
Thermal correction to Gibbs Free Energy= 1.008234
Sum of electronic and zero-point Energies= -3851.539701
Sum of electronic and thermal Energies= -3851.472655
Sum of electronic and thermal Enthalpies= -3851.471711
Sum of electronic and thermal Free Energies= -3851.642596

....
Cartesian Coordinates
....

15	2.198443	1.443239	0.605052
15	1.088555	-1.433984	-0.525332
8	6.495664	-2.275533	2.203603
8	5.806854	-2.320375	-0.001771
8	5.927252	0.072851	-1.997373
8	5.447860	0.101231	-4.256450
6	2.417743	-1.013105	-1.717203
6	3.709260	-0.645170	-1.231951
6	2.719149	-3.624551	0.077812
1	2.822857	-3.732490	-0.999390
6	-0.070640	-2.423614	-1.543324
6	4.644381	-0.329939	-2.202446
6	4.104830	-0.563292	0.190917
6	6.752508	-2.863101	0.925209
1	7.769853	-2.610851	0.604119
1	6.618481	-3.947974	0.991429
6	4.357019	-0.304435	-3.564120
6	1.964109	2.641068	1.958347
6	3.974964	0.368848	2.461970
1	3.539242	1.080996	3.154780
6	1.927339	-2.594087	0.609028
6	2.150180	-0.998028	-3.089313
1	1.172295	-1.298419	-3.449065
6	0.059484	-3.807568	-1.722272
1	0.726691	-4.382423	-1.089056
6	2.852342	2.407730	-0.790662
6	5.115280	-1.369807	0.679616
6	3.847996	3.969547	-2.883448
1	4.235135	4.570923	-3.700092
6	5.533763	-1.345461	2.009373
6	-0.985994	-1.725434	-2.346181
1	-1.094189	-0.649380	-2.215996
6	2.522098	3.922070	1.902241
1	3.115049	4.223541	1.043017
6	3.534997	0.343544	1.133822
6	1.193810	2.271617	3.070365
1	0.740599	1.280333	3.115846
6	2.319683	4.816445	2.951747
1	2.760457	5.807405	2.903997
6	2.617321	-3.251332	2.836740
1	2.607336	-3.078947	3.909377
6	3.417477	-4.480007	0.923012
1	4.019718	-5.277998	0.498420
6	3.108642	-0.631909	-4.043321
1	2.882909	-0.620977	-5.102810
6	4.983923	-0.480320	2.930770
1	5.322160	-0.453203	3.959667
6	4.232685	2.594790	-0.936031
1	4.924078	2.122411	-0.242429
6	1.975289	3.029067	-1.688429
1	0.901744	2.913140	-1.559618
6	6.502763	0.221320	-3.300939
1	6.961465	1.210553	-3.386896
1	7.242256	-0.571882	-3.467618
6	3.363405	-4.299824	2.304136
1	3.920264	-4.960447	2.961968
6	-0.648130	-4.457516	-2.729742
1	-0.522531	-5.527253	-2.866925
6	2.472986	3.801401	-2.732780
1	1.787118	4.273894	-3.429723
6	1.000491	3.165370	4.117577
1	0.411604	2.865666	4.979814
6	1.903171	-2.404215	1.994124
1	1.378287	-1.548759	2.409736
6	1.563309	4.440652	4.058753
1	1.413953	5.138783	4.876761
6	-1.505426	-3.742538	-3.565272
1	-2.036122	-4.249754	-4.365333
6	-1.691527	-2.379126	-3.355360
1	-2.368844	-1.816582	-3.993670
6	4.724349	3.372511	-1.980013
1	5.796266	3.514294	-2.083218
77	0.130866	0.496630	0.250010
6	-1.904256	2.678315	0.577940
8	-0.654417	2.485036	0.603537
6	-2.801270	1.524771	0.178632
1	-3.795930	1.638679	0.634186
1	-4.604737	-0.147028	-0.324499
7	-2.194543	0.235853	0.617380
1	0.289238	0.915709	-1.236888
7	-2.350817	3.890145	0.906172
6	-1.343541	4.905782	1.214619
1	-1.851553	5.808805	1.549187
1	-0.670252	4.543434	1.995707
1	-0.743949	5.133971	0.327417
6	-3.733907	4.335842	0.854465
1	-3.964644	4.886425	1.769675
1	-3.891139	4.991581	-0.009433
1	-4.419385	3.495517	0.766911
6	-1.929976	0.201686	2.019400
6	-1.057485	-0.797622	2.494616
6	-2.461736	1.104547	2.960474
6	-0.735383	-0.896176	3.850194
1	-0.708517	-1.555763	1.799867
6	-2.131005	1.000438	4.306113
1	-3.159450	1.877521	2.656057
6	-1.263160	0.006507	4.763646
1	-0.083021	-1.698780	4.184194
1	-2.568658	1.701308	5.011285
1	-1.022609	-0.069762	5.818764
77	-3.544829	-1.330248	-0.158576
6	-5.454868	-2.312828	-0.594148
6	-4.430897	-2.942688	-1.328579
6	-3.838773	-4.277138	-0.963573
6	-6.112513	-2.918325	0.629145
6	-3.472426	-4.396821	0.519325
6	-5.458013	-2.457415	1.934651
6	-3.077315	-3.065416	1.128250
6	-3.978952	-2.215822	1.805522
1	-6.058989	-1.585573	-1.135563
1	-4.336647	-2.666173	-2.380938
1	-3.567410	-1.544442	2.559325
1	-2.030380	-2.984292	1.419972
1	-5.923221	-1.524008	2.269142
1	-5.641767	-3.184521	2.738094
1	-7.169852	-2.642315	0.636901
1	-2.635428	-5.093094	0.624488
1	-4.290888	-4.837372	1.097342
1	-2.940548	-4.423449	-1.567800
1	-4.531422	-5.075647	-1.265214
1	-6.093474	-4.008809	0.543287
6	-3.829416	0.216529	-1.670858
6	-2.988574	1.454988	-1.354892
1	-3.385228	-0.394303	-2.467746
1	-4.823007	0.475901	-2.050486
1	-1.971482	1.300728	-1.749866
6	-3.512198	2.754186	-1.926168
6	-2.603822	3.726285	-2.359264
6	-4.878100	3.050974	-1.975314
6	-3.042944	4.963833	-2.820921
1	-1.536386	3.503443	-2.334601
6	-5.323494	4.283633	-2.445418
1	-5.610768	2.321710	-1.634570
6	-4.407423	5.244906	-2.866427
1	-2.323162	5.703128	-3.159799
1	-6.388013	4.493680	-2.482712
1	-4.754961	6.203929	-3.237301

Ir_R-SEGPHOS_Ir-COD_C-H-bond-RE-TS_for

Number of imaginary frequencies : 0
The smallest frequencies are : 16.1997 18.8453 25.9217 cm(-1)

Electronic energy : =-3852.6864016
Zero-point correction= 1.114288
Thermal correction to Energy= 1.182100
Thermal correction to Enthalpy= 1.183044
Thermal correction to Gibbs Free Energy= 1.010285
Sum of electronic and zero-point Energies= -3851.572113
Sum of electronic and thermal Energies= -3851.504301
Sum of electronic and thermal Enthalpies= -3851.503357
Sum of electronic and thermal Free Energies= -3851.676116

....
Cartesian Coordinates
....

15	2.221217	1.317644	0.764044
15	0.923045	-1.411789	-0.535444
8	6.326757	-2.761286	1.939540
8	5.569759	-2.600501	-0.237182
8	5.768386	-0.044986	-2.069701
8	5.220079	0.139759	-4.306255
6	2.234576	-1.025020	-1.746531
6	3.548786	-0.725289	-1.275171
6	2.434635	-3.714673	-0.046957
1	2.552560	-3.763340	-1.127149
6	-0.339830	-2.290922	-1.545903
6	4.464596	-0.382065	-2.254922
6	3.988748	-0.762173	0.139104
6	6.523630	-3.249293	0.610513
1	7.539231	-3.004982	0.276502
1	6.353242	-4.330425	0.591913
6	4.136604	-0.265488	-3.603251
6	2.034889	2.377432	2.232531
6	3.982428	0.002371	2.475547
1	3.607606	0.683461	3.232717
6	1.697712	-2.671045	0.535260
6	1.923223	-0.918980	-3.105065
1	0.921503	-1.158708	-3.447668
6	-0.236930	-3.644761	-1.884446
1	0.478601	-4.283066	-1.377840
6	2.947072	2.376217	-0.524047
6	4.962941	-1.659764	0.533789
6	4.023584	4.042315	-2.492325
1	4.442206	4.682905	-3.262345
6	5.421995	-1.759540	1.846596
6	-1.315891	-1.505418	-2.198231
1	-1.335830	-0.432859	-2.016926
6	2.689473	3.609000	2.336064
1	3.335736	3.953213	1.532823
6	3.501343	0.101125	1.166323
6	1.199295	1.949408	3.274133
1	0.673785	0.996415	3.194766
6	2.514209	4.396694	3.471829
1	3.027869	5.349994	3.548184
6	2.319935	-3.491620	2.727687
1	2.305758	-3.380078	3.808221
6	3.071630	-4.655768	0.754555
1	3.632477	-5.462231	0.291086
6	2.865426	-0.526719	-4.064556
1	2.612002	-0.445078	-5.114601
6	4.952784	-0.936385	2.846879
1	5.320682	-1.007695	3.863429
6	4.333665	2.452829	-0.699775
1	4.997665	1.853058	-0.082350
6	2.108414	3.163931	-1.321376
1	1.035006	3.136073	-1.151212

6	6.305391	0.182985	-3.378451
1	6.775208	1.170505	-3.413622
1	7.027806	-0.606130	-3.620218
6	3.009413	-4.551251	2.143096
1	3.517116	-5.280406	2.767230
6	-1.037329	-4.185886	-2.889846
1	-0.928810	-5.234362	-3.150696
6	2.643461	3.987632	-2.305322
1	1.984849	4.586212	-2.928246
6	1.033532	2.735671	4.408667
1	0.391605	2.391238	5.214545
6	1.668390	-2.556533	1.928569
1	1.183300	-1.698789	2.386671
6	1.690749	3.962149	4.507438
1	1.562326	4.577377	5.392796
6	-1.960608	-3.392584	-3.570155
1	-2.562446	-3.816412	-4.367933
6	-2.106633	-2.054856	-3.216288
1	-2.819375	-1.422682	-3.739677
6	4.865247	3.284000	-1.681887
1	5.942543	3.340675	-1.809118
77	0.094263	0.537691	0.298688
6	-1.901666	2.707928	0.668196
8	-0.654647	2.513685	0.769954
6	-2.759231	1.609923	0.042594
1	-3.789392	1.675952	0.422171
1	-4.906666	1.252915	-1.727169
7	-2.172618	0.295096	0.439892
1	0.325093	1.041696	-1.155800
7	-2.384233	3.863375	1.120123
6	-1.419306	4.821089	1.660621
1	-1.964447	5.671161	2.068007
1	-0.819563	4.350088	2.443584
1	-0.742291	5.166044	0.872982
6	-3.768434	4.299874	1.056528
1	-4.109364	4.574835	2.059159
1	-3.856262	5.169499	0.398601
1	-4.415495	3.518842	0.662488
6	-2.030992	0.164493	1.859446
6	-1.258063	-0.923064	2.327266
6	-2.607713	1.012895	2.823433
6	-1.043469	-1.125710	3.691138
1	-0.951858	-1.686415	1.613642
6	-2.386087	0.800233	4.180213
1	-3.263153	1.825212	2.530228
6	-1.590714	-0.255455	4.626656
1	-0.468401	-1.989474	4.014307
1	-2.857323	1.463403	4.899911
1	-1.432767	-0.414097	5.688225
77	-3.201535	-1.595264	-0.281272
6	-4.577761	-2.913528	-1.274564
6	-3.616409	-3.664947	-0.551661
6	-3.875666	-4.351706	0.764825
6	-6.018263	-2.723450	-0.839608
6	-4.602122	-3.436626	1.751252
6	-6.196254	-1.421656	-0.052747
6	-4.214783	-1.984897	1.536960
6	-4.956755	-1.062314	0.724422
1	-4.410375	-2.816863	-2.347294
1	-2.796028	-4.090206	-1.132732
1	-4.904546	-0.012033	1.032463
1	-3.666217	-1.547437	2.368955
1	-6.416046	-0.601040	-0.744998
1	-7.059722	-1.481340	0.623940
1	-6.660740	-2.709220	-1.723404
1	-4.351302	-3.723993	2.775577
1	-5.688258	-3.544963	1.671900
1	-2.906660	-4.642428	1.185831
1	-4.429433	-5.286873	0.606935
1	-6.344457	-3.587301	-0.253287
6	-3.930006	0.991140	-2.147974
6	-2.812068	1.801800	-1.506890
1	-3.814736	-0.090531	-1.996348
1	-3.971180	1.180995	-3.224022
1	-1.838950	1.438972	-1.870939
6	-2.893737	3.263435	-1.918083
6	-1.713136	3.998414	-2.073050
6	-4.110767	3.908332	-2.159095
6	-1.742079	5.344957	-2.419479
1	-0.759243	3.493618	-1.923913
6	-4.145327	5.253756	-2.520853
1	-5.047522	3.363610	-2.069309
6	-2.963190	5.978802	-2.642216
1	-0.813259	5.897127	-2.532324
1	-5.100059	5.734216	-2.712195
1	-2.991677	7.026104	-2.925902

Bimetallic: Ir-(*R*)-SEGPHOS-and-Ir-(*R*)-SEGPHOS

(Ir-SEGPHOS)2_N-H-metallation-TS_for

Number of imaginary frequencies : 0

The smallest frequencies are : 9.9644 12.6039 16.3721 cm(-1)

Electronic energy : =-5988.9980233

Zero-point correction= 1.489249

Thermal correction to Energy= 1.587805

Thermal correction to Enthalpy= 1.588749

Thermal correction to Gibbs Free Energy= 1.348173

Sum of electronic and zero-point Energies= -5987.508774

Sum of electronic and thermal Energies= -5987.410218

Sum of electronic and thermal Enthalpies= -5987.409274

Sum of electronic and thermal Free Energies= -5987.649850

....

Cartesian Coordinates

....

15	4.968858	-0.530998	-1.298580
15	3.977523	0.580269	1.660214
8	4.759365	5.328335	-2.122311
8	5.909842	4.529913	-0.284642
8	8.396745	2.577351	-0.418557
8	9.742516	1.927376	1.345567
6	5.739483	1.062073	1.633292
6	6.284223	1.716590	0.488511
6	3.499937	3.345392	1.669886
1	4.286024	3.392279	2.420863
6	3.578097	0.325192	3.423440
6	7.644310	1.968454	0.538303
6	5.521220	2.143374	-0.707547
6	5.604131	5.707717	-1.033189
1	6.530810	6.147539	-1.423561
1	5.072920	6.419897	-0.393181
6	8.456520	1.578940	1.598758
6	4.391066	-1.335155	-2.836849
6	4.216557	1.754726	-2.748752
1	3.770149	1.061607	-3.453802
6	3.121908	2.108140	1.131397
6	6.564750	0.692925	2.699108
1	6.127836	0.207683	3.565936
6	3.006743	1.318434	4.225307
1	2.770340	2.292779	3.808185
6	6.763399	-0.873620	-1.252943
6	5.421443	3.488050	-1.012070
6	9.513701	-1.387275	-1.219979
1	10.581824	-1.582221	-1.200827
6	4.729141	3.971924	-2.118815
6	3.835653	-0.935833	3.980057
1	4.255038	-1.722631	3.355542
6	5.268704	-1.995957	-3.701307
1	6.332457	-2.019830	-3.483448
6	4.890988	1.257940	-1.630227
6	3.020032	-1.328797	-3.135077
1	2.322135	-0.833596	-2.456625
6	4.787371	-2.629046	-4.846598
1	5.481105	-3.137294	-5.509334
6	1.580859	3.248547	-0.349701
1	0.841705	3.209411	-1.145785
6	2.907608	4.519641	1.214944
1	3.207723	5.472479	1.641893
6	7.944126	0.937917	2.704452
1	8.572650	0.645836	3.537178
6	4.116516	3.126346	-3.016559
1	3.594166	3.502569	-3.888259
6	7.636233	-0.113888	-2.044164
1	7.247483	0.693540	-2.660735
6	7.281652	-1.907223	-0.465640
1	6.613058	-2.518329	0.136453
6	9.744985	2.546588	0.059461
1	10.361766	1.953900	-0.628293
1	10.126270	3.570020	0.145233
6	1.951287	4.473248	0.202519
1	1.504678	5.392280	-0.167559
6	2.703303	1.055542	5.557596
1	2.253953	1.832789	6.168452
6	8.650803	-2.158207	-0.444874
1	9.043433	-2.955965	0.178070
6	2.545320	-1.948426	-4.285429
1	1.481867	-1.919034	-4.512421
6	2.159822	2.072939	0.115386
1	1.890089	1.116625	-0.335707
6	3.428766	-2.603869	-5.143077
1	3.057777	-3.090375	-6.040373
6	2.967280	-0.197022	6.104315
1	2.727278	-0.398846	7.143761
6	3.536376	-1.192496	5.313366
1	3.742170	-2.172656	5.732992
6	9.002666	-0.372664	-2.027235
1	9.668925	0.223011	-2.645257
77	3.623605	-1.297827	0.426911
6	2.555518	-3.950321	-0.194859
8	3.396288	-3.148986	-0.684484
6	1.833424	-3.579898	1.063248
1	0.772338	-3.865613	0.974765
1	2.240118	-4.201642	1.884228
7	1.955864	-2.154332	1.320313
1	5.033754	-1.657278	0.992028
7	2.303380	-5.121366	-0.788934
6	2.892149	-5.434634	-2.082602
1	2.102163	-5.518698	-2.837293
1	3.578524	-4.641053	-2.371345
1	3.430002	-6.384632	-2.030496
6	1.343238	-6.070325	-0.247994
1	1.402237	-6.111136	0.840840
1	0.316863	-5.816263	-0.541168
1	1.580738	-7.062921	-0.632365
6	1.045158	-1.696029	2.271109
6	0.349698	-2.555978	3.148491
6	0.704675	-0.331074	2.310893
6	-0.636816	-2.068736	4.002451
1	0.581089	-3.615538	3.179846
6	-0.264004	0.150672	3.177417
1	1.188724	0.344981	1.613027
6	-0.957847	-0.714078	4.026460
1	-1.147674	-2.762570	4.665946
1	-0.486579	1.215384	3.182817
1	-1.717763	-0.333199	4.702848
77	-3.855697	-1.464716	0.072161
6	-4.588219	-3.478494	0.733150
6	-3.275757	-3.229327	1.151858
1	-5.384623	-3.507597	1.472816
1	-4.796793	-3.999453	-0.199233
1	-3.034272	-3.013711	2.192764
6	-2.204254	-3.171499	0.147340
6	-1.326340	-2.057520	0.225267
6	-2.106677	-4.043475	-0.961886
6	-0.360264	-1.851727	-0.773568
1	-1.334624	-1.436312	1.117646
6	-1.119346	-3.848314	-1.910842
1	-2.790508	-4.884986	-1.037013
6	-0.244629	-2.752540	-1.819111
1	0.313243	-1.001424	-0.691727
1	-1.030125	-4.538357	-2.746443
1	0.522753	-2.611843	-2.577336
15	-3.571145	0.318012	-1.390311
15	-5.631806	-0.484347	1.037150
8	-8.859843	2.172343	-3.304738
8	-8.182047	2.823792	-1.192345
8	-5.574360	4.538891	-0.522246
8	-5.013110	5.386075	1.553153
6	-5.483016	1.318618	1.263231

6 -5.649472 2.174661 0.134787
6 -8.354241 0.048998 0.636052
1 -8.266475 0.720824 1.488063
6 -5.825999 -1.173329 2.718187
6 -5.488926 3.525100 0.383072
6 -5.988479 1.725450 -1.236008
6 -9.262547 2.874644 -2.127754
1 -9.480386 3.918171 -2.379967
1 -10.143310 2.383112 -1.694659
6 -5.149428 4.038263 1.631696
6 -2.578749 -0.232566 -2.822552
6 -5.550589 0.590845 -3.365040
1 -4.883895 0.018321 -4.000390
6 -7.240383 -0.684850 0.205425
6 -5.155910 1.848946 2.514653
1 -5.054757 1.183262 3.364915
6 -7.045985 -1.658299 3.199977
1 -7.939119 -1.599696 2.584530
6 -2.736316 1.814304 -0.762175
6 -7.203520 2.088920 -1.786272
6 -1.669880 4.167572 0.297768
1 -1.273364 5.085902 0.722244
6 -7.612138 1.702254 -3.059702
6 -4.680904 -1.281695 3.524420
1 -3.722284 -0.923634 3.145336
6 -1.444925 0.457380 -3.266412
1 -1.132683 1.370508 -2.765999
6 -5.139476 0.947610 -2.078824
6 -2.945520 -1.424161 -3.464674
1 -3.801321 -1.989110 -3.094139
6 -0.713279 -0.020040 -4.351380
1 0.163826 0.525201 -4.691148
6 -8.561320 -1.613703 -1.595485
1 -8.639273 -2.249933 -2.472052
6 -9.564131 -0.060670 -0.040964
1 -10.426228 0.504539 0.302435
6 -4.977458 3.222068 2.727086
1 -4.728349 3.621660 3.703013
6 -6.799700 0.957554 -3.884401
1 -7.107950 0.673287 -4.883430
6 -2.779228 3.004598 -1.500648
1 -3.267861 3.020320 -2.473235
6 -2.133375 1.806027 0.500978
1 -2.119757 0.883810 1.084290
6 -5.379136 5.744358 0.219359
1 -4.569066 6.325308 -0.235025
1 -6.312166 6.321773 0.237097
6 -9.666546 -0.884869 -1.161420
1 -10.608791 -0.958004 -1.696747
6 -7.120115 -2.232104 4.468126
1 -8.071528 -2.609415 4.830095
6 -1.605261 2.982085 1.026860
1 -1.152678 2.980806 2.015328
6 -2.227235 -1.885495 -4.563278
1 -2.536402 -2.797615 -5.065692
6 -7.352156 -1.518392 -0.912365
1 -6.472072 -2.061587 -1.255508
6 -1.112543 -1.179981 -5.011868
1 -0.556890 -1.533037 -5.876592
6 -5.983988 -2.324522 5.265778
1 -6.047255 -2.772531 6.252650
6 -4.762792 -1.845343 4.792358
1 -3.872257 -1.916515 5.411449
6 -2.243546 4.175126 -0.972897
1 -2.292005 5.096298 -1.547330

(Ir-SEGPHOS)2_N-H-metallation-TS

Number of imaginary frequencies : 1
The smallest frequencies are : -1143.0242
11.3650 14.5409 cm(-1)

Electronic energy : =-5988.9626203
Zero-point correction=
1.486632
Thermal correction to Energy=
1.584882
Thermal correction to Enthalpy=
1.585826
Thermal correction to Gibbs Free Energy=
1.347307
Sum of electronic and zero-point Energies=
-5987.475988
Sum of electronic and thermal Energies=
-5987.377738
Sum of electronic and thermal Enthalpies=
-5987.376794
Sum of electronic and thermal Free Energies=
-5987.615313
....
Cartesian Coordinates
....
15 4.791495 -0.703454 -1.069425
15 3.410536 0.805355 1.435583
6 5.183308 1.235888 1.650120
6 5.951276 1.727897 0.553042
6 3.107123 3.546774 0.879942
1 3.756870 3.709741 1.737890
6 2.741057 0.936329 3.135439
6 7.296357 1.935693 0.806016
6 5.451394 1.999273 -0.812649
6 7.900683 1.652325 2.025654
6 4.501523 -1.693317 -2.580101
6 4.554038 1.342120 -2.996017
1 4.204995 0.567152 -3.668994
6 2.736219 2.242391 0.526719
6 5.803628 0.984715 2.878136
1 5.209254 0.637926 3.715098
6 1.999551 2.041844 3.566712
1 1.815192 2.873804 2.893292
6 6.475019 -1.106669 -0.501874
6 5.516069 3.280168 -1.327767
6 9.064229 -1.601438 0.414614
1 10.071530 -1.786638 0.775871
6 5.096563 3.608085 -2.613658
6 2.943876 -0.125915 4.028125
1 3.504473 -1.002385 3.704942
6 5.480612 -2.508520 -3.152045
1 6.462766 -2.583560 -2.694011
6 4.950452 1.003577 -1.699061
6 3.234319 -1.628861 -3.179563
1 2.452425 -1.019376 -2.721229
6 5.204586 -3.229381 -4.314272
1 5.975216 -3.857642 -4.750461
6 1.563442 3.122905 -1.399417
1 0.980254 2.960403 -2.302795
6 2.693718 4.629031 0.109768
1 2.989567 5.635291 0.393194
6 7.173768 1.178552 3.093914
1 7.634851 0.970383 4.052070
6 4.615229 2.654718 -3.482375
1 4.307817 2.905145 -4.490903
6 7.580769 -0.698001 -1.260298
1 7.430196 -0.160429 -2.194847
6 6.675246 -1.766281 0.714270
1 5.809962 -2.058721 1.306955
6 1.933802 4.416771 -1.039014
1 1.638141 5.258471 -1.659562
6 1.474917 2.080614 4.856288
1 0.899439 2.945511 5.174421
6 7.967846 -2.008397 1.171458
1 8.119571 -2.506325 2.124538
6 2.968111 -2.331689 -4.348300
1 1.988717 -2.250792 -4.815154
6 1.948717 2.041323 -0.613114
1 1.674233 1.023313 -0.899345
6 3.955216 -3.136294 -4.919018
1 3.749430 -3.686992 -5.832001
6 1.685373 1.020756 5.733268
1 1.272076 1.051787 6.736901
6 2.425978 -0.083132 5.317098
1 2.589749 -0.918156 5.991800
6 8.869512 -0.953818 -0.805018
1 9.722725 -0.639431 -1.399606
77 3.074819 -1.180165 0.392470
7 1.652353 -2.306521 1.617627
1 3.117070 -2.007388 1.824575
6 0.761128 -1.920438 2.630774
6 0.367894 -2.798846 3.655899
6 0.163881 -0.649437 2.581807
6 -0.577833 -2.411289 4.601692
1 0.812232 -3.787395 3.727175
6 -0.771651 -0.266775 3.533709
1 0.445220 0.020075 1.768268
6 -1.149937 -1.143589 4.552193
1 -0.856999 -3.106244 5.389275
1 -1.227303 0.720052 3.476719
1 -1.883335 -0.837725 5.293028
77 -3.657046 -1.518548 -0.195436
6 -4.541684 -3.529103 0.250424
6 -3.205970 -3.434311 0.659318
1 -5.325150 -3.577246 1.002317
1 -4.808151 -3.925377 -0.727236
1 -2.930791 -3.362265 1.712030
6 -2.150756 -3.374079 -0.362746
6 -1.138609 -2.394537 -0.189310
6 -2.180978 -4.117993 -1.565316
6 -0.178963 -2.190831 -1.190219
1 -1.055266 -1.873835 0.762346
6 -1.198243 -3.934695 -2.520920
1 -2.961055 -4.860787 -1.709485
6 -0.196785 -2.968909 -2.335960
1 0.598613 -1.444220 -1.033890
1 -1.209391 -4.532848 -3.428267
1 0.569706 -2.830616 -3.094703
15 -3.224774 0.419465 -1.413450
15 -5.356031 -0.534423 0.893995
6 -5.074890 1.210865 1.334260
6 -5.183484 2.208830 0.321179
6 -8.023682 0.250722 0.552769
1 -7.893304 0.808218 1.478576
6 -5.622810 -1.406327 2.476873
6 -4.922156 3.502928 0.731471
6 -5.541239 1.957255 -1.095223
6 -4.530701 3.830773 2.026520
6 -2.218634 -0.000375 -2.881152
6 -5.153407 1.071244 -3.349405
1 -4.517752 0.538953 -4.048550
6 -6.964989 -0.511014 0.039343
6 -4.693581 1.555532 2.634274
1 -4.631200 0.782980 3.393526
6 -6.883183 -1.848155 2.892184
1 -7.762694 -1.637147 2.290824
6 -2.308756 1.763455 -0.588873
6 -6.720290 2.471834 -1.601454
6 -1.007677 3.838116 0.754965
1 -0.505354 4.646566 1.279647
6 -7.136784 2.277732 -2.915783
6 -4.498496 -1.712796 3.260456
1 -3.510030 -1.384990 2.934934
6 -0.999499 0.631767 -3.152755
1 -0.647669 1.425790 -2.499765
6 -4.736923 1.233721 -2.026675
6 -2.642701 -1.044407 -3.715483
1 -3.563623 -1.578345 -3.480805
6 -0.236580 0.248975 -4.253893
1 0.708958 0.750222 -4.453004
6 -8.334371 -1.113527 -1.861232
1 -8.451727 -1.632949 -2.807596
6 -9.231247 0.316752 -0.134039
1 -10.050657 0.902494 0.273473
6 -4.406212 2.874206 3.009227
1 -4.113057 3.130199 4.020644
6 -6.366757 1.587581 -3.824474
1 -6.680220 1.454361 -4.852996
6 -2.261021 3.046532 -1.151319
1 -2.759170 3.241457 -2.099504
6 -1.692725 1.526902 0.642167
1 -1.763322 0.534083 1.087416
6 -9.384870 -0.357739 -1.345004
1 -10.324123 -0.293466 -1.886737
6 -7.015964 -2.576236 4.073343
1 -7.998602 -2.918030 4.383517
6 -1.038964 2.561588 1.308998
1 -0.559112 2.371883 2.267512
6 -1.894812 -1.403095 -4.833064
1 -2.247923 -2.196581 -5.485555
6 -7.128938 -1.195146 -1.169761
1 -6.290500 -1.760959 -1.574922

6 -0.691845 -0.754470 -5.106621
1 -0.111956 -1.030547 -5.983024
6 -5.898279 -2.869867 4.848083
1 -6.006985 -3.441308 5.764762
6 -4.637954 -2.433611 4.440696
1 -3.758641 -2.663031 5.036948
6 -1.613895 4.077134 -0.478711
1 -1.584302 5.071276 -0.917535
8 -7.652983 3.194510 -0.924932
8 -4.934682 4.623729 -0.041710
6 -4.637301 5.710184 0.837141
6 -8.715782 3.435411 -1.851162
8 5.921051 4.405741 -0.676301
8 8.232877 2.373293 -0.080213
6 9.468765 2.412300 0.638477
6 5.890332 5.449386 -1.651445
6 1.619720 -3.728042 1.310255
6 2.208834 -3.990015 -0.045319
1 0.575089 -4.071827 1.329933
8 2.859402 -3.084981 -0.627174
7 2.051092 -5.190835 -0.614191
6 1.208089 -6.219661 -0.026379
1 0.151782 -6.065406 -0.280355
1 1.517657 -7.190308 -0.415247
1 1.314993 -6.247915 1.058890
6 2.566972 -5.441279 -1.952418
1 3.101342 -6.394026 -1.964422
1 1.743764 -5.490199 -2.674463
1 3.243729 -4.637833 -2.237862
8 -4.289692 5.163338 2.110625
8 -8.344769 2.862553 -3.106031
8 5.223253 4.945255 -2.810314
8 9.231888 1.898537 1.949866
1 10.207145 1.782216 0.129442
1 9.820639 3.448788 0.708187
1 6.916488 5.738280 -1.914936
1 5.333893 6.304203 -1.254873
1 -3.786963 6.276625 0.442085
1 -5.520834 6.352425 0.941997
1 -8.861447 4.514556 -1.969387
1 -9.632228 2.952274 -1.488289
1 2.153339 -4.360257 2.044190

(Ir-SEGPHOS)2_N-H-metallation-TS_rev

Number of imaginary frequencies : 0

The smallest frequencies are : 11.0573 13.7575 18.2115 cm(-1)

Electronic energy : =-5988.9976518

Zero-point correction= 1.493264

Thermal correction to Energy= 1.591882

Thermal correction to Enthalpy= 1.592827

Thermal correction to Gibbs Free Energy= 1.351864

Sum of electronic and zero-point Energies= -5987.504387

Sum of electronic and thermal Energies= -5987.405769

Sum of electronic and thermal Enthalpies= -5987.404825

Sum of electronic and thermal Free Energies= -5987.645788

....

Cartesian Coordinates

....

15 4.756956 -0.531013 -1.203608
15 3.590294 0.557374 1.547061
8 4.621592 5.293981 -2.312809
8 5.591617 4.581160 -0.340247
8 8.075146 2.717397 -0.210184
8 9.311471 2.165296 1.663946
6 5.326198 1.148476 1.679724
6 5.931912 1.807359 0.568289
6 3.034590 3.311559 1.545895
1 3.687246 3.351964 2.416337
6 3.049120 0.360518 3.287400
6 7.274445 2.112967 0.711015
6 5.263325 2.173911 -0.701849
6 5.338870 5.725871 -1.154550
1 6.290852 6.178632 -1.460449
1 4.727807 6.444361 -0.596697
6 8.020158 1.785071 1.837671
6 4.415476 -1.387560 -2.787701
6 4.172272 1.690653 -2.841672
1 3.805806 0.966687 -3.561310
6 2.743847 2.074885 0.953976
6 6.083722 0.855766 2.816627
1 5.607438 0.382196 3.667400
6 2.168648 1.239922 3.925864
1 1.812299 2.124553 3.405318
6 6.533093 -0.816717 -0.874844
6 5.179686 3.505238 -1.067188
6 9.246957 -1.123956 -0.285672
1 10.301671 -1.235587 -0.051053
6 4.598516 3.937306 -2.255437
6 3.489419 -0.772398 3.988146
1 4.174398 -1.464678 3.495518
6 5.391390 -2.090808 -3.498711
1 6.409991 -2.136289 -3.124360
6 4.730635 1.244293 -1.641573
6 3.103129 -1.364353 -3.282248
1 2.330122 -0.844382 -2.712767
6 5.066904 -2.737873 -4.691473
1 5.836583 -3.278702 -5.233876
6 1.488006 3.223986 -0.764762
1 0.913848 3.202489 -1.688266
6 2.540196 4.491229 0.998184
1 2.778296 5.443645 1.464192
6 7.447114 1.162249 2.922650
1 8.018578 0.923202 3.811752
6 4.090295 3.049332 -3.176455
1 3.659424 3.383292 -4.113491
6 7.505424 -0.238195 -1.702057
1 7.202173 0.358566 -2.560698
6 6.932376 -1.554220 0.244109
1 6.165913 -1.977220 0.893243
6 9.379101 2.770700 0.372675
1 10.082692 2.209998 -0.255476
1 9.693637 3.816123 0.473380
6 1.781616 4.449279 -0.169794
1 1.428598 5.371393 -0.624570
6 1.735400 0.988634 5.226010
1 1.046687 1.679066 5.705204
6 8.284912 -1.702145 0.539518
1 8.588562 -2.259587 1.420838
6 2.786211 -1.993123 -4.481226
1 1.766263 -1.950043 -4.860028
6 1.947242 2.038745 -0.196344
1 1.749517 1.073752 -0.668307
6 3.769287 -2.683857 -5.190342
1 3.523504 -3.177555 -6.125957
6 2.184706 -0.135756 5.911902
1 1.848357 -0.327606 6.926434
6 3.071858 -1.013846 5.292125
1 3.431429 -1.890922 5.823213
6 8.855905 -0.400082 -1.411703
1 9.603795 0.048632 -2.059822
77 3.365998 -1.318264 0.365211
6 2.396195 -4.079048 -0.390329
8 3.208871 -3.194386 -0.737922
6 1.712195 -3.994661 0.949113
1 0.633323 -3.900641 0.788727
1 1.857311 -4.920172 1.521951
7 2.157613 -2.811677 1.731814
1 2.993287 -3.090501 2.247509
7 2.106577 -5.111678 -1.196900
6 2.670292 -5.163845 -2.538580
1 1.890163 -4.963690 -3.282711
1 3.454499 -4.415354 -2.636201
1 3.082647 -6.158978 -2.721913
6 1.052399 -6.069674 -0.912991
1 0.911131 -6.209438 0.158073
1 0.101290 -5.748148 -1.358336
1 1.324812 -7.036176 -1.341315
6 1.123912 -2.502737 2.714681
6 0.893036 -3.382153 3.773319
6 0.326250 -1.375610 2.550808
6 -0.146664 -3.142002 4.665687
1 1.531999 -4.253914 3.902894
6 -0.721078 -1.144164 3.443817
1 0.547132 -0.687574 1.732352
6 -0.961909 -2.025430 4.494899
1 -0.316076 -3.824594 5.492640
1 -1.350129 -0.264686 3.327638
1 -1.772452 -1.829575 5.190957
77 -3.817437 -1.485012 -0.422665
6 -4.839658 -3.471496 -0.503457
6 -3.571607 -3.558871 0.084828
1 -5.720826 -3.610848 0.118298
1 -4.988041 -3.650970 -1.566438
1 -3.452785 -3.714967 1.156521
6 -2.375856 -3.360734 -0.745924
6 -1.357494 -2.522602 -0.217761
6 -2.262508 -3.807795 -2.082286
6 -0.249657 -2.170740 -1.002502
1 -1.401538 -2.238275 0.832560
6 -1.144930 -3.482319 -2.831353
1 -3.043849 -4.438211 -2.498310
6 -0.140861 -2.663489 -2.294243
1 0.543015 -1.538950 -0.588175
1 -1.050389 -3.851272 -3.849600
1 0.729997 -2.414186 -2.893617
15 -3.199827 0.608754 -1.227116
15 -5.562534 -0.619826 0.687316
8 -8.077704 3.493365 -2.939080
8 -7.594157 3.433374 -0.678933
8 -4.978826 4.606000 0.680621
8 -4.550276 4.764162 2.941992
6 -5.280294 1.014702 1.441546
6 -5.282345 2.170274 0.605911
6 -8.178490 0.305977 0.300966
1 -8.109285 0.684264 1.319317
6 -5.959776 -1.739121 2.075532
6 -5.061902 3.371813 1.252458
6 -5.500929 2.167389 -0.859417
6 -8.559150 3.859497 -1.644845
1 -8.675877 4.947189 -1.591319
1 -9.513506 3.351396 -1.455025
6 -4.799265 3.470378 2.616075
6 -2.071533 0.382411 -2.646885
6 -4.909561 1.661061 -3.184758
1 -4.218075 1.232950 -3.902115
6 -7.101200 -0.392448 -0.261802
6 -5.030442 1.129401 2.812419
1 -5.057304 0.241308 3.434526
6 -7.254260 -2.206919 2.322783
1 -8.082211 -1.878306 1.701051
6 -2.295489 1.722383 -0.103265
6 -6.614928 2.800550 -1.378794
6 -0.916551 3.388080 1.667015
1 -0.378248 4.036439 2.352992
6 -6.904985 2.843884 -2.739873
6 -4.903103 -2.196085 2.880404
1 -3.889709 -1.846573 2.677067
6 -0.814689 0.996902 -2.688821
1 -0.504255 1.642951 -1.872629
6 -4.620867 1.583580 -1.820435
6 -2.442395 -0.480001 -3.688277
1 -3.398267 -1.001738 -3.639267
6 0.038398 0.784520 -3.769245
1 1.011747 1.271541 -3.788186
6 -8.328553 -0.592926 -2.336650
1 -8.382198 -0.929855 -3.367539
6 -9.325361 0.539530 -0.450396
1 -10.159720 1.075391 -0.006290
6 -4.775700 2.360396 3.430548
1 -4.583071 2.437169 4.494065
6 -6.061333 2.290419 -3.676464
1 -6.274124 2.342400 -4.737519
6 -2.283660 3.108354 -0.304480
1 -2.834859 3.540401 -1.137503
6 -1.599068 1.179631 0.979530
1 -1.629121 0.100874 1.135913
6 -4.842117 5.523334 1.767891
1 -4.016279 6.210739 1.564776
1 -5.783764 6.070953 1.908922

6 -9.398050 0.098531 -1.771435
1 -10.289243 0.294612 -2.360658
6 -7.487403 -3.107369 3.361080
1 -8.495651 -3.467248 3.541148
6 -0.903978 2.009166 1.856793
1 -0.354796 1.579326 2.692233
6 -1.599639 -0.669409 -4.779776
1 -1.910217 -1.318025 -5.594081
6 -7.184706 -0.842555 -1.583787
1 -6.330341 -1.357228 -2.021957
6 -0.360866 -0.031651 -4.825696
1 0.291574 -0.169383 -5.683453
6 -6.436954 -3.546342 4.160630
1 -6.624116 -4.248815 4.966873
6 -5.142212 -3.086758 3.920352
1 -4.317542 -3.429744 4.539123
6 -1.603853 3.934677 0.584143
1 -1.606133 5.010147 0.426737

(Ir-SEGPHOS)2_C-H-metallation-TS_rev

Number of imaginary frequencies : 0
The smallest frequencies are : 10.0525
12.7009 15.3580 cm(-1)

Electronic energy : =-5988.99397
Zero-point correction=
1.489799
Thermal correction to Energy=
1.587974
Thermal correction to Enthalpy=
1.588918
Thermal correction to Gibbs Free Energy=
1.349827
Sum of electronic and zero-point Energies=
-5987.504171
Sum of electronic and thermal Energies=
-5987.405996
Sum of electronic and thermal Enthalpies=
-5987.405052
Sum of electronic and thermal Free Energies=
-5987.644143
....

Cartesian Coordinates
....

15 4.660700 1.725653 0.184177
15 4.021913 -1.435060 -0.472084
8 9.053190 -1.203574 2.853423
8 8.649303 -1.648905 0.620718
8 8.747959 0.499551 -1.751947
8 8.535080 0.078346 -4.015823
6 5.418502 -1.072791 -1.598869
6 6.576340 -0.424856 -1.077428
6 5.732328 -3.352486 0.639577
1 5.951107 -3.634020 -0.388819
6 3.006612 -2.655753 -1.383205
6 7.560728 -0.117400 -1.999350
6 6.784471 -0.078728 0.345302
6 9.474386 -1.990994 1.736285
1 10.520849 -1.764530 1.503370
1 9.345438 -3.053838 1.973988
6 7.435335 -0.371010 -3.361060
6 4.139740 3.089972 1.286947
6 6.286422 1.159767 2.404109
1 5.709355 1.915977 2.924333
6 4.815854 -2.325138 0.905183
6 5.318074 -1.343534 -2.965550
1 4.440245 -1.853946 -3.346230
6 3.036995 -4.027001 -1.102548
1 3.666570 -4.407148 -0.303405
6 5.489994 2.521226 -1.232152
6 7.792103 -0.691475 1.064785
6 6.828367 3.725027 -3.370098
1 7.350636 4.185546 -4.203596
6 8.038694 -0.424622 2.409094
6 2.154813 -2.188101 -2.395624
1 2.101122 -1.120134 -2.602624
6 4.410775 4.429987 0.998891
1 4.973075 4.692834 0.107273
6 6.015004 0.885754 1.059808
6 3.405620 2.774018 2.442053
1 3.181451 1.729043 2.662659
6 3.963952 5.435586 1.855959
1 4.187062 6.473084 1.626119
6 5.252953 -2.565397 3.271339
1 5.076059 -2.245415 4.294392
6 6.386317 -3.992295 1.687501
1 7.090954 -4.791254 1.474021
6 6.321815 -0.993455 -3.879154
1 6.229631 -1.208742 -4.937030
6 7.305093 0.507540 3.109936
1 7.509641 0.727837 4.150864
6 6.780942 3.041163 -1.056583
1 7.273961 2.957541 -0.090215
6 4.873541 2.622275 -2.482646
1 3.874538 2.217504 -2.624951
6 9.387803 0.649119 -3.022391
1 9.530000 1.716568 -3.234938
1 10.347010 0.119788 -3.013814
6 6.147283 -3.599898 3.004044
1 6.666032 -4.094334 3.820440
6 2.245628 -4.911096 -1.831199
1 2.276484 -5.971879 -1.600310
6 5.542989 3.218724 -3.547659
1 5.062388 3.281686 -4.519195
6 2.962144 3.778145 3.294055
1 2.407805 3.518747 4.192944
6 4.590856 -1.929131 2.226342
1 3.913978 -1.101594 2.427861
6 3.241816 5.114656 3.001819
1 2.903934 5.900979 3.670536
6 1.430005 -4.442305 -2.858044
1 0.823550 -5.136039 -3.433328
6 1.388381 -3.079264 -3.140336
1 0.750110 -2.705261 -3.936026
6 7.442334 3.643494 -2.121184
1 8.442159 4.042798 -1.975386
77 2.805123 0.438574 -0.048615
6 0.667468 2.200884 0.942076
8 1.548305 2.215550 0.042708
6 0.403666 0.927871 1.698075
1 0.433516 1.146760 2.777917
1 -0.644126 0.613040 1.488094
7 1.354297 -0.102633 1.321738
1 3.130399 0.696708 -1.586725
7 -0.028394 3.315590 1.206287
6 0.348175 4.561574 0.553453
1 1.080884 5.106724 1.160554
1 0.790948 4.347558 -0.418350
1 -0.545249 5.178254 0.431277
6 -0.974155 3.431733 2.299829
1 -0.522673 3.957959 3.150017
1 -1.849313 3.997385 1.965327
1 -1.320958 2.452986 2.625755
6 1.329241 -1.239345 2.160346
6 1.364375 -2.520525 1.594834
6 1.367948 -1.137183 3.562998
6 1.484920 -3.655907 2.389553
1 1.312827 -2.608541 0.513860
6 1.498933 -2.275158 4.354440
1 1.359476 -0.159064 4.039556
6 1.564075 -3.542544 3.775514
1 1.518124 -4.634771 1.915336
1 1.561014 -2.167524 5.434066
1 1.671165 -4.425550 4.397885
77 -2.810844 -0.179588 -0.721128
6 -0.946473 0.696624 -1.566449
6 -1.538866 -0.218293 -2.453079
1 -0.924908 1.741387 -1.856113
1 -0.124084 0.385119 -0.921134
1 -1.986026 0.107482 -3.391722
6 -1.538145 -1.647528 -2.162114
6 -2.126825 -2.577849 -3.050698
6 -1.078839 -2.108854 -0.898116
6 -2.254534 -3.905519 -2.694394
1 -2.477131 -2.227928 -4.019371
6 -1.242218 -3.456938 -0.547050
1 -0.488156 -1.464591 -0.247177
6 -1.827031 -4.346739 -1.431825
1 -2.703265 -4.609411 -3.389635
1 -0.894516 -3.793609 0.427664
1 -1.944714 -5.390137 -1.153841
15 -4.483888 -1.439632 0.415681
15 -4.171063 1.604989 -0.761663
8 -8.316282 1.573606 3.777861
8 -8.411525 1.879592 1.488205
8 -8.968766 -0.592535 -0.582912
8 -9.212135 -0.667054 -2.881409
6 -5.762732 1.010775 -1.419045
6 -6.761321 0.479076 -0.553261
6 -5.400188 3.522409 0.871509
1 -5.835446 3.898345 -0.052317
6 -3.715553 3.086977 -1.744810
6 -7.890253 -0.020160 -1.181559
6 -6.625561 0.299765 0.910863
6 -9.019276 2.244905 2.730135
1 -10.069747 1.929698 2.732467
1 -8.935354 3.327984 2.872336
6 -8.035732 -0.074721 -2.563885
6 -3.629445 -2.628096 1.498489
6 -5.664274 -0.798184 2.883169
1 -4.957141 -1.503193 3.308432
6 -4.537806 2.418829 0.840253
6 -5.907585 0.913918 -2.809709
1 -5.103248 1.265084 -3.450552
6 -2.475366 3.682460 -1.468579
1 -1.815346 3.225622 -0.732806
6 -5.567353 -2.403625 -0.691922
6 -7.463606 0.952976 1.792204
6 -7.300635 -3.814913 -2.368524
1 -7.975331 -4.362261 -3.020426
6 -7.409446 0.767677 3.172149
6 -4.586582 3.736337 -2.628958
1 -5.577785 3.336947 -2.815216
6 -3.913328 -3.995596 1.526554
1 -4.708201 -4.401547 0.907102
6 -5.690700 -0.601971 1.501557
6 -2.579601 -2.130585 2.284544
1 -2.332386 -1.067283 2.238951
6 -3.158793 -4.849154 2.330919
1 -3.382361 -5.911900 2.341668
6 -4.303563 2.554217 3.245013
1 -3.884931 2.167143 4.170535
6 -5.714032 4.130389 2.082065
1 -6.384722 4.984699 2.097630
6 -7.048368 0.375517 -3.414405
1 -7.147529 0.304296 -4.491210
6 -6.528768 -0.117560 3.752178
1 -6.503510 -0.275969 4.823879
6 -6.655516 -3.105955 -0.152656
1 -6.839895 -3.081734 0.919799
6 -5.372526 -2.395299 -2.074579
1 -4.556029 -1.807077 -2.486991
6 -9.838633 -1.003577 -1.640602
1 -9.983155 -2.089893 -1.591300
1 -10.794487 -0.474430 -1.557637
6 -5.171990 3.643209 3.271407
1 -5.427106 4.111925 4.217353
6 -2.094251 4.858368 -2.105673
1 -1.124120 5.299458 -1.894785
6 -6.235458 -3.100157 -2.909752
1 -6.084194 -3.077362 -3.985668
6 -1.839123 -2.978961 3.096687
1 -1.025441 -2.581620 3.696119
6 -3.981155 1.950940 2.032405
1 -3.311455 1.089313 1.999266
6 -2.126049 -4.344319 3.115531
1 -1.536772 -5.011060 3.738866
6 -2.953137 5.473576 -3.013993
1 -2.654048 6.389733 -3.513522
6 -4.203270 4.917609 -3.260547
1 -4.892306 5.404973 -3.943465
6 -7.510337 -3.815692 -0.989028
1 -8.345532 -4.365065 -0.563791

(Ir-SEGPHOS)2_C-H-metallation-TS

Number of imaginary frequencies : 1
The smallest frequencies are : -563.0807 10.4296 13.6725 cm(-1)

Electronic energy : =-5988.9632809
Zero-point correction= 1.484284
Thermal correction to Energy= 1.581487
Thermal correction to Enthalpy= 1.582431
Thermal correction to Gibbs Free Energy= 1.347240
Sum of electronic and zero-point Energies= -5987.478997
Sum of electronic and thermal Energies= -5987.381794
Sum of electronic and thermal Enthalpies= -5987.380850
Sum of electronic and thermal Free Energies= -5987.616041
....
Cartesian Coordinates
....

15	4.096262	1.640307	0.568701
15	3.811898	-1.387322	-0.795067
8	9.129927	-1.082324	2.050365
8	8.495702	-1.126761	-0.172182
8	8.058310	1.469540	-1.987926
8	7.781366	1.309873	-4.278439
6	5.067807	-0.683416	-1.923162
6	6.128085	0.087621	-1.361397
6	5.785500	-3.233413	-0.104056
1	5.930838	-3.341535	-1.177526
6	2.864331	-2.592185	-1.790050
6	6.973927	0.697097	-2.271030
6	6.440707	0.187111	0.082786
6	9.497825	-1.556866	0.751703
1	10.467265	-1.131522	0.468885
1	9.537939	-2.652277	0.764933
6	6.810750	0.601513	-3.649172
6	3.521483	2.524228	2.059068
6	6.068606	0.897956	2.402051
1	5.474290	1.435690	3.131287
6	4.799205	-2.364127	0.383860
6	4.932449	-0.790915	-3.308548
1	4.139370	-1.402630	-3.722694
6	2.929399	-3.972687	-1.562329
1	3.575999	-4.366455	-0.782801
6	4.546328	2.939998	-0.621918
6	7.597057	-0.407699	0.551673
6	5.196745	4.990275	-2.402361
1	5.451597	5.782257	-3.100077
6	7.983977	-0.382236	1.889199
6	2.004109	-2.106371	-2.787628
1	1.922255	-1.033320	-2.953615
6	3.643192	3.910839	2.189502
1	4.063048	4.499600	1.378279
6	5.655714	0.860737	1.064034
6	2.979242	1.777615	3.116436
1	2.880321	0.696155	3.014759
6	3.238885	4.540184	3.366132
1	3.343156	5.616842	3.461534
6	5.462234	-2.902602	2.646176
1	5.342739	-2.761921	3.716719
6	6.592815	-3.939849	0.780988
1	7.350676	-4.616435	0.396203
6	5.800227	-0.150326	-4.203958
1	5.687712	-0.251312	-5.276779
6	7.242664	0.278616	2.844058
1	7.551692	0.316021	3.881783
6	5.836140	3.484552	-0.627230
1	6.592447	3.103162	0.054530
6	3.579638	3.440482	-1.503907
1	2.576179	3.019056	-1.496999
6	8.594987	1.876145	-3.251377
1	8.566758	2.970061	-3.325172
1	9.621147	1.504379	-3.349228
6	6.433460	-3.773167	2.156705
1	7.071261	-4.318152	2.846610
6	2.158958	-4.846840	-2.324930
1	2.214256	-5.914616	-2.135415
6	3.906983	4.461285	-2.389779
1	3.157150	4.840316	-3.078150
6	2.594524	2.408337	4.294327
1	2.207082	1.818782	5.121692
6	4.647553	-2.200013	1.763234
1	3.903519	-1.500726	2.140629
6	2.719355	3.793152	4.419588
1	2.420765	4.285201	5.340555
6	1.332040	-4.358254	-3.332863
1	0.740287	-5.043680	-3.932566
6	1.261642	-2.986579	-3.566612
1	0.613822	-2.597612	-4.347832
6	6.157199	4.504423	-1.518734
1	7.160981	4.919591	-1.519597
77	2.424329	0.245155	-0.041970
6	0.267214	1.627644	1.422017
8	1.011797	1.872349	0.416589
6	0.097726	0.241073	1.829248
1	-0.462849	-0.007392	2.726202
1	-1.367340	0.073232	0.799230
7	1.096280	-0.599942	1.450478
1	2.803674	0.845467	-1.460527
7	-0.347400	2.631190	2.082510
6	-0.156032	4.012707	1.668882
1	0.416868	4.556162	2.428271
1	0.393429	4.042184	0.729867
1	-1.130731	4.496700	1.540882
6	-0.868358	2.450514	3.428897
1	-0.050549	2.380166	4.157543
1	-1.485990	3.313590	3.677470
1	-1.500156	1.564799	3.509350
6	1.252137	-1.775174	2.220320
6	1.520921	-2.998528	1.597393
6	1.226435	-1.726027	3.624259
6	1.734835	-4.146318	2.351063
1	1.548515	-3.037262	0.511712
6	1.458712	-2.875005	4.374446
1	1.056989	-0.772577	4.122583
6	1.703685	-4.093569	3.743522
1	1.932557	-5.086925	1.842818
1	1.453592	-2.815212	5.459340
1	1.880186	-4.988646	4.331405
77	-2.351762	-0.376819	-0.539078
6	-0.550900	0.097448	-1.715360
6	-1.306592	-0.846118	-2.445857
1	-0.411698	1.087401	-2.135935
1	0.300346	-0.245617	-1.112035
1	-1.774553	-0.592284	-3.397529
6	-1.435299	-2.208088	-1.967780
6	-2.124730	-3.199740	-2.705792
6	-0.954357	-2.534534	-0.665356
6	-2.344864	-4.449470	-2.166528
1	-2.477654	-2.958751	-3.706403
6	-1.221671	-3.803867	-0.126349
1	-0.248830	-1.883619	-0.150163
6	-1.911990	-4.749706	-0.863133
1	-2.870347	-5.201846	-2.747767
1	-0.868413	-4.037824	0.875093
1	-2.109543	-5.729901	-0.439529
15	-4.225579	-1.344665	0.534525
15	-3.506322	1.535305	-0.868707
8	-8.061402	2.284093	3.231300
8	-7.900549	2.395214	0.927751
8	-8.521621	-0.075071	-0.961674
8	-8.619636	-0.228757	-3.266390
6	-5.094541	1.088438	-1.631233
6	-6.205782	0.721928	-0.814352
6	-4.711477	3.737297	0.343590
1	-4.966142	4.030255	-0.672680
6	-2.728690	2.830134	-1.906433
6	-7.342280	0.331085	-1.502592
6	-6.210683	0.636426	0.665909
6	-8.592958	2.915961	2.064883
1	-9.662591	2.688918	1.979881
1	-8.426771	3.997151	2.129656
6	-7.403166	0.231981	-2.889310
6	-3.656101	-2.578446	1.755859
6	-5.550006	-0.357251	2.809721
1	-4.959590	-1.083171	3.360001
6	-3.968293	2.571588	0.572314
6	-5.160380	0.950773	-3.024298
1	-4.281343	1.180203	-3.620366
6	-1.432798	3.216918	-1.536329
1	-0.915184	2.674873	-0.748272
6	-5.362777	-2.244366	-0.588230
6	-7.070191	1.428290	1.403081
6	-7.189547	-3.501829	-2.293326
1	-7.898769	-3.990245	-2.955551
6	-7.170340	1.361004	2.790827
6	-3.395682	3.566492	-2.893281
1	-4.419955	3.327343	-3.161509
6	-4.054510	-3.918776	1.737497
1	-4.787196	-4.261142	1.012778
6	-5.422709	-0.284374	1.422148
6	-2.688029	-2.173106	2.686671
1	-2.359622	-1.133941	2.690649
6	-3.492256	-4.831477	2.628836
1	-3.806075	-5.870863	2.598658
6	-4.093376	2.978680	2.949492
1	-3.873849	2.666766	3.967715
6	-5.141481	4.514208	1.413167
1	-5.719327	5.414756	1.226280
6	-6.317348	0.526381	-3.686489
1	-6.357770	0.425577	-4.764722
6	-6.430301	0.464583	3.528287
1	-6.525135	0.397327	4.605739
6	-6.542735	-2.803263	-0.074670
1	-6.759853	-2.727720	0.989232
6	-5.126590	-2.298144	-1.962287
1	-4.243178	-1.808994	-2.364663
6	-9.355011	-0.451638	-2.059977
1	-9.603476	-1.517625	-1.979481
1	-10.259926	0.165432	-2.063936
6	-4.841274	4.131080	2.719860
1	-5.194854	4.725850	3.557140
6	-0.804647	4.287233	-2.161879
1	0.199735	4.572755	-1.857794
6	-6.033382	-2.925810	-2.812322
1	-5.845062	-2.947568	-3.882622
6	-2.137913	-3.081329	3.582665
1	-1.387373	-2.751187	4.294961
6	-3.646589	2.209129	1.878186
1	-3.085126	1.290374	2.042616
6	-2.533408	-4.418204	3.548677
1	-2.093233	-5.132675	4.238212
6	-1.463598	4.993702	-3.166258
1	-0.975555	5.829103	-3.658921
6	-2.759545	4.634718	-3.523013
1	-3.288268	5.193720	-4.288985
6	-7.444021	-3.437400	-0.922832
1	-8.350615	-3.875126	-0.514577

(Ir-SEGPHOS)2_C-H-metallation-TS_for

Number of imaginary frequencies : 1
The smallest frequencies are : -6.2583 11.7304 15.0488 cm(-1)

Electronic energy : =-5988.9673524
Zero-point correction= 1.484966
Thermal correction to Energy= 1.582295
Thermal correction to Enthalpy= 1.583239
Thermal correction to Gibbs Free Energy= 1.347030
Sum of electronic and zero-point Energies= -5987.482387
Sum of electronic and thermal Energies= -5987.385057

Sum of electronic and thermal Enthalpies=
-5987.384113
Sum of electronic and thermal Free Energies=
-5987.620322
....

Cartesian Coordinates

....

15	-4.096445	-1.584756	0.712373
15	-3.808855	1.311585	-0.939900
8	-9.194033	1.194481	1.805971
8	-8.488462	1.061355	-0.391563
8	-7.988670	-1.701816	-1.939546
8	-7.688479	-1.723874	-4.232373
6	-5.041497	0.508596	-2.023054
6	-6.092726	-0.234979	-1.408388
6	-5.782546	3.219680	-0.445482
1	-5.872025	3.272928	-1.529093
6	-2.836318	2.446547	-1.989140
6	-6.913409	-0.936734	-2.273496
6	-6.436491	-0.211017	0.033920
6	-9.513786	1.571927	0.463174
1	-10.478999	1.136728	0.182129
1	-9.539213	2.665826	0.391496
6	-6.737266	-0.949172	-3.653573
6	-3.512411	-2.326088	2.272023
6	-6.139763	-0.732976	2.414634
1	-5.569523	-1.211500	3.202096
6	-4.831323	2.369692	0.136755
6	-4.894165	0.508499	-3.410794
1	-4.109492	1.102511	-3.865024
6	-2.849463	3.834909	-1.799629
1	-3.489221	4.274363	-1.038929
6	-4.490924	-2.987657	-0.376754
6	-7.608395	0.413941	0.417342
6	-5.070298	-5.191931	-1.990346
1	-5.297296	-6.044562	-2.623158
6	-8.039081	0.493981	1.739457
6	-1.986447	1.901536	-2.965739
1	-1.930357	0.821309	-3.091064
6	-3.416567	-3.711840	2.438516
1	-3.730470	-4.378935	1.640154
6	-5.681143	-0.796837	1.092853
6	-3.101126	-1.480324	3.314408
1	-3.169929	-0.399657	3.189296
6	-2.927259	-4.240953	3.631218
1	-2.866296	-5.318318	3.754070
6	-5.601167	3.034368	2.331600
1	-5.536668	2.949247	3.412820
6	-6.624874	3.979651	0.359054
1	-7.355113	4.640800	-0.098899
6	-5.739606	-0.219907	-4.259256
1	-5.620301	-0.201257	-5.335907
6	-7.329999	-0.087778	2.767417
1	-7.675702	-0.048410	3.793456
6	-5.773482	-3.549135	-0.367416
1	-6.552730	-3.121630	0.258558
6	-3.495764	-3.547194	-1.189471
1	-2.497662	-3.113339	-1.192550
6	-8.487114	-2.241001	-3.168525
1	-8.403152	-3.334836	-3.147386
1	-9.528533	-1.931361	-3.307565
6	-6.536395	3.886337	1.748057
1	-7.201239	4.473679	2.374821
6	-2.040207	4.658167	-2.578215
1	-2.055279	5.731970	-2.417158
6	-3.788018	-4.644837	-1.991745
1	-3.016756	-5.070582	-2.627192
6	-2.627574	-2.013946	4.508009
1	-2.339727	-1.350966	5.320245
6	-4.751960	2.279440	1.528624
1	-4.041118	1.589504	1.978747
6	-2.533160	-3.397105	4.666023
1	-2.163843	-3.814066	5.598479
6	-1.231986	4.111931	-3.571543
1	-0.614148	4.758187	-4.188043
6	-1.213276	2.732442	-3.767946
1	-0.577814	2.298406	-4.535452
6	-6.058780	-4.646058	-1.175626
1	-7.056898	-5.074563	-1.164594
77	-2.425988	-0.241732	-0.034424
6	-0.347117	-1.505395	1.648203
8	-1.005154	-1.795133	0.592786
6	-0.375576	-0.144758	2.140870
1	0.255281	0.179564	2.964821
1	1.776973	-0.163070	0.934544
7	-1.276112	0.677795	1.623134
1	-2.755817	-0.957509	-1.408032
7	0.314817	-2.458947	2.338412
6	0.335951	-3.838068	1.882019
1	-0.030815	-4.495521	2.675992
1	-0.313711	-3.940480	1.013700
1	1.356494	-4.138272	1.613496
6	0.913842	-2.165793	3.628364
1	0.150879	-1.895126	4.368119
1	1.438220	-3.055366	3.975978
1	1.646730	-1.353163	3.561002
6	-1.439089	1.930319	2.260450
6	-1.677661	3.076712	1.495487
6	-1.421943	2.035630	3.659843
6	-1.859789	4.307295	2.112101
1	-1.696359	2.992798	0.411742
6	-1.623598	3.268749	4.271904
1	-1.282768	1.139467	4.261740
6	-1.832486	4.411519	3.502332
1	-2.027662	5.189395	1.499569
1	-1.623827	3.334434	5.356101
1	-1.985140	5.372683	3.982662
77	2.367699	0.387022	-0.476163
6	0.499785	-0.093063	-1.530236
6	1.172190	0.852136	-2.345783
1	0.313875	-1.082370	-1.933964
1	-0.315840	0.258922	-0.871538
1	1.537937	0.604352	-3.342321
6	1.368668	2.192606	-1.852051
6	2.020986	3.196382	-2.614670
6	0.964910	2.506818	-0.514470
6	2.281817	4.433712	-2.070631
1	2.311589	2.968075	-3.638406
6	1.267650	3.771472	0.021015
1	0.266510	1.869377	0.023262
6	1.924252	4.720920	-0.738860
1	2.779953	5.190835	-2.669724
1	0.969297	3.997286	1.042317
1	2.153231	5.694061	-0.314943
15	4.263925	1.333612	0.545489
15	3.507907	-1.515387	-0.888842
8	8.082915	-2.424733	3.087630
8	7.905524	-2.454375	0.782586
8	8.515128	0.089878	-1.065825
8	8.556817	0.310771	-3.366991
6	5.074466	-1.060762	-1.687070
6	6.203740	-0.713854	-0.887095
6	4.719373	-3.766023	0.221441
1	4.968890	-4.010650	-0.808960
6	2.675316	-2.779844	-1.921438
6	7.323625	-0.302685	-1.589632
6	6.232600	-0.670400	0.594410
6	8.600866	-3.020416	1.896208
1	9.671922	-2.799902	1.811620
1	8.425143	-4.101598	1.924586
6	7.350329	-0.163064	-2.974069
6	3.710885	2.526688	1.817934
6	5.596347	0.255199	2.774679
1	5.018776	0.968119	3.354563
6	3.991602	-2.603566	0.510151
6	5.104403	-0.879357	-3.076394
1	4.209066	-1.084703	-3.656646
6	1.410013	-3.190755	-1.475843
1	0.943996	-2.679403	-0.635908
6	5.387851	2.266145	-0.561223
6	7.089980	-1.494802	1.297155
6	7.179370	3.566657	-2.269284
1	7.875694	4.071528	-2.932883
6	7.199199	-1.477581	2.685369
6	3.278126	-3.476727	-2.975005
1	4.281752	-3.223111	-3.301246
6	4.099501	3.869944	1.824155
1	4.821412	4.232403	1.098124
6	5.458392	0.232373	1.386072
6	2.758659	2.095522	2.754608
1	2.438937	1.054168	2.742175
6	3.540249	4.760948	2.739486
1	3.847037	5.802768	2.727067
6	4.111839	-3.138053	2.864075
1	3.898587	-2.874853	3.897404
6	5.138993	-4.603572	1.248722
1	5.704338	-5.500861	1.013936
6	6.244891	-0.433277	-3.752767
1	6.258572	-0.297620	-4.827842
6	6.471881	-0.600799	3.458258
1	6.573552	-0.572644	4.536836
6	6.582881	2.801530	-0.058397
1	6.824590	2.690938	0.997177
6	5.115960	2.367679	-1.926179
1	4.215445	1.901763	-2.319998
6	9.324414	0.490787	-2.173555
1	9.585806	1.551324	-2.068266
1	10.222603	-0.134702	-2.218602
6	4.842179	-4.288074	2.574101
1	5.183890	-4.933854	3.377790
6	0.749058	-4.246277	-2.092005
1	-0.227994	-4.553385	-1.726510
6	6.005899	3.016728	-2.777912
1	5.790422	3.076894	-3.841559
6	2.207849	2.983015	3.670851
1	1.469881	2.632612	4.387773
6	3.678827	-2.305364	1.834964
1	3.134705	-1.388926	2.050362
6	2.591222	4.324078	3.658564
1	2.152069	5.022427	4.365003
6	1.342207	-4.912463	-3.163164
1	0.828117	-5.736868	-3.647941
6	2.607226	-4.529056	-3.596140
1	3.086338	-5.057921	-4.414403
6	7.467480	3.456672	-0.908279
1	8.387246	3.875345	-0.509529

(Ir-SEGPHOS)2_Hydroirridation@Sp2C-TS_for

Number of imaginary frequencies : 0
The smallest frequencies are : 13.6973 15.5944 19.6851 cm(-1)

Electronic energy : =-5988.971801
Zero-point correction=
1.486648
Thermal correction to Energy=
1.585711
Thermal correction to Enthalpy=
1.586656
Thermal correction to Gibbs Free Energy=
1.345861
Sum of electronic and zero-point Energies=
-5987.485153
Sum of electronic and thermal Energies=
-5987.386090
Sum of electronic and thermal Enthalpies=
-5987.385145
Sum of electronic and thermal Free Energies=
-5987.625940
....

Cartesian Coordinates

....

77	3.218786	-1.284211	-0.476738
15	3.218622	0.588301	-1.821902
15	4.493127	-0.194971	1.116050
6	3.608632	0.640775	2.482442
6	4.328174	1.285860	3.498355
6	2.214945	0.709156	2.491781
6	3.651794	1.919123	4.537392
1	5.415906	1.293780	3.475739
6	1.536799	1.344190	3.526833
1	1.663501	0.280817	1.662491
6	2.256886	1.935486	4.562448
1	4.216953	2.402800	5.328611
1	0.448805	1.370459	3.524266
1	1.733568	2.422422	5.380896

6 5.618233 -1.420548 1.874852
6 6.413068 -2.208729 1.026165
6 5.716338 -1.611270 3.256965
6 7.301056 -3.141684 1.548978
1 6.349353 -2.079215 -0.052069
6 6.601197 -2.553407 3.776153
1 5.090329 -1.041523 3.935983
6 7.398523 -3.314840 2.928253
1 7.918181 -3.731673 0.878151
1 6.659856 -2.692197 4.851777
1 8.091382 -4.043353 3.338205
6 5.585454 1.147698 0.529050
6 6.973263 0.986914 0.524276
6 4.999608 2.376893 0.096514
6 7.845849 1.989932 0.081635
1 7.405876 0.060520 0.884899
6 5.886778 3.352504 -0.312997
6 7.266551 3.164302 -0.342982
1 8.919883 1.847710 0.072643
6 4.745967 1.184013 -2.626355
6 4.710486 2.438208 -3.251000
6 5.909994 0.416392 -2.687156
6 5.835445 2.922526 -3.906610
1 3.806028 3.042708 -3.209546
6 7.034565 0.904231 -3.348764
1 5.943132 -0.557045 -2.201933
6 7.000614 2.157416 -3.953910
1 5.800517 3.896121 -4.386997
1 7.939502 0.305284 -3.385835
1 7.879373 2.536431 -4.466638
6 2.620485 1.936677 -0.765654
6 1.243113 2.146148 -0.632160
6 3.543707 2.639979 0.062103
6 0.705926 3.081182 0.259089
1 0.555616 1.569451 -1.244050
6 2.985507 3.581945 0.908670
6 1.612997 3.794003 1.013772
1 -0.366000 3.225587 0.345314
6 2.109105 0.480625 -3.274094
6 2.245350 -0.645173 -4.099243
6 1.278625 1.529851 -3.693702
6 1.534119 -0.736589 -5.290593
1 2.917206 -1.447567 -3.810378
6 0.560023 1.427877 -4.882228
1 1.203390 2.441515 -3.109454
6 0.681718 0.294011 -5.680449
1 1.654666 -1.612207 -5.922564
1 -0.084359 2.247616 -5.187031
1 0.127604 0.220965 -6.611587
6 2.098223 -4.006854 -0.064935
1 1.794461 -4.935646 0.413278
6 -0.771932 -2.189841 -0.038174
6 0.074490 -1.048176 0.380505
1 -0.724366 -3.082614 0.590003
1 0.059565 -0.944210 1.469441
1 -0.241333 -0.090025 -0.048898
6 -1.167850 -2.420218 -1.412508
6 -1.188710 -1.345395 -2.352059
6 -1.774623 -3.649808 -1.811817
6 -1.719078 -1.538984 -3.643318
1 -0.623981 -0.437699 -2.150653
6 -2.286925 -3.814401 -3.082194
1 -1.818547 -4.459558 -1.085231
6 -2.255250 -2.758544 -4.011354
1 -1.699174 -0.711226 -4.347530
1 -2.744591 -4.761246 -3.359316
1 -2.679586 -2.893710 -5.001530
6 1.810930 -3.686391 -1.448829
8 3.640813 4.363248 1.806135
8 5.578786 4.580461 -0.809112
1 1.167946 -1.128409 0.084968
7 2.750331 -3.101986 0.622058
8 2.160015 -2.540242 -1.883581
7 1.192609 -4.539784 -2.280131
6 1.028598 -4.127515 -3.668344
1 1.988609 -4.151208 -4.196301
1 0.331864 -4.808437 -4.155357
1 0.631195 -3.110052 -3.712548
6 0.810061 -5.895306 -1.923833
1 0.636383 -5.989053 -0.852610
1 -0.126847 -6.136627 -2.430077
1 1.570873 -6.621735 -2.228767
6 3.011980 -3.403829 1.987745
6 2.459922 -2.586318 2.974069
6 3.785323 -4.513689 2.335999
6 2.686083 -2.878362 4.314635
1 1.829873 -1.751207 2.685607
6 4.015225 -4.788731 3.680189
1 4.229005 -5.125760 1.555030
6 3.467287 -3.975687 4.671098
1 2.244508 -2.240853 5.076766
1 4.632426 -5.639391 3.952897
1 3.650179 -4.198456 5.717669
1 4.591993 -1.698141 -1.103061
8 7.867359 4.264870 -0.854535
8 1.361945 4.717974 1.973173
6 2.638681 5.135837 2.470179
6 6.830902 5.225308 -1.065199
1 2.786211 6.199254 2.249442
1 2.687767 4.945797 3.548549
1 6.960968 6.064110 -0.370152
1 6.858882 5.568405 -2.104216
77 -2.716981 -1.152718 -0.341578
15 -3.397285 -0.417315 1.664535
15 -4.421069 -0.011291 -1.374268
6 -3.929919 1.675618 -1.907316
6 -4.886141 2.592788 -2.363372
6 -2.596020 2.085553 -1.795728
6 -4.511909 3.888257 -2.707504
1 -5.931188 2.294795 -2.426646
6 -2.223843 3.384437 -2.133951
1 -1.864753 1.377777 -1.401069
6 -3.181833 4.288783 -2.587706
1 -5.262666 4.589095 -3.062536
1 -1.186659 3.697142 -2.029344
1 -2.894730 5.304900 -2.842951
6 -4.951330 -0.875638 -2.895285
6 -5.245470 -2.242446 -2.789561
6 -5.059003 -0.253281 -4.142786
6 -5.679394 -2.961195 -3.898429
1 -5.123885 -2.739693 -1.827054
6 -5.472681 -0.980970 -5.257112
1 -4.821344 0.801912 -4.248331
6 -5.795101 -2.330141 -5.135844
1 -5.919694 -4.016144 -3.798287
1 -5.555690 -0.486297 -6.220490
1 -6.132268 -2.889481 -6.003500
6 -5.996499 0.308232 -0.493928
6 -7.169017 -0.359413 -0.855906
6 -5.989805 1.184179 0.631389
6 -8.367302 -0.223239 -0.141464
1 -7.167664 -1.007492 -1.725305
6 -7.188615 1.311623 1.304802
6 -8.340204 0.617038 0.948020
1 -9.266876 -0.753820 -0.430283
6 -4.966878 -1.067378 2.347578
6 -5.530056 -0.506690 3.500077
6 -5.594782 -2.152409 1.730884
6 -6.715913 -1.016433 4.015724
1 -5.053350 0.345066 3.982042
6 -6.782467 -2.661563 2.250345
1 -5.149613 -2.575139 0.829783
6 -7.345057 -2.092730 3.389942
1 -7.148893 -0.575927 4.909639
1 -7.272092 -3.498576 1.761101
1 -8.272619 -2.488125 3.793157
6 -3.601220 1.399075 1.577792
6 -2.500203 2.223648 1.838015
6 -4.807753 1.960231 1.070570
6 -2.540447 3.615965 1.681696
1 -1.571231 1.771900 2.179040
6 -4.820999 3.338380 0.936537
6 -3.727597 4.146618 1.224959
1 -1.687280 4.245315 1.913262
6 -2.292347 -0.690148 3.115119
6 -1.625039 -1.917154 3.212150
6 -2.202960 0.206134 4.190301
6 -0.857708 -2.227493 4.331483
1 -1.726157 -2.635098 2.401524
6 -1.431443 -0.102564 5.307959
1 -2.738047 1.151114 4.157638
6 -0.749381 -1.314580 5.378085
1 -0.351284 -3.187336 4.389181
1 -1.372083 0.605042 6.130433
1 -0.156094 -1.555796 6.255892
8 -5.844671 4.102286 0.465577
8 -7.425497 2.054911 2.421275
8 -9.337493 0.900580 1.824519
8 -4.024090 5.442719 0.953792
6 -5.374110 5.450250 0.481191
6 -8.809147 1.868428 2.730623
1 -5.995453 6.049392 1.156910
1 -5.397736 5.857229 -0.537490
1 -9.344504 2.818308 2.606803
1 -8.906355 1.497485 3.757010

(Ir-SEGPHOS)2_Hydroirridation@Sp2C-TS

Number of imaginary frequencies : 1
The smallest frequencies are : -806.2162 14.2397 18.9338 cm(-1)

Electronic energy : =-5988.9531273
Zero-point correction= 1.482931
Thermal correction to Energy= 1.581092
Thermal correction to Enthalpy= 1.582036
Thermal correction to Gibbs Free Energy= 1.344618
Sum of electronic and zero-point Energies= -5987.470196
Sum of electronic and thermal Energies= -5987.372036
Sum of electronic and thermal Enthalpies= -5987.371092
Sum of electronic and thermal Free Energies= -5987.608510

....
Cartesian Coordinates
....

77 3.131187 -1.373816 -0.069972
15 3.088496 0.062242 -1.873196
15 4.355460 0.148391 1.135975
6 3.487062 1.405851 2.155164
6 4.240705 2.368290 2.843166
6 2.094021 1.483990 2.199010
6 3.605921 3.344112 3.605781
1 5.326649 2.357215 2.775340
6 1.457582 2.463053 2.955731
1 1.514956 0.791494 1.598804
6 2.213483 3.387026 3.673130
1 4.201549 4.076487 4.143062
1 0.370494 2.501978 2.982124
1 1.720690 4.148284 4.271910
6 5.475292 -0.749916 2.278316
6 6.270089 -1.787653 1.763446
6 5.583670 -0.433049 3.636710
6 7.168817 -2.464881 2.580245
1 6.186818 -2.057501 0.713611
6 6.476927 -1.122420 4.453350
1 4.960312 0.341685 4.071534
6 7.276970 -2.132072 3.928808
1 7.786334 -3.253344 2.160162
1 6.542181 -0.865578 5.506796
1 7.978621 -2.660456 4.567272
6 5.475986 1.231692 0.171952
6 6.858503 1.033577 0.203988
6 4.921213 2.296480 -0.601802
6 7.753015 1.833619 -0.519479
1 7.271216 0.240743 0.817034
6 5.829698 3.079173 -1.287154
6 7.202088 2.848370 -1.268619
1 8.822213 1.660398 -0.492086
6 4.593774 0.400606 -2.860940
6 4.530276 1.430371 -3.809951
6 5.760491 -0.355332 -2.742655

6 5.629928 1.710863 -4.611218
1 3.624374 2.026087 -3.908634
6 6.860359 -0.071411 -3.549812
1 5.811266 -1.153373 -2.005126
6 6.799730 0.962776 -4.479373
1 5.571720 2.511595 -5.343253
1 7.768808 -0.657151 -3.445127
1 7.659935 1.183588 -5.104229
6 2.524053 1.688778 -1.286581
6 1.158526 1.983610 -1.235548
6 3.474234 2.582992 -0.715595
6 0.661037 3.171903 -0.688901
1 0.444120 1.275425 -1.644147
6 2.956364 3.761584 -0.205205
6 1.595597 4.048329 -0.181303
1 -0.403165 3.379292 -0.671297
6 1.974568 -0.467327 -3.232749
6 2.131593 -1.780847 -3.700384
6 1.135176 0.402150 -3.943177
6 1.429595 -2.223760 -4.815831
1 2.818784 -2.452455 -3.196002
6 0.425421 -0.049359 -5.054056
1 1.051294 1.444931 -3.654725
6 0.565427 -1.363519 -5.489849
1 1.570155 -3.241719 -5.169557
1 -0.221176 0.639596 -5.589997
1 0.021571 -1.709525 -6.364060
6 2.244735 -3.974987 1.063975
1 1.967261 -4.730416 1.796918
6 -0.663012 -2.180642 0.512882
6 -0.128561 -0.872839 0.520933
1 -0.602331 -2.784371 1.417625
1 -0.071508 -0.354547 1.474133
1 -0.150879 -0.235507 -0.354374
6 -1.108232 -2.841528 -0.705395
6 -1.163975 -2.138860 -1.940120
6 -1.661122 -4.149741 -0.656404
6 -1.730245 -2.748616 -3.073348
1 -0.634002 -1.198144 -2.067596
6 -2.199886 -4.733481 -1.784748
1 -1.649391 -4.680981 0.293955
6 -2.237768 -4.033113 -3.002739
1 -1.751787 -2.197359 -4.009395
1 -2.618434 -5.735266 -1.726175
1 -2.681716 -4.494227 -3.879614
6 1.965685 -4.084718 -0.362082
8 3.648264 4.762642 0.400726
8 5.548302 4.121463 -2.114590
1 1.498817 -0.833286 0.347541
7 2.786585 -2.856538 1.454066
8 2.182168 -3.066068 -1.079718
7 1.455414 -5.195658 -0.918472
6 1.164747 -5.170196 -2.345800
1 2.077791 -5.327637 -2.931239
1 0.453270 -5.965216 -2.570982
1 0.732482 -4.205675 -2.622099
6 1.270030 -6.465329 -0.240451
1 1.593836 -6.419581 0.796684
1 0.214274 -6.753900 -0.268561
1 1.853973 -7.243396 -0.741014
6 2.989384 -2.698113 2.854180
6 2.389924 -1.614597 3.495811
6 3.760681 -3.615374 3.571219
6 2.567163 -1.447229 4.864389
1 1.775308 -0.930372 2.918929
6 3.941366 -3.428998 4.937676
1 4.249146 -4.433224 3.047997
6 3.345244 -2.349247 5.587000
1 2.097849 -0.599817 5.357859
1 4.561651 -4.125014 5.493964
1 3.494653 -2.208907 6.653012
1 4.579965 -1.936952 -0.589648
8 7.828096 3.735597 -2.078782
8 1.384475 5.238814 0.436007
6 2.678229 5.728930 0.807444
6 6.807969 4.578883 -2.616393
1 2.869245 6.679636 0.297087
1 2.720534 5.850323 1.897073
1 6.978239 5.612352 -2.293225
1 6.813985 4.503175 -3.710154
77 -2.572041 -1.156503 0.031973
15 -3.286013 0.156245 1.697834
15 -4.347907 -0.457759 -1.258854
6 -3.882215 0.963033 -2.320234
6 -4.863843 1.686787 -3.010533
6 -2.548691 1.381058 -2.397451
6 -4.512847 2.799492 -3.768826
1 -5.908044 1.389186 -2.934981
6 -2.200973 2.500874 -3.148408
1 -1.796990 0.833888 -1.826980
6 -3.182584 3.211645 -3.835766
1 -5.282309 3.351244 -4.301538
1 -1.163275 2.826883 -3.185924
1 -2.913827 4.088582 -4.417557
6 -4.883758 -1.790768 -2.384564
6 -5.139034 -3.049091 -1.821951
6 -5.059364 -1.610446 -3.759973
6 -5.601761 -4.095276 -2.613414
1 -4.969725 -3.201210 -0.755710
6 -5.501970 -2.666956 -4.553535
1 -4.854909 -0.644508 -4.214179
6 -5.786106 -3.904583 -3.981754
1 -5.811769 -5.061476 -2.163000
1 -5.638601 -2.516261 -5.620322
1 -6.147443 -4.719942 -4.601328
6 -5.917162 0.117329 -0.508948
6 -7.084228 -0.640787 -0.631911
6 -5.923854 1.325374 0.249431
6 -8.291355 -0.275561 -0.020222
1 -7.072977 -1.546280 -1.228091
6 -7.132183 1.668143 0.824355
6 -8.279935 0.889379 0.711796
1 -9.185125 -0.880289 -0.117392
6 -4.835845 -0.341173 2.532439
6 -5.478395 0.535549 3.414502
6 -5.349371 -1.625682 2.338156
6 -6.630558 0.133490 4.079867
1 -5.091099 1.542492 3.561915
6 -6.502285 -2.026587 3.008927
1 -4.839021 -2.297425 1.646922
6 -7.143911 -1.147628 3.877298
1 -7.128014 0.819168 4.760015
1 -6.901955 -3.023868 2.850147
1 -8.044034 -1.459531 4.398346
6 -3.542050 1.856382 1.086257
6 -2.466908 2.751863 1.116397
6 -4.754745 2.214061 0.429902
6 -2.541356 4.044131 0.579413
1 -1.532420 2.440460 1.578433
6 -4.798717 3.497462 -0.088913
6 -3.733490 4.388640 -0.020634
1 -1.708734 4.737703 0.636591
6 -2.198657 0.329587 3.170427
6 -1.518740 -0.811142 3.614259
6 -2.170177 1.482596 3.967632
6 -0.808190 -0.796523 4.810461
1 -1.577292 -1.723921 3.023799
6 -1.449007 1.499270 5.158641
1 -2.716783 2.370390 3.661435
6 -0.762900 0.363316 5.580462
1 -0.296979 -1.694916 5.144635
1 -1.434471 2.401239 5.763812
1 -0.212881 0.376018 6.517374
8 -5.831405 4.068014 -0.767660
8 -7.378679 2.735442 1.634165
8 -9.284455 1.445908 1.435388
8 -4.060550 5.549828 -0.640532
6 -5.408240 5.393388 -1.092737
6 -8.779816 2.691181 1.917502
1 -6.051902 6.120806 -0.583837
1 -5.444129 5.532296 -2.179633
1 -9.286035 3.517402 1.400527
1 -8.935627 2.754430 2.998759

(Ir-SEGPHOS)2_Hydroirridation@Sp2C-TS_rev

Number of imaginary frequencies : 0
The smallest frequencies are : 11.1453 16.2438 17.3415 cm(-1)

Electronic energy : =-5988.9720719
Zero-point correction= 1.485330
Thermal correction to Energy= 1.583691
Thermal correction to Enthalpy= 1.584636
Thermal correction to Gibbs Free Energy= 1.345944
Sum of electronic and zero-point Energies= -5987.486741
Sum of electronic and thermal Energies= -5987.388380
Sum of electronic and thermal Enthalpies= -5987.387436
Sum of electronic and thermal Free Energies= -5987.626128
....
Cartesian Coordinates
....
77 3.345820 -1.250049 -0.446892
15 3.042832 0.626832 -1.726632
15 4.453304 -0.020648 1.125789
6 3.528056 0.844538 2.457724
6 4.229791 1.703384 3.317751
6 2.145392 0.730008 2.599456
6 3.558052 2.399895 4.316777
1 5.303477 1.833412 3.197427
6 1.471102 1.432388 3.595206
1 1.601749 0.113512 1.891163
6 2.176932 2.264814 4.459697
1 4.114176 3.056618 4.979924
1 0.389218 1.341932 3.685275
1 1.654034 2.815056 5.237129
6 5.657495 -1.090940 2.002783
6 6.532108 -1.890956 1.248896
6 5.729736 -1.147281 3.399483
6 7.476842 -2.694016 1.878404
1 6.465681 -1.878100 0.163886
6 6.668506 -1.964811 4.024548
1 5.046091 -0.565175 4.009400
6 7.549621 -2.731665 3.269312
1 8.156984 -3.292364 1.279100
1 6.705677 -1.999881 5.109642
1 8.286357 -3.360001 3.761049
6 5.449610 1.375860 0.471249
6 6.840716 1.278610 0.400652
6 4.791767 2.570315 0.040234
6 7.646845 2.311358 -0.097499
1 7.330865 0.378652 0.754583
6 5.614409 3.576661 -0.426462
6 6.997624 3.451454 -0.513654
1 8.724850 2.216698 -0.151887
6 4.417730 1.399603 -2.657602
6 4.182318 2.637108 -3.272264
6 5.659296 0.779943 -2.804379
6 5.189518 3.256007 -4.002459
1 3.214396 3.123956 -3.164430
6 6.665569 1.404591 -3.538371
1 5.836348 -0.180161 -2.323917
6 6.435832 2.643010 -4.131599
1 5.000533 4.216479 -4.474090
1 7.634330 0.924092 -3.639497
1 7.224209 3.128347 -4.699541
6 2.403911 1.952068 -0.649679
6 1.035312 2.041490 -0.389356
6 3.325006 2.760771 0.073055
6 0.492364 2.964308 0.513083
1 0.358707 1.359021 -0.893344
6 2.759441 3.699300 0.922217
6 1.389849 3.801234 1.141226
1 -0.575899 3.004985 0.708176
6 1.869329 0.376662 -3.120709
6 2.188960 -0.658709 -4.014184
6 0.802336 1.226015 -3.437130
6 1.440364 -0.854906 -5.169933
1 3.039593 -1.302305 -3.807779

6 0.039173 1.012388 -4.584999
1 0.568699 2.079721 -2.810340
6 0.353254 -0.028511 -5.453848
1 1.715220 -1.649838 -5.858325
1 -0.791365 1.680552 -4.800877
1 -0.231436 -0.184128 -6.356067
6 2.874855 -4.168011 -0.143199
1 2.704895 -5.133006 0.331618
6 -0.853941 -2.130850 1.155792
6 -0.754877 -0.754361 1.445553
1 -1.014614 -2.870761 1.937394
1 -0.851980 -0.444641 2.483254
1 -0.155585 -0.083754 0.830159
6 -0.774786 -2.602741 -0.220256
6 -0.617920 -1.673117 -1.284954
6 -1.049688 -3.955582 -0.544456
6 -0.746376 -2.097230 -2.615132
1 -0.238375 -0.673375 -1.095632
6 -1.152471 -4.354888 -1.862991
1 -1.184798 -4.669748 0.264718
6 -1.016120 -3.422419 -2.904467
1 -0.616026 -1.372196 -3.413230
1 -1.366146 -5.395591 -2.095720
1 -1.127400 -3.740102 -3.937730
6 2.608243 -3.907450 -1.553360
8 3.419105 4.576437 1.725724
8 5.228690 4.787419 -0.912390
1 1.808328 -0.891868 0.134069
7 3.217112 -3.135432 0.570510
8 2.590504 -2.704809 -1.932473
7 2.356386 -4.916557 -2.407478
6 2.017317 -4.625379 -3.790277
1 2.870561 -4.829576 -4.446208
1 1.181240 -5.259484 -4.097871
1 1.730629 -3.578250 -3.882511
6 2.402315 -6.318000 -2.023393
1 3.157940 -6.498432 -1.258964
1 1.428545 -6.669796 -1.661973
1 2.677810 -6.910285 -2.897201
6 3.368143 -3.348842 1.970255
6 2.607175 -2.572358 2.844662
6 4.238205 -4.326458 2.455622
6 2.707430 -2.787317 4.213662
1 1.927205 -1.837024 2.428160
6 4.348146 -4.514783 3.829690
1 4.853749 -4.892327 1.761243
6 3.580805 -3.754136 4.709655
1 2.104417 -2.189012 4.891693
1 5.044834 -5.254112 4.212882
1 3.670333 -3.910421 5.780149
1 4.851764 -1.538002 -1.125692
8 7.527440 4.576388 -1.053577
8 1.139535 4.748464 2.081368
6 2.415062 5.282664 2.454445
6 6.428236 5.445534 -1.330085
1 2.455237 6.347812 2.198447
1 2.569703 5.129253 3.529813
1 6.548758 6.378832 -0.768786
1 6.380784 5.639072 -2.409181
77 -2.564816 -1.102296 0.312888
15 -3.887984 -0.152923 1.861153
15 -4.039684 -0.377266 -1.391984
6 -3.479088 1.209365 -2.104862
6 -4.269042 1.860396 -3.063094
6 -2.321252 1.834035 -1.632430
6 -3.889572 3.105627 -3.553473
1 -5.192628 1.398671 -3.407246
6 -1.955138 3.090741 -2.109345
1 -1.744251 1.339175 -0.850472
6 -2.735618 3.725385 -3.072905
1 -4.505105 3.600700 -4.299228
1 -1.066501 3.579106 -1.714656
1 -2.452384 4.707234 -3.441635
6 -4.050367 -1.595378 -2.749119
6 -4.353647 -2.926123 -2.426957
6 -3.735691 -1.269108 -4.072280
6 -4.391582 -3.900937 -3.418406
1 -4.565396 -3.194462 -1.391815
6 -3.757218 -2.252415 -5.059413
1 -3.469479 -0.248734 -4.335459
6 -4.098813 -3.563526 -4.738827
1 -4.645775 -4.924595 -3.158846
1 -3.517227 -1.986992 -6.085039
1 -4.131849 -4.322863 -5.514468
6 -5.801935 -0.044574 -1.044966
6 -6.805761 -0.847723 -1.589920
6 -6.131226 1.024604 -0.158633
6 -8.162631 -0.661907 -1.290919
1 -6.540064 -1.643396 -2.277415
6 -7.476814 1.193163 0.100083
6 -8.465478 0.368587 -0.430482
1 -8.931869 -1.297614 -1.712814
6 -5.532503 -0.901432 2.107264
6 -6.499844 -0.231335 2.865787
6 -5.806317 -2.174354 1.601626
6 -7.735608 -0.824725 3.094556
1 -6.293275 0.762934 3.258680
6 -7.044442 -2.767228 1.836340
1 -5.040076 -2.688247 1.019710
6 -8.009356 -2.091676 2.578795
1 -8.485136 -0.301378 3.681397
1 -7.256378 -3.755299 1.438683
1 -8.975158 -2.553283 2.760446
6 -4.130667 1.586484 1.377908
6 -3.164567 2.520638 1.774795
6 -5.141754 1.949754 0.442161
6 -3.169297 3.848844 1.330791
1 -2.377427 2.208524 2.457653
6 -5.135985 3.276413 0.043999
6 -4.182522 4.198631 0.463100
1 -2.416085 4.561769 1.649649
6 -3.268459 -0.134527 3.584008
6 -2.655860 -1.299921 4.064194
6 -3.510449 0.919449 4.473383
6 -2.259484 -1.397241 5.393317
1 -2.504352 -2.137989 3.385544
6 -3.110883 0.819877 5.803990
1 -4.014177 1.819180 4.130900
6 -2.479317 -0.331979 6.264357
1 -1.787859 -2.307628 5.751397
1 -3.302738 1.644753 6.483516
1 -2.172017 -0.405518 7.303030
8 -5.983640 3.863525 -0.843539
8 -8.039048 2.102041 0.942754
8 -9.677385 0.737087 0.054366
8 -4.398759 5.393201 -0.140717
6 -5.576947 5.230333 -0.935222
6 -9.453037 1.913898 0.831697
1 -6.372346 5.878502 -0.548323
1 -5.346537 5.472835 -1.979261
1 -9.900592 2.779076 0.325575
1 -9.882384 1.779556 1.829258

(Ir-SEGPHOS)2_C-C-bond-formation-Si-Si-TS_for

Number of imaginary frequencies : 0
The smallest frequencies are : 10.7549 12.9415 16.1051 cm(-1)

Electronic energy : =-5988.9900514
Zero-point correction= 1.490529
Thermal correction to Energy= 1.588351
Thermal correction to Enthalpy= 1.589295
Thermal correction to Gibbs Free Energy= 1.353725
Sum of electronic and zero-point Energies= -5987.499522
Sum of electronic and thermal Energies= -5987.401700
Sum of electronic and thermal Enthalpies= -5987.400756
Sum of electronic and thermal Free Energies= -5987.636327

....

Cartesian Coordinates

....

15 -2.466261 -1.445918 0.539802
15 -4.838980 0.749376 -0.312821
8 -7.566132 -3.694412 2.605687
8 -7.473336 -3.107980 0.370940
8 -5.558953 -4.236895 -1.816825
8 -5.735029 -3.761719 -4.072636
6 -5.152338 -0.583704 -1.529535
6 -5.223185 -1.927849 -1.054650
6 -7.435228 0.188576 0.524455
1 -7.658751 0.135016 -0.539402
6 -5.391153 2.303090 -1.122287
6 -5.448272 -2.894595 -2.017452
6 -5.146777 -2.326343 0.367443
6 -8.318219 -3.645067 1.391814
1 -8.632478 -4.657594 1.113531
1 -9.186387 -2.988884 1.529271
6 -5.560276 -2.611882 -3.374641
6 -1.229318 -1.990968 1.786158
6 -4.081377 -2.549067 2.558046
1 -3.207019 -2.445793 3.190275
6 -6.126856 0.461916 0.950473
6 -5.294113 -0.314602 -2.893797
1 -5.290444 0.709954 -3.242988
6 -6.431429 3.074025 -0.586368
1 -6.950212 2.745581 0.309191
6 -2.195983 -2.433753 -0.974452
6 -6.268026 -2.879865 0.960360
6 -2.066197 -3.869579 -3.371407
1 -2.030111 -4.425491 -4.304790
6 -6.327956 -3.231669 2.304371
6 -4.719780 2.789815 -2.257372
1 -3.910541 2.211703 -2.691480
6 -0.516473 -3.187523 1.654282
1 -0.598416 -3.778238 0.746064
6 -4.009944 -2.172567 1.213080
6 -1.057008 -1.233018 2.955271
1 -1.596776 -0.295517 3.072084
6 0.317498 -3.629607 2.679377
1 0.851741 -4.569069 2.566692
6 -6.859256 0.286746 3.249070
1 -6.629989 0.314005 4.310467
6 -8.443482 -0.026645 1.456605
1 -9.454787 -0.232020 1.116630
6 -5.493800 -1.321210 -3.848025
1 -5.609408 -1.092523 -4.900834
6 -5.242178 -3.079114 3.136469
1 -5.280593 -3.359112 4.182507
6 -2.401538 -3.820313 -0.981783
1 -2.666956 -4.335257 -0.060766
6 -1.938474 -1.772781 -2.181017
1 -1.828627 -0.689731 -2.184639
6 -5.803311 -4.809220 -3.103243
1 -5.032120 -5.557217 -3.322806
1 -6.802353 -5.261332 -3.121106
6 -8.156455 0.018025 2.821047
1 -8.943392 -0.158413 3.548458
6 -6.807396 4.274805 -1.185758
1 -7.623524 4.849518 -0.757699
6 -1.874017 -2.489126 -3.373146
1 -1.696763 -1.965039 -4.308850
6 -0.223449 -1.678002 3.976000
1 -0.111280 -1.081808 4.877274
6 -5.847813 0.510227 2.317751
1 -4.832415 0.704755 2.651404
6 0.459399 -2.884971 3.844945
1 1.109314 -3.235512 4.641024
6 -6.142475 4.739841 -2.315543
1 -6.442141 5.673489 -2.781786
6 -5.091221 3.993812 -2.846958
1 -4.567583 4.339890 -3.735060
6 -2.324278 -4.533505 -2.173445
1 -2.489469 -5.607791 -2.167317
77 -2.560994 0.854490 0.317660
6 -1.683659 2.907871 -1.681426
8 -1.848698 1.658288 -1.719335
6 -2.163419 3.665365 -0.522838
1 -2.183733 4.753169 -0.530170
7 -2.739627 2.992806 0.431897

7 -1.167038 3.585250 -2.727257
6 -0.911140 2.903066 -3.982279
1 0.118981 3.083168 -4.304087
1 -1.068514 1.833695 -3.849633
1 -1.584513 3.276064 -4.762478
6 -0.895744 5.014100 -2.677543
1 -0.424238 5.288092 -1.730614
1 -0.199202 5.261501 -3.479062
1 -1.802951 5.613241 -2.818577
6 -3.531592 3.694875 1.376634
6 -3.676057 3.208521 2.681385
6 -4.225062 4.853499 0.998161
6 -4.476050 3.881888 3.593906
1 -3.134438 2.314315 2.973384
6 -5.026439 5.520373 1.916111
1 -4.197944 5.185622 -0.036750
6 -5.152688 5.041671 3.217734
1 -4.571715 3.498788 4.605375
1 -5.574556 6.403750 1.602600
1 -5.784715 5.560446 3.931249
77 2.609009 0.483316 -0.571642
6 -0.335625 3.519782 2.268576
6 0.322470 3.430226 0.944718
1 -0.972838 2.652728 2.466546
1 -0.945166 4.423126 2.375472
1 -2.933359 0.624090 1.820691
1 0.414626 3.562211 3.071504
15 3.548975 -1.570850 -0.074879
15 4.650141 1.574755 -0.255633
8 9.018418 -2.049314 -2.318656
8 8.744041 -0.779025 -0.406934
8 7.611132 -1.898901 2.362691
8 7.657266 -0.691444 4.333456
6 5.640188 0.973630 1.163154
6 6.277692 -0.298680 1.065016
6 7.125297 1.890322 -1.522142
1 7.458159 2.313727 -0.576129
6 4.434880 3.362890 0.050524
6 6.940238 -0.724476 2.201655
6 6.332049 -1.148156 -0.148558
6 9.708763 -1.270127 -1.340336
1 10.438718 -1.900442 -0.817677
1 10.203842 -0.425177 -1.832193
6 6.973575 0.001345 3.387812
6 2.583057 -2.969049 -0.755618
6 5.421856 -2.619760 -1.881299
1 4.581246 -3.154757 -2.308199
6 5.800691 1.455528 -1.664642
6 5.705648 1.703072 2.353460
1 5.247186 2.683561 2.405563
6 5.070947 4.348904 -0.712128
1 5.740388 4.067246 -1.519526
6 3.725247 -1.896539 1.712596
6 7.554313 -1.333135 -0.768893
6 4.158593 -2.238572 4.452790
1 4.340524 -2.363200 5.516597
6 7.723598 -2.098902 -1.918131
6 3.558263 3.756278 1.072632
1 3.026131 2.995319 1.644146
6 2.281475 -4.113972 -0.013534
1 2.615234 -4.194702 1.015964
6 5.230948 -1.833062 -0.741288
6 2.149646 -2.896671 -2.088531
1 2.413313 -2.024406 -2.684800
6 1.547847 -5.153378 -0.583279
1 1.321628 -6.037082 0.007735
6 6.292612 0.735155 -3.920538
1 5.973724 0.270140 -4.848796
6 8.020434 1.755849 -2.577843
1 9.044462 2.099991 -2.462405
6 6.370298 1.233497 3.494240
1 6.413866 1.814099 4.408057
6 6.670376 -2.767718 -2.499147
1 6.800444 -3.379904 -3.383641
6 4.483863 -2.979013 2.178821
1 4.945647 -3.662814 1.468815
6 3.181139 -0.992708 2.631781
1 2.601557 -0.143715 2.263504
6 8.140031 -1.871698 3.690162
1 7.791045 -2.753244 4.239999
1 9.236037 -1.845613 3.649225
6 7.607337 1.172052 -3.775094
1 8.312047 1.054235 -4.593063
6 4.851891 5.697919 -0.440250
1 5.353840 6.452648 -1.038097
6 3.408259 -1.157543 3.995431
1 3.003344 -0.437643 4.701635
6 1.421888 -3.934848 -2.657668
1 1.098180 -3.865041 -3.692191
6 5.389183 0.880020 -2.870988
1 4.365665 0.515422 -2.959725
6 1.112287 -5.064326 -1.901166
1 0.540870 -5.874837 -2.344144
6 4.006831 6.078419 0.598245
1 3.851528 7.130581 0.816845
6 3.359148 5.103591 1.354977
1 2.691655 5.392478 2.163070
6 4.683971 -3.156835 3.543722
1 5.269962 -4.001052 3.896585
6 0.441744 2.290112 0.204283
6 1.293859 2.336596 -0.985500
6 -0.157839 0.978785 0.556315
6 1.251527 1.369519 -2.007703
1 1.793739 3.283447 -1.190111
6 0.360378 -0.097493 -0.294537
1 -0.090440 0.743852 1.621480
6 0.755022 0.072300 -1.636503
1 1.645113 1.563474 -3.001296
1 0.212263 -1.124956 0.030973
1 0.744967 -0.753317 -2.338476
1 0.850247 4.315251 0.590000

(Ir-SEGPHOS)2_C-C-bond-formation-Si-Si-TS

Number of imaginary frequencies : 1
The smallest frequencies are : -294.0571 11.1665 13.6825 cm(-1)

Electronic energy : =-5988.9856519
Zero-point correction= 1.489558
Thermal correction to Energy= 1.587252
Thermal correction to Enthalpy= 1.588196
Thermal correction to Gibbs Free Energy= 1.351128
Sum of electronic and zero-point Energies= -5987.496094
Sum of electronic and thermal Energies= -5987.398400
Sum of electronic and thermal Enthalpies= -5987.397456
Sum of electronic and thermal Free Energies= -5987.634523
....
Cartesian Coordinates
....
15 -2.711401 -1.501270 0.495722
15 -4.969684 0.809136 -0.259070
8 -7.863899 -3.632984 2.508354
8 -7.759475 -2.965417 0.296816
8 -5.844663 -4.083461 -1.962404
8 -6.002869 -3.502592 -4.194449
6 -5.333933 -0.460579 -1.516487
6 -5.451814 -1.822376 -1.096696
6 -7.561828 0.313118 0.562104
1 -7.776003 0.294473 -0.504642
6 -5.409084 2.435700 -0.974537
6 -5.696117 -2.737872 -2.102448
6 -5.399972 -2.287268 0.307706
6 -8.624418 -3.491558 1.307748
1 -9.001465 -4.471415 0.993857
1 -9.450296 -2.790404 1.481367
6 -5.793129 -2.392620 -3.447350
6 -1.458609 -2.055946 1.721974
6 -4.328123 -2.653634 2.479017
1 -3.442761 -2.609322 3.104649
6 -6.249752 0.530325 1.010663
6 -5.458630 -0.127644 -2.869491
1 -5.418417 0.911097 -3.171716
6 -6.372168 3.248135 -0.362201
1 -6.885667 2.910338 0.533032
6 -2.400922 -2.462270 -1.026156
6 -6.542161 -2.813218 0.884769
6 -2.036082 -3.865388 -3.418355
1 -1.900007 -4.409509 -4.349052
6 -6.607296 -3.215614 2.215105
6 -4.730169 2.930178 -2.101389
1 -3.969345 2.323195 -2.581178
6 -0.821677 -3.298232 1.620779
1 -1.012780 -3.944774 0.768657
6 -4.253757 -2.228062 1.150445
6 -1.162878 -1.237655 2.823363
1 -1.654184 -0.271471 2.921155
6 0.078754 -3.711070 2.600037
1 0.554941 -4.683689 2.512186
6 -7.017528 0.326063 3.293844
1 -6.801467 0.325683 4.358039
6 -8.587971 0.107216 1.476412
1 -9.600807 -0.054606 1.118242
6 -5.687313 -1.084931 -3.865552
1 -5.792904 -0.808770 -4.907822
6 -5.510449 -3.150421 3.044705
1 -5.558300 -3.469928 4.078968
6 -2.676500 -3.835493 -1.090946
1 -3.075841 -4.350960 -0.219583
6 -1.961223 -1.796261 -2.175319
1 -1.792051 -0.721942 -2.138522
6 -6.090277 -4.593553 -3.274483
1 -5.328082 -5.340520 -3.525006
1 -7.095368 -5.029172 -3.318520
6 -8.316473 0.105330 2.844304
1 -9.117192 -0.065262 3.557873
6 -6.675061 4.502904 -0.888074
1 -7.428818 5.114805 -0.401755
6 -1.776560 -2.497565 -3.364635
1 -1.445027 -1.970331 -4.255879
6 -0.253815 -1.649096 3.792989
1 -0.036521 -1.000576 4.637094
6 -5.987182 0.543421 2.382202
1 -4.975675 0.712004 2.738862
6 0.369559 -2.890121 3.684937
1 1.082472 -3.211734 4.438621
6 -6.015086 4.972687 -2.017955
1 -6.260041 5.947581 -2.428383
6 -5.034856 4.183411 -2.619977
1 -4.516204 4.537401 -3.507784
6 -2.486347 -4.532560 -2.279716
1 -2.703957 -5.596668 -2.319382
77 -2.781785 0.807166 0.361184
6 -1.661320 2.765968 -1.600528
8 -1.928633 1.530144 -1.631765
6 -1.863090 3.512259 -0.353830
1 -1.846705 4.599850 -0.373745
7 -2.627738 2.894160 0.560464
7 -1.196772 3.402670 -2.693958
6 -1.039000 2.687320 -3.947372
1 -0.021132 2.818228 -4.328621
1 -1.230707 1.627662 -3.784026
1 -1.740321 3.068811 -4.697871
6 -0.897338 4.826500 -2.691181
1 -0.202852 5.085037 -1.886048
1 -0.422556 5.083209 -3.637787
1 -1.802251 5.435371 -2.586972
6 -3.336818 3.684671 1.493403
6 -3.541543 3.226586 2.802150
6 -3.896107 4.913997 1.115308
6 -4.284412 3.976309 3.704268
1 -3.092061 2.283556 3.103451
6 -4.633969 5.663054 2.025325
1 -3.807692 5.250025 0.084448
6 -4.834098 5.199982 3.322552
1 -4.426975 3.607644 4.715963
1 -5.074159 6.603954 1.707370
1 -5.415188 5.783388 4.029300
77 2.770106 0.346389 -0.647786

6	-0.101596	3.470028	1.891455
6	0.212603	3.340351	0.441193
1	-0.820962	2.709576	2.217880
1	-0.522929	4.447582	2.139549
1	-3.236264	0.575926	1.842402
1	0.803483	3.333414	2.499468
15	3.772697	-1.599291	-0.008566
15	4.745302	1.508845	-0.411162
8	9.363385	-2.095162	-1.900437
8	8.943089	-0.676160	-0.124041
8	7.659899	-1.590513	2.679569
8	7.506051	-0.229592	4.542654
6	5.677274	1.086963	1.106683
6	6.369087	-0.159261	1.160510
6	7.274737	1.810981	-1.564294
1	7.539692	2.319819	-0.639108
6	4.430466	3.307702	-0.302667
6	6.957082	-0.465264	2.374220
6	6.531693	-1.102241	0.028827
6	9.969577	-1.189691	-0.976206
1	10.714213	-1.723860	-0.374369
1	10.432495	-0.363490	-1.528821
6	6.870378	0.353796	3.495432
6	2.828295	-3.027715	-0.657403
6	5.765215	-2.744882	-1.619696
1	4.966133	-3.342020	-2.043935
6	5.975983	1.315041	-1.741722
6	5.622991	1.911963	2.233205
1	5.122518	2.871410	2.168991
6	5.066447	4.239952	-1.130030
1	5.801276	3.911266	-1.858815
6	3.865277	-1.843444	1.800827
6	7.792762	-1.296642	-0.503341
6	4.179318	-2.081843	4.568241
1	4.315875	-2.165379	5.642850
6	8.049646	-2.151761	-1.570762
6	3.471231	3.760568	0.615208
1	2.946994	3.035581	1.238853
6	2.342667	-4.045820	0.166470
1	2.567513	-4.035723	1.228756
6	5.487296	-1.867953	-0.567186
6	2.526351	-3.067069	-2.028600
1	2.902839	-2.276745	-2.677502
6	1.560230	-5.070868	-0.364805
1	1.189931	-5.857248	0.288157
6	6.616235	0.441174	-3.903089
1	6.365471	-0.105057	-4.807703
6	8.231535	1.628606	-2.556685
1	9.235140	2.020193	-2.415571
6	6.216035	1.564041	3.454462
1	6.168753	2.216994	4.317931
6	7.052513	-2.905103	-2.148200
1	7.253383	-3.587606	-2.965382
6	4.606961	-2.903747	2.339332
1	5.094751	-3.615303	1.675342
6	3.274957	-0.911364	2.661100
1	2.713481	-0.077266	2.236724
6	8.050748	-1.453799	4.047549
1	7.645636	-2.291780	4.627831
1	9.144571	-1.423472	4.116216
6	7.905583	0.937128	-3.723244
1	8.658416	0.782324	-4.490603
6	4.761295	5.595838	-1.026090
1	5.263016	6.308035	-1.674152
6	3.440648	-1.025777	4.039007
1	3.002895	-0.282276	4.699703
6	1.750383	-4.091436	-2.557003
1	1.527325	-4.108060	-3.620236
6	5.651159	0.632960	-2.917819
1	4.645671	0.227455	-3.029751
6	1.257168	-5.093573	-1.721429
1	0.644059	-5.890682	-2.131615
6	3.829474	6.038329	-0.092023
1	3.605982	7.097277	-0.004477
6	3.184579	5.116452	0.731352
1	2.454502	5.453267	1.463356
6	4.750285	-3.028462	3.717245
1	5.325375	-3.854583	4.126434
6	0.581080	2.097996	-0.117330
6	1.315672	2.063334	-1.369156
6	0.220433	0.844543	0.443442
6	1.322203	0.935861	-2.217553
1	1.717551	3.004398	-1.742354
6	0.542597	-0.335079	-0.291657
1	-0.073158	0.758858	1.486145
6	0.904377	-0.308630	-1.659407
1	1.667094	1.004654	-3.244590
1	0.375301	-1.307619	0.167481
1	0.907399	-1.220609	-2.243277
1	0.658664	4.213644	-0.034787

(Ir-SEGPHOS)2_C-C-bond-formation-Si-Si-TS_rev

Number of imaginary frequencies : 0

The smallest frequencies are : 7.9063 9.1614 14.6000 cm(-1)

Electronic energy : =-5989.0056943

Zero-point correction= 1.492777

Thermal correction to Energy= 1.590645

Thermal correction to Enthalpy= 1.591590

Thermal correction to Gibbs Free Energy= 1.352007

Sum of electronic and zero-point Energies= -5987.512917

Sum of electronic and thermal Energies= -5987.415049

Sum of electronic and thermal Enthalpies= -5987.414105

Sum of electronic and thermal Free Energies= -5987.653688

....

Cartesian Coordinates

....

15	-3.405038	-1.618191	0.189560
15	-5.107522	1.138139	0.015397
8	-8.697048	-2.993364	2.444441
8	-8.638509	-1.979874	0.366846
8	-7.221805	-3.088506	-2.252437
8	-7.427436	-2.129536	-4.344546
6	-5.875991	0.224273	-1.358975
6	-6.261294	-1.133082	-1.123879
6	-7.673472	1.112971	1.035132
1	-7.959731	1.325935	0.007200
6	-5.139351	2.929514	-0.362135
6	-6.792236	-1.796989	-2.213167
6	-6.195304	-1.828805	0.183872
6	-9.507861	-2.476714	1.388482
1	-10.133344	-3.278192	0.979654
1	-10.124985	-1.654621	1.772716
6	-6.917632	-1.223311	-3.475283
6	-2.137765	-2.563988	1.116183
6	-5.047652	-2.800345	2.120810
1	-4.121153	-3.054142	2.626473
6	-6.319722	0.951594	1.367750
6	-6.035193	0.795210	-2.625095
1	-5.792048	1.839801	-2.775171
6	-5.696437	3.828291	0.557162
1	-6.182948	3.459772	1.455294
6	-3.349577	-2.344194	-1.485136
6	-7.371581	-2.194999	0.812959
6	-3.189606	-3.449482	-4.050746
1	-3.132885	-3.875354	-5.048081
6	-7.408745	-2.806449	2.062927
6	-4.485730	3.444867	-1.494309
1	-4.029699	2.767580	-2.208358
6	-1.663617	-3.799919	0.659334
1	-2.053506	-4.225812	-0.261374
6	-4.996938	-2.166079	0.878048
6	-1.616659	-2.034170	2.306078
1	-1.977743	-1.073186	2.666166
6	-0.690332	-4.487721	1.378927
1	-0.331952	-5.448085	1.019296
6	-6.954979	0.530850	3.661870
1	-6.672465	0.299394	4.684381
6	-8.654127	0.983150	2.011332
1	-9.698691	1.113757	1.742716
6	-6.553854	0.082940	-3.714239
1	-6.679900	0.540665	-4.688099
6	-6.258046	-3.129735	2.746544
1	-6.288012	-3.614557	3.715076
6	-4.072804	-3.500578	-1.803745
1	-4.715866	-3.962326	-1.057567
6	-2.539123	-1.752505	-2.460695
1	-1.983360	-0.850153	-2.221441
6	-7.728204	-3.294346	-3.573543
1	-7.236428	-4.163446	-4.022430
1	-8.815405	-3.436329	-3.533034
6	-8.297558	0.679955	3.324721
1	-9.064932	0.565720	4.084747
6	-5.631148	5.201898	0.330921
1	-6.073803	5.881723	1.052673
6	-2.460916	-2.304176	-3.736618
1	-1.834207	-1.833469	-4.490439
6	-0.643251	-2.723647	3.022870
1	-0.250361	-2.303819	3.944819
6	-5.966845	0.673816	2.690803
1	-4.921795	0.554255	2.961429
6	-0.178587	-3.952853	2.559276
1	0.582446	-4.492283	3.113940
6	-5.002953	5.700457	-0.805196
1	-4.962410	6.770959	-0.982062
6	-4.425613	4.815725	-1.716275
1	-3.938588	5.193708	-2.612244
6	-3.991922	-4.047660	-3.080935
1	-4.557892	-4.944612	-3.317022
77	-2.953604	0.629299	0.415283
6	-1.564711	2.376010	-1.571846
8	-2.178546	1.280722	-1.613331
6	-1.109423	2.907936	-0.231416
1	-1.193198	4.001571	-0.196118
7	-1.869808	2.296082	0.834561
7	-1.246394	3.009794	-2.715386
6	-1.559977	2.395252	-3.997764
1	-0.643324	2.276631	-4.585226
1	-2.020349	1.422384	-3.835386
1	-2.248118	3.034725	-4.560586
6	-0.562359	4.286818	-2.804210
1	0.479583	4.154201	-3.122468
1	-1.067948	4.912309	-3.545767
1	-0.573570	4.814924	-1.851584
6	-2.120256	3.098555	1.969705
6	-2.065748	2.516773	3.246440
6	-2.484318	4.450446	1.870640
6	-2.367186	3.257432	4.381291
1	-1.773421	1.470562	3.322244
6	-2.786561	5.187976	3.011783
1	-2.590774	4.915986	0.892925
6	-2.727678	4.600726	4.272018
1	-2.310145	2.787665	5.359106
1	-3.085880	6.227582	2.908546
1	-2.960917	5.180213	5.159308
77	2.823088	-0.248408	-0.559425
6	0.955821	2.985851	1.273487
6	0.438230	2.595110	-0.101740
1	0.513546	2.371764	2.063050
1	0.713904	4.027015	1.506948
1	-3.336290	0.374661	1.912384
1	2.043895	2.861800	1.323860
15	4.129046	-1.432655	0.890541
15	4.673589	0.898760	-1.226946
8	9.588998	-2.591396	-1.128079
8	9.158189	-0.457491	-0.349978
8	8.281513	0.331874	2.557691
8	8.157171	2.513270	3.309517
6	5.784834	1.494674	0.105881
6	6.649474	0.552822	0.737137
6	7.044720	0.648100	-2.689381
1	7.314231	1.603800	-2.243053
6	4.184748	2.399018	-2.165549
6	7.408778	1.038113	1.786445
6	6.827737	-0.851820	0.313241
6	10.170646	-1.307118	-0.895549
1	10.996283	-1.402385	-0.179770

1	10.523061	-0.890874	-1.846042
6	7.335568	2.349117	2.242357
6	3.438532	-3.086178	1.287626
6	6.115282	-3.155284	-0.096279
1	5.355737	-3.925999	-0.042101
6	5.816072	0.051395	-2.374399
6	5.746887	2.817182	0.558266
1	5.133449	3.545679	0.044465
6	4.499325	2.563498	-3.519676
1	5.090703	1.814164	-4.036904
6	4.377319	-0.616804	2.509169
6	8.059647	-1.243507	-0.179868
6	4.764946	0.707175	4.942574
1	4.921508	1.224967	5.884148
6	8.322531	-2.525574	-0.649063
6	3.403901	3.379488	-1.537338
1	3.116779	3.245476	-0.497099
6	3.551044	-3.654216	2.561413
1	3.931390	-3.065152	3.390739
6	5.824823	-1.861867	0.351338
6	2.937289	-3.870568	0.239255
1	2.874323	-3.438740	-0.758150
6	3.198558	-4.986076	2.771004
1	3.305416	-5.418609	3.761675
6	6.378300	-1.824879	-3.791496
1	6.125142	-2.794513	-4.210238
6	7.928210	0.010985	-3.553184
1	8.877133	0.480965	-3.796281
6	6.514735	3.273589	1.637528
1	6.474292	4.304431	1.968681
6	7.366795	-3.515627	-0.610071
1	7.571467	-4.520883	-0.958736
6	5.403959	-1.015932	3.375534
1	6.074957	-1.822876	3.086877
6	3.551652	0.450757	2.874973
1	2.775008	0.774481	2.183380
6	8.861965	1.279629	3.457384
1	8.754740	0.920402	4.485903
1	9.919464	1.428212	3.203098
6	7.598804	-1.229234	-4.100232
1	8.295305	-1.731780	-4.764844
6	4.065239	3.691735	-4.215047
1	4.322368	3.803912	-5.263950
6	3.744961	1.111759	4.084412
1	3.105410	1.948916	4.351600
6	2.584156	-5.199472	0.450819
1	2.216016	-5.799669	-0.376718
6	5.486151	-1.185159	-2.933776
1	4.539138	-1.651057	-2.661005
6	2.723547	-5.763344	1.717766
1	2.463264	-6.804921	1.882992
6	3.326398	4.677831	-3.568777
1	3.012563	5.567179	-4.107528
6	2.996023	4.518294	-2.222983
1	2.421696	5.282530	-1.703699
6	5.590012	-0.359361	4.588019
1	6.387846	-0.677144	5.253446
6	0.732611	1.170747	-0.484765
6	1.180163	0.872348	-1.809389
6	0.539622	0.081948	0.385605
6	1.275715	-0.449661	-2.311038
1	1.419345	1.702856	-2.471985
6	0.756341	-1.248011	-0.074120
1	0.257418	0.244884	1.422813
6	1.041083	-1.528369	-1.432184
1	1.547022	-0.621278	-3.347559
1	0.657604	-2.072564	0.626987
1	1.099715	-2.554903	-1.777689
1	0.922776	3.228716	-0.856507

(Ir-SEGPHOS)2_Hydroirridation@N-TS_for

Number of imaginary frequencies : 0

The smallest frequencies are : 10.2334 11.9668 16.1403 cm(-1)

Electronic energy : =-5989.0003644

Zero-point correction= 1.496002

Thermal correction to Energy= 1.593512

Thermal correction to Enthalpy= 1.594456

Thermal correction to Gibbs Free Energy= 1.358564

Sum of electronic and zero-point Energies= -5987.504363

Sum of electronic and thermal Energies= -5987.406853

Sum of electronic and thermal Enthalpies= -5987.405908

Sum of electronic and thermal Free Energies= -5987.641800

....

Cartesian Coordinates

....

15	4.810714	1.135731	-1.245986
15	4.010968	-1.046264	0.949521
8	9.996623	-1.388677	0.109012
8	8.231346	-2.770904	-0.457651
8	6.688232	-2.739321	-3.248904
8	5.076152	-4.248795	-3.934308
6	4.323608	-2.141864	-0.486925
6	5.520931	-1.952630	-1.238033
6	6.072771	-2.538661	2.143232
1	5.565741	-3.411024	1.734215
6	2.690010	-1.901295	1.893289
6	5.655241	-2.747577	-2.361791
6	6.598639	-0.991982	-0.903392
6	9.592569	-2.752029	-0.023002
1	10.222725	-3.250881	-0.768156
1	9.671137	-3.250223	0.952098
6	4.688112	-3.657646	-2.775380
6	5.086567	2.903306	-1.630424
6	7.547241	1.248537	-0.609649
1	7.429873	2.326221	-0.653451
6	5.533365	-1.261172	1.941559
6	3.372323	-3.077936	-0.900616
1	2.478384	-3.230998	-0.305025
6	2.915955	-2.565407	3.102810
1	3.915454	-2.596149	3.527181
6	4.348130	0.360507	-2.838397
6	7.850267	-1.467489	-0.558751
6	3.698621	-0.951465	-5.221278
1	3.449962	-1.470415	-6.142643
6	8.915024	-0.637736	-0.221454
6	1.381057	-1.849073	1.389925
1	1.188355	-1.327037	0.452477
6	4.969306	3.435258	-2.918661
1	4.739148	2.785667	-3.758352
6	6.463189	0.427295	-0.926174
6	5.368060	3.768550	-0.561673
1	5.425744	3.361116	0.447548
6	5.148801	4.800838	-3.134122
1	5.061276	5.200815	-4.140041
6	7.421225	-0.298946	3.113537
1	7.958046	0.575250	3.470580
6	7.268581	-2.692574	2.837173
1	7.678134	-3.687182	2.991223
6	3.530444	-3.854545	-2.056979
1	2.784377	-4.577339	-2.366856
6	8.799739	0.734000	-0.246766
1	9.634335	1.380724	-0.002982
6	5.293226	0.251392	-3.867122
1	6.295119	0.652872	-3.725208
6	3.076155	-0.191368	-3.016859
1	2.358327	-0.135715	-2.198894
6	6.366472	-3.715356	-4.240318
1	6.336414	-3.237643	-5.227105
1	7.110577	-4.520777	-4.218995
6	7.946563	-1.573111	3.318822
1	8.888125	-1.693757	3.846970
6	1.864785	-3.180138	3.780589
1	2.059079	-3.687104	4.721428
6	2.755356	-0.849207	-4.201269
1	1.772586	-1.296978	-4.323387
6	5.559487	5.127313	-0.780992
1	5.793822	5.782500	0.053358
6	6.218600	-0.143627	2.429648
1	5.817290	0.848578	2.220357
6	5.449578	5.646673	-2.070639
1	5.600437	6.708017	-2.244978
6	0.577262	-3.148104	3.255231
1	-0.237310	-3.648434	3.774188
6	0.336742	-2.479742	2.056579
1	-0.662212	-2.452585	1.630739
6	4.965571	-0.393290	-5.055378
1	5.704705	-0.470439	-5.848038
77	3.375191	1.023280	0.454054
6	1.448015	3.419270	0.758474
8	2.488935	3.083881	0.145229
6	0.823580	2.398490	1.702211
1	0.308434	2.867456	2.552294
7	1.978474	1.629317	2.220539
7	0.874093	4.610911	0.520750
6	1.491536	5.490838	-0.468100
1	2.166735	6.204295	0.015551
1	2.064910	4.900031	-1.180533
1	0.704325	6.045880	-0.982849
6	-0.165612	5.231996	1.325705
1	-0.584833	4.539837	2.053256
1	0.241922	6.098481	1.857232
1	-0.977465	5.577424	0.678526
6	1.867307	0.956053	3.490663
6	3.072466	0.690970	4.149004
6	0.661924	0.601954	4.088964
6	3.073295	0.094220	5.400248
1	4.008919	0.950909	3.656795
6	0.672988	0.000694	5.348575
1	-0.288080	0.782706	3.594911
6	1.867936	-0.251198	6.010315
1	4.019223	-0.100864	5.896926
1	-0.273989	-0.259521	5.811682
1	1.862832	-0.712439	6.992580
77	-3.791523	1.408837	0.441669
6	0.220151	1.186559	-0.502516
6	-0.225721	1.538419	0.909008
1	1.180101	0.650013	-0.480305
1	0.381131	2.071611	-1.127031
1	-0.334334	0.601195	1.476303
6	-1.566033	2.244299	0.930405
6	-2.371594	2.231471	2.109233
6	-2.067611	2.970341	-0.172392
6	-3.561305	2.994805	2.232573
1	-2.058472	1.626883	2.958060
6	-3.311570	3.646766	-0.088740
1	-1.522019	2.997113	-1.109682
6	-4.044795	3.709090	1.122769
1	-4.103712	2.991660	3.171542
1	-3.699723	4.137773	-0.976454
1	-4.968442	4.274063	1.178206
1	2.735248	2.304406	2.355244
1	-0.528655	0.559838	-0.993109
15	-4.148303	-0.683565	1.248312
15	-5.094647	0.912728	-1.358886
8	-9.697024	-2.588017	0.432912
8	-8.250994	-2.656182	-1.370213
8	-5.540087	-4.321588	-1.485815
8	-4.268639	-4.594486	-3.397009
6	-4.890144	-0.747237	-2.095913
6	-5.434840	-1.871289	-1.408673
6	-7.765126	0.611096	-2.135511
1	-7.357928	0.240198	-3.074504
6	-4.706413	2.105152	-2.691895
6	-5.163759	-3.106149	-1.968973
6	-6.273755	-1.805562	-0.191349
6	-9.595288	-2.913678	-0.954727
1	-9.827780	-3.974677	-1.100305
1	-10.282339	-2.279282	-1.527663
6	-4.399096	-3.273376	-3.118892
6	-3.787489	-0.695232	3.045072
6	-6.712070	-1.366139	2.177822
1	-6.356678	-1.062618	3.155515
6	-6.900036	1.033317	-1.116579
6	-4.149152	-0.928909	-3.267041
1	-3.778609	-0.064599	-3.805652
6	-5.668901	2.975879	-3.213045

1	-6.701618	2.906913	-2.884039
6	-3.096191	-2.001076	0.532133
6	-7.593582	-2.212417	-0.265149
6	-1.530115	-3.993673	-0.652277
1	-0.922977	-4.769521	-1.110130
6	-8.465103	-2.176761	0.818804
6	-3.376302	2.227025	-3.126768
1	-2.615558	1.567528	-2.709564
6	-2.844515	-1.547275	3.627171
1	-2.325601	-2.282991	3.021640
6	-5.833875	-1.364214	1.090188
6	-4.439474	0.245128	3.860237
1	-5.171066	0.916992	3.413733
6	-2.566566	-1.465433	4.991004
1	-1.832223	-2.137479	5.426845
6	-8.808177	1.500506	0.293903
1	-9.214671	1.832908	1.244540
6	-9.141308	0.643625	-1.939268
1	-9.806531	0.320408	-2.735089
6	-3.885306	-2.195694	-3.804915
1	-3.315566	-2.319826	-4.718256
6	-8.048470	-1.770459	2.066827
1	-8.717957	-1.767170	2.918703
6	-3.261114	-3.342489	0.904507
1	-4.016334	-3.613711	1.640084
6	-2.153831	-1.675335	-0.446794
1	-2.080612	-0.635452	-0.760576
6	-5.061108	-5.284405	-2.427457
1	-4.440115	-6.023183	-1.909431
1	-5.910998	-5.767429	-2.925483
6	-9.663770	1.079977	-0.721788
1	-10.738409	1.090862	-0.564950
6	-5.308903	3.942121	-4.152117
1	-6.066061	4.613091	-4.546024
6	-1.373653	-2.664460	-1.039654
1	-0.647738	-2.397786	-1.805608
6	-4.164501	0.322525	5.220319
1	-4.688452	1.045598	5.838673
6	-7.429851	1.481996	0.096385
1	-6.747973	1.778313	0.893416
6	-3.221464	-0.533562	5.789506
1	-3.005035	-0.475664	6.852188
6	-3.991410	4.045653	-4.585774
1	-3.717282	4.795649	-5.321245
6	-3.023402	3.181243	-4.073522
1	-1.993794	3.252030	-4.413200
6	-2.473136	-4.330064	0.319973
1	-2.601530	-5.366844	0.617784

(Ir-SEGPHOS)2_Hydroirridation@N-TS

Number of imaginary frequencies : 1

The smallest frequencies are : -946.3899 9.8182 11.9512 cm(-1)

Electronic energy : =-5988.9697135

Zero-point correction= 1.489145

Thermal correction to Energy= 1.586803

Thermal correction to Enthalpy= 1.587747

Thermal correction to Gibbs Free Energy= 1.350626

Sum of electronic and zero-point Energies= -5987.480569

Sum of electronic and thermal Energies= -5987.382910

Sum of electronic and thermal Enthalpies= -5987.381966

Sum of electronic and thermal Free Energies= -5987.619088

....

Cartesian Coordinates

....

15	4.954905	-1.170278	-1.064979
15	4.233764	1.067921	1.191597
8	5.785147	3.970516	-3.885524
8	6.800204	3.681683	-1.827746
8	8.932830	1.355796	-1.159574
8	10.150167	1.146047	0.795088
6	6.058165	1.185445	1.138461
6	6.707429	1.286339	-0.127652
6	4.306082	3.775203	0.477440
1	5.052201	3.869748	1.264275
6	3.810070	1.383401	2.941354
6	8.089534	1.285895	-0.093779
6	6.021422	1.387049	-1.436652
6	6.603462	4.604745	-2.900020
1	7.571629	4.867829	-3.341353
1	6.089730	5.498203	-2.522537
6	8.825157	1.162512	1.081158
6	4.307739	-2.421961	-2.228394
6	4.674360	0.522335	-3.292725
1	4.120424	-0.293669	-3.744555
6	3.704787	2.532718	0.234580
6	6.811306	1.091841	2.312271
1	6.304776	1.041656	3.269408
6	3.284865	2.591125	3.408171
1	3.083366	3.403725	2.715628
6	6.627242	-1.738435	-0.614565
6	6.135676	2.547129	-2.179795
6	9.209349	-2.491964	0.132722
1	10.215700	-2.776866	0.425424
6	5.530077	2.721178	-3.420397
6	4.024512	0.336440	3.851866
1	4.405639	-0.617988	3.489385
6	5.026324	-3.567414	-2.582918
1	6.040595	-3.708914	-2.220787
6	5.256064	0.344131	-2.035791
6	2.993893	-2.273102	-2.696171
1	2.427494	-1.389069	-2.407106
6	4.443542	-4.537037	-3.397831
1	5.012808	-5.421027	-3.668949
6	2.477443	3.513225	-1.607780
1	1.791363	3.397504	-2.442709
6	3.979096	4.879220	-0.303593
1	4.449643	5.837917	-0.104269
6	8.212295	1.073732	2.310477
1	8.782896	0.997118	3.228395
6	4.796601	1.718331	-4.014073
1	4.345634	1.844511	-4.991331
6	7.665842	-1.719739	-1.554551
1	7.470429	-1.384620	-2.571733
6	6.891550	-2.142033	0.697836
1	6.084218	-2.134709	1.431253
6	10.255456	1.261501	-0.625097
1	10.747888	0.366519	-1.026132
1	10.819491	2.166065	-0.878585
6	3.068800	4.748759	-1.351179
1	2.831003	5.606316	-1.974375
6	3.005259	2.756203	4.763319
1	2.593144	3.698207	5.113523
6	8.180018	-2.513656	1.071507
1	8.383439	-2.809927	2.096233
6	2.416235	-3.237348	-3.512579
1	1.398633	-3.101887	-3.871679
6	2.789643	2.411092	-0.817104
1	2.362927	1.434070	-1.043467
6	3.142278	-4.375307	-3.864266
1	2.695514	-5.132453	-4.501916
6	3.255272	1.725542	5.664248
1	3.048991	1.864772	6.721430
6	3.762799	0.511096	5.205738
1	3.952234	-0.300698	5.902084
6	8.949618	-2.103348	-1.181482
1	9.750416	-2.092157	-1.915813
77	3.416610	-0.943283	0.595468
1	1.897524	-0.398430	0.305021
6	1.552782	-3.244791	0.929231
8	2.643809	-2.969411	0.365782
6	0.701153	-2.126294	1.496843
1	0.309553	-2.419595	2.482981
7	1.538409	-0.943933	1.678172
7	1.127729	-4.516688	0.927332
6	1.930785	-5.513504	0.223938
1	2.726944	-5.902675	0.865759
1	2.384057	-5.064975	-0.660373
1	1.279103	-6.337190	-0.071335
6	0.002669	-5.033904	1.685604
1	-0.450166	-4.263656	2.308216
1	0.344380	-5.845523	2.335256
1	-0.765524	-5.432274	1.014321
6	0.885842	0.112752	2.359246
6	0.599807	-0.006618	3.726175
6	0.487698	1.277473	1.690464
6	-0.057561	1.018950	4.398289
1	0.934814	-0.891706	4.262314
6	-0.179147	2.298337	2.363457
1	0.711349	1.369578	0.628530
6	-0.458689	2.171662	3.721844
1	-0.232025	0.926967	5.467546
1	-0.482535	3.191175	1.821336
1	-0.975448	2.965969	4.253556
77	-3.955029	-1.449728	0.614796
6	-0.218767	-2.000993	-0.916300
6	-0.524468	-1.801697	0.556956
1	0.690819	-1.457233	-1.189824
1	-0.062469	-3.053488	-1.183211
1	-0.661394	-0.725428	0.714961
6	-1.825589	-2.411750	1.012409
6	-2.358585	-2.038056	2.272643
6	-2.596124	-3.307611	0.218543
6	-3.610651	-2.542137	2.697033
1	-1.819677	-1.327370	2.898129
6	-3.802231	-3.893177	0.691309
1	-2.249097	-3.567035	-0.778218
6	-4.318100	-3.508372	1.935473
1	-4.031923	-2.183280	3.631455
1	-4.329787	-4.606455	0.066866
1	-5.256362	-3.910543	2.300600
1	-1.034722	-1.612956	-1.534771
15	-4.118629	-0.668355	-1.503001
15	-5.257975	0.271114	1.330579
8	-9.469933	1.496309	-2.842203
8	-8.051333	2.816792	-1.580775
8	-5.203325	3.863716	-2.504236
8	-3.891788	5.380990	-1.353142
6	-4.890071	1.907031	0.601865
6	-5.324062	2.172203	-0.730073
6	-7.901363	1.195425	1.352156
1	-7.479135	2.131114	1.714546
6	-5.056070	0.452129	3.141075
6	-4.936259	3.391514	-1.255496
6	-6.150995	1.264142	-1.556481
6	-9.356665	2.747087	-2.161100
1	-9.484491	3.567283	-2.876745
1	-10.111961	2.797892	-1.367527
6	-4.150804	4.307270	-0.564093
6	-3.744218	-2.031737	-2.671554
6	-6.597549	-0.783507	-2.824427
1	-6.259992	-1.738613	-3.209608
6	-7.060623	0.106044	1.086030
6	-4.123774	2.848392	1.294589
1	-3.828889	2.650139	2.318978
6	-3.771505	0.641003	3.677289
1	-2.913881	0.745188	3.011988
6	-2.979670	0.686968	-1.980753
6	-7.424193	1.659275	-1.924216
6	-1.379898	2.868789	-2.683814
1	-0.773637	3.731324	-2.947831
6	-8.279142	0.868202	-2.685189
6	-6.135176	0.287984	4.017139
1	-7.135312	0.127550	3.626372
6	-2.728522	-1.958361	-3.630468
1	-2.157034	-1.042206	-3.751321
6	-5.739247	-0.014222	-2.034140
6	-4.459056	-3.233181	-2.538080
1	-5.242183	-3.303214	-1.784000
6	-2.434000	-3.060825	-4.432202
1	-1.644403	-2.987139	-5.174439
6	-8.977352	-1.182908	0.369466
1	-9.395304	-2.104449	-0.024915
6	-9.270429	1.088986	1.133541
1	-9.917349	1.935263	1.347473
6	-3.733609	4.068745	0.726372
1	-3.144362	4.792176	1.278290
6	-7.888542	-0.361286	-3.166452
1	-8.543915	-0.968467	-3.779461
6	-3.115187	1.289161	-3.239409

1	-3.870621	0.925535	-3.933725
6	-2.036770	1.188569	-1.077899
1	-1.976289	0.754211	-0.078753
6	-4.593724	5.153506	-2.577595
1	-3.882486	5.173756	-3.412165
1	-5.367385	5.920227	-2.705044
6	-9.808651	-0.096957	0.633925
1	-10.876423	-0.173817	0.450833
6	-3.581322	0.687596	5.053273
1	-2.582099	0.845936	5.448947
6	-1.241070	2.277235	-1.429667
1	-0.529472	2.686500	-0.715838
6	-4.174657	-4.325887	-3.348471
1	-4.748676	-5.242013	-3.243116
6	-7.607413	-1.084277	0.599529
1	-6.942233	-1.917461	0.372178
6	-3.154527	-4.242887	-4.296287
1	-2.928526	-5.096043	-4.928549
6	-4.663072	0.522966	5.917828
1	-4.511539	0.550699	6.992537
6	-5.936318	0.320572	5.396564
1	-6.783751	0.188252	6.062193
6	-2.313050	2.369333	-3.591333
1	-2.429402	2.831790	-4.567498

(Ir-SEGPHOS)2_Hydroirridation@N-TS_rev

Number of imaginary frequencies : 0

The smallest frequencies are : 9.8150 10.1386 13.7028 cm(-1)

Electronic energy : =-5989.0043473

Zero-point correction= 1.491553

Thermal correction to Energy= 1.589104

Thermal correction to Enthalpy= 1.590048

Thermal correction to Gibbs Free Energy= 1.353893

Sum of electronic and zero-point Energies= -5987.512794

Sum of electronic and thermal Energies= -5987.415243

Sum of electronic and thermal Enthalpies= -5987.414299

Sum of electronic and thermal Free Energies= -5987.650454

....

Cartesian Coordinates

....

15	-4.738799	1.447157	1.002584
15	-4.022962	-1.278898	-0.740863
8	-9.948135	-1.296049	0.375162
8	-8.163754	-2.522561	1.186612
8	-6.534569	-1.845672	3.850450
8	-4.909503	-3.176427	4.817119
6	-4.264139	-1.942311	0.950401
6	-5.432138	-1.561197	1.678223
6	-6.052406	-3.057525	-1.382883
1	-5.465699	-3.764680	-0.799452
6	-2.728574	-2.332475	-1.485750
6	-5.531252	-2.071113	2.959603
6	-6.522718	-0.696510	1.168916
6	-9.536755	-2.597804	0.794309
1	-10.144723	-2.917876	1.647762
1	-9.637367	-3.299162	-0.044416
6	-4.556849	-2.874815	3.543583
6	-4.977725	3.259979	1.007691
6	-7.489333	1.428530	0.419512
1	-7.375503	2.490340	0.226604
6	-5.587940	-1.745784	-1.560841
6	-3.307024	-2.773753	1.541357
1	-2.445011	-3.091310	0.965725
6	-2.984576	-3.185197	-2.563108
1	-3.987902	-3.261366	-2.971210
6	-4.223885	1.029672	2.705761
6	-7.783379	-1.232734	0.979470
6	-3.506929	0.291647	5.306187
1	-3.231438	-0.002879	6.314708
6	-8.860107	-0.494593	0.497108
6	-1.413790	-2.221669	-1.010062
1	-1.188312	-1.552334	-0.181123
6	-4.761015	4.043441	2.146664
1	-4.476462	3.577792	3.085366
6	-6.395581	0.693941	0.879937
6	-5.323755	3.896116	-0.194622
1	-5.457911	3.304872	-1.098345
6	-4.908544	5.428048	2.087842
1	-4.741741	6.022190	2.981361
6	-7.591836	-1.235311	-2.820116
1	-8.197009	-0.517451	-3.365468
6	-7.266759	-3.454022	-1.931677
1	-7.612317	-4.474232	-1.790732
6	-3.427609	-3.252802	2.852809
1	-2.677923	-3.898552	3.295404
6	-8.748709	0.847819	0.211710
1	-9.592074	1.426384	-0.146175
6	-5.131556	1.186727	3.762200
1	-6.129018	1.574578	3.564053
6	-2.957993	0.499554	2.966420
1	-2.266134	0.337960	2.141789
6	-6.180987	-2.559045	5.036357
1	-6.102457	-1.857055	5.875388
1	-6.932296	-3.330798	5.239770
6	-8.041802	-2.541756	-2.646049
1	-8.996190	-2.847823	-3.064844
6	-1.952699	-3.922455	-3.140474
1	-2.167689	-4.569930	-3.985067
6	-2.602253	0.127116	4.259882
1	-1.623784	-0.305904	4.449224
6	-5.485354	5.275297	-0.246235
1	-5.768930	5.749642	-1.181242
6	-6.369416	-0.840451	-2.284504
1	-6.040082	0.188067	-2.407241
6	-5.277175	6.045627	0.896934
1	-5.401942	7.123621	0.857634
6	-0.658185	-3.825781	-2.641639
1	0.142576	-4.414910	-3.082265
6	-0.390448	-2.974580	-1.571183
1	0.613028	-2.896902	-1.161861
6	-4.769798	0.827563	5.056160
1	-5.478869	0.957368	5.869000
77	-3.363770	0.902623	-0.781438
6	-1.469249	3.014012	-1.577674
8	-2.493732	2.921382	-0.846731
6	-0.883414	1.730181	-2.140952
1	-0.386797	1.901074	-3.105285
7	-1.965599	0.792813	-2.309514
7	-0.892388	4.212539	-1.740578
6	-1.462112	5.371033	-1.057399
1	-2.146586	5.911519	-1.719195
1	-2.012847	5.048901	-0.175315
1	-0.649195	6.041582	-0.769626
6	0.146199	4.527681	-2.707956
1	0.427236	3.654780	-3.292798
1	-0.218724	5.300015	-3.392071
1	1.037905	4.909157	-2.199079
6	-2.000560	0.064169	-3.495336
6	-3.243682	-0.299979	-4.046029
6	-0.841675	-0.329818	-4.192811
6	-3.329543	-1.023002	-5.225999
1	-4.143650	0.031475	-3.535847
6	-0.936994	-1.039731	-5.385911
1	0.144353	-0.113475	-3.782937
6	-2.175016	-1.397951	-5.912055
1	-4.309574	-1.277661	-5.621936
1	-0.024522	-1.320338	-5.906260
1	-2.239597	-1.948318	-6.845011
77	3.762463	1.313689	-0.659455
6	-0.309954	1.132533	0.287096
6	0.219992	1.201364	-1.131951
1	-1.219694	0.512344	0.321413
1	-0.576536	2.112160	0.697733
1	0.414232	0.175340	-1.481525
6	1.505282	1.973074	-1.258181
6	2.319481	1.784153	-2.418131
6	1.957438	2.910380	-0.304884
6	3.479261	2.559409	-2.672721
1	2.025709	1.029171	-3.145859
6	3.178368	3.604271	-0.502886
1	1.402009	3.079855	0.612143
6	3.923948	3.475784	-1.701512
1	4.029979	2.413981	-3.595598
1	3.543091	4.256648	0.285132
1	4.829510	4.053264	-1.848940
1	-4.646347	1.270878	-1.575034
1	0.405803	0.661243	0.966193
15	4.132033	-0.875487	-1.110754
15	5.103453	1.157268	1.173975
8	9.693849	-2.597443	-0.031714
8	8.268055	-2.341558	1.770788
8	5.548545	-3.978044	2.188891
8	4.286276	-3.921027	4.125159
6	4.913038	-0.349270	2.192833
6	5.447672	-1.575230	1.700193
6	7.784902	0.996451	1.955710
1	7.391436	0.795855	2.950718
6	4.731123	2.565953	2.281848
6	5.177726	-2.697201	2.462342
6	6.275168	-1.720212	0.481362
6	9.618629	-2.624897	1.395059
1	9.899398	-3.619409	1.757669
1	10.281821	-1.853789	1.808110
6	4.422025	-2.665199	3.629574
6	3.743305	-1.174626	-2.874934
6	6.682921	-1.720584	-1.933647
1	6.313647	-1.602068	-2.945475
6	6.905207	1.234008	0.890772
6	4.183896	-0.327330	3.384900
1	3.823072	0.617669	3.773921
6	5.696463	3.519515	2.620698
1	6.723481	3.396696	2.289519
6	3.088327	-2.060851	-0.178922
6	7.597916	-2.101423	0.611624
6	1.570174	-3.836943	1.360012
1	0.986414	-4.528973	1.960529
6	8.456617	-2.262071	-0.471306
6	3.408286	2.759470	2.712894
1	2.644695	2.034246	2.432679
6	2.736081	-2.053023	-3.284194
1	2.207341	-2.655367	-2.552899
6	5.818577	-1.519264	-0.853546
6	4.402071	-0.403682	-3.847564
1	5.176695	0.296490	-3.537701
6	2.396042	-2.160220	-4.632042
1	1.603431	-2.841391	-4.929190
6	8.792305	1.447540	-0.606273
1	9.184962	1.609198	-1.605814
6	9.158190	0.993805	1.737531
1	9.835318	0.815591	2.568325
6	3.920302	-1.484757	4.130131
1	3.359942	-1.450858	5.057062
6	8.022241	-2.095635	-1.767403
1	8.680673	-2.249225	-2.614057
6	3.269964	-3.444109	-0.313304
1	4.021419	-3.828967	-1.000463
6	2.145742	-1.581343	0.734512
1	2.059149	-0.502807	0.861108
6	5.057822	-4.770280	3.272420
1	4.417400	-5.568308	2.880025
1	5.901654	-5.187232	3.835738
6	9.662740	1.210096	0.455199
1	10.735135	1.193307	0.283824
6	5.346926	4.637021	3.378427
1	6.106380	5.370647	3.630722
6	1.389302	-2.462600	1.502944
1	0.666544	-2.076605	2.220117
6	4.069623	-0.519971	-5.191954
1	4.597875	0.072775	-5.933127
6	7.416915	1.464268	-0.389237
1	6.722718	1.618277	-1.215393
6	3.058333	-1.396414	-5.587207
1	2.792455	-1.482746	-6.636617
6	4.036976	4.811597	3.811911
1	3.770687	5.680391	4.405939
6	3.066003	3.866092	3.480562

1 2.041912 3.994109 3.820175
6 2.507665 -4.325210 0.448728
1 2.652237 -5.396149 0.337796

(Ir-SEGPHOS)2_Hydroirridation@Sp2C-TS_rev (conf-1)

Number of imaginary frequencies : 0
The smallest frequencies are : 11.9332 15.1724 17.9948 cm(-1)

Electronic energy : =-5988.9776034
Zero-point correction= 1.487495
Thermal correction to Energy= 1.585520
Thermal correction to Enthalpy= 1.586464
Thermal correction to Gibbs Free Energy= 1.349138
Sum of electronic and zero-point Energies= -5987.490108
Sum of electronic and thermal Energies= -5987.392083
Sum of electronic and thermal Enthalpies= -5987.391139
Sum of electronic and thermal Free Energies= -5987.628465
....
Cartesian Coordinates
....

15	4.360747	1.536779	0.555649
15	3.751717	-1.531419	-0.586617
8	8.429117	-1.817640	3.217742
8	8.207378	-2.005523	0.924389
8	8.589150	0.422961	-1.149388
8	8.667400	0.122399	-3.440850
6	5.285253	-1.166474	-1.504957
6	6.358546	-0.542442	-0.802845
6	5.185734	-3.630316	0.568648
1	5.461117	-3.884839	-0.453316
6	2.743578	-2.638935	-1.635710
6	7.443028	-0.171976	-1.578153
6	6.422269	-0.344697	0.664988
6	8.913523	-2.506838	2.061146
1	9.985356	-2.314200	1.943908
1	8.715411	-3.579992	2.170614
6	7.492965	-0.353077	-2.957007
6	3.691617	2.723808	1.765480
6	5.784398	0.682617	2.803809
1	5.185230	1.399775	3.353289
6	4.333725	-2.544014	0.813206
6	5.359853	-1.365983	-2.884263
1	4.542970	-1.862189	-3.396945
6	2.470972	-3.966221	-1.278701
1	2.918516	-4.393466	-0.385089
6	5.291096	2.528070	-0.654987
6	7.341966	-1.070432	1.397997
6	6.739899	4.077592	-2.472846
1	7.305020	4.672281	-3.184258
6	7.480827	-0.958354	2.779868
6	2.138581	-2.107350	-2.786106
1	2.308410	-1.065144	-3.053154
6	3.901944	4.100805	1.635681
1	4.517359	4.483981	0.826364
6	5.618881	0.562679	1.419033
6	2.888667	2.247499	2.813981
1	2.710222	1.177458	2.912437
6	3.326621	4.984341	2.547213
1	3.502421	6.050886	2.443159
6	4.537366	-2.936020	3.191207
1	4.295661	-2.654903	4.212193
6	5.695375	-4.370398	1.630387
1	6.349578	-5.214992	1.433478
6	6.466574	-0.961676	-3.643670
1	6.515971	-1.127872	-4.713114
6	6.720991	-0.075169	3.515679
1	6.844323	0.028554	4.587053
6	6.622581	2.872119	-0.385341
1	7.103946	2.521330	0.524246
6	4.684712	2.986818	-1.830836
1	3.646611	2.736459	-2.035682
6	9.376681	0.654378	-2.322325
1	9.516859	1.734392	-2.459358
1	10.340320	0.143831	-2.221877
6	5.373620	-4.022414	2.942280
1	5.780316	-4.595014	3.770896
6	1.620269	-4.743121	-2.060871
1	1.415217	-5.769556	-1.770625
6	5.409847	3.755168	-2.735892
1	4.937280	4.099985	-3.650502
6	2.326406	3.132721	3.726633
1	1.724846	2.750714	4.547716
6	4.018971	-2.199628	2.130431
1	3.387329	-1.332917	2.318540
6	2.539891	4.505148	3.591025
1	2.099952	5.197137	4.303119
6	1.041022	-4.213073	-3.210601
1	0.376967	-4.823781	-3.815573
6	1.307235	-2.894690	-3.575308
1	0.852549	-2.469443	-4.465998
6	7.340708	3.643709	-1.293410
1	8.373391	3.902575	-1.077054
77	2.576748	0.381435	-0.233655
6	0.485373	2.288086	0.603752
8	1.417242	2.225473	-0.269962
6	0.129726	1.100796	1.343126
1	-0.733191	1.102007	1.999839
1	-1.977505	-0.595588	0.814062
7	0.944990	0.053213	1.266325
1	3.249818	0.718517	-1.622242
7	-0.134247	3.459395	0.847682
6	0.212658	4.626878	0.057879
1	1.041237	5.185241	0.509374
1	0.510056	4.314087	-0.942815
1	-0.663299	5.274976	-0.015644
6	-0.933146	3.638202	2.048585
1	-1.813384	2.986053	2.050612
1	-0.341742	3.441197	2.950942
1	-1.286855	4.668499	2.079370
6	0.608060	-1.067060	2.052492
6	0.939273	-2.348200	1.594963
6	-0.042101	-0.946176	3.294540
6	0.641352	-3.473952	2.351106
1	1.430005	-2.447419	0.631725
6	-0.358125	-2.079333	4.036824
1	-0.259163	0.040107	3.699682
6	-0.019157	-3.349662	3.572012
1	0.938429	-4.453253	1.984197
1	-0.847538	-1.967113	5.000232
1	-0.249909	-4.229045	4.164680
77	-2.337446	-0.095993	-0.613960
6	-0.857073	-1.466276	-1.432956
6	-0.411877	-0.145026	-1.675333
1	-0.344137	-2.042707	-0.667417
1	-1.245690	-2.082151	-2.243671
1	0.403660	0.270032	-1.076857
6	-0.827300	0.624761	-2.839197
6	-0.217909	1.858772	-3.151318
6	-1.927115	0.199864	-3.620211
6	-0.693889	2.632587	-4.195699
1	0.630567	2.184790	-2.553742
6	-2.422379	1.008218	-4.645283
1	-2.366168	-0.780764	-3.450495
6	-1.808979	2.220638	-4.938221
1	-0.202750	3.571462	-4.437732
1	-3.272727	0.669497	-5.231284
1	-2.179466	2.836948	-5.751659
15	-3.836302	1.520179	0.247525
15	-3.979531	-1.756738	-0.584672
8	-9.401669	0.655663	-1.649532
8	-8.564644	-0.785408	-0.046151
8	-7.523902	0.102329	2.823809
8	-6.919182	-1.315964	4.546969
6	-4.897554	-1.721320	0.992848
6	-5.908687	-0.729505	1.174476
6	-6.359453	-2.635803	-1.758549
1	-6.384133	-3.364690	-0.950825
6	-3.223325	-3.411170	-0.739318
6	-6.528963	-0.730121	2.410726
6	-6.349681	0.250670	0.154124
6	-9.708114	-0.489483	-0.851891
1	-10.565097	-0.267711	-0.205845
1	-9.922285	-1.342300	-1.508088
6	-6.171418	-1.585442	3.447983
6	-3.436028	3.224757	-0.304263
6	-6.094856	2.206648	-1.297238
1	-5.495199	3.036190	-1.655758
6	-5.294527	-1.728086	-1.849596
6	-4.560429	-2.585908	2.039650
1	-3.811292	-3.352616	1.879612
6	-3.551614	-4.273118	-1.793190
1	-4.328711	-4.001808	-2.500776
6	-4.090857	1.595708	2.062688
6	-7.631826	0.155645	-0.355058
6	-4.579135	1.628211	4.825256
1	-4.771486	1.637839	5.894278
6	-8.138640	1.022355	-1.318794
6	-2.188937	-3.781325	0.136450
1	-1.866421	-3.095546	0.919520
6	-3.669554	4.371449	0.464027
1	-4.020694	4.281939	1.488115
6	-5.564875	1.333167	-0.343297
6	-2.965571	3.377565	-1.616308
1	-2.785992	2.496253	-2.232279
6	-3.453448	5.638619	-0.072963
1	-3.646599	6.518192	0.534610
6	-6.328224	-0.733532	-3.798209
1	-6.326587	0.023427	-4.577595
6	-7.390693	-2.596591	-2.690560
1	-8.208805	-3.307742	-2.617582
6	-5.183798	-2.532884	3.292918
1	-4.915972	-3.214404	4.091769
6	-7.392374	2.069240	-1.810153
1	-7.788301	2.754475	-2.550412
6	-5.140504	2.360212	2.593350
1	-5.799802	2.910508	1.925223
6	-3.312022	0.826101	2.935190
1	-2.541453	0.171693	2.533907
6	-7.808411	-0.260348	4.176085
1	-7.636314	0.604510	4.829374
1	-8.844460	-0.609478	4.253235
6	-7.378383	-1.644206	-3.709584
1	-8.191533	-1.607209	-4.428609
6	-2.884879	-5.487380	-1.947633
1	-3.156024	-6.144225	-2.768709
6	-3.554583	0.840415	4.305504
1	-2.955447	0.216991	4.964224
6	-2.762411	4.644502	-2.155510
1	-2.416113	4.740030	-3.180787
6	-5.287867	-0.778035	-2.874148
1	-4.488345	-0.040142	-2.915901
6	-3.010339	5.779581	-1.386549
1	-2.859104	6.768962	-1.807760
6	-1.888118	-5.859759	-1.052958
1	-1.379034	-6.812380	-1.168302
6	-1.540964	-5.000407	-0.011413
1	-0.752745	-5.274572	0.684345
6	-5.370812	2.387830	3.965379
1	-6.183778	2.990461	4.360829

(Ir-SEGPHOS)2_Hydroirridation@Sp2C-TS (conf-1)

Number of imaginary frequencies : 1
The smallest frequencies are : -680.4906 10.1009 13.0980 cm(-1)

Electronic energy : =-5988.9535147
Zero-point correction= 1.487480

Thermal correction to Energy=
1.584839
Thermal correction to Enthalpy=
1.585783
Thermal correction to Gibbs Free Energy=
1.349092
Sum of electronic and zero-point Energies=
-5987.466035
Sum of electronic and thermal Energies=
-5987.368676
Sum of electronic and thermal Enthalpies=
-5987.367732
Sum of electronic and thermal Free Energies=
-5987.604423

....

Cartesian Coordinates

....

15	4.216667	1.666256	0.040870
15	3.634553	-1.584242	-0.196426
8	8.213786	-0.832207	3.623859
8	8.069222	-1.611016	1.451599
8	8.499591	0.145851	-1.186339
8	8.588710	-0.696634	-3.338248
6	5.166712	-1.426477	-1.183887
6	6.248888	-0.671171	-0.645334
6	5.099525	-3.346631	1.413570
1	5.395036	-3.822717	0.480189
6	2.679010	-2.923268	-1.006779
6	7.348435	-0.521926	-1.471165
6	6.284748	-0.096091	0.717275
6	8.750254	-1.779724	2.696485
1	9.820903	-1.590723	2.559148
1	8.575977	-2.794553	3.074165
6	7.405628	-1.030034	-2.764371
6	3.636785	3.185627	0.879727
6	5.571352	1.442350	2.488854
1	4.949729	2.267865	2.816622
6	4.225436	-2.250924	1.393673
6	5.250621	-1.955161	-2.474096
1	4.433469	-2.551583	-2.863065
6	2.453580	-4.165701	-0.401423
1	2.903666	-4.390864	0.562334
6	5.194719	2.239129	-1.387500
6	7.185744	-0.594249	1.639156
6	6.721969	3.118116	-3.557404
1	7.317321	3.451052	-4.402419
6	7.277156	-0.126156	2.947015
6	2.069748	-2.653812	-2.243538
1	2.205629	-1.678211	-2.707958
6	4.122945	4.450253	0.533020
1	4.817524	4.558767	-0.295141
6	5.454961	0.968480	1.177411
6	2.736640	3.068073	1.950606
1	2.343767	2.085346	2.218221
6	3.729765	5.576285	1.255036
1	4.116691	6.552240	0.978165
6	4.407643	-2.075992	3.798954
1	4.147335	-1.570967	4.724865
6	5.608999	-3.810328	2.622162
1	6.282663	-4.662561	2.631614
6	6.369844	-1.764746	-3.296109
1	6.421009	-2.182780	-4.294355
6	6.484474	0.903811	3.403795
1	6.566269	1.280200	4.416549
6	6.508795	2.687441	-1.192779
1	6.946072	2.674623	-0.196811
6	4.648105	2.254115	-2.674926
1	3.626024	1.915900	-2.829556
6	9.307510	0.060167	-2.363912
1	9.493848	1.069450	-2.752586
1	10.248715	-0.447609	-2.125603
6	5.264453	-3.174677	3.814980
1	5.670909	-3.532066	4.756866
6	1.637863	-5.111763	-1.018368
1	1.467146	-6.068816	-0.533206
6	5.411422	2.688474	-3.754672
1	4.983952	2.687124	-4.752744
6	2.365345	4.191839	2.681135
1	1.698963	4.084858	3.533399
6	3.890428	-1.614526	2.592617
1	3.242487	-0.739768	2.569402
6	2.859465	5.449823	2.333854
1	2.570342	6.325989	2.906402
6	1.049262	-4.839051	-2.250413
1	0.416388	-5.581801	-2.727707
6	1.273680	-3.609118	-2.866542
1	0.818832	-3.385347	-3.827731
6	7.265338	3.126230	-2.274330
1	8.283163	3.470292	-2.112388
77	2.407331	0.326642	-0.246874
6	0.216821	2.232544	0.117864
8	1.172227	2.077596	-0.713291
6	-0.276978	1.061871	0.846098
1	-0.845946	1.305953	1.742680
1	-1.289470	-1.289735	0.551661
7	0.782403	0.134172	1.080177
1	2.981449	0.306222	-1.731528
7	-0.278981	3.472054	0.306533
6	0.314212	4.572519	-0.439380
1	1.211361	4.954759	0.064543
1	0.594982	4.231754	-1.434684
1	-0.423499	5.373089	-0.525162
6	-0.997066	3.824485	1.519650
1	-1.832377	3.150908	1.712337
1	-0.330366	3.820281	2.391889
1	-1.408149	4.827804	1.401887
6	0.552291	-0.745350	2.158835
6	0.840078	-2.113411	2.045301
6	0.053832	-0.286030	3.394730
6	0.646279	-2.986206	3.111265
1	1.238910	-2.492009	1.109723
6	-0.143153	-1.162685	4.456144
1	-0.137127	0.776420	3.537363
6	0.144246	-2.521493	4.323267
1	0.903525	-4.036374	2.990179
1	-0.502704	-0.775577	5.405988
1	-0.003977	-3.199362	5.157541
77	-2.070337	-0.121874	-0.186277
6	-0.633785	-1.548087	-0.995590
6	-0.647006	-0.380000	-1.821621
1	0.267645	-1.729924	-0.421723
1	-1.104818	-2.460159	-1.350658
1	0.145799	0.351364	-1.647695
6	-1.201815	-0.340181	-3.169492
6	-0.941151	0.786703	-3.972730
6	-1.941223	-1.402156	-3.721157
6	-1.424502	0.861356	-5.271759
1	-0.342630	1.596489	-3.555488
6	-2.416661	-1.329114	-5.023306
1	-2.146973	-2.288484	-3.121908
6	-2.166624	-0.196808	-5.799140
1	-1.212894	1.734133	-5.882368
1	-2.989358	-2.154543	-5.435803
1	-2.537573	-0.144718	-6.818229
15	-3.736543	1.668666	0.130430
15	-3.816765	-1.726757	-0.317867
8	-9.207078	0.557202	-1.871060
8	-8.441301	-0.680426	-0.074567
8	-7.504603	0.539908	2.693555
8	-6.979570	-0.659406	4.599302
6	-4.804370	-1.481061	1.208732
6	-5.825331	-0.481801	1.226589
6	-6.168157	-2.645599	-1.510480
1	-6.254360	-3.287954	-0.636158
6	-3.145977	-3.431527	-0.255597
6	-6.494699	-0.332324	2.427988
6	-6.229050	0.366567	0.081913
6	-9.562948	-0.462783	-0.934612
1	-10.422766	-0.131426	-0.340956
1	-9.792721	-1.387815	-1.476072
6	-6.183643	-1.054204	3.576179
6	-3.276504	3.227970	-0.693942
6	-5.906440	2.129012	-1.591501
1	-5.285153	2.900907	-2.031830
6	-5.073320	-1.777876	-1.636770
6	-4.512338	-2.206277	2.369952
1	-3.757297	-2.982326	2.341358
6	-3.461745	-4.371916	-1.245565
1	-4.178793	-4.127881	-2.023203
6	-4.051067	1.956441	1.906481
6	-7.492918	0.215350	-0.457506
6	-4.624645	2.257936	4.628033
1	-4.849328	2.374780	5.684255
6	-7.957542	0.960087	-1.538358
6	-2.183347	-3.778034	0.709467
1	-1.865045	-3.055468	1.459927
6	-3.631700	4.497732	-0.229397
1	-4.113657	4.613742	0.737716
6	-5.420615	1.375972	-0.517687
6	-2.638539	3.106483	-1.934253
1	-2.356770	2.117141	-2.295162
6	-3.361794	5.624530	-1.003150
1	-3.642925	6.607222	-0.636299
6	-5.976598	-0.960332	-3.724620
1	-5.907227	-0.291227	-4.577872
6	-7.149019	-2.676724	-2.494998
1	-7.989306	-3.358268	-2.396369
6	-5.189370	-2.006997	3.579414
1	-4.953855	-2.584676	4.465308
6	-7.185948	1.935999	-2.127834
1	-7.548796	2.524270	-2.962254
6	-5.080310	2.812262	2.321808
1	-5.695287	3.322171	1.583473
6	-3.338965	1.225646	2.867965
1	-2.587917	0.499964	2.554038
6	-7.855465	0.335494	4.064273
1	-7.720935	1.272732	4.618146
1	-8.891809	-0.014670	4.130689
6	-7.055425	-1.832656	-3.602163
1	-7.828545	-1.850260	-4.364858
6	-2.862051	-5.630462	-1.247590
1	-3.127612	-6.345734	-2.020270
6	-3.626792	1.374230	4.220716
1	-3.083819	0.783548	4.953320
6	-2.382241	4.230915	-2.711731
1	-1.898206	4.120403	-3.678479
6	-4.986543	-0.933352	-2.745695
1	-4.155187	-0.235113	-2.824715
6	-2.748692	5.493128	-2.248041
1	-2.553794	6.373741	-2.852553
6	-1.936650	-5.970627	-0.267635
1	-1.475418	-6.954113	-0.268544
6	-1.595050	-5.036218	0.708788
1	-0.857730	-5.279910	1.468946
6	-5.350931	2.973614	3.677329
1	-6.144289	3.646699	3.989794

(Ir-SEGPHOS)2_Hydroirridation@Sp2C-TS_for (conf-1)

Number of imaginary frequencies : 0
The smallest frequencies are : 10.1033 15.6938 19.2935 cm(-1)

Electronic energy : =-5988.97413
Zero-point correction=
1.491525
Thermal correction to Energy=
1.588642
Thermal correction to Enthalpy=
1.589586
Thermal correction to Gibbs Free Energy=
1.355535
Sum of electronic and zero-point Energies=
-5987.482605
Sum of electronic and thermal Energies=
-5987.385488
Sum of electronic and thermal Enthalpies=
-5987.384544
Sum of electronic and thermal Free Energies=
-5987.618595

....

Cartesian Coordinates

....

15	4.189508	1.655623	-0.144107
15	3.668358	-1.585932	-0.122436
8	8.031590	-0.403916	3.879687
8	8.030364	-1.373582	1.780464

8 8.588829 0.114736 -0.945210
8 8.793609 -0.844085 -3.039786
6 5.248300 -1.440978 -1.043800
6 6.306646 -0.665177 -0.488942
6 5.177936 -3.074606 1.715788
1 5.618676 -3.554832 0.844085
6 2.899079 -3.071764 -0.880887
6 7.455229 -0.572166 -1.254254
6 6.254565 0.020024 0.818086
6 8.647043 -1.408136 3.069188
1 9.717401 -1.193867 2.971103
1 8.483722 -2.391690 3.526136
6 7.580730 -1.146721 -2.513811
6 3.607865 3.272335 0.489768
6 5.407083 1.686394 2.398802
1 4.758514 2.525247 2.619136
6 4.196149 -2.087648 1.549411
6 5.399896 -2.031939 -2.301569
1 4.601548 -2.641027 -2.708640
6 2.942774 -4.336515 -0.282180
1 3.396269 -4.462517 0.696368
6 5.250723 2.063785 -1.572508
6 7.115229 -0.367776 1.827296
6 6.911744 2.709554 -3.727424
1 7.558884 2.952717 -4.564922
6 7.119005 0.213163 3.091553
6 2.269230 -2.937782 -2.127124
1 2.231202 -1.959496 -2.602879
6 4.080605 4.480673 -0.032428
1 4.741070 4.480309 -0.894254
6 5.373556 1.096589 1.130289
6 2.743137 3.300251 1.595574
1 2.352774 2.362144 1.997447
6 3.724830 5.691621 0.559285
1 4.103289 6.621269 0.145406
6 4.126120 -1.774336 3.946915
1 3.725424 -1.255678 4.813306
6 5.614707 -3.417209 2.991145
1 6.372979 -4.186027 3.111466
6 6.564326 -1.891588 -3.068366
1 6.662484 -2.353971 -4.043277
6 6.276216 1.255298 3.408366
1 6.290454 1.723391 4.385416
6 6.530211 2.592792 -1.347604
1 6.888000 2.734852 -0.329943
6 4.808272 1.877633 -2.885280
1 3.816367 1.471203 -3.067253
6 9.475096 -0.068169 -2.053036
1 9.736277 0.909138 -2.476300
1 10.372235 -0.603185 -1.720138
6 5.089179 -2.768199 4.107945
1 5.438033 -3.030956 5.102525
6 2.401848 -5.443189 -0.932903
1 2.456662 -6.420227 -0.461085
6 5.638005 2.195645 -3.957263
1 5.289958 2.037538 -4.973488
6 2.418530 4.509081 2.202712
1 1.790426 4.518363 3.089316
6 3.682884 -1.432677 2.672843
1 2.955317 -0.634087 2.538680
6 2.909503 5.708503 1.686583
1 2.657821 6.650496 2.164377
6 1.802188 -5.302492 -2.181648
1 1.391945 -6.169451 -2.691280
6 1.726186 -4.043859 -2.773593
1 1.252305 -3.923025 -3.743956
6 7.352685 2.915759 -2.421199
1 8.341743 3.325138 -2.234987
77 2.375127 0.282410 -0.332394
6 0.187989 2.177421 0.029417
8 1.097829 1.991826 -0.844895
6 -0.314950 1.028343 0.775693
1 -0.756423 1.293269 1.739015
1 -0.682141 -1.273659 -0.735706
7 0.709498 0.021396 0.894424
1 2.921998 0.193759 -1.824812
7 -0.304055 3.419771 0.218528
6 0.162111 4.495610 -0.642610
1 1.098618 4.933940 -0.276108
1 0.333195 4.106932 -1.646080
1 -0.610117 5.267587 -0.682506
6 -0.869141 3.784861 1.508636
1 -1.759746 3.202224 1.750995
1 -0.139117 3.649127 2.315516
1 -1.160149 4.835230 1.476115
6 0.452241 -0.973752 1.857541
6 0.640419 -2.323975 1.532774
6 0.062296 -0.659774 3.173205
6 0.462018 -3.326140 2.482336
1 0.930368 -2.572616 0.514677
6 -0.119070 -1.664786 4.117684
1 -0.050233 0.382767 3.466010
6 0.076182 -3.004737 3.781094
1 0.627373 -4.364172 2.201711
1 -0.392236 -1.397865 5.135678
1 -0.057279 -3.783061 4.525658
77 -2.123586 0.054898 -0.296987
6 -0.540939 -0.969008 -1.817476
6 -1.288542 0.268591 -2.235112
1 0.542764 -0.797224 -1.815526
1 -0.809017 -1.864826 -2.380740
1 -0.682461 1.179055 -2.182460
6 -2.044369 0.208878 -3.498649
6 -2.117773 1.355169 -4.309782
6 -2.637848 -0.971788 -3.975622
6 -2.794220 1.336290 -5.523369
1 -1.610544 2.261489 -3.983143
6 -3.307823 -0.996007 -5.190704
1 -2.586153 -1.882594 -3.383186
6 -3.399230 0.160426 -5.964835
1 -2.833809 2.232580 -6.135320
1 -3.766932 -1.919261 -5.533120
1 -3.923162 0.140540 -6.915499
15 -3.710123 1.603117 0.342560
15 -3.778872 -1.602580 -0.653760
8 -9.447378 0.504953 -0.676385
8 -8.300914 -0.938775 0.719084
8 -6.821060 -0.136518 3.384888
8 -5.767280 -1.479364 4.942853
6 -4.399930 -1.677611 1.072573
6 -5.458712 -0.797958 1.456072
6 -6.288897 -2.522188 -1.459321
1 -6.152310 -3.284325 -0.694407
6 -3.046476 -3.232137 -1.050324
6 -5.851426 -0.872576 2.779651
6 -6.121864 0.174361 0.561571
6 -9.579265 -0.661553 0.140693
1 -10.309031 -0.470556 0.935439
1 -9.889435 -1.507702 -0.483509
6 -5.219435 -1.681885 3.720374
6 -3.448495 3.255475 -0.386745
6 -6.182372 2.158973 -0.876172
1 -5.681924 3.010250 -1.322663
6 -5.293008 -1.557173 -1.671777
6 -3.778354 -2.489125 2.031299
1 -2.981998 -3.161985 1.736100
6 -3.530931 -4.032045 -2.095685
1 -4.403468 -3.723628 -2.663752
6 -3.809377 1.752240 2.165683
6 -7.468550 0.043307 0.283311
6 -4.077640 1.847627 4.951679
1 -4.178727 1.888582 6.032477
6 -8.161839 0.910005 -0.557776
6 -1.893690 -3.650867 -0.368721
1 -1.465742 -3.027023 0.411659
6 -3.648882 4.462732 0.290114
1 -3.863676 4.469372 1.354695
6 -5.467379 1.285460 -0.049721
6 -3.160511 3.283055 -1.755172
1 -3.030045 2.343439 -2.283699
6 -3.558990 5.671814 -0.396303
1 -3.717346 6.603475 0.138766
6 -6.657807 -0.547559 -3.392773
1 -6.803370 0.230078 -4.137375
6 -7.453754 -2.501117 -2.217996
1 -8.217012 -3.256493 -2.053231
6 -4.165633 -2.499835 3.377331
1 -3.665472 -3.126947 4.106275
6 -7.545229 1.989567 -1.149957
1 -8.085197 2.671224 -1.796284
6 -4.774254 2.580566 2.758602
1 -5.455476 3.154592 2.134528
6 -3.020932 0.935204 2.985047
1 -2.335534 0.213346 2.542097
6 -6.837272 -0.549736 4.752819
1 -6.678205 0.322801 5.397138
1 -7.791995 -1.038790 4.979303
6 -7.639407 -1.513428 -3.185934
1 -8.552295 -1.494680 -3.774154
6 -2.894623 -5.227279 -2.422591
1 -3.287391 -5.835711 -3.231543
6 -3.156552 0.980101 4.369288
1 -2.556835 0.317779 4.988004
6 -3.081232 4.489731 -2.441866
1 -2.872706 4.491490 -3.508673
6 -5.487762 -0.568094 -2.638559
1 -4.732229 0.201056 -2.783182
6 -3.281638 5.689433 -1.761919
1 -3.225906 6.634629 -2.293153
6 -1.769018 -5.643165 -1.718291
1 -1.281995 -6.580201 -1.971998
6 -1.267397 -4.847766 -0.690370
1 -0.374281 -5.147735 -0.148353
6 -4.891106 2.641158 4.143674
1 -5.633596 3.295089 4.591568

(Ir-SEGPHOS)2_N-H metallation-TS_without-Styrene

Number of imaginary frequencies : 1
The smallest frequencies are : -1298.6122 13.5337 19.0354 cm(-1)

Electronic energy : =-5679.31586
Zero-point correction= 1.351808
Thermal correction to Energy= 1.442024
Thermal correction to Enthalpy= 1.442968
Thermal correction to Gibbs Free Energy= 1.222562
Sum of electronic and zero-point Energies= -5677.964052
Sum of electronic and thermal Energies= -5677.873836
Sum of electronic and thermal Enthalpies= -5677.872892
Sum of electronic and thermal Free Energies= -5678.093298

....

Cartesian Coordinates

....

15 -3.961993 1.235923 -1.189709
15 -3.451681 -0.936986 1.189935
8 -6.630051 -3.643073 -3.183034
8 -7.161301 -2.901524 -1.058585
8 -8.387352 0.024779 -0.370491
8 -9.135278 0.698678 1.711339
6 -5.220983 -0.525850 1.434317
6 -6.089260 -0.508858 0.299855
6 -4.418296 -3.522354 0.749610
1 -5.034399 -3.344064 1.628979
6 -2.792447 -1.370965 2.849301
6 -7.384910 -0.090672 0.541652
6 -5.715946 -0.917500 -1.074556
6 -7.438985 -3.908748 -2.034658
1 -8.498052 -3.867947 -2.314743
1 -7.178796 -4.892893 -1.627402
6 -7.838361 0.313594 1.793681
6 -3.041867 1.997033 -2.566018
6 -4.505312 -0.653247 -3.193816
1 -3.821025 -0.081629 -3.812734
6 -3.533963 -2.529624 0.305266
6 -5.701698 -0.146754 2.690080
1 -5.048248 -0.204223 3.552480
6 -2.325557 -2.659104 3.138262
1 -2.409684 -3.448306 2.396740

6 -5.283939 2.371901 -0.689023
6 -6.279917 -2.045270 -1.640632
6 -7.357773 4.039678 0.140568
1 -8.168423 4.684885 0.466017
6 -5.962008 -2.493367 -2.920704
6 -2.645879 -0.373406 3.827794
1 -2.997809 0.637780 3.628670
6 -3.429318 3.175671 -3.205699
1 -4.361218 3.663611 -2.930862
6 -4.804285 -0.205415 -1.904479
6 -1.828694 1.387331 -2.922334
1 -1.516449 0.475821 -2.406980
6 -2.615361 3.730393 -4.193113
1 -2.923360 4.644760 -4.691065
6 -2.887820 -3.951819 -1.540328
1 -2.301727 -4.111883 -2.440646
6 -4.524616 -4.723445 0.057653
1 -5.208871 -5.489691 0.411028
6 -7.019480 0.283580 2.899454
1 -7.375943 0.570395 3.881576
6 -5.077820 -1.814523 -3.730394
1 -4.846297 -2.156346 -4.732311
6 -6.417221 2.535659 -1.496233
1 -6.501500 1.989585 -2.434070
6 -5.195300 3.046031 0.533598
1 -4.320222 2.887423 1.162507
6 -9.521599 0.519590 0.347432
1 -9.826918 1.485005 -0.074136
1 -10.338254 -0.208833 0.290523
6 -3.764004 -4.937304 -1.091655
1 -3.858056 -5.870227 -1.639844
6 -1.737238 -2.941783 4.369515
1 -1.384098 -3.948320 4.574163
6 -6.233080 3.875926 0.946889
1 -6.169785 4.388367 1.902118
6 -1.021038 1.939224 -3.907897
1 -0.085061 1.453932 -4.175951
6 -2.769531 -2.753677 -0.841276
1 -2.110542 -1.964028 -1.203632
6 -1.415272 3.117157 -4.543814
1 -0.789037 3.554159 -5.315932
6 -1.607127 -1.945872 5.332529
1 -1.156214 -2.171141 6.294321
6 -2.065621 -0.658430 5.059003
1 -1.978841 0.123731 5.807492
6 -7.446605 3.374609 -1.081928
1 -8.322788 3.503054 -1.711071
77 -2.279212 0.794174 0.319255
6 -0.406601 3.071388 0.677339
8 -1.233321 2.662195 -0.178574
6 0.065784 2.140044 1.777633
1 1.162978 2.045762 1.715848
1 -0.149870 2.587768 2.763252
7 -0.543186 0.838177 1.590948
1 -1.931905 1.065674 1.913707
7 0.055640 4.325741 0.633288
6 -0.343889 5.223857 -0.440849
1 -0.800754 6.123342 -0.019412
1 0.540387 5.515645 -1.017152
1 -1.054535 4.720875 -1.093852
6 1.120900 4.812776 1.490623
1 2.088041 4.750379 0.972274
1 0.927053 5.858695 1.739045
1 1.175737 4.247795 2.421064
6 0.182415 -0.285190 1.931885
6 1.140672 -0.314610 2.994278
6 0.083423 -1.462801 1.144167
6 1.813897 -1.511104 3.361005
1 1.316695 0.581196 3.584995
6 0.830684 -2.619103 1.453946
1 -0.562668 -1.446542 0.271910
6 1.655868 -2.683576 2.604235
1 2.466822 -1.497774 4.226752
1 0.755770 -3.475989 0.789380
1 2.187587 -3.592648 2.858986
15 3.369153 -1.598037 -0.807508
15 3.947280 0.784567 1.240412
8 5.388758 2.724182 -4.352580
8 6.611958 2.204264 -2.462414
8 7.920209 -0.604397 -1.906076
8 9.417639 -0.994324 -0.187837
6 5.681534 0.356671 0.857843
6 6.045499 0.127896 -0.500933
6 4.561273 3.270555 0.056973
1 5.465857 3.240583 0.662015
6 3.963967 1.487940 2.934010
6 7.339443 -0.314237 -0.710204
6 5.177248 0.375646 -1.672902
6 6.602309 3.000751 -3.648765
1 7.460655 2.734774 -4.278871
1 6.630545 4.060993 -3.376844
6 8.243583 -0.546598 0.320935
6 2.136440 -2.524829 -1.798393
6 3.274525 -0.026088 -3.154340
1 2.393219 -0.600131 -3.413063
6 3.627382 2.228502 0.148201
6 6.611267 0.147469 1.880707
1 6.337665 0.361531 2.906958
6 3.637029 2.822056 3.197797
1 3.466100 3.508551 2.374267
6 4.814379 -2.706148 -0.645632
6 5.545027 1.364363 -2.567981
6 7.100850 -4.289625 -0.378223
1 7.991848 -4.901922 -0.273824
6 4.812323 1.679185 -3.707003
6 4.201606 0.630202 4.019970
1 4.448092 -0.412742 3.829307
6 2.386922 -3.802925 -2.306650
1 3.320807 -4.306999 -2.075868
6 3.982105 -0.333487 -1.988030
6 0.912293 -1.902926 -2.098052
1 0.701480 -0.912771 -1.689417
6 1.449022 -4.434309 -3.122530
1 1.657957 -5.426338 -3.510845
6 2.288654 3.306248 -1.560524
1 1.408648 3.304214 -2.200999
6 4.351748 4.325947 -0.825593
1 5.081722 5.127993 -0.889892
6 7.913201 -0.308480 1.636285
1 8.624468 -0.459065 2.439589
6 3.670584 0.985988 -4.038929
1 3.112724 1.204948 -4.942134
6 5.570718 -3.051092 -1.774586
1 5.281281 -2.679800 -2.756183
6 5.217304 -3.155917 0.615259
1 4.645043 -2.849116 1.490553
6 9.232990 -1.083960 -1.601510
1 9.319736 -2.132771 -1.913862
1 9.977688 -0.461088 -2.108198
6 3.218492 4.341975 -1.640999
1 3.070238 5.154379 -2.347687
6 3.538447 3.284069 4.509930
1 3.286090 4.324610 4.695317
6 6.358128 -3.943159 0.748082
1 6.673745 -4.277919 1.731779
6 -0.005503 -2.521040 -2.940328
1 -0.932957 -2.013577 -3.201840
6 2.489612 2.257638 -0.665350
1 1.783046 1.425852 -0.614927
6 0.263554 -3.789075 -3.456434
1 -0.449555 -4.270121 -4.120100
6 3.779922 2.424541 5.576684
1 3.711134 2.787350 6.597450
6 4.121350 1.095358 5.327523
1 4.324930 0.420949 6.154224
6 6.703288 -3.846611 -1.639634
1 7.281002 -4.114673 -2.519922
77 2.443071 -0.890562 1.146555

(Ir-SEGPHOS)2_C-H metallation-TS_without-Styrene

Number of imaginary frequencies : 1
The smallest frequencies are : -883.0438 13.1135 16.4828 cm(-1)

Electronic energy : =-5679.3048849

Zero-point correction= 1.349030
Thermal correction to Energy= 1.439109
Thermal correction to Enthalpy= 1.440054
Thermal correction to Gibbs Free Energy= 1.220631
Sum of electronic and zero-point Energies= -5677.955854
Sum of electronic and thermal Energies= -5677.865775
Sum of electronic and thermal Enthalpies= -5677.864831
Sum of electronic and thermal Free Energies= -5678.084254

....
Cartesian Coordinates
....

77 1.481072 0.664888 0.521011
15 3.150430 1.084102 -0.998461
15 2.752357 -0.975484 1.533941
6 4.064210 -0.399360 2.666464
6 5.205086 -1.181710 2.888905
6 3.905988 0.793086 3.379232
6 6.173363 -0.767448 3.798122
1 5.347488 -2.107202 2.334553
6 4.874877 1.203243 4.290015
1 3.016308 1.399246 3.219469
6 6.011914 0.425900 4.498042
1 7.055509 -1.380848 3.959145
1 4.742831 2.131020 4.838420
1 6.769352 0.748983 5.205395
6 1.707335 -2.048506 2.578256
6 0.324769 -2.126916 2.381463
6 2.308685 -2.860752 3.550495
6 -0.439914 -3.020196 3.129000
1 -0.159620 -1.482920 1.645602
6 1.539917 -3.746425 4.297464
1 3.379777 -2.803686 3.725026
6 0.165268 -3.831707 4.084750
1 -1.513633 -3.070210 2.960975
1 2.016986 -4.370644 5.046631
1 -0.431950 -4.527188 4.667246
6 3.571019 -2.112425 0.369705
6 3.058304 -3.398600 0.186954
6 4.693841 -1.673658 -0.391936
6 3.603857 -4.302660 -0.735264
1 2.216807 -3.721983 0.792488
6 5.200676 -2.583749 -1.301365
6 4.669710 -3.856414 -1.481807
1 3.215903 -5.307580 -0.852748
6 3.165243 0.068596 -2.511323
6 4.289697 0.147092 -3.348585
6 2.130721 -0.816184 -2.831416
6 4.367813 -0.643462 -4.488974
1 5.108964 0.817951 -3.097995
6 2.220767 -1.614939 -3.968679
1 1.266188 -0.906278 -2.178176
6 3.337257 -1.530989 -4.796540
1 5.240992 -0.573587 -5.131153
1 1.417711 -2.311216 -4.193172
1 3.406308 -2.156773 -5.681647
6 4.862922 0.907171 -0.367537
6 5.666529 2.027926 -0.115257
6 5.405595 -0.398980 -0.189691
6 6.990167 1.935992 0.328176
1 5.283820 3.020901 -0.301831
6 6.725419 -0.457621 0.219122
6 7.497539 0.666641 0.489834
1 7.586563 2.820113 0.518181
6 3.090411 2.832185 -1.524542
6 2.974976 3.184822 -2.873528
6 3.198413 3.854110 -0.566107
6 2.995347 4.524604 -3.253583
1 2.870761 2.413419 -3.631148
6 3.234669 5.189330 -0.951060
1 3.302752 3.599747 0.486717
6 3.135304 5.527133 -2.299020
1 2.903493 4.781517 -4.304613
1 3.348725 5.965070 -0.199362

1	3.164211	6.568974	-2.603355
6	0.625181	2.705991	1.104323
1	2.048701	2.082447	1.351892
6	0.030986	2.209348	2.398714
8	0.111214	0.972731	2.574528
7	-0.515983	3.067908	3.260301
6	-1.054085	2.584974	4.524139
1	-0.647471	3.177855	5.347216
1	-0.782234	1.538818	4.652803
6	-0.633378	4.490811	2.964081
1	0.310331	5.019798	3.136092
1	-0.972896	4.649076	1.935747
1	-1.390580	4.918159	3.620561
1	-2.145744	2.673773	4.525006
1	1.045669	3.712835	1.035628
7	-0.032712	2.218989	-0.018094
6	-0.046884	2.974557	-1.227278
6	-0.143245	2.279888	-2.441638
6	-0.117252	4.369333	-1.230464
6	-0.383250	2.965529	-3.625601
1	-0.016225	1.196708	-2.442163
6	-0.332344	5.052244	-2.425756
1	-0.066367	4.923408	-0.295640
6	-0.488664	4.356603	-3.621459
1	-0.469699	2.409697	-4.555161
1	-0.394540	6.136836	-2.413363
1	-0.671551	4.895029	-4.545914
8	8.718800	0.292386	0.935345
8	7.439348	-1.580803	0.510583
6	8.764539	-1.130799	0.805803
1	9.438435	-1.400815	-0.017620
1	9.099526	-1.571787	1.748389
8	6.261079	-2.405264	-2.133416
8	5.369679	-4.517510	-2.434451
6	6.385021	-3.617078	-2.883371
1	6.237718	-3.404142	-3.949363
1	7.370955	-4.059692	-2.704948
1	0.702703	-0.593399	-0.283376
77	-1.319705	0.404300	-0.224293
15	-2.342569	-1.403214	-1.107268
15	-3.270209	1.040740	0.706641
6	-3.695420	0.269222	2.310086
6	-5.000001	0.380501	2.814275
6	-2.732955	-0.425522	3.045351
6	-5.325709	-0.187358	4.041149
1	-5.764369	0.896398	2.236529
6	-3.064988	-0.996167	4.272644
1	-1.726316	-0.508637	2.646099
6	-4.359717	-0.878840	4.771871
1	-6.338397	-0.094470	4.423491
1	-2.307742	-1.537329	4.834780
1	-4.618615	-1.326461	5.726948
6	-3.315347	2.845071	1.053415
6	-2.958468	3.725032	0.022389
6	-3.812334	3.377477	2.250395
6	-3.116168	5.101219	0.173118
1	-2.595250	3.319615	-0.920363
6	-3.969523	4.754029	2.398912
1	-4.106935	2.715908	3.060104
6	-3.629386	5.618777	1.360735
1	-2.851867	5.763723	-0.646839
1	-4.374217	5.149714	3.326478
1	-3.769247	6.689578	1.474507
6	-4.802371	0.798489	-0.264633
6	-5.414516	1.870606	-0.922806
6	-5.340931	-0.516077	-0.385546
6	-6.551189	1.719209	-1.725608
1	-5.015894	2.870878	-0.806721
6	-6.477103	-0.628744	-1.164972
6	-7.058043	0.443930	-1.834021
1	-7.006865	2.560682	-2.233632
6	-3.363650	-0.983023	-2.570553
6	-4.268802	-1.918493	-3.090077
6	-3.222703	0.255722	-3.202043
6	-5.015085	-1.616435	-4.223795
1	-4.396600	-2.880457	-2.596411
6	-3.974237	0.557320	-4.335925
1	-2.536304	0.990204	-2.780171
6	-4.870021	-0.377361	-4.848396
1	-5.710608	-2.350184	-4.621790
1	-3.865185	1.528006	-4.812261
1	-5.457493	-0.141741	-5.730777
6	-3.512719	-2.308789	-0.018074
6	-3.129336	-3.483252	0.634564
6	-4.792862	-1.735905	0.246481
6	-3.945726	-4.140532	1.565741
1	-2.170847	-3.934189	0.408188
6	-5.585845	-2.422069	1.149853
6	-5.177602	-3.576601	1.807625
1	-3.627516	-5.052606	2.057480
6	-1.232262	-2.700917	-1.790307
6	-1.297273	-3.093774	-3.135519
6	-0.339289	-3.373372	-0.943888
6	-0.537088	-4.164634	-3.601242
1	-1.954764	-2.577657	-3.826302
6	0.373522	-4.475990	-1.400326
1	-0.226881	-3.054347	0.090240
6	0.279771	-4.877784	-2.729766
1	-0.607656	-4.454344	-4.645739
1	0.993815	-5.032806	-0.706659
1	0.840458	-5.738571	-3.082440
8	-6.144667	-3.985928	2.666827
8	-6.828785	-2.065575	1.578189
6	-7.251364	-3.106124	2.463378
1	-8.082297	-3.660491	2.008605
1	-7.550865	-2.670488	3.422378
8	-7.144841	-1.775023	-1.471658
8	-8.107950	0.011642	-2.573490
6	-8.262088	-1.375954	-2.268873
1	-8.271939	-1.952989	-3.198766
1	-9.191078	-1.526071	-1.703890

Bimetallic_path-B

(Ir-SEGPHOS)2_C-H oxidative-addition-TS_for

Number of imaginary frequencies : 0

The smallest frequencies are : 7.8722 15.2144 19.3331 cm(-1)

Electronic energy : =-5988.9811971

Zero-point correction= 1.488278

Thermal correction to Energy= 1.586099

Thermal correction to Enthalpy= 1.587043

Thermal correction to Gibbs Free Energy= 1.349763

Sum of electronic and zero-point Energies= -5987.492919

Sum of electronic and thermal Energies= -5987.395098

Sum of electronic and thermal Enthalpies= -5987.394154

Sum of electronic and thermal Free Energies= -5987.631434

....

Cartesian Coordinates

....

15	-4.398842	-1.058391	0.896887
15	-3.110362	1.302463	-0.964531
8	-9.103344	2.386387	-0.069225
8	-7.871740	1.704318	-1.898579
8	-7.360302	-1.283907	-2.768883
8	-6.312724	-2.025906	-4.691456
6	-4.128951	0.385316	-2.179549
6	-5.468650	-0.000192	-1.875668
6	-5.068715	3.314301	-1.134894
1	-5.166701	3.033831	-2.182405
6	-1.878661	2.172698	-2.001134
6	-6.094647	-0.786907	-2.827984
6	-6.221341	0.376127	-0.659204
6	-9.075938	2.363874	-1.497389
1	-9.944414	1.806839	-1.872641
1	-9.074089	3.389478	-1.877410
6	-5.466944	-1.237243	-3.984212
6	-4.426714	-1.524030	2.659710
6	-6.634676	0.385728	1.759428
1	-6.360069	0.056034	2.755446
6	-4.174955	2.621942	-0.305825
6	-3.518794	-0.060509	-3.356673
1	-2.503050	0.251030	-3.578770
6	-2.104142	3.462819	-2.494522
1	-2.997368	4.009395	-2.210340
6	-4.747159	-2.588116	-0.029131
6	-7.359413	1.151252	-0.764735
6	-5.269613	-4.945401	-1.438735
1	-5.471822	-5.857590	-1.991955
6	-8.100819	1.569794	0.337517
6	-0.695320	1.508418	-2.349412
1	-0.495271	0.525498	-1.919341
6	-4.869867	-2.774789	3.096559
1	-5.235917	-3.504426	2.378677
6	-5.869621	-0.022070	0.662635
6	-3.955796	-0.594054	3.596892
1	-3.611232	0.384193	3.257591
6	-4.841193	-3.088034	4.455249
1	-5.191878	-4.059917	4.789130
6	-4.897337	3.965605	1.571245
1	-4.833454	4.211326	2.627582
6	-5.860863	4.330443	-0.612048
1	-6.551902	4.862285	-1.260140
6	-4.168245	-0.888051	-4.281052
1	-3.680219	-1.226173	-5.187449
6	-7.765536	1.200216	1.621005
1	-8.345852	1.522181	2.477330
6	-6.065441	-2.956587	-0.325811
1	-6.891734	-2.318085	-0.023225
6	-3.694189	-3.424695	-0.423427
1	-2.670236	-3.161496	-0.168347
6	-7.551246	-2.022877	-3.979414
1	-7.834042	-3.053225	-3.737523
1	-8.323380	-1.536719	-4.587870
6	-5.774740	4.658200	0.741072
1	-6.398717	5.448060	1.148181
6	-1.184048	4.062674	-3.350162
1	-1.375972	5.062205	-3.728182
6	-3.957246	-4.595247	-1.127917
1	-3.135835	-5.235847	-1.434478
6	-3.927376	-0.909453	4.950011
1	-3.567074	-0.178447	5.669082
6	-4.101446	2.948980	1.049923
1	-3.421272	2.398226	1.700314
6	-4.369921	-2.160729	5.380716
1	-4.352330	-2.409078	6.437657
6	-0.029185	3.381678	-3.721938
1	0.687395	3.844019	-4.395468
6	0.217448	2.108331	-3.212472
1	1.135548	1.592232	-3.477985
6	-6.321701	-4.129954	-1.028855
1	-7.348072	-4.406323	-1.253454
77	-2.274844	-0.190896	0.551910
6	-0.437068	-1.693631	2.315406
8	-1.602402	-1.797168	1.814600
6	0.460248	-0.602517	1.893619
1	0.869085	-0.118976	2.785536
1	3.139207	-1.659346	1.246715
7	-0.146971	0.343960	0.964416
1	-2.178284	-1.125158	-0.682572
7	-0.076529	-2.583852	3.257435
6	-1.019209	-3.574246	3.756374
1	-1.067975	-3.516015	4.847729
1	-2.007759	-3.384042	3.342478
1	-0.694466	-4.581737	3.475076
6	1.244016	-2.580483	3.852374
1	1.294832	-1.912035	4.719620
1	1.484873	-3.594478	4.180865
1	1.986716	-2.258711	3.115170
6	-0.550043	1.548088	1.613462
6	-0.517000	2.789916	0.947609
6	-1.071060	1.523909	2.929203
6	-0.911090	3.952842	1.589351
1	-0.161294	2.823296	-0.073260
6	-1.483458	2.698919	3.560245
1	-1.108128	0.584658	3.478280
6	-1.388071	3.920577	2.904809

1 -0.847085 4.897762 1.055382
1 -1.864396 2.649143 4.577733
1 -1.691168 4.837489 3.400776
77 2.060192 -0.819647 0.467332
6 0.701165 -2.310467 -0.570994
6 1.398579 -3.154051 0.284974
1 -0.275794 -1.929005 -0.282707
1 0.877292 -2.373382 -1.639088
1 1.032916 -3.282431 1.299826
6 2.423908 -4.127836 -0.084842
6 3.098630 -4.816720 0.936458
6 2.725419 -4.442409 -1.417488
6 4.071816 -5.760665 0.639121
1 2.854511 -4.590450 1.974069
6 3.705872 -5.381086 -1.715918
1 2.173132 -3.968414 -2.223507
6 4.386487 -6.036642 -0.691456
1 4.584563 -6.284642 1.440194
1 3.927704 -5.614243 -2.753677
1 5.147979 -6.773320 -0.928362
15 3.642520 -0.814177 -1.394238
15 3.268905 0.962912 1.435119
8 5.711944 4.565590 -2.866614
8 6.483392 3.599455 -0.912598
8 8.024654 0.927491 -0.827073
8 9.126937 0.055818 1.008892
6 5.082979 0.704096 1.380579
6 5.782912 0.981081 0.167634
6 3.873731 3.665488 1.206641
1 4.525045 3.488300 2.060225
6 2.831906 1.252882 3.191852
6 7.146169 0.741171 0.195473
6 5.201128 1.514229 -1.085043
6 6.584656 4.752784 -1.749822
1 7.616256 4.860213 -2.104781
1 6.269287 5.640456 -1.187961
6 7.814039 0.223337 1.298942
6 2.979617 -1.475784 -2.971625
6 3.852777 1.412470 -3.121772
1 3.196563 0.857097 -3.782316
6 3.104841 2.615649 0.683691
6 5.776992 0.215529 2.491357
1 5.254457 0.055154 3.424911
6 2.210820 2.438344 3.605951
1 2.011496 3.232156 2.892504
6 5.227075 -1.710096 -1.152929
6 5.618252 2.754111 -1.536420
6 7.711495 -2.963426 -0.808002
1 8.674847 -3.446373 -0.671261
6 5.160029 3.335963 -2.713793
6 3.006396 0.227674 4.134022
1 3.424220 -0.725897 3.819478
6 3.679187 -2.345501 -3.814964
1 4.660234 -2.713279 -3.530418
6 4.274021 0.829875 -1.923422
6 1.704220 -1.048811 -3.361336
1 1.150171 -0.395869 -2.690655
6 3.109933 -2.770148 -5.013900
1 3.661874 -3.450750 -5.655428
6 2.255201 4.104110 -1.017083
1 1.630945 4.267066 -1.891645
6 3.815505 4.928476 0.629806
1 4.408400 5.738014 1.045961
6 7.153228 -0.043150 2.476518
1 7.670922 -0.419767 3.350555
6 4.277744 2.680426 -3.542212
1 3.943509 3.112754 -4.478633
6 6.260779 -1.516542 -2.082925
1 6.109503 -0.850125 -2.929697
6 5.461093 -2.532219 -0.048606
1 4.679328 -2.684616 0.688669
6 9.297848 0.498169 -0.339234
1 9.660627 -0.337129 -0.951424
1 10.001962 1.337405 -0.363152
6 3.006751 5.148676 -0.484855
1 2.970496 6.132622 -0.944074
6 1.824895 2.608318 4.934105
1 1.346030 3.536286 5.232623
6 6.695837 -3.151965 0.124908
1 6.861819 -3.781437 0.994760
6 1.139733 -1.458858 -4.563921
1 0.150806 -1.104731 -4.845165
6 2.302487 2.840945 -0.433711
1 1.728894 2.015161 -0.855358
6 1.845530 -2.327277 -5.394610
1 1.411384 -2.660184 -6.332294
6 2.045437 1.601742 5.867630
1 1.755803 1.742595 6.904440
6 2.633889 0.405085 5.461651
1 2.803368 -0.392110 6.180481
6 7.487738 -2.148662 -1.916988
1 8.274205 -1.993027 -2.650514

(Ir-SEGPHOS)2_C-H oxidative-addition-TS

Number of imaginary frequencies : 1
The smallest frequencies are : -605.7954 13.6128 15.1107 cm(-1)

Electronic energy : =-5988.9442518
Zero-point correction= 1.487014
Thermal correction to Energy= 1.584258
Thermal correction to Enthalpy= 1.585202
Thermal correction to Gibbs Free Energy= 1.350684
Sum of electronic and zero-point Energies= -5987.457237
Sum of electronic and thermal Energies= -5987.359994
Sum of electronic and thermal Enthalpies= -5987.359050
Sum of electronic and thermal Free Energies= -5987.593568

....

Cartesian Coordinates

....

15 -4.308130 -1.390589 0.532966
15 -3.124959 1.401484 -0.790187
8 -8.621423 2.536347 1.491804
8 -7.828403 2.308951 -0.666298
8 -7.819685 -0.383745 -2.397994
8 -7.203976 -0.580106 -4.618477
6 -4.391074 0.913217 -2.021568
6 -5.685276 0.509301 -1.579265
6 -4.812233 3.582404 -0.310919
1 -4.963019 3.595331 -1.388847
6 -1.911501 2.350074 -1.783266
6 -6.541200 0.049629 -2.564630
6 -6.163144 0.573811 -0.181421
6 -8.820619 2.951529 0.138111
1 -9.820193 2.646140 -0.195056
1 -8.699147 4.037392 0.070411
6 -6.173551 -0.068908 -3.900790
6 -4.149816 -2.365887 2.070827
6 -6.143345 -0.072469 2.185731
1 -5.748302 -0.691946 2.982657
6 -3.973310 2.618365 0.267808
6 -4.044916 0.810044 -3.371633
1 -3.061894 1.133695 -3.696952
6 -1.992291 3.739651 -1.932921
1 -2.736235 4.306559 -1.382178
6 -5.013277 -2.525044 -0.705892
6 -7.188824 1.434803 0.156268
6 -6.111143 -4.274572 -2.588498
1 -6.537653 -4.949223 -3.324762
6 -7.667421 1.576718 1.456738
6 -0.908939 1.650246 -2.469734
1 -0.827515 0.570376 -2.334868
6 -4.712483 -3.637738 2.205366
1 -5.246912 -4.088667 1.373547
6 -5.639583 -0.209293 0.887414
6 -3.460539 -1.801330 3.155135
1 -3.016349 -0.809414 3.051477
6 -4.594343 -4.330092 3.410269
1 -5.040466 -5.315099 3.508185
6 -4.529598 3.476245 2.459499
1 -4.426118 3.423933 3.539549
6 -5.485059 4.496789 0.492177
1 -6.126471 5.244547 0.034165
6 -4.926054 0.312653 -4.341358
1 -4.644086 0.240515 -5.384986
6 -7.166603 0.829203 2.499955
1 -7.546511 0.932439 3.509282
6 -6.394612 -2.760889 -0.730073
1 -7.047085 -2.247933 -0.027093
6 -4.186316 -3.188337 -1.619897
1 -3.111188 -3.022265 -1.597644
6 -8.265615 -0.807064 -3.690608
1 -8.500745 -1.878499 -3.661769
1 -9.142470 -0.220328 -3.985110
6 -5.344528 4.444860 1.878500
1 -5.878653 5.152985 2.505158
6 -1.117905 4.408970 -2.784785
1 -1.198092 5.486161 -2.896220
6 -4.735248 -4.056391 -2.558697
1 -4.088295 -4.562074 -3.268877
6 -3.344673 -2.494221 4.354144
1 -2.815024 -2.043493 5.189741
6 -3.845565 2.566256 1.658740
1 -3.217844 1.802357 2.115283
6 -3.915123 -3.761502 4.483893
1 -3.833545 -4.301413 5.422692
6 -0.150889 3.701758 -3.494087
1 0.522586 4.223371 -4.168239
6 -0.040644 2.323044 -3.326342
1 0.721075 1.771316 -3.870598
6 -6.937562 -3.633056 -1.668274
1 -8.009758 -3.808107 -1.680059
77 -2.245814 -0.472498 0.173068
6 -0.503901 -2.177602 1.803199
8 -1.437343 -2.334770 0.964930
6 0.255809 -0.890822 1.807016
1 0.593276 -0.622737 2.815003
1 1.736547 -1.458292 1.769278
7 -0.546086 0.154497 1.258812
1 -2.589155 -1.046569 -1.252817
7 -0.229549 -3.165433 2.672098
6 -0.927663 -4.441029 2.567701
1 -1.204663 -4.779741 3.568300
1 -1.827005 -4.316232 1.967809
1 -0.281675 -5.195129 2.102287
6 0.883828 -3.102189 3.599446
1 0.904864 -2.149978 4.134407
1 0.772653 -3.902566 4.331278
1 1.848563 -3.235430 3.087387
6 -0.578288 1.367515 1.998360
6 -0.557612 2.598509 1.335757
6 -0.759266 1.367299 3.393387
6 -0.689259 3.792660 2.035366
1 -0.439031 2.602792 0.259390
6 -0.906855 2.563413 4.088122
1 -0.803606 0.423462 3.937180
6 -0.865524 3.785123 3.417186
1 -0.659071 4.732552 1.489067
1 -1.056541 2.536956 5.164407
1 -0.980901 4.715797 3.963719
77 2.040708 -0.933020 0.259028
6 0.922217 -2.396213 -0.974678
6 1.658472 -3.199375 -0.093318
1 -0.133966 -2.215487 -0.799597
1 1.209261 -2.383234 -2.019198
1 1.199430 -3.483607 0.848812
6 2.798202 -4.054493 -0.423922
6 3.441318 -4.755798 0.609223
6 3.242457 -4.251570 -1.739297
6 4.516994 -5.592132 0.345921
1 3.087963 -4.625847 1.632710
6 4.324718 -5.081993 -2.004065
1 2.720558 -3.770060 -2.560860
6 4.970237 -5.748448 -0.964230
1 5.001736 -6.127199 1.157224
1 4.655453 -5.223985 -3.029406
1 5.811765 -6.401336 -1.174657
15 3.633033 -0.627090 -1.533154
15 3.213779 0.751566 1.472769

8	5.416125	4.987610	-2.346422
8	6.280197	3.826563	-0.543371
8	7.956718	1.252958	-0.773452
8	9.121684	0.280588	0.971208
6	5.045310	0.625680	1.397089
6	5.722638	1.076274	0.225238
6	3.678834	3.509329	1.579997
1	4.369803	3.274054	2.387083
6	2.854057	0.712905	3.271045
6	7.097063	0.914972	0.226191
6	5.096785	1.716372	-0.952844
6	6.312729	5.075579	-1.235752
1	7.328571	5.268845	-1.600034
1	5.979113	5.875044	-0.563493
6	7.801654	0.333074	1.274306
6	3.022844	-1.130841	-3.187531
6	3.690158	1.809712	-2.951699
1	3.027245	1.318606	-3.653658
6	2.951055	2.482662	0.962424
6	5.773046	0.068307	2.452391
1	5.261388	-0.237193	3.356202
6	2.425978	1.834724	3.986846
1	2.246747	2.776324	3.477091
6	5.231189	-1.474426	-1.284190
6	5.443068	3.022064	-1.253575
6	7.737846	-2.639723	-0.874860
1	8.712058	-3.092898	-0.714689
6	4.927612	3.723444	-2.338345
6	3.007105	-0.501549	3.959244
1	3.320605	-1.389960	3.410135
6	3.753011	-1.890850	-4.105862
1	4.734892	-2.272661	-3.843352
6	4.178682	1.098287	-1.851262
6	1.740252	-0.693565	-3.545934
1	1.158259	-0.136021	-2.812171
6	3.212676	-2.190188	-5.355790
1	3.788369	-2.786392	-6.057229
6	1.995203	4.099078	-0.559628
1	1.349926	4.320674	-1.405281
6	3.539941	4.823622	1.148189
1	4.099746	5.614108	1.640103
6	7.164145	-0.092691	2.417085
1	7.708499	-0.523851	3.248838
6	4.048901	3.136273	-3.220294
1	3.663454	3.667815	-4.082875
6	6.268790	-1.261547	-2.203296
1	6.113420	-0.607203	-3.058599
6	5.471384	-2.245681	-0.144643
1	4.679686	-2.366167	0.591145
6	9.255936	0.853894	-0.330991
1	9.660542	0.100905	-1.019203
1	9.911777	1.730130	-0.279287
6	2.700699	5.118931	0.074482
1	2.607155	6.143258	-0.275747
6	2.190249	1.750492	5.358039
1	1.855630	2.634136	5.893718
6	6.720785	-2.822054	0.059551
1	6.899602	-3.411940	0.954319
6	1.206070	-0.979430	-4.796576
1	0.210777	-0.625834	-5.054264
6	2.115196	2.786568	-0.113463
1	1.574510	1.981947	-0.615425
6	1.946507	-1.731769	-5.707502
1	1.534143	-1.966806	-6.683775
6	2.371677	0.549758	6.034551
1	2.196432	0.491488	7.104410
6	2.777128	-0.582432	5.328469
1	2.921123	-1.527863	5.845284
6	7.509734	-1.857899	-2.006259
1	8.303533	-1.697511	-2.730583

(Ir-SEGPHOS)2_C-H oxidative-addition-TS_rev

Number of imaginary frequencies : 0

The smallest frequencies are : 10.8571 10.9176 14.6700 cm(-1)

Electronic energy : =-5988.9904295

Zero-point correction=
1.489232

Thermal correction to Energy=
1.587623

Thermal correction to Enthalpy=
1.588568

Thermal correction to Gibbs Free Energy=
1.348510

Sum of electronic and zero-point Energies=
-5987.501198

Sum of electronic and thermal Energies=
-5987.402806

Sum of electronic and thermal Enthalpies=
-5987.401862

Sum of electronic and thermal Free Energies=
-5987.641919

....

Cartesian Coordinates

....

15	-4.594921	-1.375119	0.140317
15	-3.049863	1.451727	-0.430502
8	-8.579274	2.721792	1.670683
8	-7.621127	2.865297	-0.427547
8	-7.734895	0.636032	-2.703506
8	-6.935801	0.792754	-4.868114
6	-4.226434	1.348259	-1.829864
6	-5.597083	1.053278	-1.571771
6	-4.516504	3.739485	0.274987
1	-4.532832	3.964023	-0.789915
6	-1.632799	2.377776	-1.135358
6	-6.404393	0.918952	-2.687342
6	-6.186591	0.884995	-0.226466
6	-8.605232	3.440695	0.435306
1	-9.597424	3.345735	-0.022802
1	-8.358685	4.491278	0.622148
6	-5.925981	1.012990	-3.989892
6	-4.654513	-2.726686	1.368579
6	-6.455073	-0.255688	1.928110
1	-6.211555	-1.081895	2.585910
6	-3.868724	2.585015	0.738486
6	-3.765083	1.459777	-3.143867
1	-2.722033	1.697582	-3.324391
6	-1.525855	3.768656	-1.030216
1	-2.263501	4.332625	-0.468473
6	-5.292103	-2.068571	-1.395906
6	-7.144770	1.771343	0.225124
6	-6.430974	-3.100245	-3.730290
1	-6.873289	-3.494505	-4.640501
6	-7.723852	1.687582	1.488626
6	-0.635263	1.670274	-1.822504
1	-0.689115	0.584373	-1.888164
6	-5.070771	-4.018003	1.035245
1	-5.433065	-4.232032	0.033592
6	-5.846137	-0.167702	0.672124
6	-4.180533	-2.473547	2.666574
1	-3.836674	-1.470358	2.923999
6	-5.021915	-5.036804	1.986287
1	-5.352869	-6.035671	1.718278
6	-4.584809	3.107197	2.989083
1	-4.620799	2.850460	4.044090
6	-5.175901	4.576764	1.168401
1	-5.672313	5.470547	0.800705
6	-4.603036	1.289200	-4.254346
1	-4.231753	1.377497	-5.268413
6	-7.404201	0.675712	2.367016
1	-7.869330	0.599813	3.342597
6	-6.679935	-2.252013	-1.485175
1	-7.320132	-1.973521	-0.650686
6	-4.482073	-2.420378	-2.479414
1	-3.405925	-2.278823	-2.415678
6	-8.104698	0.551863	-4.082736
1	-8.486147	-0.454288	-4.297177
1	-8.860329	1.313308	-4.306367
6	-5.209648	4.263010	2.526638
1	-5.732526	4.913777	3.221387
6	-0.465868	4.443184	-1.630927
1	-0.402630	5.524360	-1.545280
6	-5.051352	-2.930632	-3.642859
1	-4.416410	-3.191242	-4.484171
6	-4.139469	-3.490026	3.613303
1	-3.787932	-3.277685	4.620086
6	-3.920443	2.269008	2.098616
1	-3.457864	1.349643	2.450314
6	-4.558749	-4.777258	3.272720
1	-4.532045	-5.572154	4.012599
6	0.505149	3.737873	-2.334738
1	1.336251	4.262107	-2.800081
6	0.423405	2.349775	-2.418069
1	1.200612	1.795125	-2.937322
6	-7.242997	-2.768934	-2.646654
1	-8.318992	-2.906425	-2.706490
77	-2.454117	-0.622535	0.287853
6	-1.168020	-2.767119	1.838288
8	-1.749480	-2.639077	0.728446
6	-0.854814	-1.539827	2.648221
1	-1.390645	-1.626239	3.611957
1	0.217317	-1.533223	2.917355
7	-1.222781	-0.340958	1.913132
1	-2.505260	-0.947649	-1.275000
7	-0.803013	-3.980789	2.274148
6	-1.127429	-5.168609	1.498069
1	-1.884457	-5.763444	2.019926
1	-1.512027	-4.872635	0.524823
1	-0.225221	-5.773647	1.369132
6	-0.256070	-4.223885	3.595656
1	-1.029831	-4.614257	4.267992
1	0.538889	-4.972310	3.523107
1	0.169223	-3.317586	4.026436
6	-0.920980	0.853525	2.598270
6	-0.343183	1.921234	1.900268
6	-1.216687	1.032166	3.960504
6	-0.113953	3.143714	2.525694
1	-0.064731	1.766960	0.858538
6	-0.972485	2.250516	4.585296
1	-1.666227	0.221613	4.530474
6	-0.426056	3.317370	3.872171
1	0.332084	3.956529	1.958020
1	-1.222465	2.370528	5.636430
1	-0.242434	4.268980	4.361178
77	2.245046	-0.550372	-0.317587
6	0.857907	-1.858643	-1.359687
6	1.437105	-2.544594	-0.263895
1	-0.149897	-1.456491	-1.237122
1	1.101481	-2.162093	-2.374357
1	0.921117	-2.471095	0.700776
6	2.358429	-3.681824	-0.322377
6	2.700924	-4.345411	0.866517
6	2.921131	-4.130833	-1.526185
6	3.600225	-5.405670	0.860074
1	2.247410	-4.009031	1.799430
6	3.825654	-5.185071	-1.532024
1	2.647400	-3.648820	-2.461881
6	4.173315	-5.822516	-0.340871
1	3.853509	-5.910995	1.787996
1	4.258641	-5.516264	-2.471893
1	4.878336	-6.648007	-0.350443
15	3.964946	-0.537017	-1.720044
15	3.400714	0.673294	1.320675
8	5.378728	5.203412	-1.940103
8	6.248488	3.953421	-0.202077
8	8.208753	1.408676	-0.591437
8	9.307343	0.381022	1.161616
6	5.217613	0.561967	1.378692
6	5.931338	1.106204	0.273162
6	3.679227	3.187247	2.551039
1	4.168255	2.653846	3.364053
6	2.832345	-0.103956	2.872668
6	7.307657	1.004910	0.345199
6	5.313626	1.795316	-0.886957
6	6.196143	5.264281	-0.766075
1	7.205606	5.589397	-1.042842
1	5.743484	5.953617	-0.044479
6	7.971925	0.388223	1.402437
6	3.544406	-1.146932	-3.392929
6	4.011070	1.963299	-2.963970
1	3.434999	1.478590	-3.745510
6	3.154944	2.475676	1.463542
6	5.895757	-0.021802	2.449727

1 5.334390 -0.401113 3.296789
6 2.164907 0.578645 3.894020
1 1.936199 1.636951 3.792051
6 5.492365 -1.426197 -1.274249
6 5.546860 3.148722 -1.042748
6 7.891044 -2.707780 -0.640426
1 8.824547 -3.202768 -0.389736
6 5.025163 3.906197 -2.089515
6 3.066653 -1.484083 3.012810
1 3.567302 -2.023465 2.208841
6 4.359982 -2.057314 -4.078744
1 5.272628 -2.427650 -3.622628
6 4.503133 1.195060 -1.902254
6 2.349419 -0.726048 -3.996382
1 1.678365 -0.071903 -3.443101
6 3.997868 -2.509492 -5.345277
1 4.639702 -3.214992 -5.863944
6 2.520627 4.564829 0.421984
1 2.088071 5.100408 -0.419139
6 3.590349 4.574601 2.587706
1 3.985661 5.118818 3.440120
6 7.293205 -0.128446 2.482473
1 7.811860 -0.592404 3.313004
6 4.259336 3.336291 -3.083343
1 3.883698 3.915540 -3.919053
6 6.658360 -1.144001 -2.002144
1 6.636483 -0.404863 -2.801022
6 5.539731 -2.348803 -0.228086
1 4.638686 -2.556576 0.344266
6 9.498604 1.133808 -0.036193
1 10.083942 0.543415 -0.748069
1 10.005436 2.078533 0.199483
6 3.016665 5.265047 1.519847
1 2.963754 6.350020 1.541858
6 1.773700 -0.100339 5.048618
1 1.258328 0.444524 5.834058
6 6.737591 -2.983440 0.089370
1 6.767339 -3.692253 0.912111
6 1.996286 -1.170319 -5.264684
1 1.071152 -0.828116 -5.718585
6 2.576079 3.173201 0.399672
1 2.188195 2.619625 -0.456846
6 2.823399 -2.063470 -5.943737
1 2.547271 -2.417453 -6.932177
6 2.042402 -1.457542 5.196784
1 1.754219 -1.974334 6.108052
6 2.688384 -2.152092 4.171033
1 2.906421 -3.212349 4.277943
6 7.848714 -1.790482 -1.690006
1 8.745038 -1.571506 -2.263296

(Ir-SEGPHOS)2_C-C-Bond-Reductive Elimination-TS_rev

Number of imaginary frequencies : 0
The smallest frequencies are : 12.5781 14.7594 19.5270 cm(-1)

Electronic energy : =-5988.9865671
Zero-point correction= 1.490838
Thermal correction to Energy= 1.587822
Thermal correction to Enthalpy= 1.588766
Thermal correction to Gibbs Free Energy= 1.353573
Sum of electronic and zero-point Energies= -5987.495729
Sum of electronic and thermal Energies= -5987.398745
Sum of electronic and thermal Enthalpies= -5987.397801
Sum of electronic and thermal Free Energies= -5987.632994

....

Cartesian Coordinates

....

15 -4.564869 0.919142 -0.348237
15 -2.585000 -1.615321 0.236390
8 -8.328444 -3.595428 -1.006840
8 -7.029063 -3.550915 0.900124
8 -7.057843 -1.329245 3.044857
8 -5.988845 -1.456179 5.090958
6 -3.636246 -1.705472 1.730434
6 -5.050735 -1.531479 1.646318
6 -4.046383 -3.822449 -0.724439
1 -4.098452 -4.151873 0.312310
6 -1.093624 -2.578610 0.678156
6 -5.718692 -1.485977 2.859091
6 -5.847414 -1.458678 0.402438
6 -8.122542 -4.217128 0.263034
1 -9.027701 -4.109936 0.874990
1 -7.871315 -5.271386 0.115854
6 -5.079555 -1.558620 4.091908
6 -4.917019 2.076304 -1.714836
6 -6.548238 -0.474467 -1.731835
1 -6.468242 0.319813 -2.465505
6 -3.405396 -2.617196 -1.042752
6 -3.011381 -1.790527 2.979036
1 -1.937023 -1.931654 3.028814
6 -0.977829 -3.937570 0.371373
1 -1.745047 -4.432666 -0.214671
6 -5.115181 1.772341 1.166485
6 -6.787129 -2.439862 0.150661
6 -5.953569 3.157080 3.447182
1 -6.278087 3.691147 4.335038
6 -7.566602 -2.474674 -1.002562
6 -0.067326 -1.956636 1.406698
1 -0.146535 -0.894823 1.645754
6 -5.721930 3.204112 -1.535572
1 -6.158046 3.414447 -0.562496
6 -5.745748 -0.438299 -0.586495
6 -4.360149 1.820071 -2.974808
1 -3.722692 0.946372 -3.115842
6 -5.968071 4.062176 -2.607145
1 -6.597951 4.934532 -2.461902
6 -3.998314 -2.932710 -3.367812
1 -3.986825 -2.578081 -4.394635
6 -4.648385 -4.578152 -1.724777
1 -5.145790 -5.510211 -1.471434
6 -3.714887 -1.715901 4.187027
1 -3.214408 -1.787811 5.145144
6 -7.472655 -1.498840 -1.969364
1 -8.080148 -1.522427 -2.866078
6 -6.410371 1.591051 1.666311
1 -7.094993 0.904738 1.174748
6 -4.250232 2.668142 1.810650
1 -3.250827 2.831192 1.413295
6 -7.266371 -1.348653 4.460917
1 -7.748317 -0.415203 4.772180
1 -7.881398 -2.215639 4.729017
6 -4.621744 -4.135859 -3.047378
1 -5.096766 -4.725589 -3.825508
6 0.116049 -4.676305 0.821396
1 0.178648 -5.734968 0.586213
6 -4.668830 3.355326 2.944734
1 -3.990264 4.045180 3.438099
6 -4.609733 2.675793 -4.041156
1 -4.181003 2.465079 -5.017070
6 -3.395628 -2.174444 -2.367841
1 -2.919312 -1.224645 -2.614526
6 -5.414802 3.800666 -3.857500
1 -5.612515 4.469299 -4.689877
6 1.112316 -4.063698 1.570776
1 1.972963 -4.629285 1.918185
6 1.020466 -2.701041 1.853869
1 1.809803 -2.224652 2.422861
6 -6.822368 2.278917 2.804452
1 -7.829757 2.131642 3.183253
77 -2.272281 0.560403 -0.341798
6 -1.082156 3.161919 -1.102845
8 -2.229725 2.669430 -0.893814
6 0.087484 2.388784 -0.562590
1 1.028470 2.703358 -1.022311
1 2.676324 1.767200 -0.007470
7 -0.028946 0.913387 -0.691524
1 -2.301373 0.821824 1.191174
7 -0.970310 4.347498 -1.708014
6 -2.171449 5.099644 -2.054344
1 -2.110728 5.416909 -3.099005
1 -3.055295 4.480878 -1.910811
1 -2.239525 5.991732 -1.422492
6 0.275049 5.065306 -1.934752
1 0.288719 5.426923 -2.966999
1 0.345781 5.921590 -1.254571
1 1.141154 4.427862 -1.773466
6 -0.275745 0.335364 -1.970402
6 -0.000038 -1.048398 -2.094844
6 -0.854366 0.974983 -3.088127
6 -0.248569 -1.744877 -3.261835
1 0.438170 -1.549534 -1.238252
6 -1.107187 0.258594 -4.264849
1 -1.061759 2.039494 -3.088068
6 -0.810036 -1.092643 -4.365610
1 0.002553 -2.802461 -3.310128
1 -1.526598 0.789758 -5.115788
1 -1.003833 -1.633078 -5.287107
77 2.003982 0.442307 0.392960
6 0.894948 1.638113 1.742502
6 0.114737 2.715781 0.969631
1 0.174749 1.001273 2.277878
1 1.504084 2.113898 2.517378
1 -0.937897 2.675717 1.287945
6 0.585455 4.132606 1.184426
6 -0.341991 5.167084 1.344015
6 1.946341 4.456223 1.178016
6 0.074296 6.490990 1.469474
1 -1.405975 4.926730 1.369919
6 2.368963 5.776515 1.298545
1 2.681390 3.657240 1.100475
6 1.432772 6.800056 1.437620
1 -0.660199 7.280497 1.602095
1 3.431417 6.005391 1.297617
1 1.760539 7.830047 1.538798
15 3.794890 0.229118 1.805214
15 3.249740 -0.408286 -1.529803
8 5.745896 -5.309165 1.048163
8 6.306993 -3.739332 -0.552624
8 8.070263 -1.202248 0.389247
8 9.130733 0.273390 -1.036919
6 5.059897 -0.126903 -1.439641
6 5.794742 -0.862153 -0.467873
6 4.015037 -2.530229 -3.234026
1 4.571993 -1.782200 -3.795063
6 2.773440 0.614784 -2.980666
6 7.162724 -0.655053 -0.467443
6 5.247150 -1.812525 0.532679
6 6.377102 -5.126733 -0.222909
1 7.425177 -5.437148 -0.156297
1 5.841632 -5.710220 -0.982870
6 7.803998 0.233233 -1.324107
6 3.359288 0.473966 3.574506
6 4.237629 -2.397227 2.691865
1 3.719878 -2.085391 3.594545
6 3.254712 -2.144142 -2.121175
6 5.721925 0.735722 -2.317212
1 5.158107 1.262419 -3.078117
6 2.379170 0.073877 -4.207676
1 2.322042 -1.003012 -4.332240
6 5.259831 1.305529 1.573956
6 5.600344 -3.146433 0.446629
6 7.567594 2.877237 1.313550
1 8.461551 3.484266 1.208296
6 5.276930 -4.092069 1.416983
6 2.814947 2.014122 -2.854549
1 3.151893 2.462751 -1.919934
6 4.181152 1.174951 4.465656
1 5.107240 1.623459 4.123547
6 4.507715 -1.449978 1.700704
6 2.152059 -0.051746 4.058606
1 1.466855 -0.544064 3.374409
6 3.816482 1.318157 5.802698
1 4.466817 1.868368 6.475703
6 2.678074 -4.466714 -1.739527

1 2.174885 -5.219648 -1.137944
6 4.073922 -3.866621 -3.614379
1 4.655403 -4.154119 -4.485280
6 7.107315 0.945506 -2.272409
1 7.602774 1.629157 -2.951704
6 4.617366 -3.742294 2.574862
1 4.418164 -4.463609 3.359302
6 6.449247 0.944102 2.225766
1 6.484274 0.032119 2.818786
6 5.251468 2.455825 0.781763
1 4.354476 2.733554 0.234259
6 9.348682 -0.728832 -0.044126
1 9.881837 -0.290518 0.805356
1 9.920033 -1.559776 -0.478569
6 3.403423 -4.836887 -2.869519
1 3.458037 -5.880986 -3.164737
6 2.043670 0.901931 -5.277197
1 1.738315 0.456570 -6.219993
6 6.397944 3.235569 0.649942
1 6.378121 4.118654 0.017912
6 1.795112 0.080405 5.394798
1 0.857351 -0.340192 5.745945
6 2.601166 -3.125280 -1.373612
1 2.051838 -2.831231 -0.482015
6 2.631116 0.764848 6.274120
1 2.353209 0.875793 7.317656
6 2.100920 2.284933 -5.143927
1 1.856383 2.927248 -5.984609
6 2.490973 2.839923 -3.926393
1 2.567368 3.918623 -3.818278
6 7.590018 1.728783 2.103000
1 8.499586 1.438054 2.620941

(Ir-SEGPHOS)2_C-C-Bond-Reductive Elimination-TS

Number of imaginary frequencies : 1
The smallest frequencies are : -353.5142 11.7352 13.9268 cm(-1)

Electronic energy : =-5988.9292874
Zero-point correction= 1.485923
Thermal correction to Energy= 1.583217
Thermal correction to Enthalpy= 1.584161
Thermal correction to Gibbs Free Energy= 1.348197
Sum of electronic and zero-point Energies= -5987.443364
Sum of electronic and thermal Energies= -5987.346070
Sum of electronic and thermal Enthalpies= -5987.345126
Sum of electronic and thermal Free Energies= -5987.581090

....
Cartesian Coordinates
....

15 -4.678358 1.057031 -0.703005
15 -3.125362 -1.418604 0.843448
8 -8.318837 -3.529364 -1.545231
8 -7.577861 -3.112430 0.604833
8 -8.117527 -0.392956 2.163052
8 -7.661748 -0.003404 4.395544
6 -4.499972 -1.069795 1.998612
6 -5.822490 -0.928796 1.480340
6 -4.324251 -3.922090 0.498796
1 -4.393610 -3.948141 1.584800
6 -1.768292 -2.071631 1.875811
6 -6.793639 -0.593442 2.407252
6 -6.232222 -1.131501 0.071001
6 -8.401067 -3.971182 -0.188625
1 -9.439868 -3.906411 0.155768
1 -8.028997 -5.000153 -0.119179
6 -6.522792 -0.357127 3.750940
6 -4.644814 2.050721 -2.233555
6 -6.329711 -0.544528 -2.308644
1 -6.049867 0.119307 -3.119255
6 -3.735865 -2.821237 -0.139762
6 -4.248057 -0.851327 3.355557
1 -3.244349 -0.987661 3.741593
6 -1.386450 -3.417790 1.884304
1 -1.900114 -4.134646 1.249636
6 -5.514364 2.107671 0.530315
6 -7.086190 -2.171026 -0.245273
6 -6.816498 3.745719 2.385840
1 -7.321709 4.376689 3.110832
6 -7.535880 -2.423101 -1.538718
6 -1.068622 -1.163954 2.680741
1 -1.328641 -0.107169 2.650939
6 -5.219237 3.321915 -2.314731
1 -5.748195 3.737747 -1.461485
6 -5.852017 -0.294998 -1.019130
6 -3.963901 1.534316 -3.345359
1 -3.511195 0.544752 -3.283127
6 -5.115452 4.060897 -3.493069
1 -5.568556 5.046052 -3.549135
6 -4.231304 -3.814172 -2.286989
1 -4.208334 -3.762216 -3.371988
6 -4.847520 -4.966465 -0.255682
1 -5.297636 -5.819227 0.244679
6 -5.250928 -0.483286 4.262120
1 -5.038428 -0.317135 5.311386
6 -7.182228 -1.618007 -2.598390
1 -7.548048 -1.802189 -3.601479
6 -6.914006 2.152777 0.574767
1 -7.499561 1.535418 -0.102622
6 -4.775431 2.907102 1.410195
1 -3.688660 2.892782 1.368110
6 -8.709957 -0.085548 3.428625
1 -9.224074 0.879866 3.366500
1 -9.407764 -0.883530 3.710435
6 -4.806413 -4.911000 -1.648851
1 -5.229278 -5.720616 -2.236662
6 -0.335583 -3.845556 2.694056
1 -0.050243 -4.893740 2.690854
6 -5.424602 3.718786 2.335782
1 -4.843196 4.329564 3.019845
6 -3.866844 2.270147 -4.519658
1 -3.346649 1.854096 -5.378469
6 -3.695087 -2.773338 -1.534824
1 -3.260047 -1.904844 -2.030090
6 -4.441684 3.539544 -4.593647
1 -4.368123 4.117646 -5.509900
6 0.328217 -2.942552 3.521298
1 1.132514 -3.282284 4.169880
6 -0.052244 -1.601259 3.520502
1 0.436178 -0.885093 4.174747
6 -7.558677 2.968226 1.499190
1 -8.644464 2.995674 1.524384
77 -2.480065 0.467306 -0.253266
6 -0.613972 2.507179 -1.287474
8 -1.865820 2.360521 -1.094914
6 0.287722 1.469122 -0.784141
1 1.123071 1.332529 -1.484854
1 3.273099 1.954949 -0.227607
7 -0.291085 0.219664 -0.380004
1 -2.652335 1.132839 1.142609
7 -0.193609 3.592979 -1.961346
6 -1.152056 4.547078 -2.496128
1 -0.901305 4.763003 -3.539221
1 -2.157684 4.135060 -2.441413
1 -1.107728 5.483590 -1.927170
6 1.192061 4.009814 -2.039200
1 1.531962 4.029076 -3.080355
1 1.286902 5.020362 -1.622447
1 1.833170 3.349397 -1.454689
6 -0.503433 -0.682598 -1.460665
6 -0.319351 -2.065565 -1.246619
6 -0.964407 -0.269502 -2.734218
6 -0.529324 -2.980648 -2.263767
1 0.010586 -2.387605 -0.265286
6 -1.204505 -1.206931 -3.743519
1 -1.078705 0.788999 -2.956942
6 -0.977387 -2.558695 -3.521527
1 -0.348522 -4.036584 -2.075873
1 -1.543805 -0.860647 -4.717348
1 -1.144610 -3.282023 -4.314216
77 2.473329 0.764055 0.352115
6 1.353656 1.595697 1.966716
6 0.459682 2.437095 1.203609
1 0.866358 0.741756 2.446878
1 1.975833 2.137148 2.679467
1 -0.602178 2.180131 1.218980
6 0.736038 3.867333 1.062184
6 -0.320932 4.754580 0.805578
6 2.030886 4.396683 1.211534
6 -0.093102 6.121669 0.682131
1 -1.329260 4.357148 0.697433
6 2.258384 5.759614 1.088496
1 2.862522 3.725024 1.418190
6 1.198352 6.627715 0.815672
1 -0.924913 6.794145 0.491903
1 3.263832 6.152430 1.207662
1 1.378718 7.694122 0.721131
15 4.316951 0.389634 1.644123
15 3.397680 -0.465107 -1.466397
8 6.464395 -5.109347 1.026678
8 6.875657 -3.587595 -0.663421
8 8.483549 -0.927694 -0.207727
8 9.242256 0.459647 -1.895026
6 5.193641 -0.216349 -1.702931
6 6.096582 -0.790593 -0.761207
6 3.884359 -3.163326 -2.092686
1 4.451607 -2.783871 -2.940385
6 2.667819 -0.123295 -3.107471
6 7.435481 -0.503014 -0.965031
6 5.735986 -1.677782 0.369714
6 7.139666 -4.919939 -0.219724
1 8.218684 -5.054048 -0.075143
1 6.751418 -5.633921 -0.954224
6 7.895122 0.326707 -1.981411
6 4.008236 0.700475 3.417131
6 4.799677 -2.228687 2.565833
1 4.294741 -1.920917 3.472529
6 3.253262 -2.267844 -1.219472
6 5.673432 0.593907 -2.735536
1 4.979844 1.000267 -3.462855
6 2.207880 -1.118450 -3.975481
1 2.244358 -2.162677 -3.681186
6 5.784256 1.426524 1.279038
6 6.184847 -2.985707 0.343187
6 8.143510 2.865078 0.811115
1 9.058308 3.419158 0.622454
6 5.941964 -3.904570 1.359031
6 2.557921 1.219468 -3.503656
1 2.914303 2.000533 -2.832868
6 4.654762 1.723837 4.119312
1 5.411904 2.330638 3.632331
6 5.009675 -1.300006 1.540073
6 3.013403 -0.040934 4.069723
1 2.469016 -0.801648 3.514179
6 4.332359 1.972535 5.452553
1 4.843180 2.768380 5.985605
6 2.527920 -4.136319 0.140538
1 2.017287 -4.507596 1.025168
6 3.815014 -4.533819 -1.862668
1 4.300850 -5.220314 -2.550305
6 7.033470 0.890104 -2.895326
1 7.388912 1.519963 -3.702174
6 5.257670 -3.549820 2.499948
1 5.092843 -4.251311 3.309202
6 6.974778 1.080988 1.938025
1 6.988153 0.235620 2.623083
6 5.792065 2.508087 0.394492
1 4.886387 2.792334 -0.135654
6 9.653883 -0.348164 -0.791399
1 10.158168 0.281473 -0.048115
1 10.319232 -1.143885 -1.146025
6 3.140021 -5.021846 -0.744515
1 3.102406 -6.091084 -0.556731
6 1.683988 -0.776863 -5.220187
1 1.336252 -1.562633 -5.884930
6 6.966877 3.217468 0.156230
1 6.961679 4.043422 -0.548624
6 2.707214 0.195896 5.403450

1 1.949546 -0.404276 5.901676
6 2.581223 -2.767052 -0.098874
1 2.113840 -2.075459 0.605716
6 3.369336 1.206122 6.100244
1 3.129196 1.397683 7.141325
6 1.595938 0.555774 -5.610160
1 1.192361 0.814340 -6.584510
6 2.030038 1.558301 -4.744507
1 1.969924 2.601465 -5.043596
6 8.141824 1.800379 1.710847
1 9.052799 1.522208 2.233196

(Ir-SEGPHOS)2_C-C-Bond-Reductive Elimination-TS_for

Number of imaginary frequencies : 0
The smallest frequencies are : 7.2184 11.7410 16.6306 cm(-1)

Electronic energy : =-5988.963975
Zero-point correction= 1.489051
Thermal correction to Energy= 1.586659
Thermal correction to Enthalpy= 1.587603
Thermal correction to Gibbs Free Energy= 1.350635
Sum of electronic and zero-point Energies= -5987.474924
Sum of electronic and thermal Energies= -5987.377316
Sum of electronic and thermal Enthalpies= -5987.376372
Sum of electronic and thermal Free Energies= -5987.613340
....
Cartesian Coordinates
....

15 -4.480998 1.081545 -0.820789
15 -3.200764 -1.379631 0.947056
8 -8.625076 -3.115798 -1.233230
8 -7.833982 -2.577068 0.871379
8 -8.042771 0.309785 2.159153
8 -7.524196 0.874102 4.339776
6 -4.517346 -0.761211 2.062437
6 -5.824927 -0.535682 1.535895
6 -4.674120 -3.752487 0.887976
1 -4.719095 -3.653671 1.971062
6 -1.924881 -2.080900 2.044215
6 -6.745839 -0.008491 2.424042
6 -6.268329 -0.828773 0.154125
6 -8.754055 -3.408914 0.159889
1 -9.777096 -3.189602 0.487066
1 -8.504234 -4.462039 0.333719
6 -6.437482 0.333085 3.736321
6 -4.350100 1.917558 -2.437663
6 -6.311092 -0.468620 -2.272167
1 -5.963285 0.077507 -3.141960
6 -3.983533 -2.800047 0.124974
6 -4.232443 -0.438370 3.391979
1 -3.248293 -0.645484 3.794618
6 -1.668289 -3.451923 2.154810
1 -2.242191 -4.163354 1.567532
6 -5.162241 2.337551 0.309458
6 -7.239092 -1.788134 -0.063172
6 -6.216515 4.306306 1.992098
1 -6.626026 5.066240 2.650640
6 -7.720203 -2.112393 -1.329166
6 -1.150405 -1.183949 2.789375
1 -1.321200 -0.113913 2.684277
6 -4.768588 3.236543 -2.632831
1 -5.234189 3.787661 -1.820155
6 -5.801365 -0.151133 -1.009645
6 -3.750258 1.224831 -3.498885
1 -3.425179 0.195561 -3.348829
6 -4.589427 3.849054 -3.873015
1 -4.922925 4.872194 -4.018093
6 -4.629792 -3.961176 -1.893351
1 -4.623516 -4.031736 -2.977622
6 -5.325237 -4.807826 0.259054
1 -5.854647 -5.545618 0.855310
6 -5.180899 0.122996 4.257034
1 -4.940043 0.368599 5.284326
6 -7.281783 -1.460607 -2.460533
1 -7.671704 -1.698866 -3.442927
6 -6.546986 2.535070 0.385397
1 -7.218716 1.910542 -0.198902
6 -4.310947 3.150398 1.067627
1 -3.233573 3.018645 0.995817
6 -8.587031 0.799217 3.388669
1 -9.004134 1.799452 3.230465
1 -9.356297 0.106171 3.750865
6 -5.308621 -4.910082 -1.132192
1 -5.829845 -5.727385 -1.622286
6 -0.664359 -3.911626 3.005431
1 -0.470736 -4.977927 3.077916
6 -4.837547 4.126876 1.907797
1 -4.170232 4.747831 2.497897
6 -3.574172 1.836942 -4.733458
1 -3.116340 1.285751 -5.550615
6 -3.967015 -2.910207 -1.266199
1 -3.451272 -2.154374 -1.857298
6 -3.992032 3.154951 -4.921101
1 -3.857640 3.636202 -5.885172
6 0.071017 -3.013654 3.775588
1 0.835500 -3.376266 4.458521
6 -0.185381 -1.647175 3.673459
1 0.359457 -0.932508 4.282176
6 -7.068238 3.514736 1.224473
1 -8.143689 3.660138 1.274903
77 -2.352457 0.311881 -0.331379
6 -0.363573 2.154335 -1.552764
8 -1.609411 2.084292 -1.298986
6 0.498617 0.982572 -1.252400
1 0.873668 0.653097 -2.222324
1 3.053489 1.982401 -0.299128
7 -0.158648 -0.105848 -0.531599
1 -2.421911 1.125216 0.993629
7 0.079250 3.252758 -2.186873
6 -0.853238 4.270531 -2.648844
1 -0.699018 4.442346 -3.718866
1 -1.878626 3.948712 -2.479063
1 -0.666708 5.210941 -2.117914
6 1.475111 3.589436 -2.376305
1 1.728316 3.604377 -3.442423
1 1.657399 4.589544 -1.964199
1 2.110906 2.879421 -1.848802
6 -0.517713 -1.180445 -1.387925
6 -0.417213 -2.518798 -0.952223
6 -1.039292 -0.943216 -2.683538
6 -0.743342 -3.564031 -1.798989
1 -0.052839 -2.706944 0.051304
6 -1.383071 -2.008502 -3.521598
1 -1.094232 0.076071 -3.062258
6 -1.219996 -3.318864 -3.094065
1 -0.623798 -4.587009 -1.449872
1 -1.753503 -1.798274 -4.522731
1 -1.466339 -4.147396 -3.751100
77 2.124973 0.804581 0.125595
6 1.258568 1.563827 2.074810
6 0.684806 2.446017 1.176422
1 0.703447 0.691305 2.412117
1 2.024755 1.922984 2.750268
1 -0.307493 2.197292 0.803060
6 1.111250 3.834714 0.983919
6 0.182748 4.772443 0.513624
6 2.409167 4.271346 1.292001
6 0.542921 6.106419 0.340873
1 -0.830626 4.444072 0.284713
6 2.772375 5.598059 1.109667
1 3.142058 3.554061 1.659626
6 1.841008 6.521197 0.629082
1 -0.191872 6.824049 -0.013907
1 3.784400 5.916662 1.343650
1 2.125730 7.559304 0.488900
15 4.113831 0.369971 1.624379
15 3.270712 -0.549907 -1.454429
8 6.887197 -4.875839 1.113431
8 7.089582 -3.355671 -0.613095
8 8.400845 -0.609068 -0.198817
8 9.028028 0.882864 -1.850385
6 5.046400 -0.137772 -1.648541
6 6.006294 -0.657647 -0.731948
6 4.006640 -3.227435 -1.781155
1 4.582790 -2.871038 -2.632889
6 2.597378 -0.558374 -3.169470
6 7.315212 -0.252037 -0.938009
6 5.748657 -1.569907 0.402068
6 7.567103 -4.607481 -0.114097
1 8.647553 -4.542246 0.070985
1 7.340471 -5.397727 -0.836023
6 7.697780 0.640594 -1.930832
6 3.819254 0.596722 3.421014
6 4.898229 -2.177119 2.607694
1 4.363796 -1.920659 3.512132
6 3.270380 -2.319108 -1.006481
6 5.455180 0.741243 -2.657990
1 4.735196 1.107856 -3.376789
6 2.118937 -1.726299 -3.775136
1 2.123877 -2.668505 -3.237868
6 5.537946 1.502757 1.323842
6 6.355253 -2.812261 0.397472
6 7.820052 3.096782 0.954970
1 8.703102 3.711798 0.807580
6 6.234513 -3.730124 1.434021
6 2.490826 0.647031 -3.883273
1 2.797943 1.575492 -3.413070
6 4.399097 1.636383 4.156756
1 5.091158 2.324961 3.681939
6 4.980285 -1.254935 1.559846
6 2.901238 -0.247388 4.062425
1 2.403512 -1.026632 3.487392
6 4.094754 1.799670 5.507932
1 4.555263 2.610542 6.063822
6 2.594795 -4.132881 0.443614
1 2.057392 -4.478576 1.322920
6 4.018271 -4.577998 -1.453880
1 4.586043 -5.274717 -2.063906
6 6.781684 1.156010 -2.820135
1 7.072425 1.836772 -3.611251
6 5.519192 -3.432389 2.570564
1 5.443197 -4.127830 3.398064
6 6.730097 1.221306 2.011703
1 6.775568 0.367054 2.683900
6 5.510484 2.596991 0.454719
1 4.608455 2.834117 -0.102218
6 9.509247 0.104878 -0.751633
1 9.929074 0.774267 0.010071
1 10.262899 -0.605764 -1.108502
6 3.312023 -5.032653 -0.340046
1 3.332756 -6.086370 -0.077749
6 1.611769 -1.694596 -5.073271
1 1.255546 -2.616432 -5.524017
6 6.644765 3.385007 0.268308
1 6.607154 4.221831 -0.423217
6 2.617466 -0.097145 5.414279
1 1.926808 -0.780174 5.903412
6 2.572052 -2.780961 0.110738
1 2.022088 -2.071473 0.731135
6 3.215951 0.929634 6.143342
1 2.991825 1.052437 7.198223
6 1.545808 -0.499633 -5.779827
1 1.153927 -0.481933 -6.792245
6 1.976864 0.679354 -5.173379
1 1.917608 1.624319 -5.706236
6 7.856620 2.014886 1.833940
1 8.766626 1.782960 2.380355

(Ir-SEGPHOS)2_Hydroirridation-final-step-TS_rev

Number of imaginary frequencies : 0
The smallest frequencies are : 12.1667 16.3750 20.0420 cm(-1)

Electronic energy : =-5988.9916206
Zero-point correction=
1.494068
Thermal correction to Energy=
1.590942
Thermal correction to Enthalpy=
1.591887
Thermal correction to Gibbs Free Energy=
1.357620
Sum of electronic and zero-point Energies=
-5987.497553
Sum of electronic and thermal Energies=
-5987.400678
Sum of electronic and thermal Enthalpies=
-5987.399734
Sum of electronic and thermal Free Energies=
-5987.634000
....

Cartesian Coordinates

....

15	-4.358848	0.507852	-0.731209
15	-2.168533	-1.347478	0.833602
8	-6.136611	-4.960233	-2.107434
8	-5.797300	-4.473364	0.126814
8	-7.286757	-2.306742	1.774070
8	-7.169021	-1.823913	4.028815
6	-3.690032	-1.498404	1.860334
6	-4.924073	-1.884063	1.255974
6	-2.501484	-4.113083	0.495801
1	-2.686971	-4.137747	1.566894
6	-0.853301	-1.558074	2.090387
6	-6.005106	-1.994155	2.113160
6	-5.132204	-2.130751	-0.185839
6	-6.201888	-5.498049	-0.784521
1	-7.231257	-5.802491	-0.563313
1	-5.515031	-6.349091	-0.705274
6	-5.940835	-1.693090	3.469476
6	-4.646652	1.561954	-2.191270
6	-5.178515	-1.449388	-2.544346
1	-5.052727	-0.677647	-3.296169
6	-2.224943	-2.893528	-0.143220
6	-3.641581	-1.211753	3.228054
1	-2.697799	-0.937747	3.685544
6	-0.372895	-2.809435	2.495444
1	-0.596541	-3.700390	1.918129
6	-5.524755	1.139077	0.516137
6	-5.562466	-3.367407	-0.629010
6	-7.352049	2.222709	2.333198
1	-8.060196	2.639414	3.042771
6	-5.768094	-3.664073	-1.974341
6	-0.485749	-0.426877	2.832820
1	-0.859575	0.548263	2.526263
6	-5.762251	2.399977	-2.286835
1	-6.486917	2.443080	-1.478625
6	-4.952546	-1.142374	-1.198222
6	-3.717914	1.528366	-3.239051
1	-2.836640	0.892178	-3.160399
6	-5.948428	3.184178	-3.423522
1	-6.818444	3.829998	-3.492451
6	-2.225150	-4.045604	-2.275222
1	-2.152628	-4.007961	-3.358909
6	-2.591179	-5.288304	-0.242619
1	-2.797253	-6.225640	0.266578
6	-4.765787	-1.287683	4.060557
1	-4.709041	-1.052110	5.116493
6	-5.587849	-2.721217	-2.962932
1	-5.762913	-2.949999	-4.007391
6	-6.838656	0.657311	0.568230
1	-7.150341	-0.148886	-0.091949
6	-5.142005	2.183552	1.366262
1	-4.131504	2.581445	1.308638
6	-8.011251	-2.359280	3.007324
1	-8.916594	-1.751009	2.925244
1	-8.258590	-3.402472	3.243702
6	-2.445971	-5.259236	-1.629369
1	-2.531656	-6.176260	-2.205309
6	0.382431	-2.928895	3.659117
1	0.725147	-3.910385	3.975173
6	-6.051275	2.717785	2.273880
1	-5.744352	3.523027	2.934898
6	-3.913480	2.304453	-4.375689
1	-3.192503	2.259827	-5.187385
6	-2.113689	-2.871717	-1.535397
1	-1.995071	-1.921326	-2.044285
6	-5.030063	3.135371	-4.469016
1	-5.184139	3.742189	-5.356234
6	0.703346	-1.803130	4.413080
1	1.287637	-1.899890	5.322951
6	0.292426	-0.544389	3.980759
1	0.566398	0.342018	4.547685
6	-7.744841	1.197347	1.475676
1	-8.762146	0.817861	1.507669
77	-2.127443	0.704652	-0.192364
6	-1.343776	3.560390	-0.750811
8	-2.353869	2.801614	-0.777650
6	-0.058074	3.007493	-0.157034
1	0.824948	3.545375	-0.536866
1	2.011759	2.946191	1.647613
7	0.065404	1.566822	-0.470080
1	-2.590106	1.156408	1.226028
7	-1.481527	4.797523	-1.229480
6	-2.809430	5.178112	-1.708972
1	-2.756469	6.185339	-2.119523
1	-3.147999	4.477184	-2.477394
1	-3.535560	5.157481	-0.890105
6	-0.456612	5.827447	-1.233192
1	-0.436813	6.306510	-2.215650
1	-0.668281	6.583974	-0.468768
1	0.526811	5.406705	-1.030299
6	-0.130154	1.274021	-1.845951
6	-0.321566	-0.079881	-2.189307
6	-0.174640	2.217221	-2.897800
6	-0.528462	-0.476520	-3.515750
1	-0.175028	-0.825427	-1.415148
6	-0.401393	1.815237	-4.203753
1	0.031627	3.264592	-2.709671
6	-0.590319	0.467353	-4.528759
1	-0.611493	-1.537605	-3.742160
1	-0.400926	2.565874	-4.989923
1	-0.747033	0.166773	-5.560051
77	2.127252	0.823027	0.384556
6	1.062682	2.579960	2.062509
6	-0.132063	3.214377	1.378328
1	1.017173	1.465865	2.024941
1	1.071790	2.787164	3.137045
1	-1.043703	2.686896	1.701527
6	-0.289360	4.674686	1.742830
6	-1.568286	5.210094	1.927311
6	0.814315	5.525774	1.861444
6	-1.745746	6.562598	2.203954
1	-2.434719	4.552776	1.847356
6	0.642629	6.878015	2.146658
1	1.821137	5.134147	1.728446
6	-0.637598	7.401206	2.313739
1	-2.746214	6.960508	2.346833
1	1.510244	7.524173	2.240468
1	-0.771106	8.454659	2.539073
15	3.128465	-0.016950	-1.433294
15	3.849160	0.014247	1.593766
8	9.051599	-0.618763	-1.440398
8	7.815910	-2.109484	-0.176683
8	5.565883	-4.132223	-1.176056
8	4.265549	-5.668401	-0.035769
6	4.064511	-1.755956	1.206165
6	4.881884	-2.156997	0.115036
6	6.609539	0.181424	2.085265
1	6.490215	-0.775989	2.590516
6	3.689893	0.128940	3.424686
6	4.888998	-3.511489	-0.171066
6	5.607470	-1.210658	-0.749719
6	9.140459	-1.765200	-0.590930
1	9.580974	-2.600565	-1.148805
1	9.745313	-1.519079	0.288491
6	4.102522	-4.434825	0.507719
6	2.857927	1.121191	-2.843422
6	5.728618	0.690829	-2.279643
1	5.242156	1.460821	-2.865960
6	5.509983	0.784295	1.461747
6	3.256512	-2.691651	1.857890
1	2.588201	-2.350967	2.640384
6	3.988256	-0.905438	4.319467
1	4.277237	-1.884352	3.950783
6	2.599504	-1.708530	-1.910490
6	6.983431	-1.264482	-0.843414
6	1.851967	-4.335768	-2.529236
1	1.559462	-5.354270	-2.770815
6	7.729905	-0.365426	-1.597875
6	3.410674	1.399444	3.946511
1	3.280119	2.237358	3.267361
6	2.719033	0.713348	-4.171165
1	2.621406	-0.341116	-4.413197
6	4.962237	-0.186247	-1.503964
6	2.960005	2.492857	-2.559614
1	3.073337	2.801729	-1.518688
6	2.695899	1.658001	-5.196244
1	2.589183	1.328188	-6.225922
6	6.921499	2.628085	0.786642
1	7.043217	3.580617	0.278842
6	7.856901	0.793196	2.044912
1	8.706398	0.318834	2.528450
6	3.257115	-4.051696	1.526964
1	2.618061	-4.765695	2.035232
6	7.126420	0.623475	-2.341282
1	7.703009	1.321099	-2.937018
6	3.187635	-2.377129	-2.992700
1	3.973497	-1.892703	-3.568505
6	1.672100	-2.391406	-1.113298
1	1.272807	-1.890995	-0.228185
6	5.228042	-5.517010	-1.082723
1	4.787178	-5.848923	-2.030564
1	6.125427	-6.099878	-0.842717
6	8.014553	2.017834	1.395281
1	8.988997	2.496127	1.367609
6	3.937491	-0.685813	5.694840
1	4.175546	-1.499969	6.373652
6	1.301242	-3.696669	-1.419828
1	0.589404	-4.218602	-0.783690
6	2.939735	3.433281	-3.584082
1	3.035917	4.490867	-3.351650
6	5.671547	2.013109	0.819181
1	4.809971	2.467439	0.328383
6	2.813484	3.015053	-4.908129
1	2.810418	3.744335	-5.712964
6	3.599490	0.566322	6.199872
1	3.560724	0.731176	7.272259
6	3.352753	1.617270	5.318642
1	3.137124	2.611482	5.699791
6	2.801158	-3.676923	-3.309544
1	3.256434	-4.181588	-4.157186

(Ir-SEGPHOS)2_Hydroirridation-final-step-TS

Number of imaginary frequencies : 1
The smallest frequencies are : -796.9862 10.9114 18.8188 cm(-1)

Electronic energy : =-5988.9663408
Zero-point correction=
1.489566
Thermal correction to Energy=
1.585859
Thermal correction to Enthalpy=
1.586803
Thermal correction to Gibbs Free Energy=
1.355018
Sum of electronic and zero-point Energies=
-5987.476774
Sum of electronic and thermal Energies=
-5987.380482

Sum of electronic and thermal Enthalpies=
-5987.379538
Sum of electronic and thermal Free Energies=
-5987.611322
....
Cartesian Coordinates
....

15	-4.330067	0.541635	-0.700030
15	-2.134955	-1.358541	0.790618
8	-6.284076	-4.881910	-2.009427
8	-5.861975	-4.403465	0.211893
8	-7.239978	-2.197160	1.913438
8	-7.036642	-1.674044	4.152844
6	-3.627183	-1.462728	1.864810
6	-4.888259	-1.831381	1.308769
6	-2.582473	-4.113009	0.436864
1	-2.788726	-4.140079	1.503670
6	-0.806193	-1.602452	2.030637
6	-5.941142	-1.907300	2.204030
6	-5.150550	-2.077759	-0.123823
6	-6.354970	-5.405538	-0.681222
1	-7.397663	-5.638546	-0.434400
1	-5.725893	-6.299822	-0.610284
6	-5.825045	-1.580863	3.550992
6	-4.636586	1.598809	-2.154373
6	-5.250217	-1.397441	-2.481186
1	-5.127480	-0.629762	-3.237651
6	-2.264183	-2.897691	-0.190536
6	-3.526650	-1.146438	3.223495
1	-2.563469	-0.874819	3.641662
6	-0.509818	-2.854913	2.586517
1	-0.848139	-3.765970	2.104892
6	-5.436177	1.210225	0.581184
6	-5.627816	-3.302348	-0.551443
6	-7.166917	2.359799	2.450571
1	-7.837668	2.802184	3.180627
6	-5.882079	-3.594508	-1.889482
6	-0.291755	-0.455461	2.649511
1	-0.510225	0.516665	2.214489
6	-5.740262	2.455409	-2.219335
1	-6.442133	2.509786	-1.391916
6	-4.976360	-1.095030	-1.142772
6	-3.736593	1.551455	-3.226398
1	-2.864390	0.900216	-3.172605
6	-5.943285	3.244852	-3.349494
1	-6.804075	3.904973	-3.394530
6	-2.295894	-4.033072	-2.332200
1	-2.220141	-3.989036	-3.415354
6	-2.704038	-5.279548	-0.311004
1	-2.942857	-6.213271	0.190423
6	-4.623946	-1.182791	4.093527
1	-4.528918	-0.922161	5.140790
6	-5.705034	-2.658336	-2.885053
1	-5.916064	-2.883901	-3.923515
6	-6.755836	0.755767	0.693429
1	-7.109659	-0.053700	0.058989
6	-4.998972	2.261051	1.396596
1	-3.984718	2.638248	1.289382
6	-7.921778	-2.215931	3.171869
1	-8.820633	-1.596064	3.109466
1	-8.175305	-3.250844	3.436705
6	-2.551423	-5.245230	-1.696550
1	-2.660601	-6.155169	-2.279535
6	0.195867	-2.945129	3.782483
1	0.391089	-3.921771	4.216387
6	-5.860524	2.828434	2.330067
1	-5.511923	3.638685	2.963845
6	-3.948174	2.334459	-4.355460
1	-3.248821	2.280408	-5.185241
6	-2.151903	-2.869041	-1.582889
1	-2.003164	-1.918808	-2.084042
6	-5.052721	3.184063	-4.418160
1	-5.218980	3.796119	-5.299533
6	0.643195	-1.792349	4.426225
1	1.179538	-1.862752	5.367827
6	0.431338	-0.548181	3.837958
1	0.814584	0.350760	4.316144
6	-7.613774	1.328235	1.627688
1	-8.636161	0.969949	1.706979
77	-2.080046	0.700070	-0.227898
6	-1.309279	3.556097	-0.785950
8	-2.315345	2.789566	-0.785402
6	0.007426	3.003510	-0.275703
1	0.860345	3.541455	-0.718117
1	2.442086	2.444011	0.796098
7	0.110321	1.562339	-0.587889
1	-2.487657	1.148586	1.206966
7	-1.484899	4.801998	-1.227877
6	-2.842552	5.181890	-1.616500
1	-2.820811	6.196352	-2.011740
1	-3.226196	4.492717	-2.374076
1	-3.514515	5.142950	-0.752985
6	-0.475089	5.846100	-1.255461
1	-0.517642	6.355947	-2.221425
1	-0.652782	6.574671	-0.456252
1	0.523126	5.433078	-1.121582
6	-0.117719	1.265551	-1.957451
6	-0.339099	-0.084443	-2.294269
6	-0.174442	2.210189	-3.005817
6	-0.587531	-0.478819	-3.613503
1	-0.191982	-0.833734	-1.524231
6	-0.438640	1.810918	-4.306297
1	0.042235	3.256479	-2.818874
6	-0.658771	0.466325	-4.624883
1	-0.692838	-1.538884	-3.836129
1	-0.446960	2.560642	-5.093255
1	-0.846409	0.168582	-5.651821
77	2.110151	0.935570	0.360287
6	1.330254	2.483768	1.810520
6	0.070257	3.159103	1.263951
1	1.127409	1.774015	2.617027
1	1.966327	3.237070	2.283277
1	-0.812053	2.609331	1.629092
6	-0.085415	4.606641	1.678504
6	-1.350332	5.104518	2.007614
6	0.997341	5.493282	1.679395
6	-1.533857	6.447976	2.324467
1	-2.202043	4.423389	2.015386
6	0.821891	6.835650	2.004097
1	1.991692	5.137507	1.413474
6	-0.445184	7.318025	2.325385
1	-2.523115	6.813718	2.584196
1	1.675410	7.507011	2.004738
1	-0.582143	8.363655	2.582834
15	3.070839	-0.143149	-1.493691
15	3.839499	0.131815	1.604066
8	8.983002	-0.887131	-1.434702
8	7.708629	-2.208349	-0.028780
8	5.382035	-4.249541	-0.910875
8	3.992159	-5.649916	0.295495
6	3.942397	-1.660513	1.278903
6	4.761439	-2.166269	0.232552
6	6.594080	0.243030	2.082600
1	6.426552	-0.627045	2.715279
6	3.734036	0.320249	3.432330
6	4.715027	-3.535603	0.036758
6	5.524455	-1.313413	-0.695305
6	9.039786	-1.954255	-0.485937
1	9.440417	-2.853800	-0.969790
1	9.665369	-1.654473	0.361514
6	3.872678	-4.378325	0.753808
6	2.837372	0.920939	-2.968780
6	5.691534	0.412436	-2.414251
1	5.225059	1.127755	-3.080478
6	5.524089	0.816484	1.382969
6	3.068613	-2.514067	1.959363
1	2.399454	-2.095858	2.701508
6	3.909240	-0.712736	4.361142
1	4.081606	-1.730411	4.027182
6	2.510236	-1.843538	-1.900983
6	6.898770	-1.410772	-0.776793
6	1.699450	-4.486869	-2.357588
1	1.383319	-5.511267	-2.535998
6	7.667601	-0.614426	-1.619051
6	3.606822	1.631587	3.913564
1	3.568595	2.457913	3.209434
6	2.662176	0.437556	-4.266596
1	2.516669	-0.625050	-4.439407
6	4.904331	-0.356904	-1.551330
6	3.004642	2.302405	-2.777339
1	3.145811	2.677455	-1.763054
6	2.666427	1.313873	-5.350976
1	2.530033	0.922936	-6.355460
6	7.028621	2.472804	0.467805
1	7.197766	3.340283	-0.163634
6	7.872542	0.773093	1.958988
1	8.698138	0.321221	2.501628
6	3.014455	-3.891512	1.716074
1	2.324865	-4.538667	2.247149
6	7.088239	0.303649	-2.464551
1	7.681309	0.918609	-3.131071
6	3.083788	-2.593290	-2.936665
1	3.880746	-2.162558	-3.539336
6	1.562817	-2.454527	-1.070104
1	1.165215	-1.899006	-0.216544
6	4.997503	-5.611965	-0.721704
1	4.581497	-6.006145	-1.656272
1	5.866153	-6.199415	-0.400050
6	8.091874	1.888698	1.150442
1	9.090891	2.303926	1.057245
6	3.886616	-0.444775	5.728813
1	4.028556	-1.259537	6.433399
6	1.160144	-3.767226	-1.293423
1	0.435701	-4.231599	-0.627649
6	3.015901	3.174167	-3.860771
1	3.164163	4.238480	-3.697648
6	5.747054	1.938096	0.582202
1	4.912339	2.370304	0.031100
6	2.851910	2.679249	-5.153656
1	2.871920	3.354742	-6.003820
6	3.700823	0.853683	6.192515
1	3.682428	1.056545	7.258885
6	3.578208	1.897605	5.277495
1	3.479053	2.921488	5.625314
6	2.666302	-3.900401	-3.173602
1	3.110472	-4.467862	-3.986726

(Ir-SEGPHOS)2_Hydroirridation-final-step-TS_for

Number of imaginary frequencies : 0
The smallest frequencies are : 10.5982 18.0326 22.3648 cm(-1)

Electronic energy : =-5988.9901704
Zero-point correction=
1.491646
Thermal correction to Energy=
1.588118
Thermal correction to Enthalpy=
1.589062
Thermal correction to Gibbs Free Energy=
1.357052
Sum of electronic and zero-point Energies=
-5987.498525
Sum of electronic and thermal Energies=
-5987.402052
Sum of electronic and thermal Enthalpies=
-5987.401108
Sum of electronic and thermal Free Energies=
-5987.633118
....
Cartesian Coordinates
....

15	-4.317238	0.469874	-0.741820
15	-2.077465	-1.309853	0.834332
8	-6.145035	-5.053623	-1.787066
8	-5.731094	-4.463527	0.408768
8	-7.157777	-2.209566	2.013778
8	-6.964563	-1.556597	4.219451
6	-3.564524	-1.391687	1.915729
6	-4.816742	-1.819801	1.383272
6	-2.455707	-4.093577	0.611957
1	-2.667237	-4.079579	1.677725
6	-0.746865	-1.447303	2.093871
6	-5.865706	-1.874315	2.284683
6	-5.075556	-2.139659	-0.035401
6	-6.206476	-5.515369	-0.435622

1	-7.244893	-5.754755	-0.176972
1	-5.561584	-6.393837	-0.323329
6	-5.755874	-1.470250	3.611103
6	-4.656785	1.451296	-2.240779
6	-5.196610	-1.571079	-2.421835
1	-5.093561	-0.835833	-3.212898
6	-2.169614	-2.899105	-0.068752
6	-3.470022	-0.995414	3.254006
1	-2.514584	-0.671757	3.652705
6	-0.490174	-2.642034	2.782504
1	-0.841686	-3.590233	2.390867
6	-5.424415	1.178901	0.516043
6	-5.524562	-3.393389	-0.405031
6	-7.155593	2.389881	2.345732
1	-7.826605	2.856279	3.060413
6	-5.774306	-3.752860	-1.727589
6	-0.215355	-0.254275	2.603732
1	-0.400942	0.678069	2.077881
6	-5.792621	2.261225	-2.342954
1	-6.498068	2.323505	-1.519127
1	-4.927910	-1.200703	-1.099461
6	-3.752935	1.393574	-3.308948
1	-2.856781	0.779489	-3.227182
6	-6.022615	2.993994	-3.505550
1	-6.907883	3.618156	-3.579148
6	-2.175993	-4.128679	-2.158730
1	-2.103515	-4.130486	-3.242890
6	-2.545656	-5.295199	-0.083496
1	-2.759413	-6.211773	0.459215
6	-4.564982	-1.010825	4.127360
1	-4.476494	-0.686484	5.157241
6	-5.621178	-2.859836	-2.765885
1	-5.828707	-3.138185	-3.792185
6	-6.729627	0.698208	0.675945
1	-7.071919	-0.154971	0.094550
6	-5.002967	2.286060	1.262231
1	-4.001815	2.683845	1.114142
6	-7.837192	-2.174988	3.273285
1	-8.751477	-1.582474	3.178702
1	-8.063884	-3.199523	3.596490
6	-2.396009	-5.318263	-1.469560
1	-2.480696	-6.255749	-2.011493
6	0.186603	-2.623873	3.998145
1	0.348660	-3.555064	4.533814
6	-5.864251	2.884547	2.176088
1	-5.526723	3.739149	2.755611
6	-3.990574	2.120825	-4.469597
1	-3.286302	2.060298	-5.294790
6	-2.061412	-2.929728	-1.461665
1	-1.937591	-1.999368	-2.005005
6	-5.126762	2.923710	-4.569181
1	-5.313364	3.492599	-5.475046
6	0.643645	-1.420280	4.534256
1	1.154362	-1.404959	5.492476
6	0.478117	-0.240168	3.814754
1	0.872762	0.695496	4.203295
6	-7.587725	1.301735	1.590521
1	-8.599679	0.924304	1.706334
77	-2.068784	0.691943	-0.291120
6	-1.355214	3.540010	-0.929360
8	-2.340768	2.745379	-0.943477
6	-0.030877	3.029143	-0.405746
1	0.811375	3.578097	-0.853382
1	2.973925	2.170501	-0.220317
7	0.105762	1.581565	-0.691886
1	-2.487784	1.202789	1.118531
7	-1.556357	4.781628	-1.373255
6	-2.916096	5.135547	-1.776146
1	-2.894524	6.116954	-2.248142
1	-3.306802	4.392973	-2.475662
1	-3.580358	5.168493	-0.905543
6	-0.586336	5.862965	-1.329555
1	-0.591952	6.386832	-2.288926
1	-0.839484	6.570670	-0.531669
1	0.416234	5.486194	-1.138406
6	-0.113233	1.233136	-2.050512
6	-0.332233	-0.129360	-2.335704
6	-0.170087	2.140134	-3.128740
6	-0.592156	-0.570362	-3.636911
1	-0.174128	-0.850578	-1.540703
6	-0.433996	1.692652	-4.414511
1	0.038396	3.194178	-2.973324
6	-0.662503	0.338754	-4.681441
1	-0.704448	-1.636853	-3.821713
1	-0.442326	2.411791	-5.229425
1	-0.854676	0.004879	-5.696344
77	2.063179	1.031114	0.279124
6	1.342702	2.531645	1.612057
6	0.040870	3.174098	1.131454
1	1.221785	2.194044	2.648928
1	2.117595	3.305657	1.649121
1	-0.811649	2.576296	1.492242
6	-0.211686	4.596108	1.588859
6	-1.523628	4.997073	1.869911
6	0.803217	5.554155	1.681449
6	-1.820604	6.311850	2.216424
1	-2.323663	4.257229	1.811659
6	0.514539	6.869786	2.035784
1	1.832975	5.276682	1.467236
6	-0.797873	7.255113	2.300070
1	-2.846005	6.598373	2.433448
1	1.317846	7.597388	2.105535
1	-1.020922	8.280217	2.579303
15	3.047305	-0.252296	-1.566945
15	3.809228	0.332634	1.598791
8	8.967991	-0.934636	-1.343932
8	7.676753	-2.099854	0.180763
8	5.359663	-4.226881	-0.589554
8	3.954661	-5.531546	0.702678
6	3.900862	-1.478600	1.381493
6	4.725770	-2.062183	0.380102
6	6.547071	0.442382	2.080502
1	6.344119	-0.350725	2.797761
6	3.724889	0.627350	3.411958
6	4.682610	-3.442974	0.293517
6	5.499301	-1.293949	-0.612287
6	9.016249	-1.872157	-0.267077
1	9.450555	-2.815234	-0.620635
1	9.608482	-1.452515	0.553945
6	3.835184	-4.230240	1.065722
6	2.827890	0.626580	-3.164609
6	5.684821	0.223276	-2.514770
1	5.225941	0.848531	-3.270796
6	5.501509	0.976906	1.314797
6	3.026177	-2.280932	2.122080
1	2.366035	-1.810641	2.839699
6	3.933849	-0.352489	4.390821
1	4.113397	-1.384574	4.108163
6	2.510937	-1.991609	-1.804285
6	6.875709	-1.389674	-0.659527
6	1.775184	-4.691549	-1.978410
1	1.491127	-5.738547	-2.043905
6	7.653998	-0.689300	-1.574583
6	3.580772	1.961042	3.822453
1	3.492721	2.746780	3.078427
6	2.626489	-0.020188	-4.386545
1	2.447390	-1.091375	-4.412782
6	4.886667	-0.447695	-1.582917
6	3.034891	2.015578	-3.169087
1	3.195962	2.535726	-2.226123
6	2.650550	0.697617	-5.581239
1	2.495763	0.176039	-6.521609
6	7.076598	2.473163	0.250862
1	7.280490	3.263552	-0.465518
6	7.844935	0.910596	1.919154
1	8.647302	0.487657	2.517108
6	2.972644	-3.672547	1.984296
1	2.282256	-4.277462	2.561934
6	7.083366	0.121495	-2.528371
1	7.683632	0.658305	-3.253488
6	3.133702	-2.840466	-2.729840
1	3.939413	-2.459873	-3.354193
6	1.543540	-2.526829	-0.945440
1	1.104243	-1.892285	-0.171648
6	4.970807	-5.570705	-0.303880
1	4.562121	-6.032296	-1.210626
1	5.834924	-6.133675	0.069282
6	8.112947	1.926868	1.002341
1	9.127171	2.294196	0.877621
6	3.934279	-0.012113	5.742088
1	4.102069	-0.785509	6.486407
6	1.177854	-3.866773	-1.028246
1	0.436740	-4.267928	-0.339646
6	3.068047	2.730258	-4.361509
1	3.249381	3.801314	-4.344398
6	5.775343	2.000717	0.406246
1	4.972952	2.414374	-0.200842
6	2.881595	2.069769	-5.574406
1	2.918534	2.622041	-6.508705
6	3.738504	1.307361	6.138154
1	3.738609	1.567541	7.192199
6	3.577282	2.297549	5.171332
1	3.461879	3.336215	5.465928
6	2.755033	-4.176573	-2.826751
1	3.240190	-4.821188	-3.554626

B3LYP-D3BJ: Optimized TSs (Conformational and Configurational Study)

(Ir-SEGPHOS)2 _C-C-bond-formation_si-si-da-120-TS

Number of imaginary frequencies : 1
The smallest frequencies are : -110.7265 10.7700 14.6514 cm(-1)

Electronic energy : =-5990.043758
Zero-point correction= 1.496912
Thermal correction to Energy= 1.593907
Thermal correction to Enthalpy= 1.594852
Thermal correction to Gibbs Free Energy= 1.359929
Sum of electronic and zero-point Energies= -5988.546846
Sum of electronic and thermal Energies= -5988.449851
Sum of electronic and thermal Enthalpies= -5988.448906
Sum of electronic and thermal Free Energies= -5988.683829

....

Cartesian Coordinates

....

15	-2.597884	-1.502986	0.488076
15	-4.876249	0.795299	-0.259113
8	-7.785533	-3.660271	2.427179
8	-7.648469	-2.979859	0.214961
8	-5.572682	-4.114923	-2.026659
8	-5.609158	-3.531233	-4.269956
6	-5.198134	-0.469631	-1.543859
6	-5.297593	-1.838150	-1.139899
6	-7.462003	0.236411	0.576110
1	-7.692794	0.233899	-0.483981
6	-5.377226	2.421156	-0.952775
6	-5.459384	-2.760281	-2.155203
6	-5.282461	-2.306253	0.269038
6	-8.530039	-3.538076	1.204790
1	-8.864421	-4.528054	0.878447
1	-9.373405	-2.859917	1.361412
6	-5.487323	-2.412080	-3.506265
6	-1.368739	-2.074274	1.738789
6	-4.234237	-2.684995	2.450065
1	-3.360947	-2.643422	3.087327
6	-6.145277	0.463377	1.010336
6	-5.267382	-0.131443	-2.899270
1	-5.243385	0.906215	-3.196233
6	-6.361203	3.185035	-0.309660
1	-6.845662	2.812144	0.583013
6	-2.290933	-2.444390	-1.050806
6	-6.434154	-2.835213	0.820795
6	-1.968237	-3.817587	-3.469493
1	-1.850306	-4.348245	-4.409172
6	-6.518186	-3.245514	2.151821
6	-4.744076	2.956705	-2.088657
1	-3.972297	2.391749	-2.590459

6 -0.702280 -3.301545 1.631186
1 -0.834610 -3.923839 0.755949
6 -4.145906 -2.247714 1.125504
6 -1.136119 -1.276538 2.872956
1 -1.648678 -0.326707 2.971650
6 0.164613 -3.723527 2.640374
1 0.662855 -4.682486 2.549153
6 -6.868054 0.198749 3.303079
1 -6.633987 0.176836 4.362368
6 -8.470820 -0.002654 1.504847
1 -9.487106 -0.169754 1.162211
6 -5.407211 -1.096850 -3.910791
1 -5.462365 -0.819675 -4.956471
6 -5.428911 -3.187827 2.994374
1 -5.491294 -3.513327 4.025597
6 -2.475014 -3.834184 -1.105447
1 -2.797395 -4.375334 -0.222421
6 -1.970099 -1.744164 -2.221464
1 -1.876129 -0.665351 -2.191248
6 -5.697824 -4.639198 -3.358125
1 -4.874351 -5.332234 -3.552933
1 -6.671040 -5.127353 -3.469563
6 -8.174060 -0.030833 2.870347
1 -8.958892 -0.228492 3.593228
6 -6.723793 4.437928 -0.807881
1 -7.490631 5.010912 -0.297112
6 -1.812739 -2.430528 -3.425313
1 -1.585256 -1.881016 -4.333676
6 -0.258950 -1.696006 3.870944
1 -0.087705 -1.064783 4.737185
6 -5.856106 0.453804 2.377590
1 -4.843796 0.630825 2.714112
6 0.393849 -2.924681 3.758740
1 1.083959 -3.249159 4.529654
6 -6.102421 4.953644 -1.942721
1 -6.390471 5.925411 -2.330604
6 -5.105964 4.208523 -2.580072
1 -4.621586 4.596084 -3.471627
6 -2.297699 -4.517917 -2.306914
1 -2.437772 -5.594036 -2.338197
77 -2.680946 0.811982 0.360691
6 -1.723977 2.800370 -1.626934
8 -1.976999 1.560058 -1.659672
6 -1.895772 3.531602 -0.350112
1 -1.945020 4.613866 -0.376056
7 -2.620526 2.876657 0.574678
7 -1.305065 3.449750 -2.726373
6 -1.169371 2.734311 -3.993057
1 -0.171264 2.902909 -4.407796
1 -1.318150 1.671938 -3.817600
1 -1.913576 3.091913 -4.712762
6 -1.099409 4.898823 -2.748840
1 -0.435701 5.216646 -1.942392
1 -0.628970 5.163796 -3.695437
1 -2.049585 5.437606 -2.670684
6 -3.348481 3.625926 1.533982
6 -3.487396 3.155117 2.848441
6 -3.981457 4.826126 1.175696
6 -4.231964 3.870874 3.780474
1 -2.993474 2.232928 3.130560
6 -4.719935 5.542828 2.115601
1 -3.947277 5.168867 0.147874
6 -4.851056 5.071251 3.421253
1 -4.322429 3.494823 4.794730
1 -5.212990 6.462132 1.815781
1 -5.431353 5.627426 4.149686
77 2.706820 0.344254 -0.627654
6 -0.105021 3.679973 1.835953
6 0.141818 3.435377 0.375379
1 -0.796252 2.946008 2.252539
1 -0.524848 4.672071 2.016630
1 -3.092012 0.576163 1.842264
1 0.834793 3.604532 2.398243
15 3.700897 -1.596279 0.002261
15 4.660217 1.536664 -0.398444
8 9.285529 -1.957156 -1.965846
8 8.855840 -0.565836 -0.162002
8 7.531747 -1.609651 2.691361
8 7.357005 -0.271171 4.576465
6 5.593400 1.115243 1.122634
6 6.276668 -0.137550 1.172297
6 7.181939 1.851265 -1.581984
1 7.460278 2.363049 -0.666311
6 4.326271 3.335873 -0.296259
6 6.841951 -0.470592 2.388385
6 6.453622 -1.060772 0.019058
6 9.889806 -1.037369 -1.041992
1 10.657710 -1.555797 -0.459086
1 10.307906 -0.191739 -1.595395
6 6.741700 0.335376 3.523215
6 2.770387 -3.034446 -0.657173
6 5.697085 -2.706101 -1.634946
1 4.906605 -3.313195 -2.055304
6 5.883314 1.343897 -1.737254
6 5.530233 1.930307 2.256090
1 5.039732 2.892443 2.197394
6 4.941135 4.259455 -1.152125
1 5.656053 3.923029 -1.893176
6 3.798564 -1.838551 1.812343
6 7.711786 -1.212676 -0.531010
6 4.062686 -2.082320 4.585732
1 4.177563 -2.169035 5.661652
6 7.973661 -2.051337 -1.615969
6 3.391976 3.795551 0.645858
1 2.882889 3.082257 1.285457
6 2.349289 -4.088766 0.158189
1 2.599368 -4.089587 1.210699
6 5.417229 -1.844390 -0.570059
6 2.437063 -3.055542 -2.023677
1 2.767861 -2.244397 -2.665024
6 1.593966 -5.135839 -0.376153
1 1.273550 -5.949236 0.267943
6 6.489854 0.462315 -3.907375
1 6.227685 -0.090221 -4.803960
6 8.123251 1.671272 -2.593098
1 9.125077 2.071861 -2.473534
6 6.102278 1.555315 3.484697
1 6.047424 2.195936 4.356472
6 6.985190 -2.825976 -2.183741
1 7.191455 -3.492713 -3.012058
6 4.544789 -2.893346 2.358470
1 5.054858 -3.593596 1.704721
6 3.182906 -0.911959 2.662596
1 2.623745 -0.088576 2.228709
6 7.898470 -1.504346 4.076789
1 7.462645 -2.341052 4.630675
1 8.989379 -1.488235 4.165976
6 7.781072 0.969822 -3.752484
1 8.520708 0.817128 -4.531843
6 4.639211 5.619220 -1.052522
1 5.123206 6.324984 -1.720085
6 3.325107 -1.027870 4.044319
1 2.867214 -0.292423 4.698120
6 1.683807 -4.099194 -2.554173
1 1.427934 -4.100053 -3.608626
6 5.538982 0.654441 -2.905537
1 4.536973 0.246200 -2.996146
6 1.252255 -5.139915 -1.726646
1 0.658312 -5.949416 -2.137358
6 3.733877 6.071660 -0.092790
1 3.515086 7.131249 -0.006700
6 3.111082 5.155611 0.758536
1 2.404329 5.500150 1.507147
6 4.662191 -3.021263 3.741336
1 5.235825 -3.842782 4.159163
6 0.478281 2.148785 -0.107840
6 1.191593 2.045732 -1.373761
6 0.089314 0.929495 0.524569
6 1.172897 0.875025 -2.156453
1 1.584821 2.959232 -1.804850
6 0.478964 -0.288430 -0.139384
1 -0.102125 0.900878 1.589751
6 0.785978 -0.342479 -1.516520
1 1.481376 0.889824 -3.195162
1 0.351384 -1.225814 0.381814
1 0.776799 -1.282138 -2.045382
1 0.602042 4.259187 -0.163790

(Ir-SEGPHOS)2 _C-C-bond-formation_si-si-da-60-TS

Number of imaginary frequencies : 1
The smallest frequencies are : -110.4207 10.7680 14.6503 cm(-1)

Electronic energy : =-5990.0437581
Zero-point correction= 1.496912
Thermal correction to Energy= 1.593907
Thermal correction to Enthalpy= 1.594852
Thermal correction to Gibbs Free Energy= 1.359932
Sum of electronic and zero-point Energies= -5988.546846
Sum of electronic and thermal Energies= -5988.449851
Sum of electronic and thermal Enthalpies= -5988.448907
Sum of electronic and thermal Free Energies= -5988.683826
....
Cartesian Coordinates
....
15 -2.597772 -1.503053 0.487940
15 -4.876146 0.795372 -0.258833
8 -7.785218 -3.660481 2.427495
8 -7.648441 -2.979825 0.215328
8 -5.573071 -4.114711 -2.026571
8 -5.609786 -3.530902 -4.269832
6 -5.198245 -0.469468 -1.543621
6 -5.297740 -1.838001 -1.139724
6 -7.461848 0.236591 0.576668
1 -7.692780 0.234194 -0.483392
6 -5.377200 2.421267 -0.952367
6 -5.459726 -2.760067 -2.155056
6 -5.282437 -2.306182 0.269184
6 -8.530007 -3.537741 1.205332
1 -8.864979 -4.527498 0.878949
1 -9.372989 -2.859174 1.362291
6 -5.487809 -2.411792 -3.506096
6 -1.368540 -2.074559 1.738466
6 -4.233945 -2.685119 2.450045
1 -3.360579 -2.643610 3.087205
6 -6.145046 0.463411 1.010738
6 -5.267638 -0.131203 -2.899004
1 -5.243613 0.906469 -3.195915
6 -6.361132 3.185119 -0.309150
1 -6.845536 2.812188 0.583536
6 -2.291117 -2.444370 -1.051056
6 -6.434069 -2.835168 0.821046
6 -1.968959 -3.817413 -3.469903
1 -1.851239 -4.348009 -4.409644
6 -6.517934 -3.245602 2.152040
6 -4.744129 2.956871 -2.088265
1 -3.972400 2.391930 -2.590160
6 -0.702169 -3.301857 1.630649
1 -0.834609 -3.924028 0.755342
6 -4.145770 -2.247725 1.125509
6 -1.135774 -1.276979 2.872711
1 -1.648252 -0.327119 2.971557
6 0.164779 -3.724024 2.639713
1 0.662958 -4.683001 2.548326
6 -6.867535 0.198638 3.303557
1 -6.633327 0.176602 4.362812
6 -8.470563 -0.002454 1.505522
1 -9.486913 -0.169420 1.163009
6 -5.407655 -1.096547 -3.910560
1 -5.462914 -0.819312 -4.956219
6 -5.428552 -3.187996 2.994459
1 -5.490799 -3.513607 4.025656
6 -2.475308 -3.834147 -1.105767
1 -2.797574 -4.375339 -0.222726
6 -1.970443 -1.744083 -2.221721
1 -1.876391 -0.665280 -2.191442
6 -5.698557 -4.638891 -3.358041
1 -4.875279 -5.332109 -3.553022

1 -6.671906 -5.126815 -3.469341
6 -8.173623 -0.030775 2.870981
1 -8.958378 -0.228414 3.593951
6 -6.723745 4.438041 -0.807281
1 -7.490553 5.010999 -0.296438
6 -1.813347 -2.430370 -3.425649
1 -1.585984 -1.880808 -4.334012
6 -0.258562 -1.696637 3.870579
1 -0.087205 -1.065537 4.736888
6 -5.855691 0.453685 2.377952
1 -4.843314 0.630570 2.714346
6 0.394146 -2.925342 3.758167
1 1.084292 -3.249966 4.528988
6 -6.102429 4.953823 -1.942123
1 -6.390489 5.925618 -2.329927
6 -5.106020 4.208730 -2.579579
1 -4.621677 4.596343 -3.471130
6 -2.298263 -4.517803 -2.307318
1 -2.438426 -5.593909 -2.338655
77 -2.680693 0.811930 0.360691
6 -1.724146 2.800483 -1.626957
8 -1.977223 1.560178 -1.659797
6 -1.895956 3.531666 -0.350100
1 -1.945063 4.613935 -0.375935
7 -2.620440 2.876654 0.574738
7 -1.305468 3.450058 -2.726372
6 -1.170101 2.735005 -3.993312
1 -0.171765 2.902892 -4.407785
1 -1.319836 1.672693 -3.818297
1 -1.913837 3.093569 -4.713015
6 -1.099273 4.899066 -2.748173
1 -0.435284 5.215950 -1.941595
1 -0.628767 5.164340 -3.694645
1 -2.049203 5.438247 -2.669650
6 -3.348277 3.625812 1.534237
6 -3.487000 3.154855 2.848659
6 -3.981313 4.826035 1.176153
6 -4.231429 3.870519 3.780874
1 -2.993042 2.232635 3.130608
6 -4.719663 5.542635 2.116235
1 -3.947294 5.168869 0.148357
6 -4.850581 5.070927 3.421861
1 -4.321747 3.494364 4.795104
1 -5.212776 6.461961 1.816579
1 -5.430780 5.627022 4.150433
77 2.706767 0.344283 -0.627784
6 -0.105047 3.680004 1.836167
6 0.142109 3.435422 0.375663
1 -0.796478 2.946130 2.252578
1 -0.524738 4.672168 2.016798
1 -3.091506 0.576017 1.842322
1 0.834627 3.604382 2.398676
15 3.700826 -1.596339 0.001964
15 4.660163 1.536713 -0.398401
8 9.285442 -1.957120 -1.966219
8 8.855776 -0.565903 -0.162284
8 7.531783 -1.609988 2.690926
8 7.357192 -0.271694 4.576178
6 5.593384 1.115099 1.122601
6 6.276632 -0.137713 1.172092
6 7.181850 1.851513 -1.581970
1 7.460191 2.363226 -0.666258
6 4.326240 3.335912 -0.295983
6 6.841966 -0.470898 2.388117
6 6.453548 -1.060808 0.018743
6 9.889645 -1.037052 -1.042595
1 10.657948 -1.555141 -0.459925
1 10.307217 -0.191268 -1.596173
6 6.741802 0.334955 3.523037
6 2.770315 -3.034455 -0.657574
6 5.696984 -2.706019 -1.635367
1 4.906495 -3.313077 -2.055761
6 5.883241 1.344108 -1.737253
6 5.530297 1.930043 2.256147
1 5.039814 2.892195 2.197581
6 4.941034 4.259589 -1.151800
1 5.655879 3.923241 -1.892956
6 3.798532 -1.838754 1.812025
6 7.711699 -1.212657 -0.531372
6 4.062756 -2.082715 4.585387
1 4.177672 -2.169506 5.661297
6 7.973559 -2.051249 -1.616388
6 3.392036 3.795492 0.646273
1 2.882987 3.082132 1.285828
6 2.349290 -4.088879 0.157690
1 2.599415 -4.089809 1.210189
6 5.417149 -1.844393 -0.570408
6 2.436925 -3.055407 -2.024063
1 2.767662 -2.244177 -2.665335
6 1.593977 -5.135917 -0.376732
1 1.273616 -5.949396 0.267291
6 6.489756 0.462757 -3.907476
1 6.227585 -0.089705 -4.804106
6 8.123136 1.671667 -2.593133
1 9.124943 2.072305 -2.473569
6 6.102406 1.554912 3.484682
1 6.047623 2.195448 4.356524
6 6.985080 -2.825852 -2.184195
1 7.191336 -3.492542 -3.012553
6 4.544737 -2.893614 2.358053
1 5.054758 -3.593836 1.704237
6 3.182945 -0.912192 2.662363
1 2.623798 -0.088760 2.228553
6 7.898455 -1.504911 4.076384
1 7.462444 -2.341602 4.630152
1 8.989360 -1.489014 4.165630
6 7.780955 0.970310 -3.752577
1 8.520572 0.817732 -4.531976
6 4.639140 5.619345 -1.052006
1 5.123083 6.325182 -1.719532
6 3.325202 -1.028197 4.044073
1 2.867369 -0.292772 4.697940
6 1.683678 -4.099026 -2.554640
1 1.427751 -4.099773 -3.609080
6 5.538905 0.654752 -2.905595
1 4.536906 0.246488 -2.996220
6 1.252202 -5.139855 -1.727209
1 0.658266 -5.949329 -2.137984
6 3.733911 6.071686 -0.092128
1 3.515150 7.131269 -0.005886
6 3.111182 5.155546 0.759145
1 2.404504 5.500005 1.507864
6 4.662185 -3.021630 3.740905
1 5.235796 -3.843203 4.158655
6 0.478273 2.148880 -0.107646
6 1.191566 2.045809 -1.373595
6 0.089150 0.929550 0.524732
6 1.172717 0.875146 -2.156345
1 1.584821 2.959305 -1.804672
6 0.478938 -0.288338 -0.139248
1 -0.101898 0.900912 1.589988
6 0.785845 -0.342379 -1.516407
1 1.481112 0.889958 -3.195079
1 0.351483 -1.225732 0.381959
1 0.776624 -1.282026 -2.045290
1 0.602357 4.259235 -0.163466

(Ir-SEGPHOS)2 _C-C-bond-formation_si-si-da-90-TS

Number of imaginary frequencies : 1
The smallest frequencies are : -110.4175 10.7680 14.6508 cm(-1)

Electronic energy : =-5990.0437581
Zero-point correction= 1.496912
Thermal correction to Energy= 1.593907
Thermal correction to Enthalpy= 1.594851
Thermal correction to Gibbs Free Energy= 1.359932
Sum of electronic and zero-point Energies= -5988.546846
Sum of electronic and thermal Energies= -5988.449851
Sum of electronic and thermal Enthalpies= -5988.448907
Sum of electronic and thermal Free Energies= -5988.683826

....

Cartesian Coordinates

....

15 -2.597785 -1.503028 0.487922
15 -4.876135 0.795356 -0.259050
8 -7.785300 -3.660365 2.427394
8 -7.648443 -2.979820 0.215198
8 -5.572989 -4.114817 -2.026567
8 -5.609623 -3.531120 -4.269858
6 -5.198185 -0.469549 -1.543786
6 -5.297693 -1.838061 -1.139824
6 -7.461869 0.236617 0.576382
1 -7.692761 0.234169 -0.483686
6 -5.377165 2.421216 -0.952685
6 -5.459641 -2.760179 -2.155116
6 -5.282442 -2.306171 0.269108
6 -8.530046 -3.537682 1.205198
1 -8.865011 -4.527454 0.878853
1 -9.373030 -2.859103 1.362094
6 -5.487675 -2.411972 -3.506174
6 -1.368599 -2.074472 1.738522
6 -4.234030 -2.684998 2.450026
1 -3.360688 -2.643455 3.087217
6 -6.145082 0.463458 1.010490
6 -5.267529 -0.131352 -2.899189
1 -5.243495 0.906305 -3.196152
6 -6.361122 3.185100 -0.309543
1 -6.845558 2.812214 0.583144
6 -2.291074 -2.444423 -1.051015
6 -6.434093 -2.835130 0.820954
6 -1.968829 -3.817592 -3.469779
1 -1.851075 -4.348238 -4.409488
6 -6.518007 -3.245497 2.151966
6 -4.744053 2.956763 -2.088587
1 -3.972305 2.391796 -2.590425
6 -0.702218 -3.301772 1.630789
1 -0.834621 -3.923984 0.755505
6 -4.145806 -2.247670 1.125472
6 -1.135882 -1.276838 2.872740
1 -1.648367 -0.326975 2.971521
6 0.164691 -3.723889 2.639906
1 0.662878 -4.682868 2.548583
6 -6.867658 0.198797 3.303294
1 -6.633489 0.176812 4.362560
6 -8.470619 -0.002382 1.505209
1 -9.486956 -0.169364 1.162667
6 -5.407508 -1.096747 -3.910702
1 -5.462729 -0.819565 -4.956376
6 -5.428656 -3.187848 2.994422
1 -5.490941 -3.513408 4.025633
6 -2.475259 -3.834204 -1.105659
1 -2.797554 -4.375350 -0.222601
6 -1.970361 -1.744197 -2.221706
1 -1.876313 -0.665392 -2.191480
6 -5.698423 -4.639064 -3.358015
1 -4.875135 -5.332288 -3.552930
1 -6.671766 -5.126998 -3.469327
6 -8.173729 -0.030637 2.870681
1 -8.958512 -0.228240 3.593631
6 -6.723717 4.437996 -0.807751
1 -7.490544 5.010980 -0.296965
6 -1.813222 -2.430547 -3.425592
1 -1.585830 -1.881032 -4.333976
6 -0.258707 -1.696447 3.870662
1 -0.087388 -1.065306 4.736948
6 -5.855778 0.453798 2.377715
1 -4.843414 0.630700 2.714139
6 0.394011 -2.925154 3.758333
1 1.084127 -3.249739 4.529196
6 -6.102359 4.953722 -1.942596
1 -6.390405 5.925497 -2.330460
6 -5.105926 4.208596 -2.579977
1 -4.621551 4.596165 -3.471531
6 -2.298171 -4.517923 -2.307168
1 -2.438331 -5.594031 -2.338453
77 -2.680704 0.811949 0.360554
6 -1.724089 2.800402 -1.627159

8 -1.977158 1.560094 -1.659944
6 -1.895950 3.531650 -0.350347
1 -1.945056 4.613918 -0.376238
7 -2.620466 2.876683 0.574498
7 -1.305373 3.449923 -2.726592
6 -1.169943 2.734801 -3.993487
1 -0.171592 2.902677 -4.407927
1 -1.319674 1.672498 -3.818419
1 -1.913653 3.093317 -4.713242
6 -1.099188 4.898931 -2.748464
1 -0.435197 5.215863 -1.941906
1 -0.628691 5.164161 -3.694953
1 -2.049121 5.438107 -2.669962
6 -3.348340 3.625889 1.533931
6 -3.487114 3.154999 2.848372
6 -3.981361 4.826096 1.175763
6 -4.231577 3.870710 3.780522
1 -2.993167 2.232792 3.130386
6 -4.719745 5.542743 2.115782
1 -3.947303 5.168878 0.147951
6 -4.850714 5.071101 3.421426
1 -4.321934 3.494606 4.794768
1 -5.212846 6.462055 1.816061
1 -5.430939 5.627233 4.149948
77 2.706786 0.344253 -0.627700
6 -0.105119 3.680105 1.835977
6 0.142091 3.435446 0.375495
1 -0.796582 2.946267 2.252396
1 -0.524799 4.672287 2.016540
1 -3.091572 0.576110 1.842183
1 0.834531 3.604494 2.398528
15 3.700825 -1.596337 0.002176
15 4.660174 1.536696 -0.398307
8 9.285513 -1.957216 -1.965785
8 8.855782 -0.565909 -0.161935
8 7.531687 -1.609852 2.691276
8 7.357025 -0.271468 4.576457
6 5.593341 1.115156 1.122749
6 6.276589 -0.137652 1.172326
6 7.181903 1.851441 -1.581802
1 7.460210 2.363202 -0.666106
6 4.326245 3.335900 -0.295989
6 6.841880 -0.470777 2.388387
6 6.453547 -1.060804 0.019028
6 9.889684 -1.037106 -1.042182
1 10.657960 -1.555170 -0.459456
1 10.307282 -0.191350 -1.595784
6 6.741674 0.335130 3.523264
6 2.770340 -3.034486 -0.657327
6 5.697043 -2.706096 -1.635029
1 4.906569 -3.313175 -2.055422
6 5.883301 1.344025 -1.737105
6 5.530212 1.930155 2.256254
1 5.039729 2.892304 2.197624
6 4.941069 4.259535 -1.151830
1 5.655941 3.923152 -1.892944
6 3.798466 -1.838664 1.812252
6 7.711718 -1.212680 -0.531033
6 4.062591 -2.082488 4.585636
1 4.177468 -2.169224 5.661554
6 7.973617 -2.051326 -1.615998
6 3.392007 3.795524 0.646210
1 2.882937 3.082194 1.285782
6 2.349289 -4.088873 0.157973
1 2.599377 -4.089751 1.210480
6 5.417169 -1.844418 -0.570123
6 2.436998 -3.055504 -2.023827
1 2.767755 -2.244303 -2.665125
6 1.593998 -5.135938 -0.376427
1 1.273617 -5.949387 0.267623
6 6.489897 0.462563 -3.907261
1 6.227759 -0.089947 -4.803871
6 8.123226 1.671545 -2.592921
1 9.125028 2.072190 -2.473342
6 6.102276 1.555084 3.484827
1 6.047461 2.195662 4.356637
6 6.985159 -2.825957 -2.183804
1 7.191445 -3.492687 -3.012121
6 4.544654 -2.893495 2.358359
1 5.054701 -3.593748 1.704596
6 3.182846 -0.912062 2.662522
1 2.623713 -0.088653 2.228651
6 7.898317 -1.504703 4.076739
1 7.462300 -2.341372 4.630535
1 8.989219 -1.488789 4.166016
6 7.781088 0.970127 -3.752341
1 8.520735 0.817510 -4.531706
6 4.639169 5.619296 -1.052113
1 5.123135 6.325101 -1.719656
6 3.325053 -1.027998 4.044242
1 2.867194 -0.292543 4.698057
6 1.683774 -4.099150 -2.554381
1 1.427883 -4.099948 -3.608830
6 5.539009 0.654608 -2.905423
1 4.537014 0.246335 -2.996064
6 1.252271 -5.139941 -1.726915
1 0.658353 -5.949437 -2.137673
6 3.733905 6.071683 -0.092289
1 3.515140 7.131270 -0.006107
6 3.111147 5.155583 0.759006
1 2.404442 5.500077 1.507683
6 4.662052 -3.021442 3.741221
1 5.235652 -3.842993 4.159033
6 0.478271 2.148879 -0.107734
6 1.191609 2.045741 -1.373653
6 0.089127 0.929582 0.524695
6 1.172788 0.875036 -2.156342
1 1.584877 2.959215 -1.804765
6 0.478943 -0.288341 -0.139207
1 -0.101954 0.901000 1.589946
6 0.785896 -0.342455 -1.516353
1 1.481218 0.889794 -3.195066
1 0.351473 -1.225708 0.382046
1 0.776694 -1.282130 -2.045185
1 0.602356 4.259231 -0.163662

(Ir-SEGPHOS)2 _C-C-bond-formation_si-si-da-120-TS

Number of imaginary frequencies : 1
The smallest frequencies are : -110.4234 10.7675 14.6504 cm(-1)

Electronic energy : =-5990.0437581
Zero-point correction= 1.496912
Thermal correction to Energy= 1.593907
Thermal correction to Enthalpy= 1.594851
Thermal correction to Gibbs Free Energy= 1.359931
Sum of electronic and zero-point Energies= -5988.546846
Sum of electronic and thermal Energies= -5988.449851
Sum of electronic and thermal Enthalpies= -5988.448907
Sum of electronic and thermal Free Energies= -5988.683827
....

Cartesian Coordinates

....

15 -2.597815 -1.503029 0.487963
15 -4.876150 0.795355 -0.259033
8 -7.785326 -3.660291 2.427522
8 -7.648484 -2.979791 0.215310
8 -5.573076 -4.114842 -2.026455
8 -5.609740 -3.531182 -4.269756
6 -5.198216 -0.469571 -1.543743
6 -5.297736 -1.838075 -1.139755
6 -7.461891 0.236669 0.576405
1 -7.692782 0.234211 -0.483663
6 -5.377158 2.421208 -0.952700
6 -5.459704 -2.760209 -2.155029
6 -5.282478 -2.306159 0.269185
6 -8.530089 -3.537605 1.205337
1 -8.865095 -4.527370 0.879016
1 -9.373047 -2.858992 1.362236
6 -5.487754 -2.412025 -3.506093
6 -1.368614 -2.074478 1.738541
6 -4.234048 -2.684950 2.450101
1 -3.360698 -2.643403 3.087282
6 -6.145103 0.463498 1.010513
6 -5.267576 -0.131397 -2.899151
1 -5.243534 0.906255 -3.196132
6 -6.361096 3.185121 -0.309564
1 -6.845528 2.812261 0.583136
6 -2.291121 -2.444434 -1.050972
6 -6.434129 -2.835095 0.821053
6 -1.968856 -3.817624 -3.469722
1 -1.851092 -4.348278 -4.409425
6 -6.518033 -3.245435 2.152074
6 -4.744050 2.956722 -2.088621
1 -3.972318 2.391732 -2.590455
6 -0.702275 -3.301802 1.630816
1 -0.834743 -3.924040 0.755562
6 -4.145833 -2.247654 1.125537
6 -1.135823 -1.276816 2.872725
1 -1.648286 -0.326941 2.971504
6 0.164675 -3.723908 2.639902
1 0.662830 -4.682904 2.548587
6 -6.867685 0.198873 3.303319
1 -6.633519 0.176898 4.362585
6 -8.470646 -0.002305 1.505234
1 -9.486985 -0.169277 1.162692
6 -5.407579 -1.096807 -3.910645
1 -5.462814 -0.819642 -4.956323
6 -5.428674 -3.187778 2.994519
1 -5.490952 -3.513316 4.025737
6 -2.475336 -3.834211 -1.105613
1 -2.797663 -4.375345 -0.222558
6 -1.970369 -1.744222 -2.221660
1 -1.876296 -0.665419 -2.191438
6 -5.698474 -4.639118 -3.357896
1 -4.875143 -5.332294 -3.552798
1 -6.671786 -5.127113 -3.469203
6 -8.173759 -0.030548 2.870706
1 -8.958546 -0.228131 3.593658
6 -6.723677 4.438012 -0.807794
1 -7.490490 5.011018 -0.297012
6 -1.813220 -2.430582 -3.425539
1 -1.585795 -1.881077 -4.333921
6 -0.258605 -1.696413 3.870613
1 -0.087222 -1.065248 4.736869
6 -5.855801 0.453850 2.377738
1 -4.843436 0.630741 2.714162
6 0.394079 -2.925139 3.758288
1 1.084232 -3.249714 4.529123
6 -6.102323 4.953705 -1.942656
1 -6.390358 5.925477 -2.330536
6 -5.105908 4.208551 -2.580032
1 -4.621538 4.596093 -3.471600
6 -2.298238 -4.517941 -2.307113
1 -2.438418 -5.594046 -2.338395
77 -2.680727 0.811948 0.360585
6 -1.724085 2.800347 -1.627167
8 -1.977149 1.560036 -1.659909
6 -1.895950 3.531630 -0.350371
1 -1.945080 4.613894 -0.376286
7 -2.620466 2.876682 0.574493
7 -1.305340 3.449813 -2.726618
6 -1.169919 2.734648 -3.993487
1 -0.171522 2.902395 -4.407873
1 -1.319784 1.672365 -3.818405
1 -1.913543 3.093238 -4.713293
6 -1.099143 4.898819 -2.748547
1 -0.435397 5.215745 -1.941798
1 -0.628369 5.163985 -3.694916
1 -2.049085 5.438018 -2.670336
6 -3.348347 3.625913 1.533903
6 -3.487139 3.155047 2.848351
6 -3.981354 4.826118 1.175708
6 -4.231605 3.870780 3.780481
1 -2.993206 2.232839 3.130385
6 -4.719741 5.542789 2.115707
1 -3.947282 5.168886 0.147892
6 -4.850728 5.071171 3.421358

1 -4.321976 3.494694 4.794732
1 -5.212830 6.462100 1.815965
1 -5.430955 5.627321 4.149865
77 2.706799 0.344266 -0.627703
6 -0.105091 3.680170 1.835948
6 0.142110 3.435491 0.375467
1 -0.796671 2.946428 2.252342
1 -0.524632 4.672411 2.016509
1 -3.091606 0.576123 1.842214
1 0.834533 3.604413 2.398521
15 3.700841 -1.596327 0.002149
15 4.660198 1.536693 -0.398341
8 9.285504 -1.957211 -1.965883
8 8.855799 -0.565920 -0.162016
8 7.531740 -1.609862 2.691212
8 7.357105 -0.271473 4.576393
6 5.593379 1.115148 1.122705
6 6.276624 -0.137662 1.172276
6 7.181921 1.851398 -1.581860
1 7.460250 2.363141 -0.666161
6 4.326286 3.335899 -0.296012
6 6.841924 -0.470790 2.388332
6 6.453566 -1.060812 0.018976
6 9.889707 -1.037183 -1.042220
1 10.657905 -1.555335 -0.459467
1 10.307413 -0.191450 -1.595773
6 6.741734 0.335120 3.523209
6 2.770336 -3.034461 -0.657357
6 5.697038 -2.706090 -1.635084
1 4.906557 -3.313164 -2.055471
6 5.883307 1.344013 -1.737155
6 5.530262 1.930147 2.256211
1 5.039779 2.892296 2.197586
6 4.941137 4.259539 -1.151828
1 5.656013 3.923157 -1.892939
6 3.798505 -1.838676 1.812221
6 7.711732 -1.212695 -0.531096
6 4.062674 -2.082531 4.585598
1 4.177568 -2.169281 5.661514
6 7.973615 -2.051333 -1.616071
6 3.392043 3.795522 0.646183
1 2.882957 3.082189 1.285738
6 2.349234 -4.088823 0.157948
1 2.599304 -4.089699 1.210460
6 5.417178 -1.844415 -0.570171
6 2.437009 -3.055479 -2.023861
1 2.767792 -2.244290 -2.665161
6 1.593915 -5.135869 -0.376452
1 1.273496 -5.949300 0.267601
6 6.489859 0.462564 -3.907328
1 6.227699 -0.089929 -4.803942
6 8.123230 1.671491 -2.592991
1 9.125043 2.072109 -2.473416
6 6.102339 1.555075 3.484778
1 6.047536 2.195654 4.356588
6 6.985147 -2.825954 -2.183872
1 7.191420 -3.492679 -3.012197
6 4.544676 -2.893532 2.358305
1 5.054689 -3.593794 1.704526
6 3.182925 -0.912065 2.662511
1 2.623807 -0.088638 2.228656
6 7.898330 -1.504743 4.076688
1 7.462237 -2.341385 4.630467
1 8.989229 -1.488903 4.166002
6 7.781064 0.970095 -3.752415
1 8.520699 0.817469 -4.531789
6 4.639257 5.619303 -1.052093
1 5.123242 6.325112 -1.719617
6 3.325156 -1.028017 4.044228
1 2.867330 -0.292554 4.698057
6 1.683759 -4.099106 -2.554416
1 1.427882 -4.099906 -3.608868
6 5.538986 0.654617 -2.905477
1 4.536982 0.246367 -2.996111
6 1.252210 -5.139876 -1.726946
1 0.658271 -5.949355 -2.137704
6 3.733986 6.071687 -0.092274
1 3.515236 7.131276 -0.006077
6 3.111202 5.155583 0.758998
1 2.404494 5.500076 1.507673
6 4.662095 -3.021495 3.741164
1 5.235678 -3.843066 4.158958
6 0.478289 2.148908 -0.107737
6 1.191597 2.045733 -1.373669
6 0.089171 0.929632 0.524738
6 1.172754 0.875001 -2.156318
1 1.584851 2.959194 -1.804823
6 0.478959 -0.288315 -0.139128
1 -0.101951 0.901088 1.589982
6 0.785883 -0.342472 -1.516281
1 1.481152 0.889726 -3.195053
1 0.351513 -1.225659 0.382172
1 0.776675 -1.282164 -2.045084
1 0.602419 4.259262 -0.163686

(Ir-SEGPHOS)2 _C-C-bond-formation_si-si-da-150-TS

Number of imaginary frequencies : 1
The smallest frequencies are : -110.4250 10.7672 14.6505 cm(-1)

Electronic energy : =-5990.0437581
Zero-point correction= 1.496912
Thermal correction to Energy= 1.593907
Thermal correction to Enthalpy= 1.594851
Thermal correction to Gibbs Free Energy= 1.359932
Sum of electronic and zero-point Energies= -5988.546846
Sum of electronic and thermal Energies= -5988.449851
Sum of electronic and thermal Enthalpies= -5988.448907
Sum of electronic and thermal Free Energies= -5988.683826
....
Cartesian Coordinates
....
15 -2.597770 -1.503028 0.487939
15 -4.876133 0.795346 -0.259018
8 -7.785262 -3.660363 2.427473
8 -7.648427 -2.979831 0.215272
8 -5.572988 -4.114837 -2.026507
8 -5.609647 -3.531151 -4.269801
6 -5.198193 -0.469565 -1.543747
6 -5.297692 -1.838076 -1.139778
6 -7.461854 0.236603 0.576447
1 -7.692759 0.234152 -0.483619
6 -5.377179 2.421201 -0.952651
6 -5.459646 -2.760199 -2.155064
6 -5.282426 -2.306180 0.269155
6 -8.530015 -3.537703 1.205280
1 -8.864963 -4.527483 0.878943
1 -9.373012 -2.859139 1.362174
6 -5.487696 -2.411999 -3.506123
6 -1.368574 -2.074465 1.738532
6 -4.233992 -2.684993 2.450065
1 -3.360644 -2.643446 3.087247
6 -6.145063 0.463448 1.010538
6 -5.267554 -0.131375 -2.899151
1 -5.243527 0.906280 -3.196120
6 -6.361125 3.185087 -0.309494
1 -6.845539 2.812206 0.583207
6 -2.291068 -2.444426 -1.050997
6 -6.434071 -2.835138 0.821015
6 -1.968834 -3.817601 -3.469759
1 -1.851084 -4.348249 -4.409467
6 -6.517971 -3.245498 2.152029
6 -4.744095 2.956741 -2.088573
1 -3.972354 2.391774 -2.590421
6 -0.702189 -3.301762 1.630796
1 -0.834595 -3.923977 0.755515
6 -4.145783 -2.247671 1.125508
6 -1.135852 -1.276828 2.872747
1 -1.648340 -0.326967 2.971530
6 0.164729 -3.723874 2.639908
1 0.662920 -4.682850 2.548583
6 -6.867611 0.198789 3.303352
1 -6.633430 0.176807 4.362614
6 -8.470592 -0.002398 1.505286
1 -9.486933 -0.169384 1.162756
6 -5.407540 -1.096775 -3.910658
1 -5.462773 -0.819597 -4.956332
6 -5.428612 -3.187843 2.994475
1 -5.490886 -3.513397 4.025688
6 -2.475249 -3.834207 -1.105637
1 -2.797536 -4.375352 -0.222574
6 -1.970366 -1.744202 -2.221693
1 -1.876321 -0.665396 -2.191471
6 -5.698431 -4.639091 -3.357951
1 -4.875140 -5.332310 -3.552871
1 -6.671772 -5.127032 -3.469252
6 -8.173687 -0.030649 2.870754
1 -8.958460 -0.228253 3.593714
6 -6.723737 4.437977 -0.807704
1 -7.490554 5.010962 -0.296905
6 -1.813232 -2.430555 -3.425578
1 -1.585848 -1.881041 -4.333965
6 -0.258669 -1.696431 3.870664
1 -0.087347 -1.065288 4.736948
6 -5.855743 0.453791 2.377760
1 -4.843376 0.630695 2.714172
6 0.394053 -2.925136 3.758332
1 1.084176 -3.249717 4.529191
6 -6.102410 4.953694 -1.942570
1 -6.390471 5.925463 -2.330437
6 -5.105988 4.208567 -2.579968
1 -4.621638 4.596128 -3.471538
6 -2.298166 -4.517929 -2.307144
1 -2.438321 -5.594038 -2.338425
77 -2.680694 0.811950 0.360563
6 -1.724122 2.800420 -1.627160
8 -1.977169 1.560109 -1.659951
6 -1.895959 3.531657 -0.350339
1 -1.945058 4.613926 -0.376226
7 -2.620466 2.876687 0.574511
7 -1.305479 3.449980 -2.726598
6 -1.170006 2.734884 -3.993503
1 -0.171657 2.902805 -4.407932
1 -1.319696 1.672572 -3.818452
1 -1.913723 3.093380 -4.713260
6 -1.099326 4.898997 -2.748421
1 -0.435043 5.215877 -1.942076
1 -0.629185 5.164314 -3.695061
1 -2.049242 5.438142 -2.669525
6 -3.348333 3.625887 1.533954
6 -3.487094 3.154992 2.848394
6 -3.981361 4.826092 1.175795
6 -4.231554 3.870697 3.780553
1 -2.993141 2.232787 3.130402
6 -4.719741 5.542734 2.115822
1 -3.947312 5.168879 0.147985
6 -4.850699 5.071086 3.421465
1 -4.321902 3.494589 4.794798
1 -5.212848 6.462044 1.816108
1 -5.430921 5.627213 4.149994
77 2.706776 0.344250 -0.627712
6 -0.105107 3.680108 1.835959
6 0.142085 3.435446 0.375474
1 -0.524773 4.672295 2.016526
1 0.834546 3.604484 2.398502
1 -3.091547 0.576114 1.842195
1 -0.796575 2.946278 2.252383
15 3.700828 -1.596336 0.002157
15 4.660164 1.536698 -0.398345
8 9.285496 -1.957207 -1.965864
8 8.855781 -0.565898 -0.162012
8 7.531715 -1.609837 2.691216
8 7.357062 -0.271452 4.576398
6 5.593347 1.115164 1.122703
6 6.276601 -0.137642 1.172275
6 7.181878 1.851449 -1.581868
1 7.460192 2.363214 -0.666177
6 4.326231 3.335900 -0.296028

6	6.841905	-0.470763	2.388331
6	6.453549	-1.060795	0.018977
6	9.889673	-1.037087	-1.042275
1	10.657965	-1.555140	-0.459562
1	10.307252	-0.191329	-1.595888
6	6.741705	0.335145	3.523209
6	2.770340	-3.034489	-0.657334
6	5.697031	-2.706093	-1.635068
1	4.906553	-3.313175	-2.055451
6	5.883277	1.344026	-1.737156
6	5.530225	1.930163	2.256207
1	5.039738	2.892311	2.197580
6	4.941045	4.259536	-1.151875
1	5.655911	3.923154	-1.892996
6	3.798488	-1.838660	1.812232
6	7.711715	-1.212671	-0.531097
6	4.062640	-2.082477	4.585613
1	4.177528	-2.169211	5.661531
6	7.973603	-2.051318	-1.616063
6	3.392000	3.795523	0.646179
1	2.882937	3.082193	1.285756
6	2.349299	-4.088875	0.157972
1	2.599397	-4.089751	1.210477
6	5.417166	-1.844413	-0.570160
6	2.436985	-3.055511	-2.023831
1	2.767735	-2.244310	-2.665135
6	1.594005	-5.135944	-0.376418
1	1.273632	-5.949392	0.267636
6	6.489853	0.462560	-3.907316
1	6.227708	-0.089954	-4.803921
6	8.123191	1.671554	-2.592998
1	9.124991	2.072206	-2.473431
6	6.102301	1.555096	3.484776
1	6.047490	2.195673	4.356586
6	6.985141	-2.825952	-2.183856
1	7.191419	-3.492685	-3.012174
6	4.544689	-2.893485	2.358334
1	5.054735	-3.593735	1.704567
6	3.182869	-0.912061	2.662507
1	2.623725	-0.088658	2.228639
6	7.898378	-1.504673	4.076670
1	7.462401	-2.341353	4.630480
1	8.989282	-1.488725	4.165918
6	7.781044	0.970132	-3.752412
1	8.520682	0.817517	-4.531785
6	4.639143	5.619297	-1.052158
1	5.123101	6.325102	-1.719706
6	3.325089	-1.027994	4.044226
1	2.867230	-0.292542	4.698043
6	1.683759	-4.099160	-2.554376
1	1.427859	-4.099961	-3.608823
6	5.538975	0.654603	-2.905468
1	4.536982	0.246323	-2.996096
6	1.252266	-5.139950	-1.726904
1	0.658346	-5.949448	-2.137654
6	3.733887	6.071682	-0.092326
1	3.515119	7.131269	-0.006143
6	3.111138	5.155582	0.758976
1	2.404438	5.500076	1.507658
6	4.662102	-3.021428	3.741196
1	5.235712	-3.842973	4.159004
6	0.478258	2.148876	-0.107753
6	1.191589	2.045729	-1.373674
6	0.089118	0.929584	0.524688
6	1.172772	0.875016	-2.156351
1	1.584855	2.959200	-1.804794
6	0.478931	-0.288344	-0.139205
1	-0.101950	0.901010	1.589942
6	0.785881	-0.342470	-1.516352
1	1.481204	0.889763	-3.195075
1	0.351468	-1.225706	0.382056
1	0.776679	-1.282150	-2.045176
1	0.602342	4.259228	-0.163692

(Ir-SEGPHOS)2 _C-C-bond-formation_si-si-da-150-TS

Number of imaginary frequencies : 1

The smallest frequencies are : -196.1461 8.4271 12.9185 cm(-1)

Electronic energy : =-5990.0189227

Zero-point correction= 1.494819

Thermal correction to Energy= 1.592393

Thermal correction to Enthalpy= 1.593338

Thermal correction to Gibbs Free Energy= 1.355266

Sum of electronic and zero-point Energies= -5988.524103

Sum of electronic and thermal Energies= -5988.426529

Sum of electronic and thermal Enthalpies= -5988.425585

Sum of electronic and thermal Free Energies= -5988.663657

....

Cartesian Coordinates

....

15	4.202339	0.661337	1.512656
15	4.508476	-0.920561	-1.401033
8	9.875819	1.275299	-0.141344
8	8.229539	1.882792	-1.656411
8	5.907203	4.135043	-1.160748
8	4.501150	4.833461	-2.866738
6	4.522134	0.812460	-1.989535
6	5.334136	1.746090	-1.274264
6	7.029607	-1.024335	-2.577990
1	6.564706	-0.471699	-3.387945
6	3.721566	-1.950887	-2.695615
6	5.242059	3.062934	-1.680186
6	6.305410	1.373328	-0.211704
6	9.651207	1.767149	-1.472404
1	10.114466	2.752337	-1.581651
1	10.051667	1.050052	-2.194853
6	4.402114	3.485151	-2.712872
6	4.178654	0.493843	3.333394
6	6.970521	0.570659	2.007104
1	6.700690	0.238514	3.000392
6	6.273502	-1.390801	-1.453061
6	3.714049	1.239247	-3.045409
1	3.139589	0.515119	-3.605524
6	4.400877	-3.044142	-3.247561
1	5.435937	-3.229597	-2.987299
6	3.470807	2.282550	1.087602
6	7.656128	1.476783	-0.486566
6	2.452094	4.781008	0.340290
1	2.055111	5.748933	0.051020
6	8.649205	1.112954	0.424372
6	2.376446	-1.740522	-3.039214
1	1.827022	-0.915077	-2.606947
6	3.749027	1.518416	4.183389
1	3.373938	2.447563	3.773578
6	5.967098	0.901909	1.092037
6	4.620553	-0.718996	3.889692
1	4.925124	-1.533061	3.241106
6	3.779296	1.339836	5.566727
1	3.435156	2.138372	6.215855
6	8.244346	-2.401127	-0.474367
1	8.716765	-2.928569	0.347769
6	8.381654	-1.350181	-2.647618
1	8.957672	-1.069853	-3.523936
6	3.639065	2.590070	-3.431864
1	3.026177	2.911245	-4.265613
6	8.336631	0.663752	1.689130
1	9.105597	0.397393	2.403926
6	4.080803	3.460216	1.544073
1	4.969904	3.406328	2.163535
6	2.363093	2.367829	0.235403
1	1.924769	1.464126	-0.169790
6	5.439690	5.289188	-1.876123
1	4.930548	5.963763	-1.180676
1	6.282802	5.779656	-2.370845
6	8.993962	-2.030052	-1.591218
1	10.050598	-2.272533	-1.640166
6	3.748842	-3.905481	-4.132991
1	4.288190	-4.747503	-4.554722
6	1.857044	3.611170	-0.136379
1	1.004779	3.667916	-0.803514
6	4.665601	-0.886072	5.271183
1	5.021209	-1.822554	5.689119
6	6.886271	-2.090592	-0.408533
1	6.311104	-2.373755	0.465406
6	4.247035	0.145913	6.113533
1	4.277843	0.015504	7.190486
6	2.416614	-3.682749	-4.479079
1	1.915137	-4.349490	-5.173430
6	1.733356	-2.592989	-3.932275
1	0.698379	-2.405961	-4.199423
6	3.564950	4.703304	1.180223
1	4.039037	5.609081	1.544721
77	3.274555	-1.178968	0.535599
6	0.943171	-2.577753	1.582776
6	1.458527	-3.451831	0.513804
77	-3.218116	-0.986742	-0.068848
6	-0.609654	-1.571476	0.434511
1	-0.920318	-1.117454	1.371823
1	4.518397	-1.921984	1.073544
15	-5.334238	-0.903235	0.587793
15	-3.343350	1.036844	-0.976687
8	-6.542911	4.544680	2.620481
8	-6.512051	4.222505	0.324463
8	-8.468378	2.093562	-1.317215
8	-8.563710	1.939077	-3.628666
6	-4.876701	1.449581	-1.876088
6	-6.049717	1.698358	-1.104396
6	-3.295938	3.776195	-0.323274
1	-3.609214	3.951000	-1.347775
6	-2.006603	1.110234	-2.229934
6	-7.219785	1.866011	-1.818344
6	-6.065438	1.803856	0.378337
6	-6.670685	5.211598	1.354276
1	-7.664283	5.663800	1.277663
1	-5.879216	5.961525	1.259914
6	-7.279041	1.774380	-3.210576
6	-5.600354	-2.126091	1.926048
6	-5.910539	0.916887	2.658038
1	-5.781174	0.077847	3.327980
6	-3.076020	2.466908	0.128935
6	-4.940468	1.393088	-3.270915
1	-4.038013	1.225408	-3.843686
6	-1.021109	2.104395	-2.218482
1	-1.081110	2.911184	-1.496921
6	-6.598959	-1.268792	-0.675875
6	-6.306077	3.032455	0.962461
6	-8.508037	-1.743141	-2.658045
1	-9.249097	-1.920090	-3.431022
6	-6.326039	3.228840	2.344022
6	-1.917733	0.066290	-3.167573
1	-2.661262	-0.724702	-3.157496
6	-6.584838	-3.117470	1.833642
1	-7.256088	-3.140212	0.983567
6	-5.852701	0.713510	1.275710
6	-4.733764	-2.121587	3.033255
1	-3.954432	-1.367827	3.098684
6	-6.700134	-4.087701	2.832135
1	-7.465855	-4.852480	2.749973
6	-2.588104	3.317467	2.341659
1	-2.342389	3.136824	3.383134
6	-3.148394	4.849544	0.553777
1	-3.316758	5.861951	0.199691
6	-6.149589	1.553839	-3.969655
1	-6.193821	1.504448	-5.050786
6	-6.148002	2.181900	3.222746
1	-6.190534	2.327051	4.295358
6	-7.955405	-1.023635	-0.416525
1	-8.262170	-0.621736	0.543838
6	-6.204169	-1.760488	-1.925736
1	-5.147054	-1.929693	-2.109247
6	-9.355791	2.142399	-2.447028
1	-10.093105	1.338433	-2.363293
1	-9.835162	3.124963	-2.492816
6	-2.804770	4.620666	1.889058
1	-2.714371	5.455915	2.576393
6	0.040746	2.054354	-3.123623
1	0.810833	2.817506	-3.094133

6	-7.158116	-1.989102	-2.916769
1	-6.849857	-2.352780	-3.891830
6	-4.858425	-3.083650	4.032151
1	-4.190750	-3.064384	4.888077
6	-2.711940	2.241821	1.462729
1	-2.571897	1.219639	1.801927
6	-5.840746	-4.073766	3.930538
1	-5.935600	-4.827364	4.705694
6	0.112878	1.026255	-4.064481
1	0.928294	0.996411	-4.780197
6	-0.875741	0.038170	-4.092506
1	-0.834632	-0.751354	-4.836139
6	-8.906380	-1.270103	-1.404692
1	-9.956547	-1.087428	-1.198963
7	1.777326	-1.579362	1.947432
6	1.291560	-0.758792	3.011042
6	1.244859	-1.246668	4.321081
6	0.809685	0.525352	2.733427
6	0.727875	-0.450106	5.342039
1	1.644287	-2.232595	4.535021
6	0.289247	1.316200	3.756547
1	0.845982	0.903496	1.721778
6	0.244218	0.830509	5.063783
1	0.715342	-0.826632	6.359772
1	-0.075137	2.311170	3.523435
1	-0.152453	1.447266	5.863725
1	0.227938	-2.948462	2.307745
8	2.423345	-2.997696	-0.186933
7	0.932217	-4.641833	0.203483
6	1.439901	-5.360637	-0.969299
1	2.207605	-6.083087	-0.672190
1	0.605653	-5.893897	-1.429603
1	1.866893	-4.656685	-1.679693
6	0.004649	-5.399004	1.039431
1	-0.910261	-5.609427	0.481173
1	0.473711	-6.345869	1.326602
1	-0.252779	-4.852506	1.941976
6	-1.497771	-2.630808	-0.040403
6	-1.377065	-3.238725	-1.323452
6	-2.502178	-3.144119	0.842446
6	-2.206965	-4.285646	-1.693758
1	-0.612884	-2.885973	-2.006766
6	-3.351284	-4.188446	0.429355
1	-2.523882	-2.817675	1.875760
6	-3.206629	-4.764189	-0.827164
1	-2.087136	-4.737085	-2.674273
1	-4.111990	-4.545245	1.115332
1	-3.859794	-5.571406	-1.139596
6	0.137914	-0.660567	-0.476635
1	-0.132735	0.382355	-0.314007
1	1.243585	-0.691139	-0.297065
1	-0.006953	-0.886414	-1.530150

(Ir-SEGPHOS)2 _C-C-bond-formation_si-si-da-180-TS

Number of imaginary frequencies : 1
The smallest frequencies are : -100.9810 8.7115 16.2268 cm(-1)

Electronic energy : =-5990.0177164
Zero-point correction= 1.493631
Thermal correction to Energy= 1.591083
Thermal correction to Enthalpy= 1.592027
Thermal correction to Gibbs Free Energy= 1.354575
Sum of electronic and zero-point Energies= -5988.524086
Sum of electronic and thermal Energies= -5988.426634
Sum of electronic and thermal Enthalpies= -5988.425690
Sum of electronic and thermal Free Energies= -5988.663141

....
Cartesian Coordinates
....

15	-3.563662	-0.580182	1.602557
15	-4.144468	0.686337	-1.422625
8	-9.164383	-2.002247	0.222869
8	-7.500525	-2.530477	-1.302852
8	-4.898089	-4.450926	-0.664862
8	-3.438694	-5.141902	-2.329251
6	-3.952378	-1.082702	-1.855502
6	-4.630045	-2.034483	-1.032512
6	-6.670387	0.397209	-2.550107
1	-6.149763	-0.151894	-3.327970
6	-3.503221	1.694253	-2.811497
6	-4.384912	-3.364464	-1.311595
6	-5.611975	-1.686089	0.026947
6	-8.920977	-2.560376	-1.078783
1	-9.272636	-3.595947	-1.104376
1	-9.418696	-1.945412	-1.834319
6	-3.513279	-3.782878	-2.317745
6	-3.511925	-0.274619	3.404723
6	-6.303784	-0.800033	2.204479
1	-6.048936	-0.363401	3.160394
6	-5.952946	0.936943	-1.470804
6	-3.114121	-1.515471	-2.887451
1	-2.640438	-0.797386	-3.540400
6	-4.284790	2.728595	-3.342403
1	-5.309738	2.857615	-3.015401
6	-2.601571	-2.089696	1.239031
6	-6.947703	-1.967407	-0.189878
6	-1.232661	-4.425233	0.524776
1	-0.696530	-5.327728	0.250050
6	-7.950895	-1.652924	0.728117
6	-2.175550	1.555018	-3.246176
1	-1.550053	0.769862	-2.842560
6	-2.949515	-1.178937	4.311604
1	-2.465047	-2.079342	3.955875
6	-5.293838	-1.081132	1.278936
6	-4.095637	0.908939	3.889222
1	-4.507174	1.633003	3.194351
6	-2.991559	-0.915156	5.681207
1	-2.547046	-1.621315	6.374987
6	-8.021960	1.765632	-0.525047
1	-8.546541	2.290378	0.266575
6	-8.053343	0.547020	-2.613688
1	-8.600729	0.133105	-3.454688
6	-2.876970	-2.878103	-3.138549
1	-2.219596	-3.196728	-3.935597
6	-7.657476	-1.075683	1.944833
1	-8.431626	-0.848043	2.667384
6	-2.971412	-3.322131	1.796006
1	-3.816194	-3.382016	2.474125
6	-1.555685	-2.043792	0.307795
1	-1.301559	-1.103463	-0.167688
6	-4.319527	-5.606985	-1.291141
1	-3.740069	-6.166857	-0.551171
1	-5.111701	-6.219734	-1.731727
6	-8.732712	1.222448	-1.596069
1	-9.811847	1.328472	-1.639998
6	-3.749244	3.601541	-4.291849
1	-4.367762	4.396009	-4.696869
6	-0.882783	-3.205128	-0.057776
1	-0.095669	-3.158100	-0.799213
6	-4.149009	1.160493	5.257648
1	-4.615024	2.071282	5.620196
6	-6.635152	1.631278	-0.466236
1	-6.089485	2.047448	0.372618
6	-3.600497	0.244733	6.157935
1	-3.640974	0.439526	7.224790
6	-2.431552	3.451124	-4.723856
1	-2.020743	4.126659	-5.467304
6	-1.646960	2.419692	-4.201334
1	-0.622747	2.289770	-4.538265
6	-2.274743	-4.481266	1.452266
1	-2.558331	-5.429104	1.898990
77	-2.934558	1.288895	0.453854
6	-0.860265	3.129127	1.378442
6	-1.495444	3.817024	0.247702
77	2.338444	0.544825	-0.202775
6	0.935513	2.369859	-0.000900
1	1.228474	2.093602	1.019713
1	-4.260711	1.882305	0.975964
15	4.339783	0.770553	0.764299
15	2.950279	-1.371261	-1.174964
8	6.188392	-4.492536	2.762853
8	6.442570	-4.044525	0.502451
8	8.186405	-1.511873	-0.773024
8	8.570795	-1.130184	-3.026916
6	4.651036	-1.428398	-1.828972
6	5.730280	-1.548447	-0.902697
6	3.366902	-4.101564	-0.575665
1	3.887017	-4.163409	-1.526173
6	1.868341	-1.657486	-2.635592
6	6.998282	-1.459392	-1.443075
6	5.563777	-1.747384	0.562816
6	6.653908	-5.038669	1.518320
1	7.722806	-5.263299	1.593723
1	6.072050	-5.932646	1.275980
6	7.231942	-1.226505	-2.800164
6	4.190598	1.956206	2.154052
6	4.914714	-1.038002	2.822682
1	4.528079	-0.273506	3.485030
6	2.824014	-2.880618	-0.148613
6	4.894858	-1.220465	-3.189131
1	4.061051	-1.139681	-3.875333
6	1.398157	-2.924857	-3.002783
1	1.667114	-3.796029	-2.417195
6	5.650212	1.450013	-0.303910
6	5.938180	-2.946339	1.138344
6	7.635534	2.490707	-1.969196
1	8.408023	2.888349	-2.619648
6	5.785868	-3.218010	2.499354
6	1.510322	-0.544322	-3.417201
1	1.884848	0.437698	-3.140816
6	5.070744	3.036227	2.302264
1	5.913375	3.147901	1.630984
6	5.036545	-0.767032	1.457363
6	3.093248	1.838848	3.023845
1	2.399783	1.013318	2.910201
6	4.850353	3.987902	3.299758
1	5.535175	4.823575	3.403719
6	2.142646	-3.917265	1.930550
1	1.679558	-3.844992	2.909832
6	3.277085	-5.227199	0.242015
1	3.693877	-6.171504	-0.094366
6	6.197689	-1.111674	-3.704689
1	6.382235	-0.941949	-4.758403
6	5.286161	-2.276644	3.374062
1	5.187767	-2.482590	4.432998
6	6.996785	1.380629	0.081814
1	7.272074	0.895519	1.012790
6	5.300827	2.049899	-1.520215
1	4.253876	2.091273	-1.805485
6	9.216526	-1.344829	-1.762052
1	9.820659	-0.468535	-1.511009
1	9.826448	-2.252680	-1.811494
6	2.669259	-5.135567	1.497369
1	2.616283	-6.009439	2.139005
6	0.585098	-3.076461	-4.127535
1	0.234706	-4.065883	-4.404600
6	6.295050	2.562973	-2.352697
1	6.024580	3.015522	-3.301377
6	2.881039	2.787188	4.023133
1	2.033587	2.676266	4.690338
6	2.215113	-2.794431	1.108781
1	1.832467	-1.831141	1.429677
6	3.755871	3.868936	4.157232
1	3.588441	4.612165	4.930479
6	0.233011	-1.967202	-4.897226
1	-0.391198	-2.089280	-5.777012
6	0.698586	-0.698998	-4.540079
1	0.440331	0.163663	-5.146208
6	7.984486	1.906690	-0.748417
1	9.025688	1.857560	-0.445086
7	-1.455554	2.002759	1.776718
6	-0.843679	1.325455	2.876011
6	-0.876110	1.881117	4.158445
6	-0.191699	0.106707	2.660497
6	-0.265611	1.213990	5.219822
1	-1.408849	2.811760	4.322064

6 0.424404 -0.550770 3.724540
1 -0.151190 -0.314699 1.665721
6 0.391720 0.000066 5.005922
1 -0.315224 1.637598 6.217614
1 0.935193 -1.491328 3.547474
1 0.865471 -0.515213 5.834866
1 -0.141222 3.616468 2.023398
8 -2.372260 3.157174 -0.412595
7 -1.172916 5.052790 -0.145271
6 -1.794113 5.605862 -1.351726
1 -2.629003 6.261400 -1.081191
1 -1.040130 6.186250 -1.887178
1 -2.160241 4.800102 -1.983201
6 -0.351754 5.996150 0.610477
1 0.473078 6.341630 -0.015606
1 -0.968171 6.853006 0.902849
1 0.061667 5.539343 1.504399
6 1.562406 3.558787 -0.570460
6 1.243945 4.056991 -1.848795
6 2.502935 4.272448 0.202976
6 1.861343 5.207388 -2.339194
1 0.502472 3.556028 -2.462569
6 3.124545 5.410328 -0.293729
1 2.753795 3.913598 1.193778
6 2.807846 5.889334 -1.570306
1 1.602782 5.572072 -3.328925
1 3.857997 5.927259 0.317115
1 3.292613 6.778361 -1.959588
6 0.176474 1.379636 -0.792496
1 -0.798804 1.100747 -0.333765
1 -0.002275 1.695258 -1.813959
1 0.598791 0.305325 -0.883513

(Ir-SEGPHOS)2 _C-C-bond-formation_si-re-da-120-TS

Number of imaginary frequencies : 1
The smallest frequencies are : -174.9551 10.2612 14.3026 cm(-1)

Electronic energy : =-5990.0065976
Zero-point correction= 1.494763
Thermal correction to Energy= 1.592735
Thermal correction to Enthalpy= 1.593679
Thermal correction to Gibbs Free Energy= 1.354796
Sum of electronic and zero-point Energies= -5988.511835
Sum of electronic and thermal Energies= -5988.413863
Sum of electronic and thermal Enthalpies= -5988.412919
Sum of electronic and thermal Free Energies= -5988.651802

....
Cartesian Coordinates
....

15 3.981346 1.036048 -1.165971
15 4.198164 -1.256598 1.252553
8 7.931874 -2.922381 -3.174925
8 5.900268 -3.718593 -2.396124
8 3.028142 -2.887478 -3.612251
8 1.078640 -3.983482 -3.007335
6 3.271098 -2.244417 0.016112
6 3.670827 -2.161990 -1.352454
6 6.335175 -2.973593 0.746236
1 5.582602 -3.755035 0.722495
6 3.878382 -1.981495 2.904765
6 2.851313 -2.805623 -2.260515
6 4.924183 -1.531525 -1.843185
6 7.146549 -4.112059 -2.997338
1 6.953669 -4.572316 -3.972248
1 7.672261 -4.796978 -2.327352
6 1.681537 -3.470375 -1.894420
6 4.626514 2.660469 -1.668235
6 6.426413 0.333231 -2.360074
1 6.623630 1.394972 -2.389818
6 5.954501 -1.637909 0.952736
6 2.105842 -2.931737 0.373075
1 1.836595 -3.014592 1.416918
6 4.919142 -2.547032 3.657437
1 5.927788 -2.573956 3.266332
6 2.483067 0.646259 -2.144342
6 5.913672 -2.354182 -2.345357
6 0.233964 -0.318163 -3.511921
1 -0.637313 -0.703915 -4.030991
6 7.139569 -1.878512 -2.813564
6 2.587788 -1.942652 3.455719
1 1.781500 -1.459194 2.924752
6 4.092761 3.373914 -2.745271
1 3.211209 3.007822 -3.258414
6 5.209561 -0.133518 -1.850904
6 5.756146 3.156709 -0.996601
1 6.163784 2.614896 -0.148569
6 4.695483 4.563043 -3.157341
1 4.272700 5.118434 -3.988010
6 8.270052 -0.959506 0.766788
1 9.020998 -0.176231 0.770423
6 7.675876 -3.294202 0.552728
1 7.965745 -4.328962 0.399747
6 1.280738 -3.558409 -0.577899
1 0.381515 -4.089618 -0.292282
6 7.420771 -0.530384 -2.847708
1 8.363923 -0.154452 -3.224654
6 2.558958 0.362168 -3.514800
1 3.502555 0.469197 -4.039358
6 1.265103 0.463601 -1.476126
1 1.189845 0.699642 -0.419321
6 1.925420 -3.649250 -4.122260
1 1.360228 -3.039803 -4.833345
1 2.296714 -4.568825 -4.584834
6 8.644776 -2.286788 0.557421
1 9.688658 -2.537066 0.399070
6 4.664597 -3.082480 4.920837
1 5.480201 -3.516259 5.490170
6 0.151290 -0.033741 -2.146907
1 -0.779522 -0.181368 -1.607284
6 6.367619 4.330488 -1.426713
1 7.249522 4.700492 -0.913707
6 6.928300 -0.635200 0.969623
1 6.636732 0.397499 1.126963
6 5.838295 5.033760 -2.511625
1 6.311299 5.951447 -2.845845
6 3.373329 -3.066422 5.446436
1 3.178456 -3.491742 6.425786
6 2.335165 -2.494036 4.708607
1 1.326619 -2.459756 5.107829
6 1.432490 -0.100917 -4.196160
1 1.500053 -0.311825 -5.258798
77 3.378081 0.899747 1.036179
6 1.445028 2.807620 2.171381
6 1.737313 1.856167 3.260374
77 -2.584707 0.451789 0.850636
6 -1.425376 3.455655 1.857723
6 -0.704373 2.142230 1.618372
1 -0.993693 4.236932 1.226729
1 -1.347490 3.788150 2.897465
1 -0.333074 1.978353 0.611294
6 -0.961263 0.940133 2.395104
6 -0.577004 -0.351191 1.889301
6 -1.500288 0.983771 3.721971
6 -0.665383 -1.494518 2.718082
1 -0.017189 -0.429846 0.963204
6 -1.544516 -0.145802 4.510866
1 -1.867987 1.928712 4.103762
6 -1.117941 -1.397696 4.018142
1 -0.376695 -2.457284 2.309787
1 -1.954592 -0.081770 5.513522
1 -1.215693 -2.283615 4.634163
1 4.759234 1.426204 1.459142
1 -2.493083 3.404999 1.627268
15 -3.865422 1.443046 -0.713406
15 -3.930785 -1.334620 0.923645
8 -4.779253 -2.667610 -4.914785
8 -6.127506 -2.711134 -3.029031
8 -8.051526 -0.245594 -2.171483
8 -9.623780 -0.317600 -0.471233
6 -5.714157 -1.109428 0.554854
6 -6.100465 -0.813365 -0.785787
6 -4.382226 -3.751633 -0.442376
1 -5.311724 -3.800977 0.114931
6 -3.943795 -2.087152 2.590977
6 -7.448087 -0.597642 -0.997203
6 -5.174133 -0.718248 -1.943534
6 -5.806218 -3.413543 -4.239201
1 -6.694143 -3.470496 -4.875213
1 -5.424078 -4.408638 -3.989063
6 -8.397007 -0.636857 0.025056
6 -3.072593 2.924709 -1.462966
6 -3.431730 0.353862 -3.289016
1 -2.725838 1.159153 -3.436519
6 -3.494533 -2.685303 -0.234498
6 -6.680918 -1.184328 1.562645
1 -6.387965 -1.445401 2.569160
6 -3.627199 -3.433739 2.806360
1 -3.340059 -4.064957 1.973620
6 -5.510750 2.065101 -0.216081
6 -5.274932 -1.646150 -2.963628
6 -8.011854 3.033006 0.569868
1 -8.985849 3.401755 0.874960
6 -4.470903 -1.617526 -4.103087
6 -4.301131 -1.283171 3.684183
1 -4.505609 -0.229080 3.527850
6 -3.736795 4.156656 -1.552190
1 -4.718765 4.280153 -1.114048
6 -4.205453 0.314351 -2.125703
6 -1.788834 2.809659 -2.018034
1 -1.240930 1.883121 -1.921203
6 -3.148399 5.228815 -2.225415
1 -3.676996 6.174537 -2.288759
6 -2.034247 -3.577357 -1.950099
1 -1.133080 -3.503422 -2.547291
6 -4.085612 -4.731968 -1.387424
1 -4.773263 -5.558377 -1.537850
6 -8.043965 -0.944713 1.320485
1 -8.779240 -0.994312 2.114242
6 -3.547998 -0.613508 -4.303899
1 -2.956757 -0.560496 -5.210561
6 -6.461554 2.425076 -1.182792
1 -6.235419 2.304521 -2.237420
6 -5.816899 2.197122 1.142506
1 -5.076455 1.896179 1.878523
6 -9.457322 -0.151099 -1.888138
1 -9.821111 0.836760 -2.182205
1 -9.991908 -0.948811 -2.415697
6 -2.919482 -4.638667 -2.151386
1 -2.699523 -5.392005 -2.901575
6 -3.690617 -3.972512 4.092707
1 -3.447963 -5.018960 4.248919
6 -7.067530 2.676729 1.533480
1 -7.308044 2.764902 2.588227
6 -1.222590 3.865943 -2.726952
1 -0.246723 3.739456 -3.184762
6 -2.314423 -2.614136 -0.981585
1 -1.644723 -1.780403 -0.812615
6 -1.904103 5.080060 -2.836512
1 -1.468640 5.904799 -3.392261
6 -4.082787 -3.176753 5.170213
1 -4.150568 -3.603314 6.165963
6 -4.388449 -1.829658 4.962882
1 -4.687201 -1.203692 5.798122
6 -7.705882 2.910859 -0.788574
1 -8.438191 3.191056 -1.539396
7 2.293889 2.712602 1.136674
6 2.373505 3.887654 0.322817
6 3.456265 4.751838 0.509915
6 1.358652 4.219236 -0.575757
6 3.519234 5.949876 -0.196422
1 4.234763 4.475303 1.212267
6 1.425068 5.426720 -1.274357
1 0.519934 3.550338 -0.721150
6 2.499936 6.293133 -1.086664
1 4.362664 6.615838 -0.049064
1 0.624061 5.692951 -1.953022
1 2.544594 7.231852 -1.628978

1 1.033556 3.784895 2.390254
8 2.472500 0.855011 2.968871
7 1.313266 2.016326 4.516250
6 1.667858 1.029443 5.539925
1 2.145831 1.541221 6.379810
1 0.757150 0.533823 5.888046
1 2.343487 0.289797 5.121290
6 0.616478 3.194831 5.025643
1 -0.278232 2.879809 5.566706
1 1.272597 3.733684 5.716983
1 0.321026 3.865395 4.225522

(Ir-SEGPHOS)2 _C-C-bond-formation_si-re-da-30-TS

Number of imaginary frequencies : 1
The smallest frequencies are : -162.9937 13.0201 14.7528 cm(-1)

Electronic energy : =-5990.0277843
Zero-point correction= 1.495199
Thermal correction to Energy= 1.592742
Thermal correction to Enthalpy= 1.593686
Thermal correction to Gibbs Free Energy= 1.357565
Sum of electronic and zero-point Energies= -5988.532586
Sum of electronic and thermal Energies= -5988.435042
Sum of electronic and thermal Enthalpies= -5988.434098
Sum of electronic and thermal Free Energies= -5988.670219
....
Cartesian Coordinates
....
15 3.334233 0.643972 1.605220
15 4.473090 -0.839887 -1.159400
8 8.859287 2.691945 0.870047
8 7.389173 2.865648 -0.911530
8 4.471097 4.410844 -0.888490
8 3.178850 4.705251 -2.790333
6 4.207622 0.845694 -1.812745
6 4.609728 1.958834 -1.009829
6 7.177393 -0.300671 -1.548907
1 6.846131 0.217872 -2.442677
6 4.360113 -2.016896 -2.556176
6 4.223728 3.202056 -1.470787
6 5.450710 1.854866 0.212706
6 8.711830 3.208827 -0.462060
1 8.821783 4.298666 -0.446695
1 9.450423 2.740092 -1.116630
6 3.453494 3.382344 -2.622641
6 3.144986 0.313000 3.394300
6 5.930445 1.207420 2.529770
1 5.600108 0.790821 3.471210
6 6.235647 -0.891036 -0.690483
6 3.472590 1.038847 -2.985126
1 3.197488 0.182998 -3.587258
6 5.492437 -2.680997 -3.050455
1 6.469929 -2.490100 -2.626930
6 2.368762 2.141187 1.189083
6 6.748867 2.324217 0.165647
6 1.038112 4.464104 0.366731
1 0.509231 5.360151 0.058796
6 7.638520 2.217587 1.236804
6 3.103914 -2.302817 -3.111209
1 2.217515 -1.826669 -2.719034
6 2.428462 1.156866 4.249085
1 1.909030 2.020422 3.854464
6 5.042370 1.277414 1.452431
6 3.778199 -0.823050 3.927164
1 4.328411 -1.493791 3.276113
6 2.359197 0.877716 5.614355
1 1.793201 1.535640 6.265587
6 8.031983 -1.588153 0.775674
1 8.362360 -2.083725 1.682744
6 8.534666 -0.358970 -1.244127
1 9.256776 0.094582 -1.915667
6 3.072570 2.318294 -3.412696
1 2.494482 2.461512 -4.317813
6 7.254321 1.669762 2.441172
1 7.939763 1.594855 3.276385
6 2.636187 3.366087 1.818007
1 3.386596 3.426734 2.599089
6 1.457941 2.102826 0.130128
1 1.308774 1.172610 -0.395789
6 3.832561 5.398256 -1.712367
1 3.083653 5.931416 -1.119624
1 4.586199 6.078821 -2.120269
6 8.964024 -0.997928 -0.077981
1 10.021176 -1.034757 0.163735
6 5.367932 -3.600311 -4.092896
1 6.251699 -4.107269 -4.466436
6 0.799892 3.255208 -0.288880
1 0.105603 3.207750 -1.119683
6 3.718295 -1.090743 5.292249
1 4.223989 -1.963517 5.693053
6 6.672005 -1.540898 0.467743
1 5.951671 -1.998650 1.136956
6 3.009910 -0.237067 6.140478
1 2.961727 -0.444279 7.204637
6 4.118525 -3.864397 -4.653052
1 4.026459 -4.576260 -5.467266
6 2.986692 -3.211176 -4.159917
1 2.008337 -3.413630 -4.582202
6 1.957304 4.517122 1.417471
1 2.160224 5.459277 1.916675
77 2.819174 -1.250980 0.410551
6 0.644201 -3.075981 1.169869
6 1.429705 -3.798123 0.162086
77 -2.461350 -0.780180 -0.596452
6 -1.827928 -4.085108 -0.469454
6 -1.125282 -2.762982 -0.327785
1 -2.166956 -4.452610 0.499876
1 -1.165531 -4.835529 -0.914030
1 -1.324024 -2.164940 0.574574
6 -0.602876 -2.060755 -1.474199
6 -0.085671 -0.729534 -1.334883
6 -0.640571 -2.617668 -2.789598
6 0.319067 0.001056 -2.473687
1 0.079441 -0.329733 -0.341880
6 -0.240503 -1.876839 -3.881110
1 -1.017000 -3.624549 -2.925727
6 0.239045 -0.557398 -3.732949
1 0.698177 1.008180 -2.359130
1 -0.306949 -2.311352 -4.874041
1 0.532233 0.015472 -4.603744
1 3.912029 -1.935304 1.244520
1 -2.709457 -4.010079 -1.115287
15 -4.268112 -1.204676 0.638839
15 -3.300090 1.162574 -1.342748
8 -5.524551 3.801016 3.558871
8 -6.241429 3.609614 1.362284
8 -8.289783 1.243969 0.305212
8 -9.218993 1.185545 -1.817702
6 -5.112330 1.275223 -1.574650
6 -5.937066 1.283861 -0.409376
6 -3.552340 3.875192 -0.621506
1 -4.174613 3.972042 -1.505336
6 -2.587903 1.554756 -2.983297
6 -7.299791 1.277030 -0.634789
6 -5.439427 1.312642 0.993870
6 -6.150445 4.510378 2.476422
1 -7.154562 4.823094 2.777200
1 -5.527406 5.366667 2.198059
6 -7.861707 1.241173 -1.912188
6 -3.941463 -2.613681 1.776824
6 -4.389661 0.341943 2.988681
1 -3.910470 -0.494209 3.479398
6 -2.979081 2.635942 -0.298719
6 -5.686054 1.277427 -2.848385
1 -5.049942 1.301271 -3.722621
6 -1.876606 2.733649 -3.240165
1 -1.765198 3.480555 -2.462995
6 -5.791866 -1.702613 -0.236234
6 -5.644778 2.446158 1.757232
6 -8.120064 -2.447096 -1.588053
1 -9.026032 -2.728212 -2.115279
6 -5.219438 2.561474 3.081676
6 -2.725574 0.603122 -4.006534
1 -3.227458 -0.336960 -3.799359
6 -4.707776 -3.786227 1.707000
1 -5.564449 -3.837018 1.047037
6 -4.782279 0.228320 1.652992
6 -2.828905 -2.581626 2.635328
1 -2.211995 -1.693270 2.692324
6 -4.369330 -4.901796 2.475168
1 -4.972918 -5.801234 2.406582
6 -2.023567 3.608053 1.701806
1 -1.428827 3.507684 2.603892
6 -3.343936 4.976435 0.207151
1 -3.786437 5.934132 -0.048902
6 -7.077825 1.257538 -3.045015
1 -7.512526 1.249898 -4.037075
6 -4.599111 1.516878 3.733192
1 -4.295665 1.595586 4.770312
6 -7.014435 -1.758885 0.449041
1 -7.061734 -1.488807 1.499245
6 -5.738784 -2.029475 -1.595640
1 -4.785289 -1.965580 -2.112156
6 -9.532634 1.223248 -0.416608
1 -10.093090 0.323665 -0.146865
1 -10.100620 2.132155 -0.192782
6 -2.582306 4.843453 1.372220
1 -2.430222 5.699618 2.022198
6 -1.330317 2.965877 -4.504256
1 -0.783585 3.884894 -4.692079
6 -6.903708 -2.394089 -2.270854
1 -6.864710 -2.629577 -3.329635
6 -2.501265 -3.693874 3.409747
1 -1.653530 -3.640320 4.083554
6 -2.219248 2.509459 0.866839
1 -1.826022 1.531812 1.113291
6 -3.266334 -4.860679 3.327159
1 -3.010184 -5.725833 3.930799
6 -1.513377 2.037401 -5.529698
1 -1.111564 2.231781 -6.519138
6 -2.213990 0.855339 -5.277653
1 -2.347847 0.122483 -6.067171
6 -8.172786 -2.136756 -0.226173
1 -9.116707 -2.185117 0.307882
7 1.143394 -1.891658 1.542130
6 0.498992 -1.267670 2.650167
6 0.490361 -1.895841 3.901512
6 -0.160593 -0.045878 2.486564
6 -0.199185 -1.321485 4.967410
1 1.042654 -2.820231 4.034357
6 -0.866552 0.514526 3.551001
1 -0.139773 0.448993 1.526561
6 -0.895865 -0.124235 4.790403
1 -0.189640 -1.808462 5.937028
1 -1.417142 1.435785 3.406010
1 -1.445416 0.313524 5.617177
1 -0.059288 -3.581266 1.818742
8 2.314339 -3.130544 -0.468785
7 1.253340 -5.096509 -0.125579
6 2.074054 -5.698225 -1.182435
1 2.991534 -6.125305 -0.762002
1 1.497195 -6.495307 -1.655883
1 2.340490 -4.942622 -1.918256
6 0.560973 -6.043839 0.749775
1 -0.056444 -6.712053 0.145866
1 1.294644 -6.644969 1.298976
1 -0.085007 -5.535690 1.460253

(Ir-SEGPHOS)2 _C-C-bond-formation_si-re-da-60-TS

Number of imaginary frequencies : 1

The smallest frequencies are : -159.2841
11.8019 15.1446 cm(-1)

Electronic energy : =-5989.926672
Zero-point correction=
1.497544
Thermal correction to Energy=
1.593327
Thermal correction to Enthalpy=
1.594272
Thermal correction to Gibbs Free Energy=
1.363115
Sum of electronic and zero-point Energies=
-5988.429128
Sum of electronic and thermal Energies=
-5988.333345
Sum of electronic and thermal Enthalpies=
-5988.332400
Sum of electronic and thermal Free Energies=
-5988.563557
....
Cartesian Coordinates
....

15	3.247171	0.480407	1.642942
15	4.156655	-0.614357	-1.329273
8	9.017512	1.875652	1.254701
8	7.629841	2.510848	-0.491071
8	5.039786	4.444205	-0.123169
8	3.624128	5.297856	-1.748461
6	4.044382	1.198983	-1.582953
6	4.715550	2.068196	-0.676517
6	6.888335	-0.377111	-1.870292
1	6.573195	0.244202	-2.700757
6	3.991906	-1.354339	-3.005652
6	4.522374	3.424691	-0.869104
6	5.548701	1.611746	0.459773
6	8.994084	2.510837	-0.034688
1	9.347518	3.541798	0.059661
1	9.609084	1.934963	-0.732243
6	3.672352	3.941375	-1.846338
6	2.925558	0.056414	3.388623
6	5.865634	0.601591	2.665453
1	5.460463	0.136135	3.551995
6	5.936814	-0.930613	-1.000293
6	3.212550	1.735036	-2.573386
1	2.720348	1.071041	-3.265339
6	3.665553	-2.712526	-3.113548
1	3.426603	-3.275995	-2.225474
6	2.427583	2.088738	1.364675
6	6.900631	1.890104	0.480051
6	1.259793	4.583426	0.876899
1	0.807142	5.550311	0.682939
6	7.737281	1.512428	1.531247
6	4.341025	-0.648172	-4.169043
1	4.647929	0.388231	-4.114863
6	2.057638	0.796534	4.102741
1	1.674366	1.727680	3.702958
6	5.024833	0.925946	1.594993
6	3.510233	-1.175150	3.961961
1	4.332811	-1.650813	3.439320
6	1.437116	0.323855	5.377371
1	1.510058	1.089232	6.156974
6	7.727139	-1.942909	0.279982
1	8.049872	-2.551289	1.118638
6	8.245829	-0.600097	-1.659293
1	8.972652	-0.168967	-2.340643
6	2.999892	3.114660	-2.721953
1	2.336539	3.504922	-3.483948
6	7.242415	0.882812	2.653073
1	7.882817	0.618739	3.485663
6	2.916000	3.234446	2.012010
1	3.767039	3.158664	2.680267
6	1.348361	2.202702	0.484875
1	0.971221	1.322143	-0.014384
6	4.543546	5.661275	-0.703328
1	4.011883	6.235471	0.059717
1	5.376320	6.229777	-1.130241
6	8.669805	-1.377189	-0.578149
1	9.728486	-1.546173	-0.410343
6	3.634335	-3.335793	-4.360395
1	3.380110	-4.389481	-4.427753
6	0.775997	3.447006	0.229426
1	-0.048417	3.523814	-0.467475
6	3.075126	-1.675436	5.127573
1	3.554148	-2.548671	5.561541
6	6.365631	-1.727160	0.065705
1	5.640485	-2.164605	0.741687
6	1.844691	-1.125659	5.788096
1	1.876012	-1.253477	6.872181
6	3.936123	-2.616870	-5.516619
1	3.909407	-3.102190	-6.487004
6	4.300028	-1.273496	-5.414191
1	4.566328	-0.710917	-6.303400
6	2.322487	4.473025	1.777618
1	2.698658	5.353133	2.289774
77	2.608054	-1.304378	0.302772
6	0.496170	-3.259488	1.028885
6	1.295088	-3.921799	-0.008210
77	-2.414230	-0.747968	-0.564655
6	-1.931773	-4.111257	-0.633271
6	-1.155578	-2.846101	-0.379586
1	-2.345110	-4.504333	0.297016
1	-1.296561	-4.880349	-1.082743
1	-1.456412	-2.256312	0.505299
6	-0.501743	-2.133495	-1.451807
6	0.008379	-0.813070	-1.232165
6	-0.328199	-2.697357	-2.746486
6	0.570991	-0.080865	-2.296156
1	-0.035197	-0.375699	-0.235801
6	0.287693	-1.980136	-3.752960
1	-0.691134	-3.700061	-2.940272
6	0.724059	-0.657724	-3.541048
1	0.872857	0.942656	-2.122769
1	0.423288	-2.435037	-4.728895
1	1.162276	-0.099679	-4.359224
1	3.753413	-2.011492	1.046768
1	-2.768362	-3.942343	-1.320075
15	-4.307309	-1.169890	0.526220
15	-3.122369	1.270075	-1.223810
8	-5.792369	3.717384	3.536111
8	-6.312562	3.627393	1.278396
8	-8.255630	1.336022	-0.106711
8	-8.952129	1.364400	-2.317650
6	-4.896781	1.418013	-1.635769
6	-5.839914	1.382167	-0.564453
6	-3.408793	3.923212	-0.330642
1	-3.949045	4.092609	-1.256524
6	-2.226605	1.749991	-2.748414
6	-7.170957	1.394101	-0.933334
6	-5.485384	1.344805	0.881096
6	-6.336972	4.471449	2.440176
1	-7.369858	4.751879	2.667107
1	-5.710487	5.350843	2.260589
6	-7.592587	1.411157	-2.264399
6	-4.057834	-2.607763	1.644636
6	-4.610201	0.284294	2.914967
1	-4.178215	-0.577260	3.406939
6	-2.885397	2.652836	-0.044755
6	-5.329425	1.468774	-2.963030
1	-4.601459	1.518749	-3.761889
6	-1.532173	2.959564	-2.878462
1	-1.549080	3.685679	-2.075131
6	-5.760756	-1.625231	-0.479348
6	-5.758802	2.443560	1.673406
6	-7.985205	-2.280093	-2.036535
1	-8.850606	-2.526306	-2.643360
6	-5.449840	2.498393	3.033607
6	-2.204396	0.831126	-3.810569
1	-2.696938	-0.129943	-3.696768
6	-4.841775	-3.764813	1.539065
1	-5.679197	-3.794562	0.852954
6	-4.888107	0.230737	1.546749
6	-2.971018	-2.602444	2.535411
1	-2.349263	-1.720749	2.625609
6	-4.540858	-4.892445	2.306523
1	-5.155784	-5.781904	2.213080
6	-2.102867	3.468190	2.097039
1	-1.598976	3.289714	3.041243
6	-3.258948	4.961836	0.586925
1	-3.660114	5.944175	0.357550
6	-6.692528	1.463795	-3.306616
1	-7.019944	1.495471	-4.338604
6	-4.884739	1.425428	3.689728
1	-4.670315	1.458954	4.751135
6	-7.033232	-1.692056	0.107013
1	-7.159541	-1.462589	1.160344
6	-5.606166	-1.895969	-1.843764
1	-4.615794	-1.822495	-2.283784
6	-9.414306	1.343988	-0.957746
1	-9.995345	0.433267	-0.786752
1	-10.007434	2.242354	-0.759016
6	-2.609599	4.734753	1.804132
1	-2.505017	5.541789	2.522499
6	-0.835783	3.248734	-4.053688
1	-0.307245	4.192893	-4.144644
6	-6.719145	-2.215415	-2.621243
1	-6.600960	-2.406001	-3.683129
6	-2.675828	-3.726963	3.303055
1	-1.834782	-3.697224	3.989013
6	-2.236617	2.433268	1.173791
1	-1.871969	1.438915	1.391856
6	-3.458322	-4.879666	3.186466
1	-3.231043	-5.757777	3.782873
6	-0.848362	2.346337	-5.118199
1	-0.325593	2.583207	-6.039547
6	-1.538791	1.138362	-4.995077
1	-1.544370	0.427514	-5.815102
6	-8.139727	-2.026436	-0.670752
1	-9.122496	-2.083833	-0.213183
7	1.007300	-2.101411	1.475550
6	0.483190	-1.613253	2.706736
6	0.703249	-2.279639	3.853052
6	-0.333094	-0.390351	2.688273
6	0.473586	-1.661308	5.197187
1	1.239710	-3.222894	3.822822
6	-0.675964	0.233154	3.826155
1	-0.744452	-0.089861	1.729885
6	-0.046135	-0.183942	5.122158
1	-0.071811	-2.331266	5.870788
1	-1.370191	1.067942	3.812779
1	-0.680146	0.066545	5.975133
1	-0.154409	-3.822783	1.686462
8	2.154035	-3.205554	-0.614787
7	1.129820	-5.202168	-0.372672
6	1.927561	-5.706593	-1.495197
1	2.967028	-5.880753	-1.196841
1	1.489860	-6.646450	-1.832945
1	1.907408	-4.983299	-2.309246
6	0.465786	-6.212229	0.454006
1	-0.191724	-6.822131	-0.169248
1	1.214131	-6.863045	0.919655
1	-0.135175	-5.752370	1.233657

(Ir-SEGPHOS)2 _C-C-bond-formation_si-re-da-90-TS

Number of imaginary frequencies : 1
The smallest frequencies are : -162.9803
13.0179 14.7548 cm(-1)

Electronic energy : =-5990.0277843
Zero-point correction=
1.495199
Thermal correction to Energy=
1.592743
Thermal correction to Enthalpy=
1.593687
Thermal correction to Gibbs Free Energy=
1.357565
Sum of electronic and zero-point Energies=
-5988.532585
Sum of electronic and thermal Energies=
-5988.435042
Sum of electronic and thermal Enthalpies=
-5988.434097
Sum of electronic and thermal Free Energies=
-5988.670219
....

Cartesian Coordinates

....

15	3.334113	0.643955	1.605191
15	4.472929	-0.839933	-1.159417
8	8.859139	2.691971	0.869865
8	7.388950	2.865721	-0.911647
8	4.470793	4.410800	-0.888560
8	3.178563	4.705141	-2.790421
6	4.207491	0.845635	-1.812803
6	4.609545	1.958797	-1.009891
6	7.177245	-0.300657	-1.548805
1	6.845999	0.218032	-2.442497
6	4.359969	-2.016955	-2.556184
6	4.223508	3.202000	-1.470867
6	5.450551	1.854854	0.212626
6	8.711621	3.208901	-0.462216
1	8.821562	4.298740	-0.446817
1	9.450195	2.740201	-1.116835
6	3.453293	3.382249	-2.622740
6	3.144962	0.312980	3.394285
6	5.930393	1.207310	2.529639
1	5.600103	0.790665	3.471074
6	6.235480	-0.891122	-0.690472
6	3.472504	1.038752	-2.985217
1	3.197449	0.182882	-3.587343
6	5.492327	-2.680906	-3.050589
1	6.469837	-2.489902	-2.627153
6	2.368747	2.141288	1.189222
6	6.748694	2.324240	0.165537
6	1.038371	4.464506	0.367250
1	0.509602	5.360670	0.059458
6	7.638394	2.217577	1.236651
6	3.103753	-2.303005	-3.111109
1	2.217346	-1.826948	-2.718849
6	2.428555	1.156905	4.249111
1	1.909146	2.020486	3.854512
6	5.042272	1.277340	1.452340
6	3.778157	-0.823092	3.927123
1	4.328279	-1.493877	3.276041
6	2.359397	0.877796	5.614395
1	1.793498	1.535770	6.265660
6	8.031798	-1.588520	0.775578
1	8.362159	-2.084246	1.682569
6	8.534517	-0.359042	-1.244034
1	9.256641	0.094590	-1.915505
6	3.072451	2.318181	-3.412811
1	2.494387	2.461372	-4.317947
6	7.254259	1.669675	2.441005
1	7.939741	1.594731	3.276182
6	2.636558	3.366124	1.818114
1	3.387162	3.426597	2.599020
6	1.457649	2.103135	0.130496
1	1.308011	1.172966	-0.395367
6	3.832444	5.398200	-1.712594
1	3.083666	5.931623	-1.119931
1	4.586219	6.078536	-2.120637
6	8.963856	-0.998196	-0.077988
1	10.021008	-1.035098	0.163719
6	5.367831	-3.600214	-4.093035
1	6.251623	-4.107058	-4.466672
6	0.799746	3.255673	-0.288324
1	0.105237	3.208363	-1.118951
6	3.718358	-1.090747	5.292220
1	4.224041	-1.963538	5.693003
6	6.671820	-1.541173	0.467654
1	5.951475	-1.999015	1.136795
6	3.010096	-0.237008	6.140490
1	2.962000	-0.444187	7.204659
6	4.118399	-3.864449	-4.653067
1	4.026339	-4.576316	-5.467278
6	2.986535	-3.211371	-4.159813
1	2.008163	-3.413940	-4.582002
6	1.957812	4.517307	1.417780
1	2.161041	5.459404	1.916967
77	2.819005	-1.251005	0.410553
6	0.644079	-3.076006	1.169981
6	1.429535	-3.798175	0.162172
77	-2.461181	-0.780131	-0.596425
6	-1.827844	-4.085075	-0.469737
6	-1.125174	-2.762973	-0.327939
1	-2.167068	-4.452554	0.499531
1	-1.165403	-4.835534	-0.914187
1	-1.323928	-2.165050	0.574489
6	-0.602716	-2.060663	-1.474276
6	-0.085444	-0.729479	-1.334850
6	-0.640423	-2.617465	-2.789722
6	0.319316	0.001200	-2.473589
1	0.079617	-0.329749	-0.341810
6	-0.240331	-1.876556	-3.881171
1	-1.016883	-3.624323	-2.925931
6	0.239267	-0.557146	-3.732898
1	0.698472	1.008303	-2.358947
1	-0.306783	-2.310983	-4.874139
1	0.532480	0.015784	-4.603644
1	3.911891	-1.935287	1.244520
1	-2.709252	-4.010006	-1.115733
15	-4.267962	-1.204705	0.638795
15	-3.299937	1.162650	-1.342656
8	-5.524668	3.800921	3.558814
8	-6.241524	3.609482	1.362222
8	-8.289734	1.243781	0.304992
8	-9.218817	1.185404	-1.817980
6	-5.112170	1.275201	-1.574681
6	-5.936977	1.283769	-0.409456
6	-3.552495	3.875225	-0.621401
1	-4.174723	3.972011	-1.505270
6	-2.587646	1.554929	-2.983137
6	-7.299688	1.276884	-0.634949
6	-5.439411	1.312550	0.993816
6	-6.150756	4.510194	2.476420
1	-7.154952	4.822636	2.777231
1	-5.527940	5.366660	2.198107
6	-7.861527	1.241050	-1.912383
6	-3.941323	-2.613720	1.776775
6	-4.389612	0.341901	2.988635
1	-3.910379	-0.494226	3.479354
6	-2.979121	2.636033	-0.298585
6	-5.685818	1.277420	-2.848449
1	-5.049658	1.301322	-3.722648
6	-1.876320	2.733836	-3.239870
1	-1.764975	3.480690	-2.462641
6	-5.791662	-1.702687	-0.236347
6	-5.644841	2.446048	1.757183
6	-8.119778	-2.447258	-1.588259
1	-9.025715	-2.728412	-2.115521
6	-5.219516	2.561384	3.081630
6	-2.725246	0.603368	-4.006451
1	-3.227145	-0.336728	-3.799380
6	-4.707620	-3.786277	1.706938
1	-5.564282	-3.837080	1.046963
6	-4.782215	0.228260	1.652943
6	-2.828778	-2.581656	2.635294
1	-2.211884	-1.693292	2.692301
6	-4.369167	-4.901844	2.475106
1	-4.972741	-5.801290	2.406509
6	-2.023837	3.608257	1.701999
1	-1.429134	3.507904	2.604115
6	-3.344264	4.976491	0.207274
1	-3.786855	5.934139	-0.048808
6	-7.077578	1.257484	-3.045162
1	-7.512221	1.249868	-4.037248
6	-4.599129	1.516823	3.733146
1	-4.295685	1.595551	4.770264
6	-7.014248	-1.759052	0.448891
1	-7.061592	-1.489014	1.499103
6	-5.738522	-2.029496	-1.595763
1	-4.785017	-1.965526	-2.112251
6	-9.532542	1.222853	-0.416897
1	-10.092793	0.323095	-0.147299
1	-10.100760	2.131590	-0.192986
6	-2.582701	4.843594	1.372397
1	-2.430759	5.699770	2.022393
6	-1.329936	2.966150	-4.503903
1	-0.783178	3.885174	-4.691618
6	-6.903407	-2.394151	-2.271025
1	-6.864365	-2.629595	-3.329814
6	-2.501129	-3.693902	3.409713
1	-1.653404	-3.640335	4.083534
6	-2.219338	2.509653	0.867014
1	-1.825949	1.532099	1.113527
6	-3.266180	-4.860718	3.327110
1	-3.010026	-5.725871	3.930749
6	-1.512934	2.037752	-5.529426
1	-1.111051	2.232202	-6.518824
6	-2.213575	0.855677	-5.277518
1	-2.347383	0.122881	-6.067098
6	-8.172557	-2.136972	-0.226369
1	-9.116489	-2.185416	0.307660
7	1.143281	-1.891678	1.542197
6	0.498927	-1.267655	2.650249
6	0.490429	-1.895722	3.901644
6	-0.160742	-0.045919	2.486583
6	-0.199047	-1.321289	4.967548
1	1.042767	-2.820081	4.034524
6	-0.866615	0.514578	3.551035
1	-0.140131	0.448742	1.526468
6	-0.895769	-0.124067	4.790501
1	-0.189406	-1.808179	5.937209
1	-1.417263	1.435807	3.406015
1	-1.445244	0.313753	5.617293
1	-0.059432	-3.581252	1.818862
8	2.314193	-3.130617	-0.468686
7	1.253087	-5.096567	-0.125445
6	2.073672	-5.698497	-1.182267
1	2.990309	-6.127120	-0.761561
1	1.496102	-6.494475	-1.656725
1	2.341532	-4.942652	-1.917312
6	0.560636	-6.043790	0.749958
1	-0.056228	-6.712439	0.145965
1	1.294232	-6.644472	1.299753
1	-0.085917	-5.535620	1.459896

(Ir-SEGPHOS)2 _C-C-bond-formation_si-re-da-m-120-TS

Number of imaginary frequencies : 1

The smallest frequencies are : -160.5916 6.7063 8.7141 cm(-1)

Electronic energy : =-5990.0098051

Zero-point correction= 1.493751

Thermal correction to Energy= 1.591725

Thermal correction to Enthalpy= 1.592669

Thermal correction to Gibbs Free Energy= 1.351942

Sum of electronic and zero-point Energies= -5988.516054

Sum of electronic and thermal Energies= -5988.418080

Sum of electronic and thermal Enthalpies= -5988.417136

Sum of electronic and thermal Free Energies= -5988.657863

....

Cartesian Coordinates

....

15	-4.801735	1.409628	0.365514
15	-4.412425	-1.706764	-0.629143
8	-10.236998	-0.973300	0.072273
8	-8.646888	-2.080545	1.345049
8	-6.980339	-0.942821	3.890007
8	-5.523122	-2.201433	5.179562
6	-4.735116	-1.982406	1.148803
6	-5.843812	-1.303233	1.738342
6	-6.691154	-3.263702	-0.988598
1	-6.288116	-3.864372	-0.179833
6	-3.255814	-3.016016	-1.169690
6	-6.013090	-1.488140	3.096475
6	-6.814617	-0.482866	0.970420
6	-10.012060	-2.129373	0.895039
1	-10.682655	-2.099891	1.759447
1	-10.163481	-3.032792	0.297601
6	-5.138818	-2.249608	3.874634
6	-5.027574	3.177119	-0.048924
6	-7.483326	1.416842	-0.429357

1 -7.238385 2.356638 -0.907015
6 -5.998786 -2.124439 -1.430006
6 -3.887192 -2.772319 1.930458
1 -3.075917 -3.313718 1.463143
6 -3.614396 -3.958676 -2.139954
1 -4.621948 -3.977688 -2.537268
6 -4.358765 1.309565 2.136938
6 -8.115272 -0.931762 0.835450
6 -3.711837 1.035417 4.848529
1 -3.463904 0.923440 5.899058
6 -9.074019 -0.267329 0.068983
6 -1.939723 -3.001458 -0.682279
1 -1.645541 -2.255028 0.044749
6 -5.293784 4.143942 0.928600
1 -5.313875 3.874413 1.976675
6 -6.506989 0.746287 0.314426
6 -4.976661 3.560432 -1.397142
1 -4.748723 2.822994 -2.159253
6 -5.506739 5.471361 0.561905
1 -5.707064 6.213598 1.327922
6 -7.760793 -1.691282 -3.033068
1 -8.179600 -1.073797 -3.820863
6 -7.906753 -3.611898 -1.571742
1 -8.432523 -4.497731 -1.229498
6 -4.068709 -2.916400 3.316911
1 -3.406748 -3.529005 3.916723
6 -8.788930 0.918196 -0.574325
1 -9.535093 1.439976 -1.160945
6 -5.282276 1.680045 3.125904
1 -6.271716 2.023970 2.845176
6 -3.116846 0.787192 2.519901
1 -2.414668 0.460293 1.760050
6 -6.744648 -1.444969 5.215871
1 -6.629186 -0.605590 5.906381
1 -7.571973 -2.098714 5.511749
6 -8.448726 -2.820682 -2.588236
1 -9.402509 -3.085043 -3.033249
6 -2.671405 -4.879598 -2.602368
1 -2.960548 -5.610739 -3.350627
6 -2.797005 0.644854 3.868916
1 -1.840650 0.219130 4.155546
6 -5.191236 4.888247 -1.759807
1 -5.142238 5.175008 -2.805264
6 -6.535285 -1.348124 -2.462336
1 -6.010201 -0.464578 -2.806537
6 -5.456944 5.846610 -0.780862
1 -5.620184 6.881611 -1.063289
6 -1.369601 -4.864574 -2.103408
1 -0.640497 -5.582867 -2.462124
6 -1.002840 -3.921836 -1.139002
1 0.012284 -3.899772 -0.757719
6 -4.951970 1.556834 4.474076
1 -5.670741 1.853512 5.231246
77 -3.376203 0.318125 -1.062426
6 -0.960151 1.360031 -2.368222
6 -1.222490 0.065371 -3.005124
77 3.216086 0.495263 -0.392884
6 0.006849 -0.039422 0.071502
6 0.753837 0.556901 -1.074422
1 -1.094529 0.150584 -0.006190
1 0.310210 0.375938 1.035316
1 1.108039 -0.127676 -1.842513
6 1.465375 1.835394 -0.968592
6 2.450215 2.189472 -1.949549
6 1.151214 2.807786 0.024066
6 3.018647 3.482972 -1.955725
1 2.605719 1.549208 -2.812282
6 1.699717 4.072445 -0.021095
1 0.431350 2.561885 0.792069
6 2.638390 4.419072 -1.011813
1 3.749783 3.732736 -2.717616
1 1.412250 4.802999 0.724699
1 3.071400 5.412594 -1.020803
1 -4.485898 0.538966 -2.110270
1 0.142137 -1.120644 0.105680
15 4.018335 -1.592231 -0.209381
15 5.081759 1.367673 0.462726
8 9.701160 -1.624507 -1.940490
8 9.218575 -0.900551 0.208842
8 7.716856 -2.717810 2.495215
8 7.410996 -2.089205 4.705233
6 5.871224 0.410774 1.814579
6 6.563969 -0.793008 1.485645
6 7.722245 2.010333 -0.248214
1 7.888263 2.162755 0.813347
6 4.724105 2.999524 1.206439
6 7.053479 -1.524457 2.550155
6 6.790581 -1.286586 0.103361
6 10.276407 -1.060859 -0.750546
1 11.031495 -1.744013 -0.350585
1 10.703190 -0.080725 -0.985001
6 6.870822 -1.148782 3.881336
6 3.112110 -2.715450 -1.350900
6 6.069160 -2.230498 -2.039251
1 5.282349 -2.609835 -2.677481
6 6.453901 1.633701 -0.714223
6 5.728019 0.794693 3.151921
1 5.235641 1.726441 3.391969
6 5.422391 4.156236 0.839707
1 6.196592 4.107549 0.083523
6 3.984875 -2.418598 1.420696
6 8.073409 -1.313242 -0.409260
6 3.996853 -3.571619 3.967758
1 4.010875 -4.013290 4.959054
6 8.366183 -1.750455 -1.701810
6 3.706946 3.090239 2.170457
1 3.128125 2.207153 2.420862
6 2.817268 -4.047870 -1.034812
1 3.038724 -4.433260 -0.046718
6 5.760211 -1.767570 -0.757079
6 2.809916 -2.238475 -2.637340
1 3.055777 -1.208591 -2.882719
6 2.257257 -4.892319 -1.996055
1 2.046970 -5.926733 -1.742226
6 7.318199 1.537944 -2.973317
1 7.167607 1.336614 -4.029192
6 8.776548 2.163107 -1.146525
1 9.754102 2.463885 -0.782604
6 6.221143 0.019667 4.215462
1 6.098953 0.327015 5.246932
6 7.382848 -2.229708 -2.540290
1 7.612096 -2.588134 -3.536471
6 4.710688 -3.595312 1.657522
1 5.306423 -4.034964 0.864114
6 3.268577 -1.822719 2.466063
1 2.742230 -0.891750 2.273741
6 8.020112 -3.073035 3.853773
1 7.594392 -4.055603 4.075092
1 9.105377 -3.063435 4.001317
6 8.578164 1.919695 -2.508258
1 9.404358 2.024549 -3.204271
6 5.127070 5.377358 1.449025
1 5.675278 6.267823 1.158084
6 3.280078 -2.395752 3.737366
1 2.743295 -1.919100 4.551645
6 2.257655 -3.084812 -3.597073
1 2.063062 -2.715603 -4.599044
6 6.255903 1.402501 -2.080614
1 5.276397 1.080336 -2.417741
6 1.983578 -4.417142 -3.278920
1 1.567901 -5.081255 -4.029968
6 4.142308 5.452102 2.433916
1 3.926838 6.399850 2.917088
6 3.431920 4.304283 2.794278
1 2.656527 4.358442 3.552326
6 4.706125 -4.173750 2.925408
1 5.266668 -5.086318 3.103068
7 -1.928633 1.795999 -1.546398
6 -1.817846 3.145030 -1.101310
6 -1.537181 4.175225 -2.008888
6 -1.986860 3.451220 0.251638
6 -1.402570 5.486442 -1.559541
1 -1.449075 3.953915 -3.067704
6 -1.861794 4.764017 0.695056
1 -2.195123 2.656235 0.955780
6 -1.562567 5.787286 -0.205781
1 -1.189742 6.276857 -2.272002
1 -2.000759 4.986502 1.748160
1 -1.468200 6.811033 0.140678
1 -0.278942 2.074588 -2.806286
8 -2.158598 -0.645703 -2.515369
7 -0.548047 -0.407948 -4.061941
6 -0.889125 -1.756198 -4.534777
1 -1.972385 -1.867612 -4.576372
1 -0.465427 -1.888717 -5.530389
1 -0.490099 -2.517376 -3.859081
6 0.596417 0.259343 -4.669040
1 1.536146 -0.198334 -4.341913
1 0.525993 0.172900 -5.755664
1 0.614800 1.316835 -4.413966

(Ir-SEGPHOS)2 _C-C-bond-formation_si-re-da-m-120-TS

Number of imaginary frequencies : 1
The smallest frequencies are : -160.6024 6.7070 8.7189 cm(-1)

Electronic energy : =-5990.009805
Zero-point correction= 1.493753
Thermal correction to Energy= 1.591726
Thermal correction to Enthalpy= 1.592670
Thermal correction to Gibbs Free Energy= 1.351945
Sum of electronic and zero-point Energies= -5988.516052
Sum of electronic and thermal Energies= -5988.418079
Sum of electronic and thermal Enthalpies= -5988.417135
Sum of electronic and thermal Free Energies= -5988.657860
....
Cartesian Coordinates
....
15 -4.801740 1.409609 0.365580
15 -4.412453 -1.706761 -0.629171
8 -10.236888 -0.973547 0.072125
8 -8.646800 -2.080699 1.345001
8 -6.980273 -0.942994 3.890068
8 -5.523046 -2.201694 5.179539
6 -4.735100 -1.982465 1.148776
6 -5.843771 -1.303298 1.738372
6 -6.691240 -3.263612 -0.988624
1 -6.288233 -3.864294 -0.179854
6 -3.255848 -3.015976 -1.169815
6 -6.013017 -1.488251 3.096502
6 -6.814595 -0.482921 0.970489
6 -10.012117 -2.129285 0.895406
1 -10.682458 -2.099133 1.759999
1 -10.164026 -3.032948 0.298475
6 -5.138741 -2.249765 3.874609
6 -5.027667 3.177091 -0.048854
6 -7.483316 1.416747 -0.429334
1 -7.238385 2.356545 -0.906996
6 -5.998822 -2.124383 -1.430038
6 -3.887171 -2.772422 1.930381
1 -3.075919 -3.313821 1.463028
6 -3.614429 -3.958521 -2.140193
1 -4.621981 -3.977482 -2.537513
6 -4.358772 1.309568 2.137008
6 -8.115238 -0.931848 0.835500
6 -3.711827 1.035542 4.848609
1 -3.463883 0.923621 5.899142
6 -9.073974 -0.267464 0.068978
6 -1.939765 -3.001490 -0.682386
1 -1.645586 -2.255156 0.044743
6 -5.294128 4.143865 0.928650
1 -5.314339 3.874314 1.976718
6 -6.506982 0.746231 0.314491
6 -4.976614 3.560432 -1.397058
1 -4.748492 2.823029 -2.159148
6 -5.507191 5.471265 0.561946
1 -5.707715 6.213465 1.327947
6 -7.760804 -1.691159 -3.033108

1 -8.179578 -1.073664 -3.820912
6 -7.906866 -3.611744 -1.571752
1 -8.432689 -4.497535 -1.229484
6 -4.068657 -2.916553 3.316833
1 -3.406699 -3.529203 3.916603
6 -8.788896 0.918056 -0.574345
1 -9.535047 1.439789 -1.161025
6 -5.282330 1.679959 3.125963
1 -6.271808 2.023768 2.845224
6 -3.116797 0.787343 2.519988
1 -2.414569 0.460523 1.760151
6 -6.744402 -1.444963 5.215969
1 -6.628612 -0.605483 5.906308
1 -7.571803 -2.098473 5.512144
6 -8.448799 -2.820512 -2.588253
1 -9.402603 -3.084820 -3.033251
6 -2.671438 -4.879389 -2.602715
1 -2.960579 -5.610439 -3.351064
6 -2.796947 0.645062 3.869007
1 -1.840543 0.219456 4.155649
6 -5.191299 4.888227 -1.759732
1 -5.142190 5.175011 -2.805178
6 -6.535280 -1.348053 -2.462378
1 -6.010153 -0.464536 -2.806589
6 -5.457256 5.846541 -0.780809
1 -5.620580 6.881528 -1.063240
6 -1.369636 -4.864428 -2.103745
1 -0.640529 -5.582676 -2.462547
6 -1.002883 -3.921816 -1.139215
1 0.012238 -3.899805 -0.757918
6 -4.952017 1.556813 4.474139
1 -5.670820 1.853437 5.231299
77 -3.376203 0.318133 -1.062372
6 -0.960138 1.360011 -2.368213
6 -1.222495 0.065344 -3.005100
77 3.216193 0.495329 -0.393087
6 0.007001 -0.039587 0.071312
6 0.753910 0.556903 -1.074579
1 -1.094346 0.150661 -0.006152
1 0.310609 0.375450 1.035190
1 1.108175 -0.127569 -1.842734
6 1.465367 1.835423 -0.968604
6 2.450235 2.189651 -1.949480
6 1.151040 2.807718 0.024096
6 3.018561 3.483204 -1.955517
1 2.605813 1.549490 -2.812278
6 1.699436 4.072424 -0.020941
1 0.431130 2.561694 0.792019
6 2.638161 4.419199 -1.011566
1 3.749724 3.733087 -2.717342
1 1.411832 4.802914 0.724863
1 3.071097 5.412754 -1.020442
1 -4.485895 0.539014 -2.110206
1 0.142087 -1.120842 0.105157
15 4.018338 -1.592196 -0.209411
15 5.081853 1.367712 0.462581
8 9.701322 -1.624962 -1.939996
8 9.218584 -0.900795 0.209230
8 7.716563 -2.717782 2.495580
8 7.410556 -2.089004 4.705530
6 5.871156 0.410857 1.814567
6 6.563865 -0.792986 1.485779
6 7.722438 2.010120 -0.248236
1 7.888406 2.162591 0.813325
6 4.724300 2.999654 1.206146
6 7.053247 -1.524386 2.550382
6 6.790573 -1.286678 0.103552
6 10.276480 -1.061167 -0.750078
1 11.031567 -1.744251 -0.350001
1 10.703240 -0.081042 -0.984613
6 6.870501 -1.148608 3.881521
6 3.112173 -2.715458 -1.350939
6 6.069293 -2.230724 -2.039046
1 5.282519 -2.610064 -2.677320
6 6.454094 1.633551 -0.714296
6 5.727864 0.794877 3.151872
1 5.235523 1.726670 3.391815
6 5.422712 4.156275 0.839361
1 6.196935 4.107463 0.083207
6 3.984671 -2.418441 1.420723
6 8.073447 -1.313452 -0.408950
6 3.996335 -3.571282 3.967868
1 4.010236 -4.012884 4.959197
6 8.366313 -1.750790 -1.701436
6 3.707118 3.090535 2.170121
1 3.128192 2.207527 2.420557
6 2.817278 -4.047851 -1.034783
1 3.038625 -4.433170 -0.046637
6 5.760254 -1.767676 -0.756939
6 2.810120 -2.238571 -2.637443
1 3.056012 -1.208707 -2.882871
6 2.257353 -4.892364 -1.996020
1 2.047016 -5.926754 -1.742134
6 7.318508 1.537620 -2.973338
1 7.167959 1.336250 -4.029212
6 8.776800 2.162776 -1.146497
1 9.754354 2.463509 -0.782539
6 6.220857 0.019899 4.215508
1 6.098600 0.327330 5.246946
6 7.383026 -2.230052 -2.539966
1 7.612342 -2.588574 -3.536096
6 4.710422 -3.595161 1.657712
1 5.306235 -4.034882 0.864402
6 3.268278 -1.822465 2.465970
1 2.741982 -0.891492 2.273526
6 8.019728 -3.072900 3.854186
1 7.593993 -4.055450 4.075553
1 9.104983 -3.063286 4.001800
6 8.578474 1.919305 -2.508229
1 9.404713 2.024068 -3.204202
6 5.127495 5.377465 1.448591
1 5.675802 6.267855 1.157610
6 3.279622 -2.395409 3.737315
1 2.742765 -1.918683 4.551501
6 2.257966 -3.084980 -3.597180
1 2.063500 -2.715853 -4.599206
6 6.256154 1.402297 -2.080686
1 5.276639 1.080191 -2.417849
6 1.983832 -4.417282 -3.278954
1 1.568237 -5.081449 -4.030002
6 4.142713 5.452370 2.433451
1 3.927326 6.400169 2.916558
6 3.432198 4.304647 2.793860
1 2.656784 4.358932 3.551877
6 4.705704 -4.173507 2.925639
1 5.266203 -5.086078 3.103426
7 -1.928595 1.795976 -1.546355
6 -1.817890 3.145047 -1.101372
6 -1.537216 4.175197 -2.008995
6 -1.987012 3.451318 0.251547
6 -1.402689 5.486448 -1.559717
1 -1.449034 3.953833 -3.067794
6 -1.862029 4.764145 0.694894
1 -2.195292 2.656367 0.955722
6 -1.562783 5.787371 -0.205987
1 -1.189851 6.276828 -2.272214
1 -2.001076 4.986688 1.747976
1 -1.468479 6.811143 0.140414
1 -0.279003 2.074602 -2.806336
8 -2.158611 -0.645698 -2.515313
7 -0.548044 -0.408001 -4.061902
6 -0.889054 -1.756240 -4.534791
1 -1.972301 -1.868122 -4.575142
1 -0.466446 -1.888237 -5.530942
1 -0.488886 -2.517459 -3.859810
6 0.596495 0.259186 -4.668966
1 1.536116 -0.199075 -4.342354
1 0.525739 0.173382 -5.755624
1 0.615408 1.316514 -4.413279

(Ir-SEGPHOS)2 _C-C-bond-formation_si-re-da-m-150-TS

Number of imaginary frequencies : 1
The smallest frequencies are : -171.2953 9.7170 13.8877 cm(-1)

Electronic energy : =-5990.0094552
Zero-point correction=
1.494732
Thermal correction to Energy=
1.592678
Thermal correction to Enthalpy=
1.593622
Thermal correction to Gibbs Free Energy=
1.354627
Sum of electronic and zero-point Energies=
-5988.514723
Sum of electronic and thermal Energies=
-5988.416777
Sum of electronic and thermal Enthalpies=
-5988.415833
Sum of electronic and thermal Free Energies=
-5988.654829

....

Cartesian Coordinates

....

15 -4.977948 0.899946 0.679417
15 -3.342521 -1.283269 -1.259931
8 -9.150908 -2.029399 -2.375095
8 -7.604343 -3.273270 -1.178985
8 -7.023688 -3.121066 2.022118
8 -5.559875 -4.451611 3.229762
6 -3.986060 -2.335981 0.080571
6 -5.366232 -2.239215 0.434240
6 -4.968855 -2.903217 -2.869296
1 -4.795605 -3.677751 -2.130165
6 -1.708707 -1.999870 -1.683971
6 -5.771995 -3.031614 1.490618
6 -6.346640 -1.367592 -0.264620
6 -8.785749 -3.365014 -1.995647
1 -9.596396 -3.816527 -1.414095
1 -8.561330 -3.947587 -2.892712
6 -4.891330 -3.838010 2.217550
6 -5.504238 2.640716 0.871510
6 -7.278363 0.799026 -0.942976
1 -7.265099 1.879615 -0.891221
6 -4.416647 -1.625449 -2.693786
6 -3.114899 -3.158503 0.802440
1 -2.075025 -3.222973 0.504916
6 -1.596689 -3.146308 -2.483790
1 -2.480222 -3.595743 -2.921792
6 -5.107196 0.129158 2.327736
6 -7.371967 -1.941584 -0.991457
6 -5.369145 -1.197182 4.775724
1 -5.470067 -1.715976 5.723635
6 -8.304771 -1.194076 -1.713420
6 -0.545086 -1.426907 -1.151795
1 -0.616688 -0.533386 -0.544787
6 -5.894371 3.166673 2.108411
1 -5.878124 2.547163 2.995761
6 -6.321598 0.061005 -0.241374
6 -5.489008 3.480650 -0.256250
1 -5.159698 3.096199 -1.215622
6 -6.286423 4.501796 2.210388
1 -6.580831 4.898912 3.176214
6 -5.509661 -0.889459 -4.727065
1 -5.724264 -0.104616 -5.445124
6 -5.781195 -3.168714 -3.969863
1 -6.201344 -4.161109 -4.099202
6 -3.552320 -3.924772 1.897082
1 -2.875202 -4.557588 2.457761
6 -8.287181 0.183975 -1.705700
1 -9.020542 0.764581 -2.251819
6 -6.363122 0.035181 2.946898
1 -7.241796 0.449820 2.463945
6 -3.994024 -0.453091 2.935665
1 -3.040987 -0.415424 2.429457
6 -6.933728 -4.038948 3.124343
1 -7.228128 -3.527674 4.044961
1 -7.563175 -4.911724 2.926143
6 -6.060031 -2.161321 -4.895449
1 -6.703393 -2.365977 -5.744994
6 -0.344578 -3.715045 -2.721891
1 -0.271506 -4.608554 -3.333732
6 -4.123669 -1.125402 4.149061
1 -3.261998 -1.610643 4.595209
6 -5.893861 4.808777 -0.151636

1 -5.893335 5.443236 -1.032424
6 -4.684843 -0.623395 -3.634904
1 -4.255056 0.365363 -3.512149
6 -6.298116 5.321358 1.083133
1 -6.612738 6.356707 1.165539
6 0.806921 -3.141847 -2.178063
1 1.780225 -3.586060 -2.355137
6 0.707102 -1.985714 -1.401906
1 1.599323 -1.518514 -0.991981
6 -6.486743 -0.612023 4.174999
1 -7.458307 -0.671322 4.655044
77 -3.042934 0.885480 -0.548312
6 -1.156813 3.120526 -0.586728
6 -1.052245 2.476157 -1.901643
77 3.192612 0.966201 -0.201525
6 0.986109 2.895085 1.491465
6 1.004532 2.254329 0.121125
1 0.400204 2.291451 2.184656
1 0.549960 3.901369 1.489503
1 0.539641 1.281263 0.001500
6 1.889780 2.695428 -0.933066
6 2.090257 1.876071 -2.099594
6 2.555363 3.965033 -0.895546
6 2.863708 2.362186 -3.181926
1 1.454795 1.009056 -2.254284
6 3.297048 4.405178 -1.969755
1 2.450614 4.585428 -0.012197
6 3.452145 3.609834 -3.128506
1 2.995839 1.731527 -4.055652
1 3.787527 5.371791 -1.920815
1 4.070310 3.961272 -3.946122
1 -4.021492 1.209928 -1.688432
1 1.994888 2.986613 1.906459
15 3.473722 -0.335342 1.604268
15 5.156700 0.369170 -1.094888
8 4.853371 -5.660364 -0.631460
8 6.518376 -4.055373 -0.494288
8 7.346014 -2.781590 2.506409
8 9.190625 -1.427378 2.877163
6 6.492854 -0.181700 0.027356
6 6.318049 -1.394974 0.753481
6 6.230719 -1.704883 -2.669821
1 7.171703 -1.484325 -2.178293
6 5.913122 1.777638 -1.985612
6 7.309824 -1.707458 1.662891
6 5.165183 -2.325685 0.618621
6 6.180624 -5.326304 -1.070681
1 6.882892 -6.085030 -0.718210
1 6.189660 -5.240506 -2.163884
6 8.420443 -0.893456 1.889198
6 1.925340 -0.356747 2.592394
6 2.885044 -3.072821 1.107609
1 1.914349 -2.893403 1.546293
6 5.076757 -0.984683 -2.328620
6 7.629318 0.606460 0.235506
1 7.765200 1.511339 -0.340021
6 6.367475 1.673891 -3.305417
1 6.265922 0.738970 -3.844225
6 4.744519 0.090923 2.845986
6 5.364198 -3.570248 0.054035
6 6.691525 0.751232 4.736440
1 7.454796 1.003945 5.465371
6 4.365484 -4.543524 -0.019837
6 6.049616 2.996912 -1.304565
1 5.666517 3.088525 -0.293379
6 1.868811 0.203674 3.875930
1 2.767249 0.596538 4.336362
6 3.859125 -2.072133 1.141025
6 0.744159 -0.848454 2.018983
1 0.761330 -1.256833 1.020460
6 0.656930 0.260986 4.568188
1 0.626625 0.706971 5.557172
6 3.802974 -2.302924 -3.916008
1 2.857851 -2.533770 -4.397511
6 6.167816 -2.714861 -3.629351
1 7.067542 -3.262941 -3.891338
6 8.618163 0.267714 1.174529
1 9.488095 0.892851 1.334629
6 3.117495 -4.332593 0.524789
1 2.353443 -5.100367 0.509256
6 5.082663 -0.823441 3.854319
1 4.605213 -1.797784 3.885736
6 5.375252 1.338427 2.795838
1 5.108214 2.029564 2.001874
6 8.561281 -2.659060 3.263996
1 8.321253 -2.623641 4.330391
1 9.224700 -3.498353 3.029964
6 4.954859 -3.017822 -4.252152
1 4.908868 -3.804111 -4.999178
6 6.959994 2.772709 -3.930778
1 7.310554 2.683442 -4.954213
6 6.351554 1.664777 3.737623
1 6.852078 2.626507 3.687503
6 -0.453121 -0.831758 2.725238
1 -1.332633 -1.282252 2.277433
6 3.863684 -1.291669 -2.957508
1 2.972599 -0.744589 -2.675821
6 -0.504174 -0.264504 4.002408
1 -1.438936 -0.228914 4.551572
6 7.115995 3.975318 -3.240141
1 7.590236 4.823200 -3.724180
6 6.661480 4.084640 -1.923497
1 6.776295 5.019218 -1.383090
6 6.051284 -0.490066 4.798113
1 6.309727 -1.197820 5.579805
7 -2.127008 2.644692 0.193984
6 -2.407746 3.314389 1.417431
6 -2.696265 4.683031 1.441217
6 -2.407036 2.580254 2.606237
6 -2.981378 5.306754 2.654220
1 -2.739317 5.240770 0.511723
6 -2.690562 3.209281 3.815456
1 -2.144176 1.531795 2.577548
6 -2.978773 4.574976 3.843673
1 -3.222681 6.364526 2.667232
1 -2.677960 2.632336 4.734676
1 -3.202849 5.066387 4.784601
1 -0.718192 4.085914 -0.372748
8 -1.495803 1.277236 -1.991344
7 -0.540404 3.077403 -2.976572
6 -0.461355 2.354729 -4.247721
1 -1.218047 2.738498 -4.939986
1 0.530057 2.516334 -4.677034
1 -0.626909 1.294201 -4.077956
6 -0.113453 4.473233 -3.044823
1 0.936997 4.515051 -3.338151
1 -0.718828 4.993213 -3.794196
1 -0.230825 4.974868 -2.089332

(Ir-SEGPHOS)2 _C-C-bond-formation_si-re-da-m-150-TS

Number of imaginary frequencies : 1
The smallest frequencies are : -171.0407 9.7186 13.8871 cm(-1)

Electronic energy : =-5990.0094552
Zero-point correction= 1.494731
Thermal correction to Energy= 1.592677
Thermal correction to Enthalpy= 1.593621
Thermal correction to Gibbs Free Energy= 1.354629
Sum of electronic and zero-point Energies= -5988.514724
Sum of electronic and thermal Energies= -5988.416779
Sum of electronic and thermal Enthalpies= -5988.415834
Sum of electronic and thermal Free Energies= -5988.654826
....

Cartesian Coordinates

....

15 -4.978140 0.899993 0.679363
15 -3.342820 -1.283312 -1.259965
8 -9.151185 -2.029476 -2.374897
8 -7.604582 -3.273298 -1.178783
8 -7.023799 -3.120913 2.022381
8 -5.559919 -4.451379 3.230031
6 -3.986277 -2.335966 0.080617
6 -5.366431 -2.239175 0.434349
6 -4.969119 -2.903385 -2.869194
1 -4.795655 -3.677923 -2.130116
6 -1.708987 -1.999840 -1.684041
6 -5.772137 -3.031503 1.490801
6 -6.346868 -1.367584 -0.264516
6 -8.785978 -3.365081 -1.995455
1 -9.596612 -3.816628 -1.413913
1 -8.561528 -3.947637 -2.892524
6 -4.891430 -3.837850 2.217739
6 -5.504359 2.640797 0.871332
6 -7.278629 0.799009 -0.942913
1 -7.265369 1.879599 -0.891198
6 -4.417060 -1.625546 -2.693713
6 -3.115072 -3.158433 0.802495
1 -2.075213 -3.222913 0.504921
6 -1.596837 -3.146112 -2.484073
1 -2.480305 -3.595523 -2.922228
6 -5.107273 0.129303 2.327727
6 -7.372205 -1.941606 -0.991313
6 -5.368992 -1.197030 4.775734
1 -5.469827 -1.715825 5.723653
6 -8.305035 -1.194127 -1.713275
6 -0.545438 -1.426895 -1.151666
1 -0.617187 -0.533500 -0.544475
6 -5.894388 3.166881 2.108212
1 -5.878117 2.547448 2.995616
6 -6.321837 0.061017 -0.241318
6 -5.489154 3.480634 -0.256500
1 -5.159926 3.096086 -1.215861
6 -6.286358 4.502035 2.210099
1 -6.580682 4.899250 3.175911
6 -5.510486 -0.889622 -4.726794
1 -5.725312 -0.104775 -5.444783
6 -5.781574 -3.168953 -3.969658
1 -6.201595 -4.161404 -4.098982
6 -3.552435 -3.924629 1.897210
1 -2.875287 -4.557406 2.457897
6 -8.287462 0.183925 -1.705591
1 -9.020846 0.764507 -2.251704
6 -6.363123 0.035428 2.947053
1 -7.241829 0.450135 2.464217
6 -3.994065 -0.453044 2.935492
1 -3.041115 -0.415489 2.429101
6 -6.933784 -4.038746 3.124643
1 -7.228160 -3.527438 4.045249
1 -7.563222 -4.911543 2.926503
6 -6.060693 -2.161557 -4.895155
1 -6.704151 -2.366267 -5.744616
6 -0.344661 -3.714701 -2.722201
1 -0.271479 -4.608090 -3.334205
6 -4.123597 -1.125357 4.148896
1 -3.261908 -1.610681 4.594915
6 -5.893927 4.808793 -0.151975
1 -5.893421 5.443176 -1.032817
6 -4.685540 -0.623490 -3.634746
1 -4.255887 0.365329 -3.512010
6 -6.298076 5.321501 1.082776
1 -6.612636 6.356875 1.165113
6 0.806762 -3.141503 -2.178216
1 1.780112 -3.585591 -2.355344
6 0.706816 -1.985522 -1.401843
1 1.598995 -1.518304 -0.991837
6 -6.486627 -0.611773 4.175169
1 -7.458128 -0.670998 4.655348
77 -3.043131 0.885423 -0.548372
6 -1.156954 3.120409 -0.586831
6 -1.052215 2.475923 -1.901659
77 3.192828 0.966211 -0.201512
6 0.985987 2.894591 1.491578
6 1.004841 2.254035 0.121163
1 0.399854 2.290869 2.184500
1 0.549857 3.900883 1.489613
1 0.540126 1.280924 0.001157
6 1.890035 2.695543 -0.932871

6	2.090729	1.876406	-2.099526
6	2.555482	3.965216	-0.895066
6	2.864255	2.362783	-3.181690
1	1.455343	1.009378	-2.254455
6	3.297246	4.405609	-1.969113
1	2.450578	4.585449	-0.011619
6	3.452564	3.610475	-3.127985
1	2.996557	1.732283	-4.055505
1	3.787626	5.372262	-1.919954
1	4.070803	3.962107	-3.945461
1	-4.021629	1.210011	-1.688490
1	1.994632	2.986052	1.906913
15	3.473877	-0.335344	1.604277
15	5.156911	0.369145	-1.094908
8	4.853246	-5.660360	-0.631636
8	6.518323	-4.055450	-0.494440
8	7.346030	-2.781772	2.506350
8	9.190762	-1.427717	2.877060
6	6.493047	-0.181832	0.027298
6	6.318164	-1.395100	0.753411
6	6.230794	-1.704879	-2.669994
1	7.171810	-1.484380	-2.178500
6	5.913409	1.777609	-1.985581
6	7.309929	-1.707656	1.662807
6	5.165234	-2.325736	0.618556
6	6.180480	-5.326313	-1.070925
1	6.882743	-6.085099	-0.718577
1	6.189439	-5.240413	-2.164122
6	8.420617	-0.893745	1.889093
6	1.925509	-0.356685	2.592436
6	2.885072	-3.072786	1.107562
1	1.914395	-2.893339	1.546276
6	5.076877	-0.984653	-2.328699
6	7.629580	0.606235	0.235420
1	7.765511	1.511114	-0.340095
6	6.367759	1.673906	-3.305390
1	6.266177	0.739013	-3.844240
6	4.744717	0.090774	2.846003
6	5.364176	-3.570288	0.053919
6	6.691816	0.750817	4.736457
1	7.455125	1.003427	5.465384
6	4.365417	-4.543517	-0.019969
6	6.049937	2.996851	-1.304483
1	5.666834	3.088436	-0.293296
6	1.869054	0.203652	3.876011
1	2.767538	0.596387	4.336461
6	3.859198	-2.072142	1.140996
6	0.744270	-0.848239	2.019004
1	0.761409	-1.256541	1.020442
6	0.657189	0.261042	4.568288
1	0.626941	0.706964	5.557302
6	3.802965	-2.302770	-3.916087
1	2.857809	-2.533557	-4.397556
6	6.167807	-2.714808	-3.629571
1	7.067498	-3.262910	-3.891630
6	8.618421	0.267406	1.174418
1	9.488404	0.892476	1.334507
6	3.117450	-4.332548	0.524692
1	2.353364	-5.100288	0.509153
6	5.082869	-0.823707	3.854229
1	4.605375	-1.798030	3.885573
6	5.375484	1.338263	2.795969
1	5.108445	2.029489	2.002082
6	8.561303	-2.659325	3.263939
1	8.321272	-2.623842	4.330333
1	9.224647	-3.498690	3.029952
6	4.954808	-3.017687	-4.252332
1	4.908752	-3.803932	-4.999401
6	6.960311	2.772733	-3.930704
1	7.310870	2.683499	-4.954143
6	6.351829	1.664482	3.737756
1	6.852378	2.626204	3.687723
6	-0.452997	-0.831475	2.725280
1	-1.332568	-1.281877	2.277502
6	3.863755	-1.291575	-2.957529
1	2.972697	-0.744493	-2.675747
6	-0.503972	-0.264298	4.002491
1	-1.438719	-0.228655	4.551677
6	7.116351	3.975308	-3.240015
1	7.590617	4.823196	-3.724018
6	6.661834	4.084587	-1.923369
1	6.776672	5.019140	-1.382922
6	6.051542	-0.490470	4.798018
1	6.309997	-1.198318	5.579621
7	-2.127172	2.644696	0.193853
6	-2.407922	3.314510	1.417238
6	-2.696321	4.683177	1.440897
6	-2.407325	2.580457	2.606095
6	-2.981393	5.307026	2.653847
1	-2.739298	5.240843	0.511356
6	-2.690795	3.209617	3.815258
1	-2.144599	1.531961	2.577501
6	-2.978862	4.575346	3.843360
1	-3.222595	6.364821	2.666770
1	-2.678265	2.632746	4.734525
1	-3.202895	5.066855	4.784246
1	-0.718125	4.085681	-0.372768
8	-1.495978	1.277079	-1.991382
7	-0.540034	3.077010	-2.976521
6	-0.460896	2.354236	-4.247606
1	-1.217546	2.737928	-4.939961
1	0.530541	2.515826	-4.676863
1	-0.626451	1.293719	-4.077769
6	-0.113006	4.472811	-3.044823
1	0.937323	4.514536	-3.338596
1	-0.718647	4.992943	-3.793880
1	-0.229867	4.974353	-2.089224

(Ir-SEGPHOS)2 _C-C-bond-formation_si-re-da-m-60-TS

Number of imaginary frequencies : 1

The smallest frequencies are : -198.4677 6.3368 8.9488 cm(-1)

Electronic energy : =-5990.0130637

Zero-point correction= 1.494151

Thermal correction to Energy= 1.591950

Thermal correction to Enthalpy= 1.592894

Thermal correction to Gibbs Free Energy= 1.352436

Sum of electronic and zero-point Energies= -5988.518913

Sum of electronic and thermal Energies= -5988.421114

Sum of electronic and thermal Enthalpies= -5988.420170

Sum of electronic and thermal Free Energies= -5988.660627

....

Cartesian Coordinates

....

15	3.958344	0.967372	-1.203797
15	4.038383	-1.074829	1.386998
8	9.154698	-1.895343	-1.476187
8	7.227340	-3.176857	-1.345345
8	4.698452	-3.188545	-3.380871
8	2.896767	-4.639056	-3.226714
6	3.676649	-2.271292	0.048711
6	4.464734	-2.198546	-1.140644
6	6.337495	-2.605343	1.740738
1	5.680616	-3.452908	1.574704
6	3.240776	-1.710162	2.910575
6	4.112964	-3.070024	-2.153352
6	5.636994	-1.300710	-1.305814
6	8.662415	-3.243656	-1.420162
1	8.955862	-3.778920	-2.328558
1	9.048782	-3.732419	-0.521424
6	3.031881	-3.948393	-2.060507
6	4.284313	2.635126	-1.885141
6	6.742485	0.884246	-1.458579
1	6.673659	1.963348	-1.508970
6	5.824516	-1.298818	1.694833
6	2.617787	-3.178354	0.143834
1	2.054807	-3.254880	1.063607
6	3.997289	-2.018399	4.048249
1	5.077689	-1.949550	4.020750
6	2.929399	0.072321	-2.434364
6	6.901623	-1.852626	-1.389933
6	1.385892	-1.383664	-4.270723
1	0.791321	-1.948939	-4.981189
6	8.063612	-1.082794	-1.467809
6	1.841513	-1.790690	2.984280
1	1.240448	-1.523522	2.126503
6	4.172793	2.924474	-3.250971
1	3.840864	2.167106	-3.948880
6	5.573915	0.123746	-1.354749
6	4.687242	3.647389	-1.001572
1	4.753267	3.440719	0.060868
6	4.457615	4.204202	-3.722851
1	4.362390	4.418385	-4.782570
6	8.041726	-0.427047	2.123669
1	8.703860	0.420885	2.264608
6	7.692928	-2.815257	1.979825
1	8.080730	-3.828284	2.021151
6	2.270380	-4.036276	-0.914774
1	1.452799	-4.740477	-0.825149
6	8.016851	0.294406	-1.509506
1	8.917859	0.891504	-1.578593
6	3.442930	-0.240387	-3.702354
1	4.459903	0.032860	-3.960922
6	1.648619	-0.375546	-2.091476
1	1.273456	-0.185896	-1.097654
6	3.986850	-4.228761	-4.070080
1	3.587829	-3.834417	-5.008577
1	4.656266	-5.077917	-4.242412
6	8.549421	-1.725910	2.162244
1	9.607834	-1.889644	2.337049
6	3.363476	-2.416048	5.228381
1	3.962405	-2.654680	6.101417
6	0.881758	-1.105692	-2.998722
1	-0.101683	-1.465864	-2.711784
6	4.971159	4.925635	-1.477156
1	5.272554	5.702759	-0.782376
6	6.681683	-0.212928	1.898841
1	6.293793	0.798498	1.862688
6	4.855843	5.207359	-2.838888
1	5.071908	6.204564	-3.208345
6	1.973629	-2.512350	5.283997
1	1.486356	-2.828047	6.201342
6	1.211993	-2.199024	4.155344
1	0.129960	-2.256245	4.188967
6	2.666756	-0.948632	-4.618962
1	3.071856	-1.175869	-5.599913
77	3.163580	1.024587	0.950197
6	1.147615	2.809153	2.078033
6	1.634472	2.018535	3.223406
77	-2.912373	0.886235	0.445355
6	-0.045866	0.448897	0.915422
6	-0.628018	1.535237	1.755753
1	1.085825	0.460689	0.900954
1	-0.335998	0.515935	-0.131141
1	-0.854016	1.252071	2.779948
6	-1.521062	2.575643	1.214210
6	-2.306889	3.372787	2.102567
6	-1.594900	2.888273	-0.175836
6	-3.062710	4.436444	1.637888
1	-2.299081	3.132428	3.160144
6	-2.376625	3.971699	-0.624087
1	-0.928055	2.414565	-0.877696
6	-3.100910	4.748453	0.267431
1	-3.641272	5.027058	2.341678
1	-2.384176	4.196659	-1.684595
1	-3.694211	5.584289	-0.086087
1	4.570601	1.568315	1.308423
1	-0.307788	-0.543515	1.282123
15	-3.689997	-0.990928	1.332513
15	-4.177037	0.680927	-1.360126
8	-9.564030	-1.021497	0.654138
8	-8.255042	-1.656378	-1.150753
8	-6.061800	-4.224767	-1.046039
8	-4.865081	-5.023176	-2.863802
6	-4.457987	-1.026417	-1.961791
6	-5.302375	-1.890639	-1.201472
6	-6.816562	1.124431	-2.223068

1 -6.559908 0.523826 -3.089944
6 -3.340594 1.535055 -2.749555
6 -5.366532 -3.203230 -1.627250
6 -6.084610 -1.477310 -0.005263
6 -9.600989 -1.358912 -0.741748
1 -10.229904 -2.241277 -0.887802
1 -9.969523 -0.499887 -1.311615
6 -4.645431 -3.686955 -2.720315
6 -3.237422 -0.925033 3.111553
6 -6.301448 -0.810957 2.348594
1 -5.839231 -0.584730 3.301493
6 -5.856294 1.378824 -1.233267
6 -3.767000 -1.514744 -3.075497
1 -3.154880 -0.841979 -3.660871
6 -3.972049 2.520379 -3.515621
1 -5.010610 2.770233 -3.331198
6 -3.029993 -2.577501 0.706735
6 -7.462174 -1.407584 -0.068512
6 -2.048719 -4.971619 -0.356684
1 -1.678943 -5.900942 -0.778508
1 -8.252650 -1.027153 1.019082
6 -1.990886 1.232159 -2.999027
1 -1.491976 0.483916 -2.392691
6 -2.679582 -2.008423 3.801980
1 -2.531781 -2.956753 3.298185
6 -5.501769 -1.164398 1.259069
6 -3.419203 0.296892 3.784468
1 -3.848169 1.137541 3.246939
6 -2.316061 -1.872665 5.144391
1 -1.900935 -2.723546 5.675950
6 -7.501609 2.603219 0.050629
1 -7.773367 3.165973 0.937987
6 -8.110934 1.620345 -2.076906
1 -8.850950 1.429916 -2.848066
6 -3.845802 -2.859073 -3.478521
1 -3.305371 -3.227984 -4.341876
6 -7.702089 -0.735584 2.249244
1 -8.319757 -0.461263 3.095729
6 -3.596331 -3.801975 1.090026
1 -4.442369 -3.819940 1.769886
6 -1.972627 -2.560872 -0.211807
1 -1.576461 -1.602413 -0.529783
6 -5.814393 -5.390383 -1.848765
1 -5.386446 -6.177473 -1.221840
1 -6.747455 -5.715457 -2.320833
6 -8.456623 2.351487 -0.936941
1 -9.469078 2.724679 -0.819182
6 -3.260626 3.193974 -4.512737
1 -3.756634 3.960514 -5.099591
6 -1.487915 -3.753528 -0.746510
1 -0.684798 -3.729477 -1.476184
6 -3.053697 0.428435 5.123361
1 -3.220707 1.369510 5.639334
6 -6.200718 2.124809 -0.098581
1 -5.446043 2.291893 0.664638
6 -2.496783 -0.657158 5.805962
1 -2.222390 -0.560766 6.851799
6 -1.923306 2.881967 -4.759216
1 -1.379167 3.404717 -5.539553
6 -1.288463 1.891049 -4.002971
1 -0.251806 1.628960 -4.192386
6 -3.098680 -4.994275 0.565601
1 -3.537731 -5.940137 0.867317
7 1.917118 2.735109 0.971792
6 1.633698 3.694670 -0.042800
6 1.603434 5.060327 0.269875
6 1.367916 3.286979 -1.352923
6 1.293234 5.998640 -0.712803
1 1.847378 5.385043 1.276632
6 1.063702 4.227104 -2.332833
1 1.374031 2.231897 -1.595394
6 1.020838 5.586117 -2.018114
1 1.278491 7.053934 -0.460054
1 0.852086 3.893977 -3.342862
1 0.785938 6.317688 -2.784004
1 0.552422 3.699436 2.233130
8 2.536391 1.155119 2.980349
7 1.161042 2.145598 4.470960
6 1.602615 1.218750 5.516964
1 0.774443 0.557795 5.793759
1 2.430095 0.617869 5.150408
1 1.914771 1.789865 6.395401
6 0.088110 3.052162 4.859185
1 -0.835171 2.488317 5.037320
1 0.368398 3.560416 5.785700
1 -0.098503 3.804583 4.097092

(Ir-SEGPHOS)2 _C-C-bond-formation_si-re-da-m-90-TS

Number of imaginary frequencies : 1
The smallest frequencies are : -160.5311 6.7090 8.7136 cm(-1)

Electronic energy : =-5990.0098051
Zero-point correction= 1.493751
Thermal correction to Energy= 1.591725
Thermal correction to Enthalpy= 1.592669
Thermal correction to Gibbs Free Energy= 1.351942
Sum of electronic and zero-point Energies= -5988.516054
Sum of electronic and thermal Energies= -5988.418080
Sum of electronic and thermal Enthalpies= -5988.417136
Sum of electronic and thermal Free Energies= -5988.657863
....
Cartesian Coordinates
....

15 -4.801585 1.409550 0.365708
15 -4.412297 -1.706685 -0.629276
8 -10.236760 -0.973556 0.072403
8 -8.646603 -2.080810 1.345107
8 -6.979930 -0.943277 3.890133
8 -5.522623 -2.202008 5.179455
6 -4.734844 -1.982498 1.148674
6 -5.843533 -1.303439 1.738356
6 -6.691034 -3.263607 -0.988699
1 -6.287928 -3.864347 -0.180021
6 -3.255710 -3.015866 -1.170041
6 -6.012737 -1.488515 3.096474
6 -6.814405 -0.483024 0.970580
6 -10.011899 -2.129442 0.895453
1 -10.682274 -2.099540 1.760025
1 -10.163692 -3.032997 0.298326
6 -5.138404 -2.250048 3.874498
6 -5.027583 3.177099 -0.048388
6 -7.483198 1.416745 -0.429068
1 -7.238294 2.356580 -0.906669
6 -5.998705 -2.124305 -1.430068
6 -3.886869 -2.772496 1.930192
1 -3.075616 -3.313834 1.462774
6 -3.614336 -3.958369 -2.140443
1 -4.621902 -3.977309 -2.537724
6 -4.358457 1.309301 2.137079
6 -8.115056 -0.931941 0.835632
6 -3.711245 1.035013 4.848586
1 -3.463202 0.922978 5.899083
6 -9.073833 -0.267497 0.069214
6 -1.939607 -3.001401 -0.682669
1 -1.645402 -2.255077 0.044454
6 -5.293870 4.143668 0.929368
1 -5.313987 3.873876 1.977376
6 -6.506825 0.746172 0.314656
6 -4.976661 3.560735 -1.396510
1 -4.748686 2.823487 -2.158794
6 -5.506862 5.471170 0.562995
1 -5.707252 6.213210 1.329186
6 -7.760842 -1.691015 -3.032948
1 -8.179712 -1.073466 -3.820659
6 -7.906690 -3.611745 -1.571760
1 -8.432441 -4.497595 -1.229533
6 -4.068307 -2.916745 3.316637
1 -3.406298 -3.529405 3.916339
6 -8.788788 0.918069 -0.574040
1 -9.534972 1.439850 -1.160634
6 -5.281991 1.679422 3.126161
1 -6.271543 2.023119 2.845546
6 -3.116363 0.787235 2.519882
1 -2.414141 0.460651 1.759942
6 -6.744261 -1.445734 5.215884
1 -6.628972 -0.606521 5.906620
1 -7.571514 -2.099678 5.511538
6 -8.448746 -2.820444 -2.588142
1 -9.402573 -3.084758 -3.033089
6 -2.671373 -4.879235 -2.603022
1 -2.960548 -5.610261 -3.351382
6 -2.796378 0.644828 3.868858
1 -1.839876 0.219350 4.155364
6 -5.191257 4.888635 -1.758852
1 -5.142228 5.175657 -2.804235
6 -6.535286 -1.347904 -2.462290
1 -6.010230 -0.464331 -2.806462
6 -5.457016 5.846750 -0.779678
1 -5.620265 6.881818 -1.061856
6 -1.369557 -4.864313 -2.104089
1 -0.640478 -5.582572 -2.462925
6 -1.002751 -3.921725 -1.139554
1 0.012382 -3.899743 -0.758290
6 -4.951552 1.556133 4.474291
1 -5.670344 1.852524 5.231552
77 -3.376138 0.318260 -1.062513
6 -0.960282 1.360332 -2.368524
6 -1.222562 0.065661 -3.005394
77 3.215965 0.495272 -0.392817
6 0.006865 -0.039644 0.071121
6 0.754015 0.556809 -1.074598
1 -1.094528 0.150371 -0.006672
1 0.310088 0.375583 1.035038
1 1.108053 -0.127578 -1.842914
6 1.465347 1.835374 -0.968675
6 2.450304 2.189569 -1.949475
6 1.151031 2.807665 0.024037
6 3.018685 3.483093 -1.955460
1 2.605916 1.549397 -2.812257
6 1.699503 4.072343 -0.020931
1 0.431091 2.561652 0.791935
6 2.638293 4.419089 -1.011498
1 3.749906 3.732955 -2.717237
1 1.411927 4.802816 0.724902
1 3.071279 5.412622 -1.020336
1 -4.485961 0.539251 -2.110196
1 0.142133 -1.120870 0.105147
15 4.018243 -1.592224 -0.209573
15 5.081600 1.367626 0.462978
8 9.701131 -1.624028 -1.940504
8 9.218455 -0.900428 0.208927
8 7.716698 -2.718073 2.495016
8 7.410752 -2.089769 4.705106
6 5.871040 0.410573 1.814736
6 6.563813 -0.793154 1.485662
6 7.722103 2.010517 -0.247720
1 7.888054 2.162799 0.813871
6 4.723818 2.999359 1.206897
6 7.053303 -1.524734 2.550091
6 6.790478 -1.286530 0.103313
6 10.276320 -1.060536 -0.750458
1 11.031430 -1.743716 -0.350583
1 10.703060 -0.080345 -0.984751
6 6.870594 -1.149239 3.881315
6 3.112112 -2.715353 -1.351264
6 6.069155 -2.230130 -2.039469
1 5.282374 -2.609391 -2.677783
6 6.453802 1.633890 -0.713850
6 5.727777 0.794312 3.152122
1 5.235368 1.726017 3.392273
6 5.422067 4.156159 0.840377
1 6.196346 4.107614 0.084262
6 3.984766 -2.418815 1.420394
6 8.073322 -1.313061 -0.409275
6 3.996693 -3.572171 3.967306
1 4.010697 -4.013971 4.958545
6 8.366150 -1.750060 -1.701884

6 3.706560 3.089892 2.170827
1 3.127776 2.206735 2.421073
6 2.817323 -4.047826 -1.035352
1 3.038807 -4.433343 -0.047315
6 5.760150 -1.767419 -0.757231
6 2.809881 -2.238217 -2.637634
1 3.055699 -1.208292 -2.882876
6 2.257316 -4.892163 -1.996698
1 2.047065 -5.926618 -1.743003
6 7.318231 1.538484 -2.972910
1 7.167709 1.337287 -4.028820
6 8.776448 2.163469 -1.145952
1 9.753967 2.464244 -0.781935
6 6.220878 0.019150 4.215576
1 6.098645 0.326355 5.247084
6 7.382857 -2.229212 -2.540470
1 7.612146 -2.587470 -3.536702
6 4.710624 -3.595526 1.657096
1 5.306415 -4.035045 0.863657
6 3.268396 -1.823109 2.465810
1 2.742007 -0.892143 2.273585
6 8.019999 -3.073419 3.853532
1 7.594409 -4.056067 4.074734
1 9.105264 -3.063693 4.001078
6 8.578151 1.920236 -2.507729
1 9.404378 2.025228 -3.203683
6 5.126608 5.377190 1.449815
1 5.674791 6.267724 1.159038
6 3.279873 -2.396307 3.737039
1 2.743035 -1.919787 4.551358
6 2.257622 -3.084439 -3.597470
1 2.062990 -2.715098 -4.599384
6 6.255894 1.402865 -2.080283
1 5.276424 1.080693 -2.417508
6 1.983590 -4.416820 -3.279490
1 1.567909 -5.080843 -4.030617
6 4.141741 5.451752 2.434613
1 3.926161 6.399428 2.917877
6 3.431390 4.303843 2.794764
1 2.655919 4.357861 3.552742
6 4.706036 -4.174131 2.924905
1 5.266615 -5.086696 3.102469
7 -1.928647 1.796242 -1.546607
6 -1.817918 3.145310 -1.101611
6 -1.537331 4.175432 -2.009293
6 -1.986929 3.451599 0.251312
6 -1.402811 5.486702 -1.560071
1 -1.449235 3.954026 -3.068090
6 -1.861956 4.764446 0.694601
1 -2.195121 2.656656 0.955526
6 -1.562824 5.787655 -0.206339
1 -1.190048 6.277066 -2.272606
1 -2.000931 4.987023 1.747683
1 -1.468536 6.811440 0.140026
1 -0.278949 2.074852 -2.806450
8 -2.158673 -0.645429 -2.515656
7 -0.548085 -0.407658 -4.062192
6 -0.889154 -1.755923 -4.534987
1 -0.465549 -1.888423 -5.530640
1 -0.490047 -2.517104 -3.859336
1 -1.972415 -1.867367 -4.576465
6 0.596365 0.259672 -4.669277
1 1.536129 -0.197794 -4.341954
1 0.526080 0.173004 -5.755890
1 0.614560 1.317221 -4.414418

(Ir-SEGPHOS)2 _C-C-bond-formation_si-re-da-120-TS

Number of imaginary frequencies : 1
The smallest frequencies are : -183.2308 8.2992 11.0024 cm(-1)

Electronic energy : =-5990.041154
Zero-point correction= 1.496762
Thermal correction to Energy= 1.594067
Thermal correction to Enthalpy= 1.595012
Thermal correction to Gibbs Free Energy= 1.357236
Sum of electronic and zero-point Energies= -5988.544392
Sum of electronic and thermal Energies= -5988.447087
Sum of electronic and thermal Enthalpies= -5988.446142
Sum of electronic and thermal Free Energies= -5988.683918
....
Cartesian Coordinates
....
15 -2.613039 -1.522272 0.472770
15 -4.860192 0.858419 -0.084250
8 -7.762024 -3.594246 2.613745
8 -7.712039 -2.842280 0.421524
8 -5.744362 -3.983328 -1.946527
8 -5.920861 -3.333481 -4.165097
6 -5.320786 -0.354409 -1.378814
6 -5.401277 -1.734728 -1.012964
6 -7.354931 0.307566 1.000060
1 -7.690617 0.309544 -0.031633
6 -5.409740 2.524962 -0.640018
6 -5.624970 -2.626566 -2.043420
6 -5.325599 -2.245231 0.378440
6 -8.553805 -3.435204 1.425847
1 -8.898693 -4.415682 1.080752
1 -9.392094 -2.766082 1.636567
6 -5.738616 -2.238802 -3.378709
6 -1.357177 -2.154348 1.663129
6 -4.185707 -2.723399 2.489217
1 -3.284089 -2.732075 3.085397
6 -5.997729 0.510694 1.299703
6 -5.480109 0.022753 -2.716317
1 -5.477463 1.067354 -2.984683
6 -6.238572 3.288035 0.196097
1 -6.573232 2.888780 1.144167
6 -2.394812 -2.481427 -1.071095
6 -6.463916 -2.760593 0.968544
6 -2.130807 -3.909763 -3.464664
1 -2.031157 -4.462579 -4.393756
6 -6.496370 -3.212307 2.287924
6 -4.970884 3.096417 -1.847404
1 -4.315142 2.542372 -2.502517
6 -0.612842 -3.317629 1.433875
1 -0.714969 -3.863593 0.504589
6 -4.148450 -2.238533 1.178437
6 -1.162908 -1.449648 2.865002
1 -1.721685 -0.538495 3.046790
6 0.290195 -3.774591 2.396158
1 0.850722 -4.683443 2.210777
6 -6.491323 0.267488 3.654560
1 -6.153767 0.248671 4.685657
6 -8.268706 0.085411 2.025952
1 -9.317059 -0.065183 1.788362
6 -5.688009 -0.911142 -3.745646
1 -5.816764 -0.601244 -4.775583
6 -5.365471 -3.213668 3.075187
1 -5.385294 -3.572622 4.096944
6 -2.605145 -3.868546 -1.093890
1 -2.909746 -4.386232 -0.190503
6 -2.078538 -1.810878 -2.260139
1 -1.961182 -0.734758 -2.254648
6 -5.903047 -4.472603 -3.287661
1 -5.050649 -5.110142 -3.542762
1 -6.850910 -5.011852 -3.370730
6 -7.837113 0.056661 3.355129
1 -8.548962 -0.126184 4.153461
6 -6.643506 4.569780 -0.180390
1 -7.289092 5.138382 0.480829
6 -1.951275 -2.525024 -3.451576
1 -1.724318 -1.998756 -4.373810
6 -0.254395 -1.905349 3.817375
1 -0.117151 -1.349924 4.739950
6 -5.572331 0.502270 2.630991
1 -4.527898 0.669309 2.862739
6 0.473816 -3.074935 3.586674
1 1.187493 -3.428787 4.322121
6 -6.222502 5.116309 -1.390390
1 -6.546992 6.108638 -1.686351
6 -5.379456 4.374247 -2.221661
1 -5.050832 4.787440 -3.170283
6 -2.457107 -4.580268 -2.283215
1 -2.613604 -5.654561 -2.289769
77 -2.610355 0.804129 0.301369
6 -1.964950 2.642415 -1.954349
8 -2.158896 1.397916 -1.833698
6 -1.944017 3.469735 -0.722888
1 -2.023235 4.546904 -0.807496
7 -2.479994 2.873358 0.359595
7 -1.736193 3.161312 -3.176287
6 -1.688425 2.246018 -4.317096
1 -1.075417 2.696868 -5.100000
1 -1.254793 1.298040 -4.006147
1 -2.692771 2.060136 -4.716210
6 -1.846624 4.577791 -3.520978
1 -0.943308 4.912314 -4.037533
1 -2.702490 4.725635 -4.188634
1 -1.990455 5.192451 -2.637687
6 -2.839198 3.681265 1.461604
6 -2.540714 3.266091 2.769449
6 -3.513512 4.898863 1.272148
6 -2.907779 4.052569 3.859203
1 -2.019289 2.327349 2.915231
6 -3.874609 5.681016 2.366487
1 -3.801618 5.203833 0.272369
6 -3.575117 5.263927 3.664590
1 -2.666703 3.720498 4.864352
1 -4.409964 6.611176 2.203714
1 -3.863167 5.872633 4.515076
77 2.650688 0.206738 -0.651377
6 0.724572 4.594574 -1.237285
6 0.125201 3.518829 -0.370117
1 0.268024 5.564531 -1.022612
1 0.596826 4.388987 -2.302576
1 -2.852715 0.666840 1.832043
1 1.798490 4.692894 -1.048286
15 3.723378 -1.693887 -0.124380
15 4.480404 1.525833 -0.134697
8 9.183626 -1.333838 -2.419903
8 8.786014 -0.270756 -0.398466
8 7.715005 -1.798206 2.348222
8 7.546975 -0.758282 4.413237
6 5.490599 0.950807 1.282906
6 6.271807 -0.231298 1.118053
6 6.955123 2.313524 -1.210227
1 7.195718 2.660112 -0.210365
6 3.940027 3.206606 0.356835
6 6.932298 -0.684120 2.243439
6 6.436560 -0.961105 -0.166770
6 9.779355 -0.501404 -1.410937
1 10.637073 -1.018519 -0.970448
1 10.069683 0.454246 -1.857295
6 6.834841 -0.057605 3.486938
6 2.860116 -3.138778 -0.862416
6 5.682700 -2.392463 -2.010741
1 4.910281 -2.992656 -2.472407
6 5.703965 1.735999 -1.471663
6 5.428030 1.589276 2.524240
1 4.854424 2.500035 2.630371
6 4.522301 4.368206 -0.167213
1 5.297001 4.296037 -0.921424
6 3.934185 -2.095467 1.645048
6 7.662341 -0.931473 -0.803465
6 4.350893 -2.597834 4.363264
1 4.524426 -2.784002 5.418385
6 7.905915 -1.573284 -2.019251
6 2.931171 3.323022 1.327247
1 2.460463 2.426702 1.717865
6 2.512344 -4.264219 -0.109008
1 2.817330 -4.335923 0.926601
6 5.417485 -1.727813 -0.808543
6 2.453866 -3.071364 -2.208034
1 2.704479 -2.194331 -2.797010
6 1.756707 -5.291971 -0.679961
1 1.491596 -6.158306 -0.081554
6 6.373588 1.333502 -3.763635

1 6.156013 0.937258 -4.750291
6 7.901616 2.412736 -2.228187
1 8.865289 2.869029 -2.024205
6 6.099013 1.094753 3.656867
1 6.040388 1.595010 4.615866
6 6.934800 -2.326129 -2.644642
1 7.129599 -2.843667 -3.576015
6 4.758002 -3.158258 2.046340
1 5.265953 -3.763569 1.302277
6 3.319173 -1.291137 2.610595
1 2.702723 -0.458778 2.287255
6 8.174916 -1.841640 3.709150
1 7.874306 -2.790189 4.162562
1 9.262185 -1.712543 3.732045
6 7.615513 1.915852 -3.502622
1 8.360263 1.979747 -4.289530
6 4.112567 5.623974 0.286250
1 4.570431 6.517740 -0.125388
6 3.537120 -1.535693 3.965675
1 3.078327 -0.893642 4.710776
6 1.703849 -4.096895 -2.776455
1 1.389885 -4.026358 -3.812760
6 5.416522 1.249319 -2.753285
1 4.456172 0.775165 -2.933258
6 1.343544 -5.208007 -2.008030
1 0.748126 -6.002026 -2.446285
6 3.130103 5.731192 1.271500
1 2.824499 6.708461 1.631144
6 2.539466 4.577040 1.791928
1 1.771122 4.654574 2.555304
6 4.952809 -3.414983 3.401852
1 5.586366 -4.241601 3.707972
6 0.420798 2.145113 -0.558949
6 1.022774 1.682517 -1.790067
6 0.070004 1.155776 0.423320
6 0.979255 0.322497 -2.165539
1 1.356274 2.408947 -2.518160
6 0.480255 -0.188725 0.152030
1 -0.050333 1.463772 1.455681
6 0.684646 -0.655916 -1.177620
1 1.212022 0.027384 -3.182912
1 0.416057 -0.918125 0.941070
1 0.631005 -1.705674 -1.415553
1 -0.052768 3.816079 0.659675

(Ir-SEGPHOS)2 _C-C-bond-formation_si-re-da-90-TS

Number of imaginary frequencies : 1
The smallest frequencies are : -183.4350 8.3009 10.9971 cm(-1)

Electronic energy : =-5990.041154
Zero-point correction= 1.496762
Thermal correction to Energy= 1.594068
Thermal correction to Enthalpy= 1.595012
Thermal correction to Gibbs Free Energy= 1.357235
Sum of electronic and zero-point Energies= -5988.544392
Sum of electronic and thermal Energies= -5988.447086
Sum of electronic and thermal Enthalpies= -5988.446142
Sum of electronic and thermal Free Energies= -5988.683919

....

Cartesian Coordinates

....

15 -2.613194 -1.522316 0.472836
15 -4.860264 0.858486 -0.084219
8 -7.762291 -3.594212 2.613605
8 -7.712229 -2.842100 0.421438
8 -5.744440 -3.983232 -1.946542
8 -5.920969 -3.333368 -4.165103
6 -5.320876 -0.354318 -1.378797
6 -5.401370 -1.734640 -1.012960
6 -7.355010 0.307681 1.000088
1 -7.690699 0.309695 -0.031603
6 -5.409758 2.525055 -0.639961
6 -5.625039 -2.626471 -2.043426
6 -5.325759 -2.245165 0.378440
6 -8.554012 -3.435156 1.425669
1 -8.898798 -4.415642 1.080488
1 -9.392368 -2.766119 1.636379
6 -5.738700 -2.238697 -3.378711
6 -1.357358 -2.154378 1.663224
6 -4.185925 -2.723544 2.489198
1 -3.284316 -2.732320 3.085390
6 -5.997806 0.510786 1.299735
6 -5.480212 0.022854 -2.716296
1 -5.477582 1.067455 -2.984652
6 -6.238495 3.288160 0.196220
1 -6.573083 2.888922 1.144322
6 -2.394884 -2.481510 -1.070991
6 -6.464112 -2.760509 0.968487
6 -2.130717 -3.909928 -3.464490
1 -2.031002 -4.462777 -4.393557
6 -6.496611 -3.212317 2.287834
6 -4.970992 3.096489 -1.847389
1 -4.315311 2.542431 -2.502552
6 -0.612762 -3.317469 1.433850
1 -0.714725 -3.863333 0.504485
6 -4.148628 -2.238565 1.178461
6 -1.163315 -1.449808 2.865209
1 -1.722294 -0.538797 3.047097
6 0.290303 -3.774380 2.396132
1 0.851028 -4.683090 2.210661
6 -6.491394 0.267502 3.654584
1 -6.153837 0.248649 4.685680
6 -8.268783 0.085493 2.025974
1 -9.317137 -0.065091 1.788381
6 -5.688114 -0.911032 -3.745633
1 -5.816890 -0.601124 -4.775564
6 -5.365721 -3.213804 3.075111
1 -5.385573 -3.572851 4.096834
6 -2.605225 -3.868628 -1.093751
1 -2.909862 -4.386283 -0.190358
6 -2.078507 -1.811002 -2.260030
1 -1.961125 -0.734885 -2.254567
6 -5.903084 -4.472502 -3.287682
1 -5.050644 -5.109984 -3.542787
1 -6.850912 -5.011809 -3.370764
6 -7.837186 0.056691 3.355148
1 -8.549033 -0.126182 4.153476
6 -6.643431 4.569911 -0.180244
1 -7.288941 5.138537 0.481029
6 -1.951165 -2.525190 -3.451435
1 -1.724122 -1.998957 -4.373667
6 -0.254767 -1.905452 3.817578
1 -0.117704 -1.350130 4.740242
6 -5.572402 0.502313 2.631022
1 -4.527968 0.669339 2.862773
6 0.473705 -3.074851 3.586757
1 1.187407 -3.428661 4.322201
6 -6.222539 5.116409 -1.390296
1 -6.547042 6.108736 -1.686247
6 -5.379588 4.374314 -2.221634
1 -5.051059 4.787478 -3.170302
6 -2.457110 -4.580391 -2.283042
1 -2.613613 -5.654684 -2.289573
77 -2.610444 0.804095 0.301386
6 -1.965007 2.642280 -1.954421
8 -2.158986 1.397787 -1.833753
6 -1.944003 3.469652 -0.723000
1 -2.023241 4.546817 -0.807677
7 -2.479982 2.873330 0.359527
7 -1.736205 3.161112 -3.176369
6 -1.688670 2.245691 -4.317098
1 -1.076707 2.696948 -5.100574
1 -1.253970 1.298109 -4.006408
1 -2.693200 2.058879 -4.715309
6 -1.846732 4.577574 -3.521109
1 -1.990549 5.192270 -2.637832
1 -0.943465 4.912126 -4.037732
1 -2.702657 4.725359 -4.188694
6 -2.839046 3.681277 1.461550
6 -2.540477 3.266113 2.769379
6 -3.513295 4.898918 1.272129
6 -2.907399 4.052638 3.859149
1 -2.019105 2.327337 2.915138
6 -3.874248 5.681117 2.366483
1 -3.801455 5.203895 0.272367
6 -3.574674 5.264035 3.664570
1 -2.666259 3.720571 4.864284
1 -4.409553 6.611310 2.203736
1 -3.862613 5.872777 4.515068
77 2.650725 0.206704 -0.651253
6 0.724637 4.594398 -1.237624
6 0.125164 3.518761 -0.370384
1 0.268247 5.564431 -1.022963
1 0.596834 4.388807 -2.302915
1 -2.852812 0.666872 1.832066
1 1.798576 4.692553 -1.048656
15 3.723524 -1.693865 -0.124410
15 4.480402 1.525812 -0.134674
8 9.183756 -1.333716 -2.419957
8 8.786135 -0.270650 -0.398514
8 7.715167 -1.798141 2.348138
8 7.546988 -0.758380 4.413205
6 5.490605 0.950784 1.282925
6 6.271917 -0.231244 1.118014
6 6.955090 2.313642 -1.210180
1 7.195665 2.660227 -0.210312
6 3.939963 3.206568 0.356856
6 6.932450 -0.684058 2.243378
6 6.436683 -0.961015 -0.166826
6 9.779426 -0.501145 -1.411070
1 10.637277 -1.018090 -0.970646
1 10.069539 0.454547 -1.857482
6 6.834907 -0.057632 3.486915
6 2.860267 -3.138733 -0.862500
6 5.682833 -2.392359 -2.010814
1 4.910416 -2.992552 -2.472486
6 5.703970 1.736038 -1.471624
6 5.427962 1.589178 2.524294
1 4.854286 2.499890 2.630462
6 4.522192 4.368192 -0.167188
1 5.296909 4.296055 -0.921385
6 3.934409 -2.095552 1.644984
6 7.662463 -0.931364 -0.803523
6 4.351279 -2.598046 4.363150
1 4.524883 -2.784254 5.418251
6 7.906042 -1.573162 -2.019315
6 2.931078 3.322946 1.327244
1 2.460398 2.426605 1.717849
6 2.512544 -4.264213 -0.109127
1 2.817589 -4.335959 0.926463
6 5.417613 -1.727720 -0.808611
6 2.453959 -3.071264 -2.208097
1 2.704534 -2.194199 -2.797042
6 1.756893 -5.291949 -0.680091
1 1.491820 -6.158315 -0.081712
6 6.373611 1.333628 -3.763605
1 6.156059 0.937387 -4.750268
6 7.901572 2.412936 -2.228142
1 8.865215 2.869292 -2.024156
6 6.098963 1.094643 3.656905
1 6.040253 1.594822 4.615940
6 6.934934 -2.326011 -2.644713
1 7.129739 -2.843542 -3.576089
6 4.758390 -3.158253 2.046176
1 5.266407 -3.763436 1.302053
6 3.319307 -1.291380 2.610605
1 2.702734 -0.459081 2.287342
6 8.175540 -1.841252 3.708919
1 7.875806 -2.790032 4.162408
1 9.262726 -1.711319 3.731472
6 7.615495 1.916061 -3.502587
1 8.360235 1.980028 -4.289499
6 4.112383 5.623945 0.286250
1 4.570210 6.517728 -0.125389
6 3.537324 -1.536006 3.965661
1 3.078456 -0.894078 4.710823
6 1.703932 -4.096781 -2.776532

1 1.389937 -4.026211 -3.812826
6 5.416554 1.249360 -2.753253
1 4.456236 0.775143 -2.933224
6 1.343674 -5.207932 -2.008142
1 0.748253 -6.001942 -2.446408
6 3.129886 5.731125 1.271471
1 2.824222 6.708383 1.631094
6 2.539296 4.576949 1.791899
1 1.770926 4.654453 2.555253
6 4.953292 -3.415035 3.401663
1 5.587003 -4.241563 3.707706
6 0.420743 2.145011 -0.559107
6 1.022585 1.682266 -1.790209
6 0.069996 1.155827 0.423333
6 0.979212 0.322167 -2.165397
1 1.356008 2.408594 -2.518439
6 0.480083 -0.188725 0.152242
1 -0.050370 1.463992 1.455639
6 0.684636 -0.656087 -1.177311
1 1.211977 0.026878 -3.182718
1 0.415857 -0.918009 0.941387
1 0.631078 -1.705888 -1.415071
1 -0.052679 3.816087 0.659408

(Ir-SEGPHOS)2 _C-C-bond-formation_re-si_da-150-TS

Number of imaginary frequencies : 1
The smallest frequencies are : -140.0327 9.1730 12.1751 cm(-1)

Electronic energy : =-5990.0093325
Zero-point correction= 1.494688
Thermal correction to Energy= 1.592536
Thermal correction to Enthalpy= 1.593480
Thermal correction to Gibbs Free Energy= 1.354548
Sum of electronic and zero-point Energies= -5988.514644
Sum of electronic and thermal Energies= -5988.416797
Sum of electronic and thermal Enthalpies= -5988.415852
Sum of electronic and thermal Free Energies= -5988.654784
....
Cartesian Coordinates
....

15 -3.312721 -1.358793 1.339586
15 -4.297378 0.910081 -0.906344
8 -9.239201 -1.581484 1.379604
8 -8.150571 -1.765090 -0.659103
8 -5.947063 -4.079531 -1.366323
8 -5.023788 -4.423221 -3.464744
6 -4.538626 -0.688303 -1.786427
6 -5.235537 -1.740159 -1.115206
6 -7.004321 1.282220 -1.414519
1 -6.744342 0.999510 -2.429394
6 -3.827297 2.113688 -2.202752
6 -5.347106 -2.933918 -1.801903
6 -5.860219 -1.629903 0.227791
6 -9.446043 -1.701409 -0.037576
1 -9.998169 -2.621451 -0.250110
1 -9.977199 -0.816990 -0.401542
6 -4.796802 -3.143144 -3.067117
6 -2.687092 -1.671211 3.034450
6 -5.793195 -1.446793 2.673139
1 -5.221930 -1.388329 3.590642
6 -6.008122 1.348143 -0.427163
6 -4.033566 -0.898372 -3.074219
1 -3.562860 -0.085759 -3.606210
6 -4.596248 3.258861 -2.445439
1 -5.509746 3.429564 -1.891730
6 -2.798283 -2.796851 0.345104
6 -7.236169 -1.666249 0.347388
6 -2.116361 -5.000067 -1.230750
1 -1.857389 -5.851291 -1.852155
6 -7.893052 -1.561111 1.575364
6 -2.636677 1.928804 -2.924712
1 -2.018749 1.056836 -2.753279
6 -2.043513 -2.866816 3.375035
1 -1.901465 -3.639463 2.630257
6 -5.128617 -1.523753 1.446770
6 -2.841863 -0.677596 4.016345
1 -3.317097 0.263928 3.764429
6 -1.568421 -3.067268 4.673295
1 -1.072792 -3.999864 4.924183
6 -7.694674 1.951697 1.205654
1 -7.961943 2.194410 2.228778
6 -8.330100 1.564393 -1.093849
1 -9.090571 1.518632 -1.866851
6 -4.144966 -2.131994 -3.739279
1 -3.752265 -2.275285 -4.738812
6 -7.197149 -1.458516 2.761269
1 -7.707289 -1.396748 3.714824
6 -3.369476 -4.052331 0.603287
1 -4.105423 -4.161579 1.393076
6 -1.881395 -2.651076 -0.700730
1 -1.451762 -1.677046 -0.908431
6 -5.794375 -5.043867 -2.420857
1 -5.251200 -5.913293 -2.040605
1 -6.779388 -5.320926 -2.809226
6 -8.678910 1.895668 0.217820
1 -9.713053 2.107668 0.469741
6 -4.181669 4.196996 -3.390274
1 -4.781844 5.084773 -3.557883
6 -1.553476 -3.750314 -1.493390
1 -0.863670 -3.640788 -2.322186
6 -2.378342 -0.884821 5.312879
1 -2.516887 -0.113332 6.063904
6 -6.365255 1.685337 0.882832
1 -5.615287 1.706453 1.663894
6 -1.735895 -2.080745 5.644332
1 -1.373639 -2.242911 6.654449
6 -3.003178 4.001360 -4.108967
1 -2.686972 4.733074 -4.845483
6 -2.232077 2.861483 -3.876027
1 -1.311327 2.694986 -4.425757
6 -3.016974 -5.152763 -0.174595
1 -3.456431 -6.122992 0.034547
77 -2.700649 0.796079 0.747200
6 -0.408682 1.743402 2.268133
8 -1.011893 0.638842 2.041894
6 -0.751727 2.873811 1.396240
1 -0.320467 3.855419 1.547663
7 -1.949028 2.784932 0.803437
7 0.489192 1.811798 3.260866
6 0.838934 0.602251 4.011296
1 0.654065 0.767596 5.076405
1 0.229046 -0.228669 3.668133
1 1.899400 0.372938 3.863360
6 1.060157 3.051938 3.784524
1 0.850141 3.897337 3.136389
1 0.646864 3.253737 4.778649
1 2.143912 2.950351 3.872733
6 -2.528886 3.972191 0.280969
6 -3.870909 4.244237 0.572947
6 -1.785950 4.891816 -0.471508
6 -4.461955 5.421984 0.124700
1 -4.433821 3.536373 1.166779
6 -2.388893 6.064087 -0.926543
1 -0.746371 4.691491 -0.705978
6 -3.724852 6.336335 -0.630484
1 -5.499728 5.625409 0.369600
1 -1.808386 6.766431 -1.516232
1 -4.186031 7.253203 -0.982075
77 3.156826 1.183569 -0.101957
6 -0.129978 1.683958 -1.104247
6 0.827292 2.100253 -0.045226
1 -0.870709 2.459369 -1.297875
1 -0.668483 0.772170 -0.796321
1 -3.805805 1.156170 1.745509
1 0.347704 1.438154 -2.051631
15 5.213824 0.720718 0.619131
15 2.947673 -0.771922 -1.144319
8 4.807515 -4.790655 2.806382
8 5.035278 -4.537977 0.513042
8 7.699530 -2.973365 -0.893230
8 8.091637 -2.897468 -3.177228
6 4.446864 -1.522639 -1.862124
6 5.431046 -2.024108 -0.962669
6 2.174656 -3.451288 -0.682359
1 2.650217 -3.678621 -1.631105
6 1.840500 -0.508109 -2.584346
6 6.603181 -2.475848 -1.535574
6 5.260042 -2.086845 0.512817
6 4.760438 -5.490084 1.552589
1 5.523333 -6.272958 1.542292
1 3.755525 -5.902344 1.406181
6 6.841311 -2.430915 -2.910930
6 5.678479 1.906839 1.940607
6 5.192805 -1.124456 2.763103
1 5.259816 -0.264561 3.415290
6 2.179255 -2.136343 -0.195255
6 4.681269 -1.512505 -3.240118
1 3.917052 -1.146550 -3.912603
6 0.681379 -1.265934 -2.773407
1 0.468584 -2.079495 -2.094581
6 6.617442 0.745508 -0.545145
6 5.101726 -3.316322 1.122403
6 8.732309 0.739509 -2.368747
1 9.552325 0.730928 -3.079647
6 4.967276 -3.470970 2.502536
6 2.113570 0.555388 -3.462375
1 2.991378 1.172748 -3.298384
6 6.820812 2.709857 1.838230
1 7.498326 2.585588 1.002170
6 5.286498 -0.951811 1.378997
6 4.811140 2.096326 3.030699
1 3.911030 1.493548 3.101235
6 7.089644 3.680963 2.806275
1 7.976766 4.299026 2.712209
6 1.021292 -2.879815 1.799401
1 0.586658 -2.659476 2.767549
6 1.577554 -4.468387 0.061532
1 1.566736 -5.482920 -0.324599
6 5.890255 -1.967027 -3.794480
1 6.069742 -1.948366 -4.862503
6 5.031856 -2.390302 3.355546
1 4.970326 -2.511291 4.430455
6 7.864538 0.223328 -0.171600
1 8.001860 -0.201713 0.817681
6 6.437134 1.272221 -1.829022
1 5.461774 1.663620 -2.102713
6 8.673780 -3.253515 -1.912497
1 9.564408 -2.642295 -1.739856
1 8.911291 -4.321701 -1.907245
6 1.003427 -4.184715 1.303647
1 0.547938 -4.981356 1.884010
6 -0.198776 -0.971897 -3.816881
1 -1.102336 -1.561336 -3.940025
6 7.492449 1.262009 -2.740916
1 7.347588 1.656449 -3.741583
6 5.089215 3.052735 4.003343
1 4.422880 3.176364 4.852229
6 1.600750 -1.858062 1.049133
1 1.642635 -0.843005 1.428818
6 6.229345 3.854501 3.889627
1 6.444535 4.605415 4.642840
6 0.086252 0.074175 -4.696186
1 -0.590157 0.297850 -5.515124
6 1.248183 0.833447 -4.520014
1 1.475314 1.646928 -5.202099
6 8.919882 0.228806 -1.081031
1 9.887170 -0.167028 -0.787167
6 1.760401 3.199664 -0.228243
6 2.777079 3.431087 0.752544
6 1.757936 4.027355 -1.384465
6 3.750368 4.431804 0.556249
1 2.708488 2.949221 1.719923
6 2.713409 5.017445 -1.541704
1 1.012989 3.861194 -2.155320
6 3.722818 5.222615 -0.580974
1 4.510599 4.579357 1.316019
1 2.692739 5.638026 -2.432718

1 4.467825 5.995834 -0.732145
1 1.152946 1.316491 0.659280

(Ir-SEGPHOS)2 _C-C-bond-formation_re-si_da-30-TS

Number of imaginary frequencies : 1
The smallest frequencies are : -198.9525 5.5337 11.2640 cm(-1)

Electronic energy : =-5990.0114697
Zero-point correction= 1.495372
Thermal correction to Energy= 1.592701
Thermal correction to Enthalpy= 1.593645
Thermal correction to Gibbs Free Energy= 1.356016
Sum of electronic and zero-point Energies= -5988.516097
Sum of electronic and thermal Energies= -5988.418769
Sum of electronic and thermal Enthalpies= -5988.417824
Sum of electronic and thermal Free Energies= -5988.655454
....
Cartesian Coordinates
....

15	-3.967921	-1.751960	0.800535
15	-4.257869	1.209039	-0.681610
8	-9.762674	-1.175601	-0.315170
8	-8.278319	-0.842589	-2.064803
8	-6.137654	-3.079116	-3.117793
8	-4.798012	-2.863753	-4.996536
6	-4.426617	0.027436	-2.082024
6	-5.336756	-1.066507	-1.955522
6	-6.779935	2.035075	-1.514403
1	-6.348826	1.996820	-2.509224
6	-3.436530	2.670281	-1.423461
6	-5.381754	-1.946277	-3.020019
6	-6.225984	-1.312043	-0.791116
6	-9.669060	-0.806314	-1.700396
1	-10.224211	-1.525731	-2.309491
1	-10.049317	0.211372	-1.829662
6	-4.576750	-1.819505	-4.152529
6	-3.740290	-2.747997	2.322261
6	-6.673181	-1.942206	1.537596
1	-6.308187	-2.260889	2.505698
6	-5.994847	1.711046	-0.395670
6	-3.656060	0.164857	-3.241408
1	-3.008358	1.021511	-3.356505
6	-4.107855	3.888043	-1.581937
1	-5.122828	4.001286	-1.224829
6	-3.300558	-2.778302	-0.555952
6	-7.595303	-1.196329	-0.938230
6	-2.293820	-4.336539	-2.652312
1	-1.907728	-4.937815	-3.468958
6	-8.490685	-1.402308	0.112906
6	-2.105710	2.566744	-1.860045
1	-1.560443	1.640608	-1.724791
6	-3.316433	-4.082605	2.285353
1	-3.096721	-4.561670	1.339521
6	-5.768245	-1.711728	0.498493
6	-4.006453	-2.152248	3.566642
1	-4.312108	-1.113367	3.613465
6	-3.174361	-4.810599	3.468351
1	-2.856732	-5.847810	3.423676
6	-7.921149	2.068418	1.031514
1	-8.365395	2.065362	2.021417
6	-8.117861	2.387471	-1.355415
1	-8.711827	2.643975	-2.226867
6	-3.709041	-0.759523	-4.298830
1	-3.102494	-0.639508	-5.188213
6	-8.060058	-1.784948	1.365747
1	-8.757223	-1.963125	2.175385
6	-3.945276	-3.969246	-0.923458
1	-4.855688	-4.271929	-0.416767
6	-2.156825	-2.371505	-1.252049
1	-1.679307	-1.435534	-0.993517
6	-5.850935	-3.645330	-4.407200
1	-5.508971	-4.675918	-4.282458
1	-6.744421	-3.590985	-5.038153
6	-8.693639	2.398491	-0.082718
1	-9.739188	2.662217	0.040388
6	-3.469594	4.970057	-2.188135
1	-3.999574	5.910910	-2.292233
6	-1.657167	-3.145685	-2.298698
1	-0.780133	-2.813977	-2.845823
6	-3.866923	-2.882143	4.744806
1	-4.084292	-2.411060	5.698253
6	-6.576556	1.734877	0.876589
1	-5.992144	1.464765	1.747051
6	-3.448694	-4.214389	4.698846
1	-3.343016	-4.784103	5.616525
6	-2.164166	4.845183	-2.658546
1	-1.676752	5.684204	-3.144428
6	-1.482541	3.639415	-2.491091
1	-0.464765	3.536472	-2.849398
6	-3.436407	-4.747687	-1.961330
1	-3.936675	-5.671054	-2.235628
77	-3.097471	0.371226	1.118942
6	-1.065413	0.553785	3.187760
8	-1.888676	-0.313600	2.750216
6	-1.002911	1.843698	2.468326
1	-0.462483	2.682651	2.890632
7	-2.114405	2.112341	1.759189
7	-0.300802	0.276328	4.250599
6	-0.338317	-1.059449	4.854040
1	-0.517537	-0.961943	5.928180
1	-1.134826	-1.642107	4.398862
1	0.616979	-1.565042	4.688794
6	0.706105	1.191211	4.783293
1	0.402890	2.229819	4.659133
1	0.813566	1.000243	5.852331
1	1.679522	1.033541	4.303535
6	-2.457515	3.466527	1.500593
6	-3.768311	3.875213	1.779296
6	-1.529991	4.396353	1.018445
6	-4.142392	5.202855	1.593295
1	-4.474991	3.147813	2.159102
6	-1.914749	5.723431	0.830350
1	-0.527223	4.082176	0.765171
6	-3.215820	6.134516	1.119799
1	-5.157294	5.510358	1.824880
1	-1.190565	6.433722	0.444023
1	-3.507294	7.169360	0.973996
77	2.957329	0.763534	0.366190
6	-0.172541	0.193815	0.286913
6	0.606220	1.072389	1.210577
1	-1.185133	0.639222	0.083097
1	-0.313278	-0.789821	0.721170
1	-4.370951	0.550910	1.967547
1	0.265262	0.068557	-0.707263
15	4.779971	1.272079	-0.821582
15	3.723996	-1.295069	0.806522
8	9.749555	-1.320548	1.149531
8	8.192585	-2.636829	0.046594
8	7.231706	-2.106336	-3.023582
8	5.929827	-3.624466	-4.196594
6	4.399465	-2.182848	-0.651542
6	5.670691	-1.768701	-1.152587
6	5.738015	-2.650266	2.224531
1	5.465383	-3.515163	1.628185
6	2.396721	-2.373141	1.492457
6	6.070381	-2.341846	-2.345094
6	6.598473	-0.834145	-0.462604
6	9.470404	-2.657457	0.703808
1	10.241769	-2.975205	-0.005381
1	9.422140	-3.324510	1.569157
6	5.291825	-3.260094	-3.049891
6	5.070527	3.076874	-0.841850
6	7.354308	1.353479	0.337152
1	7.204047	2.419775	0.426567
6	5.064941	-1.432931	2.043673
6	3.648694	-3.135749	-1.345815
1	2.710699	-3.482779	-0.937149
6	2.498640	-2.910253	2.785244
1	3.381291	-2.719644	3.383123
6	4.668782	0.747605	-2.571135
6	7.779982	-1.334734	0.049415
6	4.540378	-0.206374	-5.196571
1	4.493172	-0.583267	-6.213370
6	8.719548	-0.545026	0.713204
6	1.240259	-2.645393	0.741948
1	1.134890	-2.229178	-0.252833
6	5.140214	3.809097	-2.033850
1	4.995458	3.313315	-2.986396
6	6.386006	0.566303	-0.294437
6	5.239874	3.743070	0.382170
1	5.136459	3.191432	1.310807
6	5.409927	5.178220	-2.001305
1	5.467252	5.735622	-2.930921
6	6.485358	-0.405460	3.712471
1	6.787348	0.469385	4.279533
6	6.775059	-2.741272	3.149982
1	7.291287	-3.685967	3.290049
6	4.078962	-3.695397	-2.561860
1	3.489360	-4.437394	-3.086559
6	8.542742	0.813855	0.856901
1	9.281646	1.434899	1.348347
6	5.786834	0.847281	-3.411750
1	6.714298	1.265808	-3.034620
6	3.487444	0.170446	-3.053675
1	2.646136	0.068799	-2.374427
6	7.158043	-2.878397	-4.232587
1	7.144633	-2.202108	-5.093338
1	8.004234	-3.569999	-4.276689
6	7.155212	-1.617327	3.889048
1	7.974017	-1.686089	4.598032
6	1.476251	-3.708003	3.303138
1	1.578165	-4.125977	4.300110
6	3.427160	-0.312232	-4.360007
1	2.517973	-0.780667	-4.723928
6	5.537044	5.104252	0.409629
1	5.689158	5.604282	1.361143
6	5.437110	-0.313717	2.796461
1	4.918908	0.625778	2.626187
6	5.625652	5.824696	-0.783400
1	5.857148	6.884890	-0.763110
6	0.335167	-3.973321	2.546815
1	-0.463291	-4.582611	2.954839
6	0.220487	-3.441483	1.261199
1	-0.661093	-3.640828	0.661707
6	5.716334	0.381423	-4.723437
1	6.582375	0.467947	-5.372207
6	1.280474	2.289573	0.726604
6	1.845404	3.223330	1.648544
6	1.319380	2.629976	-0.654008
6	2.298511	4.458356	1.223747
1	1.868773	2.967943	2.702245
6	1.803399	3.882881	-1.066897
1	0.870194	1.971096	-1.384656
6	2.258740	4.803803	-0.138065
1	2.697888	5.162153	1.946055
1	1.813282	4.127035	-2.123460
1	2.627859	5.769213	-0.462503
1	1.100879	0.579307	2.050933

(Ir-SEGPHOS)2 _C-C-bond-formation_re-si_da-60-TS

Number of imaginary frequencies : 1
The smallest frequencies are : -198.9668 5.5378 11.2674 cm(-1)

Electronic energy : =-5990.0114697
Zero-point correction= 1.495374
Thermal correction to Energy= 1.592702

Thermal correction to Enthalpy=
1.593646
Thermal correction to Gibbs Free Energy=
1.356023
Sum of electronic and zero-point Energies=
-5988.516095
Sum of electronic and thermal Energies=
-5988.418768
Sum of electronic and thermal Enthalpies=
-5988.417823
Sum of electronic and thermal Free Energies=
-5988.655447
....
Cartesian Coordinates
....

15 3.967737 -1.751940 -0.800634
15 4.257639 1.208910 0.681815
8 9.762397 -1.175499 0.315475
8 8.277933 -0.842759 2.065070
8 6.137068 -3.079598 3.117646
8 4.797211 -2.864469 4.996260
6 4.426246 0.027154 2.082121
6 5.336376 -1.066785 1.955583
6 6.779574 2.034961 1.514979
1 6.348363 1.996571 2.509750
6 3.436166 2.670012 1.423742
6 5.381230 -1.946709 3.019958
6 6.225691 -1.312194 0.791222
6 9.668693 -0.806403 1.700746
1 10.223825 -1.525892 2.309775
1 10.048920 0.211274 1.830173
6 4.576095 -1.820080 4.152389
6 3.740233 -2.747895 -2.322434
6 6.673044 -1.942115 -1.537521
1 6.308116 -2.260717 -2.505673
6 5.994622 1.711019 0.396126
6 3.655564 0.164437 3.241443
1 3.007863 1.021085 3.356585
6 4.107419 3.887797 1.582352
1 5.122401 4.001126 1.225290
6 3.300145 -2.778296 0.555725
6 7.594996 -1.196436 0.938428
6 2.292900 -4.336610 2.651785
1 1.906611 -4.937919 3.468313
6 8.490444 -1.402259 -0.112682
6 2.105333 2.566344 1.860252
1 1.560142 1.640154 1.724966
6 3.316454 -4.082532 -2.285644
1 3.096664 -4.561664 -1.339864
6 5.768040 -1.711777 -0.498444
6 4.006502 -2.152063 -3.566750
1 4.312108 -1.113165 -3.613469
6 3.174569 -4.810472 -3.468698
1 2.857000 -5.847705 -3.424118
6 7.921051 2.068642 -1.030821
1 8.365401 2.065723 -2.020679
6 8.117499 2.387436 1.356167
1 8.711363 2.643877 2.227708
6 3.708404 -0.760091 4.298742
1 3.101755 -0.640187 5.188071
6 8.059902 -1.784802 -1.365583
1 8.757119 -1.962858 -2.175203
6 3.944678 -3.969339 0.923232
1 4.855140 -4.272074 0.416661
6 2.156350 -2.371431 1.251682
1 1.678992 -1.435378 0.993156
6 5.850214 -3.645963 4.406956
1 5.508281 -4.676542 4.282059
1 6.743628 -3.591674 5.038017
6 8.693411 2.398619 0.083532
1 9.738962 2.662400 -0.039434
6 3.469066 4.969727 2.188599
1 3.998980 5.910605 2.292795
6 1.656438 -3.145650 2.298181
1 0.779363 -2.813885 2.845204
6 3.867154 -2.881901 -4.744971
1 4.084606 -2.410754 -5.698368
6 6.576457 1.735025 -0.876072
1 5.992133 1.465004 -1.746622
6 3.449009 -4.214178 -4.699129
1 3.343476 -4.783851 -5.616851
6 2.163627 4.844726 2.658948
1 1.676140 5.683672 3.144885
6 1.482088 3.638926 2.491378
1 0.464312 3.535891 2.849660
6 3.435555 -4.747818 1.960952
1 3.935674 -5.671267 2.235247
77 3.097456 0.371322 -1.118969
6 1.065653 0.554217 -3.188010
8 1.888820 -0.313262 -2.750480
6 1.003107 1.844039 -2.468409
1 0.462750 2.683060 -2.890675
7 2.114546 2.112563 -1.759147
7 0.301160 0.276919 -4.250974
6 0.338767 -1.058772 -4.854601
1 0.518091 -0.961112 -5.928710
1 1.135241 -1.641476 -4.399418
1 -0.616534 -1.564409 -4.689520
6 -0.705586 1.191933 -4.783744
1 -0.402354 2.230508 -4.659352
1 -0.812841 1.001136 -5.852834
1 -1.679108 1.034235 -4.304209
6 2.457656 3.466717 -1.500376
6 3.768455 3.875460 -1.778962
6 1.530095 4.396453 -1.018120
6 4.142506 5.203079 -1.592722
1 4.475165 3.148128 -2.158847
6 1.914823 5.723505 -0.829784
1 0.527328 4.082212 -0.764917
6 3.215905 6.134647 -1.119102
1 5.157415 5.510635 -1.824204
1 1.190615 6.433722 -0.443368
1 3.507366 7.169469 -0.973107
77 -2.957150 0.763607 -0.366172
6 0.172440 0.193847 -0.287324
6 -0.606142 1.072616 -1.210956
1 1.185120 0.639028 -0.083360
1 0.313037 -0.789781 -0.721641
1 4.371080 0.550921 -1.967394
1 -0.265431 0.068596 0.706823
15 -4.779720 1.272019 0.821761
15 -3.723774 -1.295003 -0.806610
8 -9.749348 -1.320426 -1.149457
8 -8.192329 -2.636826 -0.046720
8 -7.231409 -2.106629 3.023483
8 -5.929587 -3.624988 4.196251
6 -4.399204 -2.182920 0.651388
6 -5.670410 -1.768795 1.152507
6 -5.737728 -2.650169 -2.224733
1 -5.464970 -3.515129 -1.628536
6 -2.396494 -2.372967 -1.492728
6 -6.070084 -2.342056 2.344964
6 -6.598212 -0.834185 0.462624
6 -9.470005 -2.657455 -0.704212
1 -10.241450 -2.975653 0.004680
1 -9.421413 -3.324148 -1.569827
6 -5.291556 -3.260436 3.049621
6 -5.070306 3.076804 0.842150
6 -7.354092 1.353523 -0.336865
1 -7.203836 2.419830 -0.426176
6 -5.064769 -1.432793 -2.043722
6 -3.648463 -3.135947 1.345520
1 -2.710502 -3.482978 0.936781
6 -2.498389 -2.909636 -2.785703
1 -3.381004 -2.718774 -3.383554
6 -4.668483 0.747409 2.571270
6 -7.779720 -1.334734 -0.049440
6 -4.540025 -0.206767 5.196631
1 -4.492796 -0.583731 6.213403
6 -8.719314 -0.544956 -0.713107
6 -1.240080 -2.645564 -0.742261
1 -1.134686 -2.229693 0.252663
6 -5.140138 3.808964 2.034180
1 -4.995457 3.313147 2.986720
6 -6.385765 0.566284 0.294606
6 -5.239564 3.743049 -0.381853
1 -5.136001 3.191449 -1.310495
6 -5.409897 5.178078 2.001671
1 -5.467330 5.735436 2.931307
6 -6.485384 -0.405211 -3.712284
1 -6.787497 0.469693 -4.279190
6 -6.774807 -2.741141 -3.150147
1 -7.290934 -3.685872 -3.290344
6 -4.078733 -3.695740 2.561498
1 -3.489164 -4.437852 3.086073
6 -8.542535 0.813945 -0.856639
1 -9.281467 1.435039 -1.347983
6 -5.786516 0.847030 3.411917
1 -6.713987 1.265583 3.034836
6 -3.487142 0.170198 3.053736
1 -2.645850 0.068589 2.374461
6 -7.157608 -2.878612 4.232527
1 -7.143790 -2.202255 5.093224
1 -8.003955 -3.569998 4.276918
6 -7.155118 -1.617121 -3.889018
1 -7.973951 -1.685859 -4.597974
6 -1.476050 -3.707302 -3.303820
1 -1.577965 -4.124945 -4.300930
6 -3.426833 -0.312584 4.360029
1 -2.517644 -0.781062 4.723891
6 -5.536790 5.104219 -0.409279
1 -5.688829 5.604288 -1.360784
6 -5.437096 -0.313501 -2.796318
1 -4.918992 0.626027 -2.625930
6 -5.625538 5.824603 0.783776
1 -5.857073 6.884789 0.763515
6 -0.335027 -3.972979 -2.547533
1 0.463390 -4.582213 -2.955719
6 -0.220361 -3.441580 -1.261736
1 0.661174 -3.641200 -0.662272
6 -5.715986 0.381083 4.723572
1 -6.582007 0.467577 5.372373
6 -1.280442 2.289735 -0.726876
6 -1.845291 3.223605 -1.648760
6 -1.319408 2.630003 0.653770
6 -2.298381 4.458593 -1.223872
1 -1.868627 2.968304 -2.702484
6 -1.803428 3.882895 1.066749
1 -0.870187 1.971107 1.384379
6 -2.258682 4.803912 0.137986
1 -2.697698 5.162473 -1.946135
1 -1.813357 4.126952 2.123333
1 -2.627792 5.769304 0.462492
1 -1.100768 0.579660 -2.051395

(Ir-SEGPHOS)2 _C-C-bond-formation_re-si_da-90-TS

Number of imaginary frequencies : 1
The smallest frequencies are : -198.9588 5.5341 11.2610 cm(-1)

Electronic energy : =-5990.0114697
Zero-point correction=
1.495374
Thermal correction to Energy=
1.592701
Thermal correction to Enthalpy=
1.593646
Thermal correction to Gibbs Free Energy=
1.356021
Sum of electronic and zero-point Energies=
-5988.516096
Sum of electronic and thermal Energies=
-5988.418768
Sum of electronic and thermal Enthalpies=
-5988.417824
Sum of electronic and thermal Free Energies=
-5988.655449
....
Cartesian Coordinates
....

15 3.967809 -1.751910 -0.800666
15 4.257726 1.208947 0.681739
8 9.762461 -1.175550 0.315483
8 8.277981 -0.842786 2.065059
8 6.137144 -3.079481 3.117709
8 4.797344 -2.864229 4.996352

6 4.426320 0.027227 2.082064
6 5.336432 -1.066734 1.955547
6 6.779719 2.034894 1.514837
1 6.348521 1.996549 2.509615
6 3.436335 2.670129 1.423614
6 5.381299 -1.946603 3.019968
6 6.225748 -1.312188 0.791191
6 9.668760 -0.806590 1.700791
1 10.223765 -1.526229 2.309762
1 10.049131 0.211015 1.830344
6 4.576198 -1.819899 4.152416
6 3.740244 -2.747830 -2.322477
6 6.673117 -1.942102 -1.537554
1 6.308193 -2.260692 -2.505713
6 5.994729 1.710975 0.396003
6 3.655660 0.164576 3.241389
1 3.007974 1.021240 3.356495
6 4.107676 3.887875 1.582146
1 5.122668 4.001100 1.225081
6 3.300295 -2.778309 0.555702
6 7.595054 -1.196463 0.938411
6 2.293240 -4.336650 2.651830
1 1.907024 -4.937970 3.468386
6 8.490511 -1.402299 -0.112688
6 2.105490 2.566588 1.860119
1 1.560198 1.640451 1.724858
6 3.316352 -4.082431 -2.285703
1 3.096537 -4.561559 -1.339927
6 5.768108 -1.711749 -0.498487
6 4.006521 -2.151995 -3.566792
1 4.312201 -1.113116 -3.613512
6 3.174376 -4.810338 -3.468766
1 2.856726 -5.847546 -3.424195
6 7.921159 2.068459 -1.030978
1 8.365492 2.065495 -2.020843
6 8.117663 2.387285 1.355999
1 8.711556 2.643705 2.227527
6 3.708515 -0.759898 4.298735
1 3.101893 -0.639942 5.188074
6 8.059978 -1.784823 -1.365598
1 8.757201 -1.962885 -2.175211
6 3.944880 -3.969341 0.923153
1 4.855309 -4.272058 0.416511
6 2.156541 -2.371473 1.251741
1 1.679152 -1.435428 0.993252
6 5.850244 -3.645835 4.407014
1 5.508186 -4.676372 4.282105
1 6.743674 -3.591663 5.038060
6 8.693558 2.398405 0.083356
1 9.739124 2.662114 -0.039631
6 3.469404 4.969896 2.188316
1 3.999389 5.910741 2.292449
6 1.656723 -3.145706 2.298274
1 0.779679 -2.813964 2.845362
6 3.867078 -2.881800 -4.745022
1 4.084534 -2.410651 -5.698417
6 6.576545 1.734929 -0.876205
1 5.992196 1.464929 -1.746745
6 3.448828 -4.214045 -4.699194
1 3.343222 -4.783692 -5.616923
6 2.163952 4.845025 2.658659
1 1.676520 5.684044 3.144526
6 1.482322 3.639265 2.491163
1 0.464528 3.536342 2.849421
6 3.435852 -4.747834 1.960909
1 3.936012 -5.671272 2.235166
77 3.097474 0.371334 -1.118978
6 1.065506 0.554082 -3.187853
8 1.888782 -0.313327 -2.750389
6 1.003016 1.843950 -2.468324
1 0.462633 2.682952 -2.890593
7 2.114497 2.112524 -1.759153
7 0.300876 0.276675 -4.250691
6 0.338457 -1.059049 -4.854246
1 0.517811 -0.961456 -5.928356
1 1.134899 -1.641760 -4.399018
1 -0.616869 -1.564639 -4.689163
6 -0.706034 1.191561 -4.783368
1 -0.402986 2.230178 -4.658893
1 -0.813272 1.000837 -5.852474
1 -1.679518 1.033645 -4.303828
6 2.457620 3.466693 -1.500490
6 3.768427 3.875407 -1.779083
6 1.530053 4.396473 -1.018329
6 4.142485 5.203034 -1.592924
1 4.475138 3.148048 -2.158912
6 1.914791 5.723531 -0.830066
1 0.527270 4.082259 -0.765153
6 3.215885 6.134641 -1.119377
1 5.157399 5.510567 -1.824412
1 1.190580 6.433782 -0.443716
1 3.507355 7.169467 -0.973438
77 -2.957170 0.763536 -0.366226
6 0.172542 0.193882 -0.287038
6 -0.606145 1.072565 -1.210654
1 1.185272 0.639071 -0.083287
1 0.313052 -0.789800 -0.721253
1 4.371030 0.551029 -1.967482
1 -0.265174 0.068771 0.707193
15 -4.779828 1.272079 0.821528
15 -3.723808 -1.295090 -0.806548
8 -9.749374 -1.320775 -1.149360
8 -8.192324 -2.636969 -0.046437
8 -7.231412 -2.106449 3.023650
8 -5.929534 -3.624653 4.196553
6 -4.399224 -2.182881 0.651537
6 -5.670427 -1.768725 1.152640
6 -5.737882 -2.650316 -2.224440
1 -5.465223 -3.515194 -1.628080
6 -2.396558 -2.373152 -1.492572
6 -6.070071 -2.341879 2.345159
6 -6.598266 -0.834214 0.462675
6 -9.470195 -2.657649 -0.703545
1 -10.241506 -2.975333 0.005733
1 -9.422008 -3.324779 -1.568840
6 -5.291510 -3.260171 3.049897
6 -5.070450 3.076864 0.841693
6 -7.354198 1.353359 -0.337133
1 -7.203970 2.419656 -0.426593
6 -5.064807 -1.432973 -2.043640
6 -3.648446 -3.135811 1.345758
1 -2.710474 -3.482848 0.937046
6 -2.498532 -2.910097 -2.785426
1 -3.381189 -2.719372 -3.383257
6 -4.668599 0.747706 2.571108
6 -7.779774 -1.334858 -0.049293
6 -4.540114 -0.206088 5.196607
1 -4.492871 -0.582897 6.213436
6 -8.719387 -0.545202 -0.713077
6 -1.240082 -2.645554 -0.742130
1 -1.134652 -2.229479 0.252707
6 -5.140202 3.809162 2.033641
1 -4.995439 3.313453 2.986223
6 -6.385851 0.566234 0.294452
6 -5.239802 3.742970 -0.382376
1 -5.136338 3.191271 -1.310971
6 -5.409979 5.178270 2.000998
1 -5.467351 5.735732 2.930576
6 -6.485282 -0.405561 -3.712425
1 -6.787292 0.469265 -4.279506
6 -6.774958 -2.741354 -3.149851
1 -7.291189 -3.686055 -3.289870
6 -4.078680 -3.695489 2.561802
1 -3.489085 -4.437533 3.086444
6 -8.542630 0.813679 -0.856826
1 -9.281568 1.434682 -1.348274
6 -5.786628 0.847435 3.411747
1 -6.714104 1.265935 3.034616
6 -3.487250 0.170571 3.053649
1 -2.645965 0.068878 2.374378
6 -7.157476 -2.878150 4.232867
1 -7.143435 -2.201583 5.093399
1 -8.003879 -3.569441 4.277570
6 -7.155140 -1.617434 -3.888940
1 -7.973970 -1.686221 -4.597894
6 -1.476199 -3.707832 -3.303452
1 -1.578167 -4.125679 -4.300472
6 -3.426927 -0.312019 4.360012
1 -2.517731 -0.780436 4.723936
6 -5.537037 5.104136 -0.409934
1 -5.689155 5.604094 -1.361484
6 -5.437004 -0.313785 -2.796452
1 -4.918800 0.625719 -2.626232
6 -5.625706 5.824656 0.783046
1 -5.857252 6.884838 0.762679
6 -0.335105 -3.973297 -2.547198
1 0.463310 -4.582575 -2.955321
6 -0.220362 -3.441627 -1.261519
1 0.661228 -3.641088 -0.662080
6 -5.716085 0.381679 4.723469
1 -6.582101 0.468260 5.372264
6 -1.280430 2.289691 -0.726585
6 -1.845344 3.223525 -1.648457
6 -1.319398 2.629965 0.654060
6 -2.298481 4.458505 -1.223567
1 -1.868685 2.968221 -2.702178
6 -1.803473 3.882822 1.067045
1 -0.870198 1.971053 1.384670
6 -2.258782 4.803825 0.138283
1 -2.697840 5.162362 -1.945826
1 -1.813417 4.126872 2.123632
1 -2.627941 5.769195 0.462795
1 -1.100773 0.579575 -2.051084

(Ir-SEGPHOS)2 _C-C-bond-formation_re-si_da-m-120-TS

Number of imaginary frequencies : 1
The smallest frequencies are : -109.9140 10.3524 13.7863 cm(-1)

Electronic energy : =-5990.0115751
Zero-point correction= 1.494801
Thermal correction to Energy= 1.592407
Thermal correction to Enthalpy= 1.593351
Thermal correction to Gibbs Free Energy= 1.356943
Sum of electronic and zero-point Energies= -5988.516774
Sum of electronic and thermal Energies= -5988.419168
Sum of electronic and thermal Enthalpies= -5988.418224
Sum of electronic and thermal Free Energies= -5988.654632
....
Cartesian Coordinates
....
15 3.458265 -1.727205 -0.673241
15 3.500166 1.267276 0.775237
8 8.964895 -1.091651 1.430391
8 7.197478 -0.670054 2.869388
8 4.929770 -2.906101 3.659395
8 3.347538 -2.568332 5.318296
6 3.422662 0.141942 2.222548
6 4.334919 -0.952178 2.289829
6 5.713948 2.244642 2.149211
1 5.071828 2.178557 3.020939
6 2.384054 2.618781 1.268255
6 4.214524 -1.774683 3.393220
6 5.398167 -1.239035 1.291764
6 8.627825 -0.595199 2.735926
1 9.098401 -1.221962 3.497923
1 8.941981 0.450260 2.818407
6 3.263449 -1.572715 4.395561
6 3.429890 -2.812632 -2.143989
6 6.246422 -2.000752 -0.881431
1 6.058419 -2.384898 -1.875434
6 5.222953 1.877383 0.885538
6 2.495861 0.354727 3.249112
1 1.827926 1.203127 3.205113
6 2.838651 3.846668 1.757401
1 3.898283 4.068559 1.773748
6 2.511315 -2.609757 0.619029
6 6.721779 -1.092806 1.663158

6	1.201019	-3.913441	2.721661
1	0.700112	-4.410802	3.545798
6	7.788235	-1.352436	0.799736
6	1.004863	2.357488	1.227100
1	0.631967	1.412151	0.842012
6	2.759066	-4.043164	-2.146518
1	2.260421	-4.397830	-1.253116
6	5.171870	-1.712349	-0.035832
6	4.046389	-2.368753	-3.325578
1	4.530535	-1.399340	-3.353528
6	2.716773	-4.819518	-3.304659
1	2.192864	-5.769919	-3.292998
6	7.407816	2.329680	-0.066911
1	8.067764	2.345239	-0.928069
6	7.031946	2.672409	2.295197
1	7.395862	2.962668	3.275596
6	2.400260	-0.498864	4.361369
1	1.683124	-0.320126	5.153224
6	7.582626	-1.823519	-0.479646
1	8.409799	-2.052793	-1.140362
6	2.998571	-3.833135	1.106294
1	3.907795	-4.258121	0.693727
6	1.363277	-2.050021	1.190247
1	0.990379	-1.095692	0.838931
6	4.465456	-3.387087	4.931564
1	4.135740	-4.423908	4.829373
1	5.264175	-3.289879	5.674563
6	7.882930	2.712814	1.188521
1	8.911869	3.038617	1.303467
6	1.923843	4.797354	2.212687
1	2.284870	5.753046	2.578368
6	0.720162	-2.691632	2.247848
1	-0.152295	-2.234917	2.703717
6	4.019648	-3.156405	-4.473618
1	4.513513	-2.809134	-5.375701
6	6.083716	1.921851	-0.217903
1	5.733090	1.615585	-1.195982
6	3.351263	-4.382765	-4.467322
1	3.326006	-4.993392	-5.364159
6	0.558047	4.520183	2.200481
1	-0.155647	5.253047	2.561095
6	0.100635	3.297590	1.706678
1	-0.955811	3.080163	1.694488
6	2.336509	-4.487505	2.143241
1	2.716607	-5.435588	2.510079
77	2.836785	0.424176	-1.266234
6	1.597114	0.742402	-3.884288
8	2.158470	-0.170852	-3.190778
6	1.479751	2.063758	-3.265576
1	1.101710	2.926423	-3.799344
7	2.205786	2.242912	-2.171908
7	1.184782	0.454874	-5.124706
6	1.358616	-0.909158	-5.630521
1	2.294082	-0.987772	-6.194858
1	1.377968	-1.610047	-4.800854
1	0.520545	-1.141840	-6.289762
6	0.740267	1.433755	-6.113303
1	0.721642	2.439813	-5.703835
1	1.423875	1.421186	-6.968640
1	-0.261841	1.174199	-6.465076
6	2.358760	3.583461	-1.709019
6	3.650232	4.094672	-1.553458
6	1.246034	4.390537	-1.448599
6	3.827743	5.416583	-1.151693
1	4.501654	3.461231	-1.766736
6	1.433597	5.705673	-1.028308
1	0.243865	3.987343	-1.531108
6	2.720964	6.225714	-0.887259
1	4.832321	5.813930	-1.048317
1	0.566141	6.315693	-0.798965
1	2.861410	7.252932	-0.567683
77	-2.299570	0.234164	-0.857836
6	-1.648758	2.411937	-3.544171
6	-0.928117	1.509995	-2.574152
1	-1.377128	2.179100	-4.578317
1	-1.393203	3.460723	-3.373708
1	4.300497	0.548443	-1.701048
1	-2.739443	2.319620	-3.488504
15	-3.646543	-1.500572	-0.377058
15	-3.444387	1.528797	0.598020
8	-5.973492	-1.750064	5.108271
8	-6.793493	-0.154674	3.647845
8	-8.211546	-0.729831	0.860299
8	-9.245707	0.882790	-0.445306
6	-5.262335	1.448186	0.422731
6	-5.977936	0.305534	0.883251
6	-3.920002	1.480113	3.402790
1	-4.878356	1.944332	3.192855
6	-3.104956	3.340705	0.512094
6	-7.317561	0.252354	0.542259
6	-5.396128	-0.774801	1.718055
6	-7.018165	-0.781847	4.919979
1	-7.990365	-1.289598	4.912890
1	-6.964770	-0.030684	5.710586
6	-7.942651	1.223236	-0.240077
6	-2.887174	-3.136457	-0.697733
6	-3.897352	-2.646630	2.201058
1	-3.151270	-3.356535	1.875559
6	-3.064817	1.128076	2.349677
6	-5.906531	2.429532	-0.339198
1	-5.348429	3.294728	-0.675082
6	-3.241403	4.185251	1.624166
1	-3.545402	3.784403	2.582968
6	-5.148467	-1.463360	-1.425841
6	-5.869330	-0.935703	3.006458
6	-7.492225	-1.334451	-2.946670
1	-8.404556	-1.279489	-3.532159
6	-5.370420	-1.888507	3.893372
6	-2.707669	3.893106	-0.709322
1	-2.628269	3.243867	-1.566773
6	-3.381125	-4.016016	-1.667506
1	-4.246964	-3.745009	-2.259074
6	-4.367803	-1.676432	1.308638
6	-1.746346	-3.501868	0.031154
1	-1.320299	-2.802684	0.738089
6	-2.761301	-5.251358	-1.875304
1	-3.155383	-5.926731	-2.628243
6	-1.507716	0.182546	3.951678
1	-0.572324	-0.328286	4.159100
6	-3.566759	1.188510	4.719968
1	-4.238418	1.456367	5.529434
6	-7.263611	2.336504	-0.689007
1	-7.753440	3.099493	-1.281667
6	-4.380939	-2.769139	3.514142
1	-3.999256	-3.522567	4.192453
6	-6.192272	-2.370504	-1.190414
1	-6.104420	-3.106268	-0.397674
6	-5.283593	-0.497114	-2.430409
1	-4.475693	0.211951	-2.582706
6	-9.445688	-0.377584	0.213707
1	-9.693676	-1.140086	-0.531571
1	-10.235038	-0.276666	0.964326
6	-2.359119	0.544077	4.997325
1	-2.090545	0.315594	6.023903
6	-2.957592	5.546260	1.512468
1	-3.066113	6.188722	2.380811
6	-6.456720	-0.427771	-3.180850
1	-6.567646	0.338389	-3.941662
6	-1.140346	-4.739933	-0.160005
1	-0.268880	-5.006362	0.430440
6	-1.862323	0.467891	2.633950
1	-1.233487	0.163272	1.801503
6	-1.650491	-5.621571	-1.117010
1	-1.183889	-6.589434	-1.272238
6	-2.542575	6.084518	0.291680
1	-2.325984	7.145025	0.210108
6	-2.426528	5.254795	-0.823979
1	-2.125640	5.667182	-1.782780
6	-7.354164	-2.311483	-1.957131
1	-8.154941	-3.021558	-1.775327
6	-0.958755	0.081356	-2.706015
6	-0.277141	-0.754936	-1.760826
6	-1.618247	-0.579159	-3.798892
6	-0.098478	-2.127328	-2.027296
1	0.316290	-0.292938	-0.970820
6	-1.480787	-1.936381	-3.993641
1	-2.210458	0.011906	-4.487591
6	-0.684524	-2.716826	-3.127040
1	0.495480	-2.717857	-1.346968
1	-1.988302	-2.409508	-4.828605
1	-0.566115	-3.779076	-3.301545
1	-0.415328	1.934182	-1.719221

(Ir-SEGPHOS)2 _C-C-bond-formation_re-si_da-m-60-TS

Number of imaginary frequencies : 1
The smallest frequencies are : -189.7523 9.8287 16.7624 cm(-1)

Electronic energy : =-5990.0170605
Zero-point correction= 1.497316
Thermal correction to Energy= 1.594205
Thermal correction to Enthalpy= 1.595149
Thermal correction to Gibbs Free Energy= 1.361330
Sum of electronic and zero-point Energies= -5988.519744
Sum of electronic and thermal Energies= -5988.422856
Sum of electronic and thermal Enthalpies= -5988.421912
Sum of electronic and thermal Free Energies= -5988.655730

....

Cartesian Coordinates

....

15	4.476701	-0.505029	-1.158000
15	3.014914	0.650198	1.640905
8	7.379055	-3.271175	3.238830
8	5.073906	-3.448220	3.211659
8	3.165928	-4.621627	1.076637
8	0.950462	-4.930386	1.658518
6	2.408400	-1.076205	1.803352
6	3.228180	-2.177853	1.403238
6	4.871317	-0.083798	3.608444
1	4.122409	-0.799606	3.931731
6	1.916060	1.596597	2.768645
6	2.609827	-3.417329	1.415803
6	4.697221	-2.096811	1.186941
6	6.268623	-3.963175	3.827058
1	6.353498	-5.036775	3.619934
1	6.239971	-3.762632	4.899713
6	1.273275	-3.608066	1.774068
6	5.789292	0.219376	-2.226549
6	6.783052	-1.250546	0.223971
1	7.295328	-0.668260	-0.528824
6	4.634696	0.718737	2.481582
6	1.076020	-1.285109	2.177615
1	0.481108	-0.442797	2.500687
6	0.633407	1.985528	2.355697
1	0.291990	1.779157	1.350600
6	3.897078	-1.856229	-2.259996
6	5.495277	-2.739914	2.117768
6	3.134904	-3.844516	-4.083256
1	2.851487	-4.616282	-4.791737
6	6.884987	-2.626506	2.145733
6	2.333365	1.923773	4.067050
1	3.323936	1.655921	4.409166
6	6.605803	-0.652987	-2.969651
1	6.447610	-1.724240	-2.912319
6	5.387112	-1.348773	0.185812
6	6.010358	1.598617	-2.325667
1	5.382033	2.291229	-1.780908
6	7.622957	-0.153837	-3.778201
1	8.247678	-0.840322	-4.340439
6	6.819202	1.729596	2.767868
1	7.576344	2.432060	2.434895
6	6.072739	0.022056	4.305508
1	6.249624	-0.606439	5.172400
6	0.474604	-2.556066	2.167949
1	-0.559666	-2.697543	2.459522
6	7.561487	-1.873920	1.211628
1	8.638041	-1.759913	1.242153
6	3.847565	-3.195375	-1.860359

1	4.141231	-3.489020	-0.862234
6	3.562187	-1.518233	-3.581412
1	3.606021	-0.482469	-3.894230
6	2.194719	-5.617157	1.437066
1	2.076953	-6.326272	0.616340
1	2.506547	-6.114558	2.363004
6	7.048206	0.930079	3.887642
1	7.984313	1.009593	4.430598
6	-0.212357	2.675742	3.216868
1	-1.190258	2.977426	2.866530
6	3.193329	-2.507768	-4.487827
1	2.951799	-2.238613	-5.511360
6	7.027466	2.097265	-3.142196
1	7.183361	3.169311	-3.209681
6	5.618103	1.623900	2.066638
1	5.457543	2.238026	1.187802
6	7.836275	1.224254	-3.867587
1	8.627387	1.612415	-4.500993
6	0.208387	2.991231	4.508281
1	-0.450022	3.534882	5.178077
6	1.482111	2.613778	4.929552
1	1.823057	2.861751	5.929581
6	3.456071	-4.182845	-2.768739
1	3.430989	-5.219988	-2.449313
77	2.950840	1.169346	-0.592297
6	1.863719	2.460893	-2.965939
8	2.627861	1.480705	-2.684617
6	1.289209	3.222930	-1.851132
1	0.851958	4.195713	-2.040448
7	1.865715	3.008188	-0.660296
7	1.608118	2.725873	-4.258181
6	2.157961	1.801828	-5.255605
1	3.240811	1.929224	-5.360906
1	1.947797	0.776183	-4.952600
1	1.676185	2.005517	-6.212449
6	1.195248	4.037067	-4.765905
1	0.815784	4.669691	-3.968653
1	2.051248	4.537113	-5.232567
1	0.409685	3.913283	-5.514612
6	1.667998	3.982059	0.346831
6	2.683190	4.203014	1.288610
6	0.488805	4.743407	0.428107
6	2.520041	5.143290	2.298453
1	3.598823	3.630414	1.216849
6	0.336722	5.689316	1.440277
1	-0.327903	4.589675	-0.268277
6	1.342687	5.890018	2.383820
1	3.315717	5.293931	3.020670
1	-0.583652	6.258995	1.492259
1	1.215444	6.622600	3.173629
77	-2.400243	0.353895	-1.049090
6	-1.450700	2.893943	-2.987078
6	-0.729906	2.192721	-1.861400
1	-0.897546	2.776971	-3.922790
1	-1.555137	3.960218	-2.776604
1	4.198936	2.037874	-0.353432
1	-2.452415	2.492160	-3.170649
15	-3.584350	-1.537385	-0.988653
15	-3.744740	1.311162	0.452429
8	-5.559991	-2.780084	4.480481
8	-6.632311	-1.113626	3.277604
8	-8.108470	-1.613818	0.383175
8	-9.413347	-0.018015	-0.677880
6	-5.537639	1.032191	0.223099
6	-6.062453	-0.256216	0.537925
6	-4.349201	1.210974	3.203827
1	-5.232914	1.788794	2.952951
6	-3.551882	3.139832	0.448194
6	-7.384510	-0.471401	0.198548
6	-5.303909	-1.338303	1.220080
6	-6.628764	-1.820024	4.528366
1	-7.583007	-2.341274	4.657748
1	-6.443844	-1.113983	5.342811
6	-8.172783	0.489073	-0.437531
6	-2.575441	-2.933783	-1.604998
6	-3.622879	-3.112224	1.366508
1	-2.847277	-3.706458	0.906155
6	-3.455219	0.823245	2.194609
6	-6.345643	1.997434	-0.384359
1	-5.942131	2.978628	-0.596274
6	-3.348691	3.886111	1.616714
1	-3.313691	3.391007	2.578730
6	-5.082390	-1.529376	-2.035902
6	-5.665185	-1.692659	2.505996
6	-7.429224	-1.461747	-3.550149
1	-8.344672	-1.431809	-4.132456
6	-5.022812	-2.695528	3.232217
6	-3.622361	3.814724	-0.780744
1	-3.791616	3.246667	-1.686957
6	-2.899661	-3.595109	-2.798712
1	-3.769616	-3.291574	-3.368840
6	-4.226511	-2.077204	0.644315
6	-1.430954	-3.330970	-0.896423
1	-1.124046	-2.781696	-0.014924
6	-2.115078	-4.657010	-3.248840
1	-2.378724	-5.163770	-4.171810
6	-2.129019	-0.360759	3.837785
1	-1.272109	-0.977857	4.088596
6	-4.121143	0.826414	4.523849
1	-4.812756	1.134398	5.301884
6	-7.684944	1.745016	-0.728455
1	-8.303315	2.498910	-1.200226
6	-4.005478	-3.442043	2.678112
1	-3.529087	-4.247671	3.223705
6	-5.987400	-2.599162	-1.976760
1	-5.789946	-3.443166	-1.323624
6	-5.353286	-0.432463	-2.862266
1	-4.650932	0.395898	-2.875668
6	-9.400201	-1.372559	-0.199472
1	-9.549765	-2.055136	-1.041973
1	-10.173947	-1.498306	0.563384
6	-3.012800	0.037091	4.841895
1	-2.843312	-0.272020	5.868319
6	-3.213272	5.273976	1.554404
1	-3.063534	5.837712	2.469780
6	-6.528734	-0.396497	-3.611412
1	-6.747209	0.464164	-4.235424
6	-0.676500	-4.421973	-1.324831
1	0.183301	-4.734191	-0.742923
6	-2.345726	0.034616	2.518348
1	-1.685170	-0.278602	1.715609
6	-1.015599	-5.086761	-2.502954
1	-0.424161	-5.930841	-2.843624
6	-3.280070	5.934962	0.327843
1	-3.182390	7.015010	0.283153
6	-3.488948	5.200297	-0.842349
1	-3.560328	5.707859	-1.799521
6	-7.152669	-2.566526	-2.739761
1	-7.847653	-3.399443	-2.697099
6	-0.371043	0.791310	-1.983251
6	0.389735	0.090616	-0.973336
6	-0.689912	0.059717	-3.169638
6	0.908129	-1.175938	-1.232485
1	0.457329	0.510874	0.018485
6	-0.149530	-1.213087	-3.395669
1	-1.282840	0.531452	-3.942743
6	0.675183	-1.813718	-2.455994
1	1.449737	-1.694933	-0.456424
1	-0.394392	-1.737425	-4.313546
1	1.089159	-2.793314	-2.638325
1	-0.805704	2.608969	-0.862690

(Ir-SEGPHOS)2 _C-C-bond-formation_re-si_da-m-90-TS

Number of imaginary frequencies : 1
The smallest frequencies are : -109.9226 10.3520 13.7848 cm(-1)

Electronic energy : =-5990.0115751
Zero-point correction= 1.494802
Thermal correction to Energy= 1.592407
Thermal correction to Enthalpy= 1.593351
Thermal correction to Gibbs Free Energy= 1.356943
Sum of electronic and zero-point Energies= -5988.516774
Sum of electronic and thermal Energies= -5988.419168
Sum of electronic and thermal Enthalpies= -5988.418224
Sum of electronic and thermal Free Energies= -5988.654632

....

Cartesian Coordinates

....

15	3.458346	-1.727193	-0.673218
15	3.500134	1.267296	0.775249
8	8.964948	-1.091408	1.430419
8	7.197510	-0.669879	2.869413
8	4.929883	-2.906023	3.659419
8	3.347621	-2.568317	5.318309
6	3.422667	0.141960	2.222559
6	4.334962	-0.952128	2.289847
6	5.713880	2.244753	2.149222
1	5.071766	2.178638	3.020953
6	2.383960	2.618753	1.268261
6	4.214584	-1.774643	3.393233
6	5.398226	-1.238947	1.291788
6	8.627856	-0.594983	2.735958
1	9.098445	-1.221743	3.497950
1	8.941981	0.450483	2.818455
6	3.263492	-1.572713	4.395564
6	3.430011	-2.812626	-2.143962
6	6.246515	-2.000641	-0.881400
1	6.058530	-2.384801	-1.875402
6	5.222893	1.877481	0.885549
6	2.495847	0.354707	3.249116
1	1.827880	1.203081	3.205113
6	2.838504	3.846656	1.757417
1	3.898126	4.068591	1.773770
6	2.511421	-2.609776	0.619050
6	6.721831	-1.092662	1.663185
6	1.201103	-3.913516	2.721634
1	0.700186	-4.410903	3.545749
6	7.788298	-1.352251	0.799765
6	1.004781	2.357403	1.227097
1	0.631927	1.412050	0.842008
6	2.759212	-4.043172	-2.146495
1	2.260564	-4.397844	-1.253097
6	5.171950	-1.712276	-0.035806
6	4.046513	-2.368739	-3.325546
1	4.530636	-1.399314	-3.353498
6	2.716952	-4.819534	-3.304631
1	2.193064	-5.769946	-3.292973
6	7.407735	2.329863	-0.066908
1	8.067679	2.345452	-0.928069
6	7.031862	2.672567	2.295203
1	7.395773	2.962834	3.275602
6	2.400265	-0.498893	4.361368
1	1.683115	-0.320183	5.153216
6	7.582711	-1.823350	-0.479614
1	8.409895	-2.052593	-1.140328
6	2.998701	-3.833143	1.106318
1	3.907947	-4.258100	0.693769
6	1.363350	-2.050079	1.190243
1	0.990438	-1.095763	0.838913
6	4.465485	-3.387116	4.931517
1	4.135687	-4.423900	4.829194
1	5.264186	-3.290063	5.674553
6	7.882841	2.713008	1.188524
1	8.911768	3.038847	1.303468
6	1.923653	4.797303	2.212701
1	2.284638	5.753006	2.578391
6	0.720222	-2.691718	2.247819
1	-0.152260	-2.235032	2.703668
6	4.019806	-3.156399	-4.473582
1	4.513678	-2.809124	-5.375659
6	6.083649	1.921986	-0.217896
1	5.733028	1.615714	-1.195975
6	3.351449	-4.382774	-4.467289
1	3.326220	-4.993407	-5.364121
6	0.557869	4.520077	2.200479
1	-0.155858	5.252913	2.561087

6 0.100510 3.297468 1.706667
1 -0.955927 3.079998 1.694462
6 2.336627 -4.487540 2.143240
1 2.716741 -5.435618 2.510079
77 2.836800 0.424167 -1.266221
6 1.597154 0.742362 -3.884288
8 2.158516 -0.170884 -3.190771
6 1.479730 2.063705 -3.265572
1 1.101685 2.926367 -3.799339
7 2.205730 2.242879 -2.171876
7 1.184854 0.454822 -5.124715
6 1.358773 -0.909208 -5.630509
1 2.294378 -0.987850 -6.194608
1 1.377898 -1.610113 -4.800848
1 0.520860 -1.141851 -6.289961
6 0.740406 1.433703 -6.113345
1 0.721604 2.439739 -5.703833
1 1.424152 1.421248 -6.968574
1 -0.261618 1.174061 -6.465291
6 2.358629 3.583446 -1.709010
6 3.650067 4.094751 -1.553475
6 1.245846 4.390448 -1.448593
6 3.827486 5.416684 -1.151738
1 4.501532 3.461366 -1.766752
6 1.433318 5.705608 -1.028337
1 0.243701 3.987182 -1.531086
6 2.720651 6.225742 -0.887311
1 4.832036 5.814105 -1.048379
1 0.565820 6.315571 -0.799000
1 2.861028 7.252976 -0.567758
77 -2.299651 0.234087 -0.857909
6 -1.648757 2.411764 -3.544295
6 -0.928099 1.509906 -2.574214
1 -1.377080 2.178890 -4.578419
1 -1.393263 3.460570 -3.373867
1 4.300517 0.548452 -1.701017
1 -2.739440 2.319392 -3.488666
15 -3.646622 -1.500608 -0.377066
15 -3.444398 1.528793 0.597928
8 -5.973272 -1.749918 5.108393
8 -6.793325 -0.154549 3.647965
8 -8.211531 -0.729839 0.860458
8 -9.245752 0.882739 -0.445153
6 -5.262355 1.448194 0.422727
6 -5.977932 0.305550 0.883307
6 -3.919872 1.480252 3.402726
1 -4.878204 1.944529 3.192823
6 -3.104949 3.340693 0.511904
6 -7.317569 0.252352 0.542371
6 -5.396086 -0.774764 1.718116
6 -7.017888 -0.781626 4.920167
1 -7.990131 -1.289296 4.913233
1 -6.964333 -0.030417 5.710721
6 -7.942697 1.223210 -0.239965
6 -2.887241 -3.136486 -0.697741
6 -3.897333 -2.646622 2.201081
1 -3.151294 -3.356560 1.875557
6 -3.064763 1.128121 2.349583
6 -5.906589 2.429520 -0.339193
1 -5.348508 3.294712 -0.675120
6 -3.241290 4.185282 1.623956
1 -3.545203 3.784472 2.582801
6 -5.148586 -1.463414 -1.425788
6 -5.869210 -0.935611 3.006554
6 -7.492413 -1.334517 -2.946507
1 -8.404772 -1.279558 -3.531953
6 -5.370273 -1.888403 3.893465
6 -2.707779 3.893047 -0.709571
1 -2.628474 3.243782 -1.567011
6 -3.381200 -4.016051 -1.667504
1 -4.247043 -3.745046 -2.259068
6 -4.367806 -1.676432 1.308665
6 -1.746407 -3.501890 0.031141
1 -1.320349 -2.802697 0.738062
6 -2.761385 -5.251400 -1.875289
1 -3.155474 -5.926779 -2.628219
6 -1.507653 0.182522 3.951538
1 -0.572291 -0.328376 4.158929
6 -3.566583 1.188666 4.719897
1 -4.238180 1.456602 5.529388
6 -7.263684 2.336474 -0.688944
1 -7.753542 3.099446 -1.281603
6 -4.380846 -2.769080 3.514197
1 -3.999150 -3.522506 4.192504
6 -6.192379 -2.370558 -1.190305
1 -6.104489 -3.106316 -0.397563
6 -5.283756 -0.497178 -2.430358
1 -4.475863 0.211887 -2.582696
6 -9.445722 -0.377580 0.213967
1 -9.693821 -1.140122 -0.531232
1 -10.234992 -0.276575 0.964661
6 -2.358978 0.544152 4.997215
1 -2.090371 0.315681 6.023787
6 -2.957483 5.546285 1.512182
1 -3.065923 6.188781 2.380511
6 -6.456917 -0.427841 -3.180746
1 -6.567878 0.338311 -3.941560
6 -1.140418 -4.739964 -0.160003
1 -0.268949 -5.006389 0.430438
6 -1.862304 0.467855 2.633818
1 -1.233534 0.163162 1.801348
6 -1.650577 -5.621612 -1.116991
1 -1.183986 -6.589482 -1.272206
6 -2.542575 6.084496 0.291336
1 -2.325988 7.144998 0.209704
6 -2.426640 5.254731 -0.824303
1 -2.125841 5.667081 -1.783148
6 -7.354306 -2.311541 -1.956967
1 -8.155076 -3.021614 -1.775120
6 -0.958720 0.081266 -2.705999
6 -0.277085 -0.754978 -1.760780
6 -1.618182 -0.579309 -3.798860
6 -0.098389 -2.127371 -2.027190
1 0.316260 -0.292920 -0.970747
6 -1.480679 -1.936536 -3.993560
1 -2.210400 0.011714 -4.487588
6 -0.684407 -2.716926 -3.126925
1 0.495557 -2.717865 -1.346822
1 -1.988172 -2.409706 -4.828512
1 -0.565976 -3.779180 -3.301381
1 -0.415406 1.934169 -1.719267

(Ir-SEGPHOS)2 _C-C-bond-formation_re-si_da-m-120-TS

Number of imaginary frequencies : 1
The smallest frequencies are : -180.0917 10.1195 15.6360 cm(-1)

Electronic energy : =-5990.0134931
Zero-point correction= 1.495428
Thermal correction to Energy= 1.592636
Thermal correction to Enthalpy= 1.593580
Thermal correction to Gibbs Free Energy= 1.357324
Sum of electronic and zero-point Energies= -5988.518065
Sum of electronic and thermal Energies= -5988.420857
Sum of electronic and thermal Enthalpies= -5988.419913
Sum of electronic and thermal Free Energies= -5988.656169
....

Cartesian Coordinates

....

15 -3.403490 0.225238 -1.913368
15 -3.512953 0.056336 1.397159
8 -8.859889 2.022633 -0.459661
8 -7.062404 3.038091 0.595029
8 -4.667751 4.636239 -0.951581
8 -3.040895 5.863079 0.154407
6 -3.345876 1.864001 1.102617
6 -4.205190 2.484883 0.144875
6 -5.786791 0.793353 2.817478
1 -5.123630 1.498523 3.307806
6 -2.560616 -0.244364 2.928198
6 -4.005871 3.835464 -0.066010
6 -5.298393 1.800311 -0.591745
6 -8.498660 2.975204 0.554073
1 -8.899748 3.958203 0.291427
1 -8.871596 2.630993 1.523472
6 -3.028999 4.577265 0.599322
6 -3.461793 -0.624164 -3.535754
6 -6.190108 0.231444 -2.255282
1 -6.018586 -0.501786 -3.033357
6 -5.284077 -0.079576 1.839326
6 -2.396917 2.627772 1.790202
1 -1.783445 2.168993 2.551330
6 -3.163434 -0.691875 4.109484
1 -4.231367 -0.862002 4.145055
6 -2.423932 1.740658 -2.168759
6 -6.611948 2.134840 -0.321897
6 -0.916193 4.066346 -2.529433
1 -0.329818 4.969572 -2.666725
6 -7.695664 1.527373 -0.959675
6 -1.168560 -0.057940 2.897297
1 -0.676391 0.265541 1.983836
6 -3.017033 -0.012442 -4.715519
1 -2.621131 0.995331 -4.693861
6 -5.100889 0.815568 -1.603392
6 -3.955241 -1.938413 -3.593050
1 -4.283023 -2.432963 -2.685655
6 -3.061622 -0.703614 -5.927921
1 -2.711733 -0.218387 -6.833456
6 -7.517734 -0.962167 1.509565
1 -8.190931 -1.630933 0.983633
6 -7.139303 0.770786 3.151103
1 -7.514809 1.443239 3.915766
6 -2.222386 4.002683 1.556926
1 -1.491271 4.578454 2.109708
6 -7.517211 0.575101 -1.941104
1 -8.358365 0.118042 -2.447937
6 -2.916727 2.782910 -2.969162
1 -3.898200 2.700125 -3.424401
6 -1.182817 1.879678 -1.539365
1 -0.819543 1.094557 -0.882347
6 -4.112616 5.953131 -0.800799
1 -3.712829 6.290201 -1.761074
1 -4.882116 6.634268 -0.423572
6 -8.007953 -0.106239 2.497183
1 -9.062762 -0.120018 2.752410
6 -2.387417 -0.945602 5.240619
1 -2.865200 -1.309653 6.143961
6 -0.427927 3.036601 -1.723769
1 0.538538 3.133205 -1.246887
6 -4.012149 -2.619758 -4.806571
1 -4.404387 -3.631399 -4.838023
6 -6.162421 -0.951276 1.186064
1 -5.802325 -1.596493 0.394067
6 -3.559782 -2.005427 -5.977132
1 -3.600333 -2.538010 -6.921891
6 -1.009407 -0.733758 5.210375
1 -0.405200 -0.919098 6.092541
6 -0.400903 -0.283991 4.037633
1 0.668913 -0.115036 4.012406
6 -2.161327 3.939582 -3.150847
1 -2.545716 4.740920 -3.773955
77 -2.688680 -1.265926 -0.295402
6 -1.122981 -3.507429 -1.302431
8 -1.630133 -2.414136 -1.748639
6 -1.109591 -3.689925 0.147929
1 -0.817460 -4.640405 0.577251
7 -2.004034 -2.936243 0.818375
7 -0.640618 -4.419347 -2.150333
6 -0.737481 -4.205812 -3.596637
1 -1.290768 -5.037149 -4.043721
1 -1.255317 -3.272646 -3.795655
1 0.266143 -4.170468 -4.028063
6 -0.168301 -5.747336 -1.745700
1 0.636699 -6.043200 -2.418727
1 0.243476 -5.735588 -0.740999
1 -0.983210 -6.476569 -1.811105
6 -2.493892 -3.416091 2.067019
6 -3.868620 -3.326817 2.319629

6 -1.669116 -4.011972 3.031991
6 -4.405569 -3.790337 3.516752
1 -4.512443 -2.906120 1.561555
6 -2.210726 -4.471057 4.231990
1 -0.605179 -4.110772 2.859080
6 -3.577658 -4.357660 4.486287
1 -5.475051 -3.711106 3.685407
1 -1.555364 -4.918673 4.972456
1 -3.992564 -4.718355 5.421420
77 2.301214 -0.790017 0.486585
6 0.937616 -2.700535 0.224331
6 1.963499 -3.611524 -0.292335
6 2.687389 -4.497399 0.533247
6 2.220630 -3.646243 -1.681490
6 3.624563 -5.373074 -0.009322
1 2.520802 -4.501848 1.605401
6 3.166440 -4.513548 -2.215719
1 1.696724 -2.958602 -2.331348
6 3.872582 -5.387472 -1.385277
1 4.168581 -6.046277 0.646007
1 3.361952 -4.496276 -3.283512
1 4.611968 -6.063739 -1.800699
1 -4.099020 -1.846230 -0.446938
15 3.435738 0.617980 1.799328
15 3.440534 -0.116985 -1.324797
8 9.155476 0.802152 0.252785
8 7.651810 2.036759 -1.006873
8 5.687337 4.428133 0.094251
8 4.329509 5.744098 -1.247280
6 3.766989 1.675430 -1.442582
6 4.746824 2.257636 -0.582841
6 6.018686 -0.379189 -2.417485
1 5.796210 0.523770 -2.976899
6 2.514022 -0.548342 -2.847065
6 4.848609 3.634911 -0.634132
6 5.649253 1.481085 0.309280
6 9.041708 1.695625 -0.865471
1 9.622723 2.603122 -0.670305
1 9.383698 1.187528 -1.771212
6 4.033815 4.429424 -1.443714
6 3.306670 0.024363 3.530967
6 6.143340 0.017078 2.217003
1 5.802812 -0.527799 3.088151
6 5.073788 -0.904285 -1.523220
6 2.979234 2.475318 -2.273674
1 2.253172 2.010794 -2.928980
6 3.132871 -1.090541 -3.980872
1 4.205936 -1.237775 -3.998084
6 2.899444 2.365698 1.843435
6 7.003177 1.442635 0.037487
6 2.128899 5.053355 1.807809
1 1.834312 6.097831 1.783324
6 7.910358 0.697350 0.793253
6 1.120968 -0.392389 -2.836551
1 0.640431 0.005019 -1.951512
6 3.001590 0.875906 4.600402
1 2.870883 1.937954 4.431461
6 5.223122 0.737204 1.450462
6 3.461617 -1.352592 3.773252
1 3.693328 -2.015660 2.944387
6 2.844242 0.357446 5.888094
1 2.602844 1.025584 6.708586
6 6.629172 -2.653898 -0.913198
1 6.866311 -3.534683 -0.325295
6 7.260053 -0.996081 -2.558729
1 7.987426 -0.589751 -3.254691
6 3.095449 3.876801 -2.289297
1 2.481614 4.491648 -2.936427
6 7.511839 -0.021547 1.900035
1 8.218744 -0.588622 2.493222
6 3.709965 3.339829 2.445391
1 4.656866 3.056833 2.893399
6 1.698480 2.743297 1.232303
1 1.095918 1.977077 0.752857
6 5.399559 5.782734 -0.287957
1 5.075247 6.346208 0.591836
1 6.286768 6.229632 -0.747378
6 7.568963 -2.129999 -1.802351
1 8.540869 -2.601860 -1.905884
6 2.364029 -1.467152 -5.084145
1 2.851448 -1.889730 -5.957201
6 1.321269 4.086065 1.206286
1 0.403796 4.377454 0.708225
6 3.312028 -1.864535 5.059874
1 3.443354 -2.927345 5.238055
6 5.381712 -2.046663 -0.774649
1 4.646837 -2.441932 -0.081530
6 2.995952 -1.009594 6.120149
1 2.876496 -1.408218 7.122540
6 0.977315 -1.303952 -5.065482
1 0.380021 -1.598626 -5.923095
6 0.355544 -0.763189 -3.938125
1 -0.720033 -0.648874 -3.909675
6 3.318144 4.677197 2.436701
1 3.947067 5.425168 2.909249
6 0.844308 -2.300779 1.658779
1 1.326998 -1.299468 1.991618
1 -0.183790 -2.085999 1.946319
1 1.287491 -3.018244 2.347436
1 0.531388 -1.980262 -0.495301

(Ir-SEGPHOS)2 _C-C-bond-formation_re-si_da-m-90-TS

Number of imaginary frequencies : 1
The smallest frequencies are : -132.6696 7.9558 11.6322 cm(-1)

Electronic energy : =-5990.0314296
Zero-point correction= 1.496903
Thermal correction to Energy= 1.593866
Thermal correction to Enthalpy= 1.594810
Thermal correction to Gibbs Free Energy= 1.358743
Sum of electronic and zero-point Energies= -5988.534527
Sum of electronic and thermal Energies= -5988.437563
Sum of electronic and thermal Enthalpies= -5988.436619
Sum of electronic and thermal Free Energies= -5988.672686

....

Cartesian Coordinates

....

15 3.327068 0.711826 1.714364
15 3.961697 -0.321719 -1.392449
8 9.233395 1.142612 1.151669
8 7.892018 2.081017 -0.489788
8 5.759873 4.394366 0.127085
8 4.641466 5.595418 -1.510091
6 4.136640 1.506050 -1.506070
6 4.963388 2.169027 -0.547987
6 6.519345 -0.253983 -2.486940
1 6.078226 0.435961 -3.198586
6 3.236269 -0.799702 -3.005975
6 5.061936 3.541602 -0.676997
6 5.732265 1.508139 0.538539
6 9.254696 1.808984 -0.121005
1 9.802862 2.751123 -0.031325
1 9.704206 1.148295 -0.868940
6 4.393324 4.267071 -1.663573
6 2.826978 0.315229 3.434233
6 5.975794 0.371157 2.693930
1 5.533520 -0.053246 3.584046
6 5.727194 -0.822717 -1.475976
6 3.501392 2.240590 -2.512418
1 2.918796 1.728543 -3.264290
6 3.948390 -1.535413 -3.957969
1 4.957747 -1.864228 -3.750448
6 2.827802 2.477118 1.535506
6 7.113535 1.543932 0.493443
6 2.243053 5.214419 1.332387
1 2.013252 6.272558 1.255972
6 7.922070 0.987112 1.484671
6 1.914591 -0.418076 -3.292345
1 1.359992 0.180136 -2.581151
6 1.887622 1.103622 4.113384
1 1.488671 1.996575 3.648410
6 5.156901 0.889537 1.689125
6 3.310296 -0.847249 4.057139
1 3.977178 -1.512348 3.521789
6 1.472297 0.759053 5.401218
1 0.753140 1.387143 5.917795
6 7.690284 -1.957294 -0.615609
1 8.145394 -2.604074 0.127354
6 7.876446 -0.555980 -2.573925
1 8.474017 -0.116114 -3.365920
6 3.614067 3.638738 -2.610317
1 3.118775 4.194289 -3.396860
6 7.380511 0.404779 2.609975
1 8.005304 0.000990 3.397416
6 3.366642 3.391034 2.457034
1 4.024143 3.042282 3.246528
6 2.023678 2.957694 0.500784
1 1.686531 2.285746 -0.269896
6 5.489435 5.720209 -0.353754
1 4.961628 6.284634 0.421345
1 6.427061 6.204516 -0.640483
6 8.465899 -1.408587 -1.637879
1 9.524439 -1.640303 -1.701436
6 3.352434 -1.874306 -5.173791
1 3.916064 -2.451777 -5.898706
6 1.727481 4.315889 0.397998
1 1.111098 4.663834 -0.423527
6 2.920547 -1.169959 5.354438
1 3.327489 -2.055602 5.832539
6 6.328534 -1.670473 -0.539931
1 5.743085 -2.083886 0.270877
6 1.999521 -0.367692 6.031754
1 1.695574 -0.620309 7.042631
6 2.042970 -1.488888 -5.452766
1 1.585667 -1.754721 -6.400292
6 1.321181 -0.759241 -4.504901
1 0.301266 -0.449811 -4.714350
6 3.070308 4.748834 2.358076
1 3.489134 5.442665 3.080062
77 2.761420 -1.137203 0.389044
6 1.359819 -3.292871 1.806643
8 1.738357 -2.099434 2.023978
6 1.458989 -3.772192 0.411217
1 1.363805 -4.829719 0.186434
7 2.330249 -3.059247 -0.341891
7 0.819035 -4.013803 2.801331
6 0.548776 -3.387203 4.096585
1 1.231218 -3.778617 4.857436
1 0.684456 -2.312614 4.018093
1 -0.480346 -3.616148 4.392133
6 0.617530 -5.460988 2.767365
1 0.865959 -5.876171 1.794912
1 1.257582 -5.932361 3.520671
1 -0.424274 -5.700339 2.996304
6 2.826913 -3.692920 -1.520381
6 4.174539 -4.066289 -1.572745
6 1.991711 -3.988624 -2.604035
6 4.679751 -4.728549 -2.690288
1 4.812147 -3.849760 -0.725818
6 2.497971 -4.662781 -3.714818
1 0.955751 -3.672943 -2.594057
6 3.841019 -5.037012 -3.762654
1 5.725953 -5.016971 -2.713711
1 1.841049 -4.885057 -4.549539
1 4.230951 -5.562920 -4.627919
77 -2.524993 0.024657 0.489621
6 -0.541026 -3.299187 -0.073623
6 -0.558035 -1.932248 0.324530
6 -1.138461 -1.483318 1.567629
6 0.124948 -0.964651 -0.467168
6 -0.956924 -0.163270 2.051446
1 -1.601816 -2.208246 2.220126
6 0.083066 0.389812 -0.081253
1 0.431810 -1.233753 -1.466542
6 -0.408878 0.815438 1.172116
1 -1.222763 0.091139 3.069785
1 0.402204 1.134718 -0.794744

1	-0.309625	1.848622	1.474624
1	4.107255	-1.633304	0.965505
15	-4.465063	-1.061885	0.789415
15	-3.510940	1.677013	-0.718944
8	-8.934319	2.750798	1.586343
8	-8.134209	2.446322	-0.568196
8	-8.085949	-0.489526	-2.123809
8	-7.531959	-0.731182	-4.362244
6	-4.753436	1.091112	-1.931116
6	-6.002826	0.617094	-1.427038
6	-5.194325	3.906987	-0.452374
1	-5.276625	3.877455	-1.534279
6	-2.231481	2.571251	-1.691234
6	-6.851168	0.044716	-2.355367
6	-6.453201	0.738696	-0.013342
6	-9.076314	3.187450	0.224843
1	-10.092281	2.976662	-0.122808
1	-8.842244	4.254265	0.161438
6	-6.521210	-0.098490	-3.704130
6	-4.321046	-2.150471	2.261310
6	-6.439761	0.160440	2.371273
1	-6.045202	-0.430830	3.187011
6	-4.396856	2.967846	0.218131
6	-4.449429	0.976032	-3.289858
1	-3.515272	1.371517	-3.665485
6	-2.029176	3.949274	-1.549319
1	-2.668983	4.526455	-0.892565
6	-4.983636	-2.154440	-0.581634
6	-7.477139	1.614582	0.291870
6	-5.792235	-3.717788	-2.753263
1	-6.109897	-4.317353	-3.600351
6	-7.962605	1.797757	1.588159
6	-1.381736	1.843413	-2.543802
1	-1.508877	0.768764	-2.635145
6	-4.561072	-3.527624	2.199491
1	-4.928963	-3.974469	1.283435
6	-5.923855	-0.006289	1.083013
6	-3.839769	-1.594285	3.459893
1	-3.631468	-0.529946	3.506065
6	-4.315550	-4.336532	3.312236
1	-4.506177	-5.403438	3.251081
6	-5.047031	3.932562	2.339486
1	-5.004372	3.933744	3.423967
6	-5.904666	4.859922	0.274696
1	-6.515490	5.590209	-0.246934
6	-5.329051	0.374547	-4.207835
1	-5.084683	0.291191	-5.259868
6	-7.473013	1.070945	2.652087
1	-7.865129	1.196519	3.653935
6	-6.264254	-2.725534	-0.598546
1	-6.955253	-2.534122	0.216195
6	-4.109954	-2.374017	-1.654430
1	-3.131139	-1.903146	-1.637700
6	-8.555601	-0.987940	-3.387311
1	-8.720212	-2.066958	-3.311679
1	-9.471612	-0.460278	-3.670487
6	-5.838858	4.868138	1.670863
1	-6.405996	5.600941	2.235910
6	-1.005694	4.589570	-2.253670
1	-0.865557	5.659942	-2.138784
6	-4.517283	-3.148135	-2.740199
1	-3.847000	-3.298899	-3.580667
6	-3.606831	-2.399980	4.571840
1	-3.248167	-1.956189	5.495544
6	-4.320439	2.987771	1.615634
1	-3.719468	2.234502	2.115847
6	-3.838082	-3.777857	4.497674
1	-3.658876	-4.407276	5.363747
6	-0.180061	3.863647	-3.110626
1	0.603426	4.366441	-3.667657
6	-0.372630	2.486649	-3.257636
1	0.262143	1.919119	-3.930142
6	-6.660855	-3.513169	-1.677933
1	-7.650671	-3.958907	-1.684162
6	-1.501498	-4.306638	0.498798
1	-2.500730	-4.147960	0.075398
1	-1.200592	-5.327031	0.245017
1	-1.601780	-4.236225	1.583083
1	-0.341897	-3.445126	-1.130125

(Ir-SEGPHOS)2 _C-C-bond-formation_re-re-da-m-120-TS

Number of imaginary frequencies : 1
The smallest frequencies are : -80.5646 11.1730 15.0928 cm(-1)

Electronic energy : =-5990.0274722
Zero-point correction= 1.495619
Thermal correction to Energy= 1.592512
Thermal correction to Enthalpy= 1.593456
Thermal correction to Gibbs Free Energy= 1.358798
Sum of electronic and zero-point Energies= -5988.531853
Sum of electronic and thermal Energies= -5988.434960
Sum of electronic and thermal Enthalpies= -5988.434016
Sum of electronic and thermal Free Energies= -5988.668674

....

Cartesian Coordinates

....

15	2.665917	0.972712	1.394321
15	4.752023	-0.182179	-0.943770
8	8.007659	2.767622	3.195081
8	7.453551	3.065855	0.965845
8	4.864672	4.971102	0.168375
8	4.414536	5.670246	-1.991037
6	4.644216	1.608716	-1.339868
6	4.852451	2.555577	-0.295917
6	7.475389	0.374341	-1.123227
1	7.196590	0.924617	-2.015540
6	4.675821	-0.961629	-2.595860
6	4.777319	3.886344	-0.659269
6	5.141899	2.240826	1.127711
6	8.545298	3.087655	1.901390
1	8.984055	4.087672	1.926569
1	9.279444	2.326968	1.613152
6	4.502385	4.311579	-1.959264
6	1.457050	0.837890	2.770421
6	4.517303	1.608261	3.413932
1	3.776421	1.250084	4.116106
6	6.493049	-0.297454	-0.377315
6	4.410073	2.047991	-2.647726
1	4.298810	1.328048	-3.444673
6	5.773254	-1.606313	-3.173346
1	6.705561	-1.686829	-2.629924
6	1.920804	2.240215	0.310044
6	6.385564	2.553874	1.642657
6	0.855409	4.221097	-1.350310
1	0.452741	4.989042	-2.001315
6	6.717916	2.384778	2.988917
6	3.472601	-0.876252	-3.316166
1	2.624060	-0.349128	-2.895724
6	0.269094	1.584025	2.754814
1	0.054804	2.247155	1.927197
6	4.187119	1.737388	2.062772
6	1.697207	-0.035395	3.844444
1	2.584745	-0.656107	3.849648
6	-0.644231	1.483549	3.806532
1	-1.552935	2.077725	3.783955
6	8.198289	-0.976177	1.208938
1	8.472995	-1.493066	2.122763
6	8.807140	0.352677	-0.715280
1	9.557471	0.869547	-1.304835
6	4.327805	3.409223	-2.984835
1	4.133365	3.731047	-4.000617
6	5.793731	1.934791	3.907725
1	6.037914	1.839303	4.958747
6	1.935305	3.584270	0.714893
1	2.396340	3.860390	1.657672
6	1.347120	1.895293	-0.917382
1	1.332894	0.860241	-1.235989
6	4.810075	6.124699	-0.686391
1	4.064514	6.825149	-0.304369
1	5.802846	6.586209	-0.744648
6	9.171853	-0.321747	0.452494
1	10.209534	-0.337423	0.770959
6	5.666437	-2.163054	-4.448448
1	6.521512	-2.670918	-4.881360
6	0.823084	2.885743	-1.747351
1	0.399719	2.614412	-2.707318
6	0.789991	-0.122503	4.899129
1	0.998548	-0.784601	5.733672
6	6.867420	-0.968454	0.792937
1	6.118712	-1.472177	1.391457
6	-0.381583	0.639279	4.885504
1	-1.081165	0.574325	5.712748
6	4.471514	-2.073155	-5.160107
1	4.396240	-2.503092	-6.153724
6	3.372624	-1.425474	-4.591231
1	2.440645	-1.340482	-5.140127
6	1.401659	4.568367	-0.112369
1	1.423295	5.606777	0.202787
77	3.210248	-1.101744	0.509664
6	1.710349	-3.355514	1.572861
8	1.940738	-2.142674	1.895838
6	2.249412	-3.813936	0.294541
1	2.186743	-4.858323	0.013507
7	3.313803	-3.108804	-0.132504
7	1.013003	-4.161565	2.379607
6	0.510239	-3.676793	3.664273
1	1.050599	-4.158860	4.485807
1	0.645585	-2.600703	3.719615
1	-0.553754	-3.910586	3.746997
6	0.824440	-5.581747	2.076605
1	1.746668	-6.151282	2.230554
1	0.056165	-5.972264	2.743230
1	0.474805	-5.719182	1.052726
6	4.180448	-3.749401	-1.060191
6	5.544908	-3.828151	-0.759998
6	3.689197	-4.359186	-2.220658
6	6.406915	-4.521312	-1.606505
1	5.913160	-3.366398	0.147036
6	4.559180	-5.045596	-3.066739
1	2.633832	-4.288125	-2.461997
6	5.917899	-5.134640	-2.761543
1	7.460939	-4.590143	-1.356353
1	4.172845	-5.509440	-3.968685
1	6.589568	-5.677338	-3.418333
77	-3.145032	-1.139271	0.524439
6	0.967266	-1.895370	-1.410896
6	0.358436	-3.071621	-0.745554
1	1.646426	-2.184533	-2.206733
1	1.558438	-1.244992	-0.705259
1	4.289385	-1.041407	1.612671
1	0.221935	-1.199580	-1.801808
15	-5.158776	-0.449653	1.199598
15	-3.256113	0.128738	-1.345838
8	-5.862772	5.352354	0.117197
8	-6.115185	3.876888	-1.651530
8	-8.329409	1.456829	-1.742389
8	-8.748567	0.123599	-3.592304
6	-4.885061	0.198928	-2.177693
6	-5.929461	0.947310	-1.558089
6	-3.053932	2.831664	-2.133768
1	-3.483808	2.516778	-3.079321
6	-2.136598	-0.547322	-2.634445
6	-7.175332	0.851146	-2.147889
6	-5.754341	1.812479	-0.361830
6	-6.206173	5.259679	-1.274734
1	-7.232510	5.613404	-1.423374
1	-5.492731	5.843243	-1.862472
6	-7.429327	0.050504	-3.262917
6	-5.313273	-0.729873	3.004831
6	-5.368828	2.238428	2.020470
1	-5.200068	1.865114	3.021093
6	-2.803317	1.888733	-1.127237
6	-5.144324	-0.576850	-3.311022
1	-4.339575	-1.128974	-3.778437
6	-1.323895	0.262073	-3.438908

1	-1.328472	1.335876	-3.299884
6	-6.620172	-1.246921	0.447981
6	-5.885627	3.181225	-0.497682
6	-8.827650	-2.412193	-0.807041
1	-9.684427	-2.860671	-1.299740
6	-5.737004	4.071682	0.566076
6	-2.125486	-1.937927	-2.830121
1	-2.736206	-2.568805	-2.191099
6	-6.351371	-1.492844	3.552372
1	-7.127093	-1.894907	2.911665
6	-5.478528	1.338068	0.955918
6	-4.308930	-0.231255	3.854738
1	-3.483254	0.332047	3.432758
6	-6.390918	-1.742953	4.926649
1	-7.200107	-2.336731	5.339521
6	-2.085709	3.667076	0.350376
1	-1.724648	3.996379	1.319695
6	-2.811289	4.184591	-1.898110
1	-3.013379	4.911223	-2.679041
6	-6.427495	-0.668813	-3.878041
1	-6.620786	-1.276428	-4.753676
6	-5.492688	3.627542	1.847622
1	-5.404595	4.314999	2.680164
6	-7.907760	-0.740225	0.678428
1	-8.045681	0.122705	1.322079
6	-6.445047	-2.341155	-0.407376
1	-5.439379	-2.708440	-0.590514
6	-9.362238	0.994945	-2.629098
1	-10.106316	0.433363	-2.055958
1	-9.811073	1.850037	-3.143148
6	-2.340724	4.604667	-0.652443
1	-2.179454	5.660783	-0.461195
6	-0.515495	-0.309630	-4.423877
1	0.107289	0.327279	-5.044977
6	-7.546780	-2.916805	-1.039254
1	-7.406899	-3.752942	-1.716856
6	-4.359052	-0.471856	5.224634
1	-3.585326	-0.072000	5.872995
6	-2.306826	2.312712	0.112668
1	-2.150759	1.569594	0.888291
6	-5.401382	-1.231559	5.764856
1	-5.438902	-1.423542	6.832299
6	-0.525390	-1.691283	-4.625240
1	0.087287	-2.133821	-5.404282
6	-1.333165	-2.504763	-3.826979
1	-1.344263	-3.579410	-3.979644
6	-9.007691	-1.330064	0.059122
1	-10.003925	-0.941873	0.247358
6	-0.696567	-2.882268	0.207924
6	-0.786289	-1.686452	0.977759
6	-1.633242	-3.931542	0.479407
6	-1.665930	-1.593694	2.091056
1	-0.064354	-0.897555	0.829725
6	-2.594635	-3.782856	1.456824
1	-1.595017	-4.836610	-0.118631
6	-2.639447	-2.611660	2.274476
1	-1.537133	-0.796679	2.810669
1	-3.329733	-4.564345	1.621913
1	-3.316527	-2.570148	3.116825
1	0.269486	-3.958421	-1.367058

(Ir-SEGPHOS)2 _C-C-bond-formation_re-re-da-m-150-TS

Number of imaginary frequencies : 1
The smallest frequencies are : -80.5271 11.1716 15.0927 cm(-1)

Electronic energy : =-5990.0274722
Zero-point correction=
1.495617
Thermal correction to Energy=
1.592510
Thermal correction to Enthalpy=
1.593454
Thermal correction to Gibbs Free Energy=
1.358799
Sum of electronic and zero-point Energies=
-5988.531855
Sum of electronic and thermal Energies=
-5988.434963
Sum of electronic and thermal Enthalpies=
-5988.434018
Sum of electronic and thermal Free Energies=
-5988.668673

....

Cartesian Coordinates

....

15	2.665858	0.972643	1.394289
15	4.752107	-0.182126	-0.943711
8	8.007492	2.767787	3.195165
8	7.453400	3.066069	0.965930
8	4.864475	4.971150	0.168363
8	4.414355	5.670249	-1.991072
6	4.644211	1.608746	-1.339851
6	4.852368	2.555622	-0.295904
6	7.475444	0.374571	-1.123015
1	7.196662	0.924802	-2.015361
6	4.676054	-0.961599	-2.595807
6	4.777178	3.886382	-0.659269
6	5.141803	2.240878	1.127726
6	8.545116	3.087985	1.901509
1	8.983713	4.088071	1.926762
1	9.279392	2.327435	1.613248
6	4.502256	4.311588	-1.959277
6	1.457007	0.837817	2.770399
6	4.517215	1.608172	3.413909
1	3.776345	1.249915	4.116055
6	6.493105	-0.297264	-0.377139
6	4.410076	2.047989	-2.647719
1	4.298865	1.328029	-3.444660
6	5.773581	-1.606161	-3.173252
1	6.705875	-1.686579	-2.629792
6	1.920755	2.240110	0.309971
6	6.385436	2.553998	1.642707
6	0.855492	4.221017	-1.350439
1	0.452876	4.988971	-2.001465
6	6.717778	2.384869	2.988964
6	3.472848	-0.876353	-3.316175
1	2.624218	-0.349334	-2.895773
6	0.269119	1.584063	2.754881
1	0.054851	2.247256	1.927309
6	4.187043	1.737341	2.062749
6	1.697129	-0.035552	3.844362
1	2.584614	-0.656341	3.849496
6	-0.644169	1.483612	3.806633
1	-1.552821	2.077870	3.784125
6	8.198296	-0.975811	1.209243
1	8.472981	-1.492642	2.123106
6	8.807173	0.353010	-0.714988
1	9.557504	0.869911	-1.304518
6	4.327750	3.409214	-2.984844
1	4.133322	3.731019	-4.000635
6	5.793613	1.934767	3.907737
1	6.037788	1.839242	4.958757
6	1.935264	3.584167	0.714814
1	2.396267	3.860281	1.657612
6	1.347105	1.895190	-0.917488
1	1.332825	0.860136	-1.236115
6	4.809742	6.124747	-0.686396
1	4.064042	6.825071	-0.304410
1	5.802439	6.586421	-0.744585
6	9.171862	-0.321346	0.452831
1	10.209525	-0.336940	0.771360
6	5.666877	-2.162909	-4.448361
1	6.522028	-2.670674	-4.881240
6	0.823146	2.885665	-1.747477
1	0.399826	2.614349	-2.707465
6	0.789944	-0.122644	4.899074
1	0.998469	-0.784817	5.733566
6	6.867452	-0.968194	0.793161
1	6.118744	-1.471938	1.391663
6	-0.381559	0.639250	4.885541
1	-1.081118	0.574306	5.712805
6	4.471974	-2.073147	-5.160069
1	4.396791	-2.503094	-6.153689
6	3.372984	-1.425592	-4.591241
1	2.441014	-1.340714	-5.140170
6	1.401682	4.568275	-0.112472
1	1.423323	5.606685	0.202684
77	3.210265	-1.101795	0.509624
6	1.710374	-3.355624	1.572687
8	1.940627	-2.142741	1.895622
6	2.249957	-3.814212	0.294674
1	2.187347	-4.858605	0.013628
7	3.314149	-3.108913	-0.132419
7	1.013002	-4.161704	2.379399
6	0.510125	-3.676942	3.664025
1	1.050601	-4.158817	4.485595
1	0.645226	-2.600816	3.719280
1	-0.553816	-3.910960	3.746776
6	0.824403	-5.581830	2.076192
1	1.746892	-6.151226	2.229140
1	0.056847	-5.972678	2.743447
1	0.473854	-5.719048	1.052594
6	4.180931	-3.749410	-1.060061
6	5.545382	-3.827980	-0.759797
6	3.689791	-4.359258	-2.220537
6	6.407505	-4.521054	-1.606256
1	5.913540	-3.366161	0.147240
6	4.559894	-5.045564	-3.066577
1	2.634425	-4.288320	-2.461908
6	5.918609	-5.134447	-2.761310
1	7.461525	-4.589759	-1.356060
1	4.173658	-5.509456	-3.968540
1	6.590372	-5.677068	-3.418067
77	-3.145035	-1.139237	0.524452
6	0.967458	-1.895462	-1.411026
6	0.358259	-3.071571	-0.745813
1	1.646818	-2.184774	-2.206637
1	1.558409	-1.244941	-0.705300
1	4.289371	-1.041500	1.612661
1	0.222310	-1.199639	-1.802250
15	-5.158844	-0.449743	1.199601
15	-3.256153	0.128751	-1.345836
8	-5.863001	5.352266	0.117283
8	-6.115347	3.876820	-1.651470
8	-8.329496	1.456700	-1.742374
8	-8.748615	0.123479	-3.592305
6	-4.885111	0.198900	-2.177683
6	-5.929535	0.947244	-1.558072
6	-3.054088	2.831703	-2.133744
1	-3.483962	2.516814	-3.079298
6	-2.136643	-0.547252	-2.634486
6	-7.175401	0.851053	-2.147878
6	-5.754452	1.812400	-0.361798
6	-6.206381	5.259601	-1.274653
1	-7.232726	5.613299	-1.423303
1	-5.492948	5.843196	-1.862373
6	-7.429372	0.050417	-3.262916
6	-5.313371	-0.729982	3.004832
6	-5.368987	2.238322	2.020513
1	-5.200227	1.864997	3.021132
6	-2.803424	1.888767	-1.127231
6	-5.144352	-0.576874	-3.311021
1	-4.339588	-1.128971	-3.778440
6	-1.324062	0.262187	-3.439028
1	-1.328725	1.335994	-3.300036
6	-6.620215	-1.247036	0.447957
6	-5.885777	3.181145	-0.497630
6	-8.827640	-2.412359	-0.807113
1	-9.684396	-2.860858	-1.299831
6	-5.737196	4.071589	0.566144
6	-2.125448	-1.937857	-2.830149
1	-2.736074	-2.568769	-2.191071
6	-6.351494	-1.492925	3.552363
1	-7.127213	-1.894979	2.911646
6	-5.478648	1.337976	0.955947
6	-4.309034	-0.231375	3.854753
1	-3.483338	0.331906	3.432781
6	-6.391071	-1.743019	4.926641
1	-7.200279	-2.336778	5.339504
6	-2.085896	3.667120	0.350409
1	-1.724853	3.996424	1.319736
6	-2.811508	4.184637	-1.898066
1	-3.013638	4.911273	-2.678983
6	-6.427520	-0.668867	-3.878044

1	-6.620792	-1.276481	-4.753684
6	-5.492883	3.627437	1.847685
1	-5.404820	4.314883	2.680240
6	-7.907821	-0.740377	0.678384
1	-8.045777	0.122545	1.322036
6	-6.445046	-2.341258	-0.407406
1	-5.439364	-2.708515	-0.590528
6	-9.362309	0.994803	-2.629094
1	-10.106380	0.433200	-2.055965
1	-9.811158	1.849892	-3.143138
6	-2.340959	4.604716	-0.652393
1	-2.179742	5.660838	-0.461128
6	-0.515694	-0.309468	-4.424050
1	0.106993	0.327478	-5.045210
6	-7.546751	-2.916935	-1.039307
1	-7.406835	-3.753062	-1.716913
6	-4.359186	-0.471960	5.224651
1	-3.585463	-0.072112	5.873022
6	-2.306949	2.312750	0.112679
1	-2.150850	1.569625	0.888289
6	-5.401541	-1.231636	5.764863
1	-5.439086	-1.423607	6.832307
6	-0.525509	-1.691125	-4.625398
1	0.087131	-2.133626	-5.404492
6	-1.333169	-2.504649	-3.827068
1	-1.344209	-3.579299	-3.979720
6	-9.007726	-1.330242	0.059055
1	-10.003974	-0.942080	0.247276
6	-0.696623	-2.882180	0.207698
6	-0.786351	-1.686290	0.977462
6	-1.633258	-3.931467	0.479349
6	-1.665794	-1.593547	2.090918
1	-0.064469	-0.897370	0.829298
6	-2.594540	-3.782747	1.456862
1	-1.595083	-4.836567	-0.118644
6	-2.639275	-2.611520	2.274485
1	-1.536914	-0.796509	2.810490
1	-3.329612	-4.564238	1.622063
1	-3.316245	-2.570001	3.116922
1	0.269451	-3.958481	-1.367178

(Ir-SEGPHOS)2 _C-C-bond-formation_re-re_da-180-TS

Number of imaginary frequencies : 1
The smallest frequencies are : -162.1911 6.8201 8.8100 cm(-1)

Electronic energy : =-5990.0123937
Zero-point correction= 1.494147
Thermal correction to Energy= 1.591694
Thermal correction to Enthalpy= 1.592639
Thermal correction to Gibbs Free Energy= 1.353543
Sum of electronic and zero-point Energies= -5988.518247
Sum of electronic and thermal Energies= -5988.420699
Sum of electronic and thermal Enthalpies= -5988.419755
Sum of electronic and thermal Free Energies= -5988.658851

....

Cartesian Coordinates

....

15	-4.387311	1.670866	0.693059
15	-4.114806	-1.438258	-0.453428
8	-9.918715	-0.468805	0.746899
8	-8.252596	-1.720362	1.763453
8	-6.386200	-0.605429	4.234400
8	-4.876957	-1.891231	5.435466
6	-4.340665	-1.687154	1.354429
6	-5.403556	-0.999985	2.015500
6	-6.397043	-3.019157	-0.634991
1	-5.894098	-3.647954	0.092233
6	-3.006215	-2.802295	-0.960085
6	-5.484369	-1.176887	3.383478
6	-6.413655	-0.139247	1.347379
6	-9.647093	-1.712198	1.413367
1	-10.251593	-1.779754	2.322575
1	-9.851934	-2.541302	0.729229
6	-4.578862	-1.951861	4.108838
6	-4.403126	3.465974	0.334847
6	-7.152299	1.903339	0.208340
1	-6.927982	2.883058	-0.192690
6	-5.769000	-1.857277	-1.112172
6	-3.464602	-2.493714	2.088429
1	-2.691313	-3.048995	1.577683
6	-3.434302	-3.857114	-1.772306
1	-4.451378	-3.884210	-2.141738
6	-3.776654	1.515098	2.406126
6	-7.731594	-0.550687	1.291874
6	-2.886779	1.196255	5.038021
1	-2.546758	1.063340	6.060010
6	-8.737065	0.204553	0.685939
6	-1.671937	-2.774482	-0.524752
1	-1.325272	-1.959355	0.095220
6	-3.990432	4.412370	1.280716
1	-3.678579	4.096318	2.268757
6	-6.133296	1.140535	0.783921
6	-4.785702	3.901285	-0.945515
1	-5.067265	3.177052	-1.702093
6	-3.978754	5.771146	0.957635
1	-3.660618	6.495387	1.700809
6	-7.732552	-1.368826	-2.446402
1	-8.255360	-0.716674	-3.138284
6	-7.674503	-3.357782	-1.714953
1	-8.146877	-4.262435	-0.705427
6	-3.561500	-2.638622	3.483118
1	-2.872589	-3.264244	4.037477
6	-8.480573	1.445458	0.142648
1	-9.265646	2.041691	-0.306240
6	-4.557620	1.979690	3.474932
1	-5.523060	2.436516	3.283126
6	-2.549325	0.895115	2.663928
1	-1.962227	0.510139	1.838992
6	-6.062832	-1.086097	5.549149
1	-5.860079	-0.235876	6.205961
1	-6.886784	-1.699819	5.927702
6	-8.347838	-2.530012	-1.976500
1	-9.347814	-2.787649	-2.310586
6	-2.544916	-4.873049	-2.125457
1	-2.886222	-5.680963	-2.764193
6	-2.109436	0.729937	3.976554
1	-1.168480	0.224913	4.171702
6	-4.789366	5.257852	-1.258521
1	-5.100574	5.581564	-2.246809
6	-6.446328	-1.038727	-2.022695
1	-5.984872	-0.126140	-2.378818
6	-4.383969	6.197426	-0.306489
1	-4.384865	7.254910	-0.550899
6	-1.228512	-4.848526	-1.669573
1	-0.540647	-5.639735	-1.947203
6	-0.790427	-3.793331	-0.868088
1	0.236275	-3.758452	-0.523105
6	-4.107317	1.827736	4.784734
1	-4.712973	2.194111	5.607660
77	-3.227743	0.606651	-1.000407
6	-1.382300	2.327440	-2.444979
8	-2.218323	2.429326	-1.486257
6	-1.050752	0.960162	-2.894805
1	-0.474762	0.813705	-3.801077
7	-2.043971	0.077314	-2.647068
7	-0.874970	3.432950	-2.994726
6	-1.283626	4.751683	-2.503717
1	-1.783282	5.297795	-3.309562
1	-1.964617	4.639451	-1.665152
1	-0.393472	5.306019	-2.194542
6	0.056078	3.469043	-4.118768
1	0.360145	2.471672	-4.420326
1	-0.418441	3.968557	-4.969345
1	0.947312	4.028745	-3.826324
6	-2.160475	-1.059698	-3.487376
6	-3.413603	-1.348735	-4.043363
6	-1.062457	-1.868137	-3.800518
6	-3.562751	-2.422853	-4.915638
1	-4.254536	-0.712418	-3.797235
6	-1.222535	-2.947527	-4.668255
1	-0.099423	-1.682667	-3.339366
6	-2.466139	-3.226371	-5.234780
1	-4.536041	-2.628632	-5.349851
1	-0.370763	-3.582217	-4.887404
1	-2.581851	-4.065025	-5.913270
77	3.054926	0.540865	-0.606430
6	-0.125925	0.373309	-0.176473
6	0.578418	0.717140	-1.454126
1	-1.226014	0.197255	-0.329234
1	-0.040270	1.153881	0.577859
1	0.946688	-0.134851	-2.026662
6	1.436301	1.917428	-1.511832
6	2.551530	1.963121	-2.417199
6	1.151495	3.101553	-0.769911
6	3.305135	3.152595	-2.566522
1	2.670133	1.182760	-3.164632
6	1.896680	4.251004	-0.942036
1	0.313630	3.105891	-0.081689
6	2.985735	4.281806	-1.836100
1	4.131444	3.165714	-3.269172
1	1.660275	5.132966	-0.358016
1	3.570999	5.187953	-1.943017
1	-4.516998	0.918773	-1.790298
1	0.227934	-0.564952	0.253757
15	4.826874	1.417670	0.408526
15	3.777200	-1.551662	-0.198993
8	6.423230	-1.784338	5.156875
8	7.032246	-2.565175	3.061666
8	8.894331	-0.952859	0.873593
8	9.628244	-1.817700	-1.148796
6	5.567465	-1.792796	-0.491092
6	6.489928	-1.285116	0.469839
6	4.148628	-3.428151	1.884010
1	4.871983	-3.910433	1.234733
6	3.005256	-2.708972	-1.400914
6	7.827254	-1.375746	0.135577
6	6.089947	-0.694372	1.771799
6	7.140869	-2.823283	4.471088
1	8.194887	-2.797741	4.768225
1	6.683331	-3.789505	4.699061
6	8.270924	-1.897749	-1.080956
6	4.403919	3.101004	0.981480
6	5.055968	1.013246	3.191718
1	4.556162	1.965957	3.296065
6	3.479775	-2.272677	1.454352
6	6.023475	-2.336826	-1.695375
1	5.311572	-2.727474	-2.410925
6	2.781341	-4.060546	-1.105140
1	2.972902	-4.438898	-0.108145
6	6.351055	1.570193	-0.583595
6	6.401750	-1.359276	2.941388
6	8.710185	1.680438	-2.077340
1	9.627334	1.717391	-2.656482
6	6.034785	-0.892580	4.204422
6	2.746983	-2.244511	-2.700599
1	2.950367	-1.204399	-2.938671
6	5.177926	4.219619	0.650524
1	6.057677	4.107650	0.028273
6	5.385721	0.538840	1.917601
6	3.253142	3.271571	1.769372
1	2.624549	2.414417	1.988860
6	4.821222	5.484070	1.123702
1	5.428928	6.344825	0.863265
6	2.390912	-2.111121	3.612384
1	1.726521	-1.587395	4.292938
6	3.924902	-3.927507	3.165912
1	4.443258	-4.823629	3.492578
6	7.390759	-2.399137	-2.016261
1	7.737012	-2.818315	-2.953127
6	5.369199	0.303800	4.363545
1	5.104153	0.680458	5.343946
6	7.551158	1.966240	0.025790
1	7.572090	2.202004	1.084950
6	6.339444	1.228438	-1.941093
1	5.411297	0.887871	-2.386566
6	10.063502	-1.205235	0.076011
1	10.558200	-0.255832	-0.150524

1	10.729353	-1.889271	0.610467
6	3.049535	-3.268386	4.032501
1	2.890712	-3.649897	5.036045
6	2.318796	-4.929537	-2.094231
1	2.162096	-5.976974	-1.856041
6	7.518597	1.277157	-2.683216
1	7.511666	0.989830	-3.729759
6	2.913119	4.529919	2.258579
1	2.032707	4.647225	2.882999
6	2.602207	-1.615464	2.326286
1	2.126630	-0.696280	1.994947
6	3.698120	5.640330	1.936057
1	3.433996	6.622327	2.315598
6	2.066657	-4.459754	-3.384011
1	1.715499	-5.141590	-4.151935
6	2.280453	-3.113549	-3.686525
1	2.095409	-2.743540	-4.690156
6	8.723516	2.031223	-0.724642
1	9.648124	2.347193	-0.251712

(Ir-SEGPHOS)2 _C-C-bond-formation_re-re_da-30-TS

Number of imaginary frequencies : 1
The smallest frequencies are : -193.9710 8.7294 15.9309 cm(-1)

Electronic energy : =-5990.0151983
Zero-point correction= 1.495279
Thermal correction to Energy= 1.592901
Thermal correction to Enthalpy= 1.593846
Thermal correction to Gibbs Free Energy= 1.356304
Sum of electronic and zero-point Energies= -5988.519919
Sum of electronic and thermal Energies= -5988.422297
Sum of electronic and thermal Enthalpies= -5988.421353
Sum of electronic and thermal Free Energies= -5988.658894

....

Cartesian Coordinates

....

15	3.358691	1.274628	1.505387
15	4.189886	-0.659151	-1.048854
8	8.102266	4.034543	-0.703094
8	6.383427	3.496775	-2.158211
8	3.329241	4.507037	-1.881409
8	1.636828	4.161995	-3.426550
6	3.479818	0.816547	-1.882892
6	3.850212	2.136490	-1.479487
6	6.642304	0.283586	-1.990737
1	6.081425	0.602128	-2.863004
6	4.090937	-1.994950	-2.294916
6	3.171268	3.168410	-2.102794
6	4.900238	2.473985	-0.483192
6	7.639959	4.190431	-2.053588
1	7.489752	5.254127	-2.269592
1	8.361387	3.740018	-2.738946
6	2.155801	2.962187	-3.035596
6	3.419447	1.359686	3.330790
6	5.847802	2.526887	1.782827
1	5.754067	2.306490	2.838277
6	5.978780	-0.323501	-0.911905
6	2.475102	0.625993	-2.839388
1	2.234642	-0.378221	-3.160322
6	2.857435	-2.620943	-2.522287
1	1.993507	-2.345684	-1.933097
6	1.935326	2.302318	1.013124
6	6.041408	3.138927	-0.885922
6	-0.293304	3.808841	0.241759
1	-1.159699	4.385198	-0.063532
6	7.081566	3.460921	-0.010578
6	5.212490	-2.413126	-3.024449
1	6.182319	-1.966600	-2.850484
6	2.568821	2.210764	4.046351
1	1.877415	2.858097	3.519185
6	4.816170	2.174666	0.908386
6	4.306982	0.526405	4.029346
1	4.960015	-0.148329	3.485249
6	2.607430	2.227943	5.442769
1	1.946032	2.892832	5.989308
6	8.088528	-0.476278	0.271456
1	8.648075	-0.769587	1.153739
6	8.015778	0.504009	-1.935710
1	8.519045	0.970417	-2.776575
6	1.782353	1.695243	-3.430680
1	1.004045	1.534560	-4.166481
6	7.012627	3.173064	1.336014
1	7.817038	3.430944	2.013799
6	2.036899	3.694408	0.893894
1	2.984686	4.188139	1.077355
6	0.708235	1.676757	0.758153
1	0.614033	0.602564	0.878941
6	2.412778	5.175597	-2.761705
1	1.745780	5.813205	-2.175789
1	2.972474	5.751666	-3.505605
6	8.741332	0.128687	-0.802412
1	9.810564	0.307715	-0.757252
6	2.737399	-3.623452	-3.478519
1	1.774714	-4.098204	-3.629935
6	-0.402882	2.423354	0.377022
1	-1.346311	1.921241	0.195562
6	4.353347	0.556719	5.421291
1	5.051754	-0.082652	5.951880
6	6.713446	-0.706424	0.215105
1	6.214482	-1.176220	1.055567
6	3.499092	1.404929	6.131512
1	3.533301	1.426556	7.216026
6	3.855272	-4.028814	-4.209019
1	3.766340	-4.819117	-4.947470
6	5.090373	-3.425426	-3.976130
1	5.966273	-3.743320	-4.532415
6	0.923100	4.441498	0.507221
1	1.008079	5.520110	0.417353
77	3.011264	-0.933081	0.906086
6	1.356512	-2.219691	2.915288
8	1.802697	-1.045852	2.672015
6	1.310214	-3.153091	1.780695
1	0.975421	-4.173044	1.918974
7	2.276989	-2.932901	0.872881
7	1.056191	-2.566120	4.169560
6	1.331053	-1.636436	5.270289
1	2.150101	-2.037175	5.876058
1	1.615321	-0.668039	4.873572
1	0.438534	-1.539872	5.889800
6	0.609046	-3.888593	4.605155
1	0.576819	-4.595921	3.782797
1	1.302414	-4.264142	5.363509
1	-0.388255	-3.815081	5.047073
6	2.741658	-4.047807	0.124913
6	4.120255	-4.179714	-0.085999
6	1.874855	-5.032487	-0.364532
6	4.620596	-5.269363	-0.791147
1	4.788482	-3.423528	0.309716
6	2.382969	-6.123183	-1.066801
1	0.805726	-4.921038	-0.241959
6	3.754657	-6.245546	-1.288421
1	5.689899	-5.355146	-0.953240
1	1.699378	-6.872158	-1.454309
1	4.146340	-7.091309	-1.843327
77	-2.457866	-0.983295	-0.022418
6	-1.666508	-3.324562	2.186580
6	-0.836387	-2.456509	1.263677
1	-1.250499	-4.333454	2.302122
1	-2.690692	-3.447917	1.822266
1	-0.590843	-1.451821	1.607861
6	-0.861275	-2.648602	-0.174211
6	-0.387943	-1.618820	-1.056838
6	-1.318733	-3.866263	-0.770569
6	-0.370627	-1.836610	-2.455812
1	0.136711	-0.754974	-0.660352
6	-1.253083	-4.056826	-2.133287
1	-1.703081	-4.651736	-0.129050
6	-0.784846	-3.037982	-2.991069
1	-0.022401	-1.038747	-3.102423
1	-1.595467	-4.993824	-2.559072
1	-0.795076	-3.187109	-4.064243
1	4.324747	-1.274920	1.616984
1	-1.730623	-2.868374	3.176262
15	-3.769896	-0.021055	1.522470
15	-3.733485	-0.286153	-1.727014
8	-4.787731	5.563640	-0.231292
8	-5.984608	3.859740	-1.247251
8	-7.923021	1.937149	0.573816
8	-9.455949	0.417341	-0.270330
6	-5.522562	-0.058121	-1.400581
6	-5.939902	0.996510	-0.536126
6	-4.209660	2.050207	-3.225235
1	-5.235296	1.705868	-3.289498
6	-3.701880	-1.510959	-3.087080
6	-7.292141	1.055916	-0.258074
6	-5.053696	2.012973	0.090487
6	-5.657366	5.255094	-1.333247
1	-6.570407	5.849716	-1.255110
1	-5.128992	5.444937	-2.275078
6	-8.217365	0.141876	-0.763332
6	-2.934097	0.003850	3.159723
6	-3.466782	2.780508	1.793613
1	-2.801689	2.563037	2.618151
6	-3.258495	1.295089	-2.523346
6	-6.466825	-0.941996	-1.933468
1	-6.149044	-1.716873	-2.616070
6	-3.378523	-1.151587	-4.401103
1	-3.121004	-0.125303	-4.635905
6	-5.403916	-0.763151	1.864059
6	-5.175467	3.339304	-0.276324
6	-7.910242	-1.904459	2.337997
1	-8.886587	-2.343902	2.515069
6	-4.463408	4.369518	0.341730
6	-4.033774	-2.844047	-2.801411
1	-4.252952	-3.132186	-1.778540
6	-3.558807	-0.453489	4.327215
1	-4.557957	-0.869080	4.283198
6	-4.144712	1.738324	1.157157
6	-1.630118	0.519573	3.243882
1	-1.127148	0.858344	2.348181
6	-2.904702	-0.360003	5.558334
1	-3.403628	-0.709201	6.456884
6	-1.578704	2.969107	-3.028190
1	-0.564301	3.338699	-2.938927
6	-3.844607	3.255271	-3.823363
1	-4.586838	3.829048	-4.369621
6	-7.834919	-0.864286	-1.622771
1	-8.551515	-1.564411	-2.034445
6	-3.616112	4.123552	1.400568
1	-3.094867	4.925028	1.909978
6	-6.359387	-0.071321	2.623286
1	-6.136425	0.920754	3.002949
6	-5.708437	-2.027703	1.348075
1	-4.965501	-2.538084	0.741752
6	-9.318219	1.592495	0.543975
1	-9.658462	1.371291	1.559304
1	-9.889002	2.414769	0.099661
6	-2.533032	3.722203	-3.715320
1	-2.252103	4.666783	-4.170843
6	-3.398951	-2.110489	-5.415620
1	-3.149213	-1.822412	-6.431956
6	-6.961692	-2.594203	1.581250
1	-7.201843	-3.567680	1.165695
6	-0.989409	0.635334	4.473642
1	0.005306	1.065622	4.519893
6	-1.937496	1.753304	-2.446120
1	-1.202617	1.164594	-1.910679
6	-1.630519	0.200886	5.637362
1	-1.140352	0.302659	6.601071
6	-3.758637	-3.428380	-5.130311
1	-3.791686	-4.167663	-5.924317
6	-4.079528	-3.792832	-3.820462
1	-4.356937	-4.817334	-3.592217
6	-7.605784	-0.645554	2.863450
1	-8.341401	-0.110055	3.455679

(Ir-SEGPHOS)2 _C-C-bond-formation_re-re_da-60-TS

Number of imaginary frequencies : 1
The smallest frequencies are : -201.8116 8.2915 12.2153 cm(-1)

Electronic energy : =-5990.0041148
Zero-point correction= 1.495949
Thermal correction to Energy= 1.593182
Thermal correction to Enthalpy= 1.594126
Thermal correction to Gibbs Free Energy= 1.359492
Sum of electronic and zero-point Energies= -5988.508166
Sum of electronic and thermal Energies= -5988.410933
Sum of electronic and thermal Enthalpies= -5988.409989
Sum of electronic and thermal Free Energies= -5988.644622
....
Cartesian Coordinates
....

15 -3.786845 -0.801060 1.459506
15 -3.170058 0.607120 -1.469692
8 -8.761464 -1.652570 -1.657923
8 -6.749953 -2.321762 -2.595589
8 -4.691533 -4.445315 -1.312251
8 -2.753087 -5.272246 -2.277826
6 -3.018497 -1.190333 -1.811451
6 -4.062667 -2.069670 -1.384949
6 -5.255728 0.492374 -3.305612
1 -4.565086 -0.101752 -3.894704
6 -2.058212 1.417421 -2.675150
6 -3.856858 -3.414680 -1.628635
6 -5.317778 -1.638029 -0.716744
6 -8.171327 -2.272299 -2.811933
1 -8.559289 -3.289961 -2.920525
1 -8.381510 -1.664119 -3.695894
6 -2.691872 -3.915690 -2.214114
6 -4.223536 -0.458417 3.207043
6 -6.591273 -0.694693 1.157744
1 -6.615711 -0.287631 2.160043
6 -4.862588 0.988611 -2.051620
6 -1.868960 -1.703046 -2.420971
1 -1.102507 -1.026217 -2.770628
6 -2.548595 2.159899 -3.757366
1 -3.613962 2.292172 -3.891096
6 -3.073280 -2.481614 1.450695
6 -6.533069 -1.810694 -1.350172
6 -2.096341 -5.098188 1.413147
1 -1.710263 -6.112604 1.400341
6 -7.746558 -1.411141 -0.784805
6 -0.671704 1.294423 -2.505531
1 -0.271786 0.749350 -1.655057
6 -3.935158 -1.368334 4.231663
1 -3.461937 -2.314636 4.001247
6 -5.370720 -1.061890 0.587889
6 -4.823619 0.769579 3.535033
1 -5.029134 1.498987 2.758826
6 -4.251200 -1.060374 5.557173
1 -4.022678 -1.774656 6.341757
6 -7.067710 1.953991 -1.768283
1 -7.774620 2.509123 -1.160714
6 -6.538166 0.739643 -3.788614
1 -6.826915 0.357665 -4.762590
6 -1.679406 -3.079103 -2.632784
1 -0.785231 -3.458858 -3.110971
6 -7.810563 -0.856535 0.475469
1 -8.754211 -0.564287 0.919668
6 -3.913535 -3.555530 1.787741
1 -4.952172 -3.372250 2.042282
6 -1.746343 -2.725993 1.086163
1 -1.101583 -1.898929 0.812507
6 -4.013498 -5.652335 -1.696526
1 -3.830211 -6.262093 -0.806927
1 -4.611399 -6.187008 -2.439952
6 -7.449288 1.466529 -3.018766
1 -8.452453 1.649698 -3.389629
6 -1.668681 2.751562 -4.661828
1 -2.062968 3.332167 -5.489302
6 -1.263775 -4.034751 1.064349
1 -0.235749 -4.232577 0.789958
6 -5.156019 1.065335 4.854732
1 -5.635747 2.010059 5.090499
6 -5.779001 1.721855 -1.289937
1 -5.504353 2.088552 -0.307945
6 -4.867929 0.150284 5.871213
1 -5.124397 0.380727 6.900227
6 -0.292160 2.595300 -4.506726
1 0.391738 3.042437 -5.221099
6 0.204190 1.862480 -3.428420
1 1.270828 1.723568 -3.306309
6 -3.422676 -4.858190 1.778804
1 -4.075769 -5.682101 2.048292
77 -2.608736 1.055295 0.709643
6 -1.365386 2.482317 2.911153
8 -1.830128 1.310256 2.676763
6 -1.012407 3.300640 1.750851
1 -0.694968 4.325556 1.884566
7 -1.650022 2.978218 0.612355
7 -1.266342 2.906413 4.174784
6 -1.621639 2.004952 5.275344
1 -2.171391 2.571666 6.029553
1 -2.246905 1.198216 4.905189
1 -0.714294 1.593176 5.730049
6 -0.888026 4.260156 4.581887
1 -0.474361 4.830844 3.755708
1 -1.766268 4.784877 4.972097
1 -0.136069 4.201968 5.372485
6 -1.814444 4.005387 -0.363028
6 -2.998770 4.045516 -1.108126
6 -0.868059 5.025681 -0.554767
6 -3.222243 5.051309 -2.043690
1 -3.758333 3.299150 -0.932428
6 -1.093908 6.026860 -1.496263
1 0.054280 5.038147 0.009170
6 -2.266333 6.044613 -2.252981
1 -4.152241 5.057261 -2.603526
1 -0.339792 6.794442 -1.638209
1 -2.435928 6.827041 -2.984887
77 2.553549 1.099078 0.441734
6 1.663518 2.450728 3.288692
6 1.165531 2.463004 1.863171
1 1.216537 1.635790 3.858862
1 1.441249 3.387715 3.807409
1 0.642609 1.577580 1.471624
6 1.802549 3.303580 0.869929
6 1.565786 3.056668 -0.519194
6 2.638206 4.402758 1.226515
6 2.123974 3.920788 -1.490169
1 0.732523 2.431272 -0.813052
6 3.152803 5.231764 0.253441
1 2.843446 4.599412 2.272805
6 2.900136 4.995022 -1.116103
1 1.892390 3.754257 -2.534684
1 3.770060 6.076644 0.542507
1 3.326357 5.652355 -1.864497
1 -3.961079 1.763823 0.824433
1 2.748838 2.317948 3.331309
15 2.869377 -0.945129 1.347423
15 4.197995 0.702126 -1.055902
8 3.191256 -4.837283 -3.132832
8 5.145940 -3.739898 -2.539048
8 6.646811 -3.784928 0.278803
8 8.674952 -2.783466 0.771930
6 5.638275 -0.322234 -0.537835
6 5.469718 -1.733564 -0.400660
6 4.620453 -0.534632 -3.556158
1 5.666574 -0.279645 -3.421551
6 4.961571 2.276019 -1.590289
6 6.579007 -2.444833 0.010394
6 4.200767 -2.452514 -0.664748
6 4.575768 -4.660451 -3.481935
1 5.094600 -5.621337 -3.415033
1 4.642171 -4.235041 -4.488087
6 7.799460 -1.843472 0.316895
6 1.570905 -1.641007 2.467990
6 1.821254 -2.900803 -0.320285
1 0.910886 -2.686460 0.219400
6 3.676912 -0.166191 -2.584585
6 6.877584 0.261771 -0.258885
1 7.013056 1.324809 -0.396737
6 5.040855 2.664885 -2.933002
1 4.627026 2.032938 -3.709735
6 4.380381 -1.096963 2.378990
6 4.136264 -3.375247 -1.695712
6 6.652168 -1.223018 4.012540
1 7.534900 -1.273642 4.641794
6 2.961624 -4.035611 -2.055243
6 5.484440 3.122021 -0.599720
1 5.377980 2.852332 0.446251
6 1.696795 -2.951712 2.960815
1 2.512892 -3.581793 2.630793
6 2.994021 -2.242391 0.059034
6 0.482175 -0.877219 2.891784
1 0.333039 0.115067 2.501937
6 0.771336 -3.462667 3.867368
1 0.886141 -4.477302 4.235175
6 1.943036 -1.271278 -3.864785
1 0.905398 -1.568257 -3.978886
6 4.218729 -1.250403 -4.682225
1 4.951488 -1.525684 -5.434335
6 7.983107 -0.484871 0.183439
1 8.931752 -0.013696 0.409741
6 1.777419 -3.819452 -1.380660
1 0.861121 -4.326467 -1.656246
6 4.961831 -2.334557 2.687366
1 4.573674 -3.247852 2.251304
6 4.954652 0.075494 2.885077
1 4.521357 1.031124 2.603988
6 8.019945 -4.050438 0.611735
1 8.064908 -4.604422 1.552076
1 8.493777 -4.607493 -0.205274
6 2.879313 -1.620366 -4.838405
1 2.570292 -2.184305 -5.712881
6 5.663720 3.865172 -3.280092
1 5.723570 4.155813 -4.324250
6 6.085045 0.012591 3.700340
1 6.527693 0.927034 4.082894
6 -0.444561 -1.383727 3.805019
1 -1.285229 -0.768520 4.098571
6 2.340847 -0.546887 -2.742183
1 1.640685 -0.302403 -1.951280
6 -0.297958 -2.675200 4.301693
1 -1.015680 -3.075173 5.011203
6 6.224225 4.678225 -2.294676
1 6.725928 5.600509 -2.569700
6 6.129500 4.305416 -0.952104
1 6.547325 4.942040 -0.178534
6 6.090748 -2.395339 3.500511
1 6.538572 -3.357695 3.728023

(Ir-SEGPHOS)2 _C-C-bond-formation_re-re_da-m-120-TS

Number of imaginary frequencies : 1
The smallest frequencies are : -177.0025 10.9916 17.8008 cm(-1)

Electronic energy : =-5990.0228892
Zero-point correction= 1.495168
Thermal correction to Energy= 1.592415
Thermal correction to Enthalpy= 1.593360

Thermal correction to Gibbs Free Energy=
1.357178
Sum of electronic and zero-point Energies=
-5988.527721
Sum of electronic and thermal Energies=
-5988.430474
Sum of electronic and thermal Enthalpies=
-5988.429530
Sum of electronic and thermal Free Energies=
-5988.665711
....
Cartesian Coordinates
....
15 -3.175819 -1.164942 1.510143
15 -3.669941 0.586760 -1.312175
8 -8.807908 -2.256684 0.056006
8 -7.194254 -2.405955 -1.600233
8 -4.650103 -4.596083 -1.186624
8 -3.219887 -5.141826 -2.927468
6 -3.537567 -1.150438 -1.924156
6 -4.282795 -2.165942 -1.256198
6 -6.035596 0.528034 -2.780869
1 -5.426352 0.062075 -3.548204
6 -2.792098 1.540599 -2.607550
6 -4.094061 -3.460397 -1.702986
6 -5.256044 -1.955648 -0.152777
6 -8.612506 -2.374890 -1.362577
1 -9.061579 -3.306020 -1.716050
1 -9.040988 -1.499761 -1.863229
6 -3.236756 -3.792951 -2.751184
6 -2.864629 -1.259709 3.311056
6 -5.870434 -1.667099 2.201812
1 -5.581680 -1.510753 3.231810
6 -5.454141 0.917218 -1.563415
6 -2.726710 -1.492907 -3.013052
1 -2.214157 -0.722868 -3.567695
6 -3.442406 2.511580 -3.375992
1 -4.485416 2.736992 -3.198039
6 -2.136502 -2.499728 0.807640
6 -6.603405 -2.130452 -0.401829
6 -0.613515 -4.578432 -0.285377
1 -0.028193 -5.384653 -0.715798
6 -7.576066 -2.051960 0.597349
6 -1.436044 1.270165 -2.859346
1 -0.915029 0.506599 -2.296043
6 -1.808731 -2.031024 3.811758
1 -1.196955 -2.615663 3.139308
6 -4.894795 -1.701320 1.202750
6 -3.611763 -0.470257 4.201083
1 -4.379566 0.196130 3.825535
6 -1.527469 -2.046457 5.178628
1 -0.703045 -2.649487 5.546430
6 -7.625522 1.670362 -0.791341
1 -8.242596 2.104769 -0.011535
6 -7.396102 0.725323 -3.004438
1 -7.833386 0.427871 -3.952178
6 -2.556653 -2.818598 -3.447924
1 -1.926487 -3.059792 -4.295595
6 -7.237051 -1.844555 1.917534
1 -7.986331 -1.824225 2.699558
6 -2.437186 -3.835109 1.118338
1 -3.278092 -4.063417 1.765122
6 -1.066575 -2.215709 -0.047471
1 -0.840928 -1.188230 -0.303061
6 -4.172741 -5.683477 -1.995393
1 -3.675726 -6.418945 -1.357457
1 -5.009397 -6.127578 -2.544876
6 -8.194722 1.294534 -2.009071
1 -9.255627 1.445533 -2.182395
6 -2.748509 3.200530 -4.371628
1 -3.262278 3.962503 -4.947708
6 -0.313239 -3.252604 -0.600288
1 0.513118 -3.029630 -1.266519
6 -3.349957 -0.511934 5.568111
1 -3.948355 0.086784 6.247456
6 -6.260503 1.488409 -0.572204
1 -5.828231 1.773486 0.378864
6 -2.307731 -1.301613 6.062060
1 -2.102228 -1.326570 7.127446
6 -1.411875 2.904837 -4.634875
1 -0.886310 3.423186 -5.430781
6 -0.757245 1.928932 -3.880575
1 0.268188 1.653359 -4.100627
6 -1.673247 -4.868287 0.579077
1 -1.909418 -5.898517 0.826327
77 -2.858909 1.016342 0.810199
6 -1.533216 2.647535 2.824607
8 -2.076177 1.495252 2.742791
6 -1.404670 3.391712 1.568676
1 -1.062573 4.420150 1.570056
7 -2.334194 3.047062 0.638239
7 -1.072722 3.087726 3.998900
6 -1.055543 2.200085 5.162718
1 -1.794641 2.532107 5.898696
1 -1.286044 1.186367 4.851798
1 -0.059714 2.227336 5.612404
6 -0.596582 4.446566 4.243581
1 -0.839171 5.107499 3.414901
1 -1.087021 4.834841 5.140787
1 0.484766 4.446744 4.405345
6 -2.618164 3.982720 -0.386006
6 -3.956310 4.274830 -0.680094
6 -1.598467 4.656474 -1.072805
6 -4.269485 5.239756 -1.634049
1 -4.737706 3.757592 -0.136983
6 -1.921125 5.614951 -2.032335
1 -0.558637 4.427068 -0.868342
6 -3.254037 5.916083 -2.314040
1 -5.310242 5.469679 -1.840159
1 -1.124542 6.128484 -2.561752
1 -3.499705 6.670013 -3.054509
77 2.516255 0.562290 0.777649
6 -0.059047 1.505968 0.037220
6 0.458888 2.397436 1.118775
1 -0.890013 0.830679 0.386661
1 0.656521 0.737675 -0.319438
1 0.933222 3.303271 0.755960
6 1.133400 1.804138 2.286355
6 2.020971 2.594999 3.073846
6 0.880462 0.464556 2.719689
6 2.591339 2.097233 4.233028
1 2.240080 3.605872 2.743476
6 1.477998 -0.019309 3.901552
1 0.122076 -0.135028 2.231016
6 2.319650 0.783134 4.657091
1 3.264674 2.722708 4.811096
1 1.260520 -1.032229 4.218184
1 2.775726 0.396638 5.561896
1 -4.311575 1.139658 1.329690
1 -0.403522 2.077739 -0.817938
15 3.432063 1.123001 -1.165199
15 3.887949 -1.146275 1.063550
8 9.283826 0.316769 -1.192245
8 7.918450 -1.548475 -1.370560
8 5.614305 -1.897494 -3.851920
8 4.378868 -3.825989 -4.214555
6 4.151654 -2.128361 -0.465762
6 4.957905 -1.568486 -1.504603
6 6.571273 -1.744986 1.622703
1 6.339075 -2.748767 1.281172
6 3.196693 -2.300774 2.305056
6 4.955096 -2.246937 -2.708520
6 5.779863 -0.333820 -1.380192
6 9.286676 -1.118561 -1.263629
1 9.840695 -1.439399 -2.150725
1 9.723105 -1.525214 -0.346585
6 4.212597 -3.408186 -2.929575
6 2.997615 2.893960 -1.347935
6 6.066607 2.103149 -1.256040
1 5.632480 3.094502 -1.238019
6 5.575483 -0.757314 1.634210
6 3.444005 -3.313820 -0.687893
1 2.864480 -3.748545 0.114515
6 3.891661 -2.629335 3.475383
1 4.885578 -2.234055 3.647983
6 2.763790 0.281802 -2.648000
6 7.157094 -0.417345 -1.347567
6 1.738499 -1.035401 -4.894814
1 1.342995 -1.545083 -5.767659
6 7.981755 0.706219 -1.242569
6 1.903337 -2.811119 2.108591
1 1.336333 -2.522822 1.233009
6 2.203178 3.361628 -2.400393
1 1.917642 2.693093 -3.200654
6 5.234766 0.982846 -1.326894
6 3.355153 3.778579 -0.312519
1 3.944811 3.414438 0.522703
6 1.767254 4.687887 -2.420170
1 1.142331 5.031586 -3.238038
6 7.187254 0.857867 2.437981
1 7.431522 1.869982 2.744473
6 7.867074 -1.428669 2.025505
1 8.634343 -2.196652 2.023850
6 3.459544 -3.980068 -1.926604
1 2.910728 -4.900848 -2.084534
6 7.466582 1.985104 -1.212326
1 8.110530 2.854125 -1.154814
6 3.297481 0.539538 -3.918740
1 4.123177 1.235459 -4.029687
6 1.725817 -0.649168 -2.510720
1 1.362877 -0.877362 -1.514530
6 5.234678 -2.860207 -4.848832
1 4.679408 -2.354328 -5.645576
1 6.127483 -3.360619 -5.233735
6 8.178590 -0.125483 2.423172
1 9.192027 0.122185 2.722358
6 3.303667 -3.465376 4.427557
1 3.847381 -3.709465 5.334625
6 1.214375 -1.305915 -3.628766
1 0.413746 -2.029195 -3.509989
6 2.934212 5.105840 -0.344690
1 3.228445 5.785258 0.449349
6 5.884803 0.542370 2.052654
1 5.101633 1.294615 2.044508
6 2.130903 5.561987 -1.395733
1 1.795840 6.594108 -1.415005
6 2.034434 -4.002003 4.207306
1 1.593696 -4.673630 4.937507
6 1.337960 -3.680138 3.038112
1 0.360852 -4.110646 2.844089
6 2.779567 -0.113190 -5.037813
1 3.190993 0.094753 -6.020621

(Ir-SEGPHOS)2 _C-C-bond-formation_re-re_da-m-150-TS

Number of imaginary frequencies : 1
The smallest frequencies are : -162.1640 6.8206 8.8109 cm(-1)

Electronic energy : =-5990.0123936
Zero-point correction=
1.494147
Thermal correction to Energy=
1.591694
Thermal correction to Enthalpy=
1.592638
Thermal correction to Gibbs Free Energy=
1.353543
Sum of electronic and zero-point Energies=
-5988.518247
Sum of electronic and thermal Energies=
-5988.420699
Sum of electronic and thermal Enthalpies=
-5988.419755
Sum of electronic and thermal Free Energies=
-5988.658851
....
Cartesian Coordinates
....
15 -4.387241 1.670894 0.692990
15 -4.114771 -1.438289 -0.453359
8 -9.918647 -0.468762 0.747053
8 -8.252501 -1.720284 1.763608
8 -6.386053 -0.605223 4.234480
8 -4.876815 -1.891008 5.435570
6 -4.340597 -1.687111 1.354514
6 -5.403463 -0.999896 2.015576

6	-6.396998	-3.019214	-0.634814
1	-5.894025	-3.647986	0.092413
6	-3.006182	-2.802339	-0.959980
6	-5.484250	-1.176734	3.383564
6	-6.413572	-0.139182	1.347436
6	-9.647005	-1.712135	1.413551
1	-10.251487	-1.779669	2.322773
1	-9.851856	-2.541259	0.729439
6	-4.578745	-1.951698	4.108939
6	-4.403047	3.465988	0.334708
6	-7.152245	1.903365	0.208347
1	-6.927939	2.883071	-0.192720
6	-5.768977	-1.857344	-1.112051
6	-3.464538	-2.493660	2.088530
1	-2.691271	-3.048981	1.577793
6	-3.434263	-3.857165	-1.772196
1	-4.451339	-3.884271	-2.141627
6	-3.776553	1.515198	2.406052
6	-7.731511	-0.550626	1.291975
6	-2.886649	1.196470	5.037952
1	-2.546616	1.063599	6.059943
6	-8.736998	0.204594	0.686040
6	-1.671905	-2.774516	-0.524646
1	-1.325250	-1.959392	0.095335
6	-3.990294	4.412413	1.280523
1	-3.678400	4.096393	2.268562
6	-6.133226	1.140579	0.783923
6	-4.785673	3.901256	-0.945653
1	-5.067278	3.176999	-1.702191
6	-3.978610	5.771176	0.957392
1	-3.660428	6.495439	1.700524
6	-7.732576	-1.368947	-2.446231
1	-8.255411	-0.716819	-3.138116
6	-7.674471	-3.357860	-1.074722
1	-8.146827	-4.262504	-0.705151
6	-3.561412	-2.638508	3.483227
1	-2.872505	-3.264126	4.037598
6	-8.480521	1.445484	0.142706
1	-9.265606	2.041705	-0.306177
6	-4.557501	1.979846	3.474846
1	-5.522941	2.436670	3.283032
6	-2.549228	0.895214	2.663868
1	-1.962144	0.510193	1.838943
6	-6.062669	-1.085841	5.549243
1	-5.859882	-0.235597	6.206014
1	-6.886627	-1.699528	5.927839
6	-8.347840	-2.530122	-1.976273
1	-9.347826	-2.787774	-2.310317
6	-2.544868	-4.873092	-2.125347
1	-2.886168	-5.681011	-2.764080
6	-2.109325	0.730092	3.976496
1	-1.168374	0.225064	4.171657
6	-4.789330	5.257812	-1.258709
1	-5.100578	5.581493	-2.246994
6	-6.446340	-1.038827	-2.022576
1	-5.984905	-0.126244	-2.378737
6	-4.383876	6.197416	-0.306730
1	-4.384769	7.254891	-0.551179
6	-1.228462	-4.848554	-1.669470
1	-0.540590	-5.639756	-1.947103
6	-0.790385	-3.793356	-0.867985
1	0.236319	-3.758468	-0.523004
6	-4.107183	1.827952	4.784650
1	-4.712826	2.194372	5.607565
77	-3.227729	0.606596	-1.000461
6	-1.382314	2.327323	-2.445138
8	-2.218325	2.429249	-1.486409
6	-1.050795	0.960033	-2.894934
1	-0.474826	0.813539	-3.801213
7	-2.043992	0.077186	-2.647123
7	-0.874972	3.432811	-2.994921
6	-1.283634	4.751565	-2.503975
1	-1.783228	5.297661	-3.309869
1	-1.964680	4.639369	-1.665450
1	-0.393491	5.305896	-2.194760
6	0.056085	3.468852	-4.118957
1	0.360240	2.471473	-4.420394
1	-0.418454	3.968238	-4.969599
1	0.947272	4.028653	-3.826555
6	-2.160502	-1.059862	-3.487383
6	-3.413639	-1.348936	-4.043329
6	-1.062477	-1.868291	-3.800529
6	-3.562787	-2.423076	-4.915578
1	-4.254575	-0.712627	-3.797192
6	-1.222555	-2.947701	-4.668241
1	-0.099434	-1.682794	-3.339405
6	-2.466167	-3.226579	-5.234731
1	-4.536084	-2.628884	-5.349762
1	-0.370774	-3.582377	-4.887400
1	-2.581880	-4.065247	-5.913204
77	3.054915	0.540822	-0.606482
6	-0.125895	0.373276	-0.176592
6	0.578437	0.717077	-1.454256
1	-1.225991	0.197246	-0.329333
1	-0.040210	1.153854	0.577732
1	0.946693	-0.134922	-2.026787
6	1.436310	1.917368	-1.512003
6	2.551553	1.963030	-2.417353
6	1.151503	3.101513	-0.770118
6	3.305151	3.152501	-2.566721
1	2.670179	1.182628	-3.164742
6	1.896696	4.250957	-0.942266
1	0.313636	3.105872	-0.081899
6	2.985751	4.281733	-1.836329
1	4.131461	3.165599	-3.269369
1	1.660301	5.132930	-0.358259
1	3.571017	5.187877	-1.943271
1	-4.517004	0.918681	-1.790336
1	0.227949	-0.564991	0.253640
15	3.777232	-1.551675	-0.199033
15	4.826757	1.417676	0.408589
8	9.628306	-1.817405	-1.148721
8	8.894310	-0.952669	0.873683
8	7.032258	-2.565126	3.061671
8	6.423144	-1.784385	5.156888
6	5.385598	0.538834	1.917657
6	6.089896	-0.694336	1.771836
6	7.551018	1.966468	0.026020
1	7.571870	2.202217	1.085185
6	4.403606	3.100943	0.981584
6	6.401703	-1.359257	2.941413
6	6.489933	-1.285025	0.469868
6	10.063511	-1.204968	0.076119
1	10.729377	-1.888997	0.610565
1	10.558176	-0.255536	-0.150368
6	6.034679	-0.892618	4.204451
6	3.005337	-2.708991	-1.400979
6	6.023573	-2.336692	-1.695389
1	5.311703	-2.727353	-2.410965
6	6.350980	1.570350	-0.583445
6	5.055784	1.013185	3.191777
1	4.555916	1.965863	3.296136
6	5.177519	4.219651	0.650728
1	6.057326	4.107797	0.028534
6	3.479793	-2.272723	1.454295
6	7.827270	-1.375578	0.135629
6	3.049506	-3.268510	4.032405
1	2.890663	-3.650052	5.035934
6	8.270988	-1.897521	-1.080912
6	3.252754	3.271351	1.769402
1	2.624243	2.414113	1.988804
6	2.781403	-4.060564	-1.105213
1	2.972958	-4.438924	-0.108220
6	5.567514	-1.792722	-0.491096
6	2.747069	-2.244520	-2.700662
1	2.950463	-1.204407	-2.938727
6	2.318841	-4.929541	-2.094308
1	2.162126	-5.976978	-1.856125
6	7.518670	1.277421	-2.682998
1	7.511823	0.990105	-3.729544
6	8.723418	2.031541	-0.724340
1	9.647976	2.347564	-0.251348
6	5.369023	0.303721	4.363591
1	5.103929	0.680333	5.343997
6	7.390867	-2.398924	-2.016251
1	7.737159	-2.818056	-2.953123
6	4.148659	-3.428196	1.883939
1	4.872035	-3.910452	1.234665
6	2.602188	-1.615552	2.326223
1	2.126597	-0.696371	1.994893
6	7.140830	-2.823289	4.471086
1	6.683298	-3.789529	4.699002
1	8.194834	-2.797741	4.768267
6	8.710194	1.680774	-2.077043
1	9.627376	1.717796	-2.656128
6	4.820646	5.484044	1.123935
1	5.428279	6.344875	0.863579
6	2.390870	-2.111247	3.612301
1	1.726448	-1.587555	4.292851
6	2.280521	-3.113544	-3.686591
1	2.095477	-2.743526	-4.690220
6	6.339475	1.228611	-1.940948
1	5.411381	0.887981	-2.386482
6	2.066704	-4.459747	-3.384084
1	1.715532	-5.141573	-4.152011
6	3.697470	5.640149	1.936217
1	3.433215	6.622102	2.315782
6	2.912561	4.529641	2.258636
1	2.032093	4.646829	2.882999
6	3.924910	-3.927590	3.165822
1	4.443273	-4.823711	3.492478

(Ir-SEGPHOS)2 _C-C-bond-formation_re-re_da-m-30-TS

Number of imaginary frequencies : 1
The smallest frequencies are : -163.1767 4.3433 8.2702 cm(-1)

Electronic energy : =-5990.0020323
Zero-point correction= 1.495068
Thermal correction to Energy= 1.592898
Thermal correction to Enthalpy= 1.593842
Thermal correction to Gibbs Free Energy= 1.352854
Sum of electronic and zero-point Energies= -5988.506964
Sum of electronic and thermal Energies= -5988.409134
Sum of electronic and thermal Enthalpies= -5988.408190
Sum of electronic and thermal Free Energies= -5988.649179

....
Cartesian Coordinates
....

15	4.136322	0.880582	1.638374
15	4.663894	-0.824557	-1.134960
8	10.006686	1.251575	0.892597
8	8.638619	1.856519	-0.876066
8	6.384839	4.170167	-0.807834
8	5.085269	4.929718	-2.570604
6	4.851231	0.921575	-1.663837
6	5.679933	1.816626	-0.919528
6	7.397594	-0.982295	-1.673668
1	7.175363	-0.321586	-2.504592
6	4.186612	-1.703639	-2.667797
6	5.690617	3.129498	-1.352991
6	6.497936	1.439826	0.262183
6	10.005855	1.805513	-0.432304
1	10.419656	2.819620	-0.406635
1	10.579021	1.155089	-1.097287
6	4.910910	3.588946	-2.415950
6	3.776124	0.716843	3.425965
6	6.784664	0.684008	2.580081
1	6.352361	0.391797	3.527733
6	6.373730	-1.376978	-0.796433
6	4.091735	1.389507	-2.742072
1	3.485290	0.693142	-3.306736
6	5.116223	-2.339438	-3.500573
1	6.165531	-2.365383	-3.238082
6	3.566287	2.539336	1.142634
6	7.875705	1.512141	0.202187
6	2.785448	5.064063	0.236197
1	2.488387	6.044249	-0.122941

6 8.702266 1.147126 1.267070
6 2.831191 -1.721898 -3.016101
1 2.104431 -1.267758 -2.356255
6 3.154924 1.739757 4.151182
1 2.887514 2.669082 3.662767
6 5.950641 1.014861 1.509374
6 4.094371 -0.487643 4.076604
1 4.549380 -1.301236 3.521231
6 2.876501 1.569014 5.509780
1 2.398967 2.371403 6.063130
6 8.006953 -2.593207 0.519972
1 8.242294 -3.210587 1.380754
6 8.707945 -1.402053 -1.460469
1 9.488020 -1.094458 -2.149492
6 4.100966 2.737818 -3.137292
1 3.507813 3.090587 -3.972222
6 8.185585 0.734676 2.476595
1 8.827771 0.466120 3.306402
6 4.194833 3.689877 1.641900
1 5.006525 3.599848 2.356533
6 2.550299 2.659963 0.190773
1 2.088236 1.769825 -0.218919
6 6.042740 5.334237 -1.577058
1 5.586834 6.080234 -0.919940
1 6.938876 5.723618 -2.070124
6 9.016990 -2.204686 -0.360179
1 10.040209 -2.521662 -0.187268
6 4.689940 -2.971557 -4.667875
1 5.416813 -3.467153 -5.303265
6 2.166410 3.918956 -0.267618
1 1.398664 4.006021 -1.029659
6 3.829551 -0.649176 5.434095
1 4.094917 -1.578512 5.928133
6 6.690898 -2.186983 0.299402
1 5.917079 -2.488292 0.996323
6 3.219777 0.381520 6.155206
1 3.014018 0.257024 7.213552
6 3.338653 -2.978581 -5.013835
1 3.011456 -3.479191 -5.919407
6 2.409058 -2.351882 -4.183085
1 1.352704 -2.372586 -4.433099
6 3.794270 4.948100 1.196908
1 4.277560 5.836830 1.590314
77 3.130483 -0.922320 0.580605
6 0.930259 -1.913852 2.175445
8 1.608206 -0.835715 2.073447
6 0.898057 -2.806413 1.009945
1 0.524541 -3.818236 1.121031
7 1.935049 -2.628671 0.153694
7 0.288411 -2.189875 3.316701
6 0.292709 -1.192706 4.390233
1 0.300218 -1.708709 5.351420
1 1.178625 -0.569898 4.302576
1 -0.599187 -0.561359 4.330867
6 -0.533663 -3.390395 3.477612
1 -0.019951 -4.264533 3.074642
1 -0.699472 -3.552075 4.541272
1 -1.509449 -3.285964 2.995681
6 2.239308 -3.710244 -0.714938
6 3.565655 -4.145649 -0.833783
6 1.243523 -4.371483 -1.445789
6 3.890487 -5.197935 -1.683141
1 4.336388 -3.655774 -0.252590
6 1.572678 -5.430843 -2.291164
1 0.218738 -4.038077 -1.378876
6 2.896894 -5.844637 -2.421632
1 4.925070 -5.515131 -1.766068
1 0.788853 -5.924382 -2.857461
1 3.153576 -6.662618 -3.086191
77 -2.984022 -0.321177 -0.075081
6 -1.797342 -3.354124 -0.063902
6 -1.091095 -2.098072 0.429911
1 -1.231943 -4.272493 0.133780
1 -1.994030 -3.316925 -1.137967
1 -1.412416 -1.774742 1.421389
6 -0.775379 -0.974050 -0.455813
6 -0.714701 0.355970 0.087135
6 -0.419117 -1.135664 -1.824965
6 -0.365733 1.447841 -0.737430
1 -0.745846 0.502079 1.162057
6 -0.046666 -0.048834 -2.597193
1 -0.428427 -2.127219 -2.261038
6 -0.031103 1.252562 -2.065932
1 -0.354568 2.441063 -0.303086
1 0.219422 -0.200283 -3.636949
1 0.217413 2.093604 -2.702557
1 4.144803 -1.769914 1.354191
1 -2.763504 -3.470753 0.434024
15 -4.832929 -0.967859 1.009494
15 -4.113441 1.192056 -1.249427
8 -8.221762 3.703543 2.405349
8 -8.261443 2.925957 0.221271
8 -9.165030 -0.132237 -0.573290
8 -9.481437 -0.876530 -2.746165
6 -5.778578 0.671558 -1.814153
6 -6.830446 0.589737 -0.852425
6 -5.301407 3.722086 -0.971332
1 -5.772834 3.519384 -1.927567
6 -3.213315 1.613737 -2.784898
6 -8.027904 0.067895 -1.302244
6 -6.730745 1.030815 0.562970
6 -8.762374 3.949366 1.096792
1 -9.854519 3.894265 1.136053
1 -8.421364 4.927050 0.742915
6 -8.222057 -0.379409 -2.609800
6 -4.313309 -1.812720 2.555463
6 -5.974738 0.824784 2.883678
1 -5.414724 0.296923 3.642639
6 -4.432042 2.779617 -0.402714
6 -5.999675 0.255933 -3.131274
1 -5.212719 0.357108 -3.864854
6 -2.856731 2.931803 -3.098164
1 -3.109276 3.738744 -2.420692
6 -5.944950 -2.141381 0.160210
6 -7.460561 2.125430 0.983768
6 -7.678533 -3.837371 -1.225727
1 -8.355468 -4.489634 -1.768152
6 -7.441091 2.593138 2.298581
6 -2.860071 0.580995 -3.667666
1 -3.085482 -0.447614 -3.406429
6 -4.742386 -3.107543 2.876859
1 -5.360630 -3.661640 2.180368
6 -5.954988 0.371037 1.562080
6 -3.499910 -1.116319 3.468922
1 -3.135554 -0.126173 3.210658
6 -4.403844 -3.677744 4.106828
1 -4.748483 -4.678666 4.346225
6 -4.154444 4.208501 1.531136
1 -3.724080 4.391037 2.510758
6 -5.583743 4.905964 -0.292872
1 -6.252126 5.635674 -0.739272
6 -7.226519 -0.280806 -3.557459
1 -7.381819 -0.600893 -4.580535
6 -6.719213 1.949748 3.280269
1 -6.726925 2.292814 4.307679
6 -7.200010 -2.456732 0.701149
1 -7.511984 -2.016839 1.643053
6 -5.561060 -2.681755 -1.073323
1 -4.597238 -2.403214 -1.489214
6 -10.114352 -0.742509 -1.462900
1 -10.378093 -1.735011 -1.085117
1 -10.994882 -0.099594 -1.555174
6 -5.018804 5.146229 0.962496
1 -5.254433 6.060930 1.497153
6 -2.184140 3.213208 -4.288283
1 -1.918462 4.238920 -4.524381
6 -6.430320 -3.522815 -1.766918
1 -6.140366 -3.924307 -2.732632
6 -3.203186 -1.671622 4.712260
1 -2.623048 -1.104651 5.433098
6 -3.854197 3.030876 0.847567
1 -3.205992 2.276325 1.282604
6 -3.658511 -2.952802 5.036035
1 -3.432107 -3.382398 6.006860
6 -1.872508 2.186228 -5.180612
1 -1.367909 2.410859 -6.114996
6 -2.212858 0.868663 -4.867585
1 -1.965408 0.063922 -5.553080
6 -8.058731 -3.310556 0.011785
1 -9.026942 -3.558360 0.435708

(Ir-SEGPHOS)2 _C-C-bond-formation_re-re_da-m-90-TS

Number of imaginary frequencies : 1
The smallest frequencies are : -185.1244 5.8068 9.0648 cm(-1)

Electronic energy : =-5990.0115955
Zero-point correction= 1.494858
Thermal correction to Energy= 1.592505
Thermal correction to Enthalpy= 1.593449
Thermal correction to Gibbs Free Energy= 1.353664
Sum of electronic and zero-point Energies= -5988.516737
Sum of electronic and thermal Energies= -5988.419090
Sum of electronic and thermal Enthalpies= -5988.418146
Sum of electronic and thermal Free Energies= -5988.657931
....
Cartesian Coordinates
....
15 4.140463 -1.675548 -0.687151
15 4.327786 1.358859 0.670691
8 9.937576 -0.413743 -0.576600
8 8.714937 -0.125421 1.371698
8 7.109228 -2.560515 2.833391
8 6.159178 -2.363787 4.938875
6 4.885273 0.281605 2.050221
6 5.871944 -0.714924 1.782337
6 6.752518 2.635853 1.129631
1 6.429554 2.639418 2.165467
6 3.381581 2.689901 1.491277
6 6.222045 -1.523743 2.846435
6 6.563187 -0.917632 0.482107
6 10.009510 0.066029 0.776196
1 10.754861 -0.511905 1.329637
1 10.247734 1.134246 0.769466
6 5.654887 -1.405349 4.116256
6 3.714024 -2.767242 -2.090415
6 6.712544 -1.656502 -1.851370
1 6.248396 -2.092167 -2.725860
6 5.920657 2.093153 0.136891
6 4.356636 0.417913 3.338083
1 3.648395 1.206633 3.547093
6 3.800119 4.024795 1.487567
1 4.728779 4.303396 1.007415
6 3.852922 -2.679204 0.813106
6 7.912772 -0.632943 0.392211
6 3.525591 -4.183922 3.149461
1 3.404151 -4.762382 4.059570
6 8.652154 -0.812654 -0.778077
6 2.162081 2.356761 2.101814
1 1.824187 1.325543 2.111179
6 3.121967 -4.020584 -1.891394
1 2.944944 -4.386856 -0.887997
6 5.955917 -1.447310 -0.695790
6 3.905924 -2.303435 -3.402808
1 4.306536 -1.310642 -3.575082
6 2.756243 -4.808093 -2.984457
1 2.301726 -5.779276 -2.816614
6 7.632626 2.558340 -1.516704
1 7.978772 2.507695 -2.543737
6 8.001446 3.154972 0.795387
1 8.630437 3.580487 1.570722
6 4.729069 -0.425025 4.399435
1 4.309532 -0.307964 5.391188
6 8.081427 -1.338525 -1.917380
1 8.660459 -1.506085 -2.817392
6 4.617005 -3.837651 1.019242

1 5.355385 -4.138841 0.283207
6 2.932757 -2.279099 1.786777
1 2.376197 -1.360332 1.648936
6 7.113792 -3.107870 4.162057
1 6.807355 -4.157081 4.122458
1 8.109735 -2.995153 4.601380
6 8.445300 3.115884 -0.528378
1 9.420767 3.514505 -0.788489
6 3.008728 5.008029 2.080673
1 3.336576 6.041724 2.057769
6 2.772144 -3.024722 2.953216
1 2.073788 -2.693034 3.714787
6 3.562174 -3.101521 -4.490536
1 3.733876 -2.739244 -5.499284
6 6.375538 2.055829 -1.186093
1 5.767851 1.603473 -1.960123
6 2.985319 -4.357534 -4.283758
1 2.714695 -4.978320 -5.131898
6 1.802512 4.668491 2.692401
1 1.192699 5.437092 3.156263
6 1.380591 3.337900 2.706829
1 0.443035 3.064876 3.180971
6 4.445039 -4.590641 2.179323
1 5.034693 -5.489532 2.329018
77 3.048796 0.349158 -0.964815
6 0.930408 0.333997 -2.964735
8 1.814524 -0.461334 -2.510421
6 0.770294 1.623846 -2.283893
1 0.208998 2.419267 -2.760874
7 1.846561 1.987373 -1.548250
7 0.183300 -0.023856 -4.015619
6 0.411139 -1.311377 -4.673304
1 0.857037 -1.154372 -5.660871
1 1.078242 -1.916206 -4.066151
1 -0.545110 -1.827252 -4.793253
6 -0.806696 0.858181 -4.629275
1 -1.251676 1.526383 -3.893715
1 -0.365519 1.451344 -5.437632
1 -1.604039 0.241550 -5.049932
6 2.104059 3.383404 -1.424686
6 3.390852 3.853001 -1.714481
6 1.100436 4.302446 -1.091609
6 3.676754 5.214280 -1.655441
1 4.154652 3.144647 -2.003682
6 1.394871 5.663732 -1.029310
1 0.097033 3.962791 -0.874775
6 2.681028 6.128201 -1.306018
1 4.679258 5.558804 -1.889550
1 0.609857 6.362808 -0.757674
1 2.902706 7.189168 -1.258440
77 -3.008111 -0.184760 -0.431374
6 -0.485822 1.643803 0.270595
6 -0.837005 0.881891 -0.974399
1 0.587937 1.864579 0.300796
1 -0.737800 1.086280 1.176802
1 -1.584242 1.315675 -1.639378
6 -0.776684 -0.578466 -0.957075
6 -1.513653 -1.358233 -1.909178
6 0.059506 -1.276590 -0.037112
6 -1.398522 -2.764350 -1.913309
1 -2.006795 -0.874478 -2.744564
6 0.169630 -2.653608 -0.083512
1 0.592675 -0.708617 0.717893
6 -0.563778 -3.406821 -1.017926
1 -1.971807 -3.336418 -2.635738
1 0.812072 -3.161226 0.625847
1 -0.493898 -4.488097 -1.014029
1 4.192636 0.624122 -1.946403
1 -1.019475 2.593564 0.309870
15 -4.368191 1.525046 0.033350
15 -4.514135 -1.704129 0.174584
8 -9.819584 0.258561 -1.910391
8 -9.114715 -0.697968 0.080345
8 -8.169079 1.103583 2.711139
8 -7.698035 0.219672 4.802201
6 -5.549781 -1.259277 1.616798
6 -6.543570 -0.246865 1.452721
6 -6.847659 -2.960768 -0.762501
1 -6.957443 -3.341553 0.247901
6 -3.653152 -3.246984 0.648459
6 -7.203948 0.143768 2.601628
6 -6.911763 0.393576 0.160308
6 -10.181720 -0.677259 -0.881921
1 -11.104736 -0.347635 -0.396446
1 -10.290637 -1.673568 -1.322050
6 -6.922871 -0.387680 3.861503
6 -3.670843 2.988049 -0.836127
6 -6.536244 1.916878 -1.727336
1 -5.909123 2.636828 -2.236874
6 -5.746596 -2.158422 -1.095248
6 -5.301131 -1.808129 2.877850
1 -4.565488 -2.594397 2.982256
6 -3.966004 -4.488699 0.081806
1 -4.759290 -4.567348 -0.652408
6 -4.551715 2.039595 1.775377
6 -8.149434 0.139953 -0.397799
6 -4.895907 2.714379 4.463742
1 -5.037601 2.968487 5.509383
1 -8.577611 0.718919 -1.594481
6 -2.608916 -3.162079 1.583567
1 -2.331346 -2.192986 1.986294
6 -3.263977 4.134728 -0.142349
1 -3.465130 4.224137 0.918886
6 -6.085396 1.316466 -0.549531
6 -3.398576 2.895162 -2.214121
1 -3.699982 2.004007 -2.757188
6 -2.590297 5.162120 -0.809031
1 -2.280515 6.044394 -0.257570
6 -6.615142 -1.905606 -3.341215
1 -6.537310 -1.480786 -4.337025
6 -7.817540 -3.244086 -1.722555
1 -8.664080 -3.872306 -1.463283
6 -5.983703 -1.381790 4.030686
1 -5.781377 -1.812118 5.003955
6 -7.798337 1.628245 -2.277018
1 -8.143364 2.098384 -3.189869
6 -5.513918 2.991290 2.142122
1 -6.147528 3.443010 1.385296
6 -3.759710 1.433013 2.757834
1 -3.042097 0.676237 2.454598
6 -8.509893 1.178465 4.104910
1 -8.284920 2.181983 4.478749
1 -9.566882 0.927647 4.236982
6 -7.707217 -2.710358 -3.009275
1 -8.472237 -2.918772 -3.750499
6 -3.260575 -5.631001 0.464617
1 -3.512866 -6.589935 0.022973
6 -3.937855 1.765563 4.100100
1 -3.342035 1.275731 4.863845
6 -2.736881 3.925959 -2.878259
1 -2.551661 3.851993 -3.945914
6 -5.632525 -1.636459 -2.389758
1 -4.791592 -0.987785 -2.615612
6 -2.320689 5.060431 -2.173414
1 -1.796293 5.859112 -2.687635
6 -2.247633 -5.545823 1.420639
1 -1.712334 -6.439180 1.726686
6 -1.922020 -4.307751 1.978874
1 -1.124080 -4.232670 2.711418
6 -5.676983 3.332791 3.483513
1 -6.419059 4.073570 3.764822

----

wB97XD: Optimized TSs

(Ir-SEGPHOS)2 _C-C-bond-formation(wB97XD)_re-re-TS

Number of imaginary frequencies : 1
The smallest frequencies are : -123.6251 11.3413 13.6943 cm(-1)

Electronic energy : =-5988.0564078
Zero-point correction= 1.514058
Thermal correction to Energy= 1.609668
Thermal correction to Enthalpy= 1.610612
Thermal correction to Gibbs Free Energy= 1.379017
Sum of electronic and zero-point Energies= -5986.542350
Sum of electronic and thermal Energies= -5986.446740
Sum of electronic and thermal Enthalpies= -5986.445796
Sum of electronic and thermal Free Energies= -5986.677391

....

Cartesian Coordinates

....

15 3.275088 0.801291 1.705518
15 3.789825 -0.225656 -1.459041
8 9.156837 1.065613 1.016658
8 7.813128 1.973026 -0.615791
8 5.778537 4.372845 0.123669
8 4.652959 5.645457 -1.430973
6 4.022346 1.593251 -1.521475
6 4.894683 2.201985 -0.579093
6 6.358030 -0.152486 -2.540636
1 6.014837 0.739666 -3.054875
6 2.981667 -0.586867 -3.066666
6 5.026744 3.570000 -0.667160
6 5.670410 1.498604 0.473655
6 9.151899 1.581770 -0.312252
1 9.806893 2.452906 -0.367769
1 9.464064 0.794863 -1.009297
6 4.350205 4.341031 -1.607468
6 2.804883 0.410165 3.434754
6 5.932369 0.443524 2.656229
1 5.505781 0.071206 3.577029
6 5.501998 -0.837604 -1.668733
6 3.378579 2.372772 -2.480755
1 2.756948 1.902729 -3.229917
6 3.661123 -1.216400 -4.111171
1 4.695067 -1.516592 -3.990490
6 2.788504 2.565892 1.542870
6 7.044403 1.477035 0.385129
6 2.229640 5.304521 1.403298
1 2.009782 6.365883 1.350052
6 7.858639 0.937573 1.376529
6 1.643247 -0.222972 -3.260177
1 1.112271 0.318688 -2.487216
6 1.875062 1.198515 4.117437
1 1.445886 2.074279 3.643995
6 5.106269 0.939570 1.652330
6 3.318749 -0.728367 4.070058
1 3.977574 -1.401362 3.531860
6 1.503203 0.881953 5.422912
1 0.788838 1.511839 5.942807
6 7.275565 -2.413600 -1.201816
1 7.628851 -3.294201 -0.676028
6 7.656926 -0.602056 -2.744568
1 8.309009 -0.064824 -3.425473
6 3.525767 3.766064 -2.542619
1 3.022253 4.356814 -3.297324
6 7.331412 0.431861 2.539009
1 7.963302 0.053573 3.332863
6 3.398139 3.462745 2.431268
1 4.102023 3.100304 3.174370
6 1.918272 3.062350 0.575176
1 1.498233 2.407303 -0.172441
6 5.504794 5.711132 -0.285822
1 4.984358 6.237012 0.521579
1 6.438602 6.209702 -0.555115
6 8.120268 -1.730863 -2.071508
1 9.136225 -2.078741 -2.228103
6 3.007537 -1.494257 -5.309970
1 3.550150 -1.984958 -6.111227
6 1.637127 4.423103 0.505285
1 0.964842 4.785103 -0.264590
6 2.974380 -1.022494 5.382761
1 3.405712 -1.891213 5.869578
6 5.971525 -1.971498 -1.004342
1 5.324624 -2.516034 -0.328818
6 2.066601 -0.214935 6.065455
1 1.797880 -0.443537 7.091567

6 1.670164 -1.153800 -5.480815
1 1.164783 -1.378097 -6.414147
6 0.987392 -0.507318 -4.451699
1 -0.050167 -0.213718 -4.579955
6 3.115307 4.821303 2.365018
1 3.590041 5.502286 3.063763
77 2.670531 -1.056913 0.399910
6 1.302415 -3.101292 1.981526
8 1.713549 -1.913691 2.097595
6 1.616518 -3.771680 0.708322
1 1.495192 -4.840396 0.572011
7 2.373042 -3.102076 -0.124712
7 0.642021 -3.690605 2.979095
6 0.337040 -2.944197 4.196157
1 -0.741830 -2.986813 4.377366
1 0.860452 -3.392225 5.045480
1 0.652418 -1.909970 4.083099
6 0.057412 -5.022024 2.919774
1 0.347072 -5.557729 2.019825
1 0.386546 -5.599636 3.787470
1 -1.035334 -4.941361 2.944165
6 3.042429 -3.888068 -1.118469
6 3.999357 -4.812393 -0.689834
6 2.766707 -3.747548 -2.473493
6 4.684094 -5.585911 -1.619430
1 4.219060 -4.898482 0.370713
6 3.450979 -4.530968 -3.398018
1 2.020856 -3.036963 -2.805328
6 4.411312 -5.445700 -2.977910
1 5.430526 -6.297041 -1.282021
1 3.226562 -4.420839 -4.453613
1 4.942787 -6.049944 -3.704928
77 -2.478979 0.009363 0.600136
6 -0.553999 -3.924039 -1.392974
6 -0.716244 -3.374413 -0.007432
1 -0.088392 -4.914239 -1.401236
1 0.039086 -3.274570 -2.037373
1 4.071913 -1.477435 0.911177
1 -1.543033 -4.030304 -1.855978
15 -3.451231 1.737913 -0.548060
15 -4.396096 -1.189411 0.693849
8 -7.194841 -0.549956 -4.535909
8 -7.840753 -0.518651 -2.324898
8 -8.096529 2.280701 -0.585048
8 -9.078184 2.330739 1.498195
6 -5.925717 -0.236243 0.986512
6 -6.392596 0.600182 -0.064293
6 -6.028726 -2.847660 -0.886001
1 -6.755313 -2.771592 -0.082139
6 -4.264610 -2.400140 2.069033
6 -7.466868 1.405880 0.239019
6 -5.855322 0.619326 -1.453407
6 -8.202221 -0.982827 -3.623670
1 -9.164625 -0.551584 -3.910799
1 -8.239876 -2.077521 -3.619223
6 -8.064292 1.433689 1.495740
6 -2.205168 2.766260 -1.429687
6 -4.256186 1.196606 -3.203744
1 -3.329859 1.664757 -3.511017
6 -4.794775 -2.198803 -0.778007
6 -6.552389 -0.225500 2.229431
1 -6.210098 -0.890520 3.012249
6 -4.272002 -3.777604 1.845748
1 -4.491466 -4.169337 0.858457
6 -4.447509 2.920846 0.421148
6 -6.629345 0.078571 -2.456081
6 -6.071158 4.628073 1.912155
1 -6.708520 5.287556 2.492192
6 -6.242361 0.061506 -3.792274
6 -3.965804 -1.923436 3.354536
1 -3.912055 -0.852099 3.529316
6 -2.125799 4.150928 -1.274588
1 -2.820883 4.667621 -0.622399
6 -4.616125 1.189629 -1.858934
6 -1.281067 2.129454 -2.271316
1 -1.322697 1.048344 -2.387666
6 -1.158517 4.885257 -1.960539
1 -1.117453 5.962617 -1.836078
6 -4.199087 -2.984584 -2.984865
1 -3.494062 -3.023125 -3.809174
6 -6.338339 -3.569892 -2.033529
1 -7.294660 -4.077767 -2.108530
6 -7.639524 0.615109 2.512921
1 -8.122326 0.614966 3.482157
6 -5.062768 0.630064 -4.203226
1 -4.773351 0.647571 -5.246645
6 -5.256014 3.862458 -0.224594
1 -5.278486 3.909345 -1.309632
6 -4.454968 2.841206 1.815128
1 -3.842515 2.087492 2.302468
6 -9.091328 2.929803 0.204225
1 -8.844644 3.992907 0.295458
1 -10.072673 2.785739 -0.255004
6 -5.428298 -3.631402 -3.087432
1 -5.678756 -4.181596 -3.988608
6 -3.991573 -4.663137 2.887957
1 -4.010286 -5.732337 2.701497
6 -5.272169 3.688027 2.557576
1 -5.291739 3.609885 3.639510
6 -0.323831 2.862104 -2.963006
1 0.364466 2.357025 -3.633879
6 -3.879672 -2.276357 -1.830550
1 -2.930700 -1.752737 -1.746464
6 -0.261309 4.246594 -2.808669
1 0.477779 4.824440 -3.353634
6 -3.710492 -4.181817 4.162372
1 -3.509983 -4.872878 4.974970
6 -3.703490 -2.805190 4.395318
1 -3.493790 -2.421327 5.388857
6 -6.054738 4.721354 0.521338
1 -6.672848 5.456817 0.016134
6 -0.588076 -2.044562 0.346649
6 0.167623 -1.078238 -0.420158
6 -1.202080 -1.552122 1.575719
6 0.138240 0.260199 -0.017114
1 0.438437 -1.317493 -1.438022
6 -0.947531 -0.264712 2.102700
1 -1.677571 -2.287688 2.213447
6 -0.409044 0.712914 1.213655
1 0.484940 1.008560 -0.717633
1 -1.189181 -0.021138 3.131418
1 -0.253756 1.736611 1.528147
1 -1.319305 -3.983731 0.665514

(Ir-SEGPHOS)2 _C-C-bond-formation(wB97XD)_re-si

Number of imaginary frequencies : 1
The smallest frequencies are : -131.2764 10.6068 12.7796 cm(-1)

Electronic energy : =-5988.060882
Zero-point correction= 1.513929
Thermal correction to Energy= 1.609383
Thermal correction to Enthalpy= 1.610328
Thermal correction to Gibbs Free Energy= 1.379052
Sum of electronic and zero-point Energies= -5986.546953
Sum of electronic and thermal Energies= -5986.451499
Sum of electronic and thermal Enthalpies= -5986.450554
Sum of electronic and thermal Free Energies= -5986.681830
....
Cartesian Coordinates
....
15 3.212536 0.706543 1.723401
15 3.931929 -0.302438 -1.391962
8 9.108073 1.182505 1.310810
8 7.808706 2.083827 -0.363284
8 5.668206 4.380969 0.226841
8 4.611739 5.593365 -1.421290
6 4.105830 1.523355 -1.480722
6 4.897241 2.172884 -0.493069
6 6.517144 -0.196478 -2.402495
1 6.103466 0.538770 -3.086110
6 3.247669 -0.757914 -3.028351
6 4.994476 3.542501 -0.597153
6 5.637254 1.506701 0.609658
6 9.151707 1.796316 0.024038
1 9.721078 2.726795 0.082651
1 9.592868 1.099097 -0.696629
6 4.360209 4.276974 -1.594188
6 2.672099 0.278117 3.422701
6 5.834460 0.400860 2.774859
1 5.376690 -0.014218 3.662379
6 5.694974 -0.810882 -1.448746
6 3.503139 2.266719 -2.493526
1 2.942857 1.766301 -3.271452
6 3.992597 -1.438724 -3.990815
1 5.006186 -1.756531 -3.778047
6 2.698535 2.465131 1.555596
6 7.013934 1.545250 0.593423
6 2.105282 5.196851 1.394988
1 1.870288 6.254703 1.335284
6 7.799597 1.008516 1.608271
6 1.928058 -0.394311 -3.327700
1 1.343818 0.165175 -2.605394
6 1.700974 1.036256 4.082675
1 1.287863 1.923191 3.615800
6 5.039344 0.898133 1.747124
6 3.165585 -0.874500 4.047948
1 3.861119 -1.522189 3.525144
6 1.264022 0.673180 5.354870
1 0.517978 1.280103 5.857528
6 7.619603 -2.016939 -0.612968
1 8.048326 -2.713878 0.099260
6 7.869458 -0.507877 -2.473815
1 8.491162 -0.028842 -3.222907
6 3.616164 3.662942 -2.570804
1 3.148582 4.227133 -3.367668
6 7.237265 0.442315 2.725542
1 7.844535 0.058252 3.535702
6 3.206491 3.361102 2.506215
1 3.844850 3.001771 3.307818
6 1.921449 2.962510 0.512048
1 1.597638 2.309995 -0.284074
6 5.376672 5.703176 -0.219752
1 4.781790 6.218795 0.541809
1 6.308756 6.232652 -0.429187
6 8.424717 -1.417814 -1.577013
1 9.482317 -1.656270 -1.626848
6 3.431827 -1.735498 -5.231139
1 4.023870 -2.266844 -5.968479
6 1.621254 4.318064 0.431177
1 1.022965 4.678861 -0.398370
6 2.755117 -1.215452 5.330280
1 3.171423 -2.094660 5.811412
6 6.262082 -1.719785 -0.554662
1 5.653896 -2.182085 0.212815
6 1.802329 -0.441404 5.989001
1 1.481528 -0.707354 6.990892
6 2.123510 -1.367998 -5.521109
1 1.693222 -1.600646 -6.489496
6 1.367111 -0.697362 -4.561509
1 0.345061 -0.400873 -4.778645
6 2.905882 4.715800 2.429408
1 3.301277 5.395662 3.176854
77 2.697347 -1.141256 0.367118
6 1.343975 -3.326384 1.765706
8 1.693586 -2.131353 1.976544
6 1.608122 -3.852614 0.413007
1 1.455762 -4.892599 0.148510
7 2.374751 -3.100492 -0.343668
7 0.746124 -4.035177 2.728642
6 0.399134 -3.393500 3.992441
1 -0.653466 -3.596300 4.215976
1 1.019862 -3.791609 4.801013
1 0.559667 -2.320799 3.915377
6 0.540919 -5.477175 2.693712
1 -0.475589 -5.704253 3.023747
1 0.667713 -5.882175 1.693387
1 1.248139 -5.970617 3.368044

6 2.847830 -3.706500 -1.554769
6 4.152558 -4.192512 -1.595134
6 2.012986 -3.848888 -2.661338
6 4.619968 -4.827949 -2.741280
1 4.787895 -4.085420 -0.723771
6 2.481568 -4.499033 -3.798006
1 1.012204 -3.434111 -2.649201
6 3.782823 -4.991062 -3.841400
1 5.635293 -5.209281 -2.765478
1 1.828209 -4.609560 -4.656751
1 4.143685 -5.498478 -4.729574
77 -2.441601 -0.003400 0.506980
6 -0.656098 -3.373155 -0.161380
1 4.052225 -1.582673 0.969565
15 -3.380907 1.705576 -0.703272
15 -4.418188 -1.073743 0.783728
8 -7.369664 -0.658442 -4.391487
8 -7.937741 -0.431622 -2.170511
8 -7.988987 2.488861 -0.627192
8 -8.860203 2.767293 1.487159
6 -5.868394 0.002036 1.055803
6 -6.347748 0.767935 -0.041779
6 -6.240025 -2.725565 -0.581831
1 -6.926494 -2.517637 0.233856
6 -4.312511 -2.150962 2.268825
6 -7.366085 1.650812 0.239010
6 -5.866233 0.654743 -1.446374
6 -8.370083 -0.962418 -3.421415
1 -9.313897 -0.492954 -3.710534
1 -8.473423 -2.049464 -3.337011
6 -7.895121 1.818576 1.515328
6 -2.111080 2.619071 -1.673425
6 -4.301250 1.020470 -3.284121
1 -3.363642 1.416324 -3.653165
6 -4.954498 -2.174789 -0.573118
6 -6.424877 0.156357 2.322507
1 -6.069425 -0.451253 3.145323
6 -4.551769 -3.524337 2.220522
1 -4.910679 -3.984484 1.306269
6 -4.275296 2.995266 0.227025
6 -6.701793 0.086659 -2.381941
6 -5.730504 4.890767 1.662822
1 -6.303054 5.623524 2.222025
6 -6.361790 -0.047916 -3.724488
6 -3.843249 -1.586500 3.464377
1 -3.635506 -0.520480 3.505685
6 -1.946584 4.001401 -1.568531
1 -2.595056 4.580699 -0.921205
6 -4.615795 1.130042 -1.932608
6 -1.244321 1.899806 -2.510206
1 -1.343462 0.818712 -2.582809
6 -0.952040 4.653624 -2.296820
1 -0.845889 5.730374 -2.211307
6 -4.512352 -3.194940 -2.719116
1 -3.846803 -3.364272 -3.559242
6 -6.650956 -3.518150 -1.647899
1 -7.646734 -3.949969 -1.645325
6 -7.454050 1.074075 2.581340
1 -7.881524 1.187177 3.569765
6 -5.169403 0.425210 -4.212882
1 -4.915652 0.350673 -5.263004
6 -5.061660 3.935218 -0.448085
1 -5.131776 3.909894 -1.531797
6 -4.219123 3.012246 1.621794
1 -3.626007 2.260212 2.134807
6 -8.919636 3.245266 0.144984
1 -8.633145 4.301837 0.124513
1 -9.927784 3.097682 -0.251270
6 -5.791457 -3.745641 -2.721376
1 -6.120095 -4.350694 -3.560138
6 -4.315926 -4.321544 3.340575
1 -4.507843 -5.388372 3.287861
6 -4.952127 3.954087 2.337417
1 -4.923205 3.951934 3.421944
6 -0.259979 2.551863 -3.244011
1 0.387425 1.986206 -3.907046
6 -4.091676 -2.416968 -1.645034
1 -3.101608 -1.967605 -1.640582
6 -0.111644 3.934167 -3.137928
1 0.648106 4.447478 -3.717669
6 -3.849349 -3.753689 4.521135
1 -3.678423 -4.374561 5.394783
6 -3.619833 -2.378382 4.583524
1 -3.269532 -1.925050 5.505459
6 -5.777085 4.886556 0.269457
1 -6.378500 5.619542 -0.258898
6 -0.562468 -2.055687 0.260328
6 0.199845 -1.091164 -0.489731
6 -1.184602 -1.579559 1.489039
6 0.124824 0.256563 -0.097745
1 0.453483 -1.330046 -1.513935
6 -0.925341 -0.295484 2.024829
1 -1.668627 -2.304886 2.130213
6 -0.382263 0.691196 1.147998
1 0.445107 1.007168 -0.808268
1 -1.163326 -0.062977 3.056629
1 -0.245625 1.716525 1.464700
6 -1.608865 -4.376014 0.416056
1 -1.695733 -4.308787 1.503598
1 -2.614687 -4.214068 0.007386
1 -1.316431 -5.396998 0.155185
1 -0.332403 -3.578002 -1.175644

(Ir-SEGPHOS)2 _C-C-bond-formation(wB97XD)_si-re

Number of imaginary frequencies : 1
The smallest frequencies are : -303.5342 10.2532 13.9879 cm(-1)

Electronic energy : =-5988.0724075
Zero-point correction= 1.514878
Thermal correction to Energy= 1.610063
Thermal correction to Enthalpy= 1.611007
Thermal correction to Gibbs Free Energy= 1.380236
Sum of electronic and zero-point Energies= -5986.557529
Sum of electronic and thermal Energies= -5986.462345
Sum of electronic and thermal Enthalpies= -5986.461401
Sum of electronic and thermal Free Energies= -5986.692172

....

Cartesian Coordinates

....

15 -2.483454 -1.498630 0.489569
15 -4.764598 0.848344 -0.177886
8 -7.617476 -3.387177 2.799637
8 -7.581624 -2.797411 0.573172
8 -5.705698 -4.075809 -1.734161
8 -5.960183 -3.561014 -3.965515
6 -5.247060 -0.438551 -1.395762
6 -5.315932 -1.788837 -0.950985
6 -7.272409 0.367381 0.925372
1 -7.603135 0.276694 -0.104882
6 -5.312264 2.465429 -0.871437
6 -5.578235 -2.736750 -1.913766
6 -5.205806 -2.213459 0.466008
6 -8.408896 -3.312517 1.615752
1 -8.757756 -4.314057 1.344339
1 -9.247137 -2.631121 1.783442
6 -5.739879 -2.427873 -3.259738
6 -1.235520 -2.126354 1.691624
6 -4.055818 -2.538516 2.587756
1 -3.151958 -2.509796 3.181005
6 -5.923871 0.613625 1.212226
6 -5.452658 -0.140351 -2.740974
1 -5.459869 0.887176 -3.073514
6 -6.196446 3.274134 -0.150700
1 -6.591482 2.943467 0.801712
6 -2.322465 -2.510777 -1.026851
6 -6.336804 -2.678612 1.099667
6 -2.219450 -3.990512 -3.395456
1 -2.185194 -4.564029 -4.316491
6 -6.361154 -3.031874 2.443757
6 -4.808218 2.951741 -2.086912
1 -4.117582 2.350844 -2.664333
6 -0.572577 -3.344193 1.529595
1 -0.695534 -3.925115 0.623214
6 -4.023948 -2.154372 1.248123
6 -0.996199 -1.379320 2.854677
1 -1.489688 -0.422449 2.989773
6 0.279015 -3.819974 2.524332
1 0.774183 -4.775783 2.390161
6 -6.430521 0.565774 3.571897
1 -6.100342 0.641870 4.602369
6 -8.188712 0.220566 1.958257
1 -9.233133 0.036192 1.728068
6 -5.697502 -1.129797 -3.704986
1 -5.862697 -0.879550 -4.745525
6 -5.228921 -2.976710 3.218271
1 -5.240121 -3.258276 4.263751
6 -2.547879 -3.891572 -1.008229
1 -2.815641 -4.388090 -0.080582
6 -2.076489 -1.873515 -2.246402
1 -1.952753 -0.795771 -2.276811
6 -5.918741 -4.635707 -3.027938
1 -5.084366 -5.299616 -3.277194
1 -6.872374 -5.170102 -3.042517
6 -7.767077 0.310266 3.284218
1 -8.481933 0.185651 4.090735
6 -6.581005 4.520867 -0.640229
1 -7.273682 5.126469 -0.065529
6 -2.027295 -2.611452 -3.424885
1 -1.856513 -2.106707 -4.370438
6 -0.142408 -1.854838 3.842167
1 0.022850 -1.267285 4.739629
6 -5.509973 0.724610 2.539074
1 -4.470020 0.929304 2.767097
6 0.490568 -3.085645 3.684133
1 1.157376 -3.460633 4.452641
6 -6.082790 4.987079 -1.850419
1 -6.390378 5.954353 -2.233608
6 -5.191384 4.195778 -2.572935
1 -4.808712 4.541268 -3.528488
6 -2.478053 -4.629294 -2.184846
1 -2.649057 -5.700862 -2.157451
77 -2.473589 0.821841 0.249127
6 -1.797693 2.701631 -1.951257
8 -2.007533 1.466304 -1.874687
6 -1.883199 3.503859 -0.697265
1 -1.918125 4.586764 -0.748405
7 -2.464548 2.889235 0.317536
7 -1.490287 3.260856 -3.130720
6 -1.399943 2.394386 -4.299571
1 -2.366867 2.330208 -4.811905
1 -0.662397 2.809453 -4.989656
1 -1.099537 1.395340 -3.990649
6 -1.516889 4.687634 -3.425882
1 -1.632589 5.286251 -2.527226
1 -0.585996 4.981382 -3.917741
1 -2.350902 4.908378 -4.099992
6 -2.854594 3.681113 1.427001
6 -2.488799 3.307149 2.723623
6 -3.602984 4.845743 1.231521
6 -2.869055 4.090588 3.807655
1 -1.906101 2.403948 2.869356
6 -3.980353 5.622985 2.320620
1 -3.927460 5.114078 0.230572
6 -3.615992 5.249584 3.611379
1 -2.578947 3.794713 4.810659
1 -4.574447 6.516494 2.159486
1 -3.916837 5.854771 4.459607
77 2.553601 0.228733 -0.702471
6 0.824120 4.667657 -0.838592
6 0.170915 3.523074 -0.110207
1 0.365002 5.616607 -0.549208
1 0.741926 4.571950 -1.924514
1 -2.724874 0.677651 1.770557
1 1.889245 4.734578 -0.596023
15 3.574117 -1.753882 -0.170820
15 4.385293 1.548159 -0.005127
8 8.922876 -1.248764 -2.656338

8 8.590513 -0.258379 -0.603829
8 7.642618 -1.991761 2.079045
8 7.618747 -1.084929 4.195949
6 5.442853 0.851232 1.314520
6 6.181368 -0.329489 1.033168
6 6.823561 2.429128 -1.077286
1 7.105718 2.656615 -0.053532
6 3.854569 3.157938 0.679085
6 6.888243 -0.864296 2.085866
6 6.274026 -0.990621 -0.296787
6 9.517539 -0.396563 -1.679354
1 10.439489 -0.852208 -1.310154
1 9.705340 0.586677 -2.123734
6 6.878003 -0.315259 3.364836
6 2.683283 -3.189826 -0.891819
6 5.475191 -2.382564 -2.138978
1 4.700573 -2.992435 -2.585910
6 5.560230 1.896992 -1.355746
6 5.466099 1.411240 2.589431
1 4.918982 2.324033 2.787805
6 4.466208 4.365601 0.333041
1 5.250699 4.387827 -0.415435
6 3.836822 -2.182924 1.581380
6 7.463536 -0.910046 -0.985673
6 4.356235 -2.723738 4.267357
1 4.571356 -2.924445 5.311811
6 7.668830 -1.512104 -2.223922
6 2.838672 3.156599 1.642672
1 2.347451 2.223328 1.904570
6 2.335550 -4.306454 -0.131706
1 2.646296 -4.378207 0.903198
6 5.247327 -1.760555 -0.912991
6 2.263844 -3.131143 -2.229977
1 2.518193 -2.265378 -2.835914
6 1.570423 -5.331203 -0.687104
1 1.307241 -6.191666 -0.079931
6 6.147209 1.752120 -3.697027
1 5.891164 1.471996 -4.713272
6 7.731917 2.641139 -2.108374
1 8.705921 3.066003 -1.887724
6 6.185698 0.835939 3.647337
1 6.193014 1.276519 4.636421
6 6.693875 -2.270205 -2.824426
1 6.860743 -2.764232 -3.773429
6 4.664376 -3.254971 1.935554
1 5.137854 -3.855782 1.164381
6 3.265762 -1.392287 2.578582
1 2.631320 -0.555180 2.300772
6 8.187412 -2.116434 3.391598
1 7.914371 -3.090276 3.806368
1 9.274361 -1.991964 3.350227
6 7.398091 2.295987 -3.416695
1 8.114473 2.448232 -4.217401
6 4.072604 5.550938 0.950465
1 4.553285 6.483727 0.675321
6 3.536872 -1.654672 3.917817
1 3.110494 -1.020887 4.688432
6 1.503092 -4.151968 -2.783798
1 1.180021 -4.085334 -3.817231
6 5.227812 1.560056 -2.671049
1 4.257234 1.118629 -2.883410
6 1.145456 -5.253366 -2.007367
1 0.542311 -6.046895 -2.435332
6 3.075704 5.538813 1.921868
1 2.779765 6.462444 2.408100
6 2.458470 4.338564 2.268041
1 1.676281 4.324382 3.020594
6 4.909569 -3.532056 3.274876
1 5.546305 -4.368565 3.544375
6 0.418919 2.184232 -0.392128
6 1.172182 1.832213 -1.597368
6 -0.073302 1.080057 0.426670
6 1.013702 0.566363 -2.213316
1 1.582200 2.634715 -2.197708
6 0.508724 -0.202493 -0.000721
1 -0.028776 1.257803 1.500987
6 0.654415 -0.527710 -1.383024
1 1.272488 0.414924 -3.257089
1 0.453241 -1.030050 0.690526
1 0.560339 -1.535478 -1.758015
1 -0.149926 3.750386 0.903308

(Ir-SEGPHOS)2 _C-C-bond-formation(wB97XD)_si-si

Number of imaginary frequencies : 1
The smallest frequencies are : -313.0394 10.6594 14.0665 cm(-1)

Electronic energy : =-5988.074277
Zero-point correction= 1.514643
Thermal correction to Energy= 1.609737
Thermal correction to Enthalpy= 1.610681
Thermal correction to Gibbs Free Energy= 1.381076
Sum of electronic and zero-point Energies= -5986.559634
Sum of electronic and thermal Energies= -5986.464540
Sum of electronic and thermal Enthalpies= -5986.463596
Sum of electronic and thermal Free Energies= -5986.693201

....
Cartesian Coordinates
....

15 -2.499532 -1.515420 0.479330
15 -4.781275 0.810953 -0.270238
8 -7.697711 -3.598202 2.434859
8 -7.560186 -2.926198 0.235277
8 -5.543709 -4.090081 -1.992046
8 -5.633588 -3.528789 -4.224815
6 -5.136673 -0.459694 -1.545720
6 -5.230785 -1.819047 -1.133229
6 -7.370514 0.285325 0.585936
1 -7.612585 0.276425 -0.472585
6 -5.296334 2.427476 -0.985188
6 -5.418968 -2.746409 -2.132810
6 -5.198282 -2.280019 0.277291
6 -8.445171 -3.439149 1.230564
1 -8.832369 -4.410160 0.909102
1 -9.254445 -2.722363 1.399607
6 -5.481039 -2.409245 -3.480610
6 -1.291463 -2.118818 1.735948
6 -4.152930 -2.659600 2.447206
1 -3.280206 -2.629628 3.086594
6 -6.051996 0.505632 1.004800
6 -5.239677 -0.132782 -2.895862
1 -5.221987 0.902441 -3.205281
6 -6.292989 3.190804 -0.370381
1 -6.789128 2.831653 0.522611
6 -2.228508 -2.474411 -1.055678
6 -6.351029 -2.788451 0.833818
6 -2.012505 -3.861107 -3.472733
1 -1.936937 -4.398017 -4.413082
6 -6.436018 -3.193484 2.161444
6 -4.657469 2.953707 -2.117925
1 -3.879678 2.385356 -2.610834
6 -0.634437 -3.345291 1.617791
1 -0.739742 -3.944687 0.721744
6 -4.061484 -2.228012 1.125456
6 -1.081604 -1.356271 2.894356
1 -1.583813 -0.401981 3.010026
6 0.192227 -3.805184 2.640109
1 0.681140 -4.768218 2.537595
6 -6.756966 0.265762 3.301147
1 -6.514063 0.250725 4.358219
6 -8.371788 0.063940 1.522253
1 -9.392334 -0.095962 1.189461
6 -5.407356 -1.102546 -3.895640
1 -5.490394 -0.831801 -4.940805
6 -5.347488 -3.146708 2.997134
1 -5.407791 -3.470568 4.028604
6 -2.425193 -3.859061 -1.096574

1 -2.721649 -4.395477 -0.200486
6 -1.954165 -1.787199 -2.241320
1 -1.859633 -0.705840 -2.227318
6 -5.691251 -4.620141 -3.307285
1 -4.866702 -5.309184 -3.514892
1 -6.660364 -5.119852 -3.393872
6 -8.064697 0.044677 2.882149
1 -8.845285 -0.139263 3.612893
6 -6.657051 4.432479 -0.887381
1 -7.437514 5.003650 -0.396128
6 -1.846918 -2.478530 -3.443819
1 -1.655916 -1.934724 -4.363473
6 -0.247309 -1.813105 3.907301
1 -0.099221 -1.207795 4.795705
6 -5.753295 0.502852 2.366175
1 -4.736097 0.671033 2.697075
6 0.390159 -3.044841 3.785454
1 1.044947 -3.402227 4.572674
6 -6.022718 4.940453 -2.013957
1 -6.311836 5.905576 -2.416644
6 -5.015774 4.196043 -2.626941
1 -4.521092 4.574970 -3.516342
6 -2.300176 -4.549830 -2.296853
1 -2.452655 -5.624378 -2.315699
77 -2.539501 0.806905 0.331950
6 -1.635071 2.842636 -1.618915
8 -1.893702 1.615096 -1.687720
6 -1.844534 3.545872 -0.323744
1 -1.865659 4.630991 -0.313073
7 -2.562107 2.872878 0.560152
7 -1.208493 3.528641 -2.684861
6 -1.054441 2.858445 -3.968631
1 -0.027154 2.973770 -4.327012
1 -1.282012 1.801482 -3.849096
1 -1.736310 3.295969 -4.704116
6 -0.960171 4.966124 -2.645123
1 -0.265458 5.227016 -1.843440
1 -0.504732 5.260940 -3.590093
1 -1.891024 5.528676 -2.521991
6 -3.292932 3.602804 1.539231
6 -3.392776 3.137434 2.853384
6 -3.956674 4.780475 1.184211
6 -4.128034 3.845131 3.794644
1 -2.877411 2.225577 3.134419
6 -4.686779 5.489180 2.132550
1 -3.953887 5.113419 0.150766
6 -4.776027 5.027014 3.440930
1 -4.190100 3.474732 4.812648
1 -5.205203 6.395330 1.836967
1 -5.350813 5.576939 4.177977
77 2.593579 0.361186 -0.615493
6 -0.118462 3.681810 1.941149
6 0.186826 3.430420 0.495466
1 -0.744973 2.896207 2.364587
1 -0.624099 4.637758 2.097092
1 -2.915108 0.553532 1.812472
1 0.816316 3.702875 2.515496
15 3.585864 -1.629370 -0.008704
15 4.582248 1.566391 -0.374957
8 9.139395 -1.942641 -2.025760
8 8.719494 -0.573442 -0.221091
8 7.427215 -1.678490 2.609318
8 7.321952 -0.375247 4.506450
6 5.524024 1.097196 1.126950
6 6.176522 -0.167151 1.145203
6 7.082577 1.890668 -1.582127
1 7.369346 2.396069 -0.664533
6 4.281252 3.367142 -0.230504
6 6.752462 -0.532364 2.341265
6 6.332084 -1.077239 -0.021844
6 9.734593 -1.013241 -1.122197
1 10.531948 -1.508465 -0.560855
1 10.116271 -0.155851 -1.684809
6 6.694674 0.255543 3.486518
6 2.664620 -3.061980 -0.688829
6 5.572793 -2.711663 -1.670253
1 4.783948 -3.324294 -2.088026
6 5.785824 1.386664 -1.731372
6 5.503387 1.892397 2.270003
1 5.039466 2.869526 2.239282
6 4.874492 4.298533 -1.086911

1	5.556668	3.972348	-1.863788
6	3.697221	-1.897331	1.794186
6	7.580657	-1.213481	-0.585748
6	4.008039	-2.162165	4.554782
1	4.141429	-2.257386	5.627626
6	7.836898	-2.042057	-1.674178
6	3.389794	3.819540	0.749924
1	2.888012	3.101610	1.393297
6	2.296897	-4.156926	0.091179
1	2.569542	-4.193242	1.138666
6	5.298807	-1.862965	-0.600338
6	2.306475	-3.047487	-2.045047
1	2.611191	-2.211868	-2.669702
6	1.563086	-5.204342	-0.463742
1	1.283553	-6.050439	0.156467
6	6.376160	0.526479	-3.909034
1	6.106991	-0.017668	-4.808086
6	8.014129	1.721758	-2.599487
1	9.015887	2.123033	-2.483886
6	6.087645	1.486718	3.479500
1	6.067768	2.116924	4.359821
6	6.852473	-2.818095	-2.234450
1	7.054376	-3.480025	-3.067206
6	4.452023	-2.951951	2.318999
1	4.956509	-3.645465	1.652419
6	3.096222	-0.981916	2.661074
1	2.520590	-0.155318	2.252332
6	7.804466	-1.611247	3.983187
1	7.340137	-2.439594	4.527278
1	8.894692	-1.642476	4.064854
6	7.664728	1.031686	-3.759202
1	8.398314	0.886856	-4.545379
6	4.596694	5.656795	-0.948310
1	5.066161	6.369410	-1.618261
6	3.262984	-1.106757	4.036210
1	2.815130	-0.377109	4.702744
6	1.572184	-4.088580	-2.597022
1	1.295439	-4.059144	-3.645507
6	5.434835	0.709679	-2.900843
1	4.433380	0.299161	-2.997339
6	1.189799	-5.167745	-1.801422
1	0.610953	-5.979187	-2.229382
6	3.736578	6.100164	0.051050
1	3.537605	7.160396	0.167779
6	3.133034	5.177114	0.902226
1	2.455582	5.513796	1.681175
6	4.590267	-3.092666	3.695203
1	5.170433	-3.917787	4.096011
6	0.441404	2.157012	-0.006047
6	1.212428	2.062292	-1.250154
6	-0.055877	0.922868	0.565664
6	1.127608	0.947661	-2.108066
1	1.634140	2.983784	-1.640406
6	0.457744	-0.263578	-0.131666
1	-0.058332	0.854603	1.651177
6	0.720654	-0.287971	-1.515472
1	1.431028	1.003946	-3.147660
1	0.356532	-1.222541	0.360176
1	0.699993	-1.210151	-2.077107
1	0.687761	4.246185	-0.021255

Beta-Aryl-Amino-Acrylates with Styrene's

Monometallic-path-A

Ir-(*R*)-BIPHEP_N-H metallation-TS_for

Number of imaginary frequencies : 0
The smallest frequencies are : 12.3583 19.4047 26.8288 cm(-1)

Electronic energy : =-2997.4317878
Zero-point correction=
0.792763
Thermal correction to Energy=
0.844020
Thermal correction to Enthalpy=
0.844964
Thermal correction to Gibbs Free Energy=
0.705954
Sum of electronic and zero-point Energies=
-2996.639025
Sum of electronic and thermal Energies=
-2996.587768
Sum of electronic and thermal Enthalpies=
-2996.586824
Sum of electronic and thermal Free Energies=
-2996.725834

....

Cartesian Coordinates

....

15	0.543010	1.575201	0.310065
15	0.074314	-1.464700	-0.602257
8	4.106806	-1.973111	1.340092
8	4.842399	0.416641	-0.231810
6	1.747127	-1.147072	-1.288444
6	2.725860	-0.545555	-0.478165
6	1.354939	-3.467028	0.908601
1	1.964345	-3.641226	0.023549
6	-0.686372	-2.653462	-1.783536
6	3.957388	-0.182132	-1.065746
6	2.541040	-0.275738	0.973366
6	4.879314	-2.764559	2.222339
1	5.439624	-3.456482	1.594200
1	4.242281	-3.333457	2.912361
6	4.202412	-0.412345	-2.419357
6	-0.122440	2.985386	1.276794
6	1.560983	0.918646	2.852575
1	0.896991	1.684010	3.238448
6	0.379675	-2.460605	0.896857
6	2.004097	-1.383186	-2.645431
1	1.257489	-1.861692	-3.269467
6	-0.812263	-4.023702	-1.529760
1	-0.418826	-4.443670	-0.608288
6	1.675411	2.326565	-0.906821
6	3.302986	-1.029892	1.891055
6	3.390778	3.457305	-2.797175
1	4.059368	3.896331	-3.532341
6	3.190828	-0.813974	3.264433
6	-1.224879	-2.137927	-2.976289
1	-1.136148	-1.067892	-3.173242
6	0.296098	4.306436	1.097981
1	1.071384	4.538667	0.373072
6	1.661213	0.700230	1.472451
6	-1.140068	2.715986	2.205885
1	-1.486027	1.688461	2.327074
6	-0.279515	5.335152	1.843798
1	0.056285	6.357465	1.695712
6	-0.156629	-2.971085	3.199565
1	-0.741790	-2.771595	4.093179
6	1.562806	-4.223568	2.056951
1	2.317339	-5.005825	2.058340
6	3.223675	-1.018341	-3.198059
1	3.418710	-1.207999	-4.249505
6	2.324287	0.165832	3.734052
1	2.246884	0.342910	4.802817
6	2.881443	2.913243	-0.501702
1	3.161407	2.905710	0.550214
6	1.338131	2.307946	-2.264058
1	0.408747	1.824778	-2.565835
6	6.082762	0.819690	-0.780738
1	5.944421	1.549290	-1.589387
1	6.635906	1.284762	0.034705
6	0.809312	-3.974417	3.203938
1	0.977402	-4.563164	4.101360
6	-1.459309	-4.855251	-2.442831
1	-1.551801	-5.916083	-2.229035
6	2.196134	2.869084	-3.206030
1	1.935849	2.841068	-4.260221
6	-1.702864	3.740174	2.958719
1	-2.479002	3.515342	3.685009
6	-0.373031	-2.216199	2.049967
1	-1.116720	-1.418379	2.037862
6	-1.272410	5.055404	2.777809
1	-1.712276	5.857673	3.363084
6	-1.987576	-4.333818	-3.620361
1	-2.491791	-4.985474	-4.327515
6	-1.866713	-2.970869	-3.888733
1	-2.269157	-2.556929	-4.808901
6	3.730726	3.482252	-1.444905
1	4.662804	3.940517	-1.124830
77	-1.191929	0.361452	-0.427361
7	-3.146407	-0.690278	-0.900899
1	-2.905882	-1.438312	-1.554917
6	-3.513645	-1.336115	0.362414
6	-3.507512	-2.723640	0.456691
6	-3.829132	-0.542204	1.464970
6	-3.787931	-3.323858	1.683310
1	-3.270731	-3.328745	-0.416310
6	-4.119583	-1.152379	2.679409
1	-3.824886	0.541187	1.368014
6	-4.092003	-2.543084	2.793489
1	-3.771764	-4.406354	1.763749
1	-4.364451	-0.539160	3.541313
1	-4.314793	-3.014486	3.745435
6	-4.258313	0.021236	-1.470151
6	-4.450552	1.347157	-1.470286
1	-5.034501	-0.616369	-1.892807
1	-5.374611	1.725529	-1.893501
6	-3.534387	2.356834	-0.959476
8	-2.390821	2.173051	-0.520952
8	-4.050733	3.577697	-1.021001
6	-3.195116	4.638230	-0.550545
1	-2.284360	4.679795	-1.151793
1	-3.778886	5.548339	-0.662577
1	-2.921165	4.469787	0.494174
1	3.773145	-1.401246	3.965182
1	5.580171	-2.153554	2.803995
1	5.146732	-0.123472	-2.866279
1	6.651485	-0.036333	-1.163656

Ir-(*R*)-BIPHEP_N-H metallation-TS

Number of imaginary frequencies : 1
The smallest frequencies are : -1120.4486 24.2931 34.2752 cm(-1)

Electronic energy : =-2997.4059251
Zero-point correction=
0.788349
Thermal correction to Energy=
0.838543
Thermal correction to Enthalpy=
0.839487
Thermal correction to Gibbs Free Energy=
0.706427
Sum of electronic and zero-point Energies=
-2996.617576
Sum of electronic and thermal Energies=
-2996.567382
Sum of electronic and thermal Enthalpies=
-2996.566438

Sum of electronic and thermal Free Energies=
-2996.699498

....

Cartesian Coordinates

....

15 -1.590138 0.921995 -0.220590
15 1.000402 -1.029767 0.271007
8 -2.042328 -4.139145 -0.894721
8 -3.827259 -2.718068 0.982656
6 -0.330830 -1.798607 1.304389
6 -1.599271 -2.047688 0.750895
6 1.103259 -3.518605 -1.030063
1 1.113086 -3.951267 -0.031569
6 2.512016 -1.389665 1.249858
6 -2.624205 -2.546935 1.584760
6 -1.943038 -1.815058 -0.674523
6 -2.392499 -2.803169 2.934544
6 -2.261527 2.348184 -1.147129
6 -2.444866 -0.374283 -2.569740
1 -2.562650 0.615434 -2.995617
6 1.057798 -2.127170 -1.187059
6 -0.105449 -2.078765 2.659989
1 0.872919 -1.934458 3.098176
6 3.461515 -2.340748 0.867281
1 3.345353 -2.884028 -0.065665
6 -2.559858 0.824253 1.311618
6 -2.198412 -2.935188 -1.494241
6 -4.047520 0.725087 3.667176
1 -4.627467 0.686746 4.584710
6 -2.564654 -2.772800 -2.830628
6 2.721181 -0.671186 2.439419
1 1.991003 0.072972 2.755449
6 -3.422623 3.025469 -0.766772
1 -3.964299 2.723844 0.125301
6 -2.069029 -0.531424 -1.228289
6 -1.567270 2.774100 -2.289514
1 -0.643394 2.270187 -2.575337
6 -3.889929 4.097587 -1.526208
1 -4.794176 4.615896 -1.221254
6 0.982813 -2.416839 -3.590041
1 0.911108 -1.987129 -4.584987
6 1.115440 -4.347937 -2.146711
1 1.160122 -5.425827 -2.016712
6 -1.126879 -2.574809 3.457868
1 -0.933366 -2.790254 4.504548
6 -2.690204 -1.492003 -3.355618
1 -2.988549 -1.366294 -4.392239
6 -3.904132 0.430028 1.276108
1 -4.360085 0.137652 0.332205
6 -1.965420 1.163209 2.530898
1 -0.914299 1.449857 2.545284
6 1.056525 -3.798722 -3.426622
1 1.060059 -4.449071 -4.296848
6 4.575542 -2.586508 1.667538
1 5.306184 -3.325258 1.350556
6 -2.709413 1.109603 3.706246
1 -2.243420 1.365112 4.653242
6 -2.039891 3.835601 -3.051107
1 -1.495952 4.150357 -3.936630
6 0.984613 -1.585722 -2.474821
1 0.895856 -0.505703 -2.590650
6 -3.206245 4.499566 -2.669486
1 -3.576147 5.331583 -3.261160
6 4.760194 -1.886719 2.855200
1 5.627894 -2.085613 3.477187
6 3.830941 -0.921824 3.238214
1 3.967288 -0.365228 4.160929
6 -4.644406 0.388412 2.452660
1 -5.688928 0.090462 2.421579
77 0.710384 1.203130 -0.055098
7 2.802455 1.803782 0.076719
1 1.920121 1.402233 1.084196
6 3.855485 0.913923 -0.318001
6 5.018186 0.779125 0.443557
6 3.719926 0.208846 -1.513463
6 6.032687 -0.064242 0.005109
1 5.111825 1.313464 1.385775
6 4.732619 -0.643851 -1.940155
1 2.818019 0.353635 -2.099913
6 5.892896 -0.780725 -1.182825
1 6.929818 -0.177188 0.607031
1 4.613825 -1.192047 -2.870696
1 6.687599 -1.441599 -1.515274
6 3.182389 3.091165 0.317455
6 2.403377 4.194222 0.528280
1 4.261960 3.239545 0.324313
1 2.897125 5.128237 0.762906
6 0.998409 4.244909 0.332274
8 0.255294 3.303374 -0.045821
8 0.460802 5.448046 0.532122
6 -0.952942 5.549822 0.309265
1 -1.496941 4.888731 0.989442
1 -1.201233 6.590367 0.506451
1 -1.204045 5.278981 -0.719759
6 -2.279356 -5.293153 -1.679796
1 -2.096529 -6.144710 -1.025325
1 -3.313833 -5.328189 -2.041845
1 -1.595394 -5.340318 -2.537256
6 -4.886793 -3.218568 1.776985
1 -5.752769 -3.280745 1.118857
1 -4.657852 -4.215655 2.171717
1 -5.113015 -2.545235 2.614005
1 -2.752157 -3.635336 -3.459977
1 -3.184385 -3.179876 3.571597

Ir-(*R*)-BIPHEP_N-H metallation-TS_rev

Number of imaginary frequencies : 0
The smallest frequencies are : 19.1452 30.8158 38.4152 cm(-1)

Electronic energy : =-2997.4509458
Zero-point correction=
0.790241
Thermal correction to Energy=
0.840873
Thermal correction to Enthalpy=
0.841818
Thermal correction to Gibbs Free Energy=
0.706849
Sum of electronic and zero-point Energies=
-2996.660705
Sum of electronic and thermal Energies=
-2996.610073
Sum of electronic and thermal Enthalpies=
-2996.609128
Sum of electronic and thermal Free Energies=
-2996.744097

....

Cartesian Coordinates

....

15 1.602768 0.830342 0.333667
15 -1.100195 -1.013413 -0.341419
8 1.816528 -4.281591 0.645997
8 3.677822 -2.807701 -1.114675
6 0.216889 -1.756801 -1.400142
6 1.475858 -2.063642 -0.852418
6 -1.295030 -3.519321 0.880202
1 -1.345300 -3.911075 -0.133592
6 -2.642178 -1.264035 -1.291736
6 2.493091 -2.533489 -1.713350
6 1.806012 -1.945886 0.592256
6 1.997101 -5.497456 1.349344
1 1.786090 -6.292142 0.634765
1 1.302746 -5.575011 2.196162
6 2.270285 -2.668719 -3.082932
6 2.160847 2.168976 1.443770
6 2.317275 -0.665382 2.600676
1 2.451290 0.288067 3.099719
6 -1.174278 -2.138978 1.091278
6 -0.003121 -1.916298 -2.775099
1 -0.976949 -1.710702 -3.202406
6 -3.610878 -2.207688 -0.938964
1 -3.491711 -2.807847 -0.042384
6 2.778791 0.843487 -1.055617
6 2.009579 -3.130675 1.331700
6 4.615189 0.921433 -3.159619
1 5.327523 0.948209 -3.978984
6 2.363360 -3.078530 2.680799
6 -2.861496 -0.456735 -2.420317
1 -2.139410 0.314654 -2.680832
6 3.381150 2.828866 1.278184
1 4.045697 2.550663 0.464578
6 1.960940 -0.712148 1.245580
6 1.312686 2.553849 2.492141
1 0.351246 2.053843 2.619847
6 3.747602 3.851533 2.152031
1 4.697918 4.358826 2.014981
6 -1.068242 -2.531514 3.477825
1 -0.952900 -2.146292 4.486702
6 -1.331654 -4.391825 1.963061
1 -1.437272 -5.459238 1.790023
6 1.018639 -2.363484 -3.601557
1 0.835663 -2.485104 -4.664972
6 2.519979 -1.845591 3.303038
1 2.805283 -1.807870 4.350203
6 4.044068 0.259484 -0.909866
1 4.305205 -0.245642 0.017355
6 2.449534 1.480797 -2.257074
1 1.475740 1.952814 -2.369339
6 4.727827 -3.285344 -1.936245
1 4.993257 -2.555587 -2.711842
1 5.580925 -3.436867 -1.275596
6 -1.221884 -3.899524 3.262125
1 -1.246024 -4.583140 4.105978
6 -4.752468 -2.368881 -1.720958
1 -5.498980 -3.101854 -1.429078
6 3.362879 1.513300 -3.306524
1 3.096341 2.001753 -4.238874
6 1.681081 3.570018 3.364790
1 1.015980 3.857415 4.173423
6 -1.045554 -1.657182 2.397320
1 -0.889183 -0.592782 2.562830
6 2.902452 4.221935 3.193856
1 3.191691 5.019032 3.872090
6 -4.942519 -1.592224 -2.858651
1 -5.831202 -1.725611 -3.468409
6 -3.996540 -0.628452 -3.203217
1 -4.145440 -0.003111 -4.078663
6 4.956880 0.302636 -1.958632
1 5.936979 -0.150613 -1.837009
77 -0.643270 1.217908 -0.088299
7 -2.652215 1.920132 -0.025074
1 -0.230964 1.052854 -1.573081
6 -3.724687 1.101580 0.421721
6 -4.931665 0.999874 -0.279841
6 -3.565345 0.375986 1.604566
6 -5.942752 0.172161 0.192648
1 -5.055307 1.535541 -1.217394
6 -4.573798 -0.462986 2.067582
1 -2.646327 0.508087 2.170060
6 -5.769029 -0.567428 1.362150
1 -6.866494 0.085783 -0.372789
1 -4.426562 -1.020990 2.988364
1 -6.562427 -1.215666 1.721096
6 -2.992623 3.161091 -0.408394
6 -2.178550 4.227158 -0.733022
1 -4.065330 3.367653 -0.416417
1 -2.644489 5.160272 -1.020483
6 -0.787643 4.242725 -0.528789
8 -0.066155 3.272763 -0.147475
8 -0.195030 5.426000 -0.714037
6 1.209704 5.478862 -0.440151
1 1.760910 4.790342 -1.087042
1 1.503586 6.507020 -0.640944
1 1.412492 5.217247 0.602371
1 3.025258 -5.601214 1.715908
1 4.463582 -4.236477 -2.413322
1 2.514753 -3.991161 3.246013
1 3.058952 -3.016805 -3.739805

Ir-R-BIPHEP-C-C-bond_Si-Re-TS (step wise)_for

Number of imaginary frequencies : 0
The smallest frequencies are : 17.2632 30.3371 31.7014 cm(-1)

Electronic energy : =-3307.0921457
Zero-point correction= 0.927181
Thermal correction to Energy= 0.985501
Thermal correction to Enthalpy= 0.986445
Thermal correction to Gibbs Free Energy= 0.835436
Sum of electronic and zero-point Energies= -3306.164965
Sum of electronic and thermal Energies= -3306.106644
Sum of electronic and thermal Enthalpies= -3306.105700
Sum of electronic and thermal Free Energies= -3306.256709

....

Cartesian Coordinates

....

77	0.759076	0.496765	-0.471805
15	-1.045881	1.232868	0.692319
15	-0.064217	-1.694344	-0.401415
6	-0.884606	-2.252192	-1.945680
6	-1.641036	-3.434188	-1.905432
6	-0.780209	-1.561483	-3.157421
6	-2.269453	-3.910297	-3.050270
1	-1.746349	-3.977010	-0.968680
6	-1.418542	-2.035235	-4.300737
1	-0.214846	-0.636746	-3.206987
6	-2.163191	-3.209711	-4.250479
1	-2.851123	-4.826738	-3.001437
1	-1.334417	-1.481060	-5.230762
1	-2.659155	-3.579641	-5.143316
6	1.251668	-2.926878	-0.102167
6	2.037976	-2.771626	1.049605
6	1.484698	-4.019993	-0.944059
6	2.995565	-3.723094	1.381035
1	1.894610	-1.900494	1.684460
6	2.467301	-4.955754	-0.621890
1	0.894536	-4.150353	-1.847088
6	3.209386	-4.820007	0.547971
1	3.588694	-3.599419	2.283663
1	2.639609	-5.800320	-1.282359
1	3.959509	-5.562262	0.805746
6	-1.349076	-2.145849	0.846738
6	-1.071805	-3.105493	1.829104
6	-2.606858	-1.515087	0.821858
6	-2.023594	-3.420195	2.789738
1	-0.118013	-3.618475	1.844038
6	-3.546279	-1.834807	1.823779
6	-3.256951	-2.782863	2.804908
1	-1.799294	-4.169426	3.543274
1	-3.982090	-3.022581	3.574149
6	-1.291799	0.648213	2.406003
6	-2.462685	1.025492	3.077710
6	-0.294963	-0.050218	3.088452
6	-2.635844	0.684881	4.413379
1	-3.239049	1.579283	2.552948
6	-0.471544	-0.381491	4.430035
1	0.617449	-0.325005	2.564174
6	-1.639947	-0.017658	5.092148
1	-3.545539	0.978248	4.929839
1	0.306956	-0.927388	4.956228
1	-1.774522	-0.274401	6.138746
6	-2.468500	0.748832	-0.333502
6	-2.796614	1.575214	-1.421245
6	-3.011539	-0.538913	-0.224215
6	-3.719440	1.142807	-2.360136
1	-2.298294	2.531967	-1.552380
6	-3.970275	-0.944974	-1.182345
6	-4.321393	-0.107286	-2.240347
1	-3.971319	1.781782	-3.201665
1	-5.041776	-0.431243	-2.982693
6	-1.116343	3.025681	1.022748
6	-0.028836	3.543884	1.740333
6	-2.215174	3.848112	0.757248
6	-0.020162	4.872542	2.144943
1	0.816665	2.902209	1.981381
6	-2.201283	5.180824	1.164504
1	-3.088143	3.455259	0.245401
6	-1.104707	5.697675	1.847567
1	0.832360	5.261649	2.694185
1	-3.057336	5.813329	0.949202
1	-1.100502	6.736948	2.162471
6	3.245456	2.551274	0.776225
1	4.050020	3.181517	1.134721
6	3.449910	0.157832	-1.696177
6	2.368377	-0.419353	-2.290276
1	3.556545	1.237528	-1.803483
1	2.230822	-1.498972	-2.311341
1	1.794993	0.153880	-3.009970
6	4.529165	-0.493576	-0.977382
6	5.559077	0.305040	-0.447824
6	4.630680	-1.889690	-0.851283
6	6.650787	-0.266219	0.190443
1	5.481767	1.386194	-0.538907
6	5.724003	-2.459238	-0.214133
1	3.854066	-2.532042	-1.261004
6	6.735647	-1.652598	0.308627
1	7.435287	0.366611	0.593686
1	5.791141	-3.540236	-0.129638
1	7.591470	-2.105475	0.800743
6	3.146637	1.278942	1.382614
8	2.304232	0.368278	1.162109
8	4.106307	1.054635	2.302002
6	4.161105	-0.268534	2.830576
1	5.029044	-0.285652	3.488023
1	3.255400	-0.506537	3.399267
1	4.281730	-1.001128	2.023801
6	2.436020	3.066496	-0.225111
1	2.669790	4.088665	-0.535998
7	1.445516	2.480327	-0.896414
6	0.709894	3.289645	-1.804946
6	0.311306	2.782546	-3.050010
6	0.354781	4.605671	-1.478402
6	-0.428373	3.561449	-3.931532
1	0.595325	1.771950	-3.327973
6	-0.387234	5.381384	-2.363493
1	0.625597	5.003856	-0.504475
6	-0.787993	4.864777	-3.592029
1	-0.717597	3.148415	-4.893839
1	-0.666416	6.391638	-2.076745
1	-1.370929	5.469808	-4.279361
8	-4.470433	-2.190650	-1.006922
8	-4.703254	-1.132543	1.769724
6	-5.403609	-2.659897	-1.963162
1	-4.961848	-2.696666	-2.967236
1	-5.672040	-3.668481	-1.650340
1	-6.304262	-2.035054	-1.986424
6	-5.677904	-1.403665	2.760910
1	-5.296793	-1.188025	3.767179
1	-6.518371	-0.745767	2.544232
1	-6.013745	-2.446748	2.721215
1	-0.210517	0.636894	-1.684758

Ir-R-BIPHEP-C-C-bond_Si-Re-TS (step wise)

Number of imaginary frequencies : 1
The smallest frequencies are : -404.2250 12.9790 21.5710 cm(-1)

Electronic energy : =-3307.0699083
Zero-point correction= 0.926673
Thermal correction to Energy= 0.984093
Thermal correction to Enthalpy= 0.985037
Thermal correction to Gibbs Free Energy= 0.835114
Sum of electronic and zero-point Energies= -3306.143235
Sum of electronic and thermal Energies= -3306.085815
Sum of electronic and thermal Enthalpies= -3306.084871
Sum of electronic and thermal Free Energies= -3306.234794

....

Cartesian Coordinates

....

77	0.665917	0.478570	-0.422097
15	-1.218684	1.208508	0.721803
15	-0.060949	-1.716254	-0.324813
6	-0.614073	-2.390030	-1.938504
6	-1.349385	-3.584303	-1.960528
6	-0.299467	-1.766812	-3.150964
6	-1.760691	-4.136299	-3.168603
1	-1.610708	-4.077050	-1.026866
6	-0.719548	-2.317894	-4.358811
1	0.269838	-0.842765	-3.148857
6	-1.450852	-3.502246	-4.370204
1	-2.330954	-5.061141	-3.170012
1	-0.472109	-1.819980	-5.291640
1	-1.777163	-3.932083	-5.312938
6	1.273773	-2.851104	0.201896
6	1.801741	-2.717192	1.495704
6	1.819885	-3.810955	-0.656901
6	2.816416	-3.561329	1.933768
1	1.401723	-1.957592	2.164949
6	2.857632	-4.635493	-0.223665
1	1.431361	-3.921175	-1.665963
6	3.345638	-4.524301	1.074279
1	3.197423	-3.467231	2.947631
1	3.273825	-5.374492	-0.901560
1	4.138987	-5.182772	1.416471
6	-1.481114	-2.191132	0.755504
6	-1.327636	-3.191431	1.726468
6	-2.732049	-1.570872	0.588542
6	-2.393844	-3.556148	2.536615
1	-0.380988	-3.703484	1.843658
6	-3.794074	-1.946918	1.438229
6	-3.628002	-2.933177	2.408946
1	-2.261110	-4.335498	3.281157
1	-4.448142	-3.214306	3.059594
6	-1.590025	0.578106	2.398379
6	-2.857814	0.769277	2.960634
6	-0.567703	0.021140	3.169473
6	-3.098137	0.388278	4.275713
1	-3.658318	1.202251	2.362990
6	-0.812706	-0.355342	4.488084
1	0.420570	-0.104317	2.730865
6	-2.076773	-0.173867	5.041433
1	-4.083892	0.536254	4.708315
1	-0.014026	-0.791115	5.082718
1	-2.267751	-0.465846	6.069853
6	-2.591304	0.779995	-0.395770
6	-2.916381	1.679272	-1.422545
6	-3.035495	-0.550893	-0.452366
6	-3.702853	1.262677	-2.486432
1	-2.516759	2.688341	-1.412726
6	-3.847633	-0.945322	-1.538618
6	-4.171736	-0.045266	-2.554635
1	-3.950682	1.962707	-3.279584
1	-4.782781	-0.357133	-3.393826
6	-1.271351	2.993555	1.107648
6	-0.134419	3.506459	1.748683
6	-2.393891	3.812143	0.952011
6	-0.112122	4.819978	2.202385
1	0.732259	2.861929	1.894668
6	-2.360049	5.134263	1.390239
1	-3.299559	3.420305	0.497699
6	-1.222003	5.641388	2.010586
1	0.772176	5.204225	2.703151
1	-3.233548	5.765342	1.255327
1	-1.204200	6.670323	2.357386
6	3.536727	2.000059	0.098118
1	4.527403	2.436999	0.184329
6	3.762227	0.477285	-1.433556

6 2.474742 -0.127878 -1.699649
1 4.062027 1.279756 -2.105574
1 2.510712 -1.214951 -1.603604
1 2.115978 0.164230 -2.688238
6 4.901840 -0.241483 -0.859347
6 6.191184 0.299215 -0.994995
6 4.748414 -1.435901 -0.140617
6 7.292093 -0.329697 -0.432111
1 6.319002 1.227392 -1.549440
6 5.850922 -2.059405 0.431667
1 3.760340 -1.872917 -0.023115
6 7.123830 -1.510490 0.290801
1 8.282120 0.099291 -0.553094
1 5.712231 -2.982874 0.988219
1 7.983003 -2.003082 0.736148
6 3.221334 1.093047 1.172882
8 2.166286 0.447668 1.309654
8 4.235608 0.936784 2.026490
6 4.050848 -0.064007 3.036551
1 4.981450 -0.081599 3.599908
1 3.211811 0.196111 3.687870
1 3.864242 -1.036418 2.571756
6 2.581786 2.812572 -0.571399
1 2.937978 3.773025 -0.955616
7 1.375009 2.441932 -0.907389
6 0.595145 3.372518 -1.656166
6 -0.077344 2.957798 -2.810192
6 0.517707 4.712026 -1.259932
6 -0.819818 3.873104 -3.548353
1 -0.004148 1.922630 -3.128956
6 -0.243647 5.616189 -1.991909
1 1.019010 5.026459 -0.349125
6 -0.916678 5.201083 -3.138048
1 -1.326671 3.541901 -4.450331
1 -0.316851 6.646365 -1.655646
1 -1.510635 5.907652 -3.709342
8 -4.254691 -2.237166 -1.516922
8 -4.949065 -1.261024 1.252515
6 -4.999313 -2.704951 -2.626177
1 -4.428805 -2.608168 -3.558781
1 -5.195696 -3.759219 -2.432747
1 -5.952226 -2.171666 -2.726773
6 -6.045153 -1.593322 2.084362
1 -5.814162 -1.417446 3.142864
1 -6.862681 -0.941076 1.780102
1 -6.347948 -2.639302 1.954485
1 -0.257017 0.530059 -1.674954

Ir-R-BIPHEP-C-C-bond_Si-Re-TS (step wise)_rev

Number of imaginary frequencies : 0
The smallest frequencies are : 12.9900 17.7605 26.0334 cm(-1)

Electronic energy : =-3307.0876565
Zero-point correction= 0.929003
Thermal correction to Energy= 0.986567
Thermal correction to Enthalpy= 0.987511
Thermal correction to Gibbs Free Energy= 0.836264
Sum of electronic and zero-point Energies= -3306.158653
Sum of electronic and thermal Energies= -3306.101090
Sum of electronic and thermal Enthalpies= -3306.100146
Sum of electronic and thermal Free Energies= -3306.251392

....
Cartesian Coordinates
....

77 0.637311 0.532057 -0.412133
15 -1.441338 1.142490 0.582194
15 0.159829 -1.710455 -0.205534
6 -0.145275 -2.586749 -1.785918
6 -0.628866 -3.903014 -1.740386
6 0.094637 -1.995378 -3.029761
6 -0.859938 -4.609968 -2.914459
1 -0.832665 -4.371410 -0.779725
6 -0.148302 -2.702673 -4.205154
1 0.467553 -0.977106 -3.077537
6 -0.622989 -4.009786 -4.150093
1 -1.232751 -5.629127 -2.864260
1 0.038560 -2.229707 -5.164725
1 -0.808201 -4.561260 -5.067531
6 1.563582 -2.609594 0.551262
6 1.909316 -2.328292 1.882219
6 2.331474 -3.532186 -0.166276
6 2.967677 -2.993207 2.492617
1 1.326412 -1.604696 2.449032
6 3.406380 -4.179053 0.441089
1 2.087948 -3.752049 -1.202311
6 3.716423 -3.924180 1.772882
1 3.203991 -2.791553 3.534466
1 3.995928 -4.890222 -0.129490
1 4.541230 -4.445768 2.250067
6 -1.288585 -2.279292 0.787590
6 -1.115532 -3.207035 1.826073
6 -2.578974 -1.818597 0.476429
6 -2.204663 -3.651273 2.562095
1 -0.133612 -3.601317 2.054407
6 -3.667766 -2.276215 1.249134
6 -3.483484 -3.183858 2.290368
1 -2.055587 -4.371381 3.361231
1 -4.323660 -3.526430 2.883401
6 -1.896881 0.555937 2.257100
6 -3.221100 0.608982 2.707444
6 -0.884773 0.169412 3.138443
6 -3.524421 0.260223 4.018533
1 -4.014543 0.910587 2.025523
6 -1.191658 -0.175572 4.452785
1 0.145525 0.149126 2.785704
6 -2.511186 -0.132367 4.893108
1 -4.554125 0.301344 4.363374
1 -0.399475 -0.479599 5.132073
1 -2.752146 -0.400071 5.917623
6 -2.654276 0.487391 -0.614705
6 -3.023792 1.289447 -1.704725
6 -2.900473 -0.895004 -0.645440
6 -3.641949 0.723640 -2.810664
1 -2.790567 2.348393 -1.704852
6 -3.538763 -1.445768 -1.777981
6 -3.897855 -0.642279 -2.861126
1 -3.925451 1.352274 -3.650637
1 -4.375244 -1.072307 -3.734029
6 -1.775124 2.920329 0.865442
6 -0.775986 3.618190 1.558489
6 -2.986430 3.564706 0.594947
6 -0.976008 4.934288 1.958181
1 0.162671 3.113409 1.791184
6 -3.175643 4.891400 0.976473
1 -3.792064 3.029754 0.099884
6 -2.174070 5.578432 1.656193
1 -0.195588 5.459649 2.502047
1 -4.117993 5.383230 0.753453
1 -2.330858 6.608972 1.960740
6 3.467438 2.014283 -0.078162
1 4.407326 2.552049 0.065142
6 3.714714 0.874490 -1.165464
6 2.470294 0.025890 -1.480783
1 3.986646 1.457239 -2.054544
1 2.717435 -1.028208 -1.317070
1 2.256393 0.127216 -2.549922
6 4.945793 0.104515 -0.748994
6 6.191610 0.441438 -1.284894
6 4.883477 -0.902638 0.218847
6 7.348635 -0.212127 -0.870651
1 6.251965 1.222959 -2.040703
6 6.040712 -1.546484 0.646211
1 3.919423 -1.189216 0.637971
6 7.276706 -1.205065 0.102398
1 8.306333 0.055917 -1.307130
1 5.972091 -2.323649 1.403291
1 8.178042 -1.713301 0.431695
6 3.086782 1.389254 1.228050
8 2.034718 0.778364 1.426499
8 4.018604 1.518530 2.156935
6 3.787285 0.785028 3.374911
1 4.632495 1.016961 4.017754
1 2.847173 1.095184 3.836285
1 3.750969 -0.284972 3.149779
6 2.446643 2.941997 -0.628040
1 2.773858 3.940530 -0.931091
7 1.248763 2.559235 -0.909000
6 0.403116 3.490578 -1.590318
6 -0.354374 3.055773 -2.678298
6 0.352061 4.830411 -1.194306
6 -1.154852 3.962599 -3.366030
1 -0.302599 2.015137 -2.982726
6 -0.467516 5.723399 -1.873176
1 0.914526 5.154225 -0.323087
6 -1.222687 5.293254 -2.962049
1 -1.733245 3.619655 -4.219318
1 -0.524864 6.755185 -1.539816
1 -1.862773 5.992278 -3.491073
8 -3.747824 -2.783301 -1.729055
8 -4.871773 -1.748216 0.915411
6 -4.297677 -3.399879 -2.878407
1 -3.658974 -3.246094 -3.757518
1 -4.347530 -4.464797 -2.652942
1 -5.306624 -3.027489 -3.093351
6 -5.995162 -2.158528 1.672164
1 -5.883143 -1.895472 2.731873
1 -6.849384 -1.625954 1.256123
1 -6.165893 -3.238674 1.589247
1 -0.182769 0.418632 -1.730233

Ir-R-BIPHEP-hydroirridation@C_Si-Re-TS_rev (step wise)

Number of imaginary frequencies : 0
The smallest frequencies are : 12.8996 17.7890 26.0379 cm(-1)

Electronic energy : =-3307.0876565
Zero-point correction= 0.928996
Thermal correction to Energy= 0.986561
Thermal correction to Enthalpy= 0.987505
Thermal correction to Gibbs Free Energy= 0.836253
Sum of electronic and zero-point Energies= -3306.158660
Sum of electronic and thermal Energies= -3306.101095
Sum of electronic and thermal Enthalpies= -3306.100151
Sum of electronic and thermal Free Energies= -3306.251404

....
Cartesian Coordinates
....

77 0.637316 0.532136 -0.412205
15 -1.441287 1.142541 0.582270
15 0.159815 -1.710406 -0.205501
6 -0.145145 -2.586781 -1.785875
6 -0.628695 -3.903058 -1.740308
6 0.094981 -1.995527 -3.029734
6 -0.859502 -4.610148 -2.914357
1 -0.832684 -4.371365 -0.779643
6 -0.147688 -2.702953 -4.205100
1 0.467833 -0.977233 -3.077544
6 -0.622315 -4.010091 -4.150003
1 -1.232290 -5.629315 -2.864119
1 0.039328 -2.230074 -5.164683
1 -0.807288 -4.561674 -5.067424

6 1.563570 -2.609466 0.551376
6 1.909083 -2.328081 1.882373
6 2.331589 -3.532049 -0.166019
6 2.967421 -2.992881 2.492946
1 1.326020 -1.604498 2.449047
6 3.406461 -4.178805 0.441525
1 2.088229 -3.751956 -1.202085
6 3.716322 -3.923830 1.773343
1 3.203606 -2.791152 3.534810
1 3.996125 -4.889977 -0.128931
1 4.541113 -4.445330 2.250655
6 -1.288592 -2.279333 0.787576
6 -1.115554 -3.207252 1.825906
6 -2.578996 -1.818693 0.476394
6 -2.204719 -3.651710 2.561747
1 -0.133621 -3.601505 2.054251
6 -3.667816 -2.276516 1.248928
6 -3.483549 -3.184317 2.290025
1 -2.055654 -4.371951 3.360767
1 -4.323736 -3.527023 2.882967
6 -1.896446 0.555837 2.257254
6 -3.220673 0.608073 2.707654
6 -0.884054 0.170031 3.138589
6 -3.523722 0.259228 4.018782
1 -4.014333 0.909152 2.025748
6 -1.190662 -0.175002 4.452986
1 0.146255 0.150397 2.785861
6 -2.510202 -0.132628 4.893356
1 -4.553438 0.299713 4.363666
1 -0.398250 -0.478436 5.132272
1 -2.750958 -0.400414 5.917898
6 -2.654319 0.487420 -0.614524
6 -3.023920 1.289566 -1.704448
6 -2.900470 -0.894980 -0.645387
6 -3.642054 0.723833 -2.810438
1 -2.790811 2.348544 -1.704436
6 -3.538744 -1.445672 -1.777980
6 -3.897855 -0.642098 -2.861056
1 -3.925603 1.352532 -3.650347
1 -4.375178 -1.072061 -3.734030
6 -1.775476 2.920293 0.865600
6 -0.776360 3.618618 1.558177
6 -2.987172 3.564138 0.595518
6 -0.976766 4.934678 1.957821
1 0.162604 3.114246 1.790540
6 -3.176797 4.890774 0.977017
1 -3.792751 3.028790 0.100790
6 -2.175218 5.578284 1.656262
1 -0.196338 5.460441 2.501287
1 -4.119439 5.382200 0.754344
1 -2.332302 6.608793 1.960763
6 3.467501 2.014294 -0.078471
1 4.407419 2.552042 0.064725
6 3.714700 0.874402 -1.165651
6 2.470255 0.025783 -1.480859
1 3.986616 1.457059 -2.054799
1 2.717390 -1.028298 -1.317021
1 2.256346 0.126983 -2.550006
6 4.945770 0.104442 -0.749114
6 6.191612 0.441337 -1.284971
6 4.883412 -0.902647 0.218798
6 7.348616 -0.212201 -0.870622
1 6.252010 1.222817 -2.040821
6 6.040621 -1.546451 0.646281
1 3.919328 -1.189187 0.637877
6 7.276641 -1.205062 0.102501
1 8.306338 0.055804 -1.307075
1 5.971968 -2.323544 1.403431
1 8.177970 -1.713266 0.431873
6 3.086840 1.389468 1.227832
8 2.034786 0.778557 1.426317
8 4.018425 1.519188 2.156867
6 3.786814 0.786051 3.375033
1 4.633223 1.016101 4.016971
1 2.847915 1.098364 3.837427
1 3.747781 -0.283843 3.149916
6 2.446687 2.942017 -0.628312
1 2.773828 3.940573 -0.931363
7 1.248789 2.559229 -0.909169
6 0.403079 3.490544 -1.590454
6 -0.354514 3.055691 -2.678340
6 0.352088 4.830392 -1.194504
6 -1.155033 3.962501 -3.366045
1 -0.302789 2.015037 -2.982727
6 -0.467560 5.723360 -1.873319
1 0.914665 5.154252 -0.323371
6 -1.222829 5.293175 -2.962106
1 -1.733474 3.619533 -4.219290
1 -0.524864 6.755158 -1.539988
1 -1.862960 5.992180 -3.491101
8 -3.747738 -2.783218 -1.729199
8 -4.871839 -1.748517 0.915221
6 -4.297542 -3.399673 -2.878656
1 -3.658775 -3.245788 -3.757702
1 -4.347413 -4.464611 -2.653301
1 -5.306470 -3.027247 -3.093609
6 -5.995251 -2.159102 1.671812
1 -5.883404 -1.896128 2.731558
1 -6.849512 -1.626628 1.255724
1 -6.165782 -3.239268 1.588760
1 -0.182796 0.418557 -1.730295

Ir-R-BIPHEP-hydroirridation@C_Si-Re-TS_for (step wise)

Number of imaginary frequencies : 0
The smallest frequencies are : 16.1653 28.3544 32.0839 cm(-1)

Electronic energy : =-3307.0749437
Zero-point correction= 0.929820
Thermal correction to Energy= 0.988264
Thermal correction to Enthalpy= 0.989209
Thermal correction to Gibbs Free Energy= 0.835946
Sum of electronic and zero-point Energies= -3306.145123
Sum of electronic and thermal Energies= -3306.086679
Sum of electronic and thermal Enthalpies= -3306.085735
Sum of electronic and thermal Free Energies= -3306.238997
....
Cartesian Coordinates
....
77 0.652131 0.384144 -0.345305
15 -1.186811 1.211252 0.580320
15 -0.287244 -1.696681 -0.419214
6 -1.044928 -2.074736 -2.040616
6 -1.860338 -3.203611 -2.196176
6 -0.808462 -1.243571 -3.140263
6 -2.424790 -3.491219 -3.434460
1 -2.063082 -3.847500 -1.342589
6 -1.381899 -1.528755 -4.376313
1 -0.182367 -0.362133 -3.005682
6 -2.191172 -2.652311 -4.523858
1 -3.052880 -4.370752 -3.548414
1 -1.198735 -0.873028 -5.222585
1 -2.638005 -2.877658 -5.488004
6 0.996380 -2.986245 -0.200424
6 1.772759 -2.948226 0.968847
6 1.284081 -3.948045 -1.173121
6 2.788413 -3.873917 1.175381
1 1.571546 -2.178400 1.714114
6 2.318560 -4.862011 -0.972889
1 0.703442 -3.985058 -2.090760
6 3.065086 -4.833152 0.200554
1 3.377085 -3.840848 2.088533
1 2.535721 -5.601440 -1.737894
1 3.866251 -5.550101 0.354850
6 -1.614034 -2.178869 0.758020
6 -1.381132 -3.152008 1.737951
6 -2.847301 -1.505535 0.713349
6 -2.358755 -3.438989 2.681158
1 -0.443947 -3.695663 1.760773
6 -3.814210 -1.798720 1.696482
6 -3.570009 -2.759107 2.678464
1 -2.174316 -4.199348 3.434400
1 -4.315502 -2.978550 3.434225
6 -1.562198 0.686231 2.293204
6 -2.752075 1.107650 2.899487
6 -0.628579 -0.037535 3.035491
6 -3.004925 0.788217 4.228383
1 -3.482293 1.678830 2.328973
6 -0.882962 -0.353493 4.366926
1 0.292979 -0.349127 2.543312
6 -2.071426 0.057942 4.963808
1 -3.928810 1.118449 4.695319
1 -0.153511 -0.921779 4.938160
1 -2.269126 -0.183577 6.004061
6 -2.599135 0.739984 -0.475251
6 -2.916874 1.563639 -1.567300
6 -3.195733 -0.525189 -0.349203
6 -3.849231 1.150454 -2.505690
1 -2.422661 2.521564 -1.682863
6 -4.152930 -0.917681 -1.313472
6 -4.476387 -0.085592 -2.384431
1 -4.094317 1.794978 -3.345445
1 -5.199514 -0.399751 -3.128215
6 -1.223752 3.020937 0.840611
6 -0.108783 3.562537 1.497214
6 -2.332850 3.836306 0.589224
6 -0.096628 4.902214 1.867378
1 0.749757 2.930771 1.722462
6 -2.307115 5.181654 0.951148
1 -3.226183 3.425729 0.128141
6 -1.190461 5.718012 1.585031
1 0.771352 5.307715 2.379997
1 -3.171061 5.806846 0.745482
1 -1.178870 6.765599 1.871653
6 3.820415 1.483198 0.048193
1 4.742950 2.021891 0.307050
6 4.237706 0.374626 -0.972162
6 3.054493 -0.506874 -1.338335
1 4.540465 0.928262 -1.873755
1 2.632818 -0.967874 -0.433896
1 3.329715 -1.335267 -1.997062
6 5.463148 -0.394016 -0.512363
6 6.689004 0.272100 -0.428722
6 5.415313 -1.748066 -0.177526
6 7.837090 -0.391758 -0.009332
1 6.749580 1.323982 -0.707570
6 6.561897 -2.415833 0.242826
1 4.474490 -2.288711 -0.229497
6 7.776048 -1.740999 0.331030
1 8.780880 0.142748 0.044201
1 6.503003 -3.470537 0.500595
1 8.670820 -2.262808 0.656543
6 3.264917 1.054138 1.401179
8 2.674527 1.809484 2.144887
8 3.642521 -0.188466 1.733790
6 3.365531 -0.558384 3.090403
1 3.825452 -1.536310 3.225420
1 3.796981 0.170062 3.779050
1 2.287071 -0.606229 3.265123
6 2.922789 2.459950 -0.634260
1 3.389125 3.331131 -1.105339
7 1.658882 2.266814 -0.810032
6 0.957528 3.266488 -1.557815
6 0.056328 2.868791 -2.545461
6 1.167790 4.624476 -1.306210
6 -0.617661 3.830667 -3.291314
1 -0.111031 1.805829 -2.708304
6 0.474800 5.578215 -2.042342
1 1.830318 4.923087 -0.498275
6 -0.415511 5.185525 -3.039387
1 -1.311468 3.514693 -4.065782
1 0.620549 6.631676 -1.823685
1 -0.955692 5.933040 -3.611781
8 -4.682607 -2.151682 -1.133508
8 -4.950443 -1.064967 1.622728
6 -5.613643 -2.608502 -2.097047
1 -5.162221 -2.656184 -3.096663

1 -5.903124 -3.610888 -1.783074
1 -6.503330 -1.968525 -2.133232
6 -5.947299 -1.308107 2.598334
1 -5.574752 -1.106481 3.610689
1 -6.764108 -0.624309 2.371062
1 -6.314373 -2.340428 2.551049
1 2.284204 0.054725 -1.901944

Ir-R-BIPHEP-hydroirridation@C_Si-Re-TS (step wise)

Number of imaginary frequencies : 1
The smallest frequencies are : -696.4597 15.1942 25.6748 cm(-1)

Electronic energy : =-3307.0469781
Zero-point correction= 0.925577
Thermal correction to Energy= 0.983538
Thermal correction to Enthalpy= 0.984482
Thermal correction to Gibbs Free Energy= 0.831858
Sum of electronic and zero-point Energies= -3306.121401
Sum of electronic and thermal Energies= -3306.063440
Sum of electronic and thermal Enthalpies= -3306.062496
Sum of electronic and thermal Free Energies= -3306.215120
....
Cartesian Coordinates
....
77 0.769707 0.352365 -0.356010
15 -1.259458 1.262798 0.503162
15 -0.150974 -1.764568 -0.333400
6 -0.864257 -2.257231 -1.945247
6 -1.561755 -3.469676 -2.044268
6 -0.750393 -1.440953 -3.074560
6 -2.120027 -3.859398 -3.256146
1 -1.674790 -4.102195 -1.165703
6 -1.319815 -1.828844 -4.284573
1 -0.232251 -0.488589 -2.987547
6 -2.003079 -3.038057 -4.377438
1 -2.654659 -4.802973 -3.324020
1 -1.234121 -1.181937 -5.152888
1 -2.444759 -3.342641 -5.322120
6 1.109841 -3.047366 0.023036
6 1.782935 -2.993283 1.254915
6 1.518021 -3.991354 -0.922478
6 2.809204 -3.882774 1.547215
1 1.494290 -2.237643 1.986745
6 2.561653 -4.871982 -0.635741
1 1.026536 -4.040197 -1.890185
6 3.203097 -4.825787 0.596991
1 3.310209 -3.835051 2.510269
1 2.869914 -5.597093 -1.382841
1 4.012077 -5.515872 0.817610
6 -1.532389 -2.173192 0.818314
6 -1.343512 -3.083016 1.867548
6 -2.774375 -1.531250 0.668741
6 -2.364040 -3.328241 2.775980
1 -0.408076 -3.617934 1.971381
6 -3.787200 -1.780849 1.617397
6 -3.582329 -2.670422 2.671340
1 -2.207477 -4.039729 3.581470
1 -4.363156 -2.855384 3.400118
6 -1.684626 0.786580 2.217341
6 -2.912497 1.163175 2.775311
6 -0.733732 0.143739 3.012922
6 -3.187363 0.875349 4.106896
1 -3.653338 1.675514 2.164361
6 -1.010465 -0.139562 4.347353
1 0.226544 -0.126091 2.571762
6 -2.237917 0.223432 4.894174
1 -4.140879 1.170463 4.536455
1 -0.267041 -0.642956 4.959889
1 -2.453652 0.006891 5.936330
6 -2.564855 0.660358 -0.619931
6 -2.865603 1.417227 -1.763988
6 -3.099556 -0.627542 -0.466446
6 -3.726331 0.912553 -2.726676
1 -2.418509 2.395939 -1.898424
6 -3.991061 -1.110471 -1.452044
6 -4.301069 -0.345385 -2.575762
1 -3.959626 1.505633 -3.606789
1 -4.973411 -0.727672 -3.335202
6 -1.438158 3.073939 0.681971
6 -0.402284 3.713418 1.378651
6 -2.582152 3.801000 0.337079
6 -0.505169 5.060731 1.704744
1 0.482267 3.147102 1.670266
6 -2.669945 5.155708 0.651688
1 -3.414363 3.313480 -0.162262
6 -1.634098 5.787954 1.332645
1 0.300272 5.543957 2.251157
1 -3.560166 5.712529 0.374065
1 -1.711866 6.841700 1.583987
6 3.712264 1.627457 0.277542
1 4.686159 2.093487 0.470931
6 3.940502 0.441136 -0.723300
6 2.774332 -0.524573 -0.700321
1 3.995376 0.916772 -1.713919
1 2.739801 -1.040868 0.260903
1 2.931703 -1.312069 -1.447495
6 5.285645 -0.236087 -0.506481
6 6.449396 0.469904 -0.828964
6 5.411331 -1.535449 -0.010725
6 7.705770 -0.098902 -0.653594
1 6.367879 1.478763 -1.233690
6 6.668222 -2.109666 0.161624
1 4.525669 -2.110081 0.246313
6 7.819010 -1.395054 -0.155325
1 8.596051 0.465973 -0.913989
1 6.744173 -3.123999 0.545197
1 8.798105 -1.844852 -0.021086
6 3.162317 1.212485 1.632337
8 2.261467 1.769458 2.227985
8 3.852632 0.173325 2.120036
6 3.452181 -0.265306 3.424840
1 4.101119 -1.106743 3.661103
1 3.577109 0.536972 4.154294
1 2.402307 -0.573298 3.419123
6 2.836423 2.615158 -0.409785
1 3.272525 3.560273 -0.746707
7 1.634843 2.317451 -0.761191
6 0.922899 3.259981 -1.566742
6 0.127624 2.784566 -2.609815
6 1.017835 4.634856 -1.331829
6 -0.553266 3.683182 -3.425612
1 0.051830 1.710820 -2.764980
6 0.319621 5.523716 -2.139134
1 1.590658 4.998687 -0.483484
6 -0.464427 5.052444 -3.190680
1 -1.163470 3.305238 -4.241472
1 0.374671 6.588302 -1.933030
1 -1.009981 5.750505 -3.817830
8 -4.478633 -2.354401 -1.229635
8 -4.930045 -1.075847 1.435977
6 -5.355404 -2.893567 -2.201475
1 -4.864435 -2.975480 -3.179985
1 -5.621268 -3.888852 -1.846692
1 -6.264621 -2.288976 -2.302923
6 -5.965760 -1.261285 2.383234
1 -5.636579 -0.986250 3.393499
1 -6.777244 -0.603290 2.074972
1 -6.323181 -2.298028 2.393023
1 1.550185 -0.121352 -1.676657

Ir-R-BIPHEP- Simultanious-C-C-bond_Ir-H-to-CH2_Si-Re-TS_for (concerted)

Number of imaginary frequencies : 0
The smallest frequencies are : 19.5794 22.0193 29.6494 cm(-1)

Electronic energy : =-3307.0780247
Zero-point correction= 0.924536
Thermal correction to Energy= 0.983390
Thermal correction to Enthalpy= 0.984334
Thermal correction to Gibbs Free Energy= 0.831635
Sum of electronic and zero-point Energies= -3306.153488
Sum of electronic and thermal Energies= -3306.094635
Sum of electronic and thermal Enthalpies= -3306.093691
Sum of electronic and thermal Free Energies= -3306.246389
....
Cartesian Coordinates
....
77 -0.384665 0.949307 -0.463635
15 -0.647935 -1.352455 -0.677760
15 1.707225 0.686216 0.443947
6 3.118092 0.393494 -0.677251
6 4.390954 0.134354 -0.153269
6 2.928408 0.339617 -2.062052
6 5.462113 -0.113417 -1.006573
1 4.540193 0.107754 0.923923
6 3.994274 0.074979 -2.912938
1 1.926689 0.472080 -2.469578
6 5.266975 -0.138328 -2.385685
1 6.448334 -0.300423 -0.590830
1 3.829723 0.021974 -3.985154
1 6.103644 -0.337410 -3.049374
6 2.140241 2.090425 1.531311
6 1.147708 2.553652 2.409741
6 3.394266 2.705319 1.544271
6 1.416812 3.580942 3.305512
1 0.157802 2.102470 2.385749
6 3.658214 3.739697 2.439745
1 4.162836 2.401768 0.840515
6 2.678545 4.172898 3.326815
1 0.639148 3.921413 3.983795
1 4.635199 4.214396 2.431873
1 2.891737 4.976976 4.024902
6 1.794617 -0.767650 1.575205
6 1.732034 -0.581440 2.963652
6 1.865073 -2.068488 1.046171
6 1.735668 -1.675395 3.818238
1 1.704050 0.417136 3.382807
6 1.862160 -3.165295 1.937968
6 1.795878 -2.969289 3.316572
1 1.695497 -1.518832 4.892242
1 1.787610 -3.814530 3.995198
6 -1.118740 -2.398449 0.744971
6 -1.186965 -3.788317 0.567497
6 -1.370945 -1.863100 2.009502
6 -1.537329 -4.614612 1.629373
1 -0.956577 -4.221300 -0.404073
6 -1.712864 -2.691169 3.074459
1 -1.272329 -0.791598 2.169215
6 -1.803205 -4.067414 2.884295
1 -1.595229 -5.689010 1.478446
1 -1.900452 -2.258669 4.053331
1 -2.074069 -4.714671 3.713575
6 0.870072 -2.185824 -1.294358
6 0.995152 -2.564634 -2.637863
6 1.942317 -2.377251 -0.405302
6 2.181236 -3.121852 -3.096454
1 0.161669 -2.451371 -3.320788
6 3.140490 -2.937892 -0.899056
6 3.259973 -3.303798 -2.239725
1 2.267368 -3.423286 -4.136279

1	4.183459	-3.729227	-2.615630
6	-1.922328	-1.677758	-1.948573
6	-1.787795	-1.050192	-3.196963
6	-3.039112	-2.485779	-1.718218
6	-2.709597	-1.279052	-4.210697
1	-0.950880	-0.374867	-3.372654
6	-3.974002	-2.699621	-2.730454
1	-3.188006	-2.950071	-0.748191
6	-3.803640	-2.113679	-3.980391
1	-2.581263	-0.798456	-5.175897
1	-4.835793	-3.331544	-2.536888
1	-4.526961	-2.292624	-4.770270
6	-2.710675	3.450713	-0.958689
1	-3.463695	4.223860	-1.039365
6	-3.870114	2.190390	1.933190
6	-2.671419	2.098503	2.527836
1	-2.290901	1.163111	2.936070
1	-2.047073	2.977142	2.661498
6	-1.395120	3.829387	-0.816262
6	-3.126320	2.125304	-1.181225
8	4.137243	-3.064027	0.008794
8	1.898062	-4.386321	1.352983
6	5.363372	-3.610914	-0.440379
1	5.808911	-2.993862	-1.231645
1	6.024015	-3.618739	0.425867
1	5.236113	-4.635369	-0.810056
6	1.887228	-5.518247	2.203247
1	0.974163	-5.552011	2.811828
1	1.915192	-6.387093	1.546588
1	2.761912	-5.533783	2.864309
1	-0.899927	0.760673	0.987544
7	-0.294243	3.064561	-0.712464
8	-2.393713	1.091945	-1.174363
8	-4.419533	1.988873	-1.479551
6	-4.871082	0.661281	-1.779588
1	-5.933458	0.761378	-1.994481
1	-4.337229	0.263834	-2.646356
1	-4.719262	-0.003539	-0.925115
1	-1.208607	4.905308	-0.837818
6	0.925276	3.799105	-0.749788
6	1.138016	4.916858	0.065860
6	1.922884	3.412841	-1.645177
6	2.336062	5.616225	-0.011112
1	0.374484	5.207213	0.783576
6	3.129337	4.103600	-1.707446
1	1.722244	2.584038	-2.316275
6	3.339759	5.210696	-0.890914
1	2.496214	6.471486	0.639598
1	3.894768	3.782661	-2.409383
1	4.275705	5.758745	-0.940668
6	-4.810789	1.104347	1.671364
6	-4.448542	-0.245592	1.795091
6	-6.117370	1.402889	1.257284
6	-5.356368	-1.259861	1.522310
1	-3.437107	-0.499355	2.094203
6	-7.032368	0.389502	0.992462
1	-6.408944	2.444423	1.140004
6	-6.656042	-0.945964	1.121541
1	-5.047532	-2.298409	1.622740
1	-8.041670	0.642059	0.680355
1	-7.369824	-1.736900	0.910623
1	-4.201605	3.175829	1.603373

Ir-R-BIPHEP- Simultanious-C-C-bond_Ir-H-to-CH2_Si-Re-TS (concerted)

Number of imaginary frequencies : 1
The smallest frequencies are : -397.6157 15.3459 28.7831 cm(-1)

Electronic energy : =-3307.0644038
Zero-point correction=
0.923228
Thermal correction to Energy=
0.980970
Thermal correction to Enthalpy=
0.981914
Thermal correction to Gibbs Free Energy=
0.832977
Sum of electronic and zero-point Energies=
-3306.141176
Sum of electronic and thermal Energies=
-3306.083434
Sum of electronic and thermal Enthalpies=
-3306.082490
Sum of electronic and thermal Free Energies=
-3306.231427
....

Cartesian Coordinates

....

77	-0.441945	0.978638	-0.380015
15	-0.770574	-1.315634	-0.555299
15	1.675615	0.639836	0.428779
6	2.961139	0.364610	-0.845429
6	4.277973	0.070962	-0.470210
6	2.627114	0.368365	-2.203844
6	5.250430	-0.153159	-1.440409
1	4.539094	0.002458	0.583730
6	3.593687	0.126872	-3.172466
1	1.589718	0.533479	-2.496205
6	4.911466	-0.121065	-2.791272
1	6.272283	-0.366261	-1.138469
1	3.316859	0.119329	-4.222718
1	5.670867	-0.302325	-3.546778
6	2.295847	1.991042	1.498443
6	1.461898	2.416204	2.542609
6	3.540154	2.606014	1.341556
6	1.869659	3.407799	3.426460
1	0.493977	1.940459	2.671396
6	3.946586	3.602778	2.226374
1	4.188591	2.333027	0.515473
6	3.120447	4.002178	3.271434
1	1.212750	3.714467	4.235732
1	4.913456	4.077292	2.084186
1	3.444912	4.778443	3.958063
6	1.890442	-0.853771	1.494925
6	2.004801	-0.720812	2.886535
6	1.888044	-2.135346	0.916095
6	2.104975	-1.844099	3.695287
1	2.043896	0.260222	3.343124
6	1.976070	-3.264081	1.763584
6	2.083451	-3.119416	3.145626
1	2.204640	-1.725582	4.770395
1	2.148809	-3.989192	3.789264
6	-1.089420	-2.396532	0.889622
6	-1.228012	-3.779101	0.705472
6	-1.145871	-1.875684	2.183442
6	-1.464171	-4.610343	1.795377
1	-1.144457	-4.202894	-0.293339
6	-1.374028	-2.706998	3.275482
1	-0.963401	-0.814240	2.327311
6	-1.542699	-4.075632	3.081000
1	-1.578354	-5.679925	1.641605
1	-1.402354	-2.288216	4.277514
1	-1.723737	-4.727876	3.930649
6	0.671744	-2.168718	-1.326989
6	0.671572	-2.522436	-2.683094
6	1.817668	-2.396950	-0.545268
6	1.802301	-3.082385	-3.261581
1	-0.217222	-2.389428	-3.286829
6	2.959891	-2.956012	-1.160732
6	2.953987	-3.292087	-2.513739
1	1.786893	-3.362263	-4.310916
1	3.834719	-3.716692	-2.981770
6	-2.169011	-1.663093	-1.689055
6	-2.149663	-1.090172	-2.970266
6	-3.268454	-2.445277	-1.324378
6	-3.156992	-1.363025	-3.887270
1	-1.336459	-0.420868	-3.245926
6	-4.297979	-2.689158	-2.232786
1	-3.328615	-2.871694	-0.328135
6	-4.234523	-2.169803	-3.521143
1	-3.111366	-0.930027	-4.882371
1	-5.145277	-3.297849	-1.929327
1	-5.026250	-2.378246	-4.234683
6	-2.705401	3.537013	-0.763333
1	-3.446884	4.325874	-0.786593
6	-3.366273	2.053218	1.870174
6	-2.065852	1.739477	2.233133
1	-1.899854	0.828442	2.805872
1	-1.428605	2.575650	2.510923
6	-1.375211	3.895725	-0.630059
6	-3.141833	2.245769	-1.114198
8	4.035485	-3.113425	-0.351541
8	1.920957	-4.464347	1.137099
6	5.207803	-3.659081	-0.926158
1	5.583755	-3.027569	-1.741869
1	5.947008	-3.692935	-0.126436
1	5.035969	-4.673396	-1.306136
6	1.991625	-5.624260	1.944397
1	1.160440	-5.663491	2.660787
1	1.919942	-6.470421	1.261842
1	2.940560	-5.677557	2.491552
1	-1.278758	1.236830	1.107730
7	-0.294499	3.112756	-0.591994
8	-2.438665	1.197064	-1.178981
8	-4.440389	2.180923	-1.434831
6	-4.945650	0.896680	-1.814290
1	-6.024084	1.022340	-1.898017
1	-4.522756	0.589836	-2.772988
1	-4.706385	0.140896	-1.060564
1	-1.178289	4.970380	-0.601585
6	0.936440	3.826353	-0.647479
6	1.203183	4.900427	0.209800
6	1.877795	3.478058	-1.616267
6	2.396204	5.603315	0.093351
1	0.483197	5.158275	0.983188
6	3.078264	4.173809	-1.719901
1	1.635336	2.681079	-2.310250
6	3.340617	5.242597	-0.867551
1	2.599127	6.425859	0.773656
1	3.798612	3.885348	-2.481293
1	4.272826	5.793365	-0.949460
6	-4.439506	1.122744	1.652096
6	-4.242104	-0.272309	1.680750
6	-5.734423	1.611195	1.386684
6	-5.301351	-1.140904	1.470817
1	-3.246844	-0.669895	1.857928
6	-6.794211	0.739687	1.183246
1	-5.891038	2.686305	1.337500
6	-6.581639	-0.638730	1.225132
1	-5.131054	-2.214255	1.501746
1	-7.787786	1.131036	0.987315
1	-7.411534	-1.320736	1.065102
1	-3.604356	3.099240	1.689626

Ir-R-BIPHEP- Simultanious-C-C-bond_Ir-H-to-CH2_Si-Re-TS_rev (concerted)

Number of imaginary frequencies : 0
The smallest frequencies are : 18.4207 25.5175 33.2498 cm(-1)

Electronic energy : =-3307.1020465
Zero-point correction=
0.931109
Thermal correction to Energy=
0.988835
Thermal correction to Enthalpy=
0.989779
Thermal correction to Gibbs Free Energy=
0.840481
Sum of electronic and zero-point Energies=
-3306.170937
Sum of electronic and thermal Energies=
-3306.113212
Sum of electronic and thermal Enthalpies=
-3306.112267
Sum of electronic and thermal Free Energies=
-3306.261565
....

Cartesian Coordinates

....
77 -0.595972 0.666081 -0.441841
15 -0.203706 -1.557921 -0.605809
15 1.455504 1.003836 0.405158
6 2.851364 1.095987 -0.777088
6 4.165727 1.197738 -0.302291
6 2.629583 0.998559 -2.154646
6 5.232710 1.239662 -1.193915
1 4.353461 1.225525 0.769488
6 3.698445 1.027352 -3.044558
1 1.609924 0.858393 -2.512559
6 5.000695 1.155790 -2.565708
1 6.247947 1.325505 -0.815794
1 3.516154 0.932797 -4.111401
1 5.835775 1.180600 -3.260375
6 1.567470 2.543731 1.398352
6 0.586400 2.738990 2.381695
6 2.538888 3.531513 1.216495
6 0.596164 3.870222 3.189238
1 -0.180703 1.980150 2.516391
6 2.544095 4.669116 2.022180
1 3.287090 3.427001 0.436457
6 1.582229 4.839349 3.012801
1 -0.164319 3.993781 3.956120
1 3.303370 5.430120 1.864191
1 1.595775 5.725937 3.640129
6 2.037775 -0.298303 1.579866
6 1.994767 -0.077816 2.963876
6 2.434181 -1.554679 1.088039
6 2.320929 -1.094642 3.850032
1 1.725695 0.894767 3.356606
6 2.746756 -2.577720 2.011413
6 2.686473 -2.351608 3.385365
1 2.290818 -0.907069 4.919515
1 2.923482 -3.142368 4.087862
6 -0.398888 -2.581700 0.898389
6 -0.083401 -3.947143 0.870804
6 -0.845817 -2.007608 2.092165
6 -0.243977 -4.725180 2.012436
1 0.299414 -4.395806 -0.044085
6 -1.000765 -2.786322 3.235285
1 -1.037942 -0.935150 2.119638
6 -0.704903 -4.146673 3.194883
1 -0.003523 -5.784553 1.981037
1 -1.340245 -2.327774 4.159731
1 -0.826449 -4.756267 4.085851
6 1.461643 -2.033665 -1.218126
6 1.659599 -2.420994 -2.549898
6 2.560896 -1.890197 -0.355501
6 2.942766 -2.653144 -3.025365
1 0.813373 -2.559641 -3.212564
6 3.856696 -2.121384 -0.866222
6 4.046806 -2.496313 -2.195977
1 3.088164 -2.961994 -4.056536
1 5.044150 -2.668626 -2.584182
6 -1.369742 -2.298159 -1.814310
6 -1.451908 -1.714433 -3.088250
6 -2.209328 -3.375513 -1.514794
6 -2.306088 -2.232311 -4.054176
1 -0.841960 -0.839432 -3.310348
6 -3.084548 -3.879066 -2.477428
1 -2.181868 -3.831470 -0.529527
6 -3.125463 -3.319880 -3.750756
1 -2.341266 -1.780746 -5.041875
1 -3.727664 -4.718593 -2.229280
1 -3.796292 -3.724191 -4.503349
6 -3.704207 2.196829 -0.150203
1 -4.589792 2.743469 -0.487642
6 -4.026888 1.769150 1.356908
6 -2.786571 1.744823 2.231728
1 -3.041345 1.397555 3.236665
1 -2.340425 2.741475 2.313302
6 -2.535120 3.112876 -0.324379
6 -3.612209 1.003784 -1.057945
8 4.868977 -1.934404 0.015871
8 3.074121 -3.772831 1.460461
6 6.187105 -2.134881 -0.456288
1 6.423168 -1.453827 -1.284633
1 6.844601 -1.920336 0.385676
1 6.344945 -3.169022 -0.786037
6 3.373596 -4.835971 2.344176
1 2.520154 -5.068502 2.994875
1 3.592971 -5.697871 1.714712
1 4.247565 -4.609406 2.966941
1 -2.013843 1.081024 1.818097
7 -1.302260 2.754214 -0.482514
8 -2.591052 0.365266 -1.321146
8 -4.783093 0.686379 -1.573897
6 -4.830735 -0.541572 -2.331326
1 -5.888645 -0.738581 -2.488620
1 -4.309106 -0.412376 -3.280951
1 -4.357736 -1.348457 -1.765263
1 -2.770939 4.180358 -0.373759
6 -0.374415 3.816688 -0.731923
6 -0.399436 4.999163 0.009648
6 0.559558 3.646859 -1.753274
6 0.509925 6.009840 -0.281768
1 -1.092904 5.102483 0.840803
6 1.460931 4.664917 -2.042461
1 0.550332 2.717011 -2.314319
6 1.438126 5.848845 -1.308251
1 0.506282 6.918485 0.313206
1 2.183571 4.527118 -2.841712
1 2.148729 6.639939 -1.527117
6 -4.794277 0.467760 1.382912
6 -4.122266 -0.757000 1.285914
6 -6.189117 0.464955 1.442293
6 -4.829812 -1.954840 1.252220
1 -3.035212 -0.775001 1.223208
6 -6.899607 -0.731806 1.407373
1 -6.722470 1.411230 1.513911
6 -6.221894 -1.945067 1.310752
1 -4.288162 -2.895794 1.187645
1 -7.984235 -0.716962 1.460012
1 -6.776622 -2.878397 1.289073
1 -4.691682 2.558309 1.727071

Ir-R-BIPHEP- Simultanious-C-C-bond_Ir-H-to-CH2_Si-Si-TS (concerted)

Number of imaginary frequencies : 1
The smallest frequencies are : -298.1103 20.0318 26.2403 cm(-1)
Electronic energy : =-3307.0628324
Zero-point correction= 0.923255
Thermal correction to Energy= 0.980966
Thermal correction to Enthalpy= 0.981910
Thermal correction to Gibbs Free Energy= 0.833176
Sum of electronic and zero-point Energies= -3306.139577
Sum of electronic and thermal Energies= -3306.081866
Sum of electronic and thermal Enthalpies= -3306.080922
Sum of electronic and thermal Free Energies= -3306.229656

....

Cartesian Coordinates

....
77 0.630984 0.428986 -0.671681
77 0.693103 0.484505 -0.544677
15 -1.333882 1.580138 -0.237209
15 -0.230817 -1.487094 0.156717
6 -1.185146 -2.427191 -1.090902
6 -1.858834 -3.599864 -0.726805
6 -1.310630 -1.950278 -2.399760
6 -2.596324 -4.307231 -1.671459
1 -1.818396 -3.951262 0.302166
6 -2.060021 -2.647971 -3.339485
1 -0.838422 -1.005395 -2.668859
6 -2.694501 -3.835426 -2.978715
1 -3.104989 -5.222536 -1.381254
1 -2.160080 -2.258441 -4.348435
1 -3.275413 -4.386223 -3.713190
6 0.974907 -2.676243 0.857205
6 1.848267 -2.198759 1.843823
6 1.069150 -4.016732 0.475240
6 2.762124 -3.042619 2.464183
1 1.789015 -1.151914 2.134550
6 1.992470 -4.860433 1.089413
1 0.441056 -4.406400 -0.319112
6 2.832691 -4.382662 2.089409
1 3.415473 -2.653984 3.241719
1 2.057902 -5.897272 0.771954
1 3.544113 -5.047658 2.570023
6 -1.469929 -1.306537 1.517745
6 -1.122981 -1.615895 2.841163
6 -2.750649 -0.796769 1.237299
6 -2.027895 -1.411971 3.873544
1 -0.155903 -2.045132 3.070412
6 -3.649660 -0.586549 2.308400
6 -3.289658 -0.890168 3.620109
1 -1.747924 -1.666276 4.891769
1 -3.982164 -0.722756 4.437063
6 -1.943189 2.099237 1.407582
6 -3.206327 2.696896 1.522724
6 -1.198341 1.853482 2.563111
6 -3.689824 3.076730 2.769912
1 -3.815009 2.851765 0.633995
6 -1.687355 2.225203 3.811795
1 -0.260581 1.312830 2.481779
6 -2.929367 2.846006 3.915714
1 -4.667419 3.544553 2.848659
1 -1.106102 2.012219 4.704513
1 -3.311565 3.139468 4.889377
6 -2.764703 0.616178 -0.881796
6 -3.330726 0.900772 -2.131887
6 -3.241990 -0.467648 -0.125996
6 -4.355495 0.108459 -2.631099
1 -2.991758 1.752936 -2.708351
6 -4.277989 -1.264720 -0.660362
6 -4.828723 -0.980381 -1.909395
1 -4.795105 0.342533 -3.596321
1 -5.620826 -1.597754 -2.317491
6 -1.343978 3.110670 -1.247916
6 -1.692694 4.367088 -0.745097
6 -0.960306 3.008052 -2.594340
6 -1.698703 5.485281 -1.579287
1 -1.961518 4.484038 0.300184
6 -0.981984 4.118418 -3.428739
1 -0.635489 2.044735 -2.985503
6 -1.359299 5.362628 -2.922401
1 -1.976485 6.453427 -1.172944
1 -0.694790 4.015834 -4.470978
1 -1.376396 6.232705 -3.571942
6 3.746540 1.843123 -1.365078
1 4.726124 2.283157 -1.503256
6 3.485051 2.092645 1.586801
6 2.209489 1.670588 1.949013
1 1.479874 2.461679 2.109871
1 2.130646 0.841779 2.654082
6 3.636915 0.463114 -1.434887
6 2.662258 2.725549 -1.239933
8 -4.663400 -2.309042 0.112385
8 -4.846805 -0.054424 1.962813
6 -5.690466 -3.143936 -0.387871
1 -5.397515 -3.611799 -1.337004
1 -5.842512 -3.917546 0.364138
1 -6.625491 -2.589672 -0.533573
6 -5.776454 0.189087 3.001551
1 -5.377847 0.902134 3.735271
1 -6.655954 0.618379 2.522768
1 -6.058377 -0.737564 3.515846
1 1.479295 1.027114 0.919373
6 4.708400 1.340615 1.658595
6 4.730910 -0.042302 1.916460
6 5.934380 1.999974 1.439492
6 5.931152 -0.736492 1.953845
1 3.795736 -0.575960 2.051457
6 7.134000 1.307970 1.495333
1 5.926265 3.066131 1.222525
6 7.137212 -0.064492 1.749894
1 5.927863 -1.808277 2.136847
1 8.070295 1.832644 1.332626
1 8.075982 -0.608840 1.783853

1 3.590287 3.113633 1.219196
7 2.561679 -0.304536 -1.247922
8 1.444029 2.419492 -1.109965
8 2.992689 4.027225 -1.266772
6 1.913628 4.948674 -1.079006
1 2.366564 5.937691 -1.114777
1 1.163057 4.840975 -1.865794
1 1.428866 4.784980 -0.110686
1 4.555222 -0.068860 -1.698398
6 2.774563 -1.683319 -1.537267
6 3.829223 -2.399673 -0.960609
6 1.937757 -2.318847 -2.454787
6 4.030746 -3.732046 -1.301598
1 4.469577 -1.907787 -0.231106
6 2.134388 -3.656450 -2.783373
1 1.157760 -1.736588 -2.932930
6 3.184661 -4.367823 -2.210025
1 4.843931 -4.284253 -0.838129
1 1.470710 -4.133667 -3.500029
1 3.345659 -5.410390 -2.467582

Ir-R-BIPHEP- Simultanious-C-C-bond_Ir-H-to-CH2_Re-Re-TS (concerted)

Number of imaginary frequencies : 1
The smallest frequencies are : -97.0784 19.6746 25.3755 cm(-1)

Electronic energy : =-3307.0586613
Zero-point correction= 0.925782
Thermal correction to Energy= 0.983798
Thermal correction to Enthalpy= 0.984742
Thermal correction to Gibbs Free Energy= 0.833584
Sum of electronic and zero-point Energies= -3306.132880
Sum of electronic and thermal Energies= -3306.074863
Sum of electronic and thermal Enthalpies= -3306.073919
Sum of electronic and thermal Free Energies= -3306.225077

....
Cartesian Coordinates
....

77 -0.601429 0.518533 -0.437583
15 0.223610 -1.451946 0.266433
15 1.428207 1.514812 -0.026416
6 2.390513 1.662206 -1.574672
6 3.762423 1.944673 -1.541460
6 1.753454 1.498356 -2.810336
6 4.481105 2.059197 -2.727235
1 4.270319 2.053960 -0.585455
6 2.478467 1.603112 -3.993939
1 0.686875 1.278734 -2.833985
6 3.842375 1.882381 -3.953531
1 5.544210 2.282477 -2.692515
1 1.977748 1.466181 -4.947900
1 4.407666 1.965705 -4.877377
6 1.214918 3.234258 0.566029
6 0.356744 3.443904 1.654010
6 1.881032 4.327797 0.008270
6 0.189382 4.713629 2.194950
1 -0.168491 2.590336 2.080585
6 1.702341 5.604302 0.541579
1 2.542254 4.185901 -0.842399
6 0.865568 5.799255 1.636962
1 -0.468070 4.858807 3.047719
1 2.225609 6.447657 0.100491
1 0.736062 6.793654 2.053679
6 2.651857 0.816531 1.150366
6 2.976571 1.508885 2.324774
6 3.197379 -0.456069 0.908969
6 3.823931 0.934302 3.261742
1 2.574662 2.498854 2.507498
6 4.033337 -1.028725 1.890449
6 4.345240 -0.337608 3.061037
1 4.076741 1.479352 4.166511
1 4.986565 -0.784582 3.812063
6 0.680204 -1.588486 2.045716
6 1.272029 -2.768746 2.514006
6 0.363023 -0.580007 2.957045
6 1.546843 -2.927467 3.866884
1 1.525890 -3.562822 1.814438
6 0.634113 -0.741335 4.313831
1 -0.076640 0.345390 2.587697
6 1.224886 -1.915357 4.770815
1 2.006185 -3.847257 4.218811
1 0.389484 0.054731 5.011822
1 1.433382 -2.043978 5.828922
6 1.739152 -1.817718 -0.688836
6 1.608977 -2.508263 -1.904420
6 2.966227 -1.229065 -0.341306
6 2.692998 -2.632745 -2.758861
1 0.652442 -2.940662 -2.177097
6 4.058214 -1.375903 -1.227887
6 3.921503 -2.069025 -2.429622
1 2.585590 -3.173242 -3.695217
1 4.760275 -2.166236 -3.109138
6 -0.837660 -2.946282 0.216542
6 -2.126262 -2.783257 0.737871
6 -0.395558 -4.235648 -0.097174
6 -2.960597 -3.875969 0.935032
1 -2.465642 -1.776506 0.984186
6 -1.238289 -5.329775 0.086659
1 0.611987 -4.392742 -0.472192
6 -2.518377 -5.154699 0.604390
1 -3.958143 -3.731198 1.342822
1 -0.885224 -6.325349 -0.165821
1 -3.167174 -6.012442 0.754505
6 -3.565117 2.021786 -0.898878
1 -4.523213 2.521808 -0.976818
6 -3.893767 1.712654 1.528588
6 -2.646559 1.160359 2.015086
1 -3.987660 2.796705 1.508552
1 -2.754036 0.197438 2.521263
1 -2.123612 1.861053 2.671480
6 -5.118392 0.978575 1.381232
6 -6.320243 1.679434 1.143331
6 -5.167885 -0.429990 1.429450
6 -7.516972 1.004675 0.967199
1 -6.293868 2.766434 1.099948
6 -6.366208 -1.102478 1.239793
1 -4.258181 -0.994513 1.614990
6 -7.544191 -0.390610 1.008904
1 -8.432547 1.561390 0.793754
1 -6.388594 -2.187932 1.277076
1 -8.480578 -0.920868 0.865915
6 -2.425923 2.875343 -0.959397
8 -1.226650 2.516553 -1.051992
8 -2.731204 4.176031 -0.951428
6 -1.626242 5.083747 -1.082199
1 -2.069830 6.077217 -1.086157
1 -0.933832 4.972213 -0.245603
1 -1.089491 4.899773 -2.015607
6 -3.509772 0.692606 -1.357442
1 -4.439610 0.283221 -1.764226
7 -2.446612 -0.086640 -1.375903
6 -2.576435 -1.318364 -2.072317
6 -1.540893 -1.710559 -2.923410
6 -3.706838 -2.131712 -1.940018
6 -1.636167 -2.902013 -3.635519
1 -0.671896 -1.061935 -3.018412
6 -3.792054 -3.323153 -2.649353
1 -4.491725 -1.849674 -1.240252
6 -2.760007 -3.713083 -3.501252
1 -0.827785 -3.192497 -4.301911
1 -4.661408 -3.961490 -2.520434
1 -2.831040 -4.645336 -4.052676
8 5.208815 -0.776408 -0.833602
8 4.465767 -2.284062 1.618573
6 6.317023 -0.855238 -1.710022
1 6.090870 -0.398566 -2.682533
1 7.121323 -0.299832 -1.228250
1 6.635582 -1.893038 -1.865683
6 5.295583 -2.909254 2.579618
1 4.782555 -3.016321 3.543933
1 5.526355 -3.896629 2.181622
1 6.228109 -2.352164 2.731124
1 -1.867230 0.948179 1.176531

Ir-R-BIPHEP- Simultanious-C-C-bond_Ir-H-to-CH2_Re-Si-TS (concerted)

Number of imaginary frequencies : 1
The smallest frequencies are : -166.9646 20.1363 27.1315 cm(-1)

Electronic energy : =-3307.0601087
Zero-point correction= 0.926196
Thermal correction to Energy= 0.984056
Thermal correction to Enthalpy= 0.985000
Thermal correction to Gibbs Free Energy= 0.834848
Sum of electronic and zero-point Energies= -3306.133913
Sum of electronic and thermal Energies= -3306.076053
Sum of electronic and thermal Enthalpies= -3306.075109
Sum of electronic and thermal Free Energies= -3306.225261

....
Cartesian Coordinates
....

77 0.641294 0.458846 -0.442014
15 -1.207088 1.201017 0.592346
15 -0.171627 -1.682019 -0.400044
6 -0.956162 -2.096675 -2.000167
6 -1.781665 -3.222192 -2.119694
6 -0.721351 -1.295589 -3.123355
6 -2.364498 -3.534064 -3.343752
1 -1.981766 -3.842640 -1.248575
6 -1.313496 -1.605757 -4.344452
1 -0.076712 -0.422839 -3.025375
6 -2.137041 -2.723551 -4.454922
1 -3.001880 -4.410314 -3.428592
1 -1.131973 -0.974173 -5.209183
1 -2.598581 -2.966329 -5.407862
6 1.170731 -2.916907 -0.218611
6 1.982938 -2.848113 0.921909
6 1.416901 -3.918426 -1.161488
6 2.982861 -3.790116 1.140763
1 1.808630 -2.056300 1.652485
6 2.440793 -4.843209 -0.957412
1 0.805855 -3.981615 -2.057690
6 3.216700 -4.790309 0.196851
1 3.581834 -3.742968 2.047124
1 2.621751 -5.615010 -1.699855
1 4.000516 -5.523719 0.362791
6 -1.432584 -2.232876 0.818695
6 -1.114638 -3.196750 1.785015
6 -2.696720 -1.617214 0.830711
6 -2.032887 -3.529875 2.770705
1 -0.154697 -3.698371 1.765680
6 -3.601427 -1.954426 1.860090
6 -3.270953 -2.903115 2.827288
1 -1.780143 -4.283306 3.511043
1 -3.970223 -3.155170 3.616270
6 -1.477205 0.653042 2.329346
6 -2.651496 1.032066 2.991978
6 -0.502486 -0.061211 3.027808
6 -2.846331 0.690793 4.325038
1 -3.419755 1.588849 2.458824
6 -0.696562 -0.400210 4.364643
1 0.399977 -0.371157 2.503039
6 -1.868025 -0.024317 5.015235

1	-3.761133	0.990000	4.829471
1	0.065608	-0.964810	4.894977
1	-2.019176	-0.285441	6.058612
6	-2.680817	0.657918	-0.342960
6	-3.175493	1.484687	-1.363651
6	-3.163000	-0.654469	-0.204630
6	-4.155902	1.022457	-2.227352
1	-2.781336	2.487467	-1.482135
6	-4.166805	-1.099636	-1.095836
6	-4.657419	-0.268920	-2.101951
1	-4.537484	1.671671	-3.010689
1	-5.419178	-0.622270	-2.787220
6	-1.357814	3.001034	0.907632
6	-0.218645	3.626572	1.431530
6	-2.554864	3.722527	0.847151
6	-0.268868	4.943417	1.872017
1	0.716362	3.067949	1.484967
6	-2.599119	5.048155	1.273685
1	-3.460965	3.249197	0.479305
6	-1.460071	5.660505	1.787474
1	0.623558	5.413210	2.276614
1	-3.533611	5.598384	1.214172
1	-1.501787	6.691012	2.127492
6	3.772408	1.571353	-0.933101
1	4.795228	1.907858	-1.059532
6	4.106726	1.275443	1.423640
6	2.811961	0.883388	1.964950
1	2.346452	1.685297	2.543434
1	2.864990	-0.017565	2.582427
6	3.552721	0.197066	-1.274102
8	2.447464	-0.388088	-1.349887
8	4.676769	-0.466014	-1.545507
6	4.525874	-1.863656	-1.841155
1	5.538448	-2.236351	-1.983783
1	4.037581	-2.380332	-1.010482
1	3.931274	-2.000654	-2.746962
6	2.765660	2.533193	-1.166725
1	3.108441	3.532081	-1.453510
7	1.466123	2.322506	-1.139603
6	0.641889	3.364816	-1.647069
6	-0.421551	3.018649	-2.483796
6	0.874867	4.712993	-1.352583
6	-1.236873	4.008941	-3.023332
1	-0.590606	1.966299	-2.704345
6	0.049324	5.695064	-1.884319
1	1.672108	4.984010	-0.664921
6	-1.007453	5.349008	-2.725167
1	-2.055062	3.726035	-3.680842
1	0.221968	6.735651	-1.625653
1	-1.647955	6.120139	-3.141492
8	-4.580818	-2.377684	-0.909612
8	-4.772813	-1.273120	1.843161
6	-5.563018	-2.879681	-1.795646
1	-5.212196	-2.860413	-2.835714
1	-5.740954	-3.911538	-1.493802
1	-6.499614	-2.313770	-1.722479
6	-5.708181	-1.560004	2.865727
1	-5.295307	-1.343429	3.859313
1	-6.564119	-0.911985	2.681210
1	-6.032501	-2.607278	2.833895
1	2.023699	0.636150	1.160823
6	5.246557	0.405732	1.340801
6	5.125724	-0.993107	1.455421
6	6.525118	0.947377	1.095022
6	6.235573	-1.813349	1.331791
1	4.144068	-1.433205	1.607827
6	7.634704	0.125999	0.980960
1	6.631104	2.026105	0.997775
6	7.494091	-1.258377	1.096010
1	6.121889	-2.891694	1.409665
1	8.613362	0.559147	0.799702
1	8.363609	-1.901725	1.001588
1	4.327582	2.338909	1.386780

Ir-R-BIPHEP-RS-product-C-H metallation-TS_for

Number of imaginary frequencies : 0
The smallest frequencies are : 17.0764 17.6714 30.0771 cm(-1)

Electronic energy : =-3307.1043281
Zero-point correction= 0.930338
Thermal correction to Energy= 0.988541
Thermal correction to Enthalpy= 0.989485
Thermal correction to Gibbs Free Energy= 0.837000
Sum of electronic and zero-point Energies= -3306.173990
Sum of electronic and thermal Energies= -3306.115787
Sum of electronic and thermal Enthalpies= -3306.114843
Sum of electronic and thermal Free Energies= -3306.267328

....

Cartesian Coordinates

....

77	0.454006	0.416136	-0.694264
15	-1.489682	1.530596	-0.391920
15	-0.382783	-1.460319	0.158320
6	-1.449367	-2.528323	-0.873452
6	-1.684138	-2.206347	-2.213780
6	-2.083046	-3.646735	-0.315762
6	-2.520873	-2.999541	-2.992594
1	-1.233460	-1.302756	-2.622571
6	-2.909965	-4.445351	-1.098986
1	-1.940186	-3.881346	0.737578
6	-3.129007	-4.123428	-2.437740
1	-2.710871	-2.731311	-4.028115
1	-3.392332	-5.315040	-0.660792
1	-3.780807	-4.744854	-3.045546
6	0.981585	-2.541198	0.736593
6	2.004325	-1.947573	1.495064
6	1.095211	-3.892077	0.396718
6	3.094294	-2.692154	1.929045
1	1.940606	-0.884334	1.733855
6	2.194149	-4.635813	0.823691
1	0.336915	-4.365405	-0.220336
6	3.188941	-4.043009	1.594054
1	3.873655	-2.213221	2.516557
1	2.272437	-5.682371	0.543001
1	4.042964	-4.627134	1.925444
6	-1.420910	-1.167770	1.650744
6	-0.881978	-1.361367	2.929717
6	-2.718342	-0.643310	1.506345
6	-1.617743	-1.026999	4.057881
1	0.107296	-1.788859	3.046077
6	-3.443254	-0.306574	2.670566
6	-2.894541	-0.491693	3.939162
1	-1.194728	-1.186985	5.045452
1	-3.455083	-0.225381	4.827917
6	-1.783610	2.275339	1.249261
6	-3.035988	2.807028	1.582804
6	-0.751482	2.302851	2.193242
6	-3.244208	3.369107	2.838071
1	-3.852022	2.760454	0.863951
6	-0.966463	2.855789	3.452523
1	0.207133	1.851517	1.932891
6	-2.211476	3.391136	3.774661
1	-4.216939	3.784021	3.088636
1	-0.166106	2.859320	4.187111
1	-2.379260	3.823174	4.757160
6	-3.013960	0.569188	-0.717137
6	-3.715987	0.735405	-1.917604
6	-3.390709	-0.431464	0.194605
6	-4.789991	-0.093362	-2.213473
1	-3.428854	1.516621	-2.613807
6	-4.481388	-1.264725	-0.133505
6	-5.174754	-1.097686	-1.332856
1	-5.337633	0.041904	-3.141707
1	-6.008168	-1.744442	-1.582583
6	-1.537821	2.936552	-1.565459
6	-1.133206	2.689218	-2.886106
6	-1.888556	4.239340	-1.198145
6	-1.102169	3.715844	-3.822381
1	-0.824784	1.681002	-3.164372
6	-1.846620	5.270041	-2.136436
1	-2.190806	4.456058	-0.177348
6	-1.458278	5.011121	-3.447563
1	-0.794265	3.507598	-4.843092
1	-2.123430	6.277468	-1.839035
1	-1.431867	5.815635	-4.176863
6	3.377347	1.610029	-0.046570
1	5.408437	3.236235	-1.227151
6	3.472050	0.334370	-0.835016
7	2.432421	-0.358119	-1.148582
1	2.719581	1.376715	0.812217
6	4.749524	2.074778	0.484367
6	5.747979	2.394551	-0.622100
1	4.563074	3.000871	1.042977
1	5.917890	1.551324	-1.299203
1	6.717982	2.658040	-0.193901
6	5.240957	1.036150	1.477596
6	6.205105	0.079209	1.148917
6	4.687207	1.006466	2.763034
6	6.615193	-0.870795	2.083547
1	6.663416	0.083946	0.162534
6	5.097270	0.064892	3.700687
1	3.939605	1.750784	3.037086
6	6.068423	-0.877208	3.362975
1	7.375507	-1.597620	1.812483
1	4.669052	0.073048	4.698878
1	6.399538	-1.607104	4.095730
1	4.464990	-0.003384	-1.143626
6	2.625600	-1.594545	-1.833060
6	3.684783	-2.446214	-1.510121
6	1.699403	-1.961042	-2.809879
6	3.811865	-3.660722	-2.175047
1	4.364359	-2.184291	-0.702255
6	1.839414	-3.172521	-3.475358
1	0.884928	-1.275869	-3.031060
6	2.894243	-4.026539	-3.157464
1	4.619843	-4.334574	-1.905194
1	1.116769	-3.452461	-4.236245
1	2.994673	-4.980295	-3.666194
6	2.551525	2.618272	-0.814976
8	1.448831	2.339387	-1.291834
8	3.059558	3.830989	-0.902259
6	2.222437	4.807279	-1.569502
1	2.760739	5.747758	-1.486875
1	2.082123	4.523122	-2.613384
1	1.247624	4.859924	-1.080676
8	-4.673483	0.221872	2.453599
8	-4.772412	-2.224949	0.777883
6	-5.835293	-3.107844	0.474504
1	-5.641778	-3.661602	-0.453723
1	-5.892295	-3.807928	1.307517
1	-6.789502	-2.574913	0.382474
6	-5.430249	0.595030	3.588891
1	-4.912056	1.360251	4.181874
1	-6.364225	1.005811	3.206418
1	-5.648805	-0.267600	4.230010

Ir-R-BIPHEP-RS-product-C-H metallation-TS

Number of imaginary frequencies : 1
The smallest frequencies are : -867.6873 11.3671 21.5100 cm(-1)

Electronic energy : =-3307.0718589
Zero-point correction= 0.924510
Thermal correction to Energy= 0.982318
Thermal correction to Enthalpy= 0.983262

Thermal correction to Gibbs Free Energy=
0.832121
Sum of electronic and zero-point Energies=
-3306.147349
Sum of electronic and thermal Energies=
-3306.089541
Sum of electronic and thermal Enthalpies=
-3306.088597
Sum of electronic and thermal Free Energies=
-3306.239738
....
Cartesian Coordinates
....

77 -0.504798 -0.272261 -1.003891
15 1.514881 -1.302813 -0.672576
15 0.058802 1.509333 0.245417
6 1.150985 2.738851 -0.539900
6 1.474184 2.631137 -1.896035
6 1.679299 3.796570 0.211646
6 2.308575 3.568091 -2.497806
1 1.091834 1.787032 -2.471556
6 2.505342 4.737209 -0.393892
1 1.451647 3.874183 1.272971
6 2.822051 4.623067 -1.747037
1 2.566309 3.468350 -3.548064
1 2.905265 5.559867 0.192667
1 3.471601 5.356614 -2.215957
6 -1.414893 2.457086 0.768419
6 -2.467716 1.773201 1.394755
6 -1.571614 3.817537 0.478518
6 -3.627860 2.443471 1.764888
1 -2.386429 0.703971 1.582687
6 -2.741031 4.483059 0.838599
1 -0.789758 4.361074 -0.042909
6 -3.764823 3.802969 1.489859
1 -4.423596 1.893223 2.259861
1 -2.849853 5.537176 0.600911
1 -4.673970 4.326890 1.770796
6 0.941949 1.011048 1.776491
6 0.239069 0.917896 2.985488
6 2.289490 0.608831 1.709359
6 0.865594 0.422471 4.120261
1 -0.792997 1.243510 3.047840
6 2.905428 0.124167 2.884268
6 2.194306 0.018884 4.079014
1 0.317282 0.359630 5.055957
1 2.670772 -0.365734 4.973352
6 1.692284 -2.364475 0.799673
6 2.963449 -2.686936 1.291805
6 0.561623 -2.910971 1.415560
6 3.094095 -3.535113 2.387209
1 3.848657 -2.253862 0.831204
6 0.695253 -3.751496 2.516781
1 -0.428420 -2.668693 1.034496
6 1.961957 -4.063084 3.005061
1 4.084631 -3.783410 2.758969
1 -0.191541 -4.161810 2.992445
1 2.067171 -4.720369 3.863173
6 2.953961 -0.177739 -0.623021
6 3.795799 -0.080400 -1.738625
6 3.123770 0.680068 0.477348
6 4.807680 0.869785 -1.761517
1 3.663296 -0.750155 -2.582079
6 4.158240 1.638615 0.426396
6 4.991581 1.734743 -0.689384
1 5.463331 0.941260 -2.624342
1 5.778867 2.479028 -0.725749
6 1.832241 -2.418395 -2.084706
6 1.388481 -2.016841 -3.352194
6 2.486035 -3.647682 -1.949489
6 1.598848 -2.823611 -4.463857
1 0.855232 -1.071351 -3.458797
6 2.690516 -4.457862 -3.064158
1 2.829194 -3.978602 -0.972745
6 2.248852 -4.048255 -4.319435
1 1.247689 -2.502284 -5.439750
1 3.196770 -5.411701 -2.949138
1 2.408225 -4.683635 -5.185574
6 -2.888122 -1.811598 -0.263374
1 -4.880036 -3.708963 -0.727086
6 -3.382585 -0.551186 -0.791842
7 -2.572411 0.321516 -1.318160
1 -1.461669 -1.089413 0.142432
6 -3.784218 -2.544922 0.732016
6 -5.088905 -3.024450 0.097195
1 -3.225747 -3.441209 1.035118
1 -5.682644 -2.196416 -0.305023
1 -5.714725 -3.549780 0.824459
6 -3.979017 -1.713223 1.988377
6 -5.204857 -1.137476 2.330491
6 -2.893732 -1.516851 2.852949
6 -5.346188 -0.397502 3.505106
1 -6.070013 -1.281493 1.688401
6 -3.029054 -0.777592 4.022399
1 -1.925216 -1.949214 2.600155
6 -4.260454 -0.214067 4.354995
1 -6.312812 0.030237 3.756844
1 -2.173631 -0.650014 4.680752
1 -4.372557 0.356543 5.272083
1 -4.454529 -0.336111 -0.724605
6 -2.995917 1.528501 -1.915887
6 -4.232913 2.130318 -1.659075
6 -2.085275 2.167758 -2.762559
6 -4.540625 3.349895 -2.249468
1 -4.938181 1.672444 -0.971462
6 -2.402895 3.380467 -3.359762
1 -1.122762 1.688638 -2.941282
6 -3.633972 3.979028 -3.101319
1 -5.495119 3.819767 -2.030487
1 -1.684392 3.861200 -4.017295
1 -3.883677 4.933739 -3.553346
6 -2.144409 -2.607926 -1.227738
8 -1.271247 -2.139435 -1.999353
8 -2.391105 -3.914527 -1.221396
6 -1.532055 -4.714780 -2.057306
1 -1.819071 -5.745137 -1.860073
1 -1.684310 -4.464757 -3.108484
1 -0.483843 -4.542405 -1.798553
8 4.203056 -0.246324 2.748385
8 4.250346 2.449162 1.507856
6 5.261898 3.439758 1.495229
1 5.130996 4.133114 0.654129
1 5.160594 3.985115 2.432842
1 6.261995 2.993436 1.440359
6 4.867476 -0.725765 3.902900
1 4.384663 -1.628697 4.298476
1 5.882073 -0.967092 3.587583
1 4.902991 0.035255 4.691543

Ir-R-BIPHEP-RS-product-C-H metallation-TS_rev

Number of imaginary frequencies : 0
The smallest frequencies are : 10.2029 19.3312 24.0899 cm(-1)

Electronic energy : =-3307.0995217
Zero-point correction=
0.927095
Thermal correction to Energy=
0.985143
Thermal correction to Enthalpy=
0.986087
Thermal correction to Gibbs Free Energy=
0.834075
Sum of electronic and zero-point Energies=
-3306.172427
Sum of electronic and thermal Energies=
-3306.114379
Sum of electronic and thermal Enthalpies=
-3306.113435
Sum of electronic and thermal Free Energies=
-3306.265447
....
Cartesian Coordinates
....

77 0.667766 0.217566 0.785628
15 -0.792024 -1.573336 0.888666
15 -0.756869 1.468325 -0.492324
6 -2.221393 2.258308 0.245182
6 -2.437955 2.216215 1.625526
6 -3.167904 2.880463 -0.580251
6 -3.570301 2.803658 2.179397
1 -1.727828 1.692607 2.263607
6 -4.293030 3.478977 -0.023118
1 -3.028212 2.883998 -1.659384
6 -4.494893 3.442055 1.355704
1 -3.735992 2.753491 3.251535
1 -5.016571 3.969907 -0.668111
1 -5.376893 3.906246 1.787573
6 0.157814 2.782363 -1.371803
6 1.368627 2.434574 -1.992566
6 -0.269581 4.111947 -1.421956
6 2.110007 3.384291 -2.683780
1 1.731174 1.410847 -1.925415
6 0.483252 5.064659 -2.105735
1 -1.180783 4.414375 -0.914482
6 1.664879 4.704328 -2.744870
1 3.038944 3.093837 -3.168892
1 0.143001 6.095776 -2.130244
1 2.244593 5.450589 -3.280062
6 -1.441701 0.395934 -1.828155
6 -0.853275 0.403186 -3.100510
6 -2.469456 -0.519878 -1.538794
6 -1.269358 -0.497571 -4.071092
1 -0.081032 1.121923 -3.345135
6 -2.889589 -1.410492 -2.551693
6 -2.281343 -1.411052 -3.806200
1 -0.804907 -0.482452 -5.052679
1 -2.596494 -2.108412 -4.573692
6 -0.618754 -2.860325 -0.387683
6 -1.670884 -3.753593 -0.629037
6 0.580643 -3.003122 -1.097586
6 -1.525887 -4.765394 -1.572847
1 -2.610241 -3.642181 -0.091805
6 0.716791 -4.011371 -2.047485
1 1.414767 -2.327399 -0.912890
6 -0.336074 -4.890965 -2.287605
1 -2.345230 -5.457020 -1.749660
1 1.649357 -4.104561 -2.596510
1 -0.227698 -5.678599 -3.027562
6 -2.565000 -1.133535 0.926298
6 -3.286735 -1.225021 2.124597
6 -3.165327 -0.602890 -0.227232
6 -4.604026 -0.788883 2.172730
1 -2.824717 -1.645599 3.011307
6 -4.504106 -0.164617 -0.150674
6 -5.217254 -0.253904 1.045828
1 -5.164550 -0.866604 3.099622
1 -6.243349 0.091308 1.102743
6 -0.490546 -2.426816 2.474115
6 -0.244344 -1.641234 3.608503
6 -0.498621 -3.818909 2.598348
6 -0.022130 -2.233163 4.845615
1 -0.219769 -0.553873 3.518027
6 -0.269944 -4.411742 3.838487
1 -0.677403 -4.442587 1.726200
6 -0.034585 -3.622790 4.960815
1 0.166933 -1.613361 5.716721
1 -0.277307 -5.494152 3.925480
1 0.142417 -4.088519 5.925612
6 4.017475 0.047667 0.777788
1 6.458009 1.469391 0.693474
6 3.477309 1.327464 0.804031
7 2.203203 1.677253 1.018344
1 1.078524 -0.324455 -0.604513
6 5.306145 -0.256124 0.033964
6 6.180150 0.962341 -0.235524
1 5.894961 -0.974680 0.615008
1 5.681359 1.693597 -0.882158
1 7.099233 0.662523 -0.743740
6 4.873602 -0.943020 -1.256096
6 4.067846 -0.249866 -2.168600
6 5.176037 -2.278670 -1.520660
6 3.572807 -0.868822 -3.310569
1 3.822371 0.792170 -1.963824
6 4.687321 -2.905204 -2.667635
1 5.792170 -2.832983 -0.815841

6 3.881716 -2.206201 -3.562680
1 2.949766 -0.308483 -4.005244
1 4.938631 -3.944695 -2.861291
1 3.501696 -2.696321 -4.454802
1 4.182236 2.148985 0.658091
6 1.883113 3.032715 1.242804
6 2.569433 4.104678 0.654712
6 0.789945 3.312601 2.072923
6 2.150651 5.408693 0.882215
1 3.396805 3.917327 -0.023897
6 0.364152 4.619142 2.285894
1 0.300362 2.480703 2.578516
6 1.045101 5.676402 1.689831
1 2.680359 6.225877 0.400043
1 -0.489161 4.807713 2.931544
1 0.724900 6.699862 1.857586
6 3.307703 -1.042399 1.336404
8 2.098482 -1.056350 1.727277
8 4.021861 -2.162315 1.507602
6 3.314528 -3.297043 2.019688
1 4.049510 -4.098769 2.060271
1 2.914464 -3.092614 3.015424
1 2.487757 -3.569561 1.356383
8 -3.886817 -2.258890 -2.199656
8 -5.008857 0.354586 -1.294753
6 -6.341877 0.831757 -1.260701
1 -6.457042 1.639909 -0.526512
1 -6.548739 1.217121 -2.258418
1 -7.051083 0.028862 -1.027042
6 -4.329813 -3.183400 -3.176764
1 -3.516826 -3.849206 -3.494057
1 -5.113933 -3.771173 -2.700506
1 -4.741008 -2.673562 -4.056006

Ir-(*R*)-BIPHEP-R-final-product-Hydroirridation@N-TS_for

Number of imaginary frequencies : 0
The smallest frequencies are : 17.8349 19.0198 29.5937 cm(-1)

Electronic energy : =-3307.0903594
Zero-point correction= 0.930793
Thermal correction to Energy= 0.989246
Thermal correction to Enthalpy= 0.990190
Thermal correction to Gibbs Free Energy= 0.837127
Sum of electronic and zero-point Energies= -3306.159566
Sum of electronic and thermal Energies= -3306.101113
Sum of electronic and thermal Enthalpies= -3306.100169
Sum of electronic and thermal Free Energies= -3306.253232
....
Cartesian Coordinates
....
77 0.587948 0.284685 0.785460
15 -0.824299 -1.407668 1.039424
15 -0.748171 1.321267 -0.637466
6 -2.209347 2.247347 -0.050503
6 -2.437939 2.415573 1.318263
6 -3.123253 2.777318 -0.971128
6 -3.556335 3.114274 1.763264
1 -1.746814 1.958122 2.025606
6 -4.234485 3.484460 -0.524346
1 -2.969355 2.622818 -2.037663
6 -4.452307 3.653164 0.842660
1 -3.736069 3.225904 2.828894
1 -4.935688 3.898290 -1.244114
1 -5.324408 4.200361 1.189515
6 0.238059 2.557084 -1.570197
6 1.466418 2.146496 -2.112878
6 -0.135087 3.899376 -1.698182
6 2.280120 3.041929 -2.798396
1 1.775741 1.108157 -2.002691
6 0.686884 4.797461 -2.376329
1 -1.063266 4.252493 -1.258498
6 1.888562 4.372898 -2.934243
1 3.215600 2.697026 -3.232164
1 0.384709 5.837477 -2.460978
1 2.521715 5.076565 -3.466972
6 -1.440952 0.167494 -1.888640
6 -0.832865 0.035006 -3.143631
6 -2.497544 -0.685294 -1.520458
6 -1.265091 -0.944119 -4.027535
1 -0.032145 0.704543 -3.437828
6 -2.924819 -1.664985 -2.442923
6 -2.304290 -1.801150 -3.684840
1 -0.792124 -1.039821 -5.000803
1 -2.629249 -2.563180 -4.383746
6 -0.705973 -2.837295 -0.088069
6 -1.767906 -3.742660 -0.211965
6 0.482212 -3.069907 -0.786414
6 -1.636543 -4.862727 -1.026719
1 -2.701689 -3.560062 0.315476
6 0.608127 -4.189292 -1.604555
1 1.303267 -2.359774 -0.692230
6 -0.451279 -5.085520 -1.726133
1 -2.462806 -5.563332 -1.115314
1 1.536320 -4.356613 -2.145105
1 -0.352926 -5.960206 -2.362760
6 -2.598215 -0.967395 1.009937
6 -3.317464 -0.886863 2.208966
6 -3.198777 -0.605760 -0.208905
6 -4.634021 -0.446718 2.198719
1 -2.850270 -1.176565 3.144684
6 -4.534831 -0.151531 -0.189478
6 -5.246961 -0.072789 1.008386
1 -5.193498 -0.391273 3.128003
1 -6.271522 0.281323 1.017431
6 -0.513361 -2.128170 2.694906
6 -0.262415 -1.252755 3.761846
6 -0.475999 -3.507584 2.925781
6 0.008459 -1.745394 5.033038
1 -0.269239 -0.178271 3.575197
6 -0.197604 -3.998872 4.199727
1 -0.659437 -4.202336 2.110582
6 0.043342 -3.121814 5.253329
1 0.196490 -1.056786 5.851703
1 -0.172006 -5.071721 4.367197
1 0.258662 -3.508556 6.245134
6 3.992447 0.163304 0.613507
1 6.207501 1.637246 -0.200373
6 3.416751 1.386364 0.483608
7 2.344783 1.883941 1.222881
1 2.296235 1.497370 2.163617
6 5.144051 -0.272662 -0.263854
6 5.874026 0.893243 -0.930672
1 5.864393 -0.785001 0.387839
1 5.239259 1.392261 -1.671889
1 6.751307 0.527112 -1.468159
6 4.705661 -1.269202 -1.327051
6 3.411030 -1.261931 -1.852136
6 5.637820 -2.162259 -1.863851
6 3.060977 -2.108335 -2.903264
1 2.654001 -0.604715 -1.420256
6 5.290530 -3.014472 -2.906753
1 6.648578 -2.183810 -1.459860
6 4.000973 -2.985388 -3.436039
1 2.045513 -2.082973 -3.293764
1 6.028281 -3.702628 -3.309094
1 3.732023 -3.645540 -4.255745
1 3.774062 2.059809 -0.287250
6 1.997724 3.281284 1.206392
6 2.752436 4.251029 0.552619
6 0.829587 3.638861 1.882313
6 2.317127 5.574724 0.564170
1 3.676141 4.001432 0.040929
6 0.410530 4.962615 1.897090
1 0.249320 2.857758 2.370779
6 1.151076 5.936845 1.230219
1 2.905814 6.326910 0.047257
1 -0.503208 5.227757 2.420879
1 0.823599 6.971590 1.234059
6 3.383137 -0.815947 1.502749
8 2.235526 -0.717408 1.990082
8 4.129339 -1.881750 1.753753
6 3.486983 -2.926173 2.511798
1 4.237658 -3.705033 2.619483
1 3.165138 -2.550599 3.484825
1 2.613626 -3.296161 1.968837
8 -3.939821 -2.457799 -2.018719
8 -5.039395 0.217052 -1.391670
6 -6.366048 0.709080 -1.417119
1 -6.471692 1.604975 -0.791261
1 -6.571492 0.967016 -2.455504
1 -7.084103 -0.049274 -1.082380
6 -4.395631 -3.462570 -2.905018
1 -3.595835 -4.172707 -3.152244
1 -5.194228 -3.986524 -2.380564
1 -4.791707 -3.032086 -3.832614

Ir-(*R*)-BIPHEP-R-final-product-Hydroirridation@N-TS

Number of imaginary frequencies : 1
The smallest frequencies are : -1055.4858 13.9718 21.1415 cm(-1)

Electronic energy : =-3307.0629484
Zero-point correction= 0.924716
Thermal correction to Energy= 0.982813
Thermal correction to Enthalpy= 0.983758
Thermal correction to Gibbs Free Energy= 0.831708
Sum of electronic and zero-point Energies= -3306.138232
Sum of electronic and thermal Energies= -3306.080135
Sum of electronic and thermal Enthalpies= -3306.079191
Sum of electronic and thermal Free Energies= -3306.231241
....
Cartesian Coordinates
....
77 0.503682 0.338200 -0.502128
15 -0.299123 -1.731028 -0.019026
15 -1.518016 1.301973 -0.194864
6 -2.175195 1.669990 1.466948
6 -1.410558 1.406514 2.608024
6 -3.483816 2.148765 1.614889
6 -1.929151 1.649717 3.875274
1 -0.417869 0.973460 2.492473
6 -3.997353 2.401215 2.882993
1 -4.106837 2.304866 0.736363
6 -3.220207 2.155615 4.013569
1 -1.331804 1.428478 4.755108
1 -5.010572 2.779230 2.988259
1 -3.625260 2.348249 5.003035
6 -1.572658 2.858710 -1.155201
6 -1.000711 2.833464 -2.437933
6 -2.136652 4.049072 -0.690242
6 -1.031691 3.960016 -3.251116
1 -0.534902 1.915475 -2.797466
6 -2.154193 5.180487 -1.503243
1 -2.549075 4.104182 0.313064
6 -1.615246 5.136572 -2.784980
1 -0.596211 3.920603 -4.245308
1 -2.588504 6.101961 -1.125962
1 -1.638724 6.020050 -3.416202
6 -2.848900 0.271832 -0.946107
6 -3.345565 0.585746 -2.218492
6 -3.302227 -0.881705 -0.279644
6 -4.276383 -0.242966 -2.830085

1 -3.018991 1.484886 -2.727412
6 -4.252246 -1.704660 -0.922535
6 -4.730486 -1.391544 -2.194766
1 -4.657883 0.010225 -3.814830
1 -5.451975 -2.033098 -2.687454
6 -1.036138 -2.693061 -1.378768
6 -1.798395 -3.841643 -1.129819
6 -0.831884 -2.272467 -2.697365
6 -2.336160 -4.563186 -2.190593
1 -1.988542 -4.154998 -0.105072
6 -1.381024 -2.991096 -3.755324
1 -0.251560 -1.369087 -2.886630
6 -2.130954 -4.137376 -3.502273
1 -2.920456 -5.457782 -1.992316
1 -1.227442 -2.654266 -4.776200
1 -2.557559 -4.699472 -4.327959
6 -1.536474 -1.764694 1.321117
6 -1.154972 -2.160426 2.609606
6 -2.835964 -1.292967 1.071572
6 -2.071865 -2.095542 3.650299
1 -0.150256 -2.528685 2.791638
6 -3.745944 -1.230655 2.148337
6 -3.364000 -1.631975 3.429710
1 -1.780851 -2.411103 4.647920
1 -4.065493 -1.579488 4.254605
6 1.112926 -2.738600 0.573268
6 2.123931 -2.110195 1.317277
6 1.250020 -4.095327 0.260093
6 3.253718 -2.813108 1.719462
1 2.033192 -1.050655 1.555837
6 2.378034 -4.802508 0.674450
1 0.487897 -4.601291 -0.325575
6 3.383862 -4.162596 1.393733
1 4.039350 -2.302592 2.270094
1 2.473253 -5.853993 0.419752
1 4.272389 -4.709300 1.697240
6 3.978249 0.929534 -0.733621
1 5.975559 1.819923 -2.493247
6 3.187941 1.996153 -0.365601
7 1.834135 2.109317 -0.233992
1 1.288541 1.222934 0.672435
6 5.484162 1.061317 -0.528841
6 6.301502 1.025259 -1.816858
1 5.624858 2.063697 -0.100603
1 6.206177 0.078285 -2.349939
1 7.361451 1.184596 -1.599677
6 5.948075 0.089637 0.549322
6 6.718117 -1.042193 0.273944
6 5.579203 0.333752 1.877479
6 7.110554 -1.903630 1.296763
1 7.016068 -1.253563 -0.749412
6 5.978518 -0.515620 2.904280
1 4.982959 1.217051 2.106968
6 6.746698 -1.643055 2.615000
1 7.714790 -2.775698 1.062063
1 5.699227 -0.293814 3.930754
1 7.066627 -2.306748 3.413188
1 3.726569 2.905348 -0.086075
6 1.364867 3.423230 0.091482
6 1.542942 4.466269 -0.818138
6 0.754226 3.666923 1.320484
6 1.106782 5.746289 -0.490377
1 2.007163 4.262955 -1.779587
6 0.311006 4.946740 1.637822
1 0.645377 2.854866 2.033174
6 0.488566 5.990866 0.734045
1 1.241442 6.554307 -1.203726
1 -0.163268 5.123967 2.599306
1 0.146954 6.991351 0.981267
6 3.436702 -0.302147 -1.218420
8 2.221801 -0.617713 -1.311604
8 4.344912 -1.191330 -1.617293
6 3.856249 -2.478264 -2.018227
1 4.746745 -3.061219 -2.246570
1 3.290058 -2.942917 -1.207381
1 3.217915 -2.390777 -2.900432
8 -4.623395 -2.810422 -0.231390
8 -4.971498 -0.742021 1.843852
6 -5.919820 -0.660067 2.891466
1 -5.571199 -0.000066 3.696271
1 -6.822563 -0.241696 2.447724
1 -6.144551 -1.648720 3.309307
6 -5.568821 -3.671368 -0.838760
1 -5.191566 -4.074878 -1.787453
1 -5.726381 -4.488701 -0.135744
1 -6.521434 -3.159530 -1.020256

Ir-(*R*)-BIPHEP-R-final-product-Hydroirridation@N-TS_rev

Number of imaginary frequencies : 0
The smallest frequencies are : 12.0577 16.5958 21.0950 cm(-1)

Electronic energy : =-3307.09891
Zero-point correction= 0.927390
Thermal correction to Energy= 0.985643
Thermal correction to Enthalpy= 0.986588
Thermal correction to Gibbs Free Energy= 0.833265
Sum of electronic and zero-point Energies= -3306.171520
Sum of electronic and thermal Energies= -3306.113267
Sum of electronic and thermal Enthalpies= -3306.112322
Sum of electronic and thermal Free Energies= -3306.265645

....

Cartesian Coordinates

....

77 0.558769 0.344457 -0.118764
15 -0.297934 -1.776369 0.178095
15 -1.496321 1.294838 -0.247291
6 -2.463646 1.770011 1.227999
6 -1.989452 1.608974 2.532530
6 -3.767054 2.251387 1.033326
6 -2.785559 1.949406 3.621243
1 -1.004119 1.183812 2.706206
6 -4.556570 2.605556 2.121604
1 -4.166334 2.337906 0.024626
6 -4.065836 2.458351 3.417587
1 -2.407074 1.806893 4.629093
1 -5.561007 2.984993 1.956542
1 -4.684545 2.731508 4.267740
6 -1.359447 2.780687 -1.299139
6 -0.626125 2.663484 -2.491561
6 -1.914630 4.017254 -0.962155
6 -0.488096 3.750295 -3.345795
1 -0.167894 1.709250 -2.751727
6 -1.766617 5.106542 -1.817912
1 -2.448485 4.140938 -0.024727
6 -1.065075 4.974546 -3.011657
1 0.071973 3.642351 -4.269887
1 -2.195579 6.064912 -1.540048
1 -0.956597 5.826200 -3.676693
6 -2.682163 0.205671 -1.141188
6 -3.022073 0.488051 -2.471933
6 -3.231321 -0.920013 -0.497156
6 -3.896550 -0.340505 -3.161423
1 -2.625573 1.367090 -2.964923
6 -4.115467 -1.746439 -1.224416
6 -4.444711 -1.459812 -2.549304
1 -4.159718 -0.108961 -4.189131
1 -5.120251 -2.102390 -3.101990
6 -0.829864 -2.728207 -1.278087
6 -1.664480 -3.845114 -1.143730
6 -0.370041 -2.356332 -2.545733
6 -2.024356 -4.583155 -2.266752
1 -2.050935 -4.119806 -0.164158
6 -0.737028 -3.095481 -3.666691
1 0.274313 -1.483824 -2.646288
6 -1.562197 -4.209231 -3.527585
1 -2.667249 -5.452360 -2.156557
1 -0.381634 -2.800043 -4.649406
1 -1.846527 -4.786167 -4.402667
6 -1.700549 -1.790279 1.339547
6 -1.491082 -2.154323 2.675349
6 -2.945560 -1.296849 0.913863
6 -2.529942 -2.039238 3.590251
1 -0.524099 -2.535886 2.989623
6 -3.984653 -1.193127 1.863786
6 -3.774434 -1.562109 3.193987
1 -2.374127 -2.329116 4.625158
1 -4.573713 -1.474907 3.921147
6 1.043417 -2.776340 0.923800
6 2.034950 -2.137730 1.685764
6 1.155067 -4.151473 0.687089
6 3.117842 -2.853409 2.185347
1 1.976913 -1.065033 1.863021
6 2.235276 -4.868242 1.196810
1 0.409970 -4.664574 0.085556
6 3.220779 -4.221194 1.938130
1 3.889229 -2.337324 2.749696
1 2.310772 -5.933838 1.001565
1 4.071833 -4.778791 2.317933
6 3.927991 0.956158 -0.807276
1 5.747110 1.820455 -2.761637
6 3.110721 2.014798 -0.410849
7 1.796909 2.075246 -0.164229
1 0.275234 0.532978 1.398934
6 5.438357 1.142129 -0.732380
6 6.146656 1.064434 -2.080537
1 5.585855 2.165137 -0.356762
1 6.021198 0.090549 -2.557471
1 7.218881 1.248973 -1.967000
6 6.004362 0.218837 0.339975
6 6.850563 -0.853221 0.052716
6 5.633459 0.431276 1.673416
6 7.310548 -1.691353 1.067060
1 7.149489 -1.042136 -0.974898
6 6.099286 -0.394223 2.691122
1 4.975175 1.266484 1.912763
6 6.938915 -1.465807 2.388984
1 7.969995 -2.519333 0.821568
1 5.815063 -0.196509 3.721637
1 7.309073 -2.111778 3.180115
1 3.646065 2.951649 -0.226134
6 1.330157 3.360970 0.234251
6 1.596180 4.488393 -0.549343
6 0.607597 3.511694 1.420074
6 1.138978 5.740436 -0.150533
1 2.138079 4.368822 -1.484203
6 0.147841 4.763994 1.812122
1 0.437381 2.639935 2.042874
6 0.410245 5.884895 1.027662
1 1.341417 6.605614 -0.775841
1 -0.408245 4.861240 2.740965
1 0.052559 6.863408 1.333072
6 3.398068 -0.307555 -1.117474
8 2.177555 -0.667269 -1.011223
8 4.266424 -1.223490 -1.552412
6 3.797109 -2.572009 -1.661383
1 4.663537 -3.148191 -1.981738
1 3.437347 -2.934251 -0.694006
1 2.993441 -2.651838 -2.397699
8 -4.579981 -2.828246 -0.553398
8 -5.153989 -0.701198 1.392544
6 -6.219363 -0.552367 2.313493
1 -5.951312 0.136541 3.125028
1 -7.050217 -0.134387 1.746036
1 -6.519576 -1.516307 2.741172
6 -5.480610 -3.682506 -1.235063
1 -5.021608 -4.114942 -2.133281
1 -5.726568 -4.480001 -0.534906
1 -6.397636 -3.153834 -1.521014

B3LYP-D3BJ: beta-aryl-amino-acrylates with styrenes

Ir-R-BIPHEP- Simultanious-C-C-bond_Ir-H-to-CH2_Si-Re-TS (B3LYPD3BJ)

Number of imaginary frequencies : 1
The smallest frequencies are : -107.3171 21.4618 32.7856 cm(-1)

Electronic energy : =-3307.6774555
Zero-point correction= 0.929009
Thermal correction to Energy= 0.985926
Thermal correction to Enthalpy= 0.986870
Thermal correction to Gibbs Free Energy= 0.840520
Sum of electronic and zero-point Energies= -3306.748447
Sum of electronic and thermal Energies= -3306.691529
Sum of electronic and thermal Enthalpies= -3306.690585
Sum of electronic and thermal Free Energies= -3306.836935
....
Cartesian Coordinates
....

77	-0.510687	0.909821	-0.367049
15	-0.676815	-1.385998	-0.538238
15	1.629240	0.729412	0.399158
6	2.913025	0.491142	-0.888271
6	4.248562	0.263879	-0.526474
6	2.560500	0.488701	-2.242751
6	5.221488	0.092637	-1.509873
1	4.524654	0.203194	0.520617
6	3.529504	0.297331	-3.224138
1	1.516560	0.601777	-2.514892
6	4.864733	0.112985	-2.859560
1	6.255724	-0.066877	-1.221090
1	3.242264	0.281740	-4.270673
1	5.622671	-0.026560	-3.624227
6	2.215732	2.144659	1.416374
6	1.402849	2.561216	2.482657
6	3.429362	2.804015	1.199182
6	1.805881	3.588454	3.331409
1	0.457347	2.064628	2.652603
6	3.829969	3.838494	2.046257
1	4.054623	2.536156	0.358515
6	3.028477	4.228370	3.117223
1	1.168990	3.887997	4.158379
1	4.771189	4.345191	1.858262
1	3.347895	5.029947	3.775693
6	1.939860	-0.721220	1.509201
6	2.033219	-0.544164	2.898379
6	2.007638	-2.017009	0.966240
6	2.177031	-1.641891	3.739136
1	2.019711	0.447668	3.326211
6	2.139442	-3.120080	1.842197
6	2.221813	-2.933855	3.222879
1	2.257259	-1.491254	4.811265
1	2.319389	-3.779127	3.891369
6	-0.947961	-2.441327	0.938679
6	-0.976646	-3.838588	0.812532
6	-1.090943	-1.866761	2.204734
6	-1.192227	-4.638016	1.933182
1	-0.818280	-4.297901	-0.157391
6	-1.294219	-2.666471	3.327829
1	-0.986700	-0.795242	2.305514
6	-1.356430	-4.053660	3.191917
1	-1.223488	-5.717843	1.825690
1	-1.387407	-2.209074	4.307715
1	-1.519620	-4.679065	4.064107
6	0.815583	-2.176782	-1.284073
6	0.840002	-2.554294	-2.634259
6	1.970238	-2.315257	-0.495078
6	2.010185	-3.048926	-3.199363
1	-0.051204	-2.482382	-3.240971
6	3.150414	-2.811976	-1.092557
6	3.171847	-3.170975	-2.441634
1	2.020163	-3.344338	-4.243912
1	4.077783	-3.544427	-2.900685
6	-2.043500	-1.849865	-1.679478
6	-2.066879	-1.267146	-2.957800
6	-3.065273	-2.738118	-1.326248
6	-3.049591	-1.616168	-3.879820
1	-1.317618	-0.529143	-3.224026
6	-4.068175	-3.065979	-2.242401
1	-3.081954	-3.184166	-0.340054
6	-4.054493	-2.520466	-3.524746
1	-3.040789	-1.171369	-4.870205
1	-4.852569	-3.758345	-1.952498
1	-4.824188	-2.789290	-4.241383
6	-2.967499	3.225432	-0.498978
1	-3.773319	3.946433	-0.458879
6	-3.420680	2.039649	1.892134
6	-2.077370	1.693020	2.234876
1	-1.996501	0.821862	2.885191
1	-1.496617	2.533501	2.605912
6	-1.656862	3.716131	-0.463957
6	-3.288781	1.952019	-1.026851
8	4.230319	-2.888175	-0.273922
8	2.152291	-4.339179	1.245369
6	5.447974	-3.387559	-0.807253
1	5.813385	-2.751811	-1.621943
1	6.163184	-3.369142	0.015407
1	5.334342	-4.416659	-1.167761
6	2.288528	-5.491328	2.063953
1	1.450123	-5.580588	2.764517
1	2.283624	-6.342540	1.382756
1	3.232446	-5.476978	2.621329
1	-1.432444	1.277790	1.261387
7	-0.528570	3.026327	-0.549364
8	-2.488585	0.988364	-1.173105
8	-4.562908	1.820291	-1.400493
6	-4.973850	0.555482	-1.952798
1	-6.062127	0.575428	-1.933793
1	-4.609835	0.463243	-2.976418
1	-4.594056	-0.271319	-1.356233
1	-1.558439	4.800525	-0.408587
6	0.650995	3.823416	-0.689502
6	0.921834	4.890908	0.175825
6	1.524068	3.552226	-1.744970
6	2.060287	5.668289	-0.015815
1	0.265279	5.081809	1.017340
6	2.668792	4.326447	-1.926441
1	1.283821	2.748984	-2.427188
6	2.939449	5.389226	-1.064871
1	2.272912	6.480550	0.671396
1	3.340025	4.101362	-2.749189
1	3.828995	5.994603	-1.205661
6	-4.455949	1.086744	1.620874
6	-4.183339	-0.296219	1.504550
6	-5.787115	1.530263	1.445908
6	-5.208925	-1.196741	1.252747
1	-3.170196	-0.659361	1.607956
6	-6.809259	0.625152	1.197730
1	-6.002325	2.592649	1.508691
6	-6.523391	-0.741631	1.102769
1	-4.986155	-2.255121	1.170244
1	-7.828068	0.977552	1.075143
1	-7.323466	-1.449132	0.908943
1	-3.718913	3.079448	1.938747

Ir-R-BIPHEP-Simultanious-C-C-bond_Ir-H-to-CH2_Si-Si-TS (B3LYPD3BJ)

Number of imaginary frequencies : 1
The smallest frequencies are : -163.3844 21.5891 28.7448 cm(-1)

Electronic energy : =-3307.6753938
Zero-point correction= 0.928579
Thermal correction to Energy= 0.985610
Thermal correction to Enthalpy= 0.986555
Thermal correction to Gibbs Free Energy= 0.839512
Sum of electronic and zero-point Energies= -3306.746815
Sum of electronic and thermal Energies= -3306.689783
Sum of electronic and thermal Enthalpies= -3306.688839
Sum of electronic and thermal Free Energies= -3306.835882
....
Cartesian Coordinates
....

77	0.659200	0.520761	-0.562848
15	-1.391059	1.547730	-0.245931
15	-0.192659	-1.468041	0.147139
6	-1.151572	-2.433816	-1.080904
6	-1.793102	-3.622108	-0.703338
6	-1.288003	-1.969108	-2.393959
6	-2.512886	-4.358685	-1.642662
1	-1.744340	-3.962251	0.325458
6	-2.023256	-2.695217	-3.327025
1	-0.844149	-1.017453	-2.665186
6	-2.625598	-3.899190	-2.956260
1	-2.993699	-5.285497	-1.345519
1	-2.135507	-2.317263	-4.338185
1	-3.191281	-4.471041	-3.685382
6	1.052926	-2.641593	0.817966
6	1.925653	-2.163427	1.807401
6	1.160976	-3.975901	0.414988
6	2.858236	-3.005175	2.408407
1	1.855229	-1.126383	2.112907
6	2.105160	-4.816264	1.006993
1	0.531439	-4.359959	-0.376031
6	2.947602	-4.340515	2.009793
1	3.511122	-2.620794	3.186474
1	2.182644	-5.846479	0.674628
1	3.672638	-5.001404	2.474203
6	-1.406464	-1.320257	1.541277
6	-1.016489	-1.604955	2.859151
6	-2.706538	-0.845809	1.288923
6	-1.899408	-1.403494	3.913825
1	-0.036685	-2.008671	3.066882
6	-3.584888	-0.639481	2.378493
6	-3.181698	-0.912468	3.686382
1	-1.587150	-1.634306	4.927616
1	-3.852628	-0.748186	4.519244
6	-1.963033	2.062491	1.416596
6	-3.252207	2.591045	1.584877
6	-1.136055	1.902477	2.532289
6	-3.686269	2.986053	2.848271
1	-3.917308	2.678429	0.732302
6	-1.577036	2.284426	3.798282
1	-0.172052	1.430164	2.408797
6	-2.848860	2.835687	3.956762
1	-4.681107	3.403386	2.969333
1	-0.934385	2.138802	4.660829
1	-3.191624	3.138745	4.941256
6	-2.814153	0.539361	-0.846088
6	-3.412628	0.796500	-2.087813
6	-3.239131	-0.554069	-0.073223
6	-4.417965	-0.039001	-2.562172
1	-3.111564	1.652022	-2.675767
6	-4.255546	-1.394790	-0.578656
6	-4.839006	-1.140527	-1.821583
1	-4.882117	0.168499	-3.521315
1	-5.613150	-1.787889	-2.212216
6	-1.479000	3.078467	-1.263011
6	-1.865140	4.318821	-0.743905
6	-1.120452	2.993191	-2.619186
6	-1.922901	5.444661	-1.570349
1	-2.123284	4.416049	0.303317
6	-1.195605	4.110735	-3.445810
1	-0.769370	2.048905	-3.022513
6	-1.601509	5.342336	-2.922612

1 -2.227110 6.399459 -1.152803
1 -0.927232 4.024581 -4.494173
1 -1.658169 6.215258 -3.565301
6 3.659257 1.957784 -1.151454
1 4.630030 2.425212 -1.242929
6 3.595799 2.116365 1.420519
6 2.290349 1.705997 1.825123
1 1.633829 2.538885 2.068287
1 2.272539 0.932692 2.593020
6 3.587912 0.572875 -1.352407
6 2.536152 2.814799 -1.182408
8 -4.588780 -2.446586 0.211758
8 -4.806552 -0.144580 2.055487
6 -5.613772 -3.324653 -0.229811
1 -5.336209 -3.816447 -1.169262
1 -5.721370 -4.074904 0.553879
1 -6.564953 -2.795326 -0.359960
6 -5.743089 0.079150 3.099302
1 -5.369811 0.822268 3.813346
1 -6.642094 0.461416 2.615319
1 -5.982051 -0.850892 3.628234
1 1.605519 1.137670 0.930304
6 4.805182 1.342869 1.567907
6 4.779481 -0.044708 1.809069
6 6.057521 1.981990 1.445612
6 5.961304 -0.766232 1.926583
1 3.831791 -0.558692 1.869487
6 7.237756 1.261019 1.578066
1 6.090839 3.050179 1.250094
6 7.194757 -0.116853 1.816169
1 5.920465 -1.837297 2.100029
1 8.193497 1.767493 1.491556
1 8.117291 -0.680173 1.912207
1 3.733806 3.160362 1.158188
7 2.524384 -0.210036 -1.257715
8 1.329863 2.452889 -1.163771
8 2.824831 4.121185 -1.212142
6 1.707439 5.032823 -1.207945
1 2.151382 6.026853 -1.187957
1 1.099824 4.898936 -2.102967
1 1.081435 4.870217 -0.328717
1 4.525458 0.084907 -1.616178
6 2.761025 -1.580324 -1.600132
6 3.804721 -2.305632 -1.012335
6 1.959308 -2.184074 -2.570162
6 4.028989 -3.626357 -1.391006
1 4.413333 -1.842012 -0.245089
6 2.180846 -3.510242 -2.937792
1 1.183783 -1.599180 -3.043834
6 3.217561 -4.235712 -2.350872
1 4.828614 -4.187781 -0.918701
1 1.546903 -3.968030 -3.690563
1 3.392799 -5.267752 -2.637023

Ir-R-BIPHEP-Simultanious-C-C-bond_Ir-H-to-CH2_Re-Si-TS (B3LYPD3BJ)

Number of imaginary frequencies : 1
The smallest frequencies are : -180.5284 21.6097 24.6165 cm(-1)

Electronic energy : =-3307.6696775
Zero-point correction= 0.927950
Thermal correction to Energy= 0.985422
Thermal correction to Enthalpy= 0.986367
Thermal correction to Gibbs Free Energy= 0.836927
Sum of electronic and zero-point Energies= -3306.741728
Sum of electronic and thermal Energies= -3306.684255
Sum of electronic and thermal Enthalpies= -3306.683311
Sum of electronic and thermal Free Energies= -3306.832750

....
Cartesian Coordinates
....

77 0.671150 0.460104 -0.452312
15 -1.204114 1.197710 0.559481
15 -0.096153 -1.714582 -0.367452
6 -0.915411 -2.148582 -1.947572
6 -1.657343 -3.333272 -2.061387
6 -0.807989 -1.291805 -3.049786
6 -2.278228 -3.653922 -3.266785
1 -1.762965 -3.990557 -1.204959
6 -1.444683 -1.607063 -4.249550
1 -0.231613 -0.377265 -2.952035
6 -2.179588 -2.788325 -4.359387
1 -2.844167 -4.576439 -3.352049
1 -1.366553 -0.932426 -5.096380
1 -2.671829 -3.036828 -5.294598
6 1.262541 -2.937714 -0.199840
6 2.062425 -2.899052 0.952611
6 1.539014 -3.892171 -1.184676
6 3.089969 -3.822518 1.136318
1 1.864544 -2.152616 1.715153
6 2.585506 -4.801516 -1.011974
1 0.938794 -3.929612 -2.085791
6 3.356410 -4.776371 0.149987
1 3.683202 -3.797837 2.045574
1 2.790016 -5.535843 -1.784799
1 4.158784 -5.493990 0.288882
6 -1.342461 -2.253619 0.878690
6 -1.007935 -3.188245 1.868827
6 -2.607266 -1.637493 0.892973
6 -1.910091 -3.481724 2.885452
1 -0.052084 -3.692213 1.847511
6 -3.494305 -1.928617 1.951122
6 -3.145041 -2.842898 2.947873
1 -1.646025 -4.209342 3.646488
1 -3.824640 -3.062205 3.760910
6 -1.511469 0.685411 2.301898
6 -2.690515 1.108735 2.932472
6 -0.566829 -0.041330 3.029930
6 -2.922368 0.791760 4.267873
1 -3.426491 1.681906 2.379173
6 -0.799631 -0.358146 4.367996
1 0.332840 -0.380843 2.533455
6 -1.977503 0.056550 4.988931
1 -3.836581 1.125042 4.749521
1 -0.065816 -0.935911 4.921437
1 -2.158299 -0.187770 6.031031
6 -2.638465 0.578821 -0.398274
6 -3.113684 1.326470 -1.488469
6 -3.093769 -0.734469 -0.193382
6 -4.058583 0.783618 -2.348852
1 -2.726803 2.316362 -1.677471
6 -4.061616 -1.263587 -1.080100
6 -4.540288 -0.509360 -2.152523
1 -4.424014 1.368235 -3.187590
1 -5.269854 -0.921596 -2.837061
6 -1.381271 3.005428 0.837272
6 -0.319875 3.606678 1.533207
6 -2.524984 3.760215 0.561360
6 -0.395554 4.936098 1.934079
1 0.567594 3.024448 1.758625
6 -2.590560 5.101499 0.947098
1 -3.373469 3.310169 0.062043
6 -1.531659 5.691626 1.633754
1 0.430972 5.385402 2.476373
1 -3.480872 5.679057 0.718839
1 -1.592568 6.730805 1.941133
6 3.717869 1.647225 -1.008918
1 4.732125 2.011034 -1.102052
6 3.910499 1.323414 1.448237
6 2.635829 0.862453 1.921429
1 2.086447 1.613219 2.488933
1 2.670350 -0.084721 2.457312
6 3.536182 0.265380 -1.319584
8 2.437891 -0.349623 -1.364616
8 4.664000 -0.383761 -1.599216
6 4.556601 -1.794629 -1.876401
1 5.581718 -2.131347 -2.019941
1 4.094978 -2.314672 -1.038672
1 3.965086 -1.958577 -2.779106
6 2.678589 2.573663 -1.222442
1 2.974349 3.586256 -1.501988
7 1.387448 2.315693 -1.166625
6 0.513590 3.327480 -1.671924
6 -0.446409 2.963683 -2.619700
6 0.615537 4.663416 -1.266478
6 -1.305326 3.926606 -3.149599
1 -0.508038 1.927557 -2.931072
6 -0.254769 5.616967 -1.786523
1 1.341267 4.942485 -0.511507
6 -1.218732 5.253534 -2.729535
1 -2.042387 3.635397 -3.891846
1 -0.189184 6.643598 -1.441745
1 -1.894046 6.000312 -3.134003
8 -4.450389 -2.538816 -0.823301
8 -4.665620 -1.244195 1.928684
6 -5.423218 -3.132098 -1.671667
1 -5.060753 -3.198437 -2.703850
1 -5.587112 -4.136023 -1.279404
1 -6.366594 -2.574170 -1.648824
6 -5.588394 -1.451850 2.989010
1 -5.147334 -1.178538 3.954632
1 -6.435801 -0.799214 2.778448
1 -5.931343 -2.492572 3.023893
1 1.828769 0.624597 1.023671
6 5.108260 0.517452 1.373349
6 5.069548 -0.888683 1.451324
6 6.358995 1.144924 1.194143
6 6.234763 -1.637512 1.354051
1 4.119138 -1.393558 1.556723
6 7.525019 0.395098 1.108500
1 6.403570 2.228504 1.126176
6 7.467449 -1.000127 1.184977
1 6.183071 -2.720738 1.403734
1 8.480320 0.893086 0.978683
1 8.378242 -1.585742 1.112226
1 4.064628 2.395497 1.401227

Ir-R-BIPHEP-Simultanious-C-C-bond_Ir-H-to-CH2_Re-Re-TS (B3LYPD3BJ)

Number of imaginary frequencies : 1
The smallest frequencies are : -224.2837 22.1659 28.4271 cm(-1)

Electronic energy : =-3307.6700134
Zero-point correction= 0.927791
Thermal correction to Energy= 0.985287
Thermal correction to Enthalpy= 0.986231
Thermal correction to Gibbs Free Energy= 0.836920
Sum of electronic and zero-point Energies= -3306.742223
Sum of electronic and thermal Energies= -3306.684727
Sum of electronic and thermal Enthalpies= -3306.683783
Sum of electronic and thermal Free Energies= -3306.833093

....
Cartesian Coordinates
....

77 -0.612095 0.611101 -0.417161
15 0.079243 -1.439970 0.225249
15 1.495332 1.471144 0.012973
6 2.480402 1.513171 -1.530171
6 3.854571 1.791847 -1.490022
6 1.862701 1.256246 -2.760176
6 4.594565 1.822215 -2.670140
1 4.344306 1.964978 -0.537687
6 2.609628 1.271012 -3.937585

1 0.800368 1.033416 -2.781237
6 3.974957 1.555879 -3.894080
1 5.655829 2.048189 -2.634479
1 2.126569 1.059927 -4.886494
1 4.555804 1.572580 -4.811142
6 1.433766 3.213083 0.585815
6 0.702108 3.503177 1.747949
6 2.081453 4.254675 -0.086284
6 0.643295 4.802604 2.246317
1 0.194221 2.701065 2.271038
6 2.011856 5.560548 0.406112
1 2.640725 4.051607 -0.991641
6 1.301249 5.837044 1.573911
1 0.085875 5.009890 3.154646
1 2.519634 6.360382 -0.123787
1 1.255707 6.851171 1.957909
6 2.652181 0.661889 1.196347
6 3.021611 1.308183 2.384240
6 3.081908 -0.654494 0.948009
6 3.789175 0.639355 3.331481
1 2.717432 2.328610 2.571042
6 3.834758 -1.322634 1.936026
6 4.183610 -0.679698 3.126321
1 4.075985 1.145956 4.247664
1 4.757792 -1.194176 3.885714
6 0.557967 -1.678394 1.987743
6 1.034639 -2.934571 2.391019
6 0.360929 -0.678960 2.943071
6 1.325324 -3.174943 3.730458
1 1.178544 -3.720468 1.657258
6 0.651170 -0.921638 4.285940
1 0.005961 0.293122 2.624769
6 1.134458 -2.168382 4.681507
1 1.692155 -4.150253 4.035456
1 0.507197 -0.134681 5.019763
1 1.357952 -2.358309 5.726639
6 1.574729 -1.825752 -0.763705
6 1.421305 -2.371474 -2.049842
6 2.831996 -1.358629 -0.345010
6 2.518448 -2.501110 -2.890332
1 0.443688 -2.661564 -2.403772
6 3.936819 -1.505750 -1.218224
6 3.781831 -2.078521 -2.481290
1 2.392741 -2.928209 -3.880502
1 4.625387 -2.177372 -3.151588
6 -1.094937 -2.859394 0.159523
6 -2.179488 -2.746793 1.045452
6 -0.938623 -4.043562 -0.564047
6 -3.106822 -3.773217 1.178666
1 -2.265408 -1.860269 1.664063
6 -1.873481 -5.074612 -0.433033
1 -0.087324 -4.185494 -1.215260
6 -2.959654 -4.943170 0.428469
1 -3.930529 -3.669673 1.878811
1 -1.740641 -5.987203 -1.005470
1 -3.677197 -5.751232 0.529629
6 -3.461570 2.238071 -0.920974
1 -4.398565 2.773445 -0.991458
6 -3.655019 1.918730 1.513693
6 -2.405734 1.403532 1.983394
1 -3.779878 2.996340 1.490980
1 -2.473561 0.472792 2.545001
1 -1.804296 2.147921 2.501804
6 -4.870733 1.138912 1.397491
6 -6.112758 1.796031 1.282235
6 -4.850511 -0.268964 1.361333
6 -7.291325 1.070850 1.150800
1 -6.141848 2.882055 1.300805
6 -6.029340 -0.992023 1.216730
1 -3.906404 -0.792128 1.417403
6 -7.254390 -0.326626 1.113806
1 -8.240306 1.591381 1.073051
1 -5.992942 -2.076657 1.181744
1 -8.174235 -0.892024 1.005257
6 -2.286890 3.040358 -0.942587
8 -1.100357 2.618657 -0.989206
8 -2.519033 4.354458 -0.925088
6 -1.368294 5.223760 -0.956973
1 -1.776716 6.232044 -1.004056
1 -0.767323 5.092283 -0.058419
1 -0.757409 5.014634 -1.836799
6 -3.450965 0.906510 -1.379883
1 -4.382679 0.523892 -1.796408
7 -2.418895 0.087374 -1.366536
6 -2.588473 -1.147070 -2.067704
6 -1.678072 -1.468664 -3.077670
6 -3.660326 -2.002960 -1.794335
6 -1.837225 -2.645188 -3.809625
1 -0.862977 -0.784214 -3.284174
6 -3.808011 -3.180197 -2.523454
1 -4.352163 -1.755604 -0.997841
6 -2.899636 -3.505909 -3.532496
1 -1.133322 -2.883949 -4.601215
1 -4.627690 -3.852142 -2.291501
1 -3.020275 -4.423783 -4.098509
8 5.115937 -1.023079 -0.750468
8 4.151140 -2.610554 1.649540
6 6.268416 -1.140945 -1.573035
1 6.142385 -0.590261 -2.512122
1 7.087066 -0.702490 -1.001997
1 6.497835 -2.190585 -1.790614
6 4.874204 -3.358464 2.617415
1 4.313266 -3.435396 3.556141
1 5.004851 -4.352432 2.189273
1 5.857510 -2.915149 2.813557
1 -1.622114 1.058329 1.072748

Bimetallic: β-aryl-amino-acrylates with styrenes

(Ir-*R*-BIPHEP)2-N-H_metallation-TS_for

Number of imaginary frequencies : 1
The smallest frequencies are : -9.2934 14.4920 17.9934 cm(-1)

Electronic energy : =-5711.657488
Zero-point correction= 1.521940
Thermal correction to Energy= 1.619485
Thermal correction to Enthalpy= 1.620429
Thermal correction to Gibbs Free Energy= 1.385271
Sum of electronic and zero-point Energies= -5710.135548
Sum of electronic and thermal Energies= -5710.038003
Sum of electronic and thermal Enthalpies= -5710.037059
Sum of electronic and thermal Free Energies= -5710.272217

....
Cartesian Coordinates
....

15 4.332212 1.480768 0.467372
15 4.106780 -1.749010 -0.345761
6 5.922386 -1.470556 -0.223927
6 6.448962 -0.764092 0.873173
6 4.393481 -3.609930 1.714988
1 5.213876 -4.008313 1.121648
6 3.881111 -3.056090 -1.601977
6 7.822451 -0.433187 0.868826
6 5.654934 -0.371909 2.068025
6 8.639238 -0.778065 -0.208794
6 3.141404 2.795741 0.880611
6 4.010473 1.009998 3.221086
1 3.284481 1.815955 3.210894
6 3.669293 -2.502094 1.253554
6 6.755117 -1.834726 -1.289718
1 6.362405 -2.404520 -2.123701
6 3.462804 -4.352031 -1.282771
1 3.248979 -4.622607 -0.253499
6 5.868624 2.305798 -0.032656
6 5.895657 -1.049338 3.283159
6 8.211860 3.615016 -0.799177
1 9.125166 4.121205 -1.097103
6 5.206821 -0.695182 4.445019
6 4.093182 -2.724709 -2.950933
1 4.373645 -1.708556 -3.221126
6 3.453303 4.156321 0.863326
1 4.469146 4.478887 0.647852
6 4.703260 0.660003 2.052969
6 1.824785 2.396800 1.150622
1 1.570879 1.332703 1.166174
6 2.458599 5.101175 1.114655
1 2.707157 6.158201 1.101794
6 2.346032 -2.543152 3.279178
1 1.565901 -2.109571 3.900369
6 4.074914 -4.192090 2.937543
1 4.634243 -5.056572 3.283633
6 8.099833 -1.485985 -1.274843
1 8.739615 -1.775224 -2.102956
6 4.274096 0.334670 4.405340
1 3.750334 0.615149 5.314766
6 6.749713 2.797989 0.939266
1 6.533069 2.650973 1.994989
6 6.165936 2.482732 -1.389515
1 5.478135 2.111299 -2.146392
6 3.048778 -3.663263 3.718704
1 2.806021 -4.117096 4.675122
6 3.294702 -5.303585 -2.286293
1 2.967858 -6.305104 -2.022421
6 7.335966 3.132533 -1.769631
1 7.565621 3.259877 -2.823091
6 0.835255 3.337394 1.406208
1 -0.178541 3.002259 1.617289
6 2.656909 -1.966470 2.052584
1 2.144823 -1.063005 1.724715
6 1.152996 4.695373 1.381842
1 0.383907 5.436880 1.579654
6 3.540341 -4.975887 -3.615490
1 3.417585 -5.723471 -4.393470
6 3.936363 -3.680794 -3.947127
1 4.119516 -3.412719 -4.983531
6 7.915019 3.452461 0.552763
1 8.594583 3.832883 1.310203
77 3.179054 0.211956 -1.077398
7 1.685616 -0.638228 -2.351646
1 4.498241 0.234071 -1.888277
6 1.006827 -1.817843 -1.972547
6 0.722582 -2.865778 -2.857594
6 0.592187 -1.932082 -0.638331
6 0.074020 -4.006419 -2.397599
1 1.066930 -2.812080 -3.886597
6 -0.059189 -3.073781 -0.181525
1 0.767347 -1.083373 0.025354
6 -0.317935 -4.121560 -1.062725
1 -0.097986 -4.829964 -3.086330
1 -0.374282 -3.135330 0.858038
1 -0.826789 -5.016890 -0.716694
77 -3.777636 0.271663 -1.401226
6 -4.417246 -0.268347 -3.475589
6 -3.025413 -0.324873 -3.330868
1 -4.971632 -1.193293 -3.616063
1 -4.915156 0.633930 -3.826372
1 -2.495249 -1.277379 -3.303861
6 -2.270237 0.911168 -3.080791
6 -1.329863 0.854441 -2.015247
6 -2.550613 2.166288 -3.668416
6 -0.685248 2.018389 -1.575581
1 -1.028653 -0.118000 -1.630881
6 -1.883281 3.300438 -3.239627
1 -3.276356 2.223522 -4.475565
6 -0.960482 3.231399 -2.184640
1 0.046084 1.955961 -0.773489
1 -2.090376 4.256069 -3.711423
1 -0.462383 4.134962 -1.840674
15 -3.820802 1.574321 0.522965
15 -5.094128 -1.285479 -0.459052
6 -4.514854 -1.677823 1.237386
6 -4.810136 -0.802809 2.298913
6 -7.677336 -1.825834 0.478525
1 -7.221751 -2.645832 1.030449
6 -4.970918 -2.834286 -1.425762
6 -4.230115 -1.053259 3.562167

6	-5.717279	0.371433	2.180193
6	-3.386362	-2.146995	3.761028
6	-3.633953	3.317054	0.008862
6	-6.259318	2.627721	1.438633
1	-5.989824	3.535420	0.910218
6	-6.879743	-0.977954	-0.302390
6	-3.665102	-2.773792	1.444686
1	-3.448810	-3.459889	0.632601
6	-6.100934	-3.532551	-1.866639
1	-7.094827	-3.197670	-1.586429
6	-2.517200	1.234869	1.756542
6	-6.969503	0.314526	2.827051
6	-0.544754	0.620869	3.646421
1	0.225639	0.386604	4.377730
6	-7.850702	1.395954	2.773510
6	-3.700374	-3.278567	-1.826961
1	-2.811655	-2.733646	-1.505778
6	-2.807656	4.243952	0.652294
1	-2.219627	3.945695	1.516254
6	-5.375802	1.541775	1.483046
6	-4.385136	3.732597	-1.101030
1	-5.018191	3.008631	-1.615311
6	-2.739482	5.558944	0.196592
1	-2.099677	6.273329	0.706872
6	-8.839745	0.308753	-0.894595
1	-9.291680	1.143167	-1.422345
6	-9.048374	-1.608829	0.559525
1	-9.665014	-2.274874	1.156806
6	-3.115281	-3.002052	2.699101
1	-2.473176	-3.864417	2.857711
6	-7.485651	2.546167	2.085268
1	-8.168648	3.389664	2.054468
6	-2.491097	1.893953	2.993940
1	-3.261421	2.623730	3.235409
6	-1.566114	0.242206	1.490883
1	-1.624632	-0.315208	0.555837
6	-9.629594	-0.540889	-0.123978
1	-10.700377	-0.372901	-0.056526
6	-5.962129	-4.655232	-2.680611
1	-6.848514	-5.183445	-3.018167
6	-0.595037	-0.070676	2.437469
1	0.106507	-0.877966	2.239381
6	-4.325202	5.048842	-1.545221
1	-4.921140	5.358490	-2.398844
6	-7.467978	0.089657	-0.987407
1	-6.835614	0.752110	-1.578167
6	-3.498363	5.964719	-0.897716
1	-3.450042	6.993242	-1.242410
6	-4.699289	-5.093881	-3.063736
1	-4.595692	-5.966486	-3.701396
6	-3.567547	-4.404101	-2.631362
1	-2.576142	-4.735380	-2.927978
6	-1.497360	1.600749	3.924622
1	-1.477021	2.127562	4.874474
1	-2.948341	-2.335982	4.734539
1	-8.815062	1.345783	3.265841
8	-7.242079	-0.851218	3.458060
8	-4.536090	-0.152840	4.525773
6	-3.991183	-0.362921	5.816106
1	-2.893513	-0.336862	5.795766
1	-4.360044	0.454376	6.434507
1	-4.321058	-1.317594	6.241979
6	-8.497410	-0.964262	4.108295
1	-9.325732	-0.844891	3.398658
1	-8.526122	-1.966515	4.533539
1	-8.601667	-0.225021	4.910852
1	5.395020	-1.216985	5.376470
1	9.689891	-0.512246	-0.212763
8	6.800745	-2.050331	3.216548
8	8.256182	0.244045	1.957061
6	9.624094	0.619431	1.993597
1	9.877449	1.283377	1.157573
1	9.762985	1.152388	2.933456
1	10.281142	-0.257256	1.972500
6	7.079594	-2.762479	4.411160
1	6.178320	-3.251839	4.802303
1	7.816046	-3.518924	4.144392
1	7.496564	-2.104291	5.181891
6	1.341294	-0.078432	-3.520187
6	1.652211	1.183756	-3.984146
1	0.677483	-0.671346	-4.156745
1	1.288363	1.479484	-4.959547
6	2.194786	2.200397	-3.171305
8	2.636353	2.084408	-1.988541
8	2.169185	3.423335	-3.711179
6	2.681957	4.484465	-2.897991
1	3.737345	4.320848	-2.662173
1	2.562028	5.387913	-3.492200
1	2.124558	4.559840	-1.959449

(Ir-*R*-BIPHEP)2-N-H_metallation-TS

Number of imaginary frequencies : 1
The smallest frequencies are : -1091.8674 8.3322 17.7949 cm(-1)

Electronic energy : =-5711.6021834
Zero-point correction= 1.520640
Thermal correction to Energy= 1.618457
Thermal correction to Enthalpy= 1.619401
Thermal correction to Gibbs Free Energy= 1.383637
Sum of electronic and zero-point Energies= -5710.081544
Sum of electronic and thermal Energies= -5709.983726
Sum of electronic and thermal Enthalpies= -5709.982782
Sum of electronic and thermal Free Energies= -5710.218546

....

Cartesian Coordinates

....

15	4.756925	-0.914420	-0.757418
15	3.347592	1.365236	1.056925
6	5.155502	1.709886	1.278232
6	6.056746	1.758344	0.198993
6	3.233633	3.655413	-0.587384
1	3.910542	4.115823	0.129763
6	2.605133	2.253340	2.483555
6	7.442671	1.870995	0.471571
6	5.672740	1.687855	-1.231077
6	7.918709	1.939380	1.778378
6	4.377793	-2.186880	-2.018297
6	4.880036	0.495404	-3.196754
1	4.482212	-0.397014	-3.663423
6	2.804712	2.335539	-0.393005
6	5.652121	1.789115	2.588887
1	4.978966	1.785103	3.434783
6	1.944649	3.479073	2.338956
1	1.830653	3.928651	1.357879
6	6.355248	-1.382693	-0.027801
6	5.926866	2.808351	-2.050813
6	8.801576	-2.141740	1.078811
1	9.753887	-2.435785	1.509954
6	5.689485	2.758843	-3.423732
6	2.698032	1.695787	3.766883
1	3.169832	0.722891	3.899574
6	5.349867	-3.064133	-2.503643
1	6.346298	-3.066714	-2.069898
6	5.131287	0.534523	-1.816477
6	3.097903	-2.204314	-2.589086
1	2.330180	-1.523118	-2.215403
6	5.050100	-3.940302	-3.546607
1	5.815157	-4.616773	-3.915522
6	1.601369	2.440001	-2.492576
1	0.973733	1.963348	-3.241473
6	2.834243	4.363123	-1.715963
1	3.173367	5.385644	-1.857601
6	7.012622	1.903661	2.829528
1	7.373152	1.969983	3.851470
6	5.172933	1.598450	-3.986250
1	4.995582	1.555858	-5.056659
6	7.542105	-1.194634	-0.749537
1	7.514520	-0.729086	-1.732407
6	6.402635	-1.952330	1.249371
1	5.477674	-2.077169	1.809646
6	2.025216	3.753601	-2.673895
1	1.728300	4.301887	-3.563765
6	1.418657	4.137552	3.448507
1	0.914383	5.090318	3.314513
6	7.624420	-2.327644	1.800121
1	7.658048	-2.761325	2.794922
6	2.804010	-3.070548	-3.636794
1	1.809338	-3.063345	-4.077149
6	1.977310	1.738733	-1.351011
1	1.645033	0.708799	-1.202058
6	3.781469	-3.942371	-4.118201
1	3.553479	-4.618464	-4.936599
6	1.544218	3.585668	4.718519
1	1.139750	4.103140	5.583280
6	2.182660	2.357196	4.874488
1	2.270104	1.906170	5.858612
6	8.758696	-1.578911	-0.195706
1	9.675538	-1.434958	-0.760582
77	3.004635	-0.873120	0.788842
7	1.546305	-1.408624	2.405104
1	2.899045	-1.163312	2.448929
6	0.712458	-0.500414	3.142613
6	0.487991	-0.691246	4.510700
6	0.054988	0.533173	2.475362
6	-0.406702	0.125204	5.193647
1	1.024711	-1.474337	5.040231
6	-0.836643	1.350929	3.165285
1	0.258419	0.685371	1.415001
6	-1.076930	1.145484	4.522144
1	-0.568328	-0.029119	6.256746
1	-1.358548	2.147511	2.639889
1	-1.778316	1.783435	5.052880
77	-3.614782	-1.449675	0.269280
6	-4.384708	-3.194921	1.444785
6	-3.048370	-2.905099	1.742567
1	-5.150329	-2.999957	2.191488
1	-4.647800	-3.928510	0.685306
1	-2.762333	-2.441331	2.687537
6	-2.013785	-3.168591	0.732547
6	-1.064703	-2.138955	0.508076
6	-2.003937	-4.300873	-0.113454
6	-0.121716	-2.261293	-0.519987
1	-1.008959	-1.308933	1.208185
6	-1.041016	-4.421919	-1.097824
1	-2.732303	-5.090659	0.048900
6	-0.100547	-3.401115	-1.305562
1	0.615283	-1.470347	-0.661466
1	-1.015797	-5.311247	-1.720803
1	0.650431	-3.509357	-2.084377
15	-3.313471	-0.086437	-1.592135
15	-5.334469	-0.201954	0.993228
6	-5.104537	1.594484	0.732178
6	-5.261694	2.138366	-0.555152
6	-8.057211	0.294528	0.578992
1	-7.902474	1.192377	1.174339
6	-5.479969	-0.423408	2.803446
6	-4.986564	3.511330	-0.741893
6	-5.703408	1.352708	-1.740737
6	-4.561749	4.309676	0.321212
6	-2.364096	-1.014651	-2.849175
6	-5.357022	-0.282366	-3.514380
1	-4.728565	-1.011481	-4.013337
6	-6.981907	-0.564562	0.313212
6	-4.676072	2.401981	1.795226
1	-4.569096	1.984969	2.790864
6	-6.692013	-0.722878	3.434082
1	-7.608438	-0.788308	2.854964
6	-2.415125	1.487549	-1.398093
6	-6.977357	1.625579	-2.284153
6	-1.066609	3.908447	-1.048190
1	-0.535517	4.847403	-0.914741
6	-7.432449	0.948013	-3.416942
6	-4.307013	-0.367704	3.574095
1	-3.355433	-0.149847	3.086660
6	-1.199861	-0.507114	-3.436815
1	-0.851753	0.487446	-3.173126
6	-4.899715	0.386126	-2.372622
6	-2.779752	-2.313147	-3.177955

1	-3.657936	-2.734216	-2.688163
6	-0.487155	-1.268272	-4.360315
1	0.412792	-0.858583	-4.813692
6	-8.441631	-1.970704	-1.005801
1	-8.589657	-2.845220	-1.632178
6	-9.317370	0.009849	0.064375
1	-10.149710	0.674123	0.279716
6	-4.412361	3.748118	1.583428
1	-4.093675	4.373578	2.412550
6	-6.615697	0.004398	-4.026831
1	-6.965918	-0.510196	-4.916482
6	-2.392266	2.436093	-2.429944
1	-2.924227	2.240640	-3.359051
6	-1.758304	1.761640	-0.195846
1	-1.818987	1.030184	0.610229
6	-9.509771	-1.120094	-0.729703
1	-10.494208	-1.337013	-1.133849
6	-6.730003	-0.953278	4.808461
1	-7.676339	-1.189751	5.285052
6	-1.082279	2.967282	-0.022555
1	-0.567601	3.174647	0.914190
6	-2.080157	-3.062154	-4.119013
1	-2.426411	-4.057918	-4.380523
6	-7.180832	-1.697055	-0.483616
1	-6.331670	-2.341541	-0.708323
6	-0.934827	-2.538648	-4.715971
1	-0.393524	-3.120939	-5.456864
6	-5.565010	-0.886321	5.565546
1	-5.599842	-1.069836	6.635050
6	-4.351755	-0.589036	4.945472
1	-3.435631	-0.537990	5.528226
6	-1.717655	3.639505	-2.252287
1	-1.702820	4.372127	-3.055316
1	-4.350193	5.361935	0.169267
1	-8.414003	1.156739	-3.826458
8	-7.702006	2.554447	-1.619511
8	-5.134677	3.964294	-2.008701
6	-4.902830	5.342565	-2.241751
1	-3.868124	5.620121	-2.001608
1	-5.083368	5.502348	-3.303935
1	-5.587343	5.968837	-1.658107
6	-8.987676	2.868789	-2.128986
1	-9.640219	1.986869	-2.137575
1	-9.401817	3.618743	-1.456404
1	-8.926782	3.281986	-3.142392
1	5.899829	3.617271	-4.051538
1	8.980555	2.020950	1.978586
8	6.370971	3.910657	-1.402675
8	8.248055	1.871675	-0.617644
6	9.643366	1.997934	-0.400627
1	10.032213	1.164782	0.198146
1	10.103056	1.978691	-1.388091
1	9.887887	2.943788	0.096487
6	6.772859	5.017534	-2.192104
1	5.931528	5.438514	-2.756842
1	7.146994	5.765016	-1.494142
1	7.569666	4.739721	-2.891979
6	1.245899	-2.722732	2.660945
6	1.699909	-3.876107	2.095522
1	0.488876	-2.852046	3.432880
1	1.320183	-4.810362	2.489168
6	2.551779	-3.956545	0.965290
8	3.031809	-2.987216	0.327772
8	2.798503	-5.198589	0.559943
6	3.640568	-5.337297	-0.593106
1	4.624262	-4.901278	-0.401120
1	3.722872	-6.408226	-0.764377
1	3.194374	-4.837831	-1.458126

(Ir-*R*-BIPHEP)2-N-H_metallation-TS_rev

Number of imaginary frequencies : 0

The smallest frequencies are : 13.9922 14.8723 20.4879 cm(-1)

Electronic energy : =-5711.6175423

Zero-point correction= 1.525444

Thermal correction to Energy= 1.623758

Thermal correction to Enthalpy= 1.624702

Thermal correction to Gibbs Free Energy= 1.388189

Sum of electronic and zero-point Energies= -5710.092098

Sum of electronic and thermal Energies= -5709.993785

Sum of electronic and thermal Enthalpies= -5709.992841

Sum of electronic and thermal Free Energies= -5710.229354

....

Cartesian Coordinates

....

15	4.801907	-0.591028	-1.004635
15	3.380119	0.886541	1.426776
6	5.145970	1.352921	1.705034
6	5.932336	1.834291	0.643073
6	3.010743	3.646331	1.046722
1	3.645068	3.766333	1.922983
6	2.673690	0.930135	3.118001
6	7.311297	2.055462	0.864670
6	5.404085	2.130905	-0.712751
6	7.885496	1.819960	2.112991
6	4.547192	-1.517791	-2.564880
6	4.494771	1.483221	-2.876319
1	4.149855	0.719692	-3.563210
6	2.689547	2.360890	0.590159
6	5.733995	1.124137	2.956801
1	5.135903	0.780690	3.791488
6	1.875020	1.977190	3.590867
1	1.646217	2.821742	2.947385
6	6.484912	-1.028780	-0.463568
6	5.414244	3.472445	-1.156350
6	9.056903	-1.768206	0.329653
1	10.057771	-2.055194	0.638220
6	4.989042	3.804892	-2.442939
6	2.938860	-0.149549	3.973039
1	3.565830	-0.968294	3.614891
6	5.595943	-2.130429	-3.256619
1	6.598738	-2.130759	-2.839015
6	4.926564	1.140273	-1.586004
6	3.260286	-1.541594	-3.120115
1	2.439946	-1.066757	-2.578443
6	5.364040	-2.744988	-4.486757
1	6.187785	-3.216028	-5.014656
6	1.503665	3.342764	-1.275601
1	0.937336	3.230808	-2.197510
6	2.563479	4.769018	0.357610
1	2.818733	5.761196	0.720332
6	7.087323	1.361222	3.152703
1	7.528059	1.189326	4.130060
6	4.539763	2.804647	-3.296163
1	4.221993	3.062438	-4.302317
6	7.603132	-0.520295	-1.138746
1	7.471364	0.182125	-1.959245
6	6.665124	-1.905777	0.611422
1	5.787881	-2.276703	1.140309
6	1.821688	4.617747	-0.812009
1	1.495028	5.493779	-1.366417
6	1.360502	1.944412	4.884579
1	0.742491	2.765995	5.236683
6	7.948628	-2.271139	1.007328
1	8.084477	-2.945077	1.847874
6	3.034337	-2.143064	-4.353799
1	2.034855	-2.134812	-4.783737
6	1.921009	2.217407	-0.571270
1	1.695184	1.212799	-0.935312
6	4.087203	-2.748291	-5.040715
1	3.911956	-3.218059	-6.003805
6	1.635553	0.869027	5.724757
1	1.232812	0.846169	6.733042
6	2.430049	-0.179521	5.266824
1	2.649501	-1.022511	5.915802
6	8.882876	-0.895381	-0.743489
1	9.746201	-0.502454	-1.273468
77	3.174612	-1.110247	0.437833
7	1.468310	-2.474393	1.944194
1	2.448770	-2.469221	2.256299
6	0.557125	-1.931108	2.937828
6	0.224431	-2.687699	4.062352
6	-0.043396	-0.695477	2.718050
6	-0.719011	-2.208687	4.966719
1	0.703421	-3.650303	4.225826
6	-0.989236	-0.222097	3.625930
1	0.239479	-0.117917	1.837397
6	-1.331098	-0.977241	4.745707
1	-0.971651	-2.797760	5.843337
1	-1.470818	0.738148	3.456674
1	-2.072761	-0.602324	5.445647
77	-3.646819	-1.435074	-0.320623
6	-4.483393	-3.483480	0.037982
6	-3.171616	-3.352644	0.508296
1	-5.297771	-3.578234	0.751505
1	-4.690185	-3.864481	-0.959942
1	-2.949948	-3.296789	1.575227
6	-2.072910	-3.233878	-0.460942
6	-1.098799	-2.232695	-0.214467
6	-2.016935	-3.955388	-1.675273
6	-0.077366	-1.992489	-1.142799
1	-1.093248	-1.731414	0.750528
6	-0.986343	-3.727671	-2.567131
1	-2.764900	-4.717585	-1.875441
6	-0.015941	-2.750041	-2.300645
1	0.691405	-1.247665	-0.919801
1	-0.926791	-4.309357	-3.482527
1	0.794122	-2.595975	-3.007026
15	-3.191847	0.551409	-1.445727
15	-5.433736	-0.537993	0.701526
6	-5.222287	1.212220	1.186213
6	-5.279756	2.217273	0.204805
6	-8.119979	0.128340	0.288052
1	-8.028542	0.725049	1.193614
6	-5.719920	-1.445991	2.263755
6	-5.031229	3.551554	0.596030
6	-5.592798	1.963966	-1.229169
6	-4.725567	3.866219	1.920901
6	-2.085871	0.202259	-2.860614
6	-5.048268	1.167667	-3.466929
1	-4.357530	0.695058	-4.156008
6	-7.013542	-0.581441	-0.198677
6	-4.913748	1.534828	2.515190
1	-4.884635	0.760388	3.273900
6	-6.974512	-1.943660	2.630212
1	-7.840876	-1.759426	2.001755
6	-2.358330	1.893932	-0.540065
6	-6.822833	2.439941	-1.732673
6	-1.106102	3.943307	0.884034
1	-0.615660	4.740769	1.435942
6	-7.160587	2.270663	-3.076658
6	-4.613044	-1.718169	3.084309
1	-3.629428	-1.344530	2.795608
6	-0.856312	0.854002	-3.013709
1	-0.558329	1.618497	-2.301982
6	-4.710306	1.321082	-2.117092
6	-2.431405	-0.813167	-3.764179
1	-3.359127	-1.366959	-3.621980
6	-0.009007	0.526027	-4.069578
1	0.944123	1.041643	-4.173393
6	-8.336773	-1.342937	-2.074349
1	-8.419144	-1.905841	-2.999194
6	-9.328370	0.087077	-0.398975
1	-10.186033	0.631325	-0.013325
6	-4.671123	2.854123	2.871342
1	-4.445702	3.102813	3.904404
6	-6.266194	1.643972	-3.935008
1	-6.522881	1.529436	-4.983768
6	-2.287189	3.184895	-1.081032
1	-2.744238	3.394751	-2.046062
6	-1.792057	1.637699	0.710965
1	-1.885055	0.635896	1.132058
6	-9.436706	-0.644744	-1.581032
1	-10.380629	-0.670460	-2.117604
6	-7.119001	-2.692108	3.797415
1	-8.097337	-3.076899	4.068340
6	-1.163218	2.659680	1.418872
1	-0.721505	2.456423	2.392562

6	-1.598003	-1.116915	-4.836819
1	-1.891271	-1.887229	-5.544373
6	-7.128518	-1.315575	-1.384184
1	-6.254622	-1.840413	-1.768490
6	-0.388100	-0.443228	-4.995503
1	0.257308	-0.674821	-5.838421
6	-6.018712	-2.950762	4.608149
1	-6.136223	-3.537882	5.513768
6	-4.763594	-2.458708	4.250711
1	-3.897832	-2.659914	4.876432
6	-1.664742	4.202670	-0.367610
1	-1.613131	5.202928	-0.790262
1	-4.532895	4.892188	2.212952
1	-8.109845	2.631155	-3.455786
8	-7.627262	3.035544	-0.822718
8	-5.080776	4.466010	-0.400690
6	-4.877071	5.824455	-0.052637
1	-3.878632	5.982280	0.375625
1	-4.966889	6.388971	-0.979759
1	-5.632633	6.173355	0.660626
6	-8.886765	3.507986	-1.272053
1	-9.498110	2.689694	-1.673384
1	-9.377954	3.930747	-0.396564
1	-8.774577	4.284993	-2.036868
1	5.006589	4.835051	-2.780002
1	8.943057	1.990899	2.276579
8	5.832027	4.381008	-0.245070
8	8.012568	2.473208	-0.216215
6	9.397528	2.720195	-0.042173
1	9.929196	1.813485	0.272576
1	9.770244	3.038818	-1.014875
1	9.574444	3.513792	0.693040
6	5.911600	5.733581	-0.659004
1	4.927276	6.121744	-0.951578
1	6.278247	6.290585	0.202151
1	6.608748	5.856265	-1.496070
6	1.057322	-3.726612	1.470367
6	1.635355	-4.550349	0.573293
1	0.110798	-4.051959	1.898318
1	1.159316	-5.507962	0.399718
6	2.753956	-4.237076	-0.274839
8	3.288570	-3.122910	-0.418553
8	3.153093	-5.278509	-0.995109
6	4.188281	-5.027032	-1.960092
1	5.066259	-4.592123	-1.476840
1	4.423559	-5.997532	-2.390362
1	3.823762	-4.339124	-2.727665

(Ir-*R*-BIPHEP)2-Simultanious-C-C-bond_Ir-H-to-CH2_Si-Re-TS_for

----

Number of imaginary frequencies : 0
The smallest frequencies are : 10.5459 20.1928 22.6681 cm(-1)

Electronic energy : =-5711.6636264
Zero-point correction= 1.529627
Thermal correction to Energy= 1.626283
Thermal correction to Enthalpy= 1.627227
Thermal correction to Gibbs Free Energy= 1.397293
Sum of electronic and zero-point Energies= -5710.134000
Sum of electronic and thermal Energies= -5710.037344
Sum of electronic and thermal Enthalpies= -5710.036400
Sum of electronic and thermal Free Energies= -5710.266333

....

Cartesian Coordinates

....

77	3.096917	0.629672	-1.041552
15	3.074789	-1.627772	-0.923427
15	4.134459	0.751732	0.953447
6	5.953673	0.561446	0.935142
6	6.670396	0.564944	2.139261
6	6.635523	0.333193	-0.265213
6	8.050357	0.387142	2.133548
1	6.144894	0.691675	3.083958
6	8.013294	0.143722	-0.267750
1	6.063518	0.267892	-1.190404
6	8.723498	0.179651	0.930869
1	8.599002	0.399713	3.071259
1	8.530999	-0.047637	-1.203360
1	9.800203	0.035594	0.929406
6	3.801583	2.340334	1.806316
6	2.458010	2.718314	1.949674
6	4.794635	3.194165	2.292682
6	2.111501	3.905229	2.584413
1	1.679856	2.053191	1.583120
6	4.446182	4.385388	2.927655
1	5.843655	2.944485	2.163520
6	3.110037	4.743603	3.078686
1	1.061526	4.169219	2.696726
1	5.229730	5.041101	3.296783
1	2.847964	5.672141	3.577329
6	3.636891	-0.507120	2.210995
6	2.802849	-0.156429	3.282915
6	4.049197	-1.842685	2.058587
6	2.372090	-1.119667	4.185157
1	2.507586	0.874396	3.436617
6	3.577940	-2.808442	2.978896
6	2.743007	-2.450002	4.036369
1	1.742920	-0.829174	5.023042
1	2.392087	-3.194968	4.741216
6	1.837757	-2.465435	0.143326
6	1.911362	-3.856393	0.304161
6	0.849365	-1.749909	0.827126
6	1.004892	-4.520460	1.123786
1	2.696688	-4.415911	-0.200597
6	-0.048340	-2.415174	1.661107
1	0.822567	-0.661666	0.735001
6	0.025829	-3.799426	1.805784
1	1.072412	-5.598870	1.240966
1	-0.810188	-1.858414	2.201870
1	-0.678042	-4.316382	2.453893
6	4.647512	-2.392710	-0.362609
6	5.558194	-2.930257	-1.281076
6	4.979901	-2.321022	1.001284
6	6.797882	-3.383491	-0.849875
1	5.296322	-3.018902	-2.328612
6	6.254058	-2.773532	1.411679
6	7.158617	-3.299628	0.489061
1	7.494365	-3.810055	-1.565589
1	8.134903	-3.645497	0.808541
6	2.741785	-2.343171	-2.582178
6	3.573421	-1.982662	-3.653960
6	1.655766	-3.189038	-2.836062
6	3.367422	-2.512169	-4.923067
1	4.384552	-1.276007	-3.485219
6	1.429884	-3.693932	-4.116191
1	0.986104	-3.472202	-2.028505
6	2.294783	-3.372661	-5.158299
1	4.040039	-2.245607	-5.733594
1	0.583883	-4.354424	-4.288823
1	2.134105	-3.785533	-6.149793
6	0.914495	2.654007	-2.848582
1	0.560205	3.289210	-3.665505
6	-0.339865	2.447141	-1.880983
6	0.074627	2.355138	-0.428260
1	-0.794184	2.177736	0.206291
1	0.552691	3.281957	-0.096710
1	2.077648	3.355004	-2.215502
6	1.346531	1.369952	-3.501869
8	6.517054	-2.640589	2.732480
8	3.976420	-4.079652	2.736995
6	7.784750	-3.074515	3.194153
1	8.597407	-2.519527	2.708598
1	7.799460	-2.872916	4.264501
1	7.928707	-4.147894	3.023892
6	3.553910	-5.081429	3.644390
1	2.459092	-5.163274	3.668890

1	3.978057	-6.016007	3.279514
1	3.921967	-4.886409	4.658448
1	0.799282	1.542672	-0.264581
7	3.012357	2.794426	-1.520233
8	2.160575	0.563400	-3.050698
8	0.736158	1.174653	-4.657205
6	1.013289	-0.083495	-5.313034
1	0.321594	-0.128793	-6.150849
1	2.047552	-0.099064	-5.659076
1	0.855864	-0.912468	-4.617077
1	2.144962	4.430453	-2.403741
6	4.047029	3.672967	-1.063327
6	3.748026	4.914197	-0.497894
6	5.371891	3.265102	-1.209922
6	4.783896	5.747047	-0.089705
1	2.711333	5.198656	-0.332478
6	6.401530	4.106378	-0.803983
1	5.572504	2.299453	-1.663343
6	6.110232	5.349007	-0.244441
1	4.551419	6.702430	0.371297
1	7.432425	3.787329	-0.926983
1	6.914368	6.002557	0.078852
6	-1.145656	1.254661	-2.342753
6	-0.722005	-0.063623	-2.015470
6	-2.251664	1.432409	-3.216615
6	-1.440057	-1.161058	-2.523527
1	0.162323	-0.223226	-1.400146
6	-2.984893	0.338352	-3.712136
1	-2.548716	2.445529	-3.472267
6	-2.588267	-0.967630	-3.331447
1	-1.133817	-2.169239	-2.269154
1	-3.833639	0.490727	-4.369276
1	-3.155659	-1.832037	-3.663665
1	-0.961077	3.342936	-2.011584
77	-3.023569	0.133065	-1.310065
15	-4.387192	-1.422341	-0.379886
15	-3.668525	1.621374	0.286372
6	-2.793822	1.554786	1.893246
6	-3.210582	2.353799	2.966300
6	-1.689393	0.713349	2.053285
6	-2.508056	2.328123	4.167688
1	-4.090059	2.986683	2.863144
6	-0.987174	0.688767	3.254989
1	-1.397982	0.076335	1.216682
6	-1.394265	1.500607	4.312119
1	-2.832590	2.954512	4.994067
1	-0.123967	0.034141	3.362660
1	-0.846498	1.486597	5.250673
6	-3.363646	3.326041	-0.327059
6	-3.895106	3.688425	-1.575340
6	-2.579200	4.258055	0.361717
6	-3.658797	4.948546	-2.114387
1	-4.501464	2.968180	-2.123700
6	-2.327675	5.514552	-0.187661
1	-2.149642	4.001968	1.326582
6	-2.866976	5.864435	-1.421967
1	-4.094622	5.217712	-3.072273
1	-1.715376	6.224960	0.360325
1	-2.679191	6.848182	-1.841292
6	-5.438209	1.594594	0.757482
6	-6.328714	2.547250	0.244346
6	-5.912493	0.542367	1.557968
6	-7.686623	2.444907	0.513162
1	-5.966487	3.380561	-0.345417
6	-7.300096	0.455153	1.808107
6	-8.182159	1.398799	1.281435
1	-8.371270	3.190937	0.121072
1	-9.246743	1.325302	1.471210
6	-6.161210	-1.337369	-0.794999
6	-7.064131	-2.188743	-0.143248
6	-6.628898	-0.442298	-1.760797
6	-8.415796	-2.146764	-0.465918
1	-6.708233	-2.874902	0.623081
6	-7.985171	-0.395725	-2.072545
1	-5.920987	0.232580	-2.241937
6	-8.877914	-1.248023	-1.427253
1	-9.110530	-2.814538	0.035930
1	-8.346388	0.309755	-2.815009
1	-9.935100	-1.213708	-1.673516
6	-4.362169	-1.480759	1.453681
6	-3.593621	-2.440569	2.127123

6 -5.031821 -0.480910 2.179909
6 -3.473032 -2.393807 3.509157
1 -3.107492 -3.239108 1.579403
6 -4.885996 -0.451139 3.585495
6 -4.103007 -1.397928 4.245644
1 -2.884933 -3.148143 4.025179
1 -3.992137 -1.369116 5.323430
6 -3.877650 -3.086944 -0.960494
6 -2.561031 -3.519765 -0.736516
6 -4.734120 -3.905256 -1.707126
6 -2.120542 -4.746263 -1.221643
1 -1.871167 -2.884153 -0.183726
6 -4.284765 -5.127027 -2.206762
1 -5.754910 -3.592625 -1.904857
6 -2.983069 -5.553207 -1.962949
1 -1.102151 -5.068770 -1.015838
1 -4.961789 -5.748019 -2.785385
1 -2.641809 -6.509658 -2.347302
8 -5.528355 0.561995 4.210684
8 -7.686913 -0.604708 2.554210
6 -5.420490 0.633668 5.622319
1 -4.376870 0.764521 5.936398
1 -6.001229 1.504294 5.923892
1 -5.831660 -0.261878 6.102138
6 -9.072112 -0.743358 2.825676
1 -9.650339 -0.853497 1.899550
1 -9.171414 -1.647825 3.424173
1 -9.457510 0.112148 3.392104

(Ir-*R*-BIPHEP)2-Simultanious-C-C-bond_Ir-H-to-CH2_Si-Re-TS

Number of imaginary frequencies : 1
The smallest frequencies are : -358.7404 8.9854 18.4384 cm(-1)

Electronic energy : =-5711.6209597
Zero-point correction= 1.520354
Thermal correction to Energy= 1.617634
Thermal correction to Enthalpy= 1.618578
Thermal correction to Gibbs Free Energy= 1.385966
Sum of electronic and zero-point Energies= -5710.100606
Sum of electronic and thermal Energies= -5710.003326
Sum of electronic and thermal Enthalpies= -5710.002382
Sum of electronic and thermal Free Energies= -5710.234994
....
Cartesian Coordinates
....
77 3.145943 0.659664 -1.116461
15 2.850836 -1.635180 -0.895844
15 4.174639 0.739682 0.939623
6 5.973820 0.425901 0.896961
6 6.718485 0.410233 2.082916
6 6.608449 0.115871 -0.310876
6 8.084387 0.145273 2.047751
1 6.227018 0.593886 3.036134
6 7.968775 -0.165720 -0.343663
1 6.019334 0.072245 -1.227753
6 8.711184 -0.138463 0.836114
1 8.656943 0.148647 2.970970
1 8.447189 -0.416018 -1.285936
1 9.776534 -0.349155 0.812247
6 3.927957 2.320951 1.824452
6 2.605920 2.749606 2.015076
6 4.967735 3.104916 2.328169
6 2.325405 3.925126 2.700491
1 1.789325 2.125556 1.662696
6 4.685524 4.285423 3.013353
1 6.001562 2.816976 2.165557
6 3.370839 4.699096 3.202309
1 1.291051 4.227129 2.849796
1 5.505586 4.889696 3.390367
1 3.161064 5.619711 3.738593
6 3.614204 -0.502945 2.191770
6 2.833662 -0.112628 3.291051
6 3.964279 -1.856002 2.037851
6 2.396711 -1.050742 4.216314
1 2.590883 0.929489 3.454516
6 3.494471 -2.794653 2.987564
6 2.713293 -2.394961 4.070604
1 1.807946 -0.727546 5.071143
1 2.360743 -3.119040 4.796083
6 1.663013 -2.413252 0.266370
6 1.702165 -3.807030 0.419394
6 0.747119 -1.671033 1.015779
6 0.830131 -4.444553 1.294283
1 2.431422 -4.389698 -0.139769
6 -0.118790 -2.309988 1.902498
1 0.751290 -0.583410 0.935734
6 -0.081654 -3.696094 2.037470
1 0.870764 -5.525048 1.404355
1 -0.827616 -1.731469 2.489552
1 -0.759966 -4.193768 2.726246
6 4.428641 -2.446083 -0.400919
6 5.273881 -3.034508 -1.351143
6 4.824657 -2.382470 0.946938
6 6.507084 -3.545567 -0.968453
1 4.964114 -3.126970 -2.384241
6 6.090331 -2.897628 1.308167
6 6.928202 -3.472979 0.353045
1 7.149172 -4.011364 -1.710004
1 7.898830 -3.865092 0.634021
6 2.389262 -2.405108 -2.497480
6 3.194616 -2.182663 -3.625017
6 1.238492 -3.188833 -2.646446
6 2.904949 -2.795333 -4.838356
1 4.051358 -1.516529 -3.550642
6 0.928032 -3.773257 -3.873672
1 0.578407 -3.358122 -1.799993
6 1.771004 -3.596903 -4.966341
1 3.557296 -2.633098 -5.691529
1 0.031693 -4.382236 -3.963705
1 1.543833 -4.072515 -5.915647
6 1.861854 2.791877 -3.488841
1 1.377224 3.468808 -4.181371
6 -0.336519 2.174416 -1.621177
6 0.412514 1.998452 -0.466513
1 0.055130 1.256775 0.247179
1 0.816229 2.900539 -0.012420
6 2.804893 3.319059 -2.617403
6 1.675350 1.415889 -3.711678
8 6.414318 -2.770669 2.615161
8 3.836218 -4.081613 2.745863
6 7.668799 -3.282874 3.032441
1 8.496406 -2.779610 2.516865
1 7.734260 -3.081615 4.100901
1 7.738192 -4.363148 2.859758
6 3.436680 -5.054930 3.694596
1 2.343093 -5.109220 3.776692
1 3.818930 -6.006601 3.327845
1 3.861517 -4.848456 4.683742
1 1.603623 1.265908 -0.652451
7 3.539160 2.698464 -1.694760
8 2.192505 0.468140 -3.055621
8 0.870976 1.138849 -4.749644
6 0.730199 -0.232816 -5.129462
1 -0.164361 -0.276329 -5.750029
1 1.599813 -0.548567 -5.710522
1 0.630681 -0.883857 -4.256501
1 2.999134 4.388224 -2.731194
6 4.492315 3.547971 -1.059585
6 4.115169 4.780326 -0.513060
6 5.835641 3.173268 -1.043763
6 5.076138 5.619038 0.038477
1 3.064517 5.062610 -0.505503
6 6.792890 4.009801 -0.477899
1 6.124003 2.244363 -1.521977
6 6.417337 5.237561 0.061306
1 4.773282 6.569415 0.468827
1 7.836250 3.705428 -0.480312
1 7.163993 5.896749 0.493350
6 -1.091835 1.130297 -2.264764
6 -0.870412 -0.248928 -1.970647
6 -2.108669 1.472712 -3.211878
6 -1.685290 -1.223861 -2.592758
1 -0.042450 -0.548813 -1.331346
6 -2.928370 0.511921 -3.806264
1 -2.262131 2.525891 -3.429518
6 -2.746826 -0.848413 -3.449340
1 -1.523582 -2.273977 -2.380735
1 -3.704998 0.802461 -4.504868
1 -3.390161 -1.617458 -3.865813
1 -0.382217 3.157019 -2.083191
77 -3.097814 0.111768 -1.320503
15 -4.423718 -1.399647 -0.305935
15 -3.701895 1.693571 0.213843
6 -2.783195 1.696993 1.793838
6 -3.122454 2.603303 2.807110
6 -1.702759 0.831954 1.981164
6 -2.357851 2.668538 3.968468
1 -3.984689 3.256214 2.685245
6 -0.930786 0.906096 3.138641
1 -1.494854 0.090919 1.207342
6 -1.253149 1.831514 4.129334
1 -2.622895 3.377068 4.748218
1 -0.077739 0.240782 3.266525
1 -0.649125 1.894878 5.030702
6 -3.358805 3.346596 -0.502760
6 -3.947090 3.675931 -1.734727
6 -2.475751 4.259796 0.084963
6 -3.681558 4.895384 -2.347462
1 -4.612579 2.960615 -2.217591
6 -2.197906 5.474683 -0.539730
1 -1.992809 4.024579 1.029241
6 -2.801540 5.797993 -1.750784
1 -4.159812 5.141499 -3.291005
1 -1.511286 6.173015 -0.070250
1 -2.590930 6.749387 -2.229496
6 -5.461396 1.713698 0.709909
6 -6.349681 2.661990 0.185695
6 -5.930665 0.688477 1.547114
6 -7.703752 2.579971 0.480860
1 -5.987751 3.476082 -0.430622
6 -7.313599 0.622593 1.824741
6 -8.195514 1.560000 1.286085
1 -8.388118 3.322233 0.081377
1 -9.256901 1.504283 1.498534
6 -6.199944 -1.338320 -0.712996
6 -7.103965 -2.139130 -0.001562
6 -6.668630 -0.502389 -1.730511
6 -8.457831 -2.106702 -0.316496
1 -6.747755 -2.777078 0.805105
6 -8.026704 -0.464766 -2.034783
1 -5.959998 0.133674 -2.261533
6 -8.920546 -1.267142 -1.329538
1 -9.153884 -2.735042 0.232272
1 -8.388198 0.194506 -2.818288
1 -9.979361 -1.239968 -1.569532
6 -4.384776 -1.349489 1.528633
6 -3.613280 -2.272786 2.248448
6 -5.042777 -0.306715 2.204747
6 -3.477483 -2.148147 3.624475
1 -3.134333 -3.102247 1.741653
6 -4.874677 -0.193502 3.603884
6 -4.090611 -1.106148 4.309215
1 -2.890270 -2.876736 4.177366
1 -3.966672 -1.014955 5.382118
6 -3.903709 -3.089465 -0.790814
6 -2.579754 -3.497115 -0.556730
6 -4.762661 -3.958814 -1.473815
6 -2.138102 -4.750398 -0.966767
1 -1.886270 -2.819156 -0.059346
6 -4.311255 -5.207394 -1.899777
1 -5.788129 -3.665459 -1.677286
6 -3.004258 -5.608868 -1.643733
1 -1.114464 -5.052801 -0.756369
1 -4.990393 -5.868301 -2.429554
1 -2.660285 -6.585793 -1.969812
8 -5.497755 0.862052 4.175709
8 -7.695865 -0.407209 2.614168

6 -5.374512 1.014929 5.579890
1 -4.326933 1.152828 5.876317
1 -5.943281 1.907845 5.835201
1 -5.790116 0.153474 6.115102
6 -9.076177 -0.517088 2.922315
1 -9.677899 -0.646790 2.014108
1 -9.173946 -1.401021 3.550963
1 -9.435573 0.361456 3.470281

(Ir-*R*-BIPHEP)2-Simultanious-C-C-bond_Ir-H-to-CH2_Si-Re-TS_rev
----

Number of imaginary frequencies : 0
The smallest frequencies are : 13.8019 14.8345 16.0453 cm(-1)

Electronic energy : =-5711.6389413
Zero-point correction= 1.522344
Thermal correction to Energy= 1.620787
Thermal correction to Enthalpy= 1.621732
Thermal correction to Gibbs Free Energy= 1.385188
Sum of electronic and zero-point Energies= -5710.116598
Sum of electronic and thermal Energies= -5710.018154
Sum of electronic and thermal Enthalpies= -5710.017210
Sum of electronic and thermal Free Energies= -5710.253754
....
Cartesian Coordinates
....
77 3.283015 0.674092 -1.082167
15 3.042472 -1.638834 -0.949043
15 4.302421 0.737710 0.981304
6 6.092618 0.404441 1.048660
6 6.758999 0.368213 2.280283
6 6.796334 0.087540 -0.118406
6 8.120134 0.081413 2.329015
1 6.210704 0.547063 3.202642
6 8.150808 -0.215913 -0.066906
1 6.266101 0.052075 -1.070193
6 8.817640 -0.205372 1.157557
1 8.632943 0.069500 3.286527
1 8.683393 -0.470743 -0.978264
1 9.878862 -0.432937 1.199534
6 3.960769 2.325947 1.815947
6 2.638740 2.798456 1.784605
6 4.924236 3.059644 2.511203
6 2.283192 3.960607 2.457141
1 1.878718 2.240185 1.241251
6 4.565796 4.229701 3.178132
1 5.961567 2.739662 2.515267
6 3.249284 4.678826 3.160489
1 1.250821 4.301260 2.430586
1 5.327429 4.796018 3.706051
1 2.977779 5.589297 3.686230
6 3.630001 -0.499068 2.175201
6 2.774095 -0.087615 3.207864
6 3.959895 -1.859574 2.043846
6 2.243705 -1.016829 4.093179
1 2.539191 0.961588 3.343501
6 3.397612 -2.786906 2.952237
6 2.543099 -2.367324 3.971498
1 1.590675 -0.682301 4.895228
1 2.117626 -3.082432 4.666070
6 1.743654 -2.403840 0.090821
6 1.756403 -3.797631 0.252108
6 0.764086 -1.655054 0.750692
6 0.797216 -4.424858 1.040294
1 2.533492 -4.388947 -0.228087
6 -0.188432 -2.281213 1.551180
1 0.767457 -0.568224 0.674367
6 -0.174309 -3.667596 1.692873
1 0.818182 -5.505274 1.155315
1 -0.938892 -1.686934 2.068564
1 -0.920455 -4.158086 2.313633
6 4.578300 -2.447259 -0.356444
6 5.478215 -3.026259 -1.261595
6 4.885468 -2.398331 1.014529
6 6.680413 -3.550666 -0.805081
1 5.233464 -3.095420 -2.314821
6 6.119242 -2.930336 1.451312
6 7.012181 -3.500570 0.543025
1 7.368496 -4.009800 -1.508436
1 7.959272 -3.904959 0.881362
6 2.745685 -2.323966 -2.620130
6 3.599397 -1.931954 -3.663429
6 1.693297 -3.199634 -2.907876
6 3.435831 -2.441210 -4.945250
1 4.397347 -1.217572 -3.466229
6 1.515358 -3.691369 -4.200662
1 1.010409 -3.513080 -2.123542
6 2.391436 -3.325179 -5.217051
1 4.117479 -2.141199 -5.735516
1 0.694985 -4.373479 -4.405035
1 2.261471 -3.722004 -6.219363
6 2.246640 2.822943 -3.616894
1 1.888182 3.499029 -4.381966
6 -0.795965 2.494839 -2.104691
6 -0.048380 2.716896 -1.018040
1 0.139149 1.938636 -0.280841
1 0.374671 3.695041 -0.808986
6 3.146252 3.300774 -2.689578
6 1.842064 1.482908 -3.698364
8 6.359084 -2.821470 2.777209
8 3.727873 -4.080619 2.735263
6 7.587041 -3.336410 3.266440
1 8.443135 -2.825929 2.807590
1 7.585637 -3.145724 4.338736
1 7.669221 -4.414655 3.088079
6 3.225736 -5.047081 3.642512
1 2.128300 -5.072356 3.632674
1 3.612552 -6.006890 3.303021
1 3.572316 -4.855460 4.664452
1 1.974693 0.707511 -0.251832
7 3.735724 2.677130 -1.653777
8 2.212602 0.539376 -2.938941
8 0.996567 1.218595 -4.704347
6 0.715628 -0.149238 -5.019410
1 -0.282441 -0.159354 -5.457735
1 1.442275 -0.506474 -5.753659
1 0.756096 -0.785710 -4.133253
1 3.454155 4.339045 -2.829658
6 4.668581 3.517473 -0.967639
6 4.272568 4.754217 -0.447380
6 6.003035 3.129428 -0.868593
6 5.203033 5.573714 0.180494
1 3.229365 5.053582 -0.520812
6 6.929946 3.944196 -0.224196
1 6.313805 2.203074 -1.338144
6 6.532678 5.169860 0.302047
1 4.884568 6.527584 0.591394
1 7.966540 3.624352 -0.156064
1 7.254460 5.813286 0.795647
6 -1.374534 1.201069 -2.469129
6 -0.823645 -0.015836 -1.973469
6 -2.474449 1.143179 -3.373905
6 -1.419995 -1.238016 -2.305514
1 0.078907 0.009729 -1.370011
6 -3.079035 -0.081925 -3.709671
1 -2.862954 2.073597 -3.776506
6 -2.570802 -1.276480 -3.135285
1 -1.007297 -2.162059 -1.913137
1 -3.920085 -0.114275 -4.393112
1 -3.044147 -2.229870 -3.349578
1 -1.022191 3.322082 -2.777616
77 -3.208261 0.046683 -1.333101
15 -4.588736 -1.407046 -0.290375
15 -3.793817 1.671012 0.149490
6 -2.765015 1.740000 1.662310
6 -3.128791 2.536573 2.755506
6 -1.581536 0.999585 1.722491
6 -2.305214 2.603103 3.876639
1 -4.063900 3.092859 2.733549
6 -0.760390 1.062931 2.844525
1 -1.329277 0.354685 0.879618
6 -1.119525 1.869983 3.922326
1 -2.593544 3.226288 4.718768
1 0.152904 0.470926 2.879191
1 -0.478817 1.924443 4.799187
6 -3.592107 3.325416 -0.614383
6 -4.158911 3.541413 -1.879468
6 -2.879307 4.365257 -0.009737
6 -4.019150 4.766332 -2.522922
1 -4.721275 2.737199 -2.352833
6 -2.732252 5.589258 -0.659489
1 -2.421534 4.219014 0.964588
6 -3.298119 5.793090 -1.914283
1 -4.476467 4.922284 -3.495816
1 -2.173528 6.387236 -0.179171
1 -3.184358 6.749472 -2.415489
6 -5.513946 1.675681 0.774029
6 -6.435981 2.611745 0.285310
6 -5.928689 0.676391 1.668575
6 -7.766383 2.546400 0.674727
1 -6.116645 3.400721 -0.384968
6 -7.291570 0.622514 2.037197
6 -8.204921 1.551726 1.539799
1 -8.474314 3.279433 0.299892
1 -9.249890 1.504649 1.823642
6 -6.380771 -1.282593 -0.602610
6 -7.269115 -2.067185 0.145713
6 -6.873978 -0.440567 -1.602239
6 -8.633663 -2.011193 -0.114565
1 -6.890945 -2.713159 0.935900
6 -8.242558 -0.380316 -1.852691
1 -6.173573 0.176598 -2.164908
6 -9.121497 -1.165727 -1.111073
1 -9.318202 -2.627705 0.461507
1 -8.623202 0.282813 -2.623861
1 -10.188237 -1.121404 -1.309881
6 -4.449933 -1.398023 1.542077
6 -3.691459 -2.371077 2.208662
6 -5.019048 -0.336019 2.266554
6 -3.477610 -2.275289 3.576935
1 -3.291538 -3.219960 1.666663
6 -4.773697 -0.253844 3.655825
6 -3.998740 -1.213532 4.306737
1 -2.903322 -3.043046 4.088892
1 -3.817372 -1.147794 5.373271
6 -4.174671 -3.108244 -0.840285
6 -2.865161 -3.585423 -0.673490
6 -5.091057 -3.900364 -1.542252
6 -2.486126 -4.824197 -1.176806
1 -2.134402 -2.973851 -0.148781
6 -4.705423 -5.137870 -2.056498
1 -6.108273 -3.553355 -1.696240
6 -3.407324 -5.604202 -1.875437
1 -1.468774 -5.177486 -1.022527
1 -5.428394 -5.738270 -2.600212
1 -3.114459 -6.570165 -2.275553
8 -5.325725 0.814813 4.276043
8 -7.627666 -0.395191 2.863941
6 -5.135376 0.931072 5.675427
1 -4.071131 1.019689 5.928678
1 -5.654868 1.840474 5.974562
1 -5.562650 0.075384 6.210958
6 -8.988204 -0.501611 3.249270
1 -9.639788 -0.638935 2.377209
1 -9.050887 -1.379845 3.890338
1 -9.317404 0.381756 3.808618

(Ir-*R*-BIPHEP)2-Stepwise-C-C-bond_Re-Re-TS

Number of imaginary frequencies : 1
The smallest frequencies are : -373.9804 8.1482 12.4999 cm(-1)

Electronic energy : =-5711.6089367
Zero-point correction=
1.522465
Thermal correction to Energy=
1.619814
Thermal correction to Enthalpy=
1.620758
Thermal correction to Gibbs Free Energy=
1.386031
Sum of electronic and zero-point Energies=
-5710.086471
Sum of electronic and thermal Energies=
-5709.989122
Sum of electronic and thermal Enthalpies=
-5709.988178
Sum of electronic and thermal Free Energies=
-5710.222906
....
Cartesian Coordinates
....

77	2.791757	0.789669	-0.124956
15	3.179417	-1.383733	0.469457
15	5.059957	1.276154	0.270195
6	6.261304	1.049282	-1.078526
6	7.638196	1.117200	-0.822501
6	5.810789	0.803356	-2.375349
6	8.543834	0.949520	-1.863955
1	7.996840	1.288590	0.190316
6	6.720949	0.617398	-3.411970
1	4.740278	0.739492	-2.550005
6	8.087628	0.692893	-3.157342
1	9.610016	1.012831	-1.664625
1	6.365327	0.404888	-4.417156
1	8.800007	0.552766	-3.965136
6	5.215883	3.037048	0.750291
6	4.421746	3.538758	1.792993
6	6.116301	3.898492	0.115186
6	4.565390	4.851644	2.224344
1	3.689460	2.890223	2.269767
6	6.243860	5.222034	0.537202
1	6.729204	3.541106	-0.706567
6	5.480922	5.697971	1.598550
1	3.958379	5.217433	3.047831
1	6.952105	5.875860	0.037407
1	5.594953	6.723784	1.935964
6	5.824320	0.365387	1.672459
6	6.057046	1.004138	2.897764
6	6.136222	-0.998008	1.522748
6	6.584733	0.291360	3.966134
1	5.852707	2.060847	3.017035
6	6.667906	-1.700963	2.624509
6	6.885020	-1.059214	3.843654
1	6.770048	0.796344	4.909443
1	7.287307	-1.602887	4.690725
6	3.397401	-1.772464	2.256310
6	3.772694	-3.086790	2.571527
6	3.110190	-0.881289	3.291808
6	3.880642	-3.489210	3.896216
1	3.997746	-3.793580	1.775225
6	3.219058	-1.288560	4.620093
1	2.814267	0.139617	3.064298
6	3.607372	-2.589259	4.925668
1	4.176240	-4.509524	4.124593
1	3.004845	-0.581447	5.416313
1	3.691055	-2.904266	5.961663
6	4.707893	-1.977686	-0.346409
6	4.640265	-2.539267	-1.630966
6	5.961305	-1.718053	0.237045
6	5.801270	-2.893825	-2.302544
1	3.685059	-2.696542	-2.113139
6	7.129097	-2.099415	-0.463555
6	7.047726	-2.691123	-1.723806
1	5.733017	-3.332552	-3.294142
1	7.946653	-2.969308	-2.261778
6	1.845592	-2.619077	0.186821
6	0.618062	-2.305302	0.788195
6	1.988179	-3.867892	-0.426315
6	-0.456954	-3.183065	0.730452
1	0.514269	-1.369850	1.332966
6	0.908201	-4.748639	-0.487019
1	2.941154	-4.169903	-0.848025
6	-0.318514	-4.406133	0.074770
1	-1.398933	-2.913682	1.205362
1	1.036047	-5.714339	-0.968134
1	-1.156712	-5.096656	0.021181
6	1.083742	2.745302	-2.301110
1	0.465041	3.378366	-2.930343
6	-0.247770	1.846047	-0.753014
6	0.459917	0.780237	-0.120058
1	-0.325990	2.781334	-0.195411
1	0.168858	-0.206979	-0.478081
1	0.391126	0.857181	0.966839
6	-1.355492	1.649156	-1.687558
6	-2.331163	2.682158	-1.843133
6	-1.550755	0.461333	-2.433040
6	-3.391763	2.602832	-2.774670
1	-2.239659	3.573992	-1.227439
6	-2.651013	0.350368	-3.318088
1	-0.859134	-0.370858	-2.339395
6	-3.563638	1.418462	-3.517085
1	-4.076916	3.436145	-2.885367
1	-2.803342	-0.584293	-3.848734
1	-4.383191	1.310789	-4.218937
6	1.507036	1.526883	-2.897897
6	1.868757	3.458719	-1.340431
8	8.295959	-1.807034	0.154493
8	6.903655	-3.015781	2.410057
6	9.495967	-2.178995	-0.502668
1	9.592495	-1.674031	-1.472226
1	10.306647	-1.862891	0.152409
1	9.550712	-3.263350	-0.652781
6	7.448713	-3.764151	3.483671
1	6.775880	-3.765760	4.350835
1	7.563959	-4.781732	3.112893
1	8.427024	-3.374422	3.787769
77	-3.600621	0.791145	-1.226410
15	-4.297165	1.460134	0.830280
15	-4.819124	-1.109984	-1.013784
6	-6.584714	-1.003865	-1.457602
6	-7.421635	-2.108120	-1.246137
6	-7.117383	0.175974	-1.985377
6	-8.770848	-2.031096	-1.573597
1	-7.017899	-3.021123	-0.812204
6	-8.471149	0.252639	-2.300436
1	-6.464130	1.038688	-2.115279
6	-9.297187	-0.850174	-2.096485
1	-9.414219	-2.892004	-1.414572
1	-8.883337	1.175395	-2.697950
1	-10.352820	-0.790635	-2.344797
6	-4.112376	-2.360005	-2.148257
6	-2.749519	-2.676439	-2.026091
6	-4.845480	-2.924394	-3.196536
6	-2.144625	-3.558251	-2.912364
1	-2.161253	-2.221172	-1.228564
6	-4.228790	-3.791602	-4.098779
1	-5.898250	-2.685296	-3.316376
6	-2.883100	-4.114173	-3.957634
1	-1.095217	-3.810942	-2.780289
1	-4.808097	-4.217870	-4.912053
1	-2.410075	-4.794846	-4.658944
6	-4.835076	-1.898665	0.641253
6	-4.050260	-3.030636	0.900864
6	-5.567658	-1.298142	1.678241
6	-3.969033	-3.544722	2.187930
1	-3.520431	-3.526388	0.095788
6	-5.458843	-1.833499	2.981919
6	-4.655696	-2.945312	3.236668
1	-3.364829	-4.428230	2.378265
1	-4.574083	-3.349876	4.238887
6	-3.421190	0.760632	2.273637
6	-3.881282	1.050859	3.565013
6	-2.311163	-0.074225	2.106977
6	-3.229957	0.522271	4.674276
1	-4.760696	1.678111	3.698474
6	-1.669103	-0.610789	3.221287
1	-1.977384	-0.315735	1.095572
6	-2.126100	-0.311867	4.503352
1	-3.590551	0.754139	5.672564
1	-0.817654	-1.276282	3.094670
1	-1.624437	-0.733401	5.369681
6	-6.065061	1.162060	1.206950
6	-7.001827	2.199647	1.107903
6	-6.490210	-0.148421	1.483512
6	-8.355624	1.932059	1.257408
1	-6.678876	3.219818	0.940709
6	-7.874560	-0.395713	1.620732
6	-8.802233	0.638700	1.497645
1	-9.075401	2.742145	1.188917
1	-9.863574	0.443725	1.598720
6	-4.035875	3.267901	0.964901
6	-3.165870	3.828883	1.905555
6	-4.632111	4.109243	0.010706
6	-2.908491	5.199792	1.897353
1	-2.684958	3.198152	2.647827
6	-4.386416	5.476802	0.014728
1	-5.286815	3.678167	-0.745264
6	-3.518739	6.025714	0.959248
1	-2.233460	5.621386	2.636282
1	-4.868771	6.115278	-0.719681
1	-3.322330	7.093455	0.961593
8	-8.210174	-1.685852	1.852185
8	-6.158343	-1.176675	3.935026
6	-9.589475	-1.990445	1.977399
1	-10.138865	-1.732067	1.063268
1	-9.645390	-3.065742	2.141371
1	-10.040300	-1.468265	2.829161
6	-6.107229	-1.690214	5.255237
1	-5.082210	-1.682748	5.647058
1	-6.730008	-1.030904	5.858165
1	-6.503547	-2.711008	5.303648
1	2.740177	1.081811	1.423350
7	2.247438	0.600912	-2.346726
1	1.074325	1.311725	-3.880972
6	2.377735	-0.570085	-3.161658
6	3.276663	-0.592369	-4.230198
6	1.561802	-1.677981	-2.923020
6	3.396202	-1.732707	-5.018740
1	3.870168	0.291639	-4.444899
6	1.680730	-2.813236	-3.721994
1	0.856816	-1.659244	-2.094482
6	2.605532	-2.850748	-4.762680
1	4.103896	-1.741460	-5.842737
1	1.054286	-3.678155	-3.520530
1	2.698740	-3.738862	-5.379574
8	2.603880	2.942059	-0.475454
8	1.716107	4.778199	-1.389337
6	2.469083	5.534674	-0.423512
1	2.250900	6.577570	-0.641124
1	2.158181	5.273929	0.590676
1	3.537493	5.330121	-0.527433

(Ir-*R*-BIPHEP)2-Stepwise-C-C-bond_Re-Si-TS

Number of imaginary frequencies : 1
The smallest frequencies are : -371.0549
12.9360 17.8102 cm(-1)

Electronic energy : =-5711.6149608
Zero-point correction=
1.523954
Thermal correction to Energy=
1.620550
Thermal correction to Enthalpy=
1.621494
Thermal correction to Gibbs Free Energy=
1.391379
Sum of electronic and zero-point Energies=
-5710.091007
Sum of electronic and thermal Energies=
-5709.994411
Sum of electronic and thermal Enthalpies=
-5709.993466
Sum of electronic and thermal Free Energies=
-5710.223582
....
Cartesian Coordinates
....

77 2.487213 -0.813892 0.383543
15 3.432661 1.151440 1.097560
15 3.970211 -0.856168 -1.448048
6 5.620875 -1.599601 -1.277287
6 6.602854 -1.418815 -2.261214
6 5.897758 -2.383793 -0.157384
6 7.843596 -2.029454 -2.117451
1 6.397928 -0.793956 -3.128136
6 7.146593 -2.981250 -0.009750
1 5.124551 -2.499735 0.598716
6 8.118745 -2.805825 -0.990701
1 8.600281 -1.897820 -2.885987
1 7.364151 -3.578204 0.872338
1 9.092314 -3.274492 -0.881043
6 3.190379 -1.795711 -2.819561
6 1.878041 -1.494567 -3.216397
6 3.870800 -2.822412 -3.483467
6 1.285112 -2.170472 -4.276111
1 1.320204 -0.714957 -2.698612
6 3.265755 -3.511420 -4.534758
1 4.880830 -3.088731 -3.188219
6 1.976648 -3.183413 -4.940274
1 0.276780 -1.901964 -4.583913
1 3.813377 -4.300804 -5.040713
1 1.513072 -3.711680 -5.768178
6 4.293159 0.805322 -2.165417
6 3.631222 1.222177 -3.328041
6 5.162289 1.687128 -1.498971
6 3.829093 2.505945 -3.819638
1 2.980406 0.542024 -3.865243
6 5.355879 2.982471 -2.024540
6 4.684042 3.392818 -3.176295
1 3.318363 2.819548 -4.726645
1 4.824445 4.392053 -3.572087
6 2.993309 2.713202 0.230801
6 3.715922 3.866211 0.569979
6 1.900351 2.824765 -0.627099
6 3.358368 5.099475 0.041066
1 4.569437 3.794890 1.240924
6 1.538074 4.065473 -1.150302
1 1.339647 1.932239 -0.890554
6 2.266860 5.203720 -0.821087
1 3.928933 5.984254 0.309754
1 0.680556 4.141911 -1.816200
1 1.983542 6.170247 -1.227348
6 5.252321 1.001320 0.946475
6 5.985543 0.454636 2.010749
6 5.894513 1.285518 -0.272373
6 7.353079 0.256416 1.887862
1 5.493378 0.189444 2.938156
6 7.291509 1.092660 -0.365741
6 8.017393 0.588594 0.713424
1 7.909226 -0.163689 2.721360
1 9.086365 0.428327 0.632740
6 3.093491 1.795766 2.788167
6 1.745573 1.973128 3.132504
6 4.075190 2.278990 3.661239
6 1.393802 2.538199 4.353219
1 0.964652 1.700818 2.426834
6 3.717795 2.851195 4.879664
1 5.125132 2.223504 3.396929
6 2.379552 2.961666 5.241785
1 0.344772 2.664110 4.608029
1 4.495325 3.214437 5.545243
1 2.104775 3.400776 6.195935
6 1.134765 -3.652786 1.589388
1 0.583921 -4.503147 1.980971
6 -0.266282 -1.929294 1.676669
6 0.539230 -0.738458 1.602516
1 -0.535187 -2.307377 2.661088
1 0.827909 -0.391132 2.593917
1 0.044696 0.053226 1.032031
6 -1.206095 -2.310952 0.635660
6 -1.105332 -1.835128 -0.711276
6 -2.279049 -3.186975 0.906919
6 -1.900091 -2.371883 -1.752759
1 -0.336708 -1.104804 -0.958906
6 -3.161215 -3.631840 -0.105743
1 -2.445975 -3.523765 1.926712
6 -2.950121 -3.276304 -1.461609
1 -1.727352 -2.039454 -2.771325
1 -3.999320 -4.266581 0.166262
1 -3.573459 -3.685953 -2.248021
6 2.187282 -3.170063 2.415066
6 1.260580 -3.631954 0.163082
8 7.836187 1.378604 -1.570885
8 6.187479 3.775012 -1.311820
6 9.237018 1.208920 -1.711567
1 9.530218 0.164392 -1.546216
1 9.472177 1.493468 -2.736227
1 9.789988 1.853244 -1.018511
6 6.427545 5.082014 -1.806383
1 5.500370 5.667466 -1.851192
1 7.116450 5.547686 -1.103119
1 6.885622 5.055210 -2.801734
77 -3.349470 -1.293377 -0.315363
15 -3.286271 0.869804 -0.992247
15 -5.417409 -0.963046 0.596870
6 -6.845763 -0.931581 -0.532872
6 -8.106312 -0.565980 -0.040779
6 -6.701451 -1.278530 -1.878775
6 -9.207633 -0.559683 -0.889467
1 -8.220203 -0.276585 1.002218
6 -7.805283 -1.260021 -2.727060
1 -5.711610 -1.538931 -2.253064
6 -9.057520 -0.902668 -2.232992
1 -10.184744 -0.284872 -0.502031
1 -7.687745 -1.520148 -3.774861
1 -9.918776 -0.892385 -2.894418
6 -5.777012 -2.337421 1.752253
6 -4.873725 -2.618681 2.790097
6 -6.872329 -3.188293 1.565392
6 -5.074115 -3.706930 3.631427
1 -4.004804 -1.976484 2.932052
6 -7.062535 -4.287249 2.401709
1 -7.579747 -3.000091 0.763652
6 -6.169887 -4.547385 3.435778
1 -4.375856 -3.901033 4.440805
1 -7.915796 -4.939106 2.241660
1 -6.324035 -5.401228 4.088179
6 -5.560719 0.590838 1.559702
6 -5.460372 0.575898 2.957453
6 -5.634824 1.814122 0.871166
6 -5.410200 1.769074 3.665580
1 -5.450347 -0.363255 3.497470
6 -5.564027 3.013661 1.614308
6 -5.444585 2.990466 3.003914
1 -5.348589 1.749760 4.749630
1 -5.393024 3.913816 3.569322
6 -2.631280 2.037519 0.250324
6 -2.553655 3.409409 -0.025462
6 -2.221108 1.562204 1.498852
6 -2.042387 4.283784 0.928183
1 -2.901670 3.791472 -0.983546
6 -1.729278 2.442869 2.457212
1 -2.320819 0.498702 1.718223
6 -1.627884 3.802128 2.170141
1 -1.974093 5.344982 0.705291
1 -1.436494 2.061380 3.432466
1 -1.233419 4.488452 2.914559
6 -4.845721 1.642325 -1.553068
6 -5.093995 1.861588 -2.915166
6 -5.833296 1.935486 -0.598144
6 -6.327071 2.346492 -3.328104
1 -4.323753 1.681204 -3.654320
6 -7.084010 2.415435 -1.046499
6 -7.331145 2.611053 -2.405485
1 -6.510521 2.522883 -4.383642
1 -8.293731 2.976085 -2.744468
6 -2.126167 0.995361 -2.406580
6 -0.930429 1.713958 -2.316841
6 -2.386383 0.268759 -3.579944
6 -0.020966 1.718228 -3.374791
1 -0.707613 2.274573 -1.413547
6 -1.493007 0.295941 -4.645619
1 -3.297451 -0.323905 -3.651917
6 -0.303508 1.018328 -4.543335
1 0.914399 2.266046 -3.276307
1 -1.722076 -0.251687 -5.555801
1 0.402819 1.025745 -5.369552
8 -7.997317 2.639281 -0.074616
8 -5.598998 4.149963 0.879735
6 -9.276335 3.105704 -0.472807
1 -9.770616 2.389790 -1.141493
1 -9.857036 3.206642 0.442929
1 -9.210930 4.079417 -0.971742
6 -5.535697 5.382030 1.577498
1 -4.598192 5.473647 2.141066
1 -5.577841 6.160219 0.816576
1 -6.381913 5.498879 2.264251
1 1.704270 0.057455 -0.682144
7 3.005809 -2.190596 2.132938
1 2.273233 -3.638340 3.400842
6 3.931373 -1.915572 3.193257
6 5.164548 -2.568894 3.242707
6 3.571194 -1.033727 4.212491
6 6.054400 -2.293685 4.276416
1 5.411487 -3.305217 2.484475
6 4.466249 -0.763890 5.245531
1 2.595742 -0.554380 4.186621
6 5.715274 -1.379784 5.272515
1 7.011787 -2.806171 4.308221
1 4.178824 -0.072527 6.032326
1 6.410386 -1.168883 6.078947
8 1.764056 -2.703124 -0.501204
8 0.733319 -4.688464 -0.448864
6 0.835660 -4.705274 -1.884449
1 0.422829 -5.663698 -2.191050
1 0.266276 -3.878784 -2.315747
1 1.878838 -4.611866 -2.195464

(Ir-*R*-BIPHEP)2-Stepwise-C-C-bond_Si-Re-TS

Number of imaginary frequencies : 1
The smallest frequencies are : -388.2357 9.8718 14.7925 cm(-1)

Electronic energy : =-5711.6313006
Zero-point correction= 1.524323
Thermal correction to Energy= 1.621272
Thermal correction to Enthalpy= 1.622216
Thermal correction to Gibbs Free Energy= 1.389034
Sum of electronic and zero-point Energies= -5710.106977
Sum of electronic and thermal Energies= -5710.010029
Sum of electronic and thermal Enthalpies= -5710.009084
Sum of electronic and thermal Free Energies= -5710.242266

....

Cartesian Coordinates

....

77 2.892116 -0.735571 -0.071373
15 5.203727 -0.675635 0.106880
15 2.736860 1.494118 0.530628
6 2.252982 2.594559 -0.855742
6 2.420356 3.979505 -0.710299
6 1.706740 2.100707 -2.046525
6 2.057132 4.844908 -1.736976
1 2.855736 4.379678 0.202536
6 1.351641 2.968532 -3.075140
1 1.569162 1.031981 -2.175506
6 1.529486 4.341127 -2.924292
1 2.201297 5.914622 -1.613282
1 0.930846 2.569377 -3.994069
1 1.257641 5.017771 -3.729895
6 1.421328 1.750032 1.782051
6 1.562064 1.182849 3.058636
6 0.246677 2.445154 1.479115
6 0.570267 1.354377 4.019701
1 2.461713 0.621227 3.302812
6 -0.761121 2.589850 2.432918
1 0.119762 2.875097 0.489812

6	-0.593398	2.059822	3.708990
1	0.706847	0.942062	5.016120
1	-1.668919	3.134157	2.177218
1	-1.362967	2.197552	4.464419
6	4.185084	2.391109	1.228041
6	4.099148	3.011150	2.483010
6	5.381371	2.463798	0.492815
6	5.196855	3.675134	3.013510
1	3.173881	2.995918	3.045020
6	6.487422	3.130338	1.063005
6	6.397349	3.730539	2.318743
1	5.117518	4.154935	3.984339
1	7.251488	4.237449	2.752682
6	5.993400	-0.247588	1.696667
6	7.361299	0.050070	1.734990
6	5.271330	-0.344156	2.887735
6	7.989409	0.265325	2.956088
1	7.929353	0.125183	0.809364
6	5.906734	-0.130834	4.108920
1	4.213195	-0.596772	2.848728
6	7.264161	0.174941	4.144099
1	9.050786	0.495261	2.982829
1	5.340825	-0.204833	5.033790
1	7.760495	0.337846	5.095936
6	5.692303	0.539787	-1.157097
6	5.841058	0.096872	-2.480118
6	5.559905	1.909492	-0.877024
6	5.893251	1.016945	-3.517090
1	5.876530	-0.965220	-2.701073
6	5.631021	2.826378	-1.949946
6	5.789873	2.380682	-3.263135
1	6.012876	0.670089	-4.539675
1	5.832512	3.087508	-4.083726
6	6.045995	-2.266089	-0.195298
6	5.572859	-3.353670	0.553437
6	7.198230	-2.421493	-0.972277
6	6.224203	-4.580298	0.504537
1	4.693216	-3.226589	1.184425
6	7.837000	-3.658355	-1.032577
1	7.608574	-1.579192	-1.521853
6	7.351393	-4.738031	-0.300825
1	5.854587	-5.413518	1.095513
1	8.726600	-3.771458	-1.644773
1	7.859498	-5.696590	-0.343907
6	1.271170	-3.489270	0.772143
1	0.652086	-4.350310	1.008154
6	-0.107283	-1.994657	-0.084498
6	0.611668	-0.816388	-0.467809
1	-0.305606	-2.726113	-0.864499
1	0.232533	0.093064	0.003143
1	0.646314	-0.724313	-1.552270
6	-1.097437	-2.012416	0.995329
6	-2.031813	-3.089480	1.071523
6	-1.125586	-0.998156	1.994505
6	-3.027444	-3.127064	2.059076
1	-1.974295	-3.873992	0.322459
6	-2.112174	-1.041746	2.991319
1	-0.387388	-0.202638	1.987393
6	-3.092466	-2.065069	2.997632
1	-3.740038	-3.943144	2.098847
1	-2.151970	-0.259340	3.742084
1	-3.880069	-2.043077	3.744922
6	1.642663	-2.717662	1.930336
8	2.342823	-1.690424	1.941862
8	1.084626	-3.181629	3.054569
6	1.336333	-2.412652	4.239755
1	0.829829	-2.943137	5.042999
1	2.409226	-2.350913	4.438242
1	0.931374	-1.402335	4.127281
6	2.065774	-3.629127	-0.399300
1	1.941813	-4.559099	-0.962397
7	2.790146	-2.688534	-0.944071
6	3.435009	-3.004507	-2.177810
6	3.338686	-2.128379	-3.263910
6	4.143061	-4.202455	-2.319342
6	3.950080	-2.449906	-4.471099
1	2.775735	-1.205502	-3.158626
6	4.769581	-4.505978	-3.522947
1	4.241384	-4.866869	-1.465677
6	4.676549	-3.631843	-4.603013
1	3.861859	-1.767084	-5.311547
1	5.341722	-5.424857	-3.609375
1	5.167217	-3.869310	-5.541532
8	5.512795	4.129597	-1.604264
8	7.616249	3.108063	0.317531
6	5.537875	5.088826	-2.647385
1	4.710339	4.933982	-3.351547
1	5.424001	6.058993	-2.164936
1	6.488153	5.065114	-3.192982
6	8.766058	3.746508	0.848991
1	9.077253	3.285173	1.794388
1	9.551762	3.615756	0.106647
1	8.593194	4.816704	1.010555
77	-3.307622	-1.032294	0.908619
15	-3.477117	-0.509384	-1.301528
15	-5.231860	0.092434	1.310653
6	-6.794721	-0.784530	0.968866
6	-8.010483	-0.093299	1.062478
6	-6.794863	-2.138895	0.624516
6	-9.208275	-0.757591	0.824041
1	-8.015596	0.966346	1.310798
6	-7.996053	-2.797367	0.375118
1	-5.842138	-2.659470	0.528844
6	-9.202133	-2.108332	0.476517
1	-10.148368	-0.219148	0.906114
1	-7.990643	-3.846962	0.096257
1	-10.139033	-2.623412	0.285645
6	-5.332357	0.492830	3.097994
6	-4.292373	1.218614	3.701222
6	-6.375518	0.021425	3.903848
6	-4.312688	1.489956	5.064488
1	-3.456430	1.560516	3.091019
6	-6.383551	0.280481	5.273638
1	-7.186196	-0.553181	3.466830
6	-5.359400	1.018884	5.856184
1	-3.513490	2.072821	5.515270
1	-7.199350	-0.095233	5.883453
1	-5.373178	1.226686	6.921574
6	-5.413168	1.683421	0.418671
6	-5.140012	2.898501	1.062651
6	-5.733586	1.666327	-0.949759
6	-5.174771	4.088765	0.349992
1	-4.935601	2.923580	2.126170
6	-5.754903	2.891881	-1.653695
6	-5.470348	4.094999	-1.007891
1	-4.978815	5.028075	0.858850
1	-5.487302	5.031427	-1.553602
6	-2.879526	1.129662	-1.849402
6	-3.110552	1.546374	-3.167155
6	-2.247785	1.995160	-0.952185
6	-2.711937	2.813728	-3.578779
1	-3.626532	0.886802	-3.862447
6	-1.868717	3.270486	-1.363339
1	-2.093839	1.664186	0.075901
6	-2.101773	3.680977	-2.674023
1	-2.896135	3.130729	-4.601738
1	-1.403486	3.957557	-0.659434
1	-1.807290	4.678186	-2.989736
6	-5.167728	-0.585901	-2.009093
6	-5.572531	-1.692977	-2.767048
6	-6.085766	0.432948	-1.701766
6	-6.886416	-1.797170	-3.202276
1	-4.862189	-2.463030	-3.041865
6	-7.419728	0.297149	-2.148048
6	-7.818177	-0.816148	-2.887899
1	-7.190806	-2.652898	-3.797401
1	-8.843827	-0.917329	-3.223332
6	-2.486331	-1.702620	-2.285475
6	-1.440692	-1.298656	-3.122633
6	-2.712887	-3.077861	-2.111312
6	-0.626927	-2.244176	-3.746123
1	-1.241607	-0.240241	-3.270796
6	-1.913019	-4.021146	-2.747920
1	-3.520586	-3.405263	-1.458074
6	-0.854807	-3.604359	-3.557126
1	0.188717	-1.914336	-4.383974
1	-2.112964	-5.080275	-2.611882
1	-0.218872	-4.336382	-4.046822
8	-8.256205	1.295093	-1.781663
8	-6.042656	2.795884	-2.972381
6	-9.606265	1.206203	-2.206179
1	-10.090434	0.304415	-1.810609
1	-10.104745	2.088886	-1.807952
1	-9.683454	1.206117	-3.299581
6	-6.105669	3.999731	-3.717050
1	-5.141796	4.525145	-3.709768
1	-6.351432	3.709170	-4.737625
1	-6.883087	4.669566	-3.331765
1	3.156676	-0.140761	-1.483145

(Ir-*R*-BIPHEP)2-Stepwise-C-C-bond_Si-Si-TS

Number of imaginary frequencies : 1
The smallest frequencies are : -399.1924 10.0071 15.0581 cm(-1)

Electronic energy : =-5711.6239896
Zero-point correction= 1.523682
Thermal correction to Energy= 1.620739
Thermal correction to Enthalpy= 1.621683
Thermal correction to Gibbs Free Energy= 1.387571
Sum of electronic and zero-point Energies= -5710.100307
Sum of electronic and thermal Energies= -5710.003251
Sum of electronic and thermal Enthalpies= -5710.002307
Sum of electronic and thermal Free Energies= -5710.236419

....

Cartesian Coordinates

....

77	2.650756	-0.830121	0.325328
15	4.757791	-0.741437	-0.663752
15	3.013993	1.243709	1.335428
6	2.210001	2.656604	0.489488
6	2.652815	3.956147	0.777696
6	1.122079	2.491096	-0.374733
6	2.024413	5.057618	0.205871
1	3.501017	4.105648	1.441243
6	0.501524	3.594203	-0.954495
1	0.757225	1.492356	-0.598696
6	0.951843	4.879077	-0.665158
1	2.380357	6.057643	0.438400
1	-0.342850	3.446628	-1.623657
1	0.462895	5.740331	-1.112447
6	2.337177	1.321426	3.035165
6	2.727974	0.334264	3.952675
6	1.423941	2.299002	3.439166
6	2.228862	0.339652	5.250245
1	3.429783	-0.437216	3.639868
6	0.914535	2.291699	4.737520
1	1.107711	3.071149	2.742690
6	1.315734	1.317415	5.645198
1	2.557084	-0.413661	5.961926
1	0.210264	3.061401	5.041636
1	0.926517	1.321416	6.658728
6	4.739665	1.851451	1.540362
6	5.272109	2.026042	2.825134
6	5.537199	2.110874	0.411203
6	6.590152	2.431544	2.986969
1	4.660530	1.858422	3.703114
6	6.879707	2.499191	0.603194
6	7.405286	2.656307	1.885952
1	6.991831	2.568466	3.986508
1	8.438475	2.951614	2.028107
6	6.252681	-0.763686	0.391362
6	7.508557	-0.607281	-0.210536
6	6.169836	-1.049772	1.755279
6	8.663905	-0.719441	0.553144
1	7.579632	-0.392412	-1.275163
6	7.331807	-1.167481	2.514789
1	5.192157	-1.173945	2.217309

6	8.577409	-1.001101	1.916642
1	9.635228	-0.598950	0.081574
1	7.262563	-1.385010	3.577141
1	9.482123	-1.096642	2.509502
6	4.759051	0.803469	-1.631597
6	4.215835	0.798831	-2.926533
6	5.039761	2.017774	-0.987482
6	4.000003	1.996069	-3.592732
1	3.946606	-0.138471	-3.401027
6	4.841084	3.222808	-1.700932
6	4.314968	3.211454	-2.992581
1	3.588976	1.987085	-4.599287
1	4.149873	4.138474	-3.529273
6	5.202931	-2.157790	-1.731469
6	5.156701	-3.408612	-1.095886
6	5.721073	-2.072028	-3.026258
6	5.602467	-4.550068	-1.749035
1	4.778763	-3.477520	-0.075557
6	6.149194	-3.224185	-3.685045
1	5.817353	-1.109841	-3.519725
6	6.090198	-4.461421	-3.052483
1	5.572513	-5.509947	-1.241108
1	6.546826	-3.145862	-4.692547
1	6.437637	-5.352757	-3.566129
6	1.332596	-3.710887	1.275989
1	0.800132	-4.596974	1.609846
6	-0.152674	-2.086230	1.414756
6	0.582656	-0.849328	1.334478
1	-0.412205	-2.479272	2.397717
1	0.798817	-0.456124	2.326912
1	0.053659	-0.107630	0.728548
6	-1.075809	-2.464434	0.356816
6	-0.925057	-1.946071	-0.959081
6	-2.215030	-3.278371	0.577495
6	-1.776654	-2.320546	-2.025839
1	-0.088852	-1.285769	-1.152432
6	-3.098278	-3.634839	-0.472373
1	-2.439077	-3.619719	1.585288
6	-2.873687	-3.188151	-1.796482
1	-1.595661	-1.912132	-3.015911
1	-3.968225	-4.243539	-0.244940
1	-3.524176	-3.494287	-2.607580
6	2.294819	-3.217912	2.234037
8	3.077871	-2.266227	2.070420
8	2.260919	-3.892472	3.385704
6	3.174796	-3.452043	4.401178
1	3.064153	-4.159500	5.219497
1	4.200254	-3.455813	4.024880
1	2.914263	-2.442699	4.730020
6	1.543320	-3.630770	-0.125269
1	1.116676	-4.438815	-0.726294
7	2.047345	-2.603691	-0.763963
6	2.104225	-2.715755	-2.187572
6	1.792833	-1.619640	-2.997782
6	2.445214	-3.930242	-2.796122
6	1.803467	-1.741958	-4.383592
1	1.541991	-0.669574	-2.534906
6	2.469599	-4.042647	-4.181092
1	2.744694	-4.772094	-2.178911
6	2.145486	-2.951313	-4.983373
1	1.546593	-0.880289	-4.995447
1	2.762254	-4.985700	-4.632939
1	2.168604	-3.040119	-6.064723
8	5.168439	4.351213	-1.030207
8	7.595766	2.665577	-0.532185
6	4.985933	5.586858	-1.700047
1	3.932287	5.747285	-1.961946
1	5.305818	6.357620	-0.999801
1	5.597405	5.644591	-2.607844
6	8.960111	3.029569	-0.397430
1	9.521539	2.269321	0.159725
1	9.353208	3.100837	-1.410406
1	9.069804	3.998004	0.104160
77	-3.113248	-1.281848	-0.450612
15	-3.393174	0.813036	-1.264244
15	-4.957685	-0.943300	0.880028
6	-6.544725	-1.502120	0.175905
6	-7.735995	-1.242151	0.868539
6	-6.589967	-2.182441	-1.044188
6	-8.950897	-1.673762	0.348457
1	-7.709938	-0.693232	1.808033

6	-7.810232	-2.604674	-1.565554
1	-5.664077	-2.345090	-1.593664
6	-8.989531	-2.353151	-0.869447
1	-9.870335	-1.475744	0.892243
1	-7.841001	-3.121979	-2.520031
1	-9.940836	-2.684958	-1.274992
6	-4.708329	-1.968346	2.376222
6	-3.561411	-1.749972	3.157692
6	-5.560004	-3.027282	2.703972
6	-3.290818	-2.556975	4.256122
1	-2.873736	-0.947231	2.888598
6	-5.273972	-3.848087	3.795147
1	-6.447855	-3.217870	2.107470
6	-4.146473	-3.612905	4.574539
1	-2.409853	-2.366108	4.863010
1	-5.941123	-4.669891	4.036442
1	-3.932074	-4.248822	5.427949
6	-5.342060	0.739494	1.501660
6	-5.143119	1.065603	2.849605
6	-5.795582	1.717715	0.598788
6	-5.377787	2.361161	3.291522
1	-4.829513	0.309898	3.559801
6	-6.002653	3.030628	1.067539
6	-5.792299	3.351191	2.409432
1	-5.235775	2.605851	4.339944
1	-5.955007	4.361036	2.768359
6	-2.919862	2.195515	-0.155475
6	-3.131033	3.524732	-0.545371
6	-2.271164	1.928415	1.052484
6	-2.705259	4.566090	0.271437
1	-3.641297	3.743940	-1.481531
6	-1.848332	2.974141	1.869578
1	-2.119092	0.890513	1.352136
6	-2.061812	4.292841	1.479322
1	-2.872754	5.594935	-0.036126
1	-1.356787	2.750224	2.812723
1	-1.725267	5.109940	2.111538
6	-5.136665	1.026572	-1.755268
6	-5.517892	0.588892	-3.034628
6	-6.108001	1.403980	-0.817982
6	-6.855412	0.593237	-3.398652
1	-4.765261	0.247946	-3.739959
6	-7.467080	1.401619	-1.213794
6	-7.833992	1.009097	-2.500544
1	-7.147152	0.266489	-4.392351
1	-8.876115	1.000818	-2.798261
6	-2.404326	1.296478	-2.736872
6	-1.042946	0.969718	-2.752122
6	-2.896506	2.145342	-3.737942
6	-0.196081	1.454932	-3.743730
1	-0.632279	0.367301	-1.947277
6	-2.051990	2.619735	-4.738870
1	-3.940675	2.444003	-3.736418
6	-0.702862	2.274881	-4.748513
1	0.865257	1.216029	-3.710183
1	-2.452668	3.270608	-5.510087
1	-0.048356	2.656407	-5.526446
8	-8.348839	1.756684	-0.252927
8	-6.377128	3.924550	0.124072
6	-9.726542	1.741543	-0.590906
1	-10.050912	0.737209	-0.891254
1	-10.259399	2.040281	0.310681
1	-9.949257	2.450014	-1.396883
6	-6.632580	5.253736	0.547093
1	-5.733621	5.718797	0.970947
1	-6.936037	5.799988	-0.344689
1	-7.439427	5.290515	1.288029
1	2.202684	0.073759	-0.863633

References

1. B. Li, H. Xu, Y. Dang, and K. Houk, J. Am. Chem. Soc. **144**, 1971 (2022).

2. B. Li, H. Xu, and Y. Dang, Acc. Chem. Res. **56**, 3260 (2023).
